# Signatures of co-deregulated genes and their transcriptional regulators in colorectal cancer

**DOI:** 10.1038/s41540-020-00144-8

**Published:** 2020-07-31

**Authors:** Natalia Mastrogamvraki, Apostolos Zaravinos

**Affiliations:** 1 Department of Life Sciences, School of Sciences, European University Cyprus, 1516 Nicosia, Cyprus; 2 Department of Basic Medical Sciences, College of Medicine, Member of QU Health, Qatar University, Doha, Qatar

**Keywords:** Cancer, Virtual drug screening, Regulatory networks, Biochemical networks, Systems analysis

## Abstract

The deregulated genes in colorectal cancer (CRC) vary significantly across different studies. Thus, a systems biology approach is needed to identify the co-deregulated genes (co-DEGs), explore their molecular networks, and spot the major hub proteins within these networks. We reanalyzed 19 GEO gene expression profiles to identify and annotate CRC versus normal signatures, single-gene perturbation, and single-drug perturbation signatures. We identified the co-DEGs across different studies, their upstream regulating kinases and transcription factors (TFs). Connectivity Map was used to identify likely repurposing drugs against CRC within each group. The functional changes of the co-upregulated genes in the first category were mainly associated with negative regulation of transforming growth factor β production and glomerular epithelial cell differentiation; whereas the co-downregulated genes were enriched in cotranslational protein targeting to the membrane. We identified 17 hub proteins across the co-upregulated genes and 18 hub proteins across the co-downregulated genes, composed of well-known TFs (*MYC, TCF3, PML*) and kinases (*CSNK2A1, CDK1/4, MAPK14*), and validated most of them using GEPIA2 and HPA, but also through two signature gene lists composed of the co-up and co-downregulated genes. We further identified a list of repurposing drugs that can potentially target the co-DEGs in CRC, including camptothecin, neostigmine bromide, emetine, remoxipride, cephaeline, thioridazine, and omeprazole. Similar analyses were performed in the co-DEG signatures in single-gene or drug perturbation experiments in CRC. *MYC, PML, CDKs, CSNK2A1*, and *MAPKs* were common hub proteins among all studies. Overall, we identified the critical genes in CRC and we propose repurposing drugs that could be used against them.

## Introduction

Colorectal cancer (CRC) is the third most common cancer globally and among the most common causes of cancer-related deaths^1^. It is an extremely heterogeneous disease developing through multiple stages and genetic pathways, and consisting of several genetic and epigenetic modifications that lead to malignant transformation of the cells^2^. This heterogeneity is owing to clonal and subclonal mutations randomly dispersed throughout the genome, observed not only between patients, but also among cells within a single tumor mass^3,4^. In a recent study, it was shown that no DNA locus is wild type in every malignant cell within a tumor at the time of diagnosis^5^. Four consensus molecular CRC subtypes, each with distinguishing features, are now known to exist^6^. These, comprise of: (i) microsatellite instability (MSI) immune subtype, (ii) epithelial, with WNT, and MYC signaling activation; (iii) epithelial with metabolic dysregulation; and (iv) mesenchymal, characterized by a prominent activation in TGF-β, stromal invasion, and angiogenesis^6^.

Up to now, most efforts focus on finding differentially expressed genes (DEGs) between CRC and the phenotypically normal tissue. However, the reported DEGs vary from study to study, depending on the different methodologies or case numbers. One intriguing issue that has not as yet been considered, is the analysis of the genes and signaling pathways that are simultaneously deregulated across molecularly heterogenous subtypes of the disease. Challenging as it might seem, the analysis of pooled, diverse CRC data sets could reveal the commonly deregulated signaling pathways across these tumors. This information could be very useful for the treatment of what really should be viewed as many diseases.

Gene expression profiling is used to detect differences at the transcriptional level in CRC, aiming to find early biomarkers that could be used in the detection of the disease, or to be used as therapeutic targets^7–10^. There is a vast list of differentially expressed genes in CRC, all identified by various techniques, including qPCR, microarrays or RNA-seq^11–17^. Nevertheless, the sensitivity and specificity of these biomarkers is not always necessarily adequate; urging the need to identify an updated panel of genes that can be used as better diagnostic and preventive biomarkers, or as therapeutic targets. In addition, the co-deregulated genes (co-DEGs) across many colorectal tumors vary, have not been extensively investigated, and their role within signaling networks or their transcriptional regulatory mechanisms have been poorly investigated, thus far.

Collecting manually extracted gene expression signatures from the Gene Expression Omnibus (GEO) is highly useful. Such signatures have been applied for drug repurposing^18^, proposing new drugs against cancer^19,20^, and understanding the activity of others that have been approved^21^. The outcome of which gene expression signatures are deregulated in a disease, heavily depends on the computational methodology followed. The Characteristic direction (CD) is a relatively new geometrical multivariate approach to identify DEGs, and it can significantly improve the prioritization of DEGs in contrast to other algorithms^20,22^. Processing the library of integrated network-based cellular signatures (LINCS) L1000 data with the CD method has been proved to significantly improve the signal to noise compared with previous methods to compute L1000 signatures^23^.

In the present study, we hypothesized that there are “hubs”, i.e., well-connected nodes in the network of transcription factors and kinases, which are more centralized and have a stronger capacity of modulating adjacent genes, and can thus, be identified by looking at a large set of differentially expressed genes. Our goal was to identify these hubs and drugs or drug combinations that may be used to target their expression, with the underlying hypothesis that targeting their expression with drugs may result in new effective treatment regimes.

To explore this hypothesis, we annotated and extracted various gene expression signatures from the GEO. Our results demonstrate that our list of gene signatures can provide new insights, regarding the associations between co-DEGs, drugs and CRC.

## Results

Using a systems biology approach, we investigated the co-DEGs among several independent studies, and categorized them in three groups: (1) in CRC against the adjacent normal mucosa; (2) in CRC tissue or cell lines with a single-gene perturbation against the wild-type (*wt*) tissue or cells; and, (3) in CRC tissue or cell lines with a single-drug perturbation against non-treated tissue or cells. We concluded to such lists of co-DEGs within each of these categories, after stringent filtering and excluding the genes that were deregulated in a single study. These, contained 164 co-upregulated and 199 co-downregulated genes in CRC vs the normal colon; 275 co-upregulated and 173 co-downregulated genes after single-gene perturbation; and 255 co-upregulated and 257 co-downregulated genes after single-drug perturbation (Supplementary Table 1). For each category we identified the transcription factors (TFs), protein-protein interactions (PPIs), and kinases being accountable for the observed changes in the mRNA expression of these co-DEGs, the drugs that suppress or boost the co-DEGs, respectively, and finally the biological pathways in which the above-mentioned molecules are involved.

Next, we envisaged to identify which of these upstream regulators potentially deregulate the expression of the identified co-DEGs, and therefore lead to the formation of the transcription complex in the CRC genome. To this end, we identified the phosphorylation reactions possibly being carried out by upstream regulators, such as kinases, in each category. We then, analyzed the drugs that suppress the overexpressed genes, or those that help to enhance the expression of the underexpressed ones. The top 10 TFs and protein kinases from all categories of the co-DEGs were classified, based on the highest value of a combined score of the *p* value and *z* score.

### Co-DEGs in CRC vs the adjacent normal mucosa

**Fig. 1:**
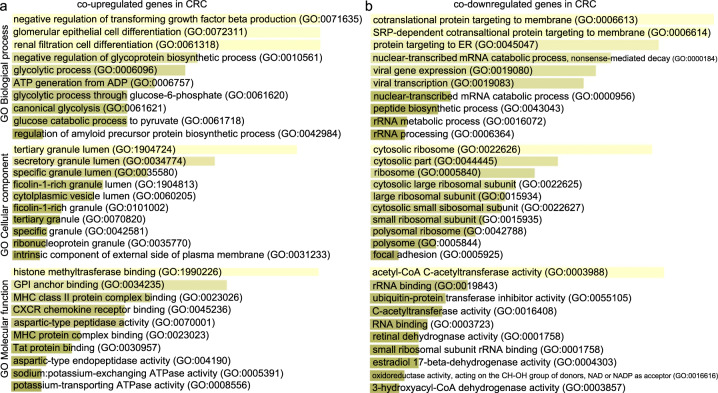
Gene Ontology (GO) enrichment analysis of the co-DEGs in CRC vs normal. **a** GO enrichment results of the co-upregulated genes in colorectal cancer against the normal mucosa. **b** GO enrichment results of the co-downregulated genes in colorectal cancer against the normal mucosa. Τhe bar graphs are sorted by the combined score. The length of each bar represents the significance of the corresponding term. The brighter the color of the bar, the more significant the corresponding term is.

We studied five independent GEO data sets to identify the co-DEGs in CRC tissues compared with their adjacent normal mucosa. We then obtained the GO annotations and KEGG pathways being significantly linked with the co-DEGs within each category. Our analysis showed that the co-upregulated genes in CRC are highly enriched in “negative regulation of TGFβ production”, “glomerular epithelial cell differentiation”, “renal filtration cell differentiation”, “negative regulation of glycoprotein biosynthetic process”, and “glycolytic process” (GO biological process). The disruption of TGFβ signaling is a major hit in CRC epithelial cells and host stromal cells, and we hypothesize that this term reflects best the epithelial tumors with activated WNT and MYC signaling pathways (consensus molecular subtype 2). The second and third terms reflect membranous nephropathy, which is the most common glomerular pathology among solid tumors, including CRC. It is also known that the abnormal WNT/β-catenin pathway and inflammation of the intestine lead to the epithelial breakdown of the intestine’s homeostasis. The other two terms reflect glycosylation aberrations, which are also present in CRC. For example, *LGALS1* (galectin-1) is hypermethylated in CRC cells. Its induction by demethylating agents induces apoptosis due to downregulation of the WNT signaling^24^. The co-upregulated genes in CRC are further enriched in “tertiary granule lumen”, “secretory granule lumen”, and “specific granule lumen” (GO cellular component); and “histone methyltransferase binding”, “GPI anchor binding”, and “MHC-II protein complex binding” (GO molecular function) (Fig. 1a and Supplementary Table 2). The enriched terms in the cellular component reflect the fact that colorectal tumorigenesis is the result of a progressive transformation of epithelial cells in the luminal surface of the intestinal tract to cancerous cells. The enriched terms pertaining to the molecular function of the co-upregulated genes reveal the important role that aberrant histone methylation plays in CRC. Histone methylation occurs on the side chains of lysine and arginine and is primarily mediated by histone methyltransferases; whereas, histone demethylases remove such methyl groups. The glycosylphosphatidylinositol (GPI) anchor is a glycan and lipid posttranslational modification added to proteins in the endoplasmic reticulum (ER). GPI-anchored proteins such as carcinoembryonic antigen and mesothelin, have been described as potential biomarkers in cancer^25^. MHC-II is responsible for presenting antigens to CD4+ T cells, the role of which is very important in antitumor immunity. We hypothesize that this term best reflects the MSI-immune subtype of CRC.

On the other hand, the co-downregulated genes in CRC were found to be enriched in “cotranslational protein targeting to membrane”, “SRP-dependent cotranslational protein targeting to membrane”, “protein targeting to ER”, and “nuclear-transcribed mRNA catabolic process, nonsense-mediated decay” (GO biological process). The intestinal epithelium is a professional secretory tissue and the downregulation of translational machinery and protein targeting to the ER is likely related to a loss of this function in CRC. The co-downregulated genes in CRC were also enriched in “cytosolic ribosome” (GO cellular component), reflecting its essential role for protein synthesis in all cells, and the promotion of tumorigenesis as a result of ribosome-related perturbations. Importantly, many ribosomal proteins are also known to be involved in other functions, as well; such as DNA replication, transcription and repair, RNA splicing and modification, cell growth and proliferation, regulation of apoptosis and development, and cellular transformation^26^. The co-downregulated genes in CRC were also enriched in “acetyl-CoA C-acetyltransferase activity”, among others (GO Molecular function) (Fig. 1b and Supplementary Table 2). The deregulation of metabolic pathways is a hallmark of cancer, and this term clearly reflects a dysfunctional lipid metabolism, in which fatty-acid imbalances are owing to defects in the long-chain acyl-CoA synthetases^27^.

**Fig. 2:**
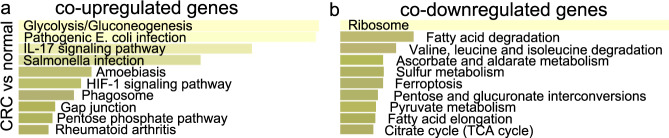
KEGG enrichment for the co-DEGs in CRC. KEGG enrichment for the co-upregulated genes **a** and the co-downregulated genes **b** in CRC vs the normal mucosa. Τhe bar graphs are sorted by the combined score. The length of each bar represents the significance of the corresponding term. The brighter the color of the bar, the more significant the corresponding term is.

KEGG enrichment analysis for the co-upregulated genes in CRC vs the normal mucosa, prioritized the pathways “glycolysis/gluconeogenesis”, “pathogenic *E. coli* infection”, “IL-17 signaling pathway”, and “salmonella infection”; whereas the co-downregulated genes participated mainly in “ribosome”, “fatty-acid degradation”, “valine, leucine, and isoleucine degradation” and “ascorbate and aldarate metabolism”, agreeing with the above-mentioned GO terms (Fig. 2a, b and Supplementary Table 3). The first term clearly reflects epithelial CRCs with a metabolic dysregulation (consensus molecular subtype 3). It is widely known that CRC cells reprogram their metabolism and shift from aerobic to anaerobic respiration even in the presence of oxygen, leading to anaerobic glycolysis (Warburg effect). This metabolic shift provides them an evolutionary advantage by providing increasing bioenergetics and biosynthesis.

Pathogenic *E. coli* infection is associated with inflammation in the gut and CRC. Similarly, Salmonella infection has a strong pathogenicity and contributes to chronic inflammation and carcinogenesis^28^. Therefore, both terms are implicated with an immune response in the colorectal tumor. Salmonella infection is further related to host cell transformation, by activating the WNT/β-catenin signaling pathway. It is also involved in stem cell maintenance through the regulation of the intestinal stem cell markers, Lgr5, and Bmi1^29^; and it can colonize the gut microbiota, resulting in dysbiosis^30^.

Interleukin-17 (IL-17) is a cytokine that promotes cancer-elicited inflammation and prevents cancer cells from immune surveillance. IL-17 is generally considered to be a promoter in CRC progression. Clearly the terms associates with the immune subtype of CRC.

Defects in the ribosomes (ribosomopathies) are well connected to cancer through two mechanisms. First, altered ribosomes may translate differentially specific mRNAs ultimately increasing the expression of oncogenes or reducing that of tumor suppressors^31,32^. In addition, ribosomal reduction can induce specific dysregulation in protein synthesis^33^. Second, the reduction of rRNA production or the lack/mutations of specific ribosomal proteins occurring in ribosomopathies lead to an excess production of ribosomal proteins, which are not incorporated into nascent ribosomes, but induce p53 stabilization^34^.

Fatty-acid degradation is currently considered a hallmark characteristic of CRC^35^. Alterations of lipid metabolism may lead to structural changes in their membranes, disruption of energy homeostasis, cell signaling, gene expression, and protein distribution^36–38^.

Valine, leucine, and isoleucine are essential branched amino acids the degradation of which might be owing to mutations in 3-hydroxyisobutyryl-CoA hydrolase (HIBCH). This enzyme converts 3-hydroxyisobutyryl-CoA to 3-hydroxyisobutyrate, which is further converted to succinyl-CoA and participates in the TCA cycle. HIBCH targeting could be used to treat CRC through the reprogramming of the metabolism of valine^39^.

Ascorbate and aldarate metabolism along with other metabolic pathways were just recently associated with CRC^40^, and obviously pertain to the epithelial subtype of tumors with evident metabolic dysregulation characteristics (consensus molecular subtype 3)^6^. Ascorbic acid (vitamin C) is synthesized by glutathione dehydrogenase in the cytosol, and shares GDP-sugar intermediates with cell-wall polysaccharide and glycoprotein synthesis. Ascorbate is involved in cell division and growth^41^.

Although molecular interaction pathways provide a potential molecular mechanistic interaction for the constituents, their coverage can be limited by the knowledge of biochemical interactions. PPI networks provide a basic abstraction of larger complex pathways that control the major cellular and molecular machinery determining the disease or healthy state of an organism. Within these networks, the hub proteins exhibiting a higher degree of interactions, are the key targets for drugging, in order to have a substantial effect on the cellular machinery^42^. However, we acknowledge that as these hub proteins are active in all cells, their drugging could also have adverse effects on non-malignant cells, as well. On the other hand, the proposed co-upregulated kinases (CSNK2A1, CDK1, MAPK14, CDK4, GSK3B, AKT1, CDK2, among others) could be more realistic druggable targets and, therefore, yield a significant clinical benefit for CRC patients. Furthermore, changes in gene expression are not necessarily the best way to find targets, as the mRNA levels and protein activity are not always well-correlated between them.

**Fig. 3:**
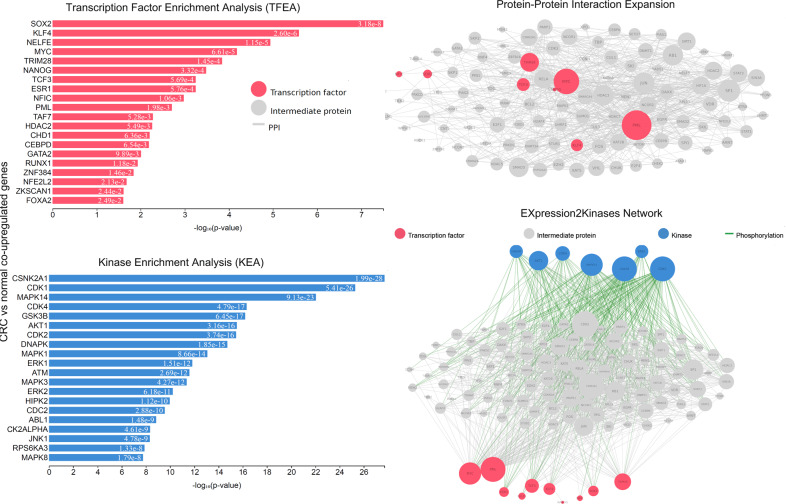
Upstream regulatory networks predicted to regulate the expression of the co-upregulated gene signatures in CRC vs the normal mucosa, as inferred from the Expression2Kinases (X2K) analysis. The inferred networks contain transcription factors (TFs, red nodes), intermediate proteins (gray nodes), and kinases (blue nodes). Gray edges indicate the interaction between two proteins (PPI) and green edges depict phosphorylation between a kinase and an intermediate protein or a TF. The size of nodes is relative to the level of expression degree. MYC and PML have higher k-core values and are hubs. These, are more centralized in the network and have a stronger capacity of modulating adjacent genes.

**Fig. 4:**
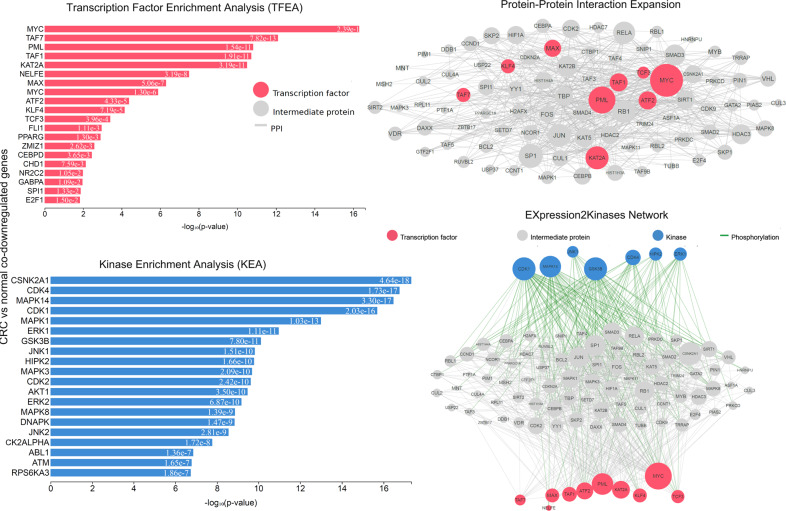
Upstream regulatory networks predicted to regulate the expression of the co-downregulated gene signatures in CRC vs the normal mucosa, as in Fig. 3. MYC, PML, KAT2A, and MAX have higher k-core values and are hubs. These, are more centralized in the network and have a stronger capacity of modulating adjacent genes.

To recognize such hub proteins, we constructed a PPI subnetwork around the proteins that the co-DEGs encode, and performed topological analysis. We identified 17 hub proteins across the co-upregulated genes (the TFs *SOX2, KLF4, NELFE, MYC, TRIM28, NANOG, TCF3, ESR1, NFIC, PML*, among others; and the kinases *CSNK2A1, CDK1, MAPK14, CDK4, GSK3B, AKT1, CDK2*, and *DNAPK*, among others; Supplementary Table 4) and of 18 hub proteins across the co-downregulated genes (the TFs *MYC, TAF7, PML, TAF1, KAT2A, NELFE, MAX, MYC, ATF2, KLF4*; and the kinases *CSNK2A1, CDK4, MAPK14, CDK1, MAPK1, ERK1, GSK3B, JNK1, HIPK2*, and *MAPK3*, among others; Supplementary Table 5) using degree and betweenness centrality metrics (Figs. 3, 4). These hub proteins are critical in the progression of CRC.

**Fig. 5:**
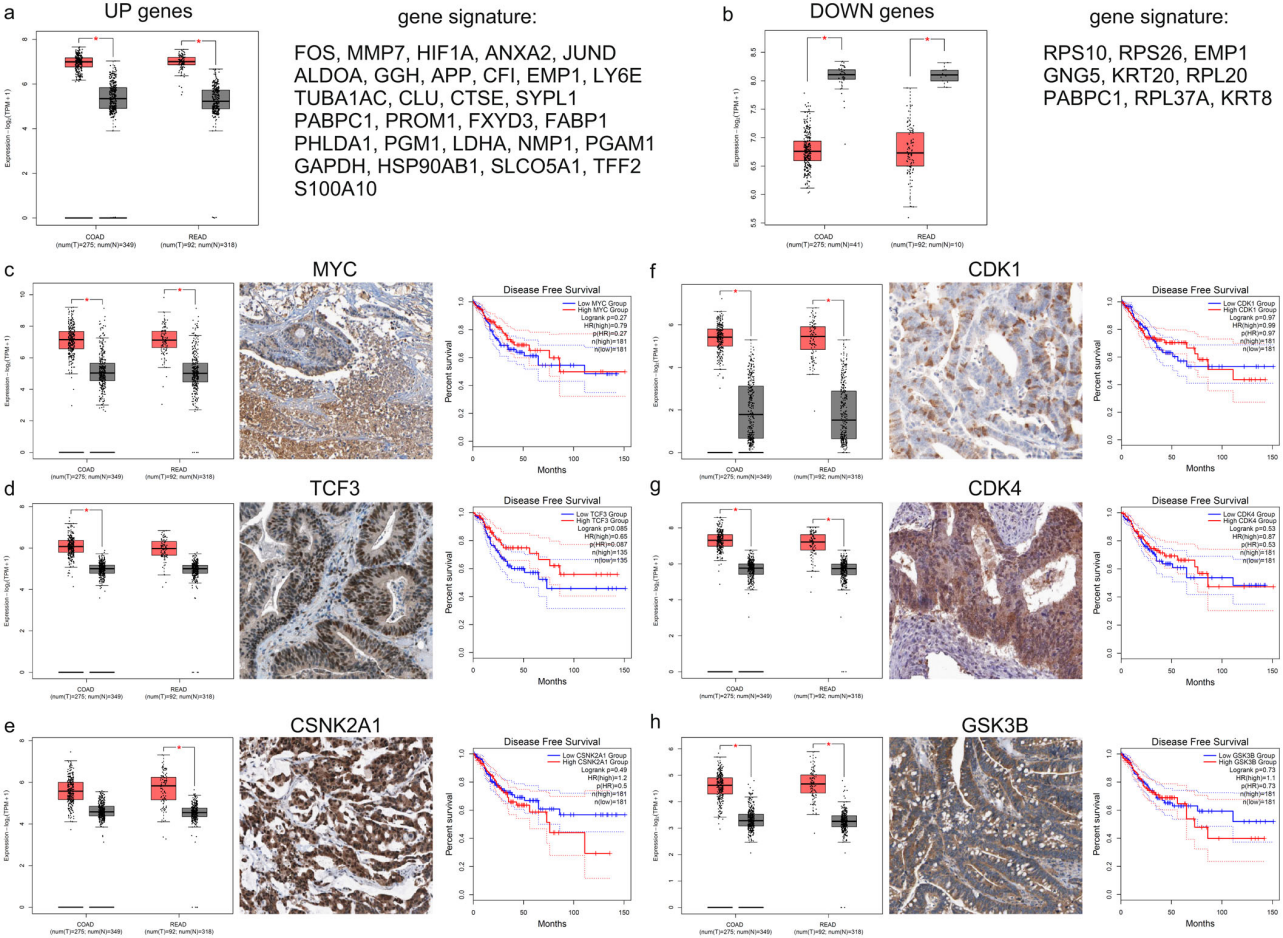
Signature gene lists of the co-DEGs in colon and rectum adenocarcinoma. The expression pattern of the co-upregulated **a** and co-downregulated **b** gene signatures was verified in the TCGA-COAD and TCGA-READ data sets, respectively. The significantly elevated expression of the major hub transcription factors, MYC and TCF3, were verified among COAD and READ tumors, respectively. MYC and TCF3 exhibited moderate protein expression. Higher levels of TCF3 shifted toward a better disease-free survival of the patients **c**–**d**. The upregulated levels of the hub kinases CSNK2A1, CDK1, CDK4, and GSK3B were also validated in COAD and READ Q7 tumors, but significant differences could be scored between low and high-expressing CRC patients **e**–**h**. The verification of the co-deregulated gene signatures was performed using GEPIA2 and HPA. The red boxes depict either colon (COAD) or rectum (READ) adenocarcinomas, termed as “T” (for tumor) in the *x* axis. Gray boxes depict the normal tissue samples used as controls (termed “N” in the x axis). The red stars denote statistical significance (*p* < 0.05), using the one-way ANOVA statistical test. GEPIA2 was also used for calculation of disease-free survival (Kaplan–Meier curves), using the Log-rank test and median values as cutoff. The cox proportional hazard ratio and the 95% confidence interval (dotted lines) are included in the survival plots. IHC images from tissue samples were derived from the Human Protein Atlas (HPA). The specifications of the antibodies used in IHC are provided in the Materials and Methods. The line in each boxplot marks the median of the data. The middle box represents the middle 50% of the values in each group (from lower quartile to upper quartile). Upper and lower whiskers represent scores outside the middle 50%.

We then constructed two signature gene lists composed of the co-upregulated (“UP genes”) and co-downregulated (“DOWN genes”) genes in colon and rectum adenocarcinoma, matching the normal tissue data from TCGA with those from the GTEx project, and validated them in the COAD and READ TCGA data sets. Indeed, the “UP genes” signature was significantly upregulated in CRC against the adjacent normal tissue (Fig. 5a). Likewise, the “DOWN genes” signature was significantly downregulated in both CRC subtypes (Fig. 5b).

The mRNA levels of specific hub genes were verified using Gene Expression Profiling Interactive Analysis (GEPIA2)^43^. We also validated the protein expression of the hub genes in tissue microarray-based immunohistochemistry (IHC) data from the Human Protein Atlas (HPA)^44^ and correlated them with the clinical outcome of the CRC patients. Overall, 275 COAD and 92 READ patients were available in GEPIA2 for the disease-free survival analysis, and a total of 349 normal colon and 318 normal rectum mucosa samples from both the TCGA and GTEx platforms were used for comparison.

MYC was the major hub gene found in our X2K analysis, and indeed, it was significantly upregulated both in COAD and READ tumors. It also exhibited moderate-to-high immunohistochemical staining in CRC tissues; however, its elevated levels did not associate significantly with the patients’ disease-free survival (Fig. 5c).

Transcription factor 3 (TCF3) is another hub gene whose elevated mRNA and protein levels we validated in the COAD and READ data sets, and high TCF3-expressing patients also have better disease-free survival (Fig. 5d).

Similarly, we validated the upregulated levels of various hub kinases, i.e., casein kinase 2α1 (CSNK2A1), cyclin-dependent kinases 1 and 4 (CDK1/4), and glycogen synthase kinase 3 beta (GSK3B) (Fig. 5e–h).

### Identification of repurposing drugs against CRC

**Fig. 6:**
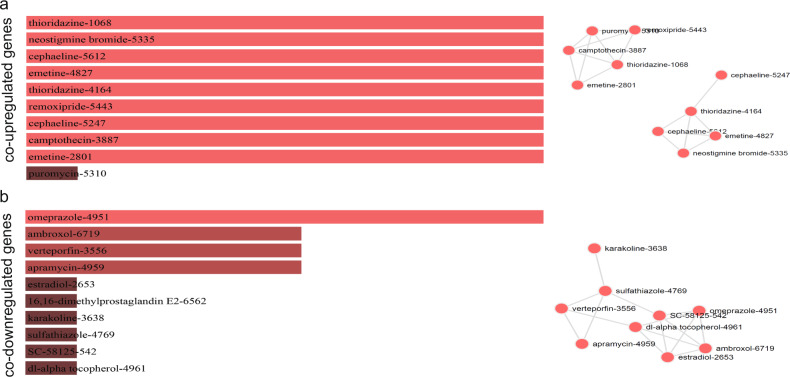
Repurposing drugs targeting the co-upregulated or co-downregulated genes in colorectal cancer, and the networks that they form. Τhe bar graphs are sorted by the combined score. The length of each bar represents the significance of its corresponding term. The brighter the color, the more significant that term is. The drugs in the network are sized according to their degree (number of edges), whereas the thickness of a connecting edge is proportional to the partial correlation coefficient between the two drugs. The nodes are arranged so that the edges are of more or less equal length and there are as few edge crossings as possible. For clarity, only the top 10 drugs ranked by partial correlation coefficient are shown.

We explored drugs that could putatively be used therapeutically in CRC, by uploading the co-DEG signatures in CMap. Overall, we found six candidate drugs targeting the co-upregulated genes (camptothecin, neostigmine bromide, emetine, remoxipride, cephaeline, and thioridazine); and four candidate drugs targeting the co-downregulated genes (omeprazole, apramycin, ambroxol, and verteporfin) (Fig. 6 and Supplementary Table 6).

The genes being targeted by the first group of repurposing drugs are as follows: *ID1;ENC1;CD14;SOX9;FOS;S100A11;ETS2;CTGF* (camptothecin); *LGALS3BP;IFITM1;SERPINA1;IFI27;CEACAM6;IFI6;ISG15;TSPAN1* (neostigmine bromide); *IL32;ID1;TXNIP;ID3;SOX9;FOS;IER2;CTGF* (emetine); *C3;IFITM1;IFI27;PTPRO;IFI6;SECTM1;ISG15;ITM2C* (remoxipride); *C3;ID1;TXNIP;ID3;SOX9;FOS;IER2;CTGF* (cephaeline); IL32;ID1;TXNIP;ID3;FOS;IER2;CTGF;PSMB9 (cephaeline); *C3;ID1;TXNIP;ID3;FOS;PHLDA1;IER2;PSMB9* (emetine); *JUND;S100P;SOX9;FOS;TGFBI;PHLDA1;IER2;ETS2* (thioridazine); *IFI27;CEACAM5;IFI6;ISG15;SOX9;FOS;LYZ;PHLDA1* (thioridazine). On the other hand, the most significant overlap between the co-downregulated genes was noted for omeprazole, which targets *IFITM2;IFI27;IFI6;S100A4;ISG15;PLP2;TSPAN1*.

On the other hand, the co-downregulated genes in CRC were found to be induced by diltiazem, and suppressed by baclofen.

In addition, pioglitazone, troglitazone, rosiglitazone, and spiradoline were predicted to increase the expression of the top hub TFs *SOX2, KLF4, NELFE, MYC*. The first three drugs are PPAR receptor agonizts, acting as insulin sensitizers. Pioglitazone is a launched drug, targeting *PPARG*, and *TRPM3*. Troglitazone and Rosiglitazone are both withdrawn. The first targets *ACSL4, ESRRA, ESRRG, PPARG, SERPINE1, SLC29A1*, and *TRPM3*; whereas the second targets *ACSL4, FFAR1, PPARG, TRPC5, TRPM3*. Spiradoline is phase 2 opioid receptor agonist, targeting *OPRK1*.

On the contrary, fulvestrant, tanespimycin, and monorden were predicted to reduce the expression of the above-mentioned hub TFs. Fulvestrant is a launched ER antagonist, known to target *ESR1, ESR2*, and *GPER1*. Tanespimycin is a phase 3 inhibitor of the heat-shock protein HSP90AA1, a chaperone responsible for protein maturation and stability^45^. Monorden (clenbuterol) is a substituted phenylaminoethanol that has β2 adrenomimetic properties at very low doses, and is used as a bronchodilator in asthma^46^.

Likewise, the drugs betaxolol, homatropine, clomifene, penbutolol, bisoprolol, atropine oxide, alpha-estradiol, propofol, prestwick, and arcaine were predicted to induce the expression of the top kinases involved in CRC (*CSNK2A1, CDK1, MAPK14, CDK4, GSK3B, AKT1*, and *CDK2*). In contrary, the drugs celastrol, irinotecan, clopamide, acetylsalicylic acid, metyrapone, betahistine, valproic acid, doxazosin, clotrimazole, and ajmaline can potentially reduce their expression.

### Co-DEGs within CRC experiments with a single-gene perturbation

**Fig. 7:**
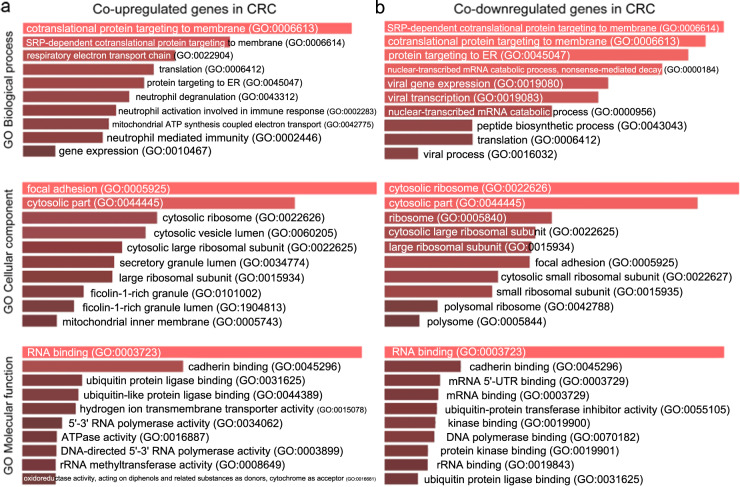
Gene Ontology (GO) enrichment analysis of the co-DEGs in single-gene perturbation experiments in colorectal cancer. **a** GO enrichment results of the co-upregulated genes in single-gene perturbation experiments in colorectal cancer. **b** GO enrichment results of the co-downregulated genes in single-gene perturbation experiments in colorectal cancer. Τhe bar graphs are sorted by the combined score. The length of each bar represents the significance of its corresponding term. The brighter the color, the more significant that term is.

The co-upregulated genes in single-gene perturbation experiments were highly enriched in the “cotranslational protein targeting to membrane” (Biological process); “methylosome”, and “cytosolic part” (cellular component); and “rRNA methyltransferase activity”, “oxidoreductase activity, acting on diphenols and related substances as donors, cytochromes as acceptor”, and “ubiquinol-cytochrome-c reductase activity” (molecular function), among others (Fig. 7a and Supplementary Table 7). Similarly, the co-DEGs in this category overlapped with “Parkinson disease”, “proteasome”, “Huntington disease” and “oxidative phosphorylation”, among others (Supplementary Table 8).

On the other hand, the co-downregulated genes in single-gene perturbation experiments were highly enriched in “SRP-dependent cotranslational protein targeting to membrane” (biological process); “cytosolic ribosome” (Cellular component); “ubiquitin-protein transferase inhibitor activity”; and “RNA binding” (molecular function) (Fig. 7b and Supplementary Table 7), and overlapped with the “ribosome” pathway in the KEGG.

**Fig. 8:**
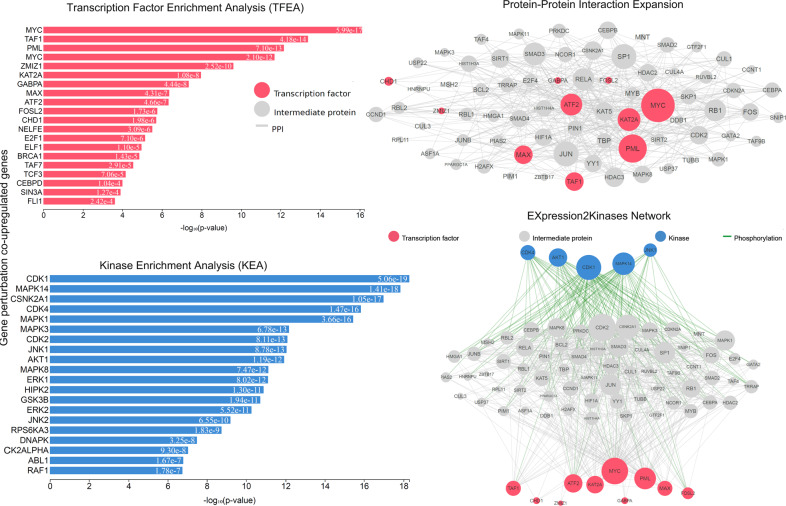
Upstream regulatory networks predicted to regulate the expression of the co-upregulated gene signatures in single-gene perturbation experiments inCRC, as inferred from the Expression2Kinases (X2K) analysis. Explanation of the inferred networks is provided in Fig. 3. MYC, PML, KAT2A, TAF1, ATF2 and MAX have higher k-core values and are hubs. MYC is more centralized in the network and has a stronger capacity of modulating adjacent genes.

**Fig. 9:**
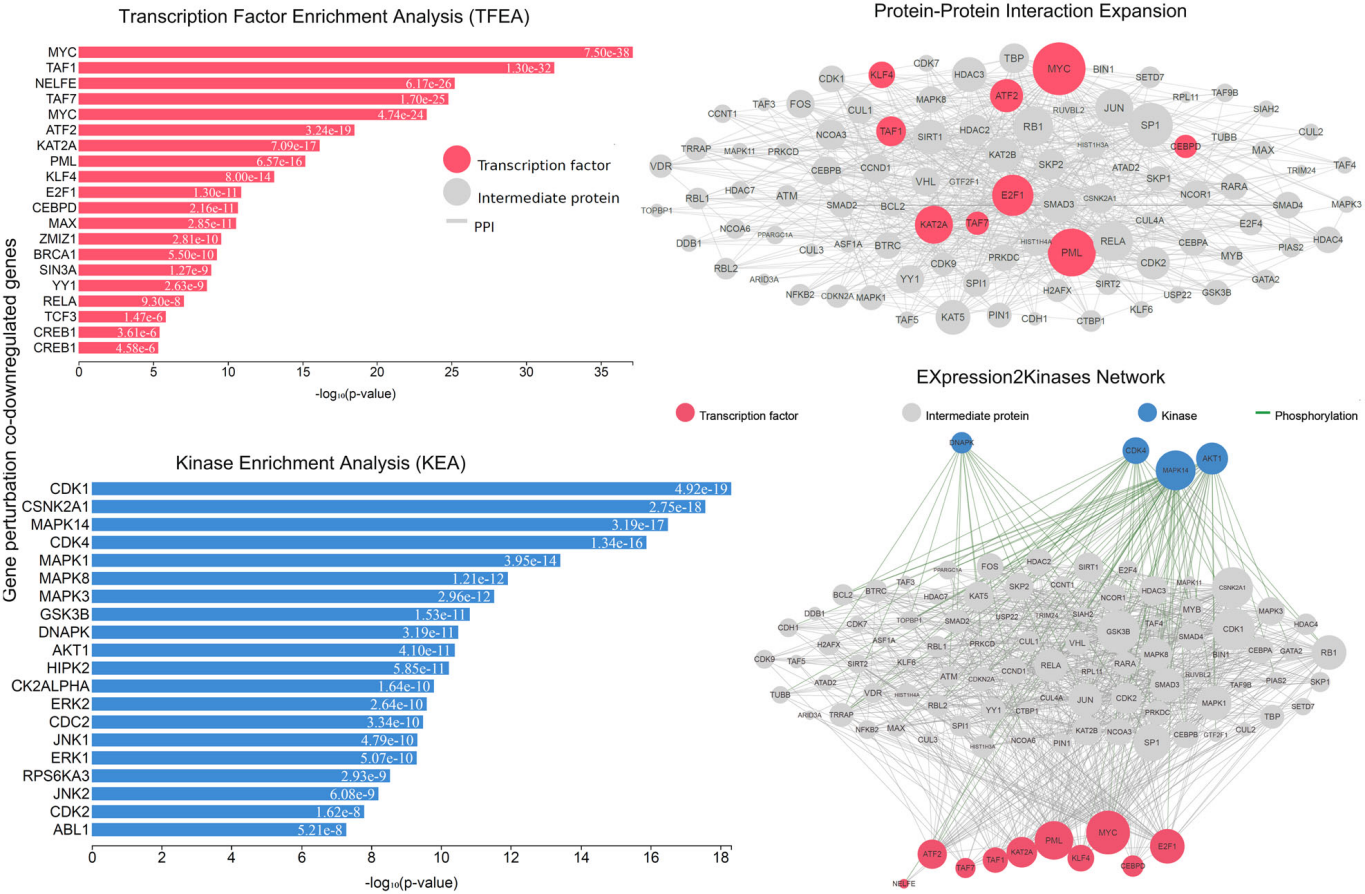
Upstream regulatory networks predicted to regulate the expression of the co-downregulated gene signatures in single-gene perturbation experiments in CRC, as inferred from the Expression2Kinases (X2K) analysis. Explanation of the inferred networks is provided in Fig. 3. MYC, PML, E2F1, TAF1/7, and KAT2A have higher k-core values and are hubs. These, are more centralized in the network and have a stronger capacity of modulating adjacent genes.

The TFs *MYC*, *KAT2A*, and *PML* were among the most significant hubs across the co-DEGs after single-gene perturbation (Figs. 8, 9). The kinases *CDK1, MAPK14, CDK4, JNK1*, and *AKT1* were among the hub genes responsible for the co-upregulated genes; whereas, *MAPK14, AKT, CDK4, and DNAPK* were hubs among the co-downregulated genes in this category (Fig. 9 and Supplementary Table 8).

Using the CMap database, we evaluated the co-upregulated genes in single-gene perturbation experiments in CRC, and found that disulfiram, and ciclopirox induce the expression of *JUN;MT1X;NDRG1;FOSL1;DNAJB1;MT2A;HSPH1;MAFF;SERPINH1;MT1H;EPHA2;MT1E;HSPA1A* (disulfiram) and *JUN;MT1X;FOS;EIF1;RHOB;FOSL1;DNAJB1;ZFP36;MT2A;MAFF;MT1H;PHLDA2;MT1E (*ciclopirox). We also found that laudanosine reduces the expression of *CCT3;VAMP8;POP5;UBL5;IRAK1;GNG5;RPL34;CFL1;PSME1;CHMP2A;ERH;SOD1*, and monastrol reduces the expression of *CHCHD2;SDC4;NDUFB11;UBE2S;RPL34;ADRM1;CIB1;PHLDA2;CCT7;NOP10;RHOB* (Supplementary Table 9). On the other hand, the co-downregulated genes in single-gene perturbation experiments were found to be induced by sulfaphenazole, and suppressed by midecamycin.

### Co-DEGs within CRC experiments with a single-drug perturbation

**Fig. 10:**
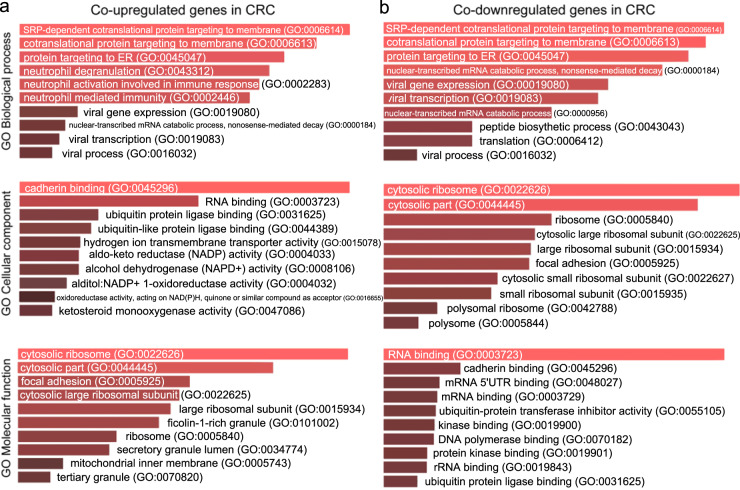
Gene Ontology (GO) enrichment analysis of the co-DEGs in single-drug perturbation experiments in colorectal cancer. **a** GO enrichment results of the co-upregulated genes in single-drug perturbation experiments in colorectal cancer. **b** GO enrichment results of the co-downregulated genes in single-drug perturbation experiments in colorectal cancer. Τhe bar graphs are sorted by the combined score. The length of each bar represents the significance of its corresponding term. The brighter the color, the more significant that term is.

Likewise, the co-upregulated genes in CRC experiments with a single-drug perturbation, were highly enriched in “SRP-dependent cotranslational protein targeting to membrane” (biological process), “cytosolic ribosome” (cellular component), and “ketosteroid monooxygenase activity” (molecular function) (Fig. 10 and Supplementary Table 10). The co-downregulated genes in this category were also highly enriched in “antigen processing and presentation of exogenous peptide antigen via MHC class I, TAP-independent”, and “cotranslational protein targeting to membrane” (biological process), “cytosolic ribosome” (cellular component), and “MHC class II protein complex binding” and “RNA binding” (molecular function) (Fig. 10 and Supplementary Table 10).

**Fig. 11:**
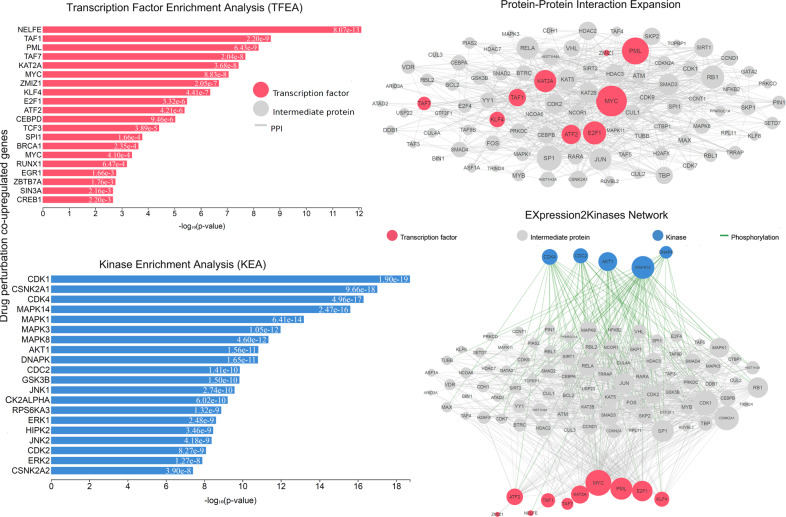
Upstream regulatory networks predicted to regulate the expression of the co-upregulated gene signatures in single-drug perturbation experiments in CRC, as inferred from the Expression2Kinases (X2K) analysis. Explanation of the inferred networks is provided in Fig. 3. MYC, PML, E2F1M, ATF2, TAF1, and KAT2A have higher k-core values and are hubs. These, are more centralized in the network and have a stronger capacity of modulating adjacent genes.

In this category, the TFs *MYC, PML, NELFE, TAF1, TAF7*, and *KAT2A*, as well as the kinases *CDK1, CSNK2A1, CDK4, MAPK14, MAPK1, MAPK3, MAPK8, AKT1*, were among the most enriched genes and MYC, KAT2A, and PML were the main hubs, driving gene upregulation after a single-drug perturbation (Fig. 11).

**Fig. 12:**
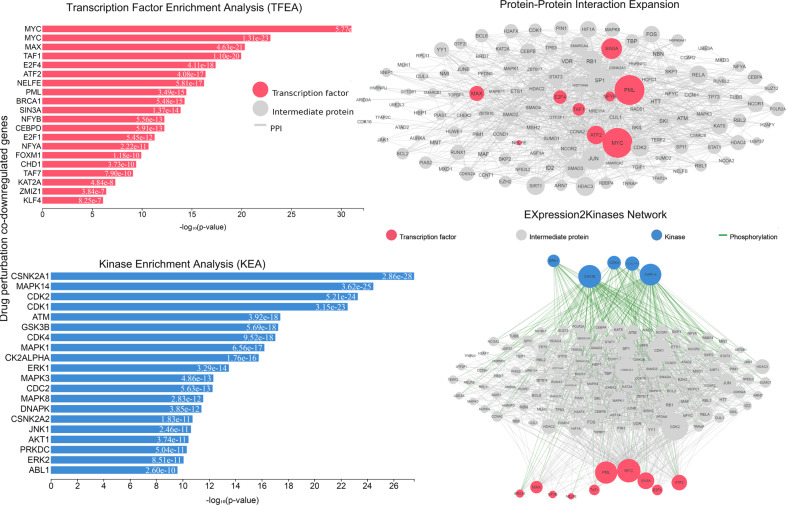
Upstream regulatory networks predicted to regulate the expression of the co-downregulated gene signatures in single-drug perturbation experiments in CRC, as inferred from the Expression2Kinases (X2K) analysis. Explanation of the inferred networks is provided in Fig. 3. MYC, ATF2, E2F4, TAF1, NFYB, SIN3A, and PML have higher k-core values and are hubs. These, are more centralized in the network and have a stronger capacity of modulating adjacent genes.

On the other hand, *MYC, MAX, TAF1, E2F4*, and *ATF2*, along with *CSNK2A1, MAPK14, CDK1/2, ATM, GSK3B, CDK4* were the most enriched TFs and kinase in the co-downregulated genes. Here also, MYC, PML, MAX, and NFYB were the major hubs in the PPI network (Fig. 12).

Using the CMap database, we evaluated these hub proteins and found that prochlorperazine, trichostatin A, and 15-delta prostaglandin induce the expression of the co-upregulated genes; whereas AG-012559-6920 inhibits their expression. In addition, we found that terfenadine induces the downregulation of the co-downregulated genes in this category, whereas resveratrol was found to suppress this downregulation.

## Discussion

Although significant steps have been made toward the understanding of the complex molecular mechanisms driving the pathogenesis of CRC, they are not fully understood. As a result, there is still a large number of genetic effects, various perturbations, and aberrations responsible for the onset and progression of the disease^47,48^. The understanding of the pathways and the deep exploration of the genes being involved in CRC, requires the interpretation of molecular signatures. Here, we followed a systems biology approach to investigate in-depth the co-DEG signatures, their upstream regulators, networks, and their hub proteins, along with protein–protein interactions (PPIs) in CRC. We observed the commonly deregulated genes, and the upstream regulatory kinases and transcription factors, which lead to the emergence of different patterns of gene expression, either upregulation or downregulation, in three categories: in CRC vs the normal tissue mucosa; and in CRC cells and tissue with a single-gene or a single-drug perturbation.

It is worth mentioning that gene expression represents literally a “snap-shot” of the state-space of the otherwise dynamic behavior of the disease in each particular biopsy. Nevertheless, this “snap-shot” is adequate to obtain useful insight on the dynamics of CRC cells.

Analysis of the co-DEG signatures in CRC using the CD approach allowed us to discover 164 co-upregulated and 199 co-downregulated genes. We identified the enriched pathways in which these co-upregulated DEGs participate, including TGF-β-signaling alterations, which are known to be implicated in the pathogenesis of CRC^49,50^. Contrary to our results, in a similar study, Guo et al.^51^found enrichment of the co-upregulated genes in Gα(i) signaling and GPCR ligand binding. In our study, the co-downregulated genes were mainly enriched in cotranslational protein targeting to the membrane, protein targeting to the ER, and nuclear-transcribed mRNA catabolic process, nonsense-mediated decay. On the other hand, Guo et al.^51^ showed that the co-downregulated genes are mainly enriched in cell cycle, mitotic prometaphase, resolution of sister chromatid cohesion, and aldosterone-regulated sodium reabsorption. This discrepancy can be owing to many reasons, including the different GEO data sets analyzed, different sample number and, of course, the different methodological approaches and testing for statistical significance. Nevertheless, both studies identified a critical role for the activation of inflammatory signaling pathways^52^, underlying the immune responses taking place in the cancer tissue. This is a main characteristic of hypermutated, MSI-H CRC tumors having a strong immune activation (consensus molecular subtype 1)^6^. Because of the expression of a defined set of tumor‐specific antigens, MSI‐H CRCs elicit a strong local and systemic anti-tumoral immune response of the host and therefore use different strategies to evade the control of the immune system^53^. Indeed, we and others^54–56^ have recently observed an increased CD8+ lymphocyte infiltration and expression of cytotoxic T-cell markers and effector cytokines, mainly in immunogenic colorectal tumors with a deficient mismatch repair system or high microsatellite instability (dMMR/MSI-H).

Guo et al. highlighted the activation of the WNT/β-catenin pathway^57,58^, which can lead to the disruption of intestinal epithelial homeostasis, increased cellular proliferation, decreased differentiation, and apoptosis in the intestinal tract^59,60^. Inflammation and DNA damage activate both the WNT/β-catenin and TGF-β1 pathways, which can interact between them^61^. Upon binding of WNT to its receptors (FZL and LRP5/6), the destruction complex AXIN/APC/GSK-3β is inactivated and the phosphorylation of β-catenin is halted, thus, saving it from proteasomal degradation. Then, β-catenin accumulates in the cytosol and translocates to the nucleus where it binds the co-transcription factor TCF/LEF and together they induce WNT target genes, including MYC and cyclin D1. TGF-β1 binds TGF-β receptor type 2, which recruits TGF-β receptor type 1, and one the hetero-tetramer is formed, it phosphorylates Smad2/3. This in turn, binds Smad4 and together they translocate to the nucleus to activate their gene targets.

The construction of a PPIs network provides insights for the major mechanisms governing the pathobiology of cancers^62^. Here, we reconstructed the PPI networks to elucidate the interactions among the co-DEGs in CRC, and found quite a few hub proteins, belonging either to TFs or to kinases, and function as signaling mediators in CRC. We defined MYC, TCF3, PML, KAT2A, and MAX as the main hubs, which are tightly related with CRC.

Among the co-upregulated TFs in CRC, we highlight the MYC proto-oncogene (MYC), transcription factor 2 (TCF3), promyelocytic leukemia (PML), and to a less degree the SRY-box transcription factor 2 (SOX2).

MYC has a critical function in CRC, orchestrating its promotion through multiple cellular pathways and is, therefore, a candidate drug target for its treatment^63–65^. It has multiple roles in the human genome, including the regulation of gene expression, and histone acetylation, and many mutant forms of MYC have been found in colorectal^66,67^ and other types of cancer^68,69^. Its upregulation in CRC was initially described >30 years ago^70,71^. However, as its upregulation is not correlated with the histologic type, stage or grade, MYC was not suggested to be a suitable prognostic marker for the disease^72,73^. MYC activates many downstream genes, leading to the promotion of cell cycle with DNA synthesis and an increase in chromosomal aberrations. These mechanisms eventually create genomic instability and chemo-resistance in CRC^74^. MYC deletion suppresses intestinal tumorigenesis in murine models, signifying that it is essential for colorectal tumorigenesis^75^. Interestingly, a recent study revealed that the metabolic reprogramming of CRC is primarily caused by aberrant expression of MYC^76^. Owing to its large PPI interfaces, the lack of deep protein pockets^77^, or low specificity of targeting its protein stability, MYC has so far been deemed undruggable. As MYC is usually overexpressed in late-stage cancers, its targeting for degradation is an attractive strategy^78^. So far, the bromodomain and extraterminal (BET) inhibitors have been designed to target MYC; however, they exhibit a pleiotropic effect and sometimes, their activity is independent of an effect on MYC, which adds further complication^79^. Other efforts focus on interrupting the dimerization of MYC with MAX, inhibiting MYC/MAX binding on the DNA, interfering with key c-MYC targets, and inhibiting c-MYC in cancer stem cells^74^.

The deregulated WNT/β-catenin signaling promotes carcinogenesis in the colon by activating MYC expression. In the nucleus, the β-catenin transcriptional co-activator binds T-cell factor (TCF) transcription factors, and together TCF/β-catenin complexes activate the expression of MYC, cyclin D1, c‐jun, fra-1, peroxisome proliferator-activated receptor δ, matrilysin, CD44, and urokinase-type plasminogen activator receptor, which contain Tcf/lymphoid enhancer factor (Lef)-binding sites in their promoters^80–82^. In addition, TCF3 along with the lncRNA ASBEL, are directly transactivated by β-catenin and form a complex that downregulates the expression of activating transcription factor 3^83^. The Tcf/Lef circuit model was also proposed to provide a mechanism downstream of β-catenin stability for regulating the potency of the activity of WNT signaling during embryonic development^84^. TCF3 represses MYC by inhibiting the formation of TCF4/β-catenin complexes^80^. Therefore, drugs or small molecules that promote the function of TCF3 could be applied to treat effectively CRC patients.

Initially viewed as a tumor suppressor, the PML protein lately re-emerged as a multifaceted molecule that controls many different aspects of cellular homeostasis. The PML gene fuses with the retinoic acid receptor‐α (RAR-α) gene, resulting in the PML protein in acute promyelocytic leukemia and disrupting the PML nuclear bodies. PML nuclear bodies accumulate several proteins involved in multiple cellular pathways such as apoptosis, differentiation, transcriptional regulation, maintenance of genomic stability, or proteasomal degradation of ubiquitinated proteins, and is disorganized in CRC^85^. In addition, cytoplasmic PML can physically interact with Smad2/3 and SARA (Smad anchor for receptor activation), modulating TGF-β signaling^86^. PML downregulation has been previously reported in CRC, where its loss of expression associates with an aggressive tumor behavior^87^. PML protein expression was also reported to correlate with the outcome of metastatic CRC patients who are treated with oxaliplatin/fluoropyrimidine‐based first line therapy^88^. In addition, Yamada et al.^89^ proposed a signaling pathway of miR-1246/PML/Smad 1/5/8 through which CRC cells secrete microvesicles, which contribute to the tumor’s angiogenesis. Interferons, arsenic, or other oxidants can induce the formation of nuclear bodies^90^.

SOX2 is involved in the bone morphogenetic proteins signaling cascade, steroid metabolic process, histone modifications, and other receptor-mediated signaling pathways^91^. SOX2 is overexpressed in CRC^91^ and its expression associates with a cancer stem cell state and downregulation of the intestinal epithelial marker CDX2^92^. In addition, its expression was shown to be controlled by BRAF and to contribute to poor patient prognosis^93^. SOX2 expression was further reported to correlate with lymph-node metastases and distant spread in right-sided colon cancer^94^.

Among the co-downregulated TFs in CRC, we highlight the hubs MYC, PML, KAT2A, and MAX. MAX (Myc-associated factor X) forms a transcriptional complex with MYC and binds to the DNA to activate the expression of multiple genes with the recruitment of other transcriptional coactivators, including p300, lysine acetyltransferase 2 A (KAT2A), and KAT5^95,96^.

Overall, we followed a systems biology approach, through which we analyzed the co-DEGs in CRC, as well as in CRC experiments with single-gene or single-drug perturbations. We detected the major hubs, including TFs and kinases, and we analyzed the molecular networks formed by these co-DEGs in each group.

In addition, we identified the candidate repurposing drugs targeting the co-DEGs and the major hub genes. We highlight camptothecin, neostigmine bromide, emetine, remoxipride, cephaeline, and thioridazine, among others.

Camptohecin (CPT) is a well-known anticancer drug with different derivatives aiming to increase its low solubility. Two such analogs are topotecan and irinotecan, both FDA-approved and currently being used as chemotherapeutic agents^97^. Camptothecin sensitizes dMMR CRC cells^98^. CPT-11 also showed promising antitumor activity against metastatic CRC that was resistant to prior therapy^99^. In addition, E2F-1 and MYC overexpression were found both to sensitize CRC cells to camptothecin^100,101^. CPT binds to the topoisomerase I and DNA, preventing DNA re-ligation and causing DNA damage, which leads to apoptosis^102,103^. CPT is selectively cytotoxic to the cells replicating DNA during the S phase^104^ and its toxicity is mainly owing to the conversion of single-strand breaks into double-strand breaks, when the replication fork collides with the cleavage complexes formed by DNA and CPT^105^.

Neostigmine bromide is the bromide salt of neostigmine. It is a cholinesterase inhibitor used in the treatment of myasthenia gravis^106^ and to reverse the effects of muscle relaxants, such as gallamine and tubocurarine^107^. Its effect has not been tested in the context of human CRC, so far. Nevertheless, some very interesting findings were reported by Tatsuta et al.^108^ a long time ago, who examined its effect on rats prior to being injected with azydomethane, a CRC-inducing carcinogen. The authors reported a significant reduction in the number of adenocarcinomas produced after treatment of rats with neostigmine, compared to the control (olive oil, vehicle). The administration of neostigmine decreased significantly the labeling indices of colonic mucosa during carcinogen treatment, but increased it after that^108^. Although this study suggested that neostigmine has an inhibitory effect in the development of colonic tumors, to our knowledge, it has not been investigated any further. In another study, neostigmine could rapidly decompress the colon of patients with acute colonic pseudo-obstruction who did not respond to conservative therapy^109^.

Emetine is an alkaloid drug produced from the *Ipecacuanaha* root species and its use so far, is to act as an anti-amebic (dehydroemetine) and to induce vomiting. Although emetine showed anticancer potential 40 years ago, it was withdrawn from the development as an anticancer agent owing to dose-dependent muscle weakness and cardiotoxicity in clinical trials^110^. Interestingly, emetine dihydrochloro hydrate binds to the 40 S ribosomal subunit in eukaryotic cells, blocking protein synthesis^111^. Emetine can promote TRAIL-induced apoptosis of pancreatic cancer cells^112^. Just recently, emetine alone demonstrated high anticancer activity against CRC cell lines, although when combined with oxaliplatin (alkylating agent) it exhibited mild synergism at higher concentrations of administration^113^. Cephaeline is a desmethyl analog of emetine. We can thus hypothesize that emetine and cephaeline could be tested against CRC cells.

Remoxipride is an atypical antipsychotic drug, but has been withdrawn owing to toxicity concerns. It acts as a selective antagonist of the dopamine 2 and 3 receptors (D2R and D3R) and has a high affinity for the sigma receptor, possibly playing a role in its atypical neuroleptic action^114^. Remoxipride was tested in the MCF-7 breast cancer cells, but was not capable to suppress their proliferation, contrary to bromocriptine (an ergoline derivative and dopamine agonist). The only effect remoxipride had, was to suppress the effect of bromocriptine^115^. Further evidence on the effect of remoxipride in cancer was just recently provided in the context of the U87 glioblastoma cells; where this drug was shown to decrease their sphere-forming frequency^116^. Nevertheless, to our knowledge, this drug has not been tested in the CRC context.

Thioridazine is another D2R antagonist, similar to remoxipride. It is a member of the phenothiazine family and a potent anti-anxiety and antipsychotic drug, which has been shown to elicit potent antitumor effects in CRC stem cells^117,118^. Therefore, there is supporting evidence that thioridazine could be used as a promising agent against the disease. We hypothesize that both D2R antagonists, remoxipride, and thioridazine, could similarly decrease spheroid formation of CRC cells, as an effect on the dopamine D2R (or other) signaling.

Regarding the putative drugs that could increase the expression of the co-downregulated genes, here we highlight omeprazole, a proton pump inhibitor that induces apoptosis^119^. Its protective role against colorectal carcinogenesis was also shown in a rat model^120^. Omeprazole can also synergistically improve the efficacy of concurrent chemoradiotherapy in rectal cancer^121^. In addition, this drug has significant dose–response efficacy effects on the progression of adenomas to adenocarcinomas, especially when combined with aspirin^122^.

Apramycin is an aminoglycoside antibiotic used to treat Gram(−) bacterial infections in animals. It functions via binding to the eukaryotic ribosome and thus, inhibiting protein synthesis A synthetic aminoglycoside derivative (NB124) was recently developed and shown to suppress premature termination codons in TP53 and APC in human cancer cells, and therefore, induce their apoptosis^123^. Although apramycin could not stabilize the mutant TP53 mRNA and restore its full-length protein production in cancer cells^123^, there seems to be a new challenging role for a new generation of aminoglycosides in CRC.

Ambroxol is a mucolytic drug used to treat respiratory diseases. A recent hypothesis suggested that it could be used against Paget’s disease of bone, Parkinsonism, and other common diseases of aging-associated diseases, involving dysfunction of autophagy^124^. Ambroxol is a potent inhibitor of the neuronal Na+ channels, but also an anti-inflammatory drug^125^. We can thus hypothesize that it might have a beneficiary action in the inflamed intestinal epithelium in CRC.

Verteporfin is a benzoporphyrin derivative used as a photosensitizer for photodynamic therapy to eliminate the abnormal blood vessels in the eye, associated with macular degeneration. Its mode of action is via its accumulation in the abnormal blood vessels and, upon stimulation, it produces highly reactive short-lived singlet oxygen and other reactive oxygen radicals, which result in local damage to the endothelium and blockage of the vessels^126^. Recently, verteporfin was shown to inhibit the growth and invasion of YAP-induced bladder cancer cells via repressing the target genes’ expression of the Hippo signaling pathway. As this pathway is also related to the progression of CRC and its resistance to EGFR inhibitors^127^, based on our data we hypothesize that verteporfin could have a beneficial effect in CRC treatment, as well.

The above-mentioned findings support the validity of our experimental approach. However, the identified co-DEG signatures and putative repurposing drugs that we propose here deserve further experimentation, as they show a great potential to be used as candidate biomarkers and therapeutic agents in CRC. Nevertheless, prior to performing a systematic evaluation of these repurposing drugs, we need to keep in mind some practical implications these might have, owing to their vastly different targets, mechanisms of action, and applications. For example, there might be restrictions on their handling and distribution or they might have low stability to survive long-term storage and handling.

Overall, we identified the critical genes involved in CRC and propose repurposing drugs that could be used against the disease. The top co-DEGs and their hubs within each group of study, are confirmed by previous studies, highlighting the accuracy of our methodology and our suggestion that they could be targeted with new therapeutic drugs.

## Methods

### Extraction and filtering of gene expression signatures from GEO

We focused on 19 independent studies and categorized them into three groups, i.e., those (1) comparing gene expression between CRC and normal tissues (five GEO studies); (2) containing a single-gene perturbation (e.g., knock-in, knockout, mutation, etc.) (six GEO studies); and (3) studies reporting experiments with a single-drug perturbation in CRC (eight GEO studies). We extracted gene expression signatures directly from GEO, using GEO2Enrichr^20,128^. After first finding the relevant GEO studies falling under each of these three categories, the perturbation and control samples (GSMs) were selected from GEO series (GSE) or GEO data sets (GDS). Only gene expression studies from human and mouse tissue samples or cell lines were considered valid. Standard names of genes, diseases, and drugs were provided as autocomplete options in the submission forms, created from controlled vocabularies: HGNC for genes^129^, disease names from the Disease Ontology^130^ and drug names from DrugBank^131^. In detail, the corresponding GEO data sets selected for each category were as follows: GDS389, containing 10 APC^Min/+^ mutant samples (five adenomas and five carcinomas) and six C57/BL6 wild type (controls); GDS2609, containing 12 early onset CRC samples and 10 healthy controls; GDS2947, containing 32 adenomas and 32 normal mucosa samples; GDS4382, containing 17 CRC tumor and 17 normal tissues; GDS4385, containing three carcinoma-associated fibroblasts, three CD133+ CRC cells, and three CD133- CRC cells (CRC vs normal signatures); GDS170, containing four p53^−/−^, four p53^−/+^, and four p53 ^+/+^ HCT116 colorectal carcinoma-derived cell lines; GDS2141, containing two PKCa wild type and two PKCa knockout intestines; GDS4101, six HT29, colon-derived, and two Colo357, pancreas-derived cancer cell lines; GDS4384, containing five wild type and five p53 mutant microsatellite-stable stage III colorectal adenocarcinomas; GDS5268, containing 74 HCT116 cells treated at 3xIC90, four untreated HCT116 cells, 20 HCT116 cells treated with vehicle (DMSO); GDS3482, containing eight x-linked inhibitor of apoptosis-depleted samples, eight control samples, and two parental controls (single-gene perturbation signatures); GDS3032, containing four Caco-2 colon cancer cells treated with quercetin, and four controls; GDS3160, containing three HT29-derived colon cancer cells sensitive and two resistant to the anticancer drug methotrexate (MTX); GDS3330, containing three HT29-derived colon cancer cells sensitive and three resistant to MTX; GDS4383, containing two saracatinib-sensitive PIK3CA mutant and three saracatinib-resistant PIK3CA mutant patient-derived CRC xenografts; GDS4386, containing six β-catenin shRNA, and six dominant-negative Tcf4 transgenes; GDS4393, containing 12 metastatic CRCs, FOLFOX non-responders, nine metastatic CRCs, FOLFOX responders, 15 primary CRCs, FOLFOX non-responders, and 18 primary CRCs, FOLFOX responders; GDS1413, containing 18 RKO colorectal carcinoma cell lines exposed to subcytotoxic and cytotoxic amounts of 4-hydroxy-2-nonenal (HNE), and six controls; GDS4397, containing five CRC cell lines treated with 5-aza-2’-deoxycytidine (5-aza-dC) and five controls (single-drug perturbation signatures).

We applied four quality control filters to get a better assembly of the extracted signatures, as previously explained^20^. Initial integrity checks were performed, using the association between GEO studies (GSE or GDS) and samples within these studies (GSMs) by re-processing all the collected gene expression signatures based on the metadata. We excluded signatures in which GSMs did not match their GSE or GDS, as well as signatures with the same GSMs in the control and perturbation groups. Regarding the single-gene perturbation studies, we checked the validity of the HUGO Gene Nomenclature Committee (HGNC) gene symbols, excluding all the invalid ones. In addition, signatures in which the perturbation and control samples were swapped, were corrected. Last, we checked if the submitted gene signatures are in accordance with the descriptions associated with the original GEO studies. Batch effects were quantified using principal variation component analysis^132^ and corrected using surrogate variable analysis^133^.

### Differential gene expression and co-DEGs among different studies

Differential gene expression was calculated using the CD algorithm^22^. GEO2Enrichr was used to submit the gene sets of the overexpressed (termed “*up*”) and underexpressed (termed “*down*”) genes in CRC vs. the adjacent normal tissue. In the case of a single-gene perturbation, DEGs were calculated with respect to the normal/wild-type gene; and in the case of a single-drug treatment, in relation to cells or tissues that were not treated, accordingly^128^. A cutoff of 500 genes was applied for all DEG analyses using the CD method. For each category/group, the co-DEGs, i.e., the genes being either co-upregulated or co-downregulated between at least two independent studies, were calculated. DEGs appearing within a single study were filtered out.

### Upstream regulators of co-DEGs

Expression2Kinases (X2K)^134,135^ was used to infer upstream regulatory networks from signatures of DEGs in each category. We produced inferred networks of transcription factors, intermediate proteins, and kinases predicted to control the expression of the inputted gene list, by combining TF enrichment analysis, protein-protein interaction (PPI) network expansion, with kinase enrichment analysis.

In specific, the X2K workflow contains the identification of the upstream TFs of the co-DEGs within each category. These, are detected using promoter analysis of ChIP-X enrichment analysis, which was followed by connecting the identified TFs between them and constructing a protein interaction subnetwork (Genes2Networks, G2N)^136^, using known protein interactions. Finally, we identified the upstream protein kinases which are likely to regulate the formation of such subnetworks, using kinase enrichment analysis^134,135,137^.

### Protein–protein interactions

The PPI were identified via a network onto which the transcription factors, the protein kinases, and the intermediate proteins for each class of the co-DEGs were recorded, as well as the values of these proteins (nodes) and lines (edges) contained in each network, using Enrichr^138^. The edges indicate PPI and phosphorylation between a kinase and either an intermediate protein or a TF. The node size signifies the level of expressed degree. Betweenness for a node N was computed by selecting a pair of nodes and finding all the shortest paths between these nodes. The fraction of these shortest paths that include node N was then computed. k-Core decomposition was used for visualization of the network structure. The proteins with higher k-core values were more centralized in the network and thus, considered hubs. These, are more able to modulate adjacent genes.

### Gene Ontology enrichment and KEGG pathway analysis

Gene ontology (GO) enrichment and Kyoto Encyclopedia of Genes and Genomes (KEGG) pathway analysis was used to explore the biological functions of the top co-DEGs. Enrichr^138^ was employed to recognize the biological processes, cellular components and molecular functions of the top co-DEGs within each category. The hypergeometric test was used in order to find GO entries which were significantly enriched compared to the whole human genome. KEGG pathway analysis was also implemented using Enrichr^138^, to discover the biological pathways in which each category’s top co-DEGs participate. Statistical significance was evaluated using a combined score, computed by taking the log of the Benjamini-Hochberg-corrected *p* value from the Fisher exact test and multiplying it by the z-score of the deviation from the expected rank.

### Detection of repurposing drugs in CRC

The Connectivity Map (CMap, https://clue.io/cmap) was used to identify repurposing drugs that could potentially induce or reverse CRC, based on the extracted gene expression signatures^139,140^. Perturbations eliciting highly similar, or dissimilar, expression signatures were termed “*connected*”. A positive connectivity value (closer to +1) indicates that the drugs could induce malignant transformation of the cells in CRC, whereas a negative connectivity value (closer to −1) indicates that greater similarity among the genes and the drugs could reverse the status of the CRC cells. Drugs were statistically associated with the disease using the hypergeometric probability test.

### Verification of the co-DEG signatures

We verified the co-DEG signatures in CRC, using the colon (COAD) and rectum (READ) adenocarcinoma data sets from the Cancer Genome Atlas (TCGA). The read counts of RNA-seq data were extracted from a total of 275 tumors and 41 normal samples from the COAD data set, and 41 rectum tumors and 10 normal samples from the READ data set, using the GDC Data Portal (https://portal.gdc.cancer.gov/). The read counts were then normalized to transcripts per million mapped reads adding an offset of 1 (TPM + 1), as previously described in detail^54,141^.

In specific, the mRNA expression levels of the gene signature “UP genes”, composed of *FOS, MMP7, HIF1A, ANXA2, JUND, ALDOA, GGH, APP, CFI, EMP1, LY6E, TUBA1AC, CLU, CTSE, SYPL1, PABPC1, PROM1, FXYD3, FABP1, PHLDA1, PGM1, LDHA, NPM1, PGAM1, GAPDH, HSP90AB1, SLCO5A1, TFF2, S100A10* (co-upregulated in CRC) and the gene signature “DOWN genes”, composed of *RPS10, RPS26, EMP1, GNG5, KRT20, RPL20, PABPC1, RPL37A, KRT8* (co-downregulated in CRC), were assessed using *limma*^142^ with a threshold of log_2_FC = 1 and *q* value = 0.01 for statistical significance.

To confirm the reliability of individual hub genes and correlate their expression with the patients’ clinical characteristics and disease-free survival, the GEPIA2^43^ and HPA databases were used, as previously described^44^. The gene expression levels of each hub were also presented in log_2_(TPM + 1) values and compared between cancer and normal tissue within each TCGA data set (COAD or READ). A *p* value equal to 0.05 was set as threshold for statistical significance (ANOVA).

IHC images from tissue samples were derived from the HPA. The antibodies used in IHC were as follows: mouse mAb anti-MYC, CAB000084, 1:600 dilution, Agilent, Cat# M3570; Antigen retrieval was performed using HIER pH9; rabbit msAb anti-TCF3, CAB018351, 1:375 dilution, Santa Cruz Biotechnology, Cat# sc-349; Antigen retrieval was performed using HIER pH6; mouse mAb anti-CSNK2A1, CAB069395, 1:20000 dilution, AbFrontier, Cat# LF-MA0223; Antigen retrieval was performed using HIER pH6; rabbit mAb anti-CDK1, CAB003799, 1:50 dilution, AbCam, Cat# 1161-1; antigen retrieval was performed using HIER pH6; mouse mAb anti-CDK4, CAB013116, 1:45 dilution, ThermoFisher Scientific, Cat# AHZ0202; antigen retrieval was performed using HIER pH6; mouse mAb anti-GSK3B, CAB016263, 1:100 dilution, Santa Cruz Biotechnology, Cat# sc-7291; antigen retrieval was performed using HIER pH6.

Disease-free survival was analyzed using the Log-rank test and median values as cutoff, in GEPIA2^43^. The Cox proportional hazard ratio and the 95% confidence interval were included in the survival plots.

## Supplementary information

Supplementary Information

## Data Availability

The data that support the findings of this study are available in the Figshare repository (10.6084/m9.figshare.11865447).
