# Author Correction: Signatures of co-deregulated genes and their transcriptional regulators in colorectal cancer

**DOI:** 10.1038/s41540-020-00149-3

**Published:** 2020-09-14

**Authors:** Natalia Mastrogamvraki, Apostolos Zaravinos

**Affiliations:** 1 Department of Life Sciences, School of Sciences, European University Cyprus, 1516 Nicosia, Cyprus; 2 Department of Basic Medical Sciences, College of Medicine, Member of QU Health, Qatar University, Doha, Qatar

Correction to: *npj Systems Biology and Applications* 10.1038/s41540-020-00144-8, published online 31 July 2020.

In the original version of this Article, the wrong images were supplied for Figures 7 and 10. This has now been corrected in both the HTML and PDF versions of the Article.

