# Supplementary material for: Signatures of co-deregulated genes and their transcriptional regulators in colorectal cancer
Source: NPJ Syst Biol Appl. 2020 Jul 31;6:23. doi: 10.1038/s41540-020-00144-8 (PMC7395738; doi:10.1038/s41540-020-00144-8)
Supplement: Supplementary file 1 — Supplementary Information [file 41540_2020_144_MOESM1_ESM.pdf]

Supplementary Information

Supplementary Table 1: Co-deregulated genes (co-DEGs) in CRC against the adjacent normal mucosa; in CRC tissue or cell lines with a single-gene perturbation against the wild type (wt) tissue or cells; and in CRC tissue or cell lines with a single-drug perturbation against non-treated tissue or cells.

co-upregulated genes_CRC vs Normal	co-downregulated genes_CRC vs Normal	co-upregulated genes_single-gene perturbation	co-downregulated genes_single-gene perturbation	co-upregulated genes_single-drug perturbation	co-downregulated genes_single-drug perturbation
COX2	SLC26A2	ATP1B1	C1orf106	NDRG1	S100A11
CEACAM6	RPS10	COX1	SNHG5	GPX3	EPHB3
SLC12A2	MT2A	RPL7A	SHCBP1	LCN2	PKM
LCN2	GUCA2A	ALDH1A3	RPL23A	PLAC8	MT1E
OLFM4	EMP1	PSMB3	RPS8	HIGD2A	REG1A
COL1A2	CHP2	KRT18	CPS1	S100A11	MCM5
CCL20	PIGR	LAP3	RPL14	GPRC5A	HIST1H4C
TUBA1A	MUC13	CLDN7	SAT1	SPARCL1	SERPINA1
SYPL1	OAT	RPL27A	IFI27	LGALS4	LCN2
DSG2	LGALS4	CRIP1	UQCRB	SFN	RPL36
IFITM3	RPL3	MT1H	HSPD1	OAT	MT1HL1
HIF1A	RPL27	PLAU	HSP90B1	TRIB3	ZWINT
AGR3	CHGB	UBE2S	TUBA1A	KRT20	DDIT4
ND2	ADAMDEC1	NDUFS6	GLO1	FAM3B	ISG15
CFI	PFN1	PGAM1	TMSB10	RPL4	ANP32B
GGH	PCBP1	RPL38	RPL9	ATF4	HSP90AB1
MMP7	RPS19	SLC25A5	IGFBP3	QPCT	BUB1B
SLCO1B3	CLCA1	DNAJB1	CD24	PLAT	RPL8
APP	CES2	C20orf24	RPS3	ODC1	ATP6V0B
ANXA2	ITLN1	TMA7	PABPC1	NQO1	PTBP1
PTPRO	RPL35	S100A4	RPL41	HIST1H2BK	KLK6
TUBA1C	SPINK5	KRT8	HSP90AA1	XIST	C1QBP

CEACAM5	RPS12	ODC1	ID2	ITM2B	COX5A
PIGR	ALDH2	PFN1	IGJ	CEACAM6	NELFCD
IFI27	RGS2	ANXA3	IFITM1	S100P	RPS6
IGFBP2	CD177	TIMP1	KLF10	CCL20	ATP1B3
LUM	DHRS11	S100A10	HMGB1	COX7C	CXorf40B
IFITM1	VIM	FAU	RPLP0	RPS23	EIF3H
JUND	SST	PSME2	NACA	SDC4	RPS3A
PABPC1	TSPAN8	TFF3	YWHAZ	TXNIP	PAICS
TUBB	AKR1B10	MT1E	RPL4	USMG5	PABPC1
ACTB	ZG16	GCLM	NDRG1	ARL6IP5	DDX39A
FOS	ETHE1	PGRMC1	ID3	COX5B	SSR4
TSPAN1	RPL23	PKM	DYNLL1	ALDH2	GINS1
SLCO5A1	IGLV1-36	CHMP2A	HNRNPA1	NDUFA1	HLA-F
FOXQ1	MDH2	TOMM20	OAZ1	NDUFA4	CKS1B
CDH3	NCOA4	ATP5L	RPS24	FABP1	NUSAP1
LY6E	GNB2L1	PNP	PPA1	PTTG1	PTMA
PKM	AQP8	RPS7	RPL7	RPL36AL	ASNS
TUBA1B	LGALS1	LGALS3BP	RPS16	ECHS1	PHB2
ID1	UGT2B17	RPS3	RPS11	MKNK2	SLC25A3
CD24	ETFA	ND2	PCNA	TSPAN13	PSAT1
SOX9	CDH17	POLR2J	RPS27	C14orf2	RPS25
DUOX2	RPS2	SPINT2	CPOX	RPL24	NME1
HSPB1	C15orf48	LRRC47	RPL3	SPINK1	TBCA
MMP12	RPL37A	IRAK1	RPS7	MYL12B	EEF1B2
DYNLL1	IL1R2	ATP5H	RPS20	EEF2	UBB
FABP1	HADH	OAS1	COX7A2	NDUFS6	SLIRP
ERRFI1	RPL23A	PPIA	PTTG1IP	BZW2	ST6GAL1
CTGF	ALDH9A1	GNG5	ID1	PFDN5	PCNA
SPP1	ADH1C	UBL5	HSPA8	PGM1	IFITM2
CXCL1	RPL32	EIF3D	RPLP1	TUBA1A	RPS26
PROM1	HEPACAM2	P4HB	TMED10	TM9SF2	H3F3B
ANXA3	SUCLG1	EIF3D	GDI2	LASP1	KPNA2
RPS14P3	PDLIM1	DCXR	HSP90AB1	HIGD1A	LDHA
GPX2	NDUFAB1	GSTP1	RPS5	TFRC	C3
PTMA	COX7C	RPS17	SOX4	FAU	IL32
PDZK1IP1	MUC2	SMS	GADD45A	ATP1B1	KRT20
MMP3	LBR	GLRX	RPS23	COX6B1	SLC7A5

ISG15	TMA7	LSM7	ATP5C1	EDF1	MYC
CTSE	NDUFB9	NDUFS3	CD164	ATP5L	MAD2L1
LDHA	RPS13	PABPC4	CCNG1	UQCRH	HLA-C
CD63	GUCA2B	DRG1	DSTN	ATP5I	DYNLRB1
IFI6	ACADM	POLR2L	EIF4A2	PSMD11	MCM6
FXYD3	RPSA	UBB	EPS8	ATP6V0B	RASA1
TMEM123	EPCAM	HLA-A	PTMA	TXNRD1	RPA3
IFITM2	PLAC8	NDRG1	UBC	OCIAD2	GARS
TSPAN13	IGKV1-5	LAPTM4B	RPL13A	SSR2	YWHAE
S100A11	CYFIP1	NOP10	MDH1	TALDO1	LYZ
ENC1	RPL22	CAP1	PON2	LAPTM4A	MRPL3
TGFBI	PHB2	SRSF9	RPL23	PSAP	MORF4L1
IL32	C19orf33	PPIF	ALDOC	S100A4	PRSS2
S100A10	KLF4	NACA	HADHB	TSPAN8	TUBB
ALDOA	HADHB	AOC1	UBA2	AKR1C2	PRR13
TXNIP	KRT8	RPL26	NCL	TUBB2A	SIGMAR1
CXCL8	TALDO1	COX7B	H1F0	AKR1C3	S100A6
PHLDA1	TDP2	UQCRC1	RPL7A	CES1	TMEM97
TUBB4B	RPS26	RPL34	HMGN3	SNRPD2	CREG1
LAPTM4A	MALL	POP5	HIF1A	PCBP1	CDC20
NDUFA4	CLCA4	EMG1	IER3	TMSB10	COX6C
RNF128	TPT1	BCAP31	RPL31	FTL	LAMP1
TCN1	CTSS	S100A6	IRF2BPL	RPL7A	TRA2B
SPARC	PDCD4	LAMP1	SLC7A5	DAD1	TOMM20
DCN	RPL30	C12orf75	ATF4	ACTB	TNS4
NPM1	CPE	SLIRP	RPL17	EIF5	HLA-A
S100A4	RPL34	HINT1	ANXA4	RARS	ODC1
BMP4	CYSTM1	ATP6V0C	SLC38A2	RBCK1	EIF6
PGM1	FTH1	PSMB7	LGALS3	PHB2	CIRH1A
CKB	CLDN8	EIF1	PPIB	TMBIM6	CCNG1
S100P	CALML4	KRT20	CDKN1B	CAPNS1	IRAK1
ATP1B1	CFL1	CXCL8	PPP1CC	AKR1C1	PTGES3
NQO1	CLDN7	RPL36A	RPL8	NHP2	CCNA2
COL1A1	SRI	CD63	SLC25A6	ETFB	EIF3E
NOP10	PKIB	CAPN2	EIF3H	GABARAP	RPLP1
PGAM1	F2RL1	TALDO1	RPS3A	LGALS3	DKC1
MIR21	CHGA	MISP	RPS29	KRT8	FABP5

PSMB9	ACAA2	ACTG1	SLC25A3	AP2S1	CCNB1
TFF2	SLC25A5	EPHA2	FAT1	ALDH3A1	RPS5
PSME2	SGK1	POLR2F	KPNB1	POLR2I	FAM60A
SPINK1	KRT20	S100P	UQCRC2	HAPLN1	HNRNPUL1
TSPAN8	RPS20	NME1	ATP5A1	RPS2	RPL9
APCDD1	RPS5	SFN	RPL30	ARPC1A	NDUFAB1
RPL28	IGKV1OR2	ALDOA	LBR	C1orf106	ARPC1B
GAPDH	ATP5A1	HLA-B	EEF1A1	ALAS1	KARS
IER2	ITM2B	GSTO1	LTA4H	CLDN7	GCNT3
CBX3	FCGBP	MRPL3	EIF3L	ANXA4	SRSF2
IFNGR2	LGALS3	HEXB	RPL10A	DYNLL1	B2M
SLC40A1	RAC1	GPX4	ANXA1	RPL12	GDI2
BACE2	CA2	MYL9	GNB2L1	SERF2	DYNLL1
QPCT	S100A14	UPP1	RAD21	ANXA2	STIP1
RPL6	FAU	MT2A	RPL19	FXYD3	IFI6
HSP90AB1	GCG	PRSS3	COX6C	TMEM258	LBR
TFF1	MGST1	SSR4	CCNI	RAB1A	HMGN3
ACSL1	PTP4A1	TAGLN2	RTN4	HLA-B	BANF1
TFF3	RPLP0	HSPA1A	TOP2A	BLVRB	VBP1
PRAC1	CMBL	IFITM3	APP	EEF1G	CKS2
ID3	TST	UQCRQ	RPS13	ATRAID	G0S2
CKS2	RPS29	HSPE1	CEBPB	DUSP5	PTTG1
CSTB	RPL6	RPL3	GAPDH	GUK1	RPS9
ITM2B	INSL5	SEPW1	SPARC	MSN	UHRF1
UCA1	ATP5C1	ARHGDIA	RPL11	RPS21	BCAP31
ITGAV	KCTD12	TUBA4A	EPCAM	EIF1	RAN
C10orf99	RPL41	RRM2	RPS18	CALB2	ADRM1
SERPINA1	PTGES3	TUBB4B	PRKACB	SLC22A18	PPIF
MMP1	AKR1C3	COX8A	LGALS1	TMED7	CTSH
GPI	RPS3	GABARAP	TUBB	FTH1P5	ALYREF
LYZ	RPS16	FAT1	RPS6	KRT18	YWHAB
TMPRSS3	IGLL3P	PGK1	RPL39	ADAM9	RPN2
YWHAG	ACAA1	EEF1B2	SGK1	ATP6V1F	UBE2C
MIF	PTTG1IP	YBX1	RPL5	CAMK2N1	IFITM1
PARK7	PABPC3	PSME1	ACTB	POLR2H	DDX21
PLCB4	NDRG1	COX4I1	RPS2	FXYD5	RPS20
S100A6	HSPA1A	CFL1	SDCBP	NEAT1	PPP1CC

C3	PARM1	IGFBP6	CD46	CRIP1	H2AFZ
MFSD4	RPL8	STRA13	NNMT	SLC2A3	TK1
CTSD	RPL11	NHP2	EIF4B	GPI	HLA-B
PFN1	MUC12	CHCHD2	NCOA4	MYL6	LY6E
DEK	NACAP1	EZR	EIF3E	VAMP8	PTPRO
PRSS23	SLC25A3	LGALS1	RGS2	INSIG1	HMGN2
ETS2	TNFSF10	ZFAS1	RPL21	TMEM147	MT2A
ATP1A1	CAPN2	MTCH1	RASSF3	CRIP2	RRM2
CD14	PYY	DSTN	NAP1L1	AGPAT2	LGALS3BP
AP1S1	DSP	RPL4	TSPAN8	PSMB5	PSME1
TIMP1	PRDX6	HSD17B2	RPL6	ARMCX6	TRMT5
ZWINT	ABCG2	FLNA	PDE4B	MZT2A	IDH3G
CEMIP	USMG5	GPRC5A	LASP1	SLC25A1	PRC1
PLP2	ERBB3	SNRPB	MYL12B	ETHE1	RBMX
IFNGR1	ARL6IP1	SH3BGRL3	RPL24	ID2	HMGN1
NOP58	MXI1	PSMB4	RPL13	TIMP1	TXNIP
PDIA3	RPS4Y1	FOSL1	SEP02	TM4SF1	TUBA1B
DPEP1	RPS4X	SERPINH1	RPS10	ID1	CDH1
TPI1	HLA-C	USMG5	CALM2	FAM96B	TIMM8B
ITM2C	GNE	COTL1	RPS19	S100A16	MCM7
CTSH	FTL	TYMS	RPS15A	CKB	AARS
LGALS3BP	CRIP1	RPL19	MYL6	C12orf57	GTF3A
ASCL2	LYPD8	ETFB	DKK1	UBC	DUT
RAN	PCK1	IRF7	EEF2	FDFT1	CFL1
SECTM1	LGALS2	MT1X	RPL36A	S100A10	KIF5B
RCN1	RPL12	PTDSS1	KRT18	CCNI	TOMM5
RNF43	HPGD	NDUFB11	NPTN	AP2M1	HSPE1
GLO1	CPA3	HSD17B10	FTL	MGST3	MYBL2
SLPI	TXN	HSPB1	QARS	PRDX5	HLA-G
MEST	VDAC1	CCNA2	YWHAQ	CD63	CEBPB
SEP15	HIGD1A	MRPS21	LDHB	HSPA5	DEK
	RPL13A	POLR2G	RPS25	TPT1	LDHB
	RARRES2	VDAC1	TPT1	LCP1	TPX2
	EEF1G	PLIN3	PTGES3	RPS27A	RPL30
	UGP2	VAMP8	PABPC3	RPL29	ATP6AP1
	TPSB2	PLEC	SRP14	EPB41L4A-AS1	CDKN3
	GNG5	APOBEC3B	SPINT2	CLIC1	RPL27A

HSP90AA1	U2AF1	ODC1	RAB25	CCT6A
UGDH	SSR2	RPS27L	FAM127A	LAPTM4B
RPS23	ATP6V1F	ASS1	SNRPB	TUBA1C
TSPAN7	CCT5		GSTO1	TOP2A
ACTN4	HSP90AA1		DBI	IARS
RPS27	UCHL3		NEU1	CRABP2
MPEG1	CDK4		S100A14	BST2
ATP5F1	NDUFA4		AHCY	RPL24
FABP5	MALL		RPL23	GGCT
RPL24	FIS1		ABLIM1	EIF2S2
HSD17B2	FOS		CD99	PTDSS1
MFSD4	MAFF		VDAC2	RPS8
S100A16	ADRM1		TOB1	EIF4A1
CA12	OAZ1		ACADVL	CYC1
GPX3	NDUFA1		F2RL1	MTHFD1
PABPC1	FTH1		LDHB	IER2
RPL36AL	HMGN2		FTH1	SUMO2
NDUFA1	PARP4		NPC2	TMA7
TP53INP2	NGRN		RPS14P3	MT1X
CAP1	YWHAZ		TCEAL4	PLSCR1
HUWE1	PAPSS1		CSTB	AHSA1
FGL2	GNB2		CHMP2A	PROM1
NACA	GOLT1A		SPINT2	CCT7
VDAC2	PHLDA2		UQCRFS1	EEF1D
COX4I1	CIB1		HLA-C	ERH
IGHM	SOD1		EFNB2	ZNF706
PRR13	PSMD8		NAA50	SEPW1
BSG	HSPH1		RABAC1	MT1H
YWHAE	IGFBP2		MDH2	PSMA4
	NDUFA13		TRIM28	TPI1
	ERH		CYB5R3	IFI27
	C14orf2		P4HB	NSA2
	MLF2		EMP1	YBX1
	CST6		SLC25A6	FEN1
	SDC4		RPS16	KRT18
	PUF60		DAP	IER3
	FABP5		ATP6V0E1	RAD21

RPL36	UQCR10	PABPC3
VDAC2	COX7A2L	RPL22L1
DYNLL1	CD55	MELK
CKS2	HSPB1	RPL19
MIF	PAM	NPTN
CYC1	RPL6	SET
UBA52	EPHX1	PSME2
PFN2	NDUFB5	MRPL16
COX6A1	TMBIM1	PNRC2
XRCC5	TSPAN1	CCT2
EBPL	SLC2A1	CXorf40A
JUN	RPS7	SRP9
RNF128	ALDOA	ARL6IP1
IARS	ISG15	TRMT11
FAM96B	ARF4	NPM1
SNRPG	JUP	FOS
DYNLT1	SEC61B	CISD1
PSMC5	PFN2	CD81
PDLIM1	IFI30	LMNB1
CCT7	RPL36A	IFITM3
TKT	SFXN3	SOD1
IFITM2	RTN4	UBE2S
UQCR11	RPL13	HSP90AA1
SNRPF	HPCAL1	KIAA0101
COX7A2L	DSTN	HSPA8
FBL	SEC61G	KRT8
TPI1	COPB1	PTPLAD1
UFD1L	LGALS1	NOP58
RPS15	ACTG1	MRPL37
RPL29	RPL3	RPL27
TRMT112	NDUFB3	HMGB2
CCT3	OST4	RPL21
TMEM258	VAT1	SLC3A2
PRMT1	EEF1A2	FAM35A
UXT	ACADM	PUF60
PSMB5	C20orf24	RRM1
C1QBP	AKR1B10	ETFA

TM9SF2
PSMB6
SLC35B1
RPL6
LDHA
RHOB
UBE2C
ISG15
SNRPD2
EIF3I
TMEM97
SNHG6
COX5B
PSMC1
CYFIP1
TUFT1
ZFP36
MT1HL1
KRT6A
JTB
S100A11
PHB
KARS
POLR1D
PRDX4
ILF2
CASP7
VIMP
PFDN5
UBE2N
HIGD2A

HERPUD1
SDCBP
ATP6V0D1
DYNLT1
RPS4X
TNFRSF21
YWHAZ
LTA4H
S100A6
RHOA
PTTG1IP

MT1F
PSMB8
HMGA1
CHORDC1
GPX1
CHCHD3
FBL
NDUFA4
ATIC
RPL4
EIF4A3
TM4SF4
ASUN

Supplementary Table 2: Gene Ontology (GO) annotations significantly linked with the co-DEGs in CRC vs the normal mucosa. The terms are sorted according to the combined score (Materials and methods).

Co-upregulated genes (Biological Process)

Term	Overlap	P-value	Adjusted P-value	Old P-value	Old Adjusted P-value	Odds Ratio	Combined Score	Genes
negative regulation of transforming growth factor beta production (GO:0071635)	3/6	1.06E-05	0.002466	0	0	60.97561	698.2679	CDH3;HSP90AB1;CD24
glomerular epithelial cell differentiation (GO:0072311)	3/6	1.06E-05	0.002359	0	0	60.97561	698.2679	PTPRO;PROM1;CD24
renal filtration cell differentiation (GO:0061318)	3/6	1.06E-05	0.002261	0	0	60.97561	698.2679	PTPRO;PROM1;CD24
negative regulation of glycoprotein biosynthetic process (GO:0010561)	3/7	1.85E-05	0.003254	0	0	52.26481	569.5831	BACE2;ITM2B;ITM2C
glycolytic process (GO:0006096)	6/23	2.49E-08	2.12E-05	0	0	31.81336	556.9478	LDHA;TPI1;PKM;PGAM1;ALDOA;PGM1
ATP generation from ADP (GO:0006757)	7/24	3.30E-08	2.41E-05	0	0	30.4878	525.1775	LDHA;TPI1;PKM;PGAM1;ALDOA;PGM1
glycolytic process through glucose-6-phosphate (GO:0061620)	6/25	4.32E-08	2.45E-05	0	0	29.26829	496.3366	GPI;TPI1;PKM;PGAM1;ALDOA;GAPDH
canonical glycolysis (GO:0061621)	7/25	4.32E-08	2.20E-05	0	0	29.26829	496.3366	GPI;TPI1;PKM;PGAM1;ALDOA;GAPDH
glucose catabolic process to pyruvate (GO:0061718)	6/25	4.32E-08	2.00E-05	0	0	29.26829	496.3366	GPI;TPI1;PKM;PGAM1;ALDOA;GAPDH
regulation of amyloid precursor protein biosynthetic process (GO:0042984)	3/9	4.39E-05	0.00746	0	0	40.65041	407.9121	BACE2;ITM2B;ITM2C

Co-upregulated genes (Cellular Component)

Term	Overlap	P-value	Adjusted P-value	Old P-value	Old Adjusted P-value	Odds Ratio	Combined Score	Genes
tertiary granule lumen (GO:1904724)	11/55	7.04E-13	1.57E-10	0	0	24.39024	682.4911	CSTB;TCN1;QPCT;CTSH;GGH;CXCL1;OLFM4;ALDOA;LYZ;CTSD;PGM1
secretory granule lumen (GO:0034774)	28/317	5.15E-21	2.30E-18	0	0	10.77172	503.2088	LGALS3BP;APP;GPI;CSTB;SPARC;HSP90AB1;SERPINA1;CXCL1;C3;CTSH;TIMP1;CTSD;S100A11; ANXA2;TUBB;PGAM1;GGH;MIF;OLFM4;LYZ;TUBB4B;PKM;SLPI;TCN1;QPCT;LCN2;S100P;ALDOA [illegible]
specific granule lumen (GO:0035580)	9/62	1.88E-09	1.68E-07	0	0	17.7026	355.6813	SLPI;TCN1;QPCT;LCN2;GGH;CXCL1;OLFM4;LYZ;CTSD

ficolin-1-rich granule lumen (GO:1904813)	12/123	4.04E-10	6.01E-08	0	0	11.89768	257.3438	GPI;CSTB;SERPINA1;HSP90AB1;PKM;PGAM1;QPCT;CTSH;MIF;ALDOA;CTSD;PGM1
cytoplasmic vesicle lumen (GO:0060205)	12/129	7.04E-10	7.85E-08	0	0	11.3443	239.0685	C3;GPI;CSTB;SPARC;HSP90AB1;PKM;PGAM1;CTSH;S100P;MIF;ALDOA;S100A11
ficolin-1-rich granule (GO:0101002)	13/184	4.04E-09	3.00E-07	0	0	8.616119	166.5331	GPI;CSTB;HSP90AB1;SERPINA1;PGAM1;MIF;DYNLL1;PKM;QPCT;CTSH;ALDOA;CTSD;PGM1
tertiary granule (GO:0070820)	12/164	1.10E-08	7.02E-07	0	0	8.92326	163.5136	CSTB;TCN1;QPCT;GGH;CTSH;CXCL1;OLFM4;LYZ;DYNLL1;ALDOA;CTSD;PGM1
specific granule (GO:0042581)	11/160	8.72E-08	4.86E-06	0	0	8.384146	136.2889	SLPI;TCN1;ANXA3;QPCT;LCN2;GGH;CXCL1;ITGAV;OLFM4;LYZ;CTSD
ribonucleoprotein granule (GO:0035770)	7/80	4.21E-06	2.09E-04	0	0	10.67073	132.0728	TUBA1A;TUBB;CTSH;PABPC1;RPL28;ACTB;RPL6
intrinsic component of external side of plasma membrane (GO:0031233)	3/22	7.43E-04	0.019503	0	0	16.62971	119.8051	CEACAM5;CD14;CD24

Co-upregulated genes (Molecular Function)

Term	Overlap	P-value	Adjusted P-value	Old P-value	Old Adjusted P-value	Odds Ratio	Combined Score	Genes
histone methyltransferase binding (GO:1990226)	2/6	9.81E-04	0.062731	0	0	40.65041	281.5822	HSP90AB1;CBX3
GPI anchor binding (GO:0034235)	2/7	0.001366	0.074871	0	0	34.84321	229.8205	CEACAM5;DPEP1
MHC class II protein complex binding (GO:0023026)	3/16	2.80E-04	0.035843	0	0	22.86585	187.0372	HSP90AB1;PKM;ATP1B1
CXCR chemokine receptor binding (GO:0045236)	3/17	3.38E-04	0.038936	0	0	21.5208	171.9863	CXCL8;TFF2;CXCL1
aspartic-type peptidase activity (GO:0070001)	3/17	3.38E-04	0.035397	0	0	21.5208	171.9863	BACE2;CTSE;CTSD
MHC protein complex binding (GO:0023023)	3/19	4.76E-04	0.045683	0	0	19.25546	147.2947	HSP90AB1;PKM;ATP1B1
Tat protein binding (GO:0030957)	2/10	0.00288	0.114315	0	0	24.39024	142.6802	NPM1;ACTB
aspartic-type endopeptidase activity (GO:0004190)	3/21	6.46E-04	0.057187	0	0	17.4216	127.9594	BACE2;CTSE;CTSD
sodium:potassium-exchanging ATPase activity (GO:0005391)	2/11	0.003501	0.12594	0	0	22.17295	125.3792	ATP1A1;ATP1B1
potassium-transporting ATPase activity (GO:0008556)	2/11	0.003501	0.122123	0	0	22.17295	125.3792	ATP1A1;ATP1B1

Co-downregulated genes (Biological Process)

Term	Overlap	P-value	Adjusted P-value	Old P-value	Old Adjusted P-value	Odds Ratio	Combined Score	Genes

cotranslational protein targeting to membrane (GO:0006613)	34/93	7.60E-45	3.88E-41	0	0	36.74285	3732.637	RPL30;RPL3;RPL32;ARL6IP1;RPL34;RPL12;RPLP0;RPL11;RPL8;RPS4Y1;RPL6;RPS4X; RPS16;RPS19;RPS3;RPL35;RPS2;RPS10;RPS13;RPL41;RPL23;RPS5;RPL22;RPL13A;RPSA; RPL23A;RPS26;RPS27;RPS29;RPL37A;RPL24;RPL27;RPS20;RPS23
SRP-dependent cotranslational protein targeting to membrane (GO:0006614)	33/89	8.85E-44	2.26E-40	0	0	37.26498	3694.218	RPL30;RPL3;RPL32;RPL34;RPL12;RPLP0;RPL11;RPL8;RPS4Y1;RPL6;RPS4X;RPS16;RPS19; RPS3;RPL35;RPS2;RPS10;RPS13;RPL41;RPL23;RPS5;RPL22;RPL13A;RPSA;RPL23A;RPS26; RPS27;RPS29;RPL37A;RPL24;RPL27;RPS20;RPS23
protein targeting to ER (GO:0045047)	33/97	2.71E-42	4.61E-39	0	0	34.19158	3272.563	RPL30;RPL3;RPL32;RPL34;RPL12;RPLP0;RPL11;RPL8;RPS4Y1;RPL6;RPS4X;RPS16;RPS19; RPS3;RPL35;RPS2;RPS10;RPS13;RPL41;RPL23;RPS5;RPL22;RPL13A;RPSA;RPL23A;RPS26; RPS27;RPS29;RPL37A;RPL24;RPL27;RPS20;RPS23
nuclear-transcribed mRNA catabolic process, nonsense-mediated decay (GO:0000184)	34/112	1.36E-41	1.74E-38	0	0	30.50969	2870.837	RPL30;RPL3;RPL32;RPL34;RPL12;RPLP0;RPL11;RPL8;RPS4Y1;RPL6;RPS4X;RPS16;RPS19; RPS3;RPL35;RPS2;RPS10;RPS13;RPL41;RPL23;RPS5;RPL22;RPL13A;RPSA;RPL23A;RPS26; RPS27;RPS29;RPL37A;RPL24;RPL27;RPS20;PABPC1;RPS23
viral gene expression (GO:0019080)	33/110	3.52E-40	3.59E-37	0	0	30.15075	2739.07	RPL30;RPL3;RPL32;RPL34;RPL12;RPLP0;RPL11;RPL8;RPS4Y1;RPL6;RPS4X;RPS16;RPS19; RPS3;RPL35;RPS2;RPS10;RPS13;RPL41;RPL23;RPS5;RPL22;RPL13A;RPSA;RPL23A;RPS26; RPS27;RPS29;RPL37A;RPL24;RPL27;RPS20;RPS23
viral transcription (GO:0019083)	33/113	9.78E-40	8.32E-37	0	0	29.35029	2636.328	RPL30;RPL3;RPL32;RPL34;RPL12;RPLP0;RPL11;RPL8;RPS4Y1;RPL6;RPS4X;RPS16;RPS19; RPS3;RPL35;RPS2;RPS10;RPS13;RPL41;RPL23;RPS5;RPL22;RPL13A;RPSA;RPL23A;RPS26; RPS27;RPS29;RPL37A;RPL24;RPL27;RPS20;RPS23
nuclear-transcribed mRNA catabolic process (GO:0000956)	34/174	2.26E-34	1.65E-31	0	0	19.63842	1521.417	RPL30;RPL3;RPL32;RPL34;RPL12;RPLP0;RPL11;RPL8;RPS4Y1;RPL6;RPS4X;RPS16;RPS19; RPS3;RPL35;RPS2;RPS10;RPS13;RPL41;RPL23;RPS5;RPL22;RPL13A;RPSA;RPL23A;RPS26; RPS27;RPS29;RPL37A;RPL24;RPL27;RPS20;PABPC1;RPS23
peptide biosynthetic process (GO:0043043)	33/174	6.47E-33	4.13E-30	0	0	19.06082	1412.744	RPL30;RPL3;RPL32;RPL34;RPL12;RPLP0;RPL11;RPL8;RPS4Y1;RPL6;RPS4X;RPS16;RPS19;RPS3; RPL35;RPS2;RPS10;RPS13;RPL41;RPL23;RPS5;RPL22;RPL13A;RPSA;RPL23A;RPS26;RPS27; RPS29;RPL37A;RPL24;RPL27;RPS20;RPS23
rRNA metabolic process (GO:0016072)	33/200	8.12E-31	4.60E-28	0	0	16.58291	1148.969	RPL30;RPL3;RPL32;RPL34;RPL12;RPLP0;RPL11;RPL8;RPS4Y1;RPL6;RPS4X;RPS16;RPS19;RPS3; RPL35;RPS2;RPS10;RPS13;RPL41;RPL23;RPS5;RPL22;RPL13A;RPSA;RPL23A;RPS26;RPS27; RPS29;RPL37A;RPL24;RPL27;RPS20;RPS23
rRNA processing (GO:0006364)	33/202	1.14E-30	5.30E-28	0	0	16.41873	1131.987	RPL30;RPL3;RPL32;RPL34;RPL12;RPLP0;RPL11;RPL8;RPS4Y1;RPL6;RPS4X;RPS16;RPS19; RPS3;RPL35;RPS2;RPS10;RPS13;RPL41;RPL23;RPS5;RPL22;RPL13A;RPSA;RPL23A;RPS26; RPS27;RPS29;RPL37A;RPL24;RPL27;RPS20;RPS23

Co-downregulated genes (Cellular Component)

Term	Overlap	P-value	Adjusted P-value	Old P-value	Old Adjusted P-value	Odds Ratio	Combined Score	Genes
cytosolic ribosome (GO:0022626)	34/124	7.20E-40	3.21E-37	0	0	27.55714	2483.718	RPL30;RPL3;RPL32;RPL34;RPL12;RPLP0;RPL11;RPL8;RPS4Y1;RPL6;RPS4X;RPS16;RPS19; RPL36AL;RPS3;RPL35; RPS2;RPS10;RPS13;RPL41;RPL23;RPS5;RPL22;RPL13A;RPSA;RPL23A;RPS26;RPS27;RPS29;

								RPL37A;RPL24;RPL27;RPS20;RPS23
cytosolic part (GO:0044445)	34/159	8.38E-36	1.87E-33	0	0	21.4911	1735.787	RPL30;RPL3;RPL32;RPL34;RPL12;RPLP0;RPL11;RPL8;RPS4Y1;RPL6;RPS4X;RPS16;RPS19; RPL36AL;RPS3;RPL35;RPS2;RPS10;RPS13;RPL41;RPL23;RPS5;RPL22;RPL13A;RPSA;RPL23A;RPS2 RPS20;RPS23
ribosome (GO:0005840)	21/76	5.53E-25	8.22E-23	0	0	27.77043	1551.115	RPL30;RPL41;RPL32;RPL23;RPS5;RPL12;RPL11;RPL13A;RPL8;RPL6;RPS4X;RPS26;RPS27;RPS19; RPS29;RPS3; RPL24;RPL27;RPS10;RPS13;RPS23
cytosolic large ribosomal subunit (GO:0022625)	19/69	1.13E-22	1.27E-20	0	0	27.6746	1398.412	RPL30;RPL41;RPL3;RPL32;RPL34;RPL23;RPL12;RPLP0;RPL22;RPL11;RPL13A;RPL23A;RPL8; RPL6;RPL36AL; RPL37A;RPL35;RPL24;RPL27
large ribosomal subunit (GO:0015934)	19/72	2.82E-22	2.09E-20	0	0	26.5215	1316.044	RPL30;RPL41;RPL3;RPL32;RPL34;RPL23;RPL12;RPLP0;RPL22;RPL11;RPL13A;RPL23A;RPL8; RPL6;RPL36AL; RPL37A;RPL35;RPL24;RPL27
cytosolic small ribosomal subunit (GO:0022627)	15/49	6.36E-19	4.05E-17	0	0	30.76608	1289.05	RPS5;RPSA;RPS4Y1;RPS4X;RPS26;RPS16;RPS27;RPS19;RPS29;RPS3;RPS20;RPS2;RPS10;RPS13; RPS23
small ribosomal subunit (GO:0015935)	15/53	2.44E-18	1.36E-16	0	0	28.44411	1153.552	RPS5;RPSA;RPS4Y1;RPS4X;RPS26;RPS16;RPS27;RPS19;RPS29;RPS3;RPS20;RPS2;RPS10;RPS13; RPS23
polysomal ribosome (GO:0042788)	10/28	8.51E-14	3.79E-12	0	0	35.89375	1080.233	RPS26;RPL30;RPL41;RPL32;RPS29;RPL11;RPL24;RPL8;RPL6;RPS23
polysome (GO:0005844)	14/63	1.43E-15	7.10E-14	0	0	22.33389	763.3602	RPL30;RPL41;RPL32;RPL11;RPL8;RPS4Y1;RPL6;RPS4X;RPS26;RPS29;RPS3;RPL24;VIM;RPS23
focal adhesion (GO:0005925)	33/356	1.47E-22	1.32E-20	0	0	9.316244	468.3121	YWHAE;CYFIP1;RPL30;RPL3;RPL12;RPLP0;RPL8;RPL6;RPS4X;PDLIM1;RPS16;RPS19;BSG;CFL1; RPS3;CAPN2;RAC1;RPS2;RPS10;RPS13;CAP1;RPL23;RPS5;RPL22;RPL13A;ACTN4;RPS29;RPL37A RPL27;PABPC1;VIM;PFN1;HSPA1A

Co-downregulated genes (Molecular Function)

Term	Overlap	P-value	Adjusted P-value	Old P-value	Old Adjusted P-value	Odds Ratio	Combined Score	Genes
acetyl-CoA C-acyltransferase activity (GO:0003988)	3/6	1.90E-05	0.003642	0	0	50.25126	546.331	HADHB;ACAA2;ACAA1
rRNA binding (GO:0019843)	7/43	2.07E-07	7.93E-05	0	0	16.36087	251.8283	TST;RPL12;RPL23;RPS5;RPL11;RPS3;RPS13
ubiquitin-protein transferase inhibitor activity (GO:0055105)	2/6	0.001439	0.127427	0	0	33.50084	219.2177	RPL23;RPL11
C-acyltransferase activity (GO:0016408)	3/12	2.00E-04	0.022996	0	0	25.12563	214.0258	HADHB;ACAA2;ACAA1
RNA binding (GO:0003723)	57/1387	6.86E-21	7.90E-18	0	0	4.13024	191.7598	YWHAE;RPL30;RPL3;ACAA2;RPL32;RPL34;RPLP0;ATP5C1;RPL8;RPL6;RPS4X;LGALS3; LGALS1;RPS16;RPS19;RPL35;RPS10;LBR;RPS13;DSP;HSP90AA1;RPL23;RPS5;RPL22; RPL13A;RPSA;ACTN4;HADHB;RPL37A;RPL24;RPL27;SUCLG1;KCTD12;PABPC1;PFN1; SLC25A5;RPL12;ATP5A1;RPL11;TXN;RPS4Y1;S100A16;PCBP1;RPS3;RPS2;RPL41;MDH2; HUWE1;RPL23A;RPS26;RPS27;RPS20;FAU;VIM;TPT1;RPS23;HSPA1A

retinal dehydrogenase activity (GO:0001758)	2/7	0.002002	0.164571	0	0	28.715	178.4276	AKR1B10;AKR1C3
small ribosomal subunit rRNA binding (GO:0070181)	2/7	0.002002	0.1536	0	0	28.715	178.4276	RPS3;RPS13
estradiol 17-beta-dehydrogenase activity (GO:0004303)	2/7	0.002002	0.144	0	0	28.715	178.4276	DHRS11;HSD17B2
oxidoreductase activity, acting on the CH-OH group of donors, NAD or NADP as acceptor (GO:0016616)	9/88	2.31E-07	6.65E-05	0	0	10.27867	157.0548	HADHB;UGDH;DHRS11;AKR1B10;ADH1C;HPGD;MDH2;AKR1C3;HADH
3-hydroxyacyl-CoA dehydrogenase activity (GO:0003857)	2/8	0.002652	0.16955	0	0	25.12563	149.0608	HADHB;HADH

Supplementary Table 3: KEGG enrichment analysis for the co-DEGs in CRC vs the normal mucosa. The terms are sorted according to the combined score (Materials and methods).

Co-upregulated genes

Term	Overlap	P-value	Adjusted P-value	Old P-value	Old Adjusted P-value	Odds Ratio	Combined Score
Glycolysis / Gluconeogenesis	8/68	8.382839318826976E-8	1.2909572550993542E-5	0	0	14.347202295552366	233.78040266833273
Pathogenic Escherichia coli infection	7/55	3.1958375260905826E-7	3.2810598601196646E-5	0	0	15.521064301552103	232.13686303087158
IL-17 signaling pathway	9/93	7.177340958809777E-8	2.2106210153134113E-5	0	0	11.801730920535011	194.1355440930409
Salmonella infection	8/86	5.310961514014025E-7	4.089440365790799E-5	0	0	11.344299489506522	163.90610046524404
Amoebiasis	7/96	1.4168809425148827E-5	7.273322171576398E-4	0	0	8.892276422764228	99.27753137728445
HIF-1 signaling pathway	7/100	1.851459309935705E-5	8.146420963717102E-4	0	0	8.536585365853659	93.0227551762848
Phagosome	9/152	4.635343488104684E-6	2.855371588672485E-4	0	0	7.220795892169447	88.68437282416313
Gap junction	6/88	8.62870136388604E-5	0.002952933355641	0	0	8.314855875831483	77.80901982097814
Pentose phosphate pathway	3/30	0.0018679317590660574	0.028766149089617284	0	0	12.195121951219512	76.62101794380487
Rheumatoid arthritis	6/91	1.0400824948934093E-4	0.003203454084271701	0	0	8.04073974805682	73.7419485925154

Co-downregulated genes

Term	Overlap	P-value	Adjusted P-value	Old P-value	Old Adjusted P-value	Odds Ratio	Combined Score
Ribosome	36/153	1.5690268268550238E-39	4.832602626713473E-37	0	0	23.64765001477978	2112.9261142206037
Fatty acid degradation	8/44	1.0878919849877325E-8	1.6753536568811081E-6	0	0	18.273184102329832	335.06512341618964
Valine, leucine and isoleucine degradation	7/48	4.528030490441448E-7	4.648777970186553E-5	0	0	14.656616415410385	214.10104697294307
Ascorbate and aldarate metabolism	4/27	1.3948520722214603E-4	0.007160240637403496	0	0	14.889261120416897	132.18018989382392
Sulfur metabolism	2/9	0.003386820924245468	0.061361226156917886	0	0	22.333891680625346	127.03212904610908
Ferroptosis	5/40	4.597935033106852E-5	0.003540409975492276	0	0	12.562814070351758	125.4688212074109

Pentose and glucuronate interconversions	4/34	3.490758792787917E-4	0.013439421352233482	0	0	11.82382500738989	94.12026296490716
Pyruvate metabolism	4/39	5.955574555022006E-4	0.020381299588297527	0	0	10.307950006442468	76.54696756636325
Fatty acid elongation	3/27	0.0023800271945585666	0.06108736466033654	0	0	11.166945840312673	67.45553729848041
Citrate cycle (TCA cycle)	3/30	0.003232078335469868	0.06221750795779495	0	0	10.050251256281408	57.63447136841482

Supplementary Table 4: Protein-protein interaction (PPI) sub-network around proteins encoded by the co-upregulated genes in CRC vs the normal mucosa.

G2N

Source	Source type	Target	Target type
ZNF281	Intermediate protein	SOX2	Seed protein
ZNF281	Intermediate protein	NANOG	Seed protein
ZNF281	Intermediate protein	POU5F1	Intermediate protein
ZBTB16	Intermediate protein	VDR	Intermediate protein
ZBTB16	Intermediate protein	ZBTB16	Intermediate protein
ZBTB16	Intermediate protein	PML	Seed protein
ZBTB16	Intermediate protein	NCOR1	Intermediate protein
ZBTB16	Intermediate protein	NCOR2	Intermediate protein
ZBTB16	Intermediate protein	PARP1	Intermediate protein
ZBTB16	Intermediate protein	CEBPA	Intermediate protein
ZBTB16	Intermediate protein	HDAC5	Intermediate protein
ZBTB16	Intermediate protein	HDAC3	Intermediate protein
ZBTB16	Intermediate protein	HDAC2	Intermediate protein
ZBTB16	Intermediate protein	GATA2	Intermediate protein
ZBTB16	Intermediate protein	HDAC7	Intermediate protein
ZBTB16	Intermediate protein	SUMO1	Intermediate protein
ZBTB16	Intermediate protein	SIN3A	Intermediate protein
MSH2	Intermediate protein	MSH2	Intermediate protein
MSH2	Intermediate protein	RAD51	Intermediate protein
MSH2	Intermediate protein	CHEK2	Intermediate protein
MSH2	Intermediate protein	MYC	Seed protein
SMARCA4	Intermediate protein	SMARCA4	Intermediate protein
SMARCA4	Intermediate protein	PML	Seed protein
SMARCA4	Intermediate protein	RB1	Intermediate protein

SMARCA4	Intermediate protein	SMAD2	Intermediate protein
SMARCA4	Intermediate protein	HIF1A	Intermediate protein
SMARCA4	Intermediate protein	SP1	Intermediate protein
SMARCA4	Intermediate protein	NFE2L2	Intermediate protein
SMARCA4	Intermediate protein	CDKN2A	Intermediate protein
SMARCA4	Intermediate protein	STAT1	Intermediate protein
SMARCA4	Intermediate protein	H2AFX	Intermediate protein
SMARCA4	Intermediate protein	STAT3	Intermediate protein
SMARCA4	Intermediate protein	CEBPA	Intermediate protein
SMARCA4	Intermediate protein	CEBPB	Intermediate protein
SMARCA4	Intermediate protein	HDAC3	Intermediate protein
SMARCA4	Intermediate protein	HDAC2	Intermediate protein
SMARCA4	Intermediate protein	SETD7	Intermediate protein
SMARCA4	Intermediate protein	SIN3A	Intermediate protein
SMARCA4	Intermediate protein	TRIM28	Seed protein
PML	Seed protein	ZBTB16	Intermediate protein
PML	Seed protein	SMARCA4	Intermediate protein
PML	Seed protein	PML	Seed protein
PML	Seed protein	SKI	Intermediate protein
PML	Seed protein	KAT6A	Intermediate protein
PML	Seed protein	RBCK1	Intermediate protein
PML	Seed protein	RELA	Intermediate protein
PML	Seed protein	RB1	Intermediate protein
PML	Seed protein	RPL11	Intermediate protein
PML	Seed protein	CCNT1	Intermediate protein
PML	Seed protein	UBE3A	Intermediate protein
PML	Seed protein	EGFR	Intermediate protein
PML	Seed protein	FOXO1	Intermediate protein
PML	Seed protein	RNF4	Intermediate protein
PML	Seed protein	CHEK2	Intermediate protein
PML	Seed protein	MYC	Seed protein
PML	Seed protein	E2F4	Intermediate protein
PML	Seed protein	PPARGC1A	Intermediate protein
PML	Seed protein	SMAD3	Intermediate protein
PML	Seed protein	SMAD2	Intermediate protein
PML	Seed protein	DAXX	Intermediate protein
PML	Seed protein	PIAS1	Intermediate protein

PML	Seed protein	PIAS2	Intermediate protein
PML	Seed protein	PIN1	Intermediate protein
PML	Seed protein	EZH2	Intermediate protein
PML	Seed protein	SPI1	Intermediate protein
PML	Seed protein	CUL3	Intermediate protein
PML	Seed protein	CUL1	Intermediate protein
PML	Seed protein	GATA2	Intermediate protein
PML	Seed protein	KAT5	Intermediate protein
PML	Seed protein	SKP1	Intermediate protein
PML	Seed protein	NCOA2	Intermediate protein
PML	Seed protein	JUN	Intermediate protein
PML	Seed protein	SETDB1	Intermediate protein
PML	Seed protein	SIRT1	Intermediate protein
PML	Seed protein	NCOR1	Intermediate protein
PML	Seed protein	SP1	Intermediate protein
PML	Seed protein	NCOR2	Intermediate protein
PML	Seed protein	CDK2	Intermediate protein
PML	Seed protein	BCL2	Intermediate protein
PML	Seed protein	SUV39H1	Intermediate protein
PML	Seed protein	NR2C1	Intermediate protein
PML	Seed protein	SKIL	Intermediate protein
PML	Seed protein	TBX2	Intermediate protein
PML	Seed protein	CDKN2A	Intermediate protein
PML	Seed protein	STAT1	Intermediate protein
PML	Seed protein	CSNK2A1	Intermediate protein
PML	Seed protein	H2AFX	Intermediate protein
PML	Seed protein	ATRX	Intermediate protein
PML	Seed protein	STAT3	Intermediate protein
PML	Seed protein	FOS	Intermediate protein
PML	Seed protein	ARNT	Intermediate protein
PML	Seed protein	RAD51	Intermediate protein
PML	Seed protein	HDAC3	Intermediate protein
PML	Seed protein	HDAC2	Intermediate protein
PML	Seed protein	UHRF1	Intermediate protein
PML	Seed protein	EHMT2	Intermediate protein
PML	Seed protein	HDAC7	Intermediate protein
PML	Seed protein	SUMO1	Intermediate protein

PML	Seed protein	SIN3A	Intermediate protein
PML	Seed protein	TDG	Intermediate protein
PML	Seed protein	MAPK3	Intermediate protein
PML	Seed protein	MAPK1	Intermediate protein
PML	Seed protein	IKBKE	Intermediate protein
PML	Seed protein	TRIM24	Intermediate protein
SKI	Intermediate protein	SMAD3	Intermediate protein
SKI	Intermediate protein	SMAD2	Intermediate protein
SKI	Intermediate protein	JUN	Intermediate protein
SKI	Intermediate protein	SIRT1	Intermediate protein
SKI	Intermediate protein	PML	Seed protein
SKI	Intermediate protein	SKI	Intermediate protein
SKI	Intermediate protein	NCOR1	Intermediate protein
SKI	Intermediate protein	RB1	Intermediate protein
SKI	Intermediate protein	HDAC3	Intermediate protein
SKI	Intermediate protein	SPI1	Intermediate protein
SKI	Intermediate protein	SIN3A	Intermediate protein
SKI	Intermediate protein	SKIL	Intermediate protein
PSMC5	Intermediate protein	VDR	Intermediate protein
PSMC5	Intermediate protein	SMURF1	Intermediate protein
PSMC5	Intermediate protein	SP1	Intermediate protein
PSMC5	Intermediate protein	PSMC5	Intermediate protein
PSMC5	Intermediate protein	MYC	Seed protein
MAPKAPK2	Intermediate protein	PIAS2	Intermediate protein
MAPKAPK2	Intermediate protein	MAPKAPK2	Intermediate protein
MAPKAPK2	Intermediate protein	TCF3	Seed protein
MAPKAPK2	Intermediate protein	MAPK3	Intermediate protein
MAPKAPK2	Intermediate protein	MAPK1	Intermediate protein
MAPKAPK2	Intermediate protein	TRIM28	Seed protein
KAT6A	Intermediate protein	PML	Seed protein
RBCK1	Intermediate protein	PML	Seed protein
RBCK1	Intermediate protein	RBCK1	Intermediate protein
CALM1	Intermediate protein	EGFR	Intermediate protein
CALM1	Intermediate protein	TCF3	Seed protein
CALM1	Intermediate protein	HSP90AA1	Intermediate protein
CALM1	Intermediate protein	CSNK2A1	Intermediate protein
RELA	Intermediate protein	PML	Seed protein

RELA	Intermediate protein	RELA	Intermediate protein
RELA	Intermediate protein	MYC	Seed protein
RELA	Intermediate protein	PPARGC1A	Intermediate protein
RELA	Intermediate protein	DAXX	Intermediate protein
RELA	Intermediate protein	EZH2	Intermediate protein
RELA	Intermediate protein	HIF1A	Intermediate protein
RELA	Intermediate protein	KAT5	Intermediate protein
RELA	Intermediate protein	MEN1	Intermediate protein
RELA	Intermediate protein	JUN	Intermediate protein
RELA	Intermediate protein	SIRT1	Intermediate protein
RELA	Intermediate protein	SP1	Intermediate protein
RELA	Intermediate protein	NCOR2	Intermediate protein
RELA	Intermediate protein	BCL2	Intermediate protein
RELA	Intermediate protein	TBP	Intermediate protein
RELA	Intermediate protein	CHUK	Intermediate protein
RELA	Intermediate protein	CSNK2A1	Intermediate protein
RELA	Intermediate protein	STAT3	Intermediate protein
RELA	Intermediate protein	PARP1	Intermediate protein
RELA	Intermediate protein	FOS	Intermediate protein
RELA	Intermediate protein	KLF4	Seed protein
RELA	Intermediate protein	STUB1	Intermediate protein
RELA	Intermediate protein	CEBPB	Intermediate protein
RELA	Intermediate protein	HDAC3	Intermediate protein
RELA	Intermediate protein	HDAC2	Intermediate protein
RELA	Intermediate protein	SETD7	Intermediate protein
RELA	Intermediate protein	IKBKE	Intermediate protein
RB1	Intermediate protein	SMARCA4	Intermediate protein
RB1	Intermediate protein	PML	Seed protein
RB1	Intermediate protein	SKI	Intermediate protein
RB1	Intermediate protein	RB1	Intermediate protein
RB1	Intermediate protein	CHEK2	Intermediate protein
RB1	Intermediate protein	E2F1	Intermediate protein
RB1	Intermediate protein	MYC	Seed protein
RB1	Intermediate protein	E2F4	Intermediate protein
RB1	Intermediate protein	TCF3	Seed protein
RB1	Intermediate protein	DNMT1	Intermediate protein
RB1	Intermediate protein	SPI1	Intermediate protein

RB1	Intermediate protein	HIF1A	Intermediate protein
RB1	Intermediate protein	KAT5	Intermediate protein
RB1	Intermediate protein	SKP2	Intermediate protein
RB1	Intermediate protein	VDR	Intermediate protein
RB1	Intermediate protein	JUN	Intermediate protein
RB1	Intermediate protein	DNMT3A	Intermediate protein
RB1	Intermediate protein	SIRT1	Intermediate protein
RB1	Intermediate protein	KAT2B	Intermediate protein
RB1	Intermediate protein	SP1	Intermediate protein
RB1	Intermediate protein	CDK2	Intermediate protein
RB1	Intermediate protein	SUV39H1	Intermediate protein
RB1	Intermediate protein	SKIL	Intermediate protein
RB1	Intermediate protein	TBP	Intermediate protein
RB1	Intermediate protein	STAT3	Intermediate protein
RB1	Intermediate protein	FOS	Intermediate protein
RB1	Intermediate protein	ARNT	Intermediate protein
RB1	Intermediate protein	CEBPB	Intermediate protein
RB1	Intermediate protein	HDAC3	Intermediate protein
RB1	Intermediate protein	HDAC2	Intermediate protein
RB1	Intermediate protein	UHRF1	Intermediate protein
RB1	Intermediate protein	SETD7	Intermediate protein
RPL11	Intermediate protein	MYC	Seed protein
RPL11	Intermediate protein	VHL	Intermediate protein
RPL11	Intermediate protein	PML	Seed protein
TRRAP	Intermediate protein	KAT2B	Intermediate protein
TRRAP	Intermediate protein	CUL1	Intermediate protein
TRRAP	Intermediate protein	KAT5	Intermediate protein
TRRAP	Intermediate protein	E2F1	Intermediate protein
TRRAP	Intermediate protein	MYC	Seed protein
TRRAP	Intermediate protein	E2F4	Intermediate protein
TRRAP	Intermediate protein	SKP1	Intermediate protein
CCNT1	Intermediate protein	NCOA2	Intermediate protein
CCNT1	Intermediate protein	SMAD3	Intermediate protein
CCNT1	Intermediate protein	PML	Seed protein
CCNT1	Intermediate protein	KAT2B	Intermediate protein
CCNT1	Intermediate protein	CCNT1	Intermediate protein
CCNT1	Intermediate protein	MYC	Seed protein

CCNH	Intermediate protein	MYC	Seed protein
CCNH	Intermediate protein	CDK2	Intermediate protein
UBE3A	Intermediate protein	PML	Seed protein
UBE3A	Intermediate protein	UBE3A	Intermediate protein
UBE3A	Intermediate protein	CEBPA	Intermediate protein
EGFR	Intermediate protein	PML	Seed protein
EGFR	Intermediate protein	CALM1	Intermediate protein
EGFR	Intermediate protein	EGFR	Intermediate protein
EGFR	Intermediate protein	PRKDC	Intermediate protein
EGFR	Intermediate protein	HSP90AA1	Intermediate protein
EGFR	Intermediate protein	STAT1	Intermediate protein
EGFR	Intermediate protein	STAT3	Intermediate protein
EGFR	Intermediate protein	H2AFX	Intermediate protein
EGFR	Intermediate protein	STUB1	Intermediate protein
EGFR	Intermediate protein	CEBPB	Intermediate protein
RNF4	Intermediate protein	TBP	Intermediate protein
RNF4	Intermediate protein	PML	Seed protein
RNF4	Intermediate protein	SP1	Intermediate protein
RNF4	Intermediate protein	NFE2L2	Intermediate protein
RNF4	Intermediate protein	UHRF1	Intermediate protein
RNF4	Intermediate protein	RNF4	Intermediate protein
RNF4	Intermediate protein	SUMO1	Intermediate protein
RNF4	Intermediate protein	TDG	Intermediate protein
RNF4	Intermediate protein	TRIM28	Seed protein
FOXO1	Intermediate protein	SMAD3	Intermediate protein
FOXO1	Intermediate protein	PARP1	Intermediate protein
FOXO1	Intermediate protein	SIRT1	Intermediate protein
FOXO1	Intermediate protein	PML	Seed protein
FOXO1	Intermediate protein	STUB1	Intermediate protein
FOXO1	Intermediate protein	CEBPB	Intermediate protein
FOXO1	Intermediate protein	SKP2	Intermediate protein
MYC	Seed protein	MSH2	Intermediate protein
MYC	Seed protein	PML	Seed protein
MYC	Seed protein	PSMC5	Intermediate protein
MYC	Seed protein	RELA	Intermediate protein
MYC	Seed protein	RB1	Intermediate protein
MYC	Seed protein	TRRAP	Intermediate protein

MYC	Seed protein	RPL11	Intermediate protein
MYC	Seed protein	CCNT1	Intermediate protein
MYC	Seed protein	CCNH	Intermediate protein
MYC	Seed protein	MYC	Seed protein
MYC	Seed protein	SMAD3	Intermediate protein
MYC	Seed protein	SMAD2	Intermediate protein
MYC	Seed protein	PRKCD	Intermediate protein
MYC	Seed protein	PIAS2	Intermediate protein
MYC	Seed protein	PIN1	Intermediate protein
MYC	Seed protein	CUL1	Intermediate protein
MYC	Seed protein	PRKDC	Intermediate protein
MYC	Seed protein	HIF1A	Intermediate protein
MYC	Seed protein	KAT5	Intermediate protein
MYC	Seed protein	SKP1	Intermediate protein
MYC	Seed protein	SKP2	Intermediate protein
MYC	Seed protein	VDR	Intermediate protein
MYC	Seed protein	HSP90AA1	Intermediate protein
MYC	Seed protein	JUN	Intermediate protein
MYC	Seed protein	DNMT3A	Intermediate protein
MYC	Seed protein	SIRT1	Intermediate protein
MYC	Seed protein	KAT2B	Intermediate protein
MYC	Seed protein	SP1	Intermediate protein
MYC	Seed protein	NCOR1	Intermediate protein
MYC	Seed protein	CDK2	Intermediate protein
MYC	Seed protein	BCL2	Intermediate protein
MYC	Seed protein	NFE2L2	Intermediate protein
MYC	Seed protein	ATAD2	Intermediate protein
MYC	Seed protein	TBP	Intermediate protein
MYC	Seed protein	CDKN2A	Intermediate protein
MYC	Seed protein	CHUK	Intermediate protein
MYC	Seed protein	CSNK2A1	Intermediate protein
MYC	Seed protein	TTC5	Intermediate protein
MYC	Seed protein	STUB1	Intermediate protein
MYC	Seed protein	PFDN5	Intermediate protein
MYC	Seed protein	CEBPA	Intermediate protein
MYC	Seed protein	CEBPB	Intermediate protein
MYC	Seed protein	HDAC3	Intermediate protein

MYC	Seed protein	HDAC2	Intermediate protein
MYC	Seed protein	TUBA1A	Intermediate protein
MYC	Seed protein	MAPK3	Intermediate protein
MYC	Seed protein	VHL	Intermediate protein
MYC	Seed protein	MAPK1	Intermediate protein
CHEK2	Intermediate protein	MSH2	Intermediate protein
CHEK2	Intermediate protein	PML	Seed protein
CHEK2	Intermediate protein	KAT2B	Intermediate protein
CHEK2	Intermediate protein	RB1	Intermediate protein
CHEK2	Intermediate protein	CUL1	Intermediate protein
CHEK2	Intermediate protein	PRKDC	Intermediate protein
CHEK2	Intermediate protein	CHEK2	Intermediate protein
CHEK2	Intermediate protein	VHL	Intermediate protein
CHEK2	Intermediate protein	TRIM28	Seed protein
E2F1	Intermediate protein	RB1	Intermediate protein
E2F1	Intermediate protein	TRRAP	Intermediate protein
E2F1	Intermediate protein	DNMT1	Intermediate protein
E2F1	Intermediate protein	CUL1	Intermediate protein
E2F1	Intermediate protein	KAT5	Intermediate protein
E2F1	Intermediate protein	SKP2	Intermediate protein
E2F1	Intermediate protein	SIRT1	Intermediate protein
E2F1	Intermediate protein	SP1	Intermediate protein
E2F1	Intermediate protein	KAT2B	Intermediate protein
E2F1	Intermediate protein	CDK2	Intermediate protein
E2F1	Intermediate protein	TBP	Intermediate protein
E2F1	Intermediate protein	ATAD2	Intermediate protein
E2F1	Intermediate protein	CDKN2A	Intermediate protein
E2F1	Intermediate protein	PARP1	Intermediate protein
E2F1	Intermediate protein	SETD7	Intermediate protein
E2F1	Intermediate protein	VHL	Intermediate protein
E2F1	Intermediate protein	TRIM28	Seed protein
E2F4	Intermediate protein	ATAD2	Intermediate protein
E2F4	Intermediate protein	SMAD3	Intermediate protein
E2F4	Intermediate protein	PML	Seed protein
E2F4	Intermediate protein	NCOR2	Intermediate protein
E2F4	Intermediate protein	RB1	Intermediate protein
E2F4	Intermediate protein	HDAC3	Intermediate protein

E2F4	Intermediate protein	TRRAP	Intermediate protein
E2F4	Intermediate protein	XPO1	Intermediate protein
E2F4	Intermediate protein	SIN3A	Intermediate protein
E2F4	Intermediate protein	TRIM28	Seed protein
PPARGC1A	Intermediate protein	SIRT1	Intermediate protein
PPARGC1A	Intermediate protein	PML	Seed protein
PPARGC1A	Intermediate protein	RELA	Intermediate protein
PPARGC1A	Intermediate protein	PIAS1	Intermediate protein
PPARGC1A	Intermediate protein	SKP1	Intermediate protein
SMAD3	Intermediate protein	PML	Seed protein
SMAD3	Intermediate protein	SKI	Intermediate protein
SMAD3	Intermediate protein	CCNT1	Intermediate protein
SMAD3	Intermediate protein	FOXO1	Intermediate protein
SMAD3	Intermediate protein	MYC	Seed protein
SMAD3	Intermediate protein	E2F4	Intermediate protein
SMAD3	Intermediate protein	SMAD3	Intermediate protein
SMAD3	Intermediate protein	SMAD2	Intermediate protein
SMAD3	Intermediate protein	PIAS2	Intermediate protein
SMAD3	Intermediate protein	PIN1	Intermediate protein
SMAD3	Intermediate protein	CUL1	Intermediate protein
SMAD3	Intermediate protein	HIF1A	Intermediate protein
SMAD3	Intermediate protein	MEN1	Intermediate protein
SMAD3	Intermediate protein	VDR	Intermediate protein
SMAD3	Intermediate protein	JUN	Intermediate protein
SMAD3	Intermediate protein	KAT2B	Intermediate protein
SMAD3	Intermediate protein	SP1	Intermediate protein
SMAD3	Intermediate protein	CDK2	Intermediate protein
SMAD3	Intermediate protein	SUV39H1	Intermediate protein
SMAD3	Intermediate protein	SKIL	Intermediate protein
SMAD3	Intermediate protein	CHUK	Intermediate protein
SMAD3	Intermediate protein	FOS	Intermediate protein
SMAD3	Intermediate protein	NFIC	Seed protein
SMAD3	Intermediate protein	STUB1	Intermediate protein
SMAD3	Intermediate protein	HDAC5	Intermediate protein
SMAD3	Intermediate protein	CEBPA	Intermediate protein
SMAD3	Intermediate protein	CEBPB	Intermediate protein
SMAD3	Intermediate protein	SIN3A	Intermediate protein

SMAD2	Intermediate protein	SMARCA4	Intermediate protein
SMAD2	Intermediate protein	PML	Seed protein
SMAD2	Intermediate protein	SKI	Intermediate protein
SMAD2	Intermediate protein	MYC	Seed protein
SMAD2	Intermediate protein	SMAD3	Intermediate protein
SMAD2	Intermediate protein	SMAD2	Intermediate protein
SMAD2	Intermediate protein	PIN1	Intermediate protein
SMAD2	Intermediate protein	JUN	Intermediate protein
SMAD2	Intermediate protein	SMURF1	Intermediate protein
SMAD2	Intermediate protein	KAT2B	Intermediate protein
SMAD2	Intermediate protein	SP1	Intermediate protein
SMAD2	Intermediate protein	CDK2	Intermediate protein
SMAD2	Intermediate protein	SKIL	Intermediate protein
SMAD2	Intermediate protein	CHUK	Intermediate protein
SMAD2	Intermediate protein	STUB1	Intermediate protein
DAXX	Intermediate protein	DNMT3A	Intermediate protein
DAXX	Intermediate protein	PML	Seed protein
DAXX	Intermediate protein	RELA	Intermediate protein
DAXX	Intermediate protein	DAXX	Intermediate protein
DAXX	Intermediate protein	CDKN2A	Intermediate protein
DAXX	Intermediate protein	CSNK2A1	Intermediate protein
DAXX	Intermediate protein	ATRX	Intermediate protein
DAXX	Intermediate protein	STAT3	Intermediate protein
DAXX	Intermediate protein	STUB1	Intermediate protein
DAXX	Intermediate protein	TCF3	Seed protein
DAXX	Intermediate protein	DNMT1	Intermediate protein
DAXX	Intermediate protein	PIN1	Intermediate protein
DAXX	Intermediate protein	CEBPB	Intermediate protein
DAXX	Intermediate protein	HDAC3	Intermediate protein
DAXX	Intermediate protein	HDAC2	Intermediate protein
DAXX	Intermediate protein	CUL3	Intermediate protein
DAXX	Intermediate protein	SUMO1	Intermediate protein
PRKCD	Intermediate protein	SP1	Intermediate protein
PRKCD	Intermediate protein	MYC	Seed protein
PRKCD	Intermediate protein	STAT1	Intermediate protein
PRKCD	Intermediate protein	PRKCD	Intermediate protein
PRKCD	Intermediate protein	STAT3	Intermediate protein

PRKCD	Intermediate protein	KLF4	Seed protein
PRKCD	Intermediate protein	HDAC5	Intermediate protein
PRKCD	Intermediate protein	SPI1	Intermediate protein
PRKCD	Intermediate protein	PRKDC	Intermediate protein
PRKCD	Intermediate protein	VHL	Intermediate protein
PIAS1	Intermediate protein	JUN	Intermediate protein
PIAS1	Intermediate protein	DNMT3A	Intermediate protein
PIAS1	Intermediate protein	PML	Seed protein
PIAS1	Intermediate protein	NCOR1	Intermediate protein
PIAS1	Intermediate protein	PPARGC1A	Intermediate protein
PIAS1	Intermediate protein	STAT1	Intermediate protein
PIAS1	Intermediate protein	CHUK	Intermediate protein
PIAS1	Intermediate protein	CSNK2A1	Intermediate protein
PIAS1	Intermediate protein	SUMO1	Intermediate protein
PIAS2	Intermediate protein	SMAD3	Intermediate protein
PIAS2	Intermediate protein	JUN	Intermediate protein
PIAS2	Intermediate protein	CDKN2A	Intermediate protein
PIAS2	Intermediate protein	DNMT3A	Intermediate protein
PIAS2	Intermediate protein	ARNT	Intermediate protein
PIAS2	Intermediate protein	PIAS2	Intermediate protein
PIAS2	Intermediate protein	PML	Seed protein
PIAS2	Intermediate protein	MAPKAPK2	Intermediate protein
PIAS2	Intermediate protein	SUMO1	Intermediate protein
PIAS2	Intermediate protein	MYC	Seed protein
NANOG	Seed protein	ZNF281	Intermediate protein
NANOG	Seed protein	NANOG	Seed protein
TCF3	Seed protein	VDR	Intermediate protein
TCF3	Seed protein	DAXX	Intermediate protein
TCF3	Seed protein	PARP1	Intermediate protein
TCF3	Seed protein	KAT2B	Intermediate protein
TCF3	Seed protein	MAPKAPK2	Intermediate protein
TCF3	Seed protein	TCF3	Seed protein
TCF3	Seed protein	DNMT1	Intermediate protein
TCF3	Seed protein	CALM1	Intermediate protein
TCF3	Seed protein	RB1	Intermediate protein
TCF3	Seed protein	HDAC2	Intermediate protein
TCF3	Seed protein	GATA2	Intermediate protein

TCF3	Seed protein	MEN1	Intermediate protein
TCF3	Seed protein	SKP2	Intermediate protein
DNMT1	Intermediate protein	DNMT3A	Intermediate protein
DNMT1	Intermediate protein	SIRT1	Intermediate protein
DNMT1	Intermediate protein	SP1	Intermediate protein
DNMT1	Intermediate protein	KAT2B	Intermediate protein
DNMT1	Intermediate protein	RB1	Intermediate protein
DNMT1	Intermediate protein	SUV39H1	Intermediate protein
DNMT1	Intermediate protein	E2F1	Intermediate protein
DNMT1	Intermediate protein	DAXX	Intermediate protein
DNMT1	Intermediate protein	STAT3	Intermediate protein
DNMT1	Intermediate protein	TCF3	Seed protein
DNMT1	Intermediate protein	DNMT1	Intermediate protein
DNMT1	Intermediate protein	EZH2	Intermediate protein
DNMT1	Intermediate protein	HDAC2	Intermediate protein
DNMT1	Intermediate protein	UHRF1	Intermediate protein
DNMT1	Intermediate protein	SETD7	Intermediate protein
DNMT1	Intermediate protein	EHMT2	Intermediate protein
DNMT1	Intermediate protein	KAT5	Intermediate protein
DNMT1	Intermediate protein	TRIM28	Seed protein
PIN1	Intermediate protein	JUN	Intermediate protein
PIN1	Intermediate protein	PML	Seed protein
PIN1	Intermediate protein	SP1	Intermediate protein
PIN1	Intermediate protein	NCOR2	Intermediate protein
PIN1	Intermediate protein	CDK2	Intermediate protein
PIN1	Intermediate protein	BCL2	Intermediate protein
PIN1	Intermediate protein	MYC	Seed protein
PIN1	Intermediate protein	SMAD3	Intermediate protein
PIN1	Intermediate protein	SMAD2	Intermediate protein
PIN1	Intermediate protein	DAXX	Intermediate protein
PIN1	Intermediate protein	CSNK2A1	Intermediate protein
PIN1	Intermediate protein	HDAC3	Intermediate protein
EZH2	Intermediate protein	DNMT3A	Intermediate protein
EZH2	Intermediate protein	SIRT1	Intermediate protein
EZH2	Intermediate protein	PML	Seed protein
EZH2	Intermediate protein	KAT2B	Intermediate protein
EZH2	Intermediate protein	CDK2	Intermediate protein

EZH2	Intermediate protein	RELA	Intermediate protein
EZH2	Intermediate protein	ATRX	Intermediate protein
EZH2	Intermediate protein	STAT3	Intermediate protein
EZH2	Intermediate protein	DNMT1	Intermediate protein
EZH2	Intermediate protein	UHRF1	Intermediate protein
EZH2	Intermediate protein	CUL1	Intermediate protein
SPI1	Intermediate protein	TBP	Intermediate protein
SPI1	Intermediate protein	JUN	Intermediate protein
SPI1	Intermediate protein	PRKCD	Intermediate protein
SPI1	Intermediate protein	CSNK2A1	Intermediate protein
SPI1	Intermediate protein	DNMT3A	Intermediate protein
SPI1	Intermediate protein	FOS	Intermediate protein
SPI1	Intermediate protein	KLF4	Seed protein
SPI1	Intermediate protein	PML	Seed protein
SPI1	Intermediate protein	SKI	Intermediate protein
SPI1	Intermediate protein	CEBPA	Intermediate protein
SPI1	Intermediate protein	CEBPB	Intermediate protein
SPI1	Intermediate protein	RB1	Intermediate protein
SPI1	Intermediate protein	HDAC3	Intermediate protein
SPI1	Intermediate protein	GATA2	Intermediate protein
SPI1	Intermediate protein	SIN3A	Intermediate protein
WHSC1	Intermediate protein	WHSC1	Intermediate protein
WHSC1	Intermediate protein	HDAC2	Intermediate protein
WHSC1	Intermediate protein	SIN3A	Intermediate protein
WHSC1	Intermediate protein	TRIM28	Seed protein
CUL3	Intermediate protein	PML	Seed protein
CUL3	Intermediate protein	NFE2L2	Intermediate protein
CUL3	Intermediate protein	DAXX	Intermediate protein
CUL3	Intermediate protein	CUL3	Intermediate protein
CUL3	Intermediate protein	KAT5	Intermediate protein
CUL1	Intermediate protein	PML	Seed protein
CUL1	Intermediate protein	TRRAP	Intermediate protein
CUL1	Intermediate protein	CHEK2	Intermediate protein
CUL1	Intermediate protein	E2F1	Intermediate protein
CUL1	Intermediate protein	MYC	Seed protein
CUL1	Intermediate protein	SMAD3	Intermediate protein
CUL1	Intermediate protein	EZH2	Intermediate protein

CUL1	Intermediate protein	CUL1	Intermediate protein
CUL1	Intermediate protein	GATA2	Intermediate protein
CUL1	Intermediate protein	SKP1	Intermediate protein
CUL1	Intermediate protein	SKP2	Intermediate protein
CUL1	Intermediate protein	CDK2	Intermediate protein
CUL1	Intermediate protein	CHUK	Intermediate protein
CUL1	Intermediate protein	UHRF1	Intermediate protein
PRKDC	Intermediate protein	JUN	Intermediate protein
PRKDC	Intermediate protein	SP1	Intermediate protein
PRKDC	Intermediate protein	EGFR	Intermediate protein
PRKDC	Intermediate protein	MYC	Seed protein
PRKDC	Intermediate protein	CHEK2	Intermediate protein
PRKDC	Intermediate protein	PRKCD	Intermediate protein
PRKDC	Intermediate protein	CHUK	Intermediate protein
PRKDC	Intermediate protein	CSNK2A1	Intermediate protein
PRKDC	Intermediate protein	H2AFX	Intermediate protein
PRKDC	Intermediate protein	PARP1	Intermediate protein
PRKDC	Intermediate protein	KAT5	Intermediate protein
HIF1A	Intermediate protein	SMARCA4	Intermediate protein
HIF1A	Intermediate protein	RB1	Intermediate protein
HIF1A	Intermediate protein	RELA	Intermediate protein
HIF1A	Intermediate protein	MYC	Seed protein
HIF1A	Intermediate protein	SMAD3	Intermediate protein
HIF1A	Intermediate protein	HIF1A	Intermediate protein
HIF1A	Intermediate protein	HSP90AA1	Intermediate protein
HIF1A	Intermediate protein	JUN	Intermediate protein
HIF1A	Intermediate protein	SP1	Intermediate protein
HIF1A	Intermediate protein	KAT2B	Intermediate protein
HIF1A	Intermediate protein	BCL2	Intermediate protein
HIF1A	Intermediate protein	CDKN2A	Intermediate protein
HIF1A	Intermediate protein	CSNK2A1	Intermediate protein
HIF1A	Intermediate protein	STAT3	Intermediate protein
HIF1A	Intermediate protein	PARP1	Intermediate protein
HIF1A	Intermediate protein	ARNT	Intermediate protein
HIF1A	Intermediate protein	HDAC5	Intermediate protein
HIF1A	Intermediate protein	HDAC3	Intermediate protein
HIF1A	Intermediate protein	HDAC7	Intermediate protein

HIF1A	Intermediate protein	SUMO1	Intermediate protein
HIF1A	Intermediate protein	SIN3A	Intermediate protein
HIF1A	Intermediate protein	VHL	Intermediate protein
HIF1A	Intermediate protein	MAPK1	Intermediate protein
HIF1A	Intermediate protein	TRIM28	Seed protein
GATA2	Intermediate protein	HDAC5	Intermediate protein
GATA2	Intermediate protein	HDAC3	Intermediate protein
GATA2	Intermediate protein	SPI1	Intermediate protein
GATA2	Intermediate protein	JUN	Intermediate protein
GATA2	Intermediate protein	ZBTB16	Intermediate protein
GATA2	Intermediate protein	CUL1	Intermediate protein
GATA2	Intermediate protein	PML	Seed protein
GATA2	Intermediate protein	TCF3	Seed protein
GATA2	Intermediate protein	SKP1	Intermediate protein
SOX2	Seed protein	TBX2	Intermediate protein
SOX2	Seed protein	SETD7	Intermediate protein
SOX2	Seed protein	ZNF281	Intermediate protein
SOX2	Seed protein	POU5F1	Intermediate protein
SOX2	Seed protein	XPO1	Intermediate protein
SOX2	Seed protein	SUMO1	Intermediate protein
KAT5	Intermediate protein	PML	Seed protein
KAT5	Intermediate protein	RELA	Intermediate protein
KAT5	Intermediate protein	RB1	Intermediate protein
KAT5	Intermediate protein	TRRAP	Intermediate protein
KAT5	Intermediate protein	E2F1	Intermediate protein
KAT5	Intermediate protein	MYC	Seed protein
KAT5	Intermediate protein	DNMT1	Intermediate protein
KAT5	Intermediate protein	CUL3	Intermediate protein
KAT5	Intermediate protein	PRKDC	Intermediate protein
KAT5	Intermediate protein	KAT5	Intermediate protein
KAT5	Intermediate protein	SIRT1	Intermediate protein
KAT5	Intermediate protein	CDKN2A	Intermediate protein
KAT5	Intermediate protein	H2AFX	Intermediate protein
KAT5	Intermediate protein	STAT3	Intermediate protein
KAT5	Intermediate protein	KLF4	Seed protein
KAT5	Intermediate protein	HDAC3	Intermediate protein
KAT5	Intermediate protein	UHRF1	Intermediate protein

KAT5	Intermediate protein	HDAC7	Intermediate protein
KAT5	Intermediate protein	VHL	Intermediate protein
NELFE	Seed protein	NELFB	Intermediate protein
NELFB	Intermediate protein	JUN	Intermediate protein
NELFB	Intermediate protein	FOS	Intermediate protein
NELFB	Intermediate protein	NELFE	Seed protein
NELFB	Intermediate protein	NELFB	Intermediate protein
SKP1	Intermediate protein	HSP90AA1	Intermediate protein
SKP1	Intermediate protein	PML	Seed protein
SKP1	Intermediate protein	CDK2	Intermediate protein
SKP1	Intermediate protein	TRRAP	Intermediate protein
SKP1	Intermediate protein	MYC	Seed protein
SKP1	Intermediate protein	PPARGC1A	Intermediate protein
SKP1	Intermediate protein	CUL1	Intermediate protein
SKP1	Intermediate protein	GATA2	Intermediate protein
SKP1	Intermediate protein	SKP2	Intermediate protein
MEN1	Intermediate protein	SMAD3	Intermediate protein
MEN1	Intermediate protein	STUB1	Intermediate protein
MEN1	Intermediate protein	TCF3	Seed protein
MEN1	Intermediate protein	RELA	Intermediate protein
MEN1	Intermediate protein	HDAC2	Intermediate protein
MEN1	Intermediate protein	SIN3A	Intermediate protein
SKP2	Intermediate protein	SP1	Intermediate protein
SKP2	Intermediate protein	CDK2	Intermediate protein
SKP2	Intermediate protein	RB1	Intermediate protein
SKP2	Intermediate protein	FOXO1	Intermediate protein
SKP2	Intermediate protein	E2F1	Intermediate protein
SKP2	Intermediate protein	MYC	Seed protein
SKP2	Intermediate protein	STUB1	Intermediate protein
SKP2	Intermediate protein	PFDN5	Intermediate protein
SKP2	Intermediate protein	TCF3	Seed protein
SKP2	Intermediate protein	CUL1	Intermediate protein
SKP2	Intermediate protein	VHL	Intermediate protein
SKP2	Intermediate protein	SKP1	Intermediate protein
SKP2	Intermediate protein	SKP2	Intermediate protein
NCOA2	Intermediate protein	VDR	Intermediate protein
NCOA2	Intermediate protein	ARNT	Intermediate protein

NCOA2	Intermediate protein	FOS	Intermediate protein
NCOA2	Intermediate protein	PML	Seed protein
NCOA2	Intermediate protein	CCNT1	Intermediate protein
VDR	Intermediate protein	NCOA2	Intermediate protein
VDR	Intermediate protein	VDR	Intermediate protein
VDR	Intermediate protein	JUN	Intermediate protein
VDR	Intermediate protein	ZBTB16	Intermediate protein
VDR	Intermediate protein	SIRT1	Intermediate protein
VDR	Intermediate protein	NCOR1	Intermediate protein
VDR	Intermediate protein	NCOR2	Intermediate protein
VDR	Intermediate protein	PSMC5	Intermediate protein
VDR	Intermediate protein	RB1	Intermediate protein
VDR	Intermediate protein	MYC	Seed protein
VDR	Intermediate protein	SMAD3	Intermediate protein
VDR	Intermediate protein	STAT1	Intermediate protein
VDR	Intermediate protein	FOS	Intermediate protein
VDR	Intermediate protein	TCF3	Seed protein
VDR	Intermediate protein	CEBPA	Intermediate protein
VDR	Intermediate protein	TDG	Intermediate protein
JUN	Intermediate protein	PML	Seed protein
JUN	Intermediate protein	SKI	Intermediate protein
JUN	Intermediate protein	RB1	Intermediate protein
JUN	Intermediate protein	RELA	Intermediate protein
JUN	Intermediate protein	MYC	Seed protein
JUN	Intermediate protein	SMAD3	Intermediate protein
JUN	Intermediate protein	SMAD2	Intermediate protein
JUN	Intermediate protein	PIAS1	Intermediate protein
JUN	Intermediate protein	PIAS2	Intermediate protein
JUN	Intermediate protein	PIN1	Intermediate protein
JUN	Intermediate protein	SPI1	Intermediate protein
JUN	Intermediate protein	PRKDC	Intermediate protein
JUN	Intermediate protein	HIF1A	Intermediate protein
JUN	Intermediate protein	GATA2	Intermediate protein
JUN	Intermediate protein	NELFB	Intermediate protein
JUN	Intermediate protein	VDR	Intermediate protein
JUN	Intermediate protein	JUN	Intermediate protein
JUN	Intermediate protein	SIRT1	Intermediate protein

JUN	Intermediate protein	SP1	Intermediate protein
JUN	Intermediate protein	NCOR1	Intermediate protein
JUN	Intermediate protein	KAT2B	Intermediate protein
JUN	Intermediate protein	NCOR2	Intermediate protein
JUN	Intermediate protein	NFE2L2	Intermediate protein
JUN	Intermediate protein	TBP	Intermediate protein
JUN	Intermediate protein	STAT1	Intermediate protein
JUN	Intermediate protein	CSNK2A1	Intermediate protein
JUN	Intermediate protein	STAT3	Intermediate protein
JUN	Intermediate protein	PARP1	Intermediate protein
JUN	Intermediate protein	ARNT	Intermediate protein
JUN	Intermediate protein	FOS	Intermediate protein
JUN	Intermediate protein	HDAC3	Intermediate protein
JUN	Intermediate protein	SUMO1	Intermediate protein
JUN	Intermediate protein	MAPK3	Intermediate protein
JUN	Intermediate protein	MAPK1	Intermediate protein
HSP90AA1	Intermediate protein	CALM1	Intermediate protein
HSP90AA1	Intermediate protein	MYC	Seed protein
HSP90AA1	Intermediate protein	HIF1A	Intermediate protein
HSP90AA1	Intermediate protein	SKP1	Intermediate protein
HSP90AA1	Intermediate protein	SIRT1	Intermediate protein
HSP90AA1	Intermediate protein	CSNK2A1	Intermediate protein
HSP90AA1	Intermediate protein	STAT3	Intermediate protein
HSP90AA1	Intermediate protein	TUBA1A	Intermediate protein
HSP90AA1	Intermediate protein	EGFR	Intermediate protein
HSP90AA1	Intermediate protein	HSP90AA1	Intermediate protein
HSP90AA1	Intermediate protein	SP1	Intermediate protein
HSP90AA1	Intermediate protein	BCL2	Intermediate protein
HSP90AA1	Intermediate protein	CHUK	Intermediate protein
HSP90AA1	Intermediate protein	STUB1	Intermediate protein
HSP90AA1	Intermediate protein	SIN3A	Intermediate protein
HSP90AA1	Intermediate protein	VHL	Intermediate protein
SETDB1	Intermediate protein	DNMT3A	Intermediate protein
SETDB1	Intermediate protein	PML	Seed protein
SETDB1	Intermediate protein	ATRX	Intermediate protein
SETDB1	Intermediate protein	POU5F1	Intermediate protein
SETDB1	Intermediate protein	HDAC2	Intermediate protein

SETDB1	Intermediate protein	SIN3A	Intermediate protein
SETDB1	Intermediate protein	TRIM28	Seed protein
DNMT3A	Intermediate protein	DAXX	Intermediate protein
DNMT3A	Intermediate protein	SETDB1	Intermediate protein
DNMT3A	Intermediate protein	DNMT3A	Intermediate protein
DNMT3A	Intermediate protein	PIAS1	Intermediate protein
DNMT3A	Intermediate protein	PIAS2	Intermediate protein
DNMT3A	Intermediate protein	DNMT1	Intermediate protein
DNMT3A	Intermediate protein	EZH2	Intermediate protein
DNMT3A	Intermediate protein	RB1	Intermediate protein
DNMT3A	Intermediate protein	SPI1	Intermediate protein
DNMT3A	Intermediate protein	UHRF1	Intermediate protein
DNMT3A	Intermediate protein	SUV39H1	Intermediate protein
DNMT3A	Intermediate protein	EHMT2	Intermediate protein
DNMT3A	Intermediate protein	MYC	Seed protein
DNMT3A	Intermediate protein	TRIM28	Seed protein
SMURF1	Intermediate protein	SMURF1	Intermediate protein
SMURF1	Intermediate protein	PSMC5	Intermediate protein
SMURF1	Intermediate protein	SMAD2	Intermediate protein
SMURF1	Intermediate protein	STAT1	Intermediate protein
SMURF1	Intermediate protein	NFIC	Seed protein
SMURF1	Intermediate protein	XPO1	Intermediate protein
SIRT1	Intermediate protein	PML	Seed protein
SIRT1	Intermediate protein	SKI	Intermediate protein
SIRT1	Intermediate protein	RELA	Intermediate protein
SIRT1	Intermediate protein	RB1	Intermediate protein
SIRT1	Intermediate protein	FOXO1	Intermediate protein
SIRT1	Intermediate protein	E2F1	Intermediate protein
SIRT1	Intermediate protein	MYC	Seed protein
SIRT1	Intermediate protein	PPARGC1A	Intermediate protein
SIRT1	Intermediate protein	DNMT1	Intermediate protein
SIRT1	Intermediate protein	EZH2	Intermediate protein
SIRT1	Intermediate protein	KAT5	Intermediate protein
SIRT1	Intermediate protein	VDR	Intermediate protein
SIRT1	Intermediate protein	HSP90AA1	Intermediate protein
SIRT1	Intermediate protein	JUN	Intermediate protein
SIRT1	Intermediate protein	SIRT1	Intermediate protein

SIRT1	Intermediate protein	KAT2B	Intermediate protein
SIRT1	Intermediate protein	NCOR1	Intermediate protein
SIRT1	Intermediate protein	SUV39H1	Intermediate protein
SIRT1	Intermediate protein	CSNK2A1	Intermediate protein
SIRT1	Intermediate protein	STAT3	Intermediate protein
SIRT1	Intermediate protein	PARP1	Intermediate protein
SIRT1	Intermediate protein	FOS	Intermediate protein
SIRT1	Intermediate protein	SETD7	Intermediate protein
SIRT1	Intermediate protein	VHL	Intermediate protein
SIRT1	Intermediate protein	TRIM28	Seed protein
KAT2B	Intermediate protein	RB1	Intermediate protein
KAT2B	Intermediate protein	TRRAP	Intermediate protein
KAT2B	Intermediate protein	CCNT1	Intermediate protein
KAT2B	Intermediate protein	CHEK2	Intermediate protein
KAT2B	Intermediate protein	E2F1	Intermediate protein
KAT2B	Intermediate protein	MYC	Seed protein
KAT2B	Intermediate protein	SMAD3	Intermediate protein
KAT2B	Intermediate protein	SMAD2	Intermediate protein
KAT2B	Intermediate protein	TCF3	Seed protein
KAT2B	Intermediate protein	DNMT1	Intermediate protein
KAT2B	Intermediate protein	EZH2	Intermediate protein
KAT2B	Intermediate protein	HIF1A	Intermediate protein
KAT2B	Intermediate protein	JUN	Intermediate protein
KAT2B	Intermediate protein	SIRT1	Intermediate protein
KAT2B	Intermediate protein	KAT2B	Intermediate protein
KAT2B	Intermediate protein	CDK2	Intermediate protein
KAT2B	Intermediate protein	H2AFX	Intermediate protein
KAT2B	Intermediate protein	PARP1	Intermediate protein
KAT2B	Intermediate protein	CEBPB	Intermediate protein
KAT2B	Intermediate protein	HDAC3	Intermediate protein
KAT2B	Intermediate protein	EHMT2	Intermediate protein
KAT2B	Intermediate protein	VHL	Intermediate protein
SP1	Intermediate protein	SMARCA4	Intermediate protein
SP1	Intermediate protein	PML	Seed protein
SP1	Intermediate protein	PSMC5	Intermediate protein
SP1	Intermediate protein	RB1	Intermediate protein
SP1	Intermediate protein	RELA	Intermediate protein

SP1	Intermediate protein	RNF4	Intermediate protein
SP1	Intermediate protein	E2F1	Intermediate protein
SP1	Intermediate protein	MYC	Seed protein
SP1	Intermediate protein	SMAD3	Intermediate protein
SP1	Intermediate protein	SMAD2	Intermediate protein
SP1	Intermediate protein	PRKCD	Intermediate protein
SP1	Intermediate protein	DNMT1	Intermediate protein
SP1	Intermediate protein	PIN1	Intermediate protein
SP1	Intermediate protein	PRKDC	Intermediate protein
SP1	Intermediate protein	HIF1A	Intermediate protein
SP1	Intermediate protein	SKP2	Intermediate protein
SP1	Intermediate protein	HSP90AA1	Intermediate protein
SP1	Intermediate protein	JUN	Intermediate protein
SP1	Intermediate protein	SP1	Intermediate protein
SP1	Intermediate protein	NCOR1	Intermediate protein
SP1	Intermediate protein	NCOR2	Intermediate protein
SP1	Intermediate protein	CDK2	Intermediate protein
SP1	Intermediate protein	TBP	Intermediate protein
SP1	Intermediate protein	CDKN2A	Intermediate protein
SP1	Intermediate protein	STAT3	Intermediate protein
SP1	Intermediate protein	PARP1	Intermediate protein
SP1	Intermediate protein	ARNT	Intermediate protein
SP1	Intermediate protein	KLF4	Seed protein
SP1	Intermediate protein	RAD51	Intermediate protein
SP1	Intermediate protein	PFDN5	Intermediate protein
SP1	Intermediate protein	CEBPB	Intermediate protein
SP1	Intermediate protein	HDAC3	Intermediate protein
SP1	Intermediate protein	HDAC2	Intermediate protein
SP1	Intermediate protein	SIN3A	Intermediate protein
SP1	Intermediate protein	MAPK3	Intermediate protein
SP1	Intermediate protein	VHL	Intermediate protein
SP1	Intermediate protein	MAPK1	Intermediate protein
NCOR1	Intermediate protein	ZBTB16	Intermediate protein
NCOR1	Intermediate protein	PML	Seed protein
NCOR1	Intermediate protein	SKI	Intermediate protein
NCOR1	Intermediate protein	MYC	Seed protein
NCOR1	Intermediate protein	PIAS1	Intermediate protein

NCOR1	Intermediate protein	VDR	Intermediate protein
NCOR1	Intermediate protein	JUN	Intermediate protein
NCOR1	Intermediate protein	SIRT1	Intermediate protein
NCOR1	Intermediate protein	SP1	Intermediate protein
NCOR1	Intermediate protein	NCOR2	Intermediate protein
NCOR1	Intermediate protein	NFE2L2	Intermediate protein
NCOR1	Intermediate protein	SKIL	Intermediate protein
NCOR1	Intermediate protein	CHUK	Intermediate protein
NCOR1	Intermediate protein	CSNK2A1	Intermediate protein
NCOR1	Intermediate protein	HDAC5	Intermediate protein
NCOR1	Intermediate protein	CEBPB	Intermediate protein
NCOR1	Intermediate protein	HDAC3	Intermediate protein
NCOR1	Intermediate protein	HDAC7	Intermediate protein
NCOR1	Intermediate protein	SIN3A	Intermediate protein
NCOR2	Intermediate protein	VDR	Intermediate protein
NCOR2	Intermediate protein	JUN	Intermediate protein
NCOR2	Intermediate protein	ZBTB16	Intermediate protein
NCOR2	Intermediate protein	PML	Seed protein
NCOR2	Intermediate protein	NCOR1	Intermediate protein
NCOR2	Intermediate protein	SP1	Intermediate protein
NCOR2	Intermediate protein	NFE2L2	Intermediate protein
NCOR2	Intermediate protein	RELA	Intermediate protein
NCOR2	Intermediate protein	E2F4	Intermediate protein
NCOR2	Intermediate protein	SKIL	Intermediate protein
NCOR2	Intermediate protein	CHUK	Intermediate protein
NCOR2	Intermediate protein	ARNT	Intermediate protein
NCOR2	Intermediate protein	FOS	Intermediate protein
NCOR2	Intermediate protein	PIN1	Intermediate protein
NCOR2	Intermediate protein	HDAC5	Intermediate protein
NCOR2	Intermediate protein	CEBPB	Intermediate protein
NCOR2	Intermediate protein	HDAC3	Intermediate protein
NCOR2	Intermediate protein	HDAC2	Intermediate protein
NCOR2	Intermediate protein	HDAC7	Intermediate protein
NCOR2	Intermediate protein	SUMO1	Intermediate protein
NCOR2	Intermediate protein	SIN3A	Intermediate protein
CDK2	Intermediate protein	PML	Seed protein
CDK2	Intermediate protein	RB1	Intermediate protein

CDK2	Intermediate protein	CCNH	Intermediate protein
CDK2	Intermediate protein	E2F1	Intermediate protein
CDK2	Intermediate protein	MYC	Seed protein
CDK2	Intermediate protein	SMAD3	Intermediate protein
CDK2	Intermediate protein	SMAD2	Intermediate protein
CDK2	Intermediate protein	EZH2	Intermediate protein
CDK2	Intermediate protein	PIN1	Intermediate protein
CDK2	Intermediate protein	CUL1	Intermediate protein
CDK2	Intermediate protein	SKP1	Intermediate protein
CDK2	Intermediate protein	SKP2	Intermediate protein
CDK2	Intermediate protein	SP1	Intermediate protein
CDK2	Intermediate protein	KAT2B	Intermediate protein
CDK2	Intermediate protein	CDK2	Intermediate protein
CDK2	Intermediate protein	CEBPA	Intermediate protein
BCL2	Intermediate protein	PML	Seed protein
BCL2	Intermediate protein	RELA	Intermediate protein
BCL2	Intermediate protein	MYC	Seed protein
BCL2	Intermediate protein	PIN1	Intermediate protein
BCL2	Intermediate protein	HIF1A	Intermediate protein
BCL2	Intermediate protein	HSP90AA1	Intermediate protein
BCL2	Intermediate protein	BCL2	Intermediate protein
BCL2	Intermediate protein	CHUK	Intermediate protein
BCL2	Intermediate protein	PARP1	Intermediate protein
BCL2	Intermediate protein	CEBPA	Intermediate protein
BCL2	Intermediate protein	MAPK1	Intermediate protein
NFE2L2	Intermediate protein	JUN	Intermediate protein
NFE2L2	Intermediate protein	SMARCA4	Intermediate protein
NFE2L2	Intermediate protein	NCOR1	Intermediate protein
NFE2L2	Intermediate protein	NCOR2	Intermediate protein
NFE2L2	Intermediate protein	HDAC3	Intermediate protein
NFE2L2	Intermediate protein	HDAC2	Intermediate protein
NFE2L2	Intermediate protein	CUL3	Intermediate protein
NFE2L2	Intermediate protein	RNF4	Intermediate protein
NFE2L2	Intermediate protein	SUMO1	Intermediate protein
NFE2L2	Intermediate protein	MYC	Seed protein
NFE2L2	Intermediate protein	TRIM28	Seed protein
SUV39H1	Intermediate protein	DNMT3A	Intermediate protein

SUV39H1	Intermediate protein	SIRT1	Intermediate protein
SUV39H1	Intermediate protein	PML	Seed protein
SUV39H1	Intermediate protein	RB1	Intermediate protein
SUV39H1	Intermediate protein	SUV39H1	Intermediate protein
SUV39H1	Intermediate protein	SMAD3	Intermediate protein
SUV39H1	Intermediate protein	DNMT1	Intermediate protein
SUV39H1	Intermediate protein	HDAC5	Intermediate protein
SUV39H1	Intermediate protein	HDAC3	Intermediate protein
SUV39H1	Intermediate protein	HDAC2	Intermediate protein
SUV39H1	Intermediate protein	UHRF1	Intermediate protein
NR2C1	Intermediate protein	HDAC3	Intermediate protein
NR2C1	Intermediate protein	PML	Seed protein
SKIL	Intermediate protein	SMAD3	Intermediate protein
SKIL	Intermediate protein	RB1	Intermediate protein
SKIL	Intermediate protein	SMAD2	Intermediate protein
SKIL	Intermediate protein	PML	Seed protein
SKIL	Intermediate protein	SKI	Intermediate protein
SKIL	Intermediate protein	NCOR1	Intermediate protein
SKIL	Intermediate protein	NCOR2	Intermediate protein
SKIL	Intermediate protein	SIN3A	Intermediate protein
SKIL	Intermediate protein	SKIL	Intermediate protein
TBX2	Intermediate protein	SOX2	Seed protein
TBX2	Intermediate protein	PML	Seed protein
ATAD2	Intermediate protein	MYC	Seed protein
ATAD2	Intermediate protein	E2F1	Intermediate protein
ATAD2	Intermediate protein	E2F4	Intermediate protein
TBP	Intermediate protein	RB1	Intermediate protein
TBP	Intermediate protein	RELA	Intermediate protein
TBP	Intermediate protein	RNF4	Intermediate protein
TBP	Intermediate protein	E2F1	Intermediate protein
TBP	Intermediate protein	MYC	Seed protein
TBP	Intermediate protein	SPI1	Intermediate protein
TBP	Intermediate protein	JUN	Intermediate protein
TBP	Intermediate protein	SP1	Intermediate protein
TBP	Intermediate protein	TBP	Intermediate protein
TBP	Intermediate protein	FOS	Intermediate protein
TBP	Intermediate protein	SETD7	Intermediate protein

CDKN2A	Intermediate protein	SMARCA4	Intermediate protein
CDKN2A	Intermediate protein	PML	Seed protein
CDKN2A	Intermediate protein	SP1	Intermediate protein
CDKN2A	Intermediate protein	MYC	Seed protein
CDKN2A	Intermediate protein	E2F1	Intermediate protein
CDKN2A	Intermediate protein	DAXX	Intermediate protein
CDKN2A	Intermediate protein	PIAS2	Intermediate protein
CDKN2A	Intermediate protein	HIF1A	Intermediate protein
CDKN2A	Intermediate protein	KAT5	Intermediate protein
CDKN2A	Intermediate protein	VHL	Intermediate protein
CDKN2A	Intermediate protein	TRIM28	Seed protein
STAT1	Intermediate protein	VDR	Intermediate protein
STAT1	Intermediate protein	JUN	Intermediate protein
STAT1	Intermediate protein	SMURF1	Intermediate protein
STAT1	Intermediate protein	SMARCA4	Intermediate protein
STAT1	Intermediate protein	PML	Seed protein
STAT1	Intermediate protein	EGFR	Intermediate protein
STAT1	Intermediate protein	PRKCD	Intermediate protein
STAT1	Intermediate protein	STAT1	Intermediate protein
STAT1	Intermediate protein	STAT3	Intermediate protein
STAT1	Intermediate protein	FOS	Intermediate protein
STAT1	Intermediate protein	PIAS1	Intermediate protein
STAT1	Intermediate protein	HDAC3	Intermediate protein
STAT1	Intermediate protein	TRIM28	Seed protein
CSNK2A1	Intermediate protein	PML	Seed protein
CSNK2A1	Intermediate protein	CALM1	Intermediate protein
CSNK2A1	Intermediate protein	RELA	Intermediate protein
CSNK2A1	Intermediate protein	MYC	Seed protein
CSNK2A1	Intermediate protein	DAXX	Intermediate protein
CSNK2A1	Intermediate protein	PIAS1	Intermediate protein
CSNK2A1	Intermediate protein	PIN1	Intermediate protein
CSNK2A1	Intermediate protein	SPI1	Intermediate protein
CSNK2A1	Intermediate protein	PRKDC	Intermediate protein
CSNK2A1	Intermediate protein	HIF1A	Intermediate protein
CSNK2A1	Intermediate protein	HSP90AA1	Intermediate protein
CSNK2A1	Intermediate protein	JUN	Intermediate protein
CSNK2A1	Intermediate protein	SIRT1	Intermediate protein

CSNK2A1	Intermediate protein	NCOR1	Intermediate protein
CSNK2A1	Intermediate protein	CSNK2A1	Intermediate protein
CSNK2A1	Intermediate protein	CHUK	Intermediate protein
CSNK2A1	Intermediate protein	H2AFX	Intermediate protein
CSNK2A1	Intermediate protein	FOS	Intermediate protein
CSNK2A1	Intermediate protein	HDAC3	Intermediate protein
CSNK2A1	Intermediate protein	HDAC2	Intermediate protein
CSNK2A1	Intermediate protein	VHL	Intermediate protein
CHUK	Intermediate protein	RELA	Intermediate protein
CHUK	Intermediate protein	MYC	Seed protein
CHUK	Intermediate protein	SMAD3	Intermediate protein
CHUK	Intermediate protein	SMAD2	Intermediate protein
CHUK	Intermediate protein	PIAS1	Intermediate protein
CHUK	Intermediate protein	CUL1	Intermediate protein
CHUK	Intermediate protein	PRKDC	Intermediate protein
CHUK	Intermediate protein	HSP90AA1	Intermediate protein
CHUK	Intermediate protein	NCOR1	Intermediate protein
CHUK	Intermediate protein	NCOR2	Intermediate protein
CHUK	Intermediate protein	BCL2	Intermediate protein
CHUK	Intermediate protein	CHUK	Intermediate protein
CHUK	Intermediate protein	CSNK2A1	Intermediate protein
CHUK	Intermediate protein	STUB1	Intermediate protein
CHUK	Intermediate protein	IKBKE	Intermediate protein
ATRX	Intermediate protein	DAXX	Intermediate protein
ATRX	Intermediate protein	SETDB1	Intermediate protein
ATRX	Intermediate protein	PML	Seed protein
ATRX	Intermediate protein	RAD51	Intermediate protein
ATRX	Intermediate protein	TRIM28	Seed protein
ATRX	Intermediate protein	EZH2	Intermediate protein
H2AFX	Intermediate protein	SMARCA4	Intermediate protein
H2AFX	Intermediate protein	PML	Seed protein
H2AFX	Intermediate protein	KAT2B	Intermediate protein
H2AFX	Intermediate protein	EGFR	Intermediate protein
H2AFX	Intermediate protein	CSNK2A1	Intermediate protein
H2AFX	Intermediate protein	PARP1	Intermediate protein
H2AFX	Intermediate protein	RAD51	Intermediate protein
H2AFX	Intermediate protein	PRKDC	Intermediate protein

H2AFX	Intermediate protein	KAT5	Intermediate protein
H2AFX	Intermediate protein	TRIM28	Seed protein
STAT3	Intermediate protein	SMARCA4	Intermediate protein
STAT3	Intermediate protein	PML	Seed protein
STAT3	Intermediate protein	RB1	Intermediate protein
STAT3	Intermediate protein	RELA	Intermediate protein
STAT3	Intermediate protein	EGFR	Intermediate protein
STAT3	Intermediate protein	DAXX	Intermediate protein
STAT3	Intermediate protein	PRKCD	Intermediate protein
STAT3	Intermediate protein	DNMT1	Intermediate protein
STAT3	Intermediate protein	EZH2	Intermediate protein
STAT3	Intermediate protein	HIF1A	Intermediate protein
STAT3	Intermediate protein	KAT5	Intermediate protein
STAT3	Intermediate protein	HSP90AA1	Intermediate protein
STAT3	Intermediate protein	JUN	Intermediate protein
STAT3	Intermediate protein	SIRT1	Intermediate protein
STAT3	Intermediate protein	SP1	Intermediate protein
STAT3	Intermediate protein	STAT1	Intermediate protein
STAT3	Intermediate protein	STAT3	Intermediate protein
STAT3	Intermediate protein	CEBPB	Intermediate protein
STAT3	Intermediate protein	HDAC3	Intermediate protein
STAT3	Intermediate protein	HDAC2	Intermediate protein
STAT3	Intermediate protein	SETD7	Intermediate protein
STAT3	Intermediate protein	SIN3A	Intermediate protein
STAT3	Intermediate protein	MAPK1	Intermediate protein
STAT3	Intermediate protein	TRIM28	Seed protein
PARP1	Intermediate protein	ZBTB16	Intermediate protein
PARP1	Intermediate protein	RELA	Intermediate protein
PARP1	Intermediate protein	FOXO1	Intermediate protein
PARP1	Intermediate protein	E2F1	Intermediate protein
PARP1	Intermediate protein	TCF3	Seed protein
PARP1	Intermediate protein	PRKDC	Intermediate protein
PARP1	Intermediate protein	HIF1A	Intermediate protein
PARP1	Intermediate protein	JUN	Intermediate protein
PARP1	Intermediate protein	SIRT1	Intermediate protein
PARP1	Intermediate protein	KAT2B	Intermediate protein
PARP1	Intermediate protein	SP1	Intermediate protein

PARP1	Intermediate protein	BCL2	Intermediate protein
PARP1	Intermediate protein	H2AFX	Intermediate protein
PARP1	Intermediate protein	PARP1	Intermediate protein
PARP1	Intermediate protein	NFIC	Seed protein
PARP1	Intermediate protein	CEBPA	Intermediate protein
PARP1	Intermediate protein	HDAC2	Intermediate protein
PARP1	Intermediate protein	UHRF1	Intermediate protein
PARP1	Intermediate protein	TRIM28	Seed protein
FOS	Intermediate protein	NCOA2	Intermediate protein
FOS	Intermediate protein	VDR	Intermediate protein
FOS	Intermediate protein	JUN	Intermediate protein
FOS	Intermediate protein	SIRT1	Intermediate protein
FOS	Intermediate protein	PML	Seed protein
FOS	Intermediate protein	NCOR2	Intermediate protein
FOS	Intermediate protein	RB1	Intermediate protein
FOS	Intermediate protein	RELA	Intermediate protein
FOS	Intermediate protein	SMAD3	Intermediate protein
FOS	Intermediate protein	TBP	Intermediate protein
FOS	Intermediate protein	STAT1	Intermediate protein
FOS	Intermediate protein	CSNK2A1	Intermediate protein
FOS	Intermediate protein	SPI1	Intermediate protein
FOS	Intermediate protein	MAPK1	Intermediate protein
FOS	Intermediate protein	NELFB	Intermediate protein
ARNT	Intermediate protein	NCOA2	Intermediate protein
ARNT	Intermediate protein	JUN	Intermediate protein
ARNT	Intermediate protein	ARNT	Intermediate protein
ARNT	Intermediate protein	PIAS2	Intermediate protein
ARNT	Intermediate protein	PML	Seed protein
ARNT	Intermediate protein	SP1	Intermediate protein
ARNT	Intermediate protein	NCOR2	Intermediate protein
ARNT	Intermediate protein	RB1	Intermediate protein
ARNT	Intermediate protein	HIF1A	Intermediate protein
ARNT	Intermediate protein	VHL	Intermediate protein
KLF4	Seed protein	HDAC5	Intermediate protein
KLF4	Seed protein	RELA	Intermediate protein
KLF4	Seed protein	SPI1	Intermediate protein
KLF4	Seed protein	PRKCD	Intermediate protein

KLF4	Seed protein	HDAC2	Intermediate protein
KLF4	Seed protein	SETD7	Intermediate protein
KLF4	Seed protein	HDAC7	Intermediate protein
KLF4	Seed protein	SP1	Intermediate protein
KLF4	Seed protein	KAT5	Intermediate protein
KLF4	Seed protein	VHL	Intermediate protein
POU5F1	Intermediate protein	SETDB1	Intermediate protein
POU5F1	Intermediate protein	ZNF281	Intermediate protein
POU5F1	Intermediate protein	POU5F1	Intermediate protein
POU5F1	Intermediate protein	SOX2	Seed protein
POU5F1	Intermediate protein	TRIM28	Seed protein
RAD51	Intermediate protein	ATRX	Intermediate protein
RAD51	Intermediate protein	H2AFX	Intermediate protein
RAD51	Intermediate protein	MSH2	Intermediate protein
RAD51	Intermediate protein	PML	Seed protein
RAD51	Intermediate protein	RAD51	Intermediate protein
RAD51	Intermediate protein	SP1	Intermediate protein
RAD51	Intermediate protein	SUMO1	Intermediate protein
NFIC	Seed protein	SMAD3	Intermediate protein
NFIC	Seed protein	SMURF1	Intermediate protein
NFIC	Seed protein	PARP1	Intermediate protein
TTC5	Intermediate protein	MYC	Seed protein
STUB1	Intermediate protein	RELA	Intermediate protein
STUB1	Intermediate protein	EGFR	Intermediate protein
STUB1	Intermediate protein	FOXO1	Intermediate protein
STUB1	Intermediate protein	MYC	Seed protein
STUB1	Intermediate protein	SMAD3	Intermediate protein
STUB1	Intermediate protein	SMAD2	Intermediate protein
STUB1	Intermediate protein	DAXX	Intermediate protein
STUB1	Intermediate protein	MEN1	Intermediate protein
STUB1	Intermediate protein	SKP2	Intermediate protein
STUB1	Intermediate protein	HSP90AA1	Intermediate protein
STUB1	Intermediate protein	CHUK	Intermediate protein
STUB1	Intermediate protein	STUB1	Intermediate protein
PFDN5	Intermediate protein	SP1	Intermediate protein
PFDN5	Intermediate protein	SIN3A	Intermediate protein
PFDN5	Intermediate protein	MYC	Seed protein

PFDN5	Intermediate protein	TRIM28	Seed protein
PFDN5	Intermediate protein	SKP2	Intermediate protein
CEBPA	Intermediate protein	SMAD3	Intermediate protein
CEBPA	Intermediate protein	VDR	Intermediate protein
CEBPA	Intermediate protein	PARP1	Intermediate protein
CEBPA	Intermediate protein	ZBTB16	Intermediate protein
CEBPA	Intermediate protein	SMARCA4	Intermediate protein
CEBPA	Intermediate protein	CDK2	Intermediate protein
CEBPA	Intermediate protein	BCL2	Intermediate protein
CEBPA	Intermediate protein	CEBPA	Intermediate protein
CEBPA	Intermediate protein	CEBPB	Intermediate protein
CEBPA	Intermediate protein	SPI1	Intermediate protein
CEBPA	Intermediate protein	HDAC2	Intermediate protein
CEBPA	Intermediate protein	UBE3A	Intermediate protein
CEBPA	Intermediate protein	MYC	Seed protein
HDAC5	Intermediate protein	ZBTB16	Intermediate protein
HDAC5	Intermediate protein	NCOR1	Intermediate protein
HDAC5	Intermediate protein	NCOR2	Intermediate protein
HDAC5	Intermediate protein	SUV39H1	Intermediate protein
HDAC5	Intermediate protein	SMAD3	Intermediate protein
HDAC5	Intermediate protein	PRKCD	Intermediate protein
HDAC5	Intermediate protein	KLF4	Seed protein
HDAC5	Intermediate protein	HDAC5	Intermediate protein
HDAC5	Intermediate protein	HDAC3	Intermediate protein
HDAC5	Intermediate protein	HIF1A	Intermediate protein
HDAC5	Intermediate protein	GATA2	Intermediate protein
HDAC5	Intermediate protein	HDAC7	Intermediate protein
HDAC5	Intermediate protein	SIN3A	Intermediate protein
CEBPB	Intermediate protein	SMARCA4	Intermediate protein
CEBPB	Intermediate protein	SP1	Intermediate protein
CEBPB	Intermediate protein	KAT2B	Intermediate protein
CEBPB	Intermediate protein	NCOR1	Intermediate protein
CEBPB	Intermediate protein	NCOR2	Intermediate protein
CEBPB	Intermediate protein	RELA	Intermediate protein
CEBPB	Intermediate protein	RB1	Intermediate protein
CEBPB	Intermediate protein	EGFR	Intermediate protein
CEBPB	Intermediate protein	FOXO1	Intermediate protein

CEBPB	Intermediate protein	MYC	Seed protein
CEBPB	Intermediate protein	SMAD3	Intermediate protein
CEBPB	Intermediate protein	DAXX	Intermediate protein
CEBPB	Intermediate protein	STAT3	Intermediate protein
CEBPB	Intermediate protein	CEBPA	Intermediate protein
CEBPB	Intermediate protein	CEBPB	Intermediate protein
CEBPB	Intermediate protein	SPI1	Intermediate protein
CEBPB	Intermediate protein	EHMT2	Intermediate protein
CEBPB	Intermediate protein	MAPK1	Intermediate protein
CEBPB	Intermediate protein	TRIM28	Seed protein
HDAC3	Intermediate protein	ZBTB16	Intermediate protein
HDAC3	Intermediate protein	SMARCA4	Intermediate protein
HDAC3	Intermediate protein	PML	Seed protein
HDAC3	Intermediate protein	SKI	Intermediate protein
HDAC3	Intermediate protein	RELA	Intermediate protein
HDAC3	Intermediate protein	RB1	Intermediate protein
HDAC3	Intermediate protein	MYC	Seed protein
HDAC3	Intermediate protein	E2F4	Intermediate protein
HDAC3	Intermediate protein	DAXX	Intermediate protein
HDAC3	Intermediate protein	PIN1	Intermediate protein
HDAC3	Intermediate protein	SPI1	Intermediate protein
HDAC3	Intermediate protein	HIF1A	Intermediate protein
HDAC3	Intermediate protein	GATA2	Intermediate protein
HDAC3	Intermediate protein	KAT5	Intermediate protein
HDAC3	Intermediate protein	JUN	Intermediate protein
HDAC3	Intermediate protein	NCOR1	Intermediate protein
HDAC3	Intermediate protein	SP1	Intermediate protein
HDAC3	Intermediate protein	KAT2B	Intermediate protein
HDAC3	Intermediate protein	NCOR2	Intermediate protein
HDAC3	Intermediate protein	NFE2L2	Intermediate protein
HDAC3	Intermediate protein	SUV39H1	Intermediate protein
HDAC3	Intermediate protein	NR2C1	Intermediate protein
HDAC3	Intermediate protein	STAT1	Intermediate protein
HDAC3	Intermediate protein	CSNK2A1	Intermediate protein
HDAC3	Intermediate protein	STAT3	Intermediate protein
HDAC3	Intermediate protein	HDAC5	Intermediate protein
HDAC3	Intermediate protein	HDAC3	Intermediate protein

HDAC3	Intermediate protein	HDAC7	Intermediate protein
HDAC3	Intermediate protein	XPO1	Intermediate protein
HDAC3	Intermediate protein	TRIM28	Seed protein
HDAC2	Intermediate protein	ZBTB16	Intermediate protein
HDAC2	Intermediate protein	SMARCA4	Intermediate protein
HDAC2	Intermediate protein	PML	Seed protein
HDAC2	Intermediate protein	RELA	Intermediate protein
HDAC2	Intermediate protein	RB1	Intermediate protein
HDAC2	Intermediate protein	MYC	Seed protein
HDAC2	Intermediate protein	DAXX	Intermediate protein
HDAC2	Intermediate protein	TCF3	Seed protein
HDAC2	Intermediate protein	DNMT1	Intermediate protein
HDAC2	Intermediate protein	WHSC1	Intermediate protein
HDAC2	Intermediate protein	MEN1	Intermediate protein
HDAC2	Intermediate protein	SETDB1	Intermediate protein
HDAC2	Intermediate protein	SP1	Intermediate protein
HDAC2	Intermediate protein	NCOR2	Intermediate protein
HDAC2	Intermediate protein	NFE2L2	Intermediate protein
HDAC2	Intermediate protein	SUV39H1	Intermediate protein
HDAC2	Intermediate protein	CSNK2A1	Intermediate protein
HDAC2	Intermediate protein	STAT3	Intermediate protein
HDAC2	Intermediate protein	PARP1	Intermediate protein
HDAC2	Intermediate protein	KLF4	Seed protein
HDAC2	Intermediate protein	CEBPA	Intermediate protein
HDAC2	Intermediate protein	HDAC7	Intermediate protein
HDAC2	Intermediate protein	SIN3A	Intermediate protein
HDAC2	Intermediate protein	VHL	Intermediate protein
SETD7	Intermediate protein	TBP	Intermediate protein
SETD7	Intermediate protein	STAT3	Intermediate protein
SETD7	Intermediate protein	SIRT1	Intermediate protein
SETD7	Intermediate protein	KLF4	Seed protein
SETD7	Intermediate protein	SMARCA4	Intermediate protein
SETD7	Intermediate protein	DNMT1	Intermediate protein
SETD7	Intermediate protein	RELA	Intermediate protein
SETD7	Intermediate protein	RB1	Intermediate protein
SETD7	Intermediate protein	SETD7	Intermediate protein
SETD7	Intermediate protein	SOX2	Seed protein

SETD7	Intermediate protein	E2F1	Intermediate protein
UHRF1	Intermediate protein	DNMT3A	Intermediate protein
UHRF1	Intermediate protein	PARP1	Intermediate protein
UHRF1	Intermediate protein	PML	Seed protein
UHRF1	Intermediate protein	DNMT1	Intermediate protein
UHRF1	Intermediate protein	EZH2	Intermediate protein
UHRF1	Intermediate protein	RB1	Intermediate protein
UHRF1	Intermediate protein	UHRF1	Intermediate protein
UHRF1	Intermediate protein	SUV39H1	Intermediate protein
UHRF1	Intermediate protein	EHMT2	Intermediate protein
UHRF1	Intermediate protein	CUL1	Intermediate protein
UHRF1	Intermediate protein	RNF4	Intermediate protein
UHRF1	Intermediate protein	KAT5	Intermediate protein
UHRF1	Intermediate protein	TRIM28	Seed protein
EHMT2	Intermediate protein	DNMT3A	Intermediate protein
EHMT2	Intermediate protein	PML	Seed protein
EHMT2	Intermediate protein	KAT2B	Intermediate protein
EHMT2	Intermediate protein	DNMT1	Intermediate protein
EHMT2	Intermediate protein	CEBPB	Intermediate protein
EHMT2	Intermediate protein	UHRF1	Intermediate protein
EHMT2	Intermediate protein	EHMT2	Intermediate protein
TUBA1A	Intermediate protein	HSP90AA1	Intermediate protein
TUBA1A	Intermediate protein	MYC	Seed protein
TUBA1A	Intermediate protein	VHL	Intermediate protein
HDAC7	Intermediate protein	ZBTB16	Intermediate protein
HDAC7	Intermediate protein	KLF4	Seed protein
HDAC7	Intermediate protein	PML	Seed protein
HDAC7	Intermediate protein	NCOR1	Intermediate protein
HDAC7	Intermediate protein	NCOR2	Intermediate protein
HDAC7	Intermediate protein	HDAC5	Intermediate protein
HDAC7	Intermediate protein	HDAC3	Intermediate protein
HDAC7	Intermediate protein	HDAC2	Intermediate protein
HDAC7	Intermediate protein	HIF1A	Intermediate protein
HDAC7	Intermediate protein	HDAC7	Intermediate protein
HDAC7	Intermediate protein	KAT5	Intermediate protein
XPO1	Intermediate protein	SMURF1	Intermediate protein
XPO1	Intermediate protein	E2F4	Intermediate protein

XPO1	Intermediate protein	HDAC3	Intermediate protein
XPO1	Intermediate protein	SOX2	Seed protein
SUMO1	Intermediate protein	ZBTB16	Intermediate protein
SUMO1	Intermediate protein	PML	Seed protein
SUMO1	Intermediate protein	RNF4	Intermediate protein
SUMO1	Intermediate protein	DAXX	Intermediate protein
SUMO1	Intermediate protein	PIAS1	Intermediate protein
SUMO1	Intermediate protein	PIAS2	Intermediate protein
SUMO1	Intermediate protein	HIF1A	Intermediate protein
SUMO1	Intermediate protein	SOX2	Seed protein
SUMO1	Intermediate protein	JUN	Intermediate protein
SUMO1	Intermediate protein	NCOR2	Intermediate protein
SUMO1	Intermediate protein	NFE2L2	Intermediate protein
SUMO1	Intermediate protein	RAD51	Intermediate protein
SUMO1	Intermediate protein	SUMO1	Intermediate protein
SUMO1	Intermediate protein	SIN3A	Intermediate protein
SUMO1	Intermediate protein	TDG	Intermediate protein
SUMO1	Intermediate protein	IKBKE	Intermediate protein
SIN3A	Intermediate protein	ZBTB16	Intermediate protein
SIN3A	Intermediate protein	SMARCA4	Intermediate protein
SIN3A	Intermediate protein	PML	Seed protein
SIN3A	Intermediate protein	SKI	Intermediate protein
SIN3A	Intermediate protein	E2F4	Intermediate protein
SIN3A	Intermediate protein	SMAD3	Intermediate protein
SIN3A	Intermediate protein	SPI1	Intermediate protein
SIN3A	Intermediate protein	WHSC1	Intermediate protein
SIN3A	Intermediate protein	HIF1A	Intermediate protein
SIN3A	Intermediate protein	MEN1	Intermediate protein
SIN3A	Intermediate protein	HSP90AA1	Intermediate protein
SIN3A	Intermediate protein	SETDB1	Intermediate protein
SIN3A	Intermediate protein	NCOR1	Intermediate protein
SIN3A	Intermediate protein	SP1	Intermediate protein
SIN3A	Intermediate protein	NCOR2	Intermediate protein
SIN3A	Intermediate protein	SKIL	Intermediate protein
SIN3A	Intermediate protein	STAT3	Intermediate protein
SIN3A	Intermediate protein	PFDN5	Intermediate protein
SIN3A	Intermediate protein	HDAC5	Intermediate protein

SIN3A	Intermediate protein	HDAC2	Intermediate protein
SIN3A	Intermediate protein	SUMO1	Intermediate protein
TDG	Intermediate protein	VDR	Intermediate protein
TDG	Intermediate protein	PML	Seed protein
TDG	Intermediate protein	RNF4	Intermediate protein
TDG	Intermediate protein	SUMO1	Intermediate protein
VHL	Intermediate protein	RPL11	Intermediate protein
VHL	Intermediate protein	CHEK2	Intermediate protein
VHL	Intermediate protein	E2F1	Intermediate protein
VHL	Intermediate protein	MYC	Seed protein
VHL	Intermediate protein	PRKCD	Intermediate protein
VHL	Intermediate protein	HIF1A	Intermediate protein
VHL	Intermediate protein	KAT5	Intermediate protein
VHL	Intermediate protein	SKP2	Intermediate protein
VHL	Intermediate protein	HSP90AA1	Intermediate protein
VHL	Intermediate protein	SIRT1	Intermediate protein
VHL	Intermediate protein	KAT2B	Intermediate protein
VHL	Intermediate protein	SP1	Intermediate protein
VHL	Intermediate protein	CDKN2A	Intermediate protein
VHL	Intermediate protein	CSNK2A1	Intermediate protein
VHL	Intermediate protein	ARNT	Intermediate protein
VHL	Intermediate protein	KLF4	Seed protein
VHL	Intermediate protein	HDAC2	Intermediate protein
VHL	Intermediate protein	TUBA1A	Intermediate protein
VHL	Intermediate protein	VHL	Intermediate protein
VHL	Intermediate protein	TRIM28	Seed protein
MAPK3	Intermediate protein	JUN	Intermediate protein
MAPK3	Intermediate protein	PML	Seed protein
MAPK3	Intermediate protein	SP1	Intermediate protein
MAPK3	Intermediate protein	MAPKAPK2	Intermediate protein
MAPK3	Intermediate protein	MYC	Seed protein
MAPK1	Intermediate protein	PML	Seed protein
MAPK1	Intermediate protein	MAPKAPK2	Intermediate protein
MAPK1	Intermediate protein	MYC	Seed protein
MAPK1	Intermediate protein	HIF1A	Intermediate protein
MAPK1	Intermediate protein	JUN	Intermediate protein
MAPK1	Intermediate protein	SP1	Intermediate protein

MAPK1	Intermediate protein	BCL2	Intermediate protein
MAPK1	Intermediate protein	STAT3	Intermediate protein
MAPK1	Intermediate protein	FOS	Intermediate protein
MAPK1	Intermediate protein	CEBPB	Intermediate protein
MAPK1	Intermediate protein	MAPK1	Intermediate protein
TRIM28	Seed protein	SETDB1	Intermediate protein
TRIM28	Seed protein	DNMT3A	Intermediate protein
TRIM28	Seed protein	SIRT1	Intermediate protein
TRIM28	Seed protein	SMARCA4	Intermediate protein
TRIM28	Seed protein	MAPKAPK2	Intermediate protein
TRIM28	Seed protein	NFE2L2	Intermediate protein
TRIM28	Seed protein	RNF4	Intermediate protein
TRIM28	Seed protein	CHEK2	Intermediate protein
TRIM28	Seed protein	E2F1	Intermediate protein
TRIM28	Seed protein	E2F4	Intermediate protein
TRIM28	Seed protein	CDKN2A	Intermediate protein
TRIM28	Seed protein	STAT1	Intermediate protein
TRIM28	Seed protein	ATRX	Intermediate protein
TRIM28	Seed protein	H2AFX	Intermediate protein
TRIM28	Seed protein	STAT3	Intermediate protein
TRIM28	Seed protein	PARP1	Intermediate protein
TRIM28	Seed protein	POU5F1	Intermediate protein
TRIM28	Seed protein	PFDN5	Intermediate protein
TRIM28	Seed protein	DNMT1	Intermediate protein
TRIM28	Seed protein	CEBPB	Intermediate protein
TRIM28	Seed protein	HDAC3	Intermediate protein
TRIM28	Seed protein	WHSC1	Intermediate protein
TRIM28	Seed protein	UHRF1	Intermediate protein
TRIM28	Seed protein	HIF1A	Intermediate protein
TRIM28	Seed protein	VHL	Intermediate protein
TRIM28	Seed protein	TRIM28	Seed protein
TRIM28	Seed protein	TRIM24	Intermediate protein
IKBKE	Intermediate protein	CHUK	Intermediate protein
IKBKE	Intermediate protein	PML	Seed protein
IKBKE	Intermediate protein	RELA	Intermediate protein
IKBKE	Intermediate protein	SUMO1	Intermediate protein
TRIM24	Intermediate protein	PML	Seed protein

TRIM24	Intermediate protein	TRIM28	Seed protein
TRIM24	Intermediate protein	TRIM24	Intermediate protein

X2K

Name	Simple name	P-value	Z-score	Combined score	Targets																	
SOX2_CHEA	SOX2	{}	3.18E-08	0	0	SLCO5A1	FOS	SPP1	CTGF	LAPTM4A	LY6E	HSP90AB1	ITGAV	ERRFI1	ID1	CD24	PTPRO	ENC1	PTMA	BMP4	YWHAG	RCN1
KLF4_CHEA	KLF4	{}	2.6E-06	0	0	TXNIP	TUBA1C	TUBA1B	FOS	SPP1	LAPTM4A	HSP90AB1	GAPDH	ACTB	MIF	PTMA	BMP4	YWHAG	RCN1	IER2	JUND	ANXA2
NELFE_ENCODE	NELFE	{}	1.15E-05	0	0	ACTB	LDHA	TUBA1C	TUBA1B	TPI1	FOS	PABPC1	NPM1	TUBB4B	HSP90AB1	GAPDH						
MYC_CHEA	MYC	{}	6.61E-05	0	0	RPL6	IER2	RPL28	TUBB4B	LAPTM4A	NOP10	HSP90AB1	PKM	NOP58	MIF	PFN1	TUBB	PABPC1	PTMA	NPM1	RAN	
TRIM28_CHEA	TRIM28	{}	0.000145	0	0	SOX9	HIF1A	ENC1	PTMA	PDIA3	IGFBP2	GGH	HSPB1	ITGAV								
NANOG_CHEA	NANOG	{}	0.000332	0	0	CKS2	SPP1	TUBB4B	CTGF	CDH3	IGFBP2	IFITM1	IFITM2	SPARC	ID1	ENC1	NQO1	BMP4	YWHAG	RCN1		
TCF3_ENCODE	TCF3	{}	0.000569	0	0	TUBA1C	TUBA1B	HSP90AB1	ITGAV	NDUFA4	ERRFI1	ACTB	ENC1	CD63	HIF1A	LDHA	TUBB4B	PHLDA1	PKM	ACSL1	PFN1	TMEM123
ESR1_CHEA	ESR1	{}	0.000576	0	0	ACTB	C3	ID3	SERPINA1	CTSD	FOS	TFF1										
NFIC_ENCODE	NFIC	{}	0.001063	0	0	LDHA	ENC1	FOS	PHLDA1	CBX3	NOP10	HSPB1	PKM	GAPDH								
PML_ENCODE	PML	{}	0.00198	0	0	FOS	ITGAV	GAPDH	NOP58	PABPC1	PTMA	LDHA	TPI1	TUBB4B	CSTB	CBX3	ATP1A1	TUBA1C	TUBA1B	CKS2	SLC12A2	MIF
TAF7_ENCODE	TAF7	{}	0.005283	0	0	RPL6	RPL28	JUND	TUBA1C	TUBA1B	FOS	CKS2	CKB	TUBB4B	HSP90AB1	Sep-15	PABPC1	CBX3				
HDAC2_ENCODE	HDAC2	{}	0.005485	0	0	SYPL1	ID1	TUBA1B	PKM	NQO1	DEK											
CHD1_ENCODE	CHD1	{}	0.00636	0	0	RPL28	LDHA	S100A11	JUND	TUBA1C	FOS	CKS2	LAPTM4A	PKM	PFN1	ITM2B	MIR21	PTMA				
CEBPD_ENCODE	CEBPD	{}	0.006536	0	0	TXNIP	TUBA1C	TUBA1B	FOS	CKS2	TUBB4B	PKM	ACTB	PFN1	ID1	CTSH	MIR21	CBX3	SLC40A1			
GATA2_CHEA	GATA2	{}	0.009891	0	0	TXNIP	TUBA1C	FOS	ERRFI1	IER2	S100A11	IFNGR2	PLCB4	PKM	PDZK1IP1	QPCT	ATP1B1	UCA1	ATP1A1			
RUNX1_CHEA	RUNX1	{}	0.011762	0	0	TUBA1C	FOS	DYNLL1	CKS2	S100A6	LY6E	PGM1	ID3	CTSE	CTSD	NPM1	IER2	S100A11	S100A10	TUBB4B	SYPL1	ACSL1
ZNF384_ENCODE	ZNF384	{}	0.014636	0	0	RPL6	HIF1A	TUBA1C	TUBA1A	ANXA2	PGAM1	DYNLL1	GLO1	HSP90AB1	NOP58	ACTB	ID3	YWHAG				
NFE2L2_CHEA	NFE2L2	{}	0.021255	0	0	COL1A2	FOXQ1	SLPI	ENC1	ETS2	BMP4	YWHAG	PHLDA1	PKM	ZWINT	CSTB	NQO1	QPCT	ATP1B1	SLC40A1	DCN	
ZKSCAN1_ENCODE	ZKSCAN1	{}	0.02441	0	0	AP1S1	ITM2C	ANXA2	ALDOA													
FOXA2_ENCODE	FOXA2	{}	0.024921	0	0	ERRFI1	TSPAN8	TFF3	FOXQ1	RNF43	TMEM123	S100P										
IRF8_CHEA	IRF8	{}	0.025049	0	0	PSME2	HSP90AB1	SECTM1	PSMB9													
KAT2A_ENCODE	KAT2A	{}	0.049534	0	0	HSP90AB1	RPL6	PTMA														
ZMIZ1_ENCODE	ZMIZ1	{}	0.067289	0	0	TUBA1C	HSP90AB1	ACTB	PTMA	IER2	RPL28	JUND	TPI1	TUBB4B	PKM	MIR21	ALDOA	PDIA3				
ZBTB7A_ENCODE	ZBTB7A	{}	0.076758	0	0	FXYD3	ACTB	PTMA	ETS2	AP1S1	CKB	TUBB4B	CSTB	ATP1B1	CBX3	PSME2	TUBA1C	TUBA1B	DYNLL1	HSP90AB1	ID1	CD63
TCF7L2_ENCODE	TCF7L2	{}	0.080085	0	0	S100A10	PHLDA1	ACTB	ZWINT	ID1	PDIA3	ATP1B1	TMEM123	GGH								
SIN3A_ENCODE	SIN3A	{}	0.082299	0	0	TUBA1C	TUBA1B	CKS2	HSP90AB1	GAPDH	SLC12A2	NOP58	ID1	PABPC1	NPM1	HIF1A	LDHA	PHLDA1	PKM	ALDOA		
E2F1_CHEA	E2F1	{}	0.085227	0	0	TUBA1B	MIF	ID3	RAN	AP1S1	HIF1A	S100A10	PLCB4	PFN1	ALDOA	NQO1	DEK					
IRF1_ENCODE	IRF1	{}	0.089837	0	0	PSME2	JUND	IFI6	ISG15	YWHAG												
SPI1_CHEA	SPI1	{}	0.091627	0	0	TUBA1C	PGAM1	FOS	CKS2	S100A4	ITGAV	CD14	ID3	RPL6	IER2	RPL28	ALDOA	PDIA3	TMEM123			
SALL4_CHEA	SALL4	{}	0.101628	0	0	ID3	PRSS23	CTGF	RCN1	PKM	DEK											

GATA1_CHEA	GATA1	{}	0.10979	0	0	FOS	ERRFI1	ACTB	LGALS3BP	IFNGR2	SYPL1	IFITM3	PDZK1IP1	ATP1B1	TMEM123	SLC40A1						
EGR1_ENCODE	EGR1	{}	0.111039	0	0	IER2	COL1A1	TUBA1A	NDUFA4	PABPC1	CD63	CBX3	HSPB1									
TCF3_CHEA	TCF3	{}	0.117418	0	0	SLCO5A1	SPP1	CTGF	SLPI	ID1	ID3	PTMA	YWHAG	RCN1	PKM	IFITM1	IFITM2	HSPB1				
MYC_ENCODE	MYC	{}	0.131209	0	0	TUBA1C	TUBA1B	CKS2	NOP10	HSP90AB1	NOP58	MIF	PABPC1	PTMA	CD63	NPM1	YWHAG	RAN	RPL6	LDHA	TUBB4B	PARK7
UBTF_ENCODE	UBTF	{}	0.140526	0	0	ITGAV	PTMA	ETS2	TUBB4B	ATP1A1	DEK	DYNLL1	SLC12A2	ID3	JUND	IFNGR1	PHLDA1	SYPL1	ACSL1	ITM2B	ITM2C	ALDOA
RELA_ENCODE	RELA	{}	0.145713	0	0	PARK7	HSP90AB1	PKM	ACTB	PFN1	PTMA	IL32										
VDR_CHEA	VDR	{}	0.151763	0	0	PABPC1	ITM2C															
EGR1_CHEA	EGR1	{}	0.154741	0	0	IFITM2	SERPINA1	SECTM1	IFNGR1	CSTB												
ETS1_ENCODE	ETS1	{}	0.166957	0	0	HSP90AB1	PKM	CKS2														
HNF4A_ENCODE	HNF4A	{}	0.168917	0	0	PSME2	C3	FABP1	CTGF	FOXQ1	LAPTM4A	ID1	SECTM1	ENC1	IL32	JUND	IFNGR1	CCL20	TUBB4B	ATP1B1		
RCOR1_ENCODE	RCOR1	{}	0.179202	0	0	IFNGR2	FOS	DYNLL1	RNF43	GLO1	HSP90AB1	PKM	PTMA	DEK								
PPARG_CHEA	PPARG	{}	0.205193	0	0	TUBA1C	FOS	PHLDA1	ACTB	ACSL1	PTMA	YWHAG										
FOXP2_ENCODE	FOXP2	{}	0.216181	0	0	IER2	JUND	PABPC1														
E2F6_ENCODE	E2F6	{}	0.230011	0	0	GAPDH	NOP58	PTMA	ETS2	CKB	ATP1B1	ATP1A1	DEK	TUBA1B	DYNLL1	MIF	NPM1	RCN1	SYPL1	GLO1	MEST	PFN1
BCL3_ENCODE	BCL3	{}	0.23069	0	0	ACTB	PFN1	JUND	PTMA													
TP63_CHEA	TP63	{}	0.268988	0	0	ERRFI1	DPEP1	RNF128	ANXA2	IFNGR2	ASCL2	CDH3	GPX2	PKM	ACSL1	C10ORF99	QPCT	ATP1B1	MFSD4	ATP1A1		
SMAD4_CHEA	SMAD4	{}	0.26964	0	0	S100A10	TSPAN8	ID1	ID3	ENC1	ETS2	CBX3										
AR_CHEA	AR	{}	0.27508	0	0	PGM1	ERRFI1	MMP7	PLCB4	CCL20	APP	SOX9	ZWINT	CEACAM6	PDIA3	CXCL8	CXCL1					
FOXA1_ENCODE	FOXA1	{}	0.279993	0	0	FOS	CXCL8	S100P														
SRF_ENCODE	SRF	{}	0.281014	0	0	ACTB	IER2	PFN1	FOS													
TAF1_ENCODE	TAF1	{}	0.291276	0	0	LAPTM4A	GAPDH	NOP58	PTMA	TPI1	PARK7	TUBA1C	TUBA1B	MIF	Sep-15	NPM1	YWHAG	RPL28	JUND	PHLDA1	PFN1	FOS
CREB1_CHEA	CREB1	{}	0.310243	0	0	TUBA1C	TUBA1A	FOS	HSP90AB1	GAPDH	NOP58	ACTB	ID1	PABPC1	NPM1	YWHAG	IER2	RPL28	LDHA	JUND		
ZC3H11A_ENCODE	ZC3H11A	{}	0.32326	0	0	RNF43	FXYD3															
STAT3_ENCODE	STAT3	{}	0.323457	0	0	RPL28	IFNGR1	FOS	LAPTM4A	MIR21	ALDOA	CBX3	YWHAG									
CTCF_ENCODE	CTCF	{}	0.334767	0	0	FXYD3	SPP1	NOP10	ACTB	PTMA	ETS2	PARK7	DEK	COL1A1	COL1A2	DYNLL1	ENC1	YWHAG	PHLDA1	RNF43	CEMIP	APP
PBX3_ENCODE	PBX3	{}	0.346452	0	0	TUBA1B	LAPTM4A	HSP90AB1	GAPDH	ACTB	PTMA	CD63	HIF1A	JUND	TPI1	TUBB4B	PKM	PFN1				
STAT5A_ENCODE	STAT5A	{}	0.347744	0	0	MIR21	LAPTM4A	PKM														
ERG_CHEA	ERG	{}	0.37146	0	0	ACSL1	S100A4															
CEBPB_ENCODE	CEBPB	{}	0.37146	0	0	ACTB	SLPI															
BHLHE40_ENCODE	BHLHE40	{}	0.379171	0	0	PFN1	HIF1A	PTMA	TMEM123													
RFX5_ENCODE	RFX5	{}	0.384333	0	0	AP1S1	TUBA1A	CKS2	PKM	IFI27	ATP1A1											
SPI1_ENCODE	SPI1	{}	0.403226	0	0	LAPTM4A	ITGAV	PABPC1	CKB	CBX3	ATP1A1	TUBA1B	DYNLL1	CD14	ID3	YWHAG	RPL28	S100A11	IFNGR1	PDIA3		
POU5F1_CHEA	POU5F1	{}	0.414925	0	0	IFITM1	IFITM2	ASCL2														
BRCA1_ENCODE	BRCA1	{}	0.429961	0	0	TXNIP	GAPDH	NOP58	PTMA	PARK7	COL1A1	TUBA1B	DYNLL1	MIF	Sep-15	NPM1	JUND	PHLDA1	MEST	ALDOA	FOS	ACTB
MYOD1_ENCODE	MYOD1	{}	0.43951	0	0	HSPB1	TUBA1B															
E2F4_ENCODE	E2F4	{}	0.450839	0	0	IER2	TUBA1B	NOP58	ALDOA	PTMA	NPM1	DEK										
RAD21_ENCODE	RAD21	{}	0.458743	0	0	COL1A1	COL1A2	FXYD3	SPP1	NOP10	CD14	ENC1	ETS2	PHLDA1	RNF43	CEMIP	APP					

PPARD_CHEA	PPARD	{}	0.470754	0	0	TXNIP	QPCT	TGFBI														
STAT3_CHEA	STAT3	{}	0.472027	0	0	FOS	PABPC1															
SMC3_ENCODE	SMC3	{}	0.48872	0	0	COL1A1	COL1A2	FXYD3	NOP10	ENC1	ETS2	PHLDA1	RNF43	CEMIP	APP	ITM2C						
MAX_ENCODE	MAX	{}	0.489936	0	0	TXNIP	NOP10	NOP58	PABPC1	PTMA	ETS2	LDHA	TUBB4B	PARK7	TUBA1C	TUBA1B	HSP90AB1	SLC12A2	ERRFI1	MIF	NPM1	RAN
USF1_ENCODE	USF1	{}	0.509166	0	0	TUBA1B	S100A6	NOP10	GAPDH	ENC1	CTSD	PTMA	CD63	NPM1	HIF1A	LDHA	APP	CSTB				
CBX3_ENCODE	CBX3	{}	0.518606	0	0	NOP10																
FOSL2_ENCODE	FOSL2	{}	0.525455	0	0	S100A10	TUBA1C															
SUZ12_CHEA	SUZ12	{}	0.545151	0	0	PROM1	ASCL2	DUOX2	MFSD4	PSMB9	FOXQ1	CD14	ID3	CD24	PTPRO	BMP4	PRSS23	BACE2	APP	SOX9		
TP53_CHEA	TP53	{}	0.545479	0	0	COL1A1	ENC1	TUBB4B														
FOXM1_ENCODE	FOXM1	{}	0.575257	0	0	TUBB4B																
EZH2_CHEA	EZH2	{}	0.627884	0	0	CD24	FOXQ1															
USF2_ENCODE	USF2	{}	0.636471	0	0	LAPTM4A	GAPDH	CTSD	PTMA	NPM1	HIF1A	LDHA	CSTB									
ATF2_ENCODE	ATF2	{}	0.660147	0	0	TXNIP	FOS	NOP10	GAPDH	NOP58	PTMA	PARK7	CBX3	TUBA1C	TUBA1B	TUBA1A	CKS2	HSP90AB1	Sep-15	TSPAN1	ID1	NPM1
TRIM28_ENCODE	TRIM28	{}	0.660448	0	0	HSP90AB1																
FOS_ENCODE	FOS	{}	0.676666	0	0	TXNIP	TUBB4B	LAPTM4A	GLO1	PTMA												
BCLAF1_ENCODE	BCLAF1	{}	0.776841	0	0	TUBA1B	HSP90AB1	PTMA	JUND	MIR21	CBX3											
YY1_CHEA	YY1	{}	0.798867	0	0	PTMA	DCN															
NRF1_ENCODE	NRF1	{}	0.802576	0	0	LAPTM4A	PTMA	AP1S1	LDHA	TUBB4B	PARK7	TUBA1B	PGAM1	DYNLL1	CKS2	NPM1	YWHAG	PFN1	ZWINT			
ZBTB33_ENCODE	ZBTB33	{}	0.876682	0	0	SLC12A2	NOP58	NPM1	GGH													
SP1_ENCODE	SP1	{}	0.880091	0	0	PSME2	TUBB4B	SYPL1	PTMA													
NFYB_ENCODE	NFYB	{}	0.909934	0	0	TXNIP	LAPTM4A	GAPDH	PTMA	CKB	ATP1B1	DEK	COL1A1	TUBA1C	Sep-15	RPL28	JUND	RNF43	GLO1	ZWINT	NQO1	TSPAN13
FLI1_ENCODE	FLI1	{}	0.932416	0	0	RPL6	IER2	LDHA														
IRF3_ENCODE	IRF3	{}	0.937984	0	0	TUBB4B	LAPTM4A	GLO1														
NFYA_ENCODE	NFYA	{}	0.949461	0	0	TXNIP	LAPTM4A	ACTB	PTMA	TUBB4B	PKM	PSME2	CKS2	ERRFI1	Sep-15	ID1	HIF1A	GLO1	ZWINT			
EZH2_ENCODE	EZH2	{}	0.952526	0	0	SLCO5A1																
REST_ENCODE	REST	{}	0.968482	0	0	CEMIP																
CREB1_ENCODE	CREB1	{}	0.97037	0	0	FOS	LAPTM4A	NOP58	PABPC1	LDHA	PARK7	CBX3	ATP1A1	CKS2	HSP90AB1	ID1	JUND	IFNGR1				
SIX5_ENCODE	SIX5	{}	0.989656	0	0	PABPC1	YWHAG	RPL6	GLO1													
SP2_ENCODE	SP2	{}	0.994178	0	0	LAPTM4A	PTMA	TUBB4B														
YY1_ENCODE	YY1	{}	0.997994	0	0	GAPDH	NOP58	PTMA	TUBB4B	CBX3	DEK	Sep-15	NPM1	YWHAG	RAN	IER2	HIF1A	GLO1				
ELF1_ENCODE	ELF1	{}	0.999336	0	0	NOP10	PABPC1	TUBA1C	CKS2	MIF	ID3	CD63	RPL6	IER2	PFN1							
REST_CHEA	REST	{}	0.999386	0	0	OLFM4	ENC1	NPM1														
GABPA_ENCODE	GABPA	{}	0.999977	0	0	NOP10	RPL6	IER2	RPL28	PFN1												
CSNK2A1	1.99E-28	0	0	SPI1	EHMT2	DAXX	CSNK2A1	PPARGC1A	JUN	EZH2	NELFE	FOS	SIRT1	POU5F1	EGFR	STAT1	ATAD2	STAT3	PIAS1	MSH2	FOXO1	PIN1
CDK1	5.41E-26	0	0	EHMT2	CSNK2A1	GATA2	PPARGC1A	JUN	EZH2	NELFE	FOS	SIRT1	EGFR	STAT1	ATAD2	STAT3	CCNT1	MSH2	MAPKAPK2	FOXO1	SP1	NFE2L2
MAPK14	9.13E-23	0	0	EHMT2	PPARGC1A	JUN	EZH2	TUBA1A	NELFE	FOS	POU5F1	EGFR	STAT1	ATAD2	STAT3	MSH2	MAPKAPK2	FOXO1	SP1	NFE2L2	HSP90AA1	ATRX
CDK4	4.79E-17	0	0	RB1	CCNH	TRRAP	MYC	SKP2	DNMT1	FOXO1	SMAD2	JUN	SMAD3	ESR1	SP1	CDK2	BCL2	TCF3	SUMO1	HSP90AA1	PARP1	CEBPA

GSK3B	6.45E-17	0	0	GATA2	JUN	FOS	EGFR	ATAD2	PIAS1	MSH2	SP1	ATRX	UHRF1	E2F1	TRIM24	SMARCA4	RELA	UBE3A	TCF3	WHSC1	NCOR2	NCOR1
AKT1	3.16E-16	0	0	TRRAP	SOX2	MYC	CSNK2A1	GATA2	PPARGC1A	JUN	EZH2	FOS	POU5F1	NCOR1	EGFR	CDKN2A	STAT1	STAT3	SKI	PIAS2	KAT6A	MAPKAPK2
CDK2	3.74E-16	0	0	JUN	FOS	EGFR	ATAD2	MSH2	SP1	ATRX	UHRF1	E2F1	E2F4	TRIM28	SMARCA4	UBE3A	TCF3	WHSC1	RNF4	TDG	PRKDC	NCOR2
DNAPK	1.85E-15	0	0	TRRAP	SOX2	KAT5	TRIM28	CHEK2	PRKCD	PRKDC	SMAD2	JUN	ESR1	SP1	TCF3	HSP90AA1	CHUK	PARP1	ATRX	H2AFX	RELA	RAD51
MAPK1	8.66E-14	0	0	RB1	MYC	CSNK2A1	HIF1A	JUN	EZH2	FOS	POU5F1	NCOR2	EGFR	CDKN2A	STAT1	STAT3	MAPKAPK2	FOXO1	SP1	CDK2	BCL2	CHUK
ERK1	1.51E-12	0	0	KAT5	MYC	PRKCD	HIF1A	SMAD2	JUN	SMAD3	ESR1	SP1	BCL2	TCF3	FOS	NCOR2	CEBPB	EGFR	RELA	STAT1	STAT3	NFIC
ATM	2.69E-12	0	0	RB1	SOX2	KAT5	TRIM28	CHEK2	DAXX	PRKDC	HIF1A	SMARCA4	EZH2	PARP1	ATAD2	TTC5	MEN1	MSH2	SP1	CDK2	HSP90AA1	H2AFX
MAPK3	4.27E-12	0	0	MYC	JUN	NCOA2	FOS	POU5F1	EGFR	STAT1	STAT3	MAPKAPK2	FOXO1	SMAD2	SMAD3	ESR1	SP1	CDK2	BCL2	CHUK	CEBPA	CEBPB
ERK2	6.18E-11	0	0	RB1	MYC	MAPKAPK2	SMAD2	JUN	SMAD3	ESR1	SP1	BCL2	TCF3	FOS	NCOR2	CEBPB	EGFR	RELA	STAT1	STAT3		
HIPK2	1.12E-10	0	0	TRIM28	MYC	PRKDC	HIF1A	SMAD2	SMAD3	BCL2	SUMO1	SIN3A	CHUK	PARP1	SIRT1	NCOR1	CEBPB	RELA	PML			
CDC2	2.88E-10	0	0	RB1	CCNH	KAT5	TRIM28	MYC	SKP2	CSNK2A1	PRKDC	JUN	EZH2	XPO1	PARP1	FOS	NCOR1	RELA	ATAD2	STAT3	FOXO1	ZBTB16
ABL1	1.48E-09	0	0	RB1	SPI1	UBE3A	KAT5	MYC	PRKCD	JUN	ESR1	BCL2	FOS	CEBPB	HDAC2	EGFR	STAT1	STAT3	RAD51			
CK2ALPHA	4.61E-09	0	0	RB1	SPI1	CCNH	MYC	JUN	SMARCA4	NCOR2	RELA	UBE3A	VDR	SP1	SUMO1	SIN3A	HSP90AA1	ARNT	HDAC2	HDAC3	PML	
JNK1	4.78E-09	0	0	MYC	FOS	SIRT1	HIF1A	RELA	E2F1	SMAD2	JUN	SMAD3	STAT3	SP1	BCL2							
RPS6KA3	1.33E-08	0	0	TRRAP	MYC	PRKDC	JUN	FOS	POU5F1	NCOR2	KAT2B	EGFR	CDKN2A	STAT1	STAT3	MAPKAPK2	SMAD3	ESR1	BCL2	SIN3A	CHUK	MAPK3
MAPK8	1.79E-08	0	0	SOX2	MYC	JUN	FOS	SIRT1	EGFR	CDKN2A	STAT1	STAT3	SP1	BCL2	NFE2L2	SIN3A	H2AFX	MAPK1	KLF4	STUB1		
CSNK2A2	8.15E-08	0	0	CCNH	TRRAP	SOX2	MYC	JUN	CDK2	BCL2	NELFE	FOS	POU5F1	RPL11	EGFR	STAT1						
GSK3BETA	1.29E-07	0	0	MYC	DNMT1	FOXO1	PPARGC1A	JUN	SMAD3	ESR1	BCL2	PIN1	PARP1	CEBPB	E2F1	STAT3						
JNK2	4.85E-07	0	0	RB1	PRKDC	RELA	MYC	SMAD2	JUN	FOS												
MAPK11	1.05E-06	0	0	MAPKAPK2	PPARGC1A	JUN	ESR1	BCL2	FOS	CEBPB	EGFR	E2F1	STAT1	PML								
RAF1	2.17E-06	0	0	RB1	MYC	PRKCD	ESR1	BCL2	PIN1	HSP90AA1	PARP1	FOS	MAPK1	MAPK3								
PRKDC	3.35E-06	0	0	TRRAP	CHEK2	MYC	SKP2	PRKDC	HSP90AA1	H2AFX	EGFR	RELA	RAD51									
PRKCD	5.12E-06	0	0	TRIM28	PRKCD	PRKDC	JUN	NFE2L2	FOS	HDAC5	MAPK1	MAPK3	STAT1	STAT3								
CDK3	7.27E-06	0	0	RB1	CCNH	E2F1	JUN	ESR1	CDK2													
PKBALPHA	7.71E-06	0	0	MYC	SKP2	PRKDC	PPARGC1A	EZH2	NCOR2	RELA	FOXO1	SMAD2	SMAD3	ESR1	HSP90AA1	CHUK						
SRC	1.66E-05	0	0	RB1	SPI1	MYC	PRKCD	JUN	FOS	POU5F1	EGFR	STAT1	STAT3	MAPKAPK2	BCL2	CEBPB	E2F4	KLF4	CALM1	SMAD3	ESR1	MAPK1
PRKCA	2.35E-05	0	0	SPI1	JUN	TUBA1A	FOS	POU5F1	EGFR	STAT1	ATAD2	STAT3	MAPKAPK2	NFE2L2	STUB1	RELA	VDR	TDG	RB1	MYC	NCOR2	NCOR1
CHUK	2.46E-05	0	0	MYC	IKBKE	PIAS1	NCOR2	NCOR1	RELA	ESR1	CALM1											
CSNK1E	2.7E-05	0	0	MYC	POU5F1	DNMT1	CDKN2A	STAT1	ESR1	SMARCA4												
PRKACA	3.15E-05	0	0	SPI1	PPARGC1A	JUN	FOS	POU5F1	EGFR	STAT3	PSMC5	ZBTB16	PIN1	HSP90AA1	UHRF1	TBX2	RELA	UBE3A	VDR	RB1	MYC	HIF1A
CHEK1	4.09E-05	0	0	RB1	SOX2	TRIM28	MYC	SKP2	EZH2	NELFE	FOS	SIN3A	H2AFX	E2F1	E2F4							
ATR	5E-05	0	0	CHEK2	DAXX	MSH2	PRKDC	SP1	H2AFX	E2F1	CDKN2A	PML										
EGFR	5.89E-05	0	0	PRKCD	PRKDC	JUN	ESR1	CALM1	IKBKE	HSP90AA1	FOS	EGFR	STAT1	STAT3								
CDK8	6.18E-05	0	0	CCNH	E2F1	SMAD3	STAT1	STAT3														
MAP3K7	6.86E-05	0	0	SMAD3	SMURF1	CALM1	HSP90AA1	HDAC5	HDAC7	E2F1	E2F4											
CHEK2	7.49E-05	0	0	RB1	TRIM28	CHEK2	E2F1	VHL	PML													
IKBKE	0.000133	0	0	IKBKE	SKP1	CUL1	RELA	STAT1	ESR1	CALM1												

MAPK10	0.000135	0	0	MYC	FOS	EGFR	JUN	CDKN2A	BCL2				
CDK5	0.00024	0	0	RB1	MYC	DNMT1	HIF1A	FOXO1	BCL2	NFE2L2	HDAC5	STAT3	STUB1
DNA-PK	0.000333	0	0	HSP90AA1	JUN	PRKDC							
CK2-A	0.000364	0	0	SPI1	HSP90AA1	HDAC2	RELA	JUN	VDR				
IKKALPHA	0.000385	0	0	HSP90AA1	CHUK	RELA	NCOR2						
MAP2K1	0.00052	0	0	MYC	FOS	EGFR	MAPK1	MAPK3	STAT3				
TGFBR2	0.00052	0	0	MYC	HSP90AA1	SMAD2	SMAD3	PML	SP1				
GSK-3-B	0.00075	0	0	CEBPB	RELA	MYC	ESR1						
GSK3ALPHA	0.000783	0	0	MYC	PARP1	FOXO1	JUN	ESR1					
TBK1	0.000823	0	0	RPL11	RELA	E2F1	STAT3	ESR1	CALM1				
JAK2	0.00085	0	0	MAPK1	MAPK3	STAT1	STAT3	EZH2					
RPS6KA1	0.000876	0	0	FOS	CEBPB	MAPK1	MAPK3	JUN	ESR1				
ADRBK1	0.000883	0	0	PRKCD	EGFR	MAPK3							
PIM1	0.00092	0	0	MYC	SKP2	POU5F1	UHRF1	RELA					
PKCEPSILON	0.000931	0	0	HSP90AA1	PRKCD	MAPK1	MAPK3	SP1	BCL2				
PRKCZ	0.001049	0	0	RELA	MAPK1	MAPK3	STAT3	SP1	PIN1				
CDK9	0.00145	0	0	RB1	CCNT1	NCOA2	SMAD3						
IKBKB	0.001473	0	0	IKBKE	PARP1	FOS	RELA	CDKN2A	CALM1				
MAPK9	0.001473	0	0	MYC	H2AFX	FOS	EGFR	JUN	STAT3				
PKD1	0.001653	0	0	XPO1	HDAC5	EGFR	HDAC7	JUN					
CK2A2	0.001768	0	0	MYC	HSP90AA1	HDAC2	RELA	JUN					
NPM/ALK	0.002457	0	0	MSH2	STAT1								
PDHK1	0.002909	0	0	HSP90AA1	CHUK	PRKCD	EGFR	FOXO1					
PKR	0.002959	0	0	HSP90AA1	CHUK	STAT1	STAT3						
TTK	0.002962	0	0	CHEK2	CDK2	XPO1	HSP90AA1	E2F1	E2F4				
PRKAA1	0.003391	0	0	RB1	PIAS1	FOXO1	NFE2L2	HDAC5	EGFR				
IKK-A	0.00341	0	0	NCOR2	RELA								
IKKBETA	0.004013	0	0	RELA	CHUK	STAT3	FOS						
ERBB2	0.004034	0	0	ESR1	HSP90AA1	NCOR1	EGFR	STAT1	STAT3				
BARK1	0.00428	0	0	PRKCD	HDAC5	EGFR	MAPK3	PIN1					
PKBBETA	0.004311	0	0	FOXO1	RELA	CHUK	ESR1						
MTOR	0.004387	0	0	EHMT2	MYC	DNMT1	JUN	ESR1	STAT3				
CHK2	0.004508	0	0	CHEK2	E2F1								
MAP2K3	0.004624	0	0	MAPK3	SMAD2	SMAD3	SMURF1						
PKCTHETA	0.005654	0	0	CHUK	PRKCD	MAPK1	MAPK3						
RSK3	0.005746	0	0	MAPK1	MAPK3								
PKBGAMMA	0.007494	0	0	FOXO1	RELA	CHUK							

MAP3K8	0.012474	0	0	HSP90AA1	MAPK1	MAPK3	CALM1				
RET	0.015134	0	0	MAPK1	MAPK3	STAT3					
ALK	0.015134	0	0	RELA	HSP90AA1	STAT3					
PLK3	0.017258	0	0	HIF1A	CHEK2	JUN					
DYRK2	0.017258	0	0	MYC	JUN	STAT3					
PRKD1	0.018044	0	0	HDAC5	EGFR	HDAC7	JUN				
ACTR2B	0.018047	0	0	SMAD2	SMAD3						
PRKAA2	0.018379	0	0	HDAC5	PPARGC1A	PARP1					
PDGFRBETA	0.018379	0	0	EGFR	PRKCD	STAT3					
RPS6KA5	0.019541	0	0	RELA	TRIM28	STAT3					
CDK7	0.019642	0	0	CCNH	E2F1	ESR1	CDK2				
LYN	0.019771	0	0	SPI1	TRIM28	MYC	CSNK2A1	PRKCD	STAT1		
PAK2	0.020474	0	0	MYC	HDAC5	SMAD2	JUN				
PKCIOTA	0.023262	0	0	RELA	HSP90AA1	CHUK					
CSNK1D	0.024583	0	0	DNMT1	UHRF1	HIF1A					
NLK	0.024583	0	0	FOXO1	SETDB1	STAT3					
LCK	0.024987	0	0	SPI1	PRKCD	ESR1	MAPK1	MAPK3	STAT1		
CDKN1B	0.02599	0	0	CDK2							
BAZ1B	0.02599	0	0	H2AFX							
PCNA	0.02599	0	0	CDK2							
EP400	0.02599	0	0	TRRAP							
AURKA	0.026898	0	0	VHL	ESR1	SKI	PIN1				
GSK3A	0.03178	0	0	MYC	CEBPA	CEBPB					
PKN3	0.033024	0	0	GATA2	EGFR						
PKMYT1	0.033024	0	0	SMARCA4	RAD51						
PLK1	0.033392	0	0	CHEK2	MYC	XPO1	ATAD2	PSMC5	CCNT1	PIN1	RAD51
PIM3	0.035865	0	0	PML	POU5F1						
HIPK1	0.038798	0	0	DAXX	KAT6A						
WEE1	0.038798	0	0	HSP90AA1	CDK2						
PBK	0.044925	0	0	E2F4	JUN						
PIM2	0.048114	0	0	MYC	POU5F1						
AKT2	0.049101	0	0	CHUK	PPARGC1A	ESR1					
TGFBR1	0.049101	0	0	SMAD2	SMURF1	STAT1					
MAP3K1	0.051026	0	0	ESR1	CALM1	TUBA1A	HSP90AA1	STAT3			
MAP3K20	0.051312	0	0	CHEK2							
MOK	0.051383	0	0	MYC	JUN						
MELK	0.05473	0	0	SMAD2	SMAD3						

AURKB	0.057054	0	0	RB1	PRKDC	PIN1	NELFE	HDAC5
JAK3	0.061644	0	0	STAT1	EZH2			
CDK6	0.061644	0	0	RB1	CEBPA			
UHMK1	0.061644	0	0	SMAD2	SMURF1			
CAMK4	0.061644	0	0	HDAC5	RELA			
MAPK7	0.061644	0	0	FOS	PML			
MAPKAPK2	0.063453	0	0	TRIM28	PIAS1	MAPKAPK2		
PRKACB	0.065208	0	0	STAT1	FOS			
RPS6KB1	0.065646	0	0	PPARGC1A	CDKN2A	ESR1		
PKN1	0.068839	0	0	HDAC5	EGFR			
CAMK1	0.068839	0	0	HDAC5	HDAC7			
DAPK1	0.072535	0	0	MAPK3	PIN1			
PAK1	0.075486	0	0	PRKCD	FOXO1	ESR1	EGFR	
CAM-KIV	0.07598	0	0	HDAC5				
PKM	0.07598	0	0	STAT3				
MAPK13	0.079518	0	0	FOS	MAPKAPK2	JUN		
CSK	0.080115	0	0	EGFR	PML			
MAP3K5	0.083993	0	0	IKBKE	DAXX			
NIK	0.088076	0	0	CEBPB				
HSPB8	0.088076	0	0	MAPK3				
CAMK2A	0.088442	0	0	MYC	SMAD2	NCOR2	HDAC5	
INSR	0.089417	0	0	FOS	JUN	STAT1		
MAP3K14	0.094305	0	0	CALM1	CHUK	RPL11	STUB1	
PRKCE	0.094553	0	0	MAPK3	STAT3	NANOG		
MARK2	0.095958	0	0	HDAC5	HDAC7			
CSNK1A1	0.097165	0	0	FOXO1	RELA	VHL		
RSK-1	0.100014	0	0	CEBPB				
PKCALPHA	0.100349	0	0	MYC	PRKCD	BCL2	EGFR	
MAPK12	0.107905	0	0	FOS	MAPKAPK2	JUN		
MAP2K2	0.108375	0	0	MAPK1	MAPK3			
DYRK1A	0.108375	0	0	FOXO1	SIRT1			
ABL2	0.108375	0	0	CEBPB	EGFR			
CK1ALPHA	0.110659	0	0	RB1	FOS	JUN		
BCR/ABL	0.111797	0	0	RAD51				
FYN	0.117345	0	0	SPI1	PRKCD	FOS	STAT1	NFE2L2
RIPK2	0.121191	0	0	HSP90AA1	CALM1			
TRRAP	0.123428	0	0	NCOR2				

RSK-2	0.123428	0	0	FOS			
CK1GAMMA1	0.123428	0	0	FOXO1			
PTK2	0.129933	0	0	SUMO1	NANOG		
PKC-Z	0.134908	0	0	RELA			
CAMKIV	0.134908	0	0	RELA			
CK1-A	0.134908	0	0	FOXO1			
PRKCI	0.138817	0	0	SP1	EZH2		
JNK3	0.146238	0	0	JUN			
TRKB	0.157422	0	0	FOS			
BRD2	0.157422	0	0	RB1			
IGF1R	0.166194	0	0	FOS	EGFR		
CAMKIIBETA	0.166194	0	0	FOS	PPARGC1A		
ABL	0.180216	0	0	RAD51	CDK2		
RIPK3	0.187144	0	0	DAXX	CALM1	HSP90AA1	
PKC-D	0.19011	0	0	STAT3			
PKAALPHA	0.193778	0	0	ESR1	FOS	RELA	
CAMKIIALPHA	0.194412	0	0	CEBPB	EGFR		
SGK1	0.218364	0	0	FOXO1	MAPK1		
TAOK3	0.221541	0	0	ZNF281			
RPS6KA6	0.221541	0	0	XPO1			
ALPK1	0.221541	0	0	IKBKE			
LMTK3	0.221541	0	0	ZBTB16			
PRKCQ	0.223188	0	0	SPI1	CHUK		
BTK	0.228021	0	0	FOS	JUN		
PDK-1	0.231747	0	0	PRKCD			
CDK13	0.231747	0	0	KAT6A			
CDKL2	0.231747	0	0	CALM1			
ROR1	0.231747	0	0	ZNF281			
ULK4	0.231747	0	0	SMURF1			
PKCETA	0.231747	0	0	PRKCD			
MINK1	0.231747	0	0	SMAD2			
STK40	0.231747	0	0	MAPKAPK2			
MAP3K3	0.237175	0	0	CALM1	TUBA1A	PFDN5	STUB1
CDK20	0.241821	0	0	CDK2			
DMPK	0.241821	0	0	SP1			
PKA-A	0.241821	0	0	RELA			
NTRK3	0.241821	0	0	FOS			

CSNK2B	0.241821	0	0	FOS			
MARK4	0.241821	0	0	SMARCA4			
PRKD3	0.251763	0	0	HDAC5			
MAP3K4	0.251763	0	0	TRRAP			
CAM-KII-A	0.251763	0	0	EGFR			
PNCK	0.251763	0	0	HSP90AA1			
MAPK15	0.251763	0	0	JUN			
MAP3K10	0.261577	0	0	TCF3			
SIK1	0.261577	0	0	NCOR2			
MAP4K4	0.261577	0	0	SMARCA4			
CDK16	0.271263	0	0	SMARCA4			
FRK	0.271263	0	0	FOS			
CSNK1G2	0.271263	0	0	EHMT2			
CSNK1G1	0.271263	0	0	RELA			
MAP3K11	0.271263	0	0	PIN1			
MERTK	0.271263	0	0	STAT3			
DYRK3	0.271263	0	0	SIRT1			
CAMK2G	0.271263	0	0	STAT1			
AURORAB	0.276605	0	0	RB1	XPO1		
RPS6KA4	0.280823	0	0	RELA			
YES	0.280823	0	0	EGFR			
PAK5	0.280823	0	0	TCF3			
MYLK	0.290259	0	0	MAPK3			
NEK1	0.290259	0	0	VHL			
EPHB3	0.290259	0	0	SMARCA4			
EPHA8	0.290259	0	0	SMARCA4			
PKC-A	0.291191	0	0	EGFR	NFE2L2		
PRKCB	0.298029	0	0	JUN	STAT3	ESR1	BCL2
VRK1	0.299572	0	0	JUN			
RPS6KA2	0.299572	0	0	MAPK3			
MKNK1	0.299572	0	0	MAPK3			
AURORAA	0.300897	0	0	NCOR1	HDAC3		
CAMKIIGAMMA	0.308765	0	0	EGFR			
ACVR1	0.308765	0	0	STAT1			
CK1EPSILON	0.308765	0	0	FOXO1			
FGFR3	0.308765	0	0	STAT1			
AKT3	0.317837	0	0	PRKCD			

IRAK1	0.317837	0	0	STAT3
DYRK1B	0.326792	0	0	HDAC5
LKB1	0.326792	0	0	PPARGC1A
PTK6	0.326792	0	0	EGFR
GRK2	0.326792	0	0	SMAD2
JAK1	0.33563	0	0	STAT1
EEF2K	0.33563	0	0	MAPKAPK2
NEK6	0.344353	0	0	STAT3
AXL	0.344353	0	0	STAT3
FER	0.344353	0	0	STAT3
EIF2AK2	0.352963	0	0	CHUK
YES1	0.352963	0	0	PRKCD
EPHB1	0.361461	0	0	STAT3
STK4	0.361461	0	0	H2AFX
BMPR1B	0.361461	0	0	SMAD2
PDGFRB	0.369848	0	0	PRKCD
PKG1CGKI	0.386297	0	0	SP1
ARAF	0.40232	0	0	PRKCD
BLK	0.410175	0	0	EGFR
HCK	0.417929	0	0	SPI1
KIT	0.417929	0	0	STAT3
RIPK1	0.433133	0	0	PML
MET	0.440587	0	0	PARP1
PRKD2	0.455206	0	0	HDAC5
PRKCG	0.469445	0	0	HSP90AA1
PAK4	0.476426	0	0	ESR1
MAP2K4	0.496828	0	0	DAXX
FGR	0.496828	0	0	HSP90AA1
CK1DELTA	0.516442	0	0	FOXO1
PKAGAMMA	0.54142	0	0	RELA
ERBB3	0.54142	0	0	EGFR
SYK	0.614099	0	0	TUBA1A
TYK2	0.624218	0	0	STAT3
PKCGAMMA	0.624218	0	0	PRKCD

KEA

Rank	Protein Kinase	Hypergeometric p-value	Z-score	Combined score	Enriched Substrates
1	CSNK2A1	1.99E-28	0	0	title=Overlapping EnrichrSubstrates" data-content="CSNK2A1 targets 67 genes from the input gene list.The full list of substrates is available below:SPI1 EHMT2 DAXX CSNK2A1 PPARGC1A JUN EZH2 NELFE FOS SIRT1 POU5F1 EGFR STAT1 ATAD2 STAT3 PIAS1 MSH2 FOXO1 PIN1 SUMO1 HSP90AA1 ATRX ARNT UHRF1 E2F1 STUB1 CCNH TRIM28 SMARCA4 NCOA2 RELA UBE3A VDR TCF3 TDG VHL RB1 TRRAP MYC PRKDC HIF1A NCOR2 NCOR1 RPL11 CDKN2A DNMT1 CUL3 SKIL CDK2 BCL2 SIN3A CHUK CEBPB HDAC2 HDAC3 KLF4 PML RAD51 SOX2 CHEK2 XPO1 SMAD3 ESR1 H2AFX PFDN5 NR2C1 MAPK1" style="cursor: pointer; text-decoration: underline dotted;">67 substrates
2	CDK1	5.41E-26	0	0	title=Overlapping EnrichrSubstrates" data-content="CDK1 targets 67 genes from the input gene list.The full list of substrates is available below:EHMT2 CSNK2A1 GATA2 PPARGC1A JUN EZH2 NELFE FOS SIRT1 EGFR STAT1 ATAD2 STAT3 CCNT1 MSH2 MAPKAPK2 FOXO1 SP1 NFE2L2 HSP90AA1 ATRX UHRF1 E2F1 E2F4 STUB1 TRIM28 SMARCA4 NCOA2 RELA UBE3A TCF3 WHSC1 TDG VHL RB1 TRRAP MYC PRKDC HIF1A NCOR2 NCOR1 CDKN2A MEN1 KAT6A DNMT1 CDK2 BCL2 SIN3A CHUK DNMT3A CEBPA CEBPB HDAC2 KLF4 PML RAD51 SOX2 KAT5 CHEK2 SKP2 SETDB1 XPO1 SMAD3 ESR1 H2AFX MAPK1 MAPK3" style="cursor: pointer; text-decoration: underline dotted;">67 substrates
3	MAPK14	9.13E-23	0	0	title=Overlapping EnrichrSubstrates" data-content="MAPK14 targets 50 genes from the input gene list.The full list of substrates is available below:EHMT2 PPARGC1A JUN EZH2 TUBA1A NELFE FOS POU5F1 EGFR STAT1 ATAD2 STAT3 MSH2 MAPKAPK2 FOXO1 SP1 NFE2L2 HSP90AA1 ATRX E2F1 SMARCA4 NCOA2 RELA TCF3 VHL RB1 TRRAP MYC PRKDC HIF1A NCOR2 NCOR1 CDKN2A DNMT1 CDK2 BCL2 SIN3A CHUK CEBPA CEBPB KLF4 RAD51 KAT5 SKP2 XPO1 PARP1 SMAD3 ESR1 NR2C1 MAPK1" style="cursor: pointer; text-decoration: underline dotted;">50 substrates
4	CDK4	4.79E-17	0	0	title=Overlapping EnrichrSubstrates" data-content="CDK4 targets 22 genes from the input gene list.The full list of substrates is available below:RB1 CCNH TRRAP MYC SKP2 DNMT1 FOXO1 SMAD2 JUN SMAD3 ESR1 SP1 CDK2 BCL2 TCF3 SUMO1 HSP90AA1 PARP1 CEBPA CEBPB CDKN2A STAT3" style="cursor: pointer; text-decoration: underline dotted;">22 substrates
5	GSK3B	6.45E-17	0	0	title=Overlapping EnrichrSubstrates" data-content="GSK3B targets 62 genes from the input gene list.The full list of substrates is available below:GATA2 JUN FOS EGFR ATAD2 PIAS1 MSH2 SP1 ATRX UHRF1 E2F1 TRIM24 SMARCA4 RELA UBE3A TCF3 WHSC1 NCOR2 NCOR1 CDKN2A KAT6A BCL2 CEBPA CEBPB PML KAT5 SETDB1 NR2C1 MAPK1 EHMT2 DAXX EZH2 SIRT1 POU5F1 STAT1 STAT3 CCNT1 MAPKAPK2 FOXO1 NFE2L2 CALM1 NCOA2 VHL RB1 TRRAP MYC HIF1A RPL11 DNMT1 SKIL CDK2 SIN3A CHUK DNMT3A KLF4 RAD51 SOX2 SKP2 SMAD2 SMAD3 SMURF1 ESR1" style="cursor: pointer; text-decoration: underline dotted;">62 substrates
6	AKT1	3.16E-16	0	0	title=Overlapping EnrichrSubstrates" data-content="AKT1 targets 29 genes from the input gene list.The full list of substrates is available below:TRRAP SOX2 MYC CSNK2A1 GATA2 PPARGC1A JUN EZH2 FOS POU5F1 NCOR1 EGFR CDKN2A STAT1 STAT3 SKI PIAS2 KAT6A MAPKAPK2 DNMT1 FOXO1 SMAD3 ESR1 SP1 CDK2 BCL2 TCF3 CHUK MAPK1" style="cursor: pointer; text-decoration: underline dotted;">29 substrates
7	CDK2	3.74E-16	0	0	title=Overlapping EnrichrSubstrates" data-content="CDK2 targets 66 genes from the input gene list.The full list of substrates is available below:JUN FOS EGFR ATAD2 MSH2 SP1 ATRX UHRF1 E2F1 E2F4 TRIM28 SMARCA4 UBE3A TCF3 WHSC1 RNF4 TDG PRKDC NCOR2 CDKN2A KAT6A BCL2 CEBPB HDAC2 PML KAT5 SETDB1 XPO1 PARP1 H2AFX MAPK1 EHMT2 DAXX EZH2 NELFB NELFE POU5F1 STAT1 STAT3 PSMC5 CCNT1 FOXO1 ZBTB16 NFE2L2 SUMO1 HSP90AA1 CCNH NCOA2 VHL RB1 MYC HIF1A RPL11 MEN1 DNMT1 CDK2 SIN3A CHUK DNMT3A RAD51 SOX2 CHEK2 SKP2 SMAD2 SMAD3 ESR1" style="cursor: pointer; text-decoration: underline dotted;">66 substrates

8	DNAPK	1.85E-15	0	0	title=Overlapping EnrichrSubstrates" data-content="DNAPK targets 19 genes from the input gene list.The full list of substrates is available below:TRRAP SOX2 KAT5 TRIM28 CHEK2 PRKCD PRKDC SMAD2 JUN ESR1 SP1 TCF3 HSP90AA1 CHUK PARP1 ATRX H2AFX RELA RAD51" style="cursor: pointer; text-decoration: underline dotted;">19 substrates
9	MAPK1	8.66E-14	0	0	title=Overlapping EnrichrSubstrates" data-content="MAPK1 targets 34 genes from the input gene list.The full list of substrates is available below:RB1 MYC CSNK2A1 HIF1A JUN EZH2 FOS POU5F1 NCOR2 EGFR CDKN2A STAT1 STAT3 MAPKAPK2 FOXO1 SP1 CDK2 BCL2 CHUK CEBPA CEBPB KLF4 PML SKP2 CALM1 NCOA2 PARP1 RELA SMAD2 SMAD3 ESR1 TCF3 NR2C1 MAPK1" style="cursor: pointer; text-decoration: underline dotted;">34 substrates
10	ERK1	1.51E-12	0	0	title=Overlapping EnrichrSubstrates" data-content="ERK1 targets 19 genes from the input gene list.The full list of substrates is available below:KAT5 MYC PRKCD HIF1A SMAD2 JUN SMAD3 ESR1 SP1 BCL2 TCF3 FOS NCOR2 CEBPB EGFR RELA STAT1 STAT3 NFIC" style="cursor: pointer; text-decoration: underline dotted;">19 substrates
11	ATM	2.69E-12	0	0	title=Overlapping EnrichrSubstrates" data-content="ATM targets 22 genes from the input gene list.The full list of substrates is available below:RB1 SOX2 KAT5 TRIM28 CHEK2 DAXX PRKDC HIF1A SMARCA4 EZH2 PARP1 ATAD2 TTC5 MEN1 MSH2 SP1 CDK2 HSP90AA1 H2AFX E2F1 VHL RAD51" style="cursor: pointer; text-decoration: underline dotted;">22 substrates
12	MAPK3	4.27E-12	0	0	title=Overlapping EnrichrSubstrates" data-content="MAPK3 targets 24 genes from the input gene list.The full list of substrates is available below:MYC JUN NCOA2 FOS POU5F1 EGFR STAT1 STAT3 MAPKAPK2 FOXO1 SMAD2 SMAD3 ESR1 SP1 CDK2 BCL2 CHUK CEBPA CEBPB E2F4 MAPK3 KLF4 PML STUB1" style="cursor: pointer; text-decoration: underline dotted;">24 substrates
13	ERK2	6.18E-11	0	0	title=Overlapping EnrichrSubstrates" data-content="ERK2 targets 17 genes from the input gene list.The full list of substrates is available below:RB1 MYC MAPKAPK2 SMAD2 JUN SMAD3 ESR1 SP1 BCL2 TCF3 FOS NCOR2 CEBPB EGFR RELA STAT1 STAT3" style="cursor: pointer; text-decoration: underline dotted;">17 substrates
14	HIPK2	1.12E-10	0	0	title=Overlapping EnrichrSubstrates" data-content="HIPK2 targets 16 genes from the input gene list.The full list of substrates is available below:TRIM28 MYC PRKDC HIF1A SMAD2 SMAD3 BCL2 SUMO1 SIN3A CHUK PARP1 SIRT1 NCOR1 CEBPB RELA PML" style="cursor: pointer; text-decoration: underline dotted;">16 substrates
15	CDC2	2.88E-10	0	0	title=Overlapping EnrichrSubstrates" data-content="CDC2 targets 21 genes from the input gene list.The full list of substrates is available below:RB1 CCNH KAT5 TRIM28 MYC SKP2 CSNK2A1 PRKDC JUN EZH2 XPO1 PARP1 FOS NCOR1 RELA ATAD2 STAT3 FOXO1 ZBTB16 BCL2 E2F1" style="cursor: pointer; text-decoration: underline dotted;">21 substrates
16	ABL1	1.48E-09	0	0	title=Overlapping EnrichrSubstrates" data-content="ABL1 targets 16 genes from the input gene list.The full list of substrates is available below:RB1 SPI1 UBE3A KAT5 MYC PRKCD JUN ESR1 BCL2 FOS CEBPB HDAC2 EGFR STAT1 STAT3 RAD51" style="cursor: pointer; text-decoration: underline dotted;">16 substrates
17	CK2ALPHA	4.61E-09	0	0	title=Overlapping EnrichrSubstrates" data-content="CK2ALPHA targets 18 genes from the input gene list.The full list of substrates is available below:RB1 SPI1 CCNH MYC JUN SMARCA4 NCOR2 RELA UBE3A VDR SP1 SUMO1 SIN3A HSP90AA1 ARNT HDAC2 HDAC3 PML" style="cursor: pointer; text-decoration: underline dotted;">18 substrates
18	JNK1	4.78E-09	0	0	title=Overlapping EnrichrSubstrates" data-content="JNK1 targets 12 genes from the input gene list.The full list of substrates is available below:MYC FOS SIRT1 HIF1A RELA E2F1 SMAD2 JUN SMAD3 STAT3 SP1 BCL2" style="cursor: pointer; text-decoration: underline dotted;">12 substrates
19	RPS6KA3	1.33E-08	0	0	title=Overlapping EnrichrSubstrates" data-content="RPS6KA3 targets 19 genes from the input gene list.The full list of substrates is available below:TRRAP MYC PRKDC JUN FOS POU5F1 NCOR2 KAT2B EGFR CDKN2A STAT1 STAT3 MAPKAPK2 SMAD3 ESR1 BCL2 SIN3A CHUK MAPK3" style="cursor: pointer; text-decoration: underline dotted;">19 substrates

20	MAPK8	1.79E-08	0	0	title=Overlapping EnrichrSubstrates" data-content="MAPK8 targets 17 genes from the input gene list.The full list of substrates is available below:SOX2 MYC JUN FOS SIRT1 EGFR CDKN2A STAT1 STAT3 SP1 BCL2 NFE2L2 SIN3A H2AFX MAPK1 KLF4 STUB1" style="cursor: pointer; text-decoration: underline dotted;">17 substrates
21	CSNK2A2	8.15E-08	0	0	title=Overlapping EnrichrSubstrates" data-content="CSNK2A2 targets 13 genes from the input gene list.The full list of substrates is available below:CCNH TRRAP SOX2 MYC JUN CDK2 BCL2 NELFE FOS POU5F1 RPL11 EGFR STAT1" style="cursor: pointer; text-decoration: underline dotted;">13 substrates
22	GSK3BETA	1.29E-07	0	0	title=Overlapping EnrichrSubstrates" data-content="GSK3BETA targets 13 genes from the input gene list.The full list of substrates is available below:MYC DNMT1 FOXO1 PPARGC1A JUN SMAD3 ESR1 BCL2 PIN1 PARP1 CEBPB E2F1 STAT3" style="cursor: pointer; text-decoration: underline dotted;">13 substrates
23	JNK2	4.85E-07	0	0	title=Overlapping EnrichrSubstrates" data-content="JNK2 targets 7 genes from the input gene list.The full list of substrates is available below:RB1 PRKDC RELA MYC SMAD2 JUN FOS" style="cursor: pointer; text-decoration: underline dotted;">7 substrates
24	MAPK11	1.05E-06	0	0	title=Overlapping EnrichrSubstrates" data-content="MAPK11 targets 11 genes from the input gene list.The full list of substrates is available below:MAPKAPK2 PPARGC1A JUN ESR1 BCL2 FOS CEBPB EGFR E2F1 STAT1 PML" style="cursor: pointer; text-decoration: underline dotted;">11 substrates
25	RAF1	2.17E-06	0	0	title=Overlapping EnrichrSubstrates" data-content="RAF1 targets 11 genes from the input gene list.The full list of substrates is available below:RB1 MYC PRKCD ESR1 BCL2 PIN1 HSP90AA1 PARP1 FOS MAPK1 MAPK3" style="cursor: pointer; text-decoration: underline dotted;">11 substrates
26	PRKDC	3.35E-06	0	0	title=Overlapping EnrichrSubstrates" data-content="PRKDC targets 10 genes from the input gene list.The full list of substrates is available below:TRRAP CHEK2 MYC SKP2 PRKDC HSP90AA1 H2AFX EGFR RELA RAD51" style="cursor: pointer; text-decoration: underline dotted;">10 substrates
27	PRKCD	5.12E-06	0	0	title=Overlapping EnrichrSubstrates" data-content="PRKCD targets 11 genes from the input gene list.The full list of substrates is available below:TRIM28 PRKCD PRKDC JUN NFE2L2 FOS HDAC5 MAPK1 MAPK3 STAT1 STAT3" style="cursor: pointer; text-decoration: underline dotted;">11 substrates
28	CDK3	7.27E-06	0	0	title=Overlapping EnrichrSubstrates" data-content="CDK3 targets 6 genes from the input gene list.The full list of substrates is available below:RB1 CCNH E2F1 JUN ESR1 CDK2" style="cursor: pointer; text-decoration: underline dotted;">6 substrates
29	PKBALPHA	7.71E-06	0	0	title=Overlapping EnrichrSubstrates" data-content="PKBALPHA targets 13 genes from the input gene list.The full list of substrates is available below:MYC SKP2 PRKDC PPARGC1A EZH2 NCOR2 RELA FOXO1 SMAD2 SMAD3 ESR1 HSP90AA1 CHUK" style="cursor: pointer; text-decoration: underline dotted;">13 substrates
30	SRC	1.66E-05	0	0	title=Overlapping EnrichrSubstrates" data-content="SRC targets 19 genes from the input gene list.The full list of substrates is available below:RB1 SPI1 MYC PRKCD JUN FOS POU5F1 EGFR STAT1 STAT3 MAPKAPK2 BCL2 CEBPB E2F4 KLF4 CALM1 SMAD3 ESR1 MAPK1" style="cursor: pointer; text-decoration: underline dotted;">19 substrates
31	PRKCA	2.35E-05	0	0	title=Overlapping EnrichrSubstrates" data-content="PRKCA targets 28 genes from the input gene list.The full list of substrates is available below:SPI1 JUN TUBA1A FOS POU5F1 EGFR STAT1 ATAD2 STAT3 MAPKAPK2 NFE2L2 STUB1 RELA VDR TDG RB1 MYC NCOR2 NCOR1 CDKN2A BCL2 CEBPB KLF4 SMAD3 ESR1 PFDN5 NR2C1 MAPK1" style="cursor: pointer; text-decoration: underline dotted;">28 substrates
32	CHUK	2.46E-05	0	0	title=Overlapping EnrichrSubstrates" data-content="CHUK targets 8 genes from the input gene list.The full list of substrates is available below:MYC IKBKE PIAS1 NCOR2 NCOR1 RELA ESR1 CALM1" style="cursor: pointer; text-decoration: underline dotted;">8 substrates

33	CSNK1E	2.7E-05	0	0	title=Overlapping EnrichrSubstrates" data-content="CSNK1E targets 7 genes from the input gene list.The full list of substrates is available below:MYC POU5F1 DNMT1 CDKN2A STAT1 ESR1 SMARCA4" style="cursor: pointer; text-decoration: underline dotted;">7 substrates
34	PRKACA	3.15E-05	0	0	title=Overlapping EnrichrSubstrates" data-content="PRKACA targets 30 genes from the input gene list.The full list of substrates is available below:SPI1 PPARGC1A JUN FOS POU5F1 EGFR STAT3 PSMC5 ZBTB16 PIN1 HSP90AA1 UHRF1 TBX2 RELA UBE3A VDR RB1 MYC HIF1A NCOR2 NCOR1 CDKN2A BCL2 HDAC5 CEBPB HDAC7 KLF4 SMAD3 ESR1 MAPK1" style="cursor: pointer; text-decoration: underline dotted;">30 substrates
35	CHEK1	4.09E-05	0	0	title=Overlapping EnrichrSubstrates" data-content="CHEK1 targets 12 genes from the input gene list.The full list of substrates is available below:RB1 SOX2 TRIM28 MYC SKP2 EZH2 NELFE FOS SIN3A H2AFX E2F1 E2F4" style="cursor: pointer; text-decoration: underline dotted;">12 substrates
36	ATR	5E-05	0	0	title=Overlapping EnrichrSubstrates" data-content="ATR targets 9 genes from the input gene list.The full list of substrates is available below:CHEK2 DAXX MSH2 PRKDC SP1 H2AFX E2F1 CDKN2A PML" style="cursor: pointer; text-decoration: underline dotted;">9 substrates
37	EGFR	5.89E-05	0	0	title=Overlapping EnrichrSubstrates" data-content="EGFR targets 11 genes from the input gene list.The full list of substrates is available below:PRKCD PRKDC JUN ESR1 CALM1 IKBKE HSP90AA1 FOS EGFR STAT1 STAT3" style="cursor: pointer; text-decoration: underline dotted;">11 substrates
38	CDK8	6.18E-05	0	0	title=Overlapping EnrichrSubstrates" data-content="CDK8 targets 5 genes from the input gene list.The full list of substrates is available below:CCNH E2F1 SMAD3 STAT1 STAT3" style="cursor: pointer; text-decoration: underline dotted;">5 substrates
39	MAP3K7	6.86E-05	0	0	title=Overlapping EnrichrSubstrates" data-content="MAP3K7 targets 8 genes from the input gene list.The full list of substrates is available below:SMAD3 SMURF1 CALM1 HSP90AA1 HDAC5 HDAC7 E2F1 E2F4" style="cursor: pointer; text-decoration: underline dotted;">8 substrates
40	CHEK2	7.49E-05	0	0	title=Overlapping EnrichrSubstrates" data-content="CHEK2 targets 6 genes from the input gene list.The full list of substrates is available below:RB1 TRIM28 CHEK2 E2F1 VHL PML" style="cursor: pointer; text-decoration: underline dotted;">6 substrates
41	IKBKE	0.000133	0	0	title=Overlapping EnrichrSubstrates" data-content="IKBKE targets 7 genes from the input gene list.The full list of substrates is available below:IKBKE SKP1 CUL1 RELA STAT1 ESR1 CALM1" style="cursor: pointer; text-decoration: underline dotted;">7 substrates
42	MAPK10	0.000135	0	0	title=Overlapping EnrichrSubstrates" data-content="MAPK10 targets 6 genes from the input gene list.The full list of substrates is available below:MYC FOS EGFR JUN CDKN2A BCL2" style="cursor: pointer; text-decoration: underline dotted;">6 substrates
43	CDK5	0.000241	0	0	title=Overlapping EnrichrSubstrates" data-content="CDK5 targets 10 genes from the input gene list.The full list of substrates is available below:RB1 MYC DNMT1 HIF1A FOXO1 BCL2 NFE2L2 HDAC5 STAT3 STUB1" style="cursor: pointer; text-decoration: underline dotted;">10 substrates
44	DNA	0.000333	0	0	title=Overlapping EnrichrSubstrates" data-content="DNA targets 3 genes from the input gene list.The full list of substrates is available below:HSP90AA1 JUN PRKDC" style="cursor: pointer; text-decoration: underline dotted;">3 substrates
45	CK2	0.000364	0	0	title=Overlapping EnrichrSubstrates" data-content="CK2 targets 6 genes from the input gene list.The full list of substrates is available below:SPI1 HSP90AA1 HDAC2 RELA JUN VDR" style="cursor: pointer; text-decoration: underline dotted;">6 substrates

46	IKKALPHA	0.000385	0	0	title=Overlapping EnrichrSubstrates" data-content="IKKALPHA targets 4 genes from the input gene list.The full list of substrates is available below:HSP90AA1 CHUK RELA NCOR2" style="cursor: pointer; text-decoration: underline dotted;">4 substrates
47	MAP2K1	0.00052	0	0	title=Overlapping EnrichrSubstrates" data-content="MAP2K1 targets 6 genes from the input gene list.The full list of substrates is available below:MYC FOS EGFR MAPK1 MAPK3 STAT3" style="cursor: pointer; text-decoration: underline dotted;">6 substrates
48	TGFBR2	0.00052	0	0	title=Overlapping EnrichrSubstrates" data-content="TGFBR2 targets 6 genes from the input gene list.The full list of substrates is available below:MYC HSP90AA1 SMAD2 SMAD3 PML SP1" style="cursor: pointer; text-decoration: underline dotted;">6 substrates
49	GSK	0.00075	0	0	title=Overlapping EnrichrSubstrates" data-content="GSK targets 4 genes from the input gene list.The full list of substrates is available below:CEBPB RELA MYC ESR1" style="cursor: pointer; text-decoration: underline dotted;">4 substrates
50	GSK3ALPHA	0.000783	0	0	title=Overlapping EnrichrSubstrates" data-content="GSK3ALPHA targets 5 genes from the input gene list.The full list of substrates is available below:MYC PARP1 FOXO1 JUN ESR1" style="cursor: pointer; text-decoration: underline dotted;">5 substrates
51	TBK1	0.000823	0	0	title=Overlapping EnrichrSubstrates" data-content="TBK1 targets 6 genes from the input gene list.The full list of substrates is available below:RPL11 RELA E2F1 STAT3 ESR1 CALM1" style="cursor: pointer; text-decoration: underline dotted;">6 substrates
52	JAK2	0.00085	0	0	title=Overlapping EnrichrSubstrates" data-content="JAK2 targets 5 genes from the input gene list.The full list of substrates is available below:MAPK1 MAPK3 STAT1 STAT3 EZH2" style="cursor: pointer; text-decoration: underline dotted;">5 substrates
53	RPS6KA1	0.000876	0	0	title=Overlapping EnrichrSubstrates" data-content="RPS6KA1 targets 6 genes from the input gene list.The full list of substrates is available below:FOS CEBPB MAPK1 MAPK3 JUN ESR1" style="cursor: pointer; text-decoration: underline dotted;">6 substrates
54	ADRBK1	0.000883	0	0	title=Overlapping EnrichrSubstrates" data-content="ADRBK1 targets 3 genes from the input gene list.The full list of substrates is available below:PRKCD EGFR MAPK3" style="cursor: pointer; text-decoration: underline dotted;">3 substrates
55	PIM1	0.00092	0	0	title=Overlapping EnrichrSubstrates" data-content="PIM1 targets 5 genes from the input gene list.The full list of substrates is available below:MYC SKP2 POU5F1 UHRF1 RELA" style="cursor: pointer; text-decoration: underline dotted;">5 substrates
56	PKCEPSILON	0.000931	0	0	title=Overlapping EnrichrSubstrates" data-content="PKCEPSILON targets 6 genes from the input gene list.The full list of substrates is available below:HSP90AA1 PRKCD MAPK1 MAPK3 SP1 BCL2" style="cursor: pointer; text-decoration: underline dotted;">6 substrates
57	PRKCZ	0.001049	0	0	title=Overlapping EnrichrSubstrates" data-content="PRKCZ targets 6 genes from the input gene list.The full list of substrates is available below:RELA MAPK1 MAPK3 STAT3 SP1 PIN1" style="cursor: pointer; text-decoration: underline dotted;">6 substrates
58	CDK9	0.00145	0	0	title=Overlapping EnrichrSubstrates" data-content="CDK9 targets 4 genes from the input gene list.The full list of substrates is available below:RB1 CCNT1 NCOA2 SMAD3" style="cursor: pointer; text-decoration: underline dotted;">4 substrates
59	IKBKB	0.001473	0	0	title=Overlapping EnrichrSubstrates" data-content="IKBKB targets 6 genes from the input gene list.The full list of substrates is available below:IKBKE PARP1 FOS RELA CDKN2A CALM1" style="cursor: pointer; text-decoration: underline dotted;">6 substrates

60	MAPK9	0.001473	0	0	title=Overlapping EnrichrSubstrates" data-content="MAPK9 targets 6 genes from the input gene list.The full list of substrates is available below:MYC H2AFX FOS EGFR JUN STAT3" style="cursor: pointer; text-decoration: underline dotted;">6 substrates
61	PKD1	0.001653	0	0	title=Overlapping EnrichrSubstrates" data-content="PKD1 targets 5 genes from the input gene list.The full list of substrates is available below:XPO1 HDAC5 EGFR HDAC7 JUN" style="cursor: pointer; text-decoration: underline dotted;">5 substrates
62	CK2A2	0.001768	0	0	title=Overlapping EnrichrSubstrates" data-content="CK2A2 targets 5 genes from the input gene list.The full list of substrates is available below:MYC HSP90AA1 HDAC2 RELA JUN" style="cursor: pointer; text-decoration: underline dotted;">5 substrates
63	NPM/ALK	0.002457	0	0	title=Overlapping EnrichrSubstrates" data-content="NPM/ALK targets 2 genes from the input gene list.The full list of substrates is available below:MSH2 STAT1" style="cursor: pointer; text-decoration: underline dotted;">2 substrates
64	PDHK1	0.002909	0	0	title=Overlapping EnrichrSubstrates" data-content="PDHK1 targets 5 genes from the input gene list.The full list of substrates is available below:HSP90AA1 CHUK PRKCD EGFR FOXO1" style="cursor: pointer; text-decoration: underline dotted;">5 substrates
65	PKR	0.002959	0	0	title=Overlapping EnrichrSubstrates" data-content="PKR targets 4 genes from the input gene list.The full list of substrates is available below:HSP90AA1 CHUK STAT1 STAT3" style="cursor: pointer; text-decoration: underline dotted;">4 substrates
66	TTK	0.002962	0	0	title=Overlapping EnrichrSubstrates" data-content="TTK targets 6 genes from the input gene list.The full list of substrates is available below:CHEK2 CDK2 XPO1 HSP90AA1 E2F1 E2F4" style="cursor: pointer; text-decoration: underline dotted;">6 substrates
67	PRKAA1	0.003391	0	0	title=Overlapping EnrichrSubstrates" data-content="PRKAA1 targets 6 genes from the input gene list.The full list of substrates is available below:RB1 PIAS1 FOXO1 NFE2L2 HDAC5 EGFR" style="cursor: pointer; text-decoration: underline dotted;">6 substrates
68	IKK	0.00341	0	0	title=Overlapping EnrichrSubstrates" data-content="IKK targets 2 genes from the input gene list.The full list of substrates is available below:NCOR2 RELA" style="cursor: pointer; text-decoration: underline dotted;">2 substrates
69	IKKBETA	0.004013	0	0	title=Overlapping EnrichrSubstrates" data-content="IKKBETA targets 4 genes from the input gene list.The full list of substrates is available below:RELA CHUK STAT3 FOS" style="cursor: pointer; text-decoration: underline dotted;">4 substrates
70	ERBB2	0.004034	0	0	title=Overlapping EnrichrSubstrates" data-content="ERBB2 targets 6 genes from the input gene list.The full list of substrates is available below:ESR1 HSP90AA1 NCOR1 EGFR STAT1 STAT3" style="cursor: pointer; text-decoration: underline dotted;">6 substrates
71	BARK1	0.00428	0	0	title=Overlapping EnrichrSubstrates" data-content="BARK1 targets 5 genes from the input gene list.The full list of substrates is available below:PRKCD HDAC5 EGFR MAPK3 PIN1" style="cursor: pointer; text-decoration: underline dotted;">5 substrates
72	PKBBETA	0.004311	0	0	title=Overlapping EnrichrSubstrates" data-content="PKBBETA targets 4 genes from the input gene list.The full list of substrates is available below:FOXO1 RELA CHUK ESR1" style="cursor: pointer; text-decoration: underline dotted;">4 substrates
73	MTOR	0.004387	0	0	title=Overlapping EnrichrSubstrates" data-content="MTOR targets 6 genes from the input gene list.The full list of substrates is available below:EHMT2 MYC DNMT1 JUN ESR1 STAT3" style="cursor: pointer; text-decoration: underline dotted;">6 substrates
74	CHK2	0.004508	0	0	title=Overlapping EnrichrSubstrates" data-content="CHK2 targets 2 genes from the input gene list.The full list of substrates is available below:CHEK2 E2F1" style="cursor: pointer; text-decoration: underline dotted;">2 substrates

75	MAP2K3	0.004624	0	0	title=Overlapping EnrichrSubstrates" data-content="MAP2K3 targets 4 genes from the input gene list.The full list of substrates is available below:MAPK3 SMAD2 SMAD3 SMURF1" style="cursor: pointer; text-decoration: underline dotted;">4 substrates
76	PKCTHETA	0.005654	0	0	title=Overlapping EnrichrSubstrates" data-content="PKCTHETA targets 4 genes from the input gene list.The full list of substrates is available below:CHUK PRKCD MAPK1 MAPK3" style="cursor: pointer; text-decoration: underline dotted;">4 substrates
77	RSK3	0.005746	0	0	title=Overlapping EnrichrSubstrates" data-content="RSK3 targets 2 genes from the input gene list.The full list of substrates is available below:MAPK1 MAPK3" style="cursor: pointer; text-decoration: underline dotted;">2 substrates
78	PKBGAMMA	0.007494	0	0	title=Overlapping EnrichrSubstrates" data-content="PKBGAMMA targets 3 genes from the input gene list.The full list of substrates is available below:FOXO1 RELA CHUK" style="cursor: pointer; text-decoration: underline dotted;">3 substrates
79	MAP3K8	0.01247	0	0	title=Overlapping EnrichrSubstrates" data-content="MAP3K8 targets 4 genes from the input gene list.The full list of substrates is available below:HSP90AA1 MAPK1 MAPK3 CALM1" style="cursor: pointer; text-decoration: underline dotted;">4 substrates
80	RET	0.01513	0	0	title=Overlapping EnrichrSubstrates" data-content="RET targets 3 genes from the input gene list.The full list of substrates is available below:MAPK1 MAPK3 STAT3" style="cursor: pointer; text-decoration: underline dotted;">3 substrates
81	ALK	0.01513	0	0	title=Overlapping EnrichrSubstrates" data-content="ALK targets 3 genes from the input gene list.The full list of substrates is available below:RELA HSP90AA1 STAT3" style="cursor: pointer; text-decoration: underline dotted;">3 substrates
82	PLK3	0.01726	0	0	title=Overlapping EnrichrSubstrates" data-content="PLK3 targets 3 genes from the input gene list.The full list of substrates is available below:HIF1A CHEK2 JUN" style="cursor: pointer; text-decoration: underline dotted;">3 substrates
83	DYRK2	0.01726	0	0	title=Overlapping EnrichrSubstrates" data-content="DYRK2 targets 3 genes from the input gene list.The full list of substrates is available below:MYC JUN STAT3" style="cursor: pointer; text-decoration: underline dotted;">3 substrates
84	PRKD1	0.01804	0	0	title=Overlapping EnrichrSubstrates" data-content="PRKD1 targets 4 genes from the input gene list.The full list of substrates is available below:HDAC5 EGFR HDAC7 JUN" style="cursor: pointer; text-decoration: underline dotted;">4 substrates
85	ACTR2B	0.01805	0	0	title=Overlapping EnrichrSubstrates" data-content="ACTR2B targets 2 genes from the input gene list.The full list of substrates is available below:SMAD2 SMAD3" style="cursor: pointer; text-decoration: underline dotted;">2 substrates
86	PRKAA2	0.01838	0	0	title=Overlapping EnrichrSubstrates" data-content="PRKAA2 targets 3 genes from the input gene list.The full list of substrates is available below:HDAC5 PPARGC1A PARP1" style="cursor: pointer; text-decoration: underline dotted;">3 substrates
87	PDGFRBETA	0.01838	0	0	title=Overlapping EnrichrSubstrates" data-content="PDGFRBETA targets 3 genes from the input gene list.The full list of substrates is available below:EGFR PRKCD STAT3" style="cursor: pointer; text-decoration: underline dotted;">3 substrates
88	RPS6KA5	0.01954	0	0	title=Overlapping EnrichrSubstrates" data-content="RPS6KA5 targets 3 genes from the input gene list.The full list of substrates is available below:RELA TRIM28 STAT3" style="cursor: pointer; text-decoration: underline dotted;">3 substrates
89	CDK7	0.01964	0	0	title=Overlapping EnrichrSubstrates" data-content="CDK7 targets 4 genes from the input gene list.The full list of substrates is available below:CCNH E2F1 ESR1 CDK2" style="cursor: pointer; text-decoration: underline dotted;">4 substrates
90	LYN	0.01977	0	0	title=Overlapping EnrichrSubstrates" data-content="LYN targets 6 genes from the input gene list.The full list of substrates is available below:SPI1 TRIM28 MYC CSNK2A1 PRKCD STAT1" style="cursor: pointer; text-decoration: underline dotted;">6 substrates
91	PAK2	0.02047	0	0	title=Overlapping EnrichrSubstrates" data-content="PAK2 targets 4 genes from the input gene list.The full list of substrates is available below:MYC HDAC5 SMAD2 JUN" style="cursor: pointer; text-decoration: underline dotted;">4 substrates

92	PKCIOTA	0.02326	0	0	title=Overlapping EnrichrSubstrates" data-content="PKCIOTA targets 3 genes from the input gene list.The full list of substrates is available below:RELA HSP90AA1 CHUK" style="cursor: pointer; text-decoration: underline dotted;">3 substrates
93	CSNK1D	0.02458	0	0	title=Overlapping EnrichrSubstrates" data-content="CSNK1D targets 3 genes from the input gene list.The full list of substrates is available below:DNMT1 UHRF1 HIF1A" style="cursor: pointer; text-decoration: underline dotted;">3 substrates
94	NLK	0.02458	0	0	title=Overlapping EnrichrSubstrates" data-content="NLK targets 3 genes from the input gene list.The full list of substrates is available below:FOXO1 SETDB1 STAT3" style="cursor: pointer; text-decoration: underline dotted;">3 substrates
95	LCK	0.02499	0	0	title=Overlapping EnrichrSubstrates" data-content="LCK targets 6 genes from the input gene list.The full list of substrates is available below:SPI1 PRKCD ESR1 MAPK1 MAPK3 STAT1" style="cursor: pointer; text-decoration: underline dotted;">6 substrates
96	CDKN1B	0.02599	0	0	title=Overlapping EnrichrSubstrates" data-content="CDKN1B targets 1 genes from the input gene list.The full list of substrates is available below:CDK2" style="cursor: pointer; text-decoration: underline dotted;">1 substrates
97	BAZ1B	0.02599	0	0	title=Overlapping EnrichrSubstrates" data-content="BAZ1B targets 1 genes from the input gene list.The full list of substrates is available below:H2AFX" style="cursor: pointer; text-decoration: underline dotted;">1 substrates
98	PCNA	0.02599	0	0	title=Overlapping EnrichrSubstrates" data-content="PCNA targets 1 genes from the input gene list.The full list of substrates is available below:CDK2" style="cursor: pointer; text-decoration: underline dotted;">1 substrates
99	EP400	0.02599	0	0	title=Overlapping EnrichrSubstrates" data-content="EP400 targets 1 genes from the input gene list.The full list of substrates is available below:TRRAP" style="cursor: pointer; text-decoration: underline dotted;">1 substrates
100	AURKA	0.0269	0	0	title=Overlapping EnrichrSubstrates" data-content="AURKA targets 4 genes from the input gene list.The full list of substrates is available below:VHL ESR1 SKI PIN1" style="cursor: pointer; text-decoration: underline dotted;">4 substrates
101	GSK3A	0.03178	0	0	title=Overlapping EnrichrSubstrates" data-content="GSK3A targets 3 genes from the input gene list.The full list of substrates is available below:MYC CEBPA CEBPB" style="cursor: pointer; text-decoration: underline dotted;">3 substrates
102	PKN3	0.03302	0	0	title=Overlapping EnrichrSubstrates" data-content="PKN3 targets 2 genes from the input gene list.The full list of substrates is available below:GATA2 EGFR" style="cursor: pointer; text-decoration: underline dotted;">2 substrates
103	PKMYT1	0.03302	0	0	title=Overlapping EnrichrSubstrates" data-content="PKMYT1 targets 2 genes from the input gene list.The full list of substrates is available below:SMARCA4 RAD51" style="cursor: pointer; text-decoration: underline dotted;">2 substrates
104	PLK1	0.03339	0	0	title=Overlapping EnrichrSubstrates" data-content="PLK1 targets 8 genes from the input gene list.The full list of substrates is available below:CHEK2 MYC XPO1 ATAD2 PSMC5 CCNT1 PIN1 RAD51" style="cursor: pointer; text-decoration: underline dotted;">8 substrates
105	PIM3	0.03587	0	0	title=Overlapping EnrichrSubstrates" data-content="PIM3 targets 2 genes from the input gene list.The full list of substrates is available below:PML POU5F1" style="cursor: pointer; text-decoration: underline dotted;">2 substrates
106	HIPK1	0.0388	0	0	title=Overlapping EnrichrSubstrates" data-content="HIPK1 targets 2 genes from the input gene list.The full list of substrates is available below:DAXX KAT6A" style="cursor: pointer; text-decoration: underline dotted;">2 substrates
107	WEE1	0.0388	0	0	title=Overlapping EnrichrSubstrates" data-content="WEE1 targets 2 genes from the input gene list.The full list of substrates is available below:HSP90AA1 CDK2" style="cursor: pointer; text-decoration: underline dotted;">2 substrates
108	PBK	0.04493	0	0	title=Overlapping EnrichrSubstrates" data-content="PBK targets 2 genes from the input gene list.The full list of substrates is available below:E2F4 JUN" style="cursor: pointer; text-decoration: underline dotted;">2 substrates
109	PIM2	0.04811	0	0	title=Overlapping EnrichrSubstrates" data-content="PIM2 targets 2 genes from the input gene list.The full list of substrates is available below:MYC POU5F1" style="cursor: pointer; text-decoration: underline dotted;">2 substrates

110	AKT2	0.0491	0	0	title=Overlapping EnrichrSubstrates" data-content="AKT2 targets 3 genes from the input gene list.The full list of substrates is available below:CHUK PPARGC1A ESR1" style="cursor: pointer; text-decoration: underline dotted;">3 substrates
111	TGFBR1	0.0491	0	0	title=Overlapping EnrichrSubstrates" data-content="TGFBR1 targets 3 genes from the input gene list.The full list of substrates is available below:SMAD2 SMURF1 STAT1" style="cursor: pointer; text-decoration: underline dotted;">3 substrates
112	MAP3K1	0.05103	0	0	title=Overlapping EnrichrSubstrates" data-content="MAP3K1 targets 5 genes from the input gene list.The full list of substrates is available below:ESR1 CALM1 TUBA1A HSP90AA1 STAT3" style="cursor: pointer; text-decoration: underline dotted;">5 substrates
113	MAP3K20	0.05131	0	0	title=Overlapping EnrichrSubstrates" data-content="MAP3K20 targets 1 genes from the input gene list.The full list of substrates is available below:CHEK2" style="cursor: pointer; text-decoration: underline dotted;">1 substrates
114	MOK	0.05138	0	0	title=Overlapping EnrichrSubstrates" data-content="MOK targets 2 genes from the input gene list.The full list of substrates is available below:MYC JUN" style="cursor: pointer; text-decoration: underline dotted;">2 substrates
115	MELK	0.05473	0	0	title=Overlapping EnrichrSubstrates" data-content="MELK targets 2 genes from the input gene list.The full list of substrates is available below:SMAD2 SMAD3" style="cursor: pointer; text-decoration: underline dotted;">2 substrates
116	AURKB	0.05705	0	0	title=Overlapping EnrichrSubstrates" data-content="AURKB targets 5 genes from the input gene list.The full list of substrates is available below:RB1 PRKDC PIN1 NELFE HDAC5" style="cursor: pointer; text-decoration: underline dotted;">5 substrates
117	JAK3	0.06164	0	0	title=Overlapping EnrichrSubstrates" data-content="JAK3 targets 2 genes from the input gene list.The full list of substrates is available below:STAT1 EZH2" style="cursor: pointer; text-decoration: underline dotted;">2 substrates
118	CDK6	0.06164	0	0	title=Overlapping EnrichrSubstrates" data-content="CDK6 targets 2 genes from the input gene list.The full list of substrates is available below:RB1 CEBPA" style="cursor: pointer; text-decoration: underline dotted;">2 substrates
119	UHMK1	0.06164	0	0	title=Overlapping EnrichrSubstrates" data-content="UHMK1 targets 2 genes from the input gene list.The full list of substrates is available below:SMAD2 SMURF1" style="cursor: pointer; text-decoration: underline dotted;">2 substrates
120	CAMK4	0.06164	0	0	title=Overlapping EnrichrSubstrates" data-content="CAMK4 targets 2 genes from the input gene list.The full list of substrates is available below:HDAC5 RELA" style="cursor: pointer; text-decoration: underline dotted;">2 substrates
121	MAPK7	0.06164	0	0	title=Overlapping EnrichrSubstrates" data-content="MAPK7 targets 2 genes from the input gene list.The full list of substrates is available below:FOS PML" style="cursor: pointer; text-decoration: underline dotted;">2 substrates
122	MAPKAPK2	0.06345	0	0	title=Overlapping EnrichrSubstrates" data-content="MAPKAPK2 targets 3 genes from the input gene list.The full list of substrates is available below:TRIM28 PIAS1 MAPKAPK2" style="cursor: pointer; text-decoration: underline dotted;">3 substrates
123	PRKACB	0.06521	0	0	title=Overlapping EnrichrSubstrates" data-content="PRKACB targets 2 genes from the input gene list.The full list of substrates is available below:STAT1 FOS" style="cursor: pointer; text-decoration: underline dotted;">2 substrates
124	RPS6KB1	0.06565	0	0	title=Overlapping EnrichrSubstrates" data-content="RPS6KB1 targets 3 genes from the input gene list.The full list of substrates is available below:PPARGC1A CDKN2A ESR1" style="cursor: pointer; text-decoration: underline dotted;">3 substrates
125	PKN1	0.06884	0	0	title=Overlapping EnrichrSubstrates" data-content="PKN1 targets 2 genes from the input gene list.The full list of substrates is available below:HDAC5 EGFR" style="cursor: pointer; text-decoration: underline dotted;">2 substrates
126	CAMK1	0.06884	0	0	title=Overlapping EnrichrSubstrates" data-content="CAMK1 targets 2 genes from the input gene list.The full list of substrates is available below:HDAC5 HDAC7" style="cursor: pointer; text-decoration: underline dotted;">2 substrates

127	DAPK1	0.07254	0	0	title=Overlapping EnrichrSubstrates" data-content="DAPK1 targets 2 genes from the input gene list.The full list of substrates is available below:MAPK3 PIN1" style="cursor: pointer; text-decoration: underline dotted;">2 substrates
128	PAK1	0.07549	0	0	title=Overlapping EnrichrSubstrates" data-content="PAK1 targets 4 genes from the input gene list.The full list of substrates is available below:PRKCD FOXO1 ESR1 EGFR" style="cursor: pointer; text-decoration: underline dotted;">4 substrates
129	CAM	0.07598	0	0	title=Overlapping EnrichrSubstrates" data-content="CAM targets 1 genes from the input gene list.The full list of substrates is available below:HDAC5" style="cursor: pointer; text-decoration: underline dotted;">1 substrates
130	PKM	0.07598	0	0	title=Overlapping EnrichrSubstrates" data-content="PKM targets 1 genes from the input gene list.The full list of substrates is available below:STAT3" style="cursor: pointer; text-decoration: underline dotted;">1 substrates
131	MAPK13	0.07952	0	0	title=Overlapping EnrichrSubstrates" data-content="MAPK13 targets 3 genes from the input gene list.The full list of substrates is available below:FOS MAPKAPK2 JUN" style="cursor: pointer; text-decoration: underline dotted;">3 substrates
132	CSK	0.08011	0	0	title=Overlapping EnrichrSubstrates" data-content="CSK targets 2 genes from the input gene list.The full list of substrates is available below:EGFR PML" style="cursor: pointer; text-decoration: underline dotted;">2 substrates
133	MAP3K5	0.08399	0	0	title=Overlapping EnrichrSubstrates" data-content="MAP3K5 targets 2 genes from the input gene list.The full list of substrates is available below:IKBKE DAXX" style="cursor: pointer; text-decoration: underline dotted;">2 substrates
134	NIK	0.08808	0	0	title=Overlapping EnrichrSubstrates" data-content="NIK targets 1 genes from the input gene list.The full list of substrates is available below:CEBPB" style="cursor: pointer; text-decoration: underline dotted;">1 substrates
135	HSPB8	0.08808	0	0	title=Overlapping EnrichrSubstrates" data-content="HSPB8 targets 1 genes from the input gene list.The full list of substrates is available below:MAPK3" style="cursor: pointer; text-decoration: underline dotted;">1 substrates
136	CAMK2A	0.08844	0	0	title=Overlapping EnrichrSubstrates" data-content="CAMK2A targets 4 genes from the input gene list.The full list of substrates is available below:MYC SMAD2 NCOR2 HDAC5" style="cursor: pointer; text-decoration: underline dotted;">4 substrates
137	INSR	0.08942	0	0	title=Overlapping EnrichrSubstrates" data-content="INSR targets 3 genes from the input gene list.The full list of substrates is available below:FOS JUN STAT1" style="cursor: pointer; text-decoration: underline dotted;">3 substrates
138	MAP3K14	0.09431	0	0	title=Overlapping EnrichrSubstrates" data-content="MAP3K14 targets 4 genes from the input gene list.The full list of substrates is available below:CALM1 CHUK RPL11 STUB1" style="cursor: pointer; text-decoration: underline dotted;">4 substrates
139	PRKCE	0.09455	0	0	title=Overlapping EnrichrSubstrates" data-content="PRKCE targets 3 genes from the input gene list.The full list of substrates is available below:MAPK3 STAT3 NANOG" style="cursor: pointer; text-decoration: underline dotted;">3 substrates
140	MARK2	0.09596	0	0	title=Overlapping EnrichrSubstrates" data-content="MARK2 targets 2 genes from the input gene list.The full list of substrates is available below:HDAC5 HDAC7" style="cursor: pointer; text-decoration: underline dotted;">2 substrates
141	CSNK1A1	0.09717	0	0	title=Overlapping EnrichrSubstrates" data-content="CSNK1A1 targets 3 genes from the input gene list.The full list of substrates is available below:FOXO1 RELA VHL" style="cursor: pointer; text-decoration: underline dotted;">3 substrates
142	RSK	0.1	0	0	title=Overlapping EnrichrSubstrates" data-content="RSK targets 1 genes from the input gene list.The full list of substrates is available below:CEBPB" style="cursor: pointer; text-decoration: underline dotted;">1 substrates
143	PKCALPHA	0.1003	0	0	title=Overlapping EnrichrSubstrates" data-content="PKCALPHA targets 4 genes from the input gene list.The full list of substrates is available below:MYC PRKCD BCL2 EGFR" style="cursor: pointer; text-decoration: underline dotted;">4 substrates
144	MAPK12	0.1079	0	0	title=Overlapping EnrichrSubstrates" data-content="MAPK12 targets 3 genes from the input gene list.The full list of substrates is available below:FOS MAPKAPK2 JUN" style="cursor: pointer; text-decoration: underline dotted;">3 substrates

145	MAP2K2	0.1084	0	0	title=Overlapping EnrichrSubstrates" data-content="MAP2K2 targets 2 genes from the input gene list.The full list of substrates is available below:MAPK1 MAPK3" style="cursor: pointer; text-decoration: underline dotted;">2 substrates
146	DYRK1A	0.1084	0	0	title=Overlapping EnrichrSubstrates" data-content="DYRK1A targets 2 genes from the input gene list.The full list of substrates is available below:FOXO1 SIRT1" style="cursor: pointer; text-decoration: underline dotted;">2 substrates
147	ABL2	0.1084	0	0	title=Overlapping EnrichrSubstrates" data-content="ABL2 targets 2 genes from the input gene list.The full list of substrates is available below:CEBPB EGFR" style="cursor: pointer; text-decoration: underline dotted;">2 substrates
148	CK1ALPHA	0.1107	0	0	title=Overlapping EnrichrSubstrates" data-content="CK1ALPHA targets 3 genes from the input gene list.The full list of substrates is available below:RB1 FOS JUN" style="cursor: pointer; text-decoration: underline dotted;">3 substrates
149	BCR/ABL	0.1118	0	0	title=Overlapping EnrichrSubstrates" data-content="BCR/ABL targets 1 genes from the input gene list.The full list of substrates is available below:RAD51" style="cursor: pointer; text-decoration: underline dotted;">1 substrates
150	FYN	0.1173	0	0	title=Overlapping EnrichrSubstrates" data-content="FYN targets 5 genes from the input gene list.The full list of substrates is available below:SPI1 PRKCD FOS STAT1 NFE2L2" style="cursor: pointer; text-decoration: underline dotted;">5 substrates
151	RIPK2	0.1212	0	0	title=Overlapping EnrichrSubstrates" data-content="RIPK2 targets 2 genes from the input gene list.The full list of substrates is available below:HSP90AA1 CALM1" style="cursor: pointer; text-decoration: underline dotted;">2 substrates
152	TRRAP	0.1234	0	0	title=Overlapping EnrichrSubstrates" data-content="TRRAP targets 1 genes from the input gene list.The full list of substrates is available below:NCOR2" style="cursor: pointer; text-decoration: underline dotted;">1 substrates
153	RSK	0.1234	0	0	title=Overlapping EnrichrSubstrates" data-content="RSK targets 1 genes from the input gene list.The full list of substrates is available below:FOS" style="cursor: pointer; text-decoration: underline dotted;">1 substrates
154	CK1GAMMA1	0.1234	0	0	title=Overlapping EnrichrSubstrates" data-content="CK1GAMMA1 targets 1 genes from the input gene list.The full list of substrates is available below:FOXO1" style="cursor: pointer; text-decoration: underline dotted;">1 substrates
155	PTK2	0.1299	0	0	title=Overlapping EnrichrSubstrates" data-content="PTK2 targets 2 genes from the input gene list.The full list of substrates is available below:SUMO1 NANOG" style="cursor: pointer; text-decoration: underline dotted;">2 substrates
156	PKC	0.1349	0	0	title=Overlapping EnrichrSubstrates" data-content="PKC targets 1 genes from the input gene list.The full list of substrates is available below:RELA" style="cursor: pointer; text-decoration: underline dotted;">1 substrates
157	CAMKIV	0.1349	0	0	title=Overlapping EnrichrSubstrates" data-content="CAMKIV targets 1 genes from the input gene list.The full list of substrates is available below:RELA" style="cursor: pointer; text-decoration: underline dotted;">1 substrates
158	CK1	0.1349	0	0	title=Overlapping EnrichrSubstrates" data-content="CK1 targets 1 genes from the input gene list.The full list of substrates is available below:FOXO1" style="cursor: pointer; text-decoration: underline dotted;">1 substrates
159	PRKCI	0.1388	0	0	title=Overlapping EnrichrSubstrates" data-content="PRKCI targets 2 genes from the input gene list.The full list of substrates is available below:SP1 EZH2" style="cursor: pointer; text-decoration: underline dotted;">2 substrates
160	JNK3	0.1462	0	0	title=Overlapping EnrichrSubstrates" data-content="JNK3 targets 1 genes from the input gene list.The full list of substrates is available below:JUN" style="cursor: pointer; text-decoration: underline dotted;">1 substrates
161	TRKB	0.1574	0	0	title=Overlapping EnrichrSubstrates" data-content="TRKB targets 1 genes from the input gene list.The full list of substrates is available below:FOS" style="cursor: pointer; text-decoration: underline dotted;">1 substrates
162	BRD2	0.1574	0	0	title=Overlapping EnrichrSubstrates" data-content="BRD2 targets 1 genes from the input gene list.The full list of substrates is available below:RB1" style="cursor: pointer; text-decoration: underline dotted;">1 substrates
163	IGF1R	0.1662	0	0	title=Overlapping EnrichrSubstrates" data-content="IGF1R targets 2 genes from the input gene list.The full list of substrates is available below:FOS EGFR" style="cursor: pointer; text-decoration: underline dotted;">2 substrates
164	CAMKIIBETA	0.1662	0	0	title=Overlapping EnrichrSubstrates" data-content="CAMKIIBETA targets 2 genes from the input gene list.The full list of substrates is available below:FOS PPARGC1A" style="cursor: pointer; text-decoration: underline dotted;">2 substrates

165	ABL	0.1802	0	0 title=Overlapping EnrichrSubstrates" data-content="ABL targets 2 genes from the input gene list.The full list of substrates is available below:RAD51 CDK2" style="cursor: pointer; text-decoration: underline dotted;">2 substrates
166	RIPK3	0.1871	0	0 title=Overlapping EnrichrSubstrates" data-content="RIPK3 targets 3 genes from the input gene list.The full list of substrates is available below:DAXX CALM1 HSP90AA1" style="cursor: pointer; text-decoration: underline dotted;">3 substrates
167	PKC	0.1901	0	0 title=Overlapping EnrichrSubstrates" data-content="PKC targets 1 genes from the input gene list.The full list of substrates is available below:STAT3" style="cursor: pointer; text-decoration: underline dotted;">1 substrates
168	PKAALPHA	0.1938	0	0 title=Overlapping EnrichrSubstrates" data-content="PKAALPHA targets 3 genes from the input gene list.The full list of substrates is available below:ESR1 FOS RELA" style="cursor: pointer; text-decoration: underline dotted;">3 substrates
169	CAMKIIALPHA	0.1944	0	0 title=Overlapping EnrichrSubstrates" data-content="CAMKIIALPHA targets 2 genes from the input gene list.The full list of substrates is available below:CEBPB EGFR" style="cursor: pointer; text-decoration: underline dotted;">2 substrates
170	SGK1	0.2184	0	0 title=Overlapping EnrichrSubstrates" data-content="SGK1 targets 2 genes from the input gene list.The full list of substrates is available below:FOXO1 MAPK1" style="cursor: pointer; text-decoration: underline dotted;">2 substrates
171	TAOK3	0.2215	0	0 title=Overlapping EnrichrSubstrates" data-content="TAOK3 targets 1 genes from the input gene list.The full list of substrates is available below:ZNF281" style="cursor: pointer; text-decoration: underline dotted;">1 substrates
172	RPS6KA6	0.2215	0	0 title=Overlapping EnrichrSubstrates" data-content="RPS6KA6 targets 1 genes from the input gene list.The full list of substrates is available below:XPO1" style="cursor: pointer; text-decoration: underline dotted;">1 substrates
173	ALPK1	0.2215	0	0 title=Overlapping EnrichrSubstrates" data-content="ALPK1 targets 1 genes from the input gene list.The full list of substrates is available below:IKBKE" style="cursor: pointer; text-decoration: underline dotted;">1 substrates
174	LMTK3	0.2215	0	0 title=Overlapping EnrichrSubstrates" data-content="LMTK3 targets 1 genes from the input gene list.The full list of substrates is available below:ZBTB16" style="cursor: pointer; text-decoration: underline dotted;">1 substrates
175	PRKCQ	0.2232	0	0 title=Overlapping EnrichrSubstrates" data-content="PRKCQ targets 2 genes from the input gene list.The full list of substrates is available below:SPI1 CHUK" style="cursor: pointer; text-decoration: underline dotted;">2 substrates
176	BTK	0.228	0	0 title=Overlapping EnrichrSubstrates" data-content="BTK targets 2 genes from the input gene list.The full list of substrates is available below:FOS JUN" style="cursor: pointer; text-decoration: underline dotted;">2 substrates
177	PDK	0.2317	0	0 title=Overlapping EnrichrSubstrates" data-content="PDK targets 1 genes from the input gene list.The full list of substrates is available below:PRKCD" style="cursor: pointer; text-decoration: underline dotted;">1 substrates
178	CDK13	0.2317	0	0 title=Overlapping EnrichrSubstrates" data-content="CDK13 targets 1 genes from the input gene list.The full list of substrates is available below:KAT6A" style="cursor: pointer; text-decoration: underline dotted;">1 substrates
179	CDKL2	0.2317	0	0 title=Overlapping EnrichrSubstrates" data-content="CDKL2 targets 1 genes from the input gene list.The full list of substrates is available below:CALM1" style="cursor: pointer; text-decoration: underline dotted;">1 substrates
180	ROR1	0.2317	0	0 title=Overlapping EnrichrSubstrates" data-content="ROR1 targets 1 genes from the input gene list.The full list of substrates is available below:ZNF281" style="cursor: pointer; text-decoration: underline dotted;">1 substrates
181	ULK4	0.2317	0	0 title=Overlapping EnrichrSubstrates" data-content="ULK4 targets 1 genes from the input gene list.The full list of substrates is available below:SMURF1" style="cursor: pointer; text-decoration: underline dotted;">1 substrates
182	PKCETA	0.2317	0	0 title=Overlapping EnrichrSubstrates" data-content="PKCETA targets 1 genes from the input gene list.The full list of substrates is available below:PRKCD" style="cursor: pointer; text-decoration: underline dotted;">1 substrates
183	MINK1	0.2317	0	0 title=Overlapping EnrichrSubstrates" data-content="MINK1 targets 1 genes from the input gene list.The full list of substrates is available below:SMAD2" style="cursor: pointer; text-decoration: underline dotted;">1 substrates
184	STK40	0.2317	0	0 title=Overlapping EnrichrSubstrates" data-content="STK40 targets 1 genes from the input gene list.The full list of substrates is available below:MAPKAPK2" style="cursor: pointer; text-decoration: underline dotted;">1 substrates

185	MAP3K3	0.2372	0	0	title=Overlapping EnrichrSubstrates" data-content="MAP3K3 targets 4 genes from the input gene list.The full list of substrates is available below:CALM1 TUBA1A PFDN5 STUB1" style="cursor: pointer; text-decoration: underline dotted;">4 substrates
186	CDK20	0.2418	0	0	title=Overlapping EnrichrSubstrates" data-content="CDK20 targets 1 genes from the input gene list.The full list of substrates is available below:CDK2" style="cursor: pointer; text-decoration: underline dotted;">1 substrates
187	DMPK	0.2418	0	0	title=Overlapping EnrichrSubstrates" data-content="DMPK targets 1 genes from the input gene list.The full list of substrates is available below:SP1" style="cursor: pointer; text-decoration: underline dotted;">1 substrates
188	PKA	0.2418	0	0	title=Overlapping EnrichrSubstrates" data-content="PKA targets 1 genes from the input gene list.The full list of substrates is available below:RELA" style="cursor: pointer; text-decoration: underline dotted;">1 substrates
189	NTRK3	0.2418	0	0	title=Overlapping EnrichrSubstrates" data-content="NTRK3 targets 1 genes from the input gene list.The full list of substrates is available below:FOS" style="cursor: pointer; text-decoration: underline dotted;">1 substrates
190	CSNK2B	0.2418	0	0	title=Overlapping EnrichrSubstrates" data-content="CSNK2B targets 1 genes from the input gene list.The full list of substrates is available below:FOS" style="cursor: pointer; text-decoration: underline dotted;">1 substrates
191	MARK4	0.2418	0	0	title=Overlapping EnrichrSubstrates" data-content="MARK4 targets 1 genes from the input gene list.The full list of substrates is available below:SMARCA4" style="cursor: pointer; text-decoration: underline dotted;">1 substrates
192	PRKD3	0.2518	0	0	title=Overlapping EnrichrSubstrates" data-content="PRKD3 targets 1 genes from the input gene list.The full list of substrates is available below:HDAC5" style="cursor: pointer; text-decoration: underline dotted;">1 substrates
193	MAP3K4	0.2518	0	0	title=Overlapping EnrichrSubstrates" data-content="MAP3K4 targets 1 genes from the input gene list.The full list of substrates is available below:TRRAP" style="cursor: pointer; text-decoration: underline dotted;">1 substrates
194	CAM	0.2518	0	0	title=Overlapping EnrichrSubstrates" data-content="CAM targets 1 genes from the input gene list.The full list of substrates is available below:EGFR" style="cursor: pointer; text-decoration: underline dotted;">1 substrates
195	PNCK	0.2518	0	0	title=Overlapping EnrichrSubstrates" data-content="PNCK targets 1 genes from the input gene list.The full list of substrates is available below:HSP90AA1" style="cursor: pointer; text-decoration: underline dotted;">1 substrates
196	MAPK15	0.2518	0	0	title=Overlapping EnrichrSubstrates" data-content="MAPK15 targets 1 genes from the input gene list.The full list of substrates is available below:JUN" style="cursor: pointer; text-decoration: underline dotted;">1 substrates
197	MAP3K10	0.2616	0	0	title=Overlapping EnrichrSubstrates" data-content="MAP3K10 targets 1 genes from the input gene list.The full list of substrates is available below:TCF3" style="cursor: pointer; text-decoration: underline dotted;">1 substrates
198	SIK1	0.2616	0	0	title=Overlapping EnrichrSubstrates" data-content="SIK1 targets 1 genes from the input gene list.The full list of substrates is available below:NCOR2" style="cursor: pointer; text-decoration: underline dotted;">1 substrates
199	MAP4K4	0.2616	0	0	title=Overlapping EnrichrSubstrates" data-content="MAP4K4 targets 1 genes from the input gene list.The full list of substrates is available below:SMARCA4" style="cursor: pointer; text-decoration: underline dotted;">1 substrates
200	CDK16	0.2713	0	0	title=Overlapping EnrichrSubstrates" data-content="CDK16 targets 1 genes from the input gene list.The full list of substrates is available below:SMARCA4" style="cursor: pointer; text-decoration: underline dotted;">1 substrates
201	FRK	0.2713	0	0	title=Overlapping EnrichrSubstrates" data-content="FRK targets 1 genes from the input gene list.The full list of substrates is available below:FOS" style="cursor: pointer; text-decoration: underline dotted;">1 substrates
202	CSNK1G2	0.2713	0	0	title=Overlapping EnrichrSubstrates" data-content="CSNK1G2 targets 1 genes from the input gene list.The full list of substrates is available below:EHMT2" style="cursor: pointer; text-decoration: underline dotted;">1 substrates
203	CSNK1G1	0.2713	0	0	title=Overlapping EnrichrSubstrates" data-content="CSNK1G1 targets 1 genes from the input gene list.The full list of substrates is available below:RELA" style="cursor: pointer; text-decoration: underline dotted;">1 substrates

204	MAP3K11	0.2713	0	0	title=Overlapping EnrichrSubstrates" data-content="MAP3K11 targets 1 genes from the input gene list.The full list of substrates is available below:PIN1" style="cursor: pointer; text-decoration: underline dotted;">1 substrates
205	MERTK	0.2713	0	0	title=Overlapping EnrichrSubstrates" data-content="MERTK targets 1 genes from the input gene list.The full list of substrates is available below:STAT3" style="cursor: pointer; text-decoration: underline dotted;">1 substrates
206	DYRK3	0.2713	0	0	title=Overlapping EnrichrSubstrates" data-content="DYRK3 targets 1 genes from the input gene list.The full list of substrates is available below:SIRT1" style="cursor: pointer; text-decoration: underline dotted;">1 substrates
207	CAMK2G	0.2713	0	0	title=Overlapping EnrichrSubstrates" data-content="CAMK2G targets 1 genes from the input gene list.The full list of substrates is available below:STAT1" style="cursor: pointer; text-decoration: underline dotted;">1 substrates
208	AURORAB	0.2766	0	0	title=Overlapping EnrichrSubstrates" data-content="AURORAB targets 2 genes from the input gene list.The full list of substrates is available below:RB1 XPO1" style="cursor: pointer; text-decoration: underline dotted;">2 substrates
209	RPS6KA4	0.2808	0	0	title=Overlapping EnrichrSubstrates" data-content="RPS6KA4 targets 1 genes from the input gene list.The full list of substrates is available below:RELA" style="cursor: pointer; text-decoration: underline dotted;">1 substrates
210	YES	0.2808	0	0	title=Overlapping EnrichrSubstrates" data-content="YES targets 1 genes from the input gene list.The full list of substrates is available below:EGFR" style="cursor: pointer; text-decoration: underline dotted;">1 substrates
211	PAK5	0.2808	0	0	title=Overlapping EnrichrSubstrates" data-content="PAK5 targets 1 genes from the input gene list.The full list of substrates is available below:TCF3" style="cursor: pointer; text-decoration: underline dotted;">1 substrates
212	MYLK	0.2903	0	0	title=Overlapping EnrichrSubstrates" data-content="MYLK targets 1 genes from the input gene list.The full list of substrates is available below:MAPK3" style="cursor: pointer; text-decoration: underline dotted;">1 substrates
213	NEK1	0.2903	0	0	title=Overlapping EnrichrSubstrates" data-content="NEK1 targets 1 genes from the input gene list.The full list of substrates is available below:VHL" style="cursor: pointer; text-decoration: underline dotted;">1 substrates
214	EPHB3	0.2903	0	0	title=Overlapping EnrichrSubstrates" data-content="EPHB3 targets 1 genes from the input gene list.The full list of substrates is available below:SMARCA4" style="cursor: pointer; text-decoration: underline dotted;">1 substrates
215	EPHA8	0.2903	0	0	title=Overlapping EnrichrSubstrates" data-content="EPHA8 targets 1 genes from the input gene list.The full list of substrates is available below:SMARCA4" style="cursor: pointer; text-decoration: underline dotted;">1 substrates
216	PKC	0.2912	0	0	title=Overlapping EnrichrSubstrates" data-content="PKC targets 2 genes from the input gene list.The full list of substrates is available below:EGFR NFE2L2" style="cursor: pointer; text-decoration: underline dotted;">2 substrates
217	PRKCB	0.298	0	0	title=Overlapping EnrichrSubstrates" data-content="PRKCB targets 4 genes from the input gene list.The full list of substrates is available below:JUN STAT3 ESR1 BCL2" style="cursor: pointer; text-decoration: underline dotted;">4 substrates
218	VRK1	0.2996	0	0	title=Overlapping EnrichrSubstrates" data-content="VRK1 targets 1 genes from the input gene list.The full list of substrates is available below:JUN" style="cursor: pointer; text-decoration: underline dotted;">1 substrates
219	RPS6KA2	0.2996	0	0	title=Overlapping EnrichrSubstrates" data-content="RPS6KA2 targets 1 genes from the input gene list.The full list of substrates is available below:MAPK3" style="cursor: pointer; text-decoration: underline dotted;">1 substrates
220	MKNK1	0.2996	0	0	title=Overlapping EnrichrSubstrates" data-content="MKNK1 targets 1 genes from the input gene list.The full list of substrates is available below:MAPK3" style="cursor: pointer; text-decoration: underline dotted;">1 substrates
221	AURORAA	0.3009	0	0	title=Overlapping EnrichrSubstrates" data-content="AURORAA targets 2 genes from the input gene list.The full list of substrates is available below:NCOR1 HDAC3" style="cursor: pointer; text-decoration: underline dotted;">2 substrates
222	CAMKIIGAMMA	0.3088	0	0	title=Overlapping EnrichrSubstrates" data-content="CAMKIIGAMMA targets 1 genes from the input gene list.The full list of substrates is available below:EGFR" style="cursor: pointer; text-decoration: underline dotted;">1 substrates
223	ACVR1	0.3088	0	0	title=Overlapping EnrichrSubstrates" data-content="ACVR1 targets 1 genes from the input gene list.The full list of substrates is available below:STAT1" style="cursor: pointer; text-decoration: underline dotted;">1 substrates

224	CK1EPSILON	0.3088	0	0	title=Overlapping EnrichrSubstrates" data-content="CK1EPSILON targets 1 genes from the input gene list.The full list of substrates is available below:FOXO1" style="cursor: pointer; text-decoration: underline dotted;">1 substrates
225	FGFR3	0.3088	0	0	title=Overlapping EnrichrSubstrates" data-content="FGFR3 targets 1 genes from the input gene list.The full list of substrates is available below:STAT1" style="cursor: pointer; text-decoration: underline dotted;">1 substrates
226	AKT3	0.3178	0	0	title=Overlapping EnrichrSubstrates" data-content="AKT3 targets 1 genes from the input gene list.The full list of substrates is available below:PRKCD" style="cursor: pointer; text-decoration: underline dotted;">1 substrates
227	IRAK1	0.3178	0	0	title=Overlapping EnrichrSubstrates" data-content="IRAK1 targets 1 genes from the input gene list.The full list of substrates is available below:STAT3" style="cursor: pointer; text-decoration: underline dotted;">1 substrates
228	DYRK1B	0.3268	0	0	title=Overlapping EnrichrSubstrates" data-content="DYRK1B targets 1 genes from the input gene list.The full list of substrates is available below:HDAC5" style="cursor: pointer; text-decoration: underline dotted;">1 substrates
229	LKB1	0.3268	0	0	title=Overlapping EnrichrSubstrates" data-content="LKB1 targets 1 genes from the input gene list.The full list of substrates is available below:PPARGC1A" style="cursor: pointer; text-decoration: underline dotted;">1 substrates
230	PTK6	0.3268	0	0	title=Overlapping EnrichrSubstrates" data-content="PTK6 targets 1 genes from the input gene list.The full list of substrates is available below:EGFR" style="cursor: pointer; text-decoration: underline dotted;">1 substrates
231	GRK2	0.3268	0	0	title=Overlapping EnrichrSubstrates" data-content="GRK2 targets 1 genes from the input gene list.The full list of substrates is available below:SMAD2" style="cursor: pointer; text-decoration: underline dotted;">1 substrates
232	JAK1	0.3356	0	0	title=Overlapping EnrichrSubstrates" data-content="JAK1 targets 1 genes from the input gene list.The full list of substrates is available below:STAT1" style="cursor: pointer; text-decoration: underline dotted;">1 substrates
233	EEF2K	0.3356	0	0	title=Overlapping EnrichrSubstrates" data-content="EEF2K targets 1 genes from the input gene list.The full list of substrates is available below:MAPKAPK2" style="cursor: pointer; text-decoration: underline dotted;">1 substrates
234	NEK6	0.3444	0	0	title=Overlapping EnrichrSubstrates" data-content="NEK6 targets 1 genes from the input gene list.The full list of substrates is available below:STAT3" style="cursor: pointer; text-decoration: underline dotted;">1 substrates
235	AXL	0.3444	0	0	title=Overlapping EnrichrSubstrates" data-content="AXL targets 1 genes from the input gene list.The full list of substrates is available below:STAT3" style="cursor: pointer; text-decoration: underline dotted;">1 substrates
236	FER	0.3444	0	0	title=Overlapping EnrichrSubstrates" data-content="FER targets 1 genes from the input gene list.The full list of substrates is available below:STAT3" style="cursor: pointer; text-decoration: underline dotted;">1 substrates
237	EIF2AK2	0.353	0	0	title=Overlapping EnrichrSubstrates" data-content="EIF2AK2 targets 1 genes from the input gene list.The full list of substrates is available below:CHUK" style="cursor: pointer; text-decoration: underline dotted;">1 substrates
238	YES1	0.353	0	0	title=Overlapping EnrichrSubstrates" data-content="YES1 targets 1 genes from the input gene list.The full list of substrates is available below:PRKCD" style="cursor: pointer; text-decoration: underline dotted;">1 substrates
239	EPHB1	0.3615	0	0	title=Overlapping EnrichrSubstrates" data-content="EPHB1 targets 1 genes from the input gene list.The full list of substrates is available below:STAT3" style="cursor: pointer; text-decoration: underline dotted;">1 substrates
240	STK4	0.3615	0	0	title=Overlapping EnrichrSubstrates" data-content="STK4 targets 1 genes from the input gene list.The full list of substrates is available below:H2AFX" style="cursor: pointer; text-decoration: underline dotted;">1 substrates
241	BMPR1B	0.3615	0	0	title=Overlapping EnrichrSubstrates" data-content="BMPR1B targets 1 genes from the input gene list.The full list of substrates is available below:SMAD2" style="cursor: pointer; text-decoration: underline dotted;">1 substrates
242	PDGFRB	0.3698	0	0	title=Overlapping EnrichrSubstrates" data-content="PDGFRB targets 1 genes from the input gene list.The full list of substrates is available below:PRKCD" style="cursor: pointer; text-decoration: underline dotted;">1 substrates
243	PKG1CGKI	0.3863	0	0	title=Overlapping EnrichrSubstrates" data-content="PKG1CGKI targets 1 genes from the input gene list.The full list of substrates is available below:SP1" style="cursor: pointer; text-decoration: underline dotted;">1 substrates

244	ARAF	0.4023	0	0	title=Overlapping EnrichrSubstrates" data-content="ARAF targets 1 genes from the input gene list.The full list of substrates is available below:PRKCD" style="cursor: pointer; text-decoration: underline dotted;">1 substrates
245	BLK	0.4102	0	0	title=Overlapping EnrichrSubstrates" data-content="BLK targets 1 genes from the input gene list.The full list of substrates is available below:EGFR" style="cursor: pointer; text-decoration: underline dotted;">1 substrates
246	HCK	0.4179	0	0	title=Overlapping EnrichrSubstrates" data-content="HCK targets 1 genes from the input gene list.The full list of substrates is available below:SPI1" style="cursor: pointer; text-decoration: underline dotted;">1 substrates
247	KIT	0.4179	0	0	title=Overlapping EnrichrSubstrates" data-content="KIT targets 1 genes from the input gene list.The full list of substrates is available below:STAT3" style="cursor: pointer; text-decoration: underline dotted;">1 substrates
248	RIPK1	0.4331	0	0	title=Overlapping EnrichrSubstrates" data-content="RIPK1 targets 1 genes from the input gene list.The full list of substrates is available below:PML" style="cursor: pointer; text-decoration: underline dotted;">1 substrates
249	MET	0.4406	0	0	title=Overlapping EnrichrSubstrates" data-content="MET targets 1 genes from the input gene list.The full list of substrates is available below:PARP1" style="cursor: pointer; text-decoration: underline dotted;">1 substrates
250	PRKD2	0.4552	0	0	title=Overlapping EnrichrSubstrates" data-content="PRKD2 targets 1 genes from the input gene list.The full list of substrates is available below:HDAC5" style="cursor: pointer; text-decoration: underline dotted;">1 substrates
251	PRKCG	0.4694	0	0	title=Overlapping EnrichrSubstrates" data-content="PRKCG targets 1 genes from the input gene list.The full list of substrates is available below:HSP90AA1" style="cursor: pointer; text-decoration: underline dotted;">1 substrates
252	PAK4	0.4764	0	0	title=Overlapping EnrichrSubstrates" data-content="PAK4 targets 1 genes from the input gene list.The full list of substrates is available below:ESR1" style="cursor: pointer; text-decoration: underline dotted;">1 substrates
253	MAP2K4	0.4968	0	0	title=Overlapping EnrichrSubstrates" data-content="MAP2K4 targets 1 genes from the input gene list.The full list of substrates is available below:DAXX" style="cursor: pointer; text-decoration: underline dotted;">1 substrates
254	FGR	0.4968	0	0	title=Overlapping EnrichrSubstrates" data-content="FGR targets 1 genes from the input gene list.The full list of substrates is available below:HSP90AA1" style="cursor: pointer; text-decoration: underline dotted;">1 substrates
255	CK1DELTA	0.5164	0	0	title=Overlapping EnrichrSubstrates" data-content="CK1DELTA targets 1 genes from the input gene list.The full list of substrates is available below:FOXO1" style="cursor: pointer; text-decoration: underline dotted;">1 substrates
256	PKAGAMMA	0.5414	0	0	title=Overlapping EnrichrSubstrates" data-content="PKAGAMMA targets 1 genes from the input gene list.The full list of substrates is available below:RELA" style="cursor: pointer; text-decoration: underline dotted;">1 substrates
257	ERBB3	0.5414	0	0	title=Overlapping EnrichrSubstrates" data-content="ERBB3 targets 1 genes from the input gene list.The full list of substrates is available below:EGFR" style="cursor: pointer; text-decoration: underline dotted;">1 substrates
258	SYK	0.6141	0	0	title=Overlapping EnrichrSubstrates" data-content="SYK targets 1 genes from the input gene list.The full list of substrates is available below:TUBA1A" style="cursor: pointer; text-decoration: underline dotted;">1 substrates
259	TYK2	0.6242	0	0	title=Overlapping EnrichrSubstrates" data-content="TYK2 targets 1 genes from the input gene list.The full list of substrates is available below:STAT3" style="cursor: pointer; text-decoration: underline dotted;">1 substrates
260	PKCGAMMA	0.6242	0	0	title=Overlapping EnrichrSubstrates" data-content="PKCGAMMA targets 1 genes from the input gene list.The full list of substrates is available below:PRKCD" style="cursor: pointer; text-decoration: underline dotted;">1 substrates

Supplementary Table 5: Protein-protein interaction (PPI) sub-network around proteins encoded by the co-downregulated genes in CRC vs the normal mucosa.

G2N

Source	Source type	Target	Target type
VDR	Intermediate protein	VDR	Intermediate protein
VDR	Intermediate protein	JUN	Intermediate protein
VDR	Intermediate protein	SIRT1	Intermediate protein
VDR	Intermediate protein	NCOR1	Intermediate protein
VDR	Intermediate protein	RB1	Intermediate protein
VDR	Intermediate protein	MYC	Seed protein
VDR	Intermediate protein	SMAD3	Intermediate protein
VDR	Intermediate protein	FOS	Intermediate protein
VDR	Intermediate protein	TAF7	Seed protein
VDR	Intermediate protein	TCF3	Seed protein
VDR	Intermediate protein	CEBPA	Intermediate protein
JUN	Intermediate protein	GTF2F1	Intermediate protein
JUN	Intermediate protein	PML	Seed protein
JUN	Intermediate protein	ATF2	Seed protein
JUN	Intermediate protein	RB1	Intermediate protein
JUN	Intermediate protein	RELA	Intermediate protein
JUN	Intermediate protein	MYC	Seed protein

JUN	Intermediate protein	SMAD3	Intermediate protein
JUN	Intermediate protein	SMAD2	Intermediate protein
JUN	Intermediate protein	PIAS2	Intermediate protein
JUN	Intermediate protein	PIN1	Intermediate protein
JUN	Intermediate protein	SPI1	Intermediate protein
JUN	Intermediate protein	PRKDC	Intermediate protein
JUN	Intermediate protein	HIF1A	Intermediate protein
JUN	Intermediate protein	GATA2	Intermediate protein
JUN	Intermediate protein	VDR	Intermediate protein
JUN	Intermediate protein	JUN	Intermediate protein
JUN	Intermediate protein	SIRT1	Intermediate protein
JUN	Intermediate protein	SP1	Intermediate protein
JUN	Intermediate protein	NCOR1	Intermediate protein
JUN	Intermediate protein	KAT2B	Intermediate protein
JUN	Intermediate protein	TBP	Intermediate protein
JUN	Intermediate protein	CSNK2A1	Intermediate protein
JUN	Intermediate protein	FOS	Intermediate protein
JUN	Intermediate protein	TAF1	Seed protein
JUN	Intermediate protein	TAF4	Intermediate protein
JUN	Intermediate protein	HDAC3	Intermediate protein

JUN	Intermediate protein	MAPK8	Intermediate protein
JUN	Intermediate protein	MAPK3	Intermediate protein
JUN	Intermediate protein	MAPK1	Intermediate protein
USP22	Intermediate protein	TRRAP	Intermediate protein
USP22	Intermediate protein	SIRT1	Intermediate protein
USP22	Intermediate protein	KAT2A	Seed protein
USP22	Intermediate protein	MYC	Seed protein
USP22	Intermediate protein	TAF9B	Intermediate protein
ZBTB17	Intermediate protein	MAX	Seed protein
ZBTB17	Intermediate protein	CDKN2A	Intermediate protein
ZBTB17	Intermediate protein	ZBTB17	Intermediate protein
ZBTB17	Intermediate protein	MYC	Seed protein
TUBB	Intermediate protein	SIRT2	Intermediate protein
TUBB	Intermediate protein	KAT2A	Seed protein
TUBB	Intermediate protein	MYC	Seed protein
MSH2	Intermediate protein	MSH2	Intermediate protein
MSH2	Intermediate protein	MAX	Seed protein
MSH2	Intermediate protein	MYC	Seed protein
GTF2F1	Intermediate protein	TBP	Intermediate protein
GTF2F1	Intermediate protein	JUN	Intermediate protein

GTF2F1	Intermediate protein	FOS	Intermediate protein
GTF2F1	Intermediate protein	TAF1	Seed protein
GTF2F1	Intermediate protein	HNRNPU	Intermediate protein
GTF2F1	Intermediate protein	MYC	Seed protein
SIRT1	Intermediate protein	PML	Seed protein
SIRT1	Intermediate protein	RELA	Intermediate protein
SIRT1	Intermediate protein	RB1	Intermediate protein
SIRT1	Intermediate protein	MYC	Seed protein
SIRT1	Intermediate protein	PPARGC1A	Intermediate protein
SIRT1	Intermediate protein	HIST1H3A	Intermediate protein
SIRT1	Intermediate protein	KAT5	Intermediate protein
SIRT1	Intermediate protein	VDR	Intermediate protein
SIRT1	Intermediate protein	JUN	Intermediate protein
SIRT1	Intermediate protein	USP22	Intermediate protein
SIRT1	Intermediate protein	SIRT1	Intermediate protein
SIRT1	Intermediate protein	SIRT2	Intermediate protein
SIRT1	Intermediate protein	KAT2B	Intermediate protein
SIRT1	Intermediate protein	NCOR1	Intermediate protein
SIRT1	Intermediate protein	KAT2A	Seed protein
SIRT1	Intermediate protein	CSNK2A1	Intermediate protein

SIRT1	Intermediate protein	FOS	Intermediate protein
SIRT1	Intermediate protein	SETD7	Intermediate protein
SIRT1	Intermediate protein	MAPK8	Intermediate protein
SIRT1	Intermediate protein	VHL	Intermediate protein
SIRT2	Intermediate protein	TUBB	Intermediate protein
SIRT2	Intermediate protein	SIRT1	Intermediate protein
SIRT2	Intermediate protein	HIST1H4A	Intermediate protein
SIRT2	Intermediate protein	KAT2B	Intermediate protein
SIRT2	Intermediate protein	KAT2A	Seed protein
SIRT2	Intermediate protein	RELA	Intermediate protein
SIRT2	Intermediate protein	HIST1H3A	Intermediate protein
SIRT2	Intermediate protein	MYC	Seed protein
SIRT2	Intermediate protein	SKP2	Intermediate protein
PML	Seed protein	PML	Seed protein
PML	Seed protein	MAPK11	Intermediate protein
PML	Seed protein	ASF1A	Intermediate protein
PML	Seed protein	ATF2	Seed protein
PML	Seed protein	RELA	Intermediate protein
PML	Seed protein	RB1	Intermediate protein
PML	Seed protein	RPL11	Intermediate protein
PML	Seed protein	CCNT1	Intermediate protein
PML	Seed protein	MYB	Intermediate protein
PML	Seed protein	MYC	Seed protein
PML	Seed protein	E2F4	Intermediate protein
PML	Seed protein	PPARGC1A	Intermediate protein

PML	Seed protein	SMAD3	Intermediate protein
PML	Seed protein	SMAD2	Intermediate protein
PML	Seed protein	DAXX	Intermediate protein
PML	Seed protein	PIAS2	Intermediate protein
PML	Seed protein	PIN1	Intermediate protein
PML	Seed protein	SPI1	Intermediate protein
PML	Seed protein	CUL3	Intermediate protein
PML	Seed protein	CUL1	Intermediate protein
PML	Seed protein	GATA2	Intermediate protein
PML	Seed protein	KAT5	Intermediate protein
PML	Seed protein	SKP1	Intermediate protein
PML	Seed protein	JUN	Intermediate protein
PML	Seed protein	SIRT1	Intermediate protein
PML	Seed protein	NCOR1	Intermediate protein
PML	Seed protein	SP1	Intermediate protein
PML	Seed protein	RBL2	Intermediate protein
PML	Seed protein	CDK2	Intermediate protein
PML	Seed protein	BCL2	Intermediate protein
PML	Seed protein	CDKN2A	Intermediate protein
PML	Seed protein	CSNK2A1	Intermediate protein
PML	Seed protein	H2AFX	Intermediate protein
PML	Seed protein	FOS	Intermediate protein
PML	Seed protein	HDAC3	Intermediate protein
PML	Seed protein	HDAC2	Intermediate protein
PML	Seed protein	HDAC7	Intermediate protein
PML	Seed protein	MAPK3	Intermediate protein
PML	Seed protein	MAPK1	Intermediate protein
PML	Seed protein	TRIM24	Intermediate protein
MAPK11	Intermediate protein	ATF2	Seed protein
MAPK11	Intermediate protein	HDAC3	Intermediate protein
MAPK11	Intermediate protein	PML	Seed protein
MAPK11	Intermediate protein	SP1	Intermediate protein

MAPK11	Intermediate protein	MYB	Intermediate protein
MAPK11	Intermediate protein	CCND1	Intermediate protein
HIST1H4A	Intermediate protein	ASF1A	Intermediate protein
HIST1H4A	Intermediate protein	RB1	Intermediate protein
HIST1H4A	Intermediate protein	DAXX	Intermediate protein
HIST1H4A	Intermediate protein	HIST1H3A	Intermediate protein
HIST1H4A	Intermediate protein	KAT5	Intermediate protein
HIST1H4A	Intermediate protein	SIRT2	Intermediate protein
HIST1H4A	Intermediate protein	KAT2B	Intermediate protein
HIST1H4A	Intermediate protein	NCOR1	Intermediate protein
HIST1H4A	Intermediate protein	KAT2A	Seed protein
HIST1H4A	Intermediate protein	CDK2	Intermediate protein
HIST1H4A	Intermediate protein	H2AFX	Intermediate protein
HIST1H4A	Intermediate protein	TAF1	Seed protein
HIST1H4A	Intermediate protein	HDAC3	Intermediate protein
HIST1H4A	Intermediate protein	HDAC2	Intermediate protein
HIST1H4A	Intermediate protein	SETD7	Intermediate protein
CUL4A	Intermediate protein	CUL4A	Intermediate protein
CUL4A	Intermediate protein	KAT2A	Seed protein
CUL4A	Intermediate protein	MYB	Intermediate protein

CUL4A	Intermediate protein	MYC	Seed protein
CUL4A	Intermediate protein	DDB1	Intermediate protein
CUL4A	Intermediate protein	SKP2	Intermediate protein
CDK9	Intermediate protein	CDK9	Intermediate protein
CDK9	Intermediate protein	RB1	Intermediate protein
CDK9	Intermediate protein	RELA	Intermediate protein
CDK9	Intermediate protein	CCNT1	Intermediate protein
CDK9	Intermediate protein	MYC	Seed protein
CDK9	Intermediate protein	TAF7	Seed protein
CDK9	Intermediate protein	CUL1	Intermediate protein
CDK9	Intermediate protein	SKP1	Intermediate protein
CDK9	Intermediate protein	SKP2	Intermediate protein
KAT2B	Intermediate protein	HIST1H4A	Intermediate protein
KAT2B	Intermediate protein	PTF1A	Intermediate protein
KAT2B	Intermediate protein	RB1	Intermediate protein
KAT2B	Intermediate protein	TRRAP	Intermediate protein
KAT2B	Intermediate protein	CCNT1	Intermediate protein
KAT2B	Intermediate protein	YY1	Intermediate protein
KAT2B	Intermediate protein	MYB	Intermediate protein
KAT2B	Intermediate protein	CCND1	Intermediate protein

KAT2B	Intermediate protein	MYC	Seed protein
KAT2B	Intermediate protein	SMAD3	Intermediate protein
KAT2B	Intermediate protein	SMAD2	Intermediate protein
KAT2B	Intermediate protein	TCF3	Seed protein
KAT2B	Intermediate protein	CTBP1	Intermediate protein
KAT2B	Intermediate protein	HIF1A	Intermediate protein
KAT2B	Intermediate protein	HIST1H3A	Intermediate protein
KAT2B	Intermediate protein	JUN	Intermediate protein
KAT2B	Intermediate protein	SIRT1	Intermediate protein
KAT2B	Intermediate protein	SIRT2	Intermediate protein
KAT2B	Intermediate protein	KAT2B	Intermediate protein
KAT2B	Intermediate protein	CDK2	Intermediate protein
KAT2B	Intermediate protein	H2AFX	Intermediate protein
KAT2B	Intermediate protein	CEBPB	Intermediate protein
KAT2B	Intermediate protein	HDAC3	Intermediate protein
KAT2B	Intermediate protein	VHL	Intermediate protein
SP1	Intermediate protein	PML	Seed protein
SP1	Intermediate protein	MAPK11	Intermediate protein
SP1	Intermediate protein	RB1	Intermediate protein
SP1	Intermediate protein	RELA	Intermediate protein

SP1	Intermediate protein	YY1	Intermediate protein
SP1	Intermediate protein	CCND1	Intermediate protein
SP1	Intermediate protein	MYC	Seed protein
SP1	Intermediate protein	SMAD3	Intermediate protein
SP1	Intermediate protein	SMAD2	Intermediate protein
SP1	Intermediate protein	SMAD4	Intermediate protein
SP1	Intermediate protein	PRKCD	Intermediate protein
SP1	Intermediate protein	PIN1	Intermediate protein
SP1	Intermediate protein	CTBP1	Intermediate protein
SP1	Intermediate protein	PRKDC	Intermediate protein
SP1	Intermediate protein	HIF1A	Intermediate protein
SP1	Intermediate protein	SKP2	Intermediate protein
SP1	Intermediate protein	JUN	Intermediate protein
SP1	Intermediate protein	RBL1	Intermediate protein
SP1	Intermediate protein	SP1	Intermediate protein
SP1	Intermediate protein	NCOR1	Intermediate protein
SP1	Intermediate protein	CDK2	Intermediate protein
SP1	Intermediate protein	TBP	Intermediate protein
SP1	Intermediate protein	CDKN2A	Intermediate protein
SP1	Intermediate protein	KLF4	Seed protein

SP1	Intermediate protein	TAF4	Intermediate protein
SP1	Intermediate protein	CEBPB	Intermediate protein
SP1	Intermediate protein	HDAC3	Intermediate protein
SP1	Intermediate protein	HDAC2	Intermediate protein
SP1	Intermediate protein	MAPK8	Intermediate protein
SP1	Intermediate protein	MAPK3	Intermediate protein
SP1	Intermediate protein	VHL	Intermediate protein
SP1	Intermediate protein	MAPK1	Intermediate protein
RBL1	Intermediate protein	SMAD3	Intermediate protein
RBL1	Intermediate protein	SMAD4	Intermediate protein
RBL1	Intermediate protein	TAF1	Seed protein
RBL1	Intermediate protein	SP1	Intermediate protein
RBL1	Intermediate protein	RBL2	Intermediate protein
RBL1	Intermediate protein	CDK2	Intermediate protein
RBL1	Intermediate protein	MYC	Seed protein
RBL1	Intermediate protein	E2F4	Intermediate protein
NCOR1	Intermediate protein	PML	Seed protein
NCOR1	Intermediate protein	HIST1H4A	Intermediate protein
NCOR1	Intermediate protein	MYC	Seed protein
NCOR1	Intermediate protein	HIST1H3A	Intermediate protein

NCOR1	Intermediate protein	VDR	Intermediate protein
NCOR1	Intermediate protein	JUN	Intermediate protein
NCOR1	Intermediate protein	SIRT1	Intermediate protein
NCOR1	Intermediate protein	SP1	Intermediate protein
NCOR1	Intermediate protein	CSNK2A1	Intermediate protein
NCOR1	Intermediate protein	CEBPB	Intermediate protein
NCOR1	Intermediate protein	HDAC3	Intermediate protein
NCOR1	Intermediate protein	HDAC7	Intermediate protein
KAT2A	Seed protein	USP22	Intermediate protein
KAT2A	Seed protein	TUBB	Intermediate protein
KAT2A	Seed protein	SIRT1	Intermediate protein
KAT2A	Seed protein	SIRT2	Intermediate protein
KAT2A	Seed protein	HIST1H4A	Intermediate protein
KAT2A	Seed protein	CUL4A	Intermediate protein
KAT2A	Seed protein	KAT2A	Seed protein
KAT2A	Seed protein	CDK2	Intermediate protein
KAT2A	Seed protein	PTF1A	Intermediate protein
KAT2A	Seed protein	RELA	Intermediate protein
KAT2A	Seed protein	TRRAP	Intermediate protein
KAT2A	Seed protein	MYB	Intermediate protein
KAT2A	Seed protein	MYC	Seed protein
KAT2A	Seed protein	E2F4	Intermediate protein
KAT2A	Seed protein	PPARGC1A	Intermediate protein
KAT2A	Seed protein	TAF9B	Intermediate protein
KAT2A	Seed protein	SMAD3	Intermediate protein
KAT2A	Seed protein	SMAD2	Intermediate protein
KAT2A	Seed protein	H2AFX	Intermediate protein
KAT2A	Seed protein	DDB1	Intermediate protein

KAT2A	Seed protein	TAF4	Intermediate protein
KAT2A	Seed protein	TAF3	Intermediate protein
KAT2A	Seed protein	CEBPB	Intermediate protein
KAT2A	Seed protein	MAX	Seed protein
KAT2A	Seed protein	CTBP1	Intermediate protein
KAT2A	Seed protein	CUL2	Intermediate protein
KAT2A	Seed protein	PRKDC	Intermediate protein
KAT2A	Seed protein	GATA2	Intermediate protein
KAT2A	Seed protein	HIST1H3A	Intermediate protein
RBL2	Intermediate protein	PML	Seed protein
RBL2	Intermediate protein	TAF1	Seed protein
RBL2	Intermediate protein	RBL1	Intermediate protein
RBL2	Intermediate protein	CDK2	Intermediate protein
RBL2	Intermediate protein	CUL1	Intermediate protein
RBL2	Intermediate protein	E2F4	Intermediate protein
RBL2	Intermediate protein	SKP1	Intermediate protein
RBL2	Intermediate protein	SKP2	Intermediate protein
CDK2	Intermediate protein	PML	Seed protein
CDK2	Intermediate protein	HIST1H4A	Intermediate protein
CDK2	Intermediate protein	RB1	Intermediate protein
CDK2	Intermediate protein	CCND1	Intermediate protein
CDK2	Intermediate protein	MYC	Seed protein
CDK2	Intermediate protein	SMAD3	Intermediate protein

CDK2	Intermediate protein	SMAD2	Intermediate protein
CDK2	Intermediate protein	PIN1	Intermediate protein
CDK2	Intermediate protein	CUL1	Intermediate protein
CDK2	Intermediate protein	SKP1	Intermediate protein
CDK2	Intermediate protein	SKP2	Intermediate protein
CDK2	Intermediate protein	SP1	Intermediate protein
CDK2	Intermediate protein	RBL1	Intermediate protein
CDK2	Intermediate protein	KAT2B	Intermediate protein
CDK2	Intermediate protein	KAT2A	Seed protein
CDK2	Intermediate protein	RBL2	Intermediate protein
CDK2	Intermediate protein	CDK2	Intermediate protein
CDK2	Intermediate protein	USP37	Intermediate protein
CDK2	Intermediate protein	CEBPA	Intermediate protein
BCL2	Intermediate protein	PML	Seed protein
BCL2	Intermediate protein	RELA	Intermediate protein
BCL2	Intermediate protein	MYC	Seed protein
BCL2	Intermediate protein	PIN1	Intermediate protein
BCL2	Intermediate protein	HIF1A	Intermediate protein
BCL2	Intermediate protein	BCL2	Intermediate protein
BCL2	Intermediate protein	CEBPA	Intermediate protein

BCL2	Intermediate protein	MAPK8	Intermediate protein
BCL2	Intermediate protein	MAPK1	Intermediate protein
ASF1A	Intermediate protein	CUL1	Intermediate protein
ASF1A	Intermediate protein	PML	Seed protein
ASF1A	Intermediate protein	TAF1	Seed protein
ASF1A	Intermediate protein	HIST1H4A	Intermediate protein
ASF1A	Intermediate protein	HIST1H3A	Intermediate protein
PTF1A	Intermediate protein	KAT2B	Intermediate protein
PTF1A	Intermediate protein	KAT2A	Seed protein
PTF1A	Intermediate protein	TCF3	Seed protein
ATF2	Seed protein	SMAD3	Intermediate protein
ATF2	Seed protein	JUN	Intermediate protein
ATF2	Seed protein	SMAD4	Intermediate protein
ATF2	Seed protein	CSNK2A1	Intermediate protein
ATF2	Seed protein	H2AFX	Intermediate protein
ATF2	Seed protein	FOS	Intermediate protein
ATF2	Seed protein	PML	Seed protein
ATF2	Seed protein	MAPK11	Intermediate protein
ATF2	Seed protein	ATF2	Seed protein
ATF2	Seed protein	CEBPA	Intermediate protein
ATF2	Seed protein	CEBPB	Intermediate protein
ATF2	Seed protein	RB1	Intermediate protein
ATF2	Seed protein	CUL3	Intermediate protein
ATF2	Seed protein	YY1	Intermediate protein
ATF2	Seed protein	MAPK8	Intermediate protein
ATF2	Seed protein	KAT5	Intermediate protein
ATF2	Seed protein	CCND1	Intermediate protein

ATF2	Seed protein	RUVBL2	Intermediate protein
ATF2	Seed protein	MAPK1	Intermediate protein
RB1	Intermediate protein	PML	Seed protein
RB1	Intermediate protein	HIST1H4A	Intermediate protein
RB1	Intermediate protein	ATF2	Seed protein
RB1	Intermediate protein	RB1	Intermediate protein
RB1	Intermediate protein	YY1	Intermediate protein
RB1	Intermediate protein	CCND1	Intermediate protein
RB1	Intermediate protein	MYC	Seed protein
RB1	Intermediate protein	E2F4	Intermediate protein
RB1	Intermediate protein	TCF3	Seed protein
RB1	Intermediate protein	SPI1	Intermediate protein
RB1	Intermediate protein	HIF1A	Intermediate protein
RB1	Intermediate protein	KAT5	Intermediate protein
RB1	Intermediate protein	SKP2	Intermediate protein
RB1	Intermediate protein	VDR	Intermediate protein
RB1	Intermediate protein	JUN	Intermediate protein
RB1	Intermediate protein	SIRT1	Intermediate protein
RB1	Intermediate protein	CDK9	Intermediate protein
RB1	Intermediate protein	KAT2B	Intermediate protein
RB1	Intermediate protein	SP1	Intermediate protein

RB1	Intermediate protein	CDK2	Intermediate protein
RB1	Intermediate protein	TBP	Intermediate protein
RB1	Intermediate protein	FOS	Intermediate protein
RB1	Intermediate protein	TAF1	Seed protein
RB1	Intermediate protein	CEBPB	Intermediate protein
RB1	Intermediate protein	HDAC3	Intermediate protein
RB1	Intermediate protein	HDAC2	Intermediate protein
RB1	Intermediate protein	SETD7	Intermediate protein
RB1	Intermediate protein	MAPK8	Intermediate protein
RELA	Intermediate protein	PML	Seed protein
RELA	Intermediate protein	RELA	Intermediate protein
RELA	Intermediate protein	MYC	Seed protein
RELA	Intermediate protein	RUVBL2	Intermediate protein
RELA	Intermediate protein	PPARGC 1A	Intermediate protein
RELA	Intermediate protein	DAXX	Intermediate protein
RELA	Intermediate protein	CUL2	Intermediate protein
RELA	Intermediate protein	HIF1A	Intermediate protein
RELA	Intermediate protein	KAT5	Intermediate protein
RELA	Intermediate protein	SNIP1	Intermediate protein
RELA	Intermediate protein	JUN	Intermediate protein

RELA	Intermediate protein	SIRT1	Intermediate protein
RELA	Intermediate protein	SIRT2	Intermediate protein
RELA	Intermediate protein	CDK9	Intermediate protein
RELA	Intermediate protein	SP1	Intermediate protein
RELA	Intermediate protein	KAT2A	Seed protein
RELA	Intermediate protein	BCL2	Intermediate protein
RELA	Intermediate protein	TBP	Intermediate protein
RELA	Intermediate protein	CSNK2A1	Intermediate protein
RELA	Intermediate protein	FOS	Intermediate protein
RELA	Intermediate protein	KLF4	Seed protein
RELA	Intermediate protein	TAF1	Seed protein
RELA	Intermediate protein	CEBPB	Intermediate protein
RELA	Intermediate protein	HDAC3	Intermediate protein
RELA	Intermediate protein	HDAC2	Intermediate protein
RELA	Intermediate protein	SETD7	Intermediate protein
USP37	Intermediate protein	USP37	Intermediate protein
USP37	Intermediate protein	MAX	Seed protein
USP37	Intermediate protein	CUL1	Intermediate protein
USP37	Intermediate protein	MYC	Seed protein
USP37	Intermediate protein	CDK2	Intermediate protein

USP37	Intermediate protein	SKP1	Intermediate protein
TRRAP	Intermediate protein	USP22	Intermediate protein
TRRAP	Intermediate protein	KAT2B	Intermediate protein
TRRAP	Intermediate protein	KAT2A	Seed protein
TRRAP	Intermediate protein	MAX	Seed protein
TRRAP	Intermediate protein	CUL1	Intermediate protein
TRRAP	Intermediate protein	KAT5	Intermediate protein
TRRAP	Intermediate protein	MYC	Seed protein
TRRAP	Intermediate protein	E2F4	Intermediate protein
TRRAP	Intermediate protein	SKP1	Intermediate protein
TRRAP	Intermediate protein	TAF9B	Intermediate protein
RPL11	Intermediate protein	MYC	Seed protein
RPL11	Intermediate protein	VHL	Intermediate protein
RPL11	Intermediate protein	PML	Seed protein
CCNT1	Intermediate protein	SMAD3	Intermediate protein
CCNT1	Intermediate protein	PML	Seed protein
CCNT1	Intermediate protein	CDK9	Intermediate protein
CCNT1	Intermediate protein	KAT2B	Intermediate protein
CCNT1	Intermediate protein	TAF7	Seed protein
CCNT1	Intermediate protein	MAX	Seed protein

CCNT1	Intermediate protein	CCNT1	Intermediate protein
CCNT1	Intermediate protein	MYC	Seed protein
HNRNPU	Intermediate protein	MAX	Seed protein
HNRNPU	Intermediate protein	GTF2F1	Intermediate protein
HNRNPU	Intermediate protein	MYC	Seed protein
YY1	Intermediate protein	KAT2B	Intermediate protein
YY1	Intermediate protein	SP1	Intermediate protein
YY1	Intermediate protein	ATF2	Seed protein
YY1	Intermediate protein	RB1	Intermediate protein
YY1	Intermediate protein	YY1	Intermediate protein
YY1	Intermediate protein	RUVBL2	Intermediate protein
YY1	Intermediate protein	MYC	Seed protein
YY1	Intermediate protein	SMAD3	Intermediate protein
YY1	Intermediate protein	SMAD2	Intermediate protein
YY1	Intermediate protein	SMAD4	Intermediate protein
YY1	Intermediate protein	CDKN2A	Intermediate protein
YY1	Intermediate protein	HDAC3	Intermediate protein
YY1	Intermediate protein	MAX	Seed protein
YY1	Intermediate protein	HDAC2	Intermediate protein
YY1	Intermediate protein	SKP2	Intermediate protein

MYB	Intermediate protein	PML	Seed protein
MYB	Intermediate protein	MAPK11	Intermediate protein
MYB	Intermediate protein	CUL4A	Intermediate protein
MYB	Intermediate protein	DDB1	Intermediate protein
MYB	Intermediate protein	KAT2B	Intermediate protein
MYB	Intermediate protein	KAT2A	Seed protein
MYB	Intermediate protein	CEBPB	Intermediate protein
MYB	Intermediate protein	KAT5	Intermediate protein
CCND1	Intermediate protein	MAPK11	Intermediate protein
CCND1	Intermediate protein	KAT2B	Intermediate protein
CCND1	Intermediate protein	SP1	Intermediate protein
CCND1	Intermediate protein	CDK2	Intermediate protein
CCND1	Intermediate protein	ATF2	Seed protein
CCND1	Intermediate protein	RB1	Intermediate protein
CCND1	Intermediate protein	CDKN2A	Intermediate protein
CCND1	Intermediate protein	TAF1	Seed protein
CCND1	Intermediate protein	HDAC3	Intermediate protein
CCND1	Intermediate protein	HDAC2	Intermediate protein
CCND1	Intermediate protein	CUL1	Intermediate protein
CCND1	Intermediate protein	SKP1	Intermediate protein

MYC	Seed protein	ZBTB17	Intermediate protein
MYC	Seed protein	MSH2	Intermediate protein
MYC	Seed protein	GTF2F1	Intermediate protein
MYC	Seed protein	PML	Seed protein
MYC	Seed protein	RELA	Intermediate protein
MYC	Seed protein	RB1	Intermediate protein
MYC	Seed protein	TRRAP	Intermediate protein
MYC	Seed protein	RPL11	Intermediate protein
MYC	Seed protein	CCNT1	Intermediate protein
MYC	Seed protein	HNRNPU	Intermediate protein
MYC	Seed protein	YY1	Intermediate protein
MYC	Seed protein	MYC	Seed protein
MYC	Seed protein	RUVBL2	Intermediate protein
MYC	Seed protein	SMAD3	Intermediate protein
MYC	Seed protein	SMAD2	Intermediate protein
MYC	Seed protein	PRKCD	Intermediate protein
MYC	Seed protein	PIAS2	Intermediate protein
MYC	Seed protein	PIN1	Intermediate protein
MYC	Seed protein	MAX	Seed protein
MYC	Seed protein	CUL1	Intermediate protein
MYC	Seed protein	PRKDC	Intermediate protein
MYC	Seed protein	HIF1A	Intermediate protein
MYC	Seed protein	KAT5	Intermediate protein
MYC	Seed protein	SNIP1	Intermediate protein
MYC	Seed protein	PIM1	Intermediate protein
MYC	Seed protein	SKP1	Intermediate protein
MYC	Seed protein	SKP2	Intermediate protein
MYC	Seed protein	VDR	Intermediate protein
MYC	Seed protein	JUN	Intermediate protein
MYC	Seed protein	USP22	Intermediate protein
MYC	Seed protein	TUBB	Intermediate protein
MYC	Seed protein	SIRT1	Intermediate protein
MYC	Seed protein	SIRT2	Intermediate protein
MYC	Seed protein	CDK9	Intermediate protein
MYC	Seed protein	CUL4A	Intermediate protein
MYC	Seed protein	KAT2B	Intermediate protein
MYC	Seed protein	SP1	Intermediate protein

MYC	Seed protein	RBL1	Intermediate protein
MYC	Seed protein	NCOR1	Intermediate protein
MYC	Seed protein	KAT2A	Seed protein
MYC	Seed protein	CDK2	Intermediate protein
MYC	Seed protein	BCL2	Intermediate protein
MYC	Seed protein	USP37	Intermediate protein
MYC	Seed protein	TBP	Intermediate protein
MYC	Seed protein	CDKN2A	Intermediate protein
MYC	Seed protein	CSNK2A 1	Intermediate protein
MYC	Seed protein	DDB1	Intermediate protein
MYC	Seed protein	CEBPA	Intermediate protein
MYC	Seed protein	CEBPB	Intermediate protein
MYC	Seed protein	HDAC3	Intermediate protein
MYC	Seed protein	HDAC2	Intermediate protein
MYC	Seed protein	MAPK8	Intermediate protein
MYC	Seed protein	MNT	Intermediate protein
MYC	Seed protein	MAPK3	Intermediate protein
MYC	Seed protein	VHL	Intermediate protein
MYC	Seed protein	MAPK1	Intermediate protein
RUVBL2	Intermediate protein	TBP	Intermediate protein
RUVBL2	Intermediate protein	ATF2	Seed protein
RUVBL2	Intermediate protein	RELA	Intermediate protein
RUVBL2	Intermediate protein	HDAC2	Intermediate protein
RUVBL2	Intermediate protein	HIF1A	Intermediate protein
RUVBL2	Intermediate protein	YY1	Intermediate protein
RUVBL2	Intermediate protein	KAT5	Intermediate protein
RUVBL2	Intermediate protein	MYC	Seed protein
RUVBL2	Intermediate protein	RUVBL2	Intermediate protein

E2F4	Intermediate protein	SMAD3	Intermediate protein
E2F4	Intermediate protein	PML	Seed protein
E2F4	Intermediate protein	RBL1	Intermediate protein
E2F4	Intermediate protein	RBL2	Intermediate protein
E2F4	Intermediate protein	KAT2A	Seed protein
E2F4	Intermediate protein	RB1	Intermediate protein
E2F4	Intermediate protein	HDAC3	Intermediate protein
E2F4	Intermediate protein	TRRAP	Intermediate protein
PPARGC1A	Intermediate protein	SIRT1	Intermediate protein
PPARGC1A	Intermediate protein	PML	Seed protein
PPARGC1A	Intermediate protein	KAT2A	Seed protein
PPARGC1A	Intermediate protein	RELA	Intermediate protein
PPARGC1A	Intermediate protein	SKP1	Intermediate protein
TAF9B	Intermediate protein	TAF1	Seed protein
TAF9B	Intermediate protein	TBP	Intermediate protein
TAF9B	Intermediate protein	KAT2A	Seed protein
TAF9B	Intermediate protein	TRRAP	Intermediate protein
TAF9B	Intermediate protein	USP22	Intermediate protein
TAF9B	Intermediate protein	TAF7	Seed protein
TAF9B	Intermediate protein	TAF5	Intermediate protein

TAF9B	Intermediate protein	TAF9B	Intermediate protein
SMAD3	Intermediate protein	PML	Seed protein
SMAD3	Intermediate protein	ATF2	Seed protein
SMAD3	Intermediate protein	CCNT1	Intermediate protein
SMAD3	Intermediate protein	YY1	Intermediate protein
SMAD3	Intermediate protein	MYC	Seed protein
SMAD3	Intermediate protein	E2F4	Intermediate protein
SMAD3	Intermediate protein	SMAD3	Intermediate protein
SMAD3	Intermediate protein	SMAD2	Intermediate protein
SMAD3	Intermediate protein	SMAD4	Intermediate protein
SMAD3	Intermediate protein	PIAS2	Intermediate protein
SMAD3	Intermediate protein	PIN1	Intermediate protein
SMAD3	Intermediate protein	MAX	Seed protein
SMAD3	Intermediate protein	CUL1	Intermediate protein
SMAD3	Intermediate protein	HIF1A	Intermediate protein
SMAD3	Intermediate protein	VDR	Intermediate protein
SMAD3	Intermediate protein	JUN	Intermediate protein
SMAD3	Intermediate protein	KAT2B	Intermediate protein
SMAD3	Intermediate protein	SP1	Intermediate protein
SMAD3	Intermediate protein	RBL1	Intermediate protein

SMAD3	Intermediate protein	KAT2A	Seed protein
SMAD3	Intermediate protein	CDK2	Intermediate protein
SMAD3	Intermediate protein	FOS	Intermediate protein
SMAD3	Intermediate protein	CEBPA	Intermediate protein
SMAD3	Intermediate protein	CEBPB	Intermediate protein
TBP	Intermediate protein	GTF2F1	Intermediate protein
TBP	Intermediate protein	RB1	Intermediate protein
TBP	Intermediate protein	RELA	Intermediate protein
TBP	Intermediate protein	MYC	Seed protein
TBP	Intermediate protein	RUVBL2	Intermediate protein
TBP	Intermediate protein	TAF9B	Intermediate protein
TBP	Intermediate protein	SPI1	Intermediate protein
TBP	Intermediate protein	CTBP1	Intermediate protein
TBP	Intermediate protein	JUN	Intermediate protein
TBP	Intermediate protein	SP1	Intermediate protein
TBP	Intermediate protein	TBP	Intermediate protein
TBP	Intermediate protein	FOS	Intermediate protein
TBP	Intermediate protein	TAF1	Seed protein
TBP	Intermediate protein	TAF7	Seed protein
TBP	Intermediate protein	TAF5	Intermediate protein

TBP	Intermediate protein	TAF4	Intermediate protein
TBP	Intermediate protein	TAF3	Intermediate protein
TBP	Intermediate protein	SETD7	Intermediate protein
SMAD2	Intermediate protein	PML	Seed protein
SMAD2	Intermediate protein	YY1	Intermediate protein
SMAD2	Intermediate protein	MYC	Seed protein
SMAD2	Intermediate protein	SMAD3	Intermediate protein
SMAD2	Intermediate protein	SMAD2	Intermediate protein
SMAD2	Intermediate protein	SMAD4	Intermediate protein
SMAD2	Intermediate protein	PIN1	Intermediate protein
SMAD2	Intermediate protein	SNIP1	Intermediate protein
SMAD2	Intermediate protein	JUN	Intermediate protein
SMAD2	Intermediate protein	KAT2B	Intermediate protein
SMAD2	Intermediate protein	SP1	Intermediate protein
SMAD2	Intermediate protein	KAT2A	Seed protein
SMAD2	Intermediate protein	CDK2	Intermediate protein
SMAD4	Intermediate protein	SP1	Intermediate protein
SMAD4	Intermediate protein	RBL1	Intermediate protein
SMAD4	Intermediate protein	ATF2	Seed protein
SMAD4	Intermediate protein	YY1	Intermediate protein

SMAD4	Intermediate protein	SMAD3	Intermediate protein
SMAD4	Intermediate protein	SMAD2	Intermediate protein
SMAD4	Intermediate protein	DAXX	Intermediate protein
SMAD4	Intermediate protein	SMAD4	Intermediate protein
SMAD4	Intermediate protein	PIAS2	Intermediate protein
SMAD4	Intermediate protein	CEBPA	Intermediate protein
SMAD4	Intermediate protein	CEBPB	Intermediate protein
SMAD4	Intermediate protein	MAX	Seed protein
SMAD4	Intermediate protein	SNIP1	Intermediate protein
SMAD4	Intermediate protein	SKP2	Intermediate protein
DAXX	Intermediate protein	PML	Seed protein
DAXX	Intermediate protein	HIST1H4A	Intermediate protein
DAXX	Intermediate protein	RELA	Intermediate protein
DAXX	Intermediate protein	DAXX	Intermediate protein
DAXX	Intermediate protein	SMAD4	Intermediate protein
DAXX	Intermediate protein	CDKN2A	Intermediate protein
DAXX	Intermediate protein	CSNK2A1	Intermediate protein
DAXX	Intermediate protein	TCF3	Seed protein
DAXX	Intermediate protein	PIN1	Intermediate protein
DAXX	Intermediate protein	CEBPB	Intermediate protein

DAXX	Intermediate protein	HDAC3	Intermediate protein
DAXX	Intermediate protein	HDAC2	Intermediate protein
DAXX	Intermediate protein	CUL3	Intermediate protein
PRKCD	Intermediate protein	SP1	Intermediate protein
PRKCD	Intermediate protein	MYC	Seed protein
PRKCD	Intermediate protein	PRKCD	Intermediate protein
PRKCD	Intermediate protein	KLF4	Seed protein
PRKCD	Intermediate protein	SPI1	Intermediate protein
PRKCD	Intermediate protein	PRKDC	Intermediate protein
PRKCD	Intermediate protein	VHL	Intermediate protein
CDKN2A	Intermediate protein	ZBTB17	Intermediate protein
CDKN2A	Intermediate protein	PML	Seed protein
CDKN2A	Intermediate protein	SP1	Intermediate protein
CDKN2A	Intermediate protein	YY1	Intermediate protein
CDKN2A	Intermediate protein	CCND1	Intermediate protein
CDKN2A	Intermediate protein	MYC	Seed protein
CDKN2A	Intermediate protein	DAXX	Intermediate protein
CDKN2A	Intermediate protein	PIAS2	Intermediate protein
CDKN2A	Intermediate protein	HIF1A	Intermediate protein
CDKN2A	Intermediate protein	MAPK8	Intermediate protein

CDKN2A	Intermediate protein	KAT5	Intermediate protein
CDKN2A	Intermediate protein	VHL	Intermediate protein
CSNK2A1	Intermediate protein	PML	Seed protein
CSNK2A1	Intermediate protein	ATF2	Seed protein
CSNK2A1	Intermediate protein	RELA	Intermediate protein
CSNK2A1	Intermediate protein	MYC	Seed protein
CSNK2A1	Intermediate protein	DAXX	Intermediate protein
CSNK2A1	Intermediate protein	PIN1	Intermediate protein
CSNK2A1	Intermediate protein	SPI1	Intermediate protein
CSNK2A1	Intermediate protein	PRKDC	Intermediate protein
CSNK2A1	Intermediate protein	HIF1A	Intermediate protein
CSNK2A1	Intermediate protein	JUN	Intermediate protein
CSNK2A1	Intermediate protein	SIRT1	Intermediate protein
CSNK2A1	Intermediate protein	NCOR1	Intermediate protein
CSNK2A1	Intermediate protein	CSNK2A1	Intermediate protein
CSNK2A1	Intermediate protein	H2AFX	Intermediate protein
CSNK2A1	Intermediate protein	FOS	Intermediate protein
CSNK2A1	Intermediate protein	TAF1	Seed protein
CSNK2A1	Intermediate protein	HDAC3	Intermediate protein
CSNK2A1	Intermediate protein	HDAC2	Intermediate protein

CSNK2A1	Intermediate protein	VHL	Intermediate protein
H2AFX	Intermediate protein	PML	Seed protein
H2AFX	Intermediate protein	HIST1H4A	Intermediate protein
H2AFX	Intermediate protein	KAT2B	Intermediate protein
H2AFX	Intermediate protein	KAT2A	Seed protein
H2AFX	Intermediate protein	ATF2	Seed protein
H2AFX	Intermediate protein	CSNK2A1	Intermediate protein
H2AFX	Intermediate protein	PRKDC	Intermediate protein
H2AFX	Intermediate protein	HIST1H3A	Intermediate protein
H2AFX	Intermediate protein	MAPK8	Intermediate protein
H2AFX	Intermediate protein	KAT5	Intermediate protein
FOS	Intermediate protein	VDR	Intermediate protein
FOS	Intermediate protein	JUN	Intermediate protein
FOS	Intermediate protein	GTF2F1	Intermediate protein
FOS	Intermediate protein	SIRT1	Intermediate protein
FOS	Intermediate protein	PML	Seed protein
FOS	Intermediate protein	ATF2	Seed protein
FOS	Intermediate protein	RB1	Intermediate protein
FOS	Intermediate protein	RELA	Intermediate protein
FOS	Intermediate protein	SMAD3	Intermediate protein

FOS	Intermediate protein	TBP	Intermediate protein
FOS	Intermediate protein	CSNK2A1	Intermediate protein
FOS	Intermediate protein	TAF1	Seed protein
FOS	Intermediate protein	SPI1	Intermediate protein
FOS	Intermediate protein	MAPK1	Intermediate protein
KLF4	Seed protein	RELA	Intermediate protein
KLF4	Seed protein	SPI1	Intermediate protein
KLF4	Seed protein	PRKCD	Intermediate protein
KLF4	Seed protein	HDAC2	Intermediate protein
KLF4	Seed protein	CTBP1	Intermediate protein
KLF4	Seed protein	SETD7	Intermediate protein
KLF4	Seed protein	CUL2	Intermediate protein
KLF4	Seed protein	HDAC7	Intermediate protein
KLF4	Seed protein	SP1	Intermediate protein
KLF4	Seed protein	KAT5	Intermediate protein
KLF4	Seed protein	VHL	Intermediate protein
PIAS2	Intermediate protein	SMAD3	Intermediate protein
PIAS2	Intermediate protein	JUN	Intermediate protein
PIAS2	Intermediate protein	SMAD4	Intermediate protein
PIAS2	Intermediate protein	CDKN2A	Intermediate protein
PIAS2	Intermediate protein	PIAS2	Intermediate protein
PIAS2	Intermediate protein	PML	Seed protein
PIAS2	Intermediate protein	MAPK8	Intermediate protein
PIAS2	Intermediate protein	MYC	Seed protein
TAF1	Seed protein	TBP	Intermediate protein
TAF1	Seed protein	JUN	Intermediate protein

TAF1	Seed protein	CSNK2A1	Intermediate protein
TAF1	Seed protein	FOS	Intermediate protein
TAF1	Seed protein	GTF2F1	Intermediate protein
TAF1	Seed protein	HIST1H4A	Intermediate protein
TAF1	Seed protein	RBL1	Intermediate protein
TAF1	Seed protein	RBL2	Intermediate protein
TAF1	Seed protein	ASF1A	Intermediate protein
TAF1	Seed protein	TAF7	Seed protein
TAF1	Seed protein	TAF5	Intermediate protein
TAF1	Seed protein	TAF4	Intermediate protein
TAF1	Seed protein	RB1	Intermediate protein
TAF1	Seed protein	RELA	Intermediate protein
TAF1	Seed protein	HIST1H3A	Intermediate protein
TAF1	Seed protein	CCND1	Intermediate protein
TAF1	Seed protein	TAF9B	Intermediate protein
DDB1	Intermediate protein	CUL4A	Intermediate protein
DDB1	Intermediate protein	KAT2A	Seed protein
DDB1	Intermediate protein	MYB	Intermediate protein
DDB1	Intermediate protein	MYC	Seed protein
DDB1	Intermediate protein	DDB1	Intermediate protein
DDB1	Intermediate protein	SKP2	Intermediate protein
TAF7	Seed protein	TBP	Intermediate protein
TAF7	Seed protein	VDR	Intermediate protein
TAF7	Seed protein	CCNT1	Intermediate protein
TAF7	Seed protein	SETD7	Intermediate protein
TAF7	Seed protein	TAF1	Seed protein
TAF7	Seed protein	CDK9	Intermediate protein
TAF7	Seed protein	TAF5	Intermediate protein
TAF7	Seed protein	TAF3	Intermediate protein

TAF7	Seed protein	TAF9B	Intermediate protein
TAF7	Seed protein	TRIM24	Intermediate protein
TCF3	Seed protein	VDR	Intermediate protein
TCF3	Seed protein	DAXX	Intermediate protein
TCF3	Seed protein	KAT2B	Intermediate protein
TCF3	Seed protein	TCF3	Seed protein
TCF3	Seed protein	PTF1A	Intermediate protein
TCF3	Seed protein	RB1	Intermediate protein
TCF3	Seed protein	HDAC2	Intermediate protein
TCF3	Seed protein	GATA2	Intermediate protein
TCF3	Seed protein	SKP2	Intermediate protein
TAF5	Intermediate protein	TBP	Intermediate protein
TAF5	Intermediate protein	TAF1	Seed protein
TAF5	Intermediate protein	TAF7	Seed protein
TAF5	Intermediate protein	TAF4	Intermediate protein
TAF5	Intermediate protein	TAF3	Intermediate protein
TAF5	Intermediate protein	TAF9B	Intermediate protein
TAF4	Intermediate protein	TBP	Intermediate protein
TAF4	Intermediate protein	JUN	Intermediate protein
TAF4	Intermediate protein	TAF1	Seed protein
TAF4	Intermediate protein	SP1	Intermediate protein
TAF4	Intermediate protein	KAT2A	Seed protein
TAF4	Intermediate protein	TAF5	Intermediate protein
TAF4	Intermediate protein	TAF4	Intermediate protein
TAF3	Intermediate protein	TBP	Intermediate protein

TAF3	Intermediate protein	KAT2A	Seed protein
TAF3	Intermediate protein	TAF7	Seed protein
TAF3	Intermediate protein	TAF5	Intermediate protein
PIN1	Intermediate protein	JUN	Intermediate protein
PIN1	Intermediate protein	PML	Seed protein
PIN1	Intermediate protein	SP1	Intermediate protein
PIN1	Intermediate protein	CDK2	Intermediate protein
PIN1	Intermediate protein	BCL2	Intermediate protein
PIN1	Intermediate protein	MYC	Seed protein
PIN1	Intermediate protein	SMAD3	Intermediate protein
PIN1	Intermediate protein	SMAD2	Intermediate protein
PIN1	Intermediate protein	DAXX	Intermediate protein
PIN1	Intermediate protein	CSNK2A1	Intermediate protein
PIN1	Intermediate protein	HDAC3	Intermediate protein
PIN1	Intermediate protein	PIM1	Intermediate protein
CEBPA	Intermediate protein	SMAD3	Intermediate protein
CEBPA	Intermediate protein	VDR	Intermediate protein
CEBPA	Intermediate protein	SMAD4	Intermediate protein
CEBPA	Intermediate protein	CDK2	Intermediate protein
CEBPA	Intermediate protein	BCL2	Intermediate protein

CEBPA	Intermediate protein	ATF2	Seed protein
CEBPA	Intermediate protein	CEBPA	Intermediate protein
CEBPA	Intermediate protein	CEBPB	Intermediate protein
CEBPA	Intermediate protein	SPI1	Intermediate protein
CEBPA	Intermediate protein	HDAC2	Intermediate protein
CEBPA	Intermediate protein	MAPK8	Intermediate protein
CEBPA	Intermediate protein	MYC	Seed protein
CEBPB	Intermediate protein	SP1	Intermediate protein
CEBPB	Intermediate protein	KAT2B	Intermediate protein
CEBPB	Intermediate protein	NCOR1	Intermediate protein
CEBPB	Intermediate protein	KAT2A	Seed protein
CEBPB	Intermediate protein	ATF2	Seed protein
CEBPB	Intermediate protein	RELA	Intermediate protein
CEBPB	Intermediate protein	RB1	Intermediate protein
CEBPB	Intermediate protein	MYB	Intermediate protein
CEBPB	Intermediate protein	MYC	Seed protein
CEBPB	Intermediate protein	SMAD3	Intermediate protein
CEBPB	Intermediate protein	DAXX	Intermediate protein
CEBPB	Intermediate protein	SMAD4	Intermediate protein
CEBPB	Intermediate protein	CEBPA	Intermediate protein

CEBPB	Intermediate protein	CEBPB	Intermediate protein
CEBPB	Intermediate protein	SPI1	Intermediate protein
CEBPB	Intermediate protein	MAPK1	Intermediate protein
MAX	Seed protein	SMAD3	Intermediate protein
MAX	Seed protein	SMAD4	Intermediate protein
MAX	Seed protein	ZBTB17	Intermediate protein
MAX	Seed protein	MSH2	Intermediate protein
MAX	Seed protein	KAT2A	Seed protein
MAX	Seed protein	USP37	Intermediate protein
MAX	Seed protein	MAX	Seed protein
MAX	Seed protein	TRRAP	Intermediate protein
MAX	Seed protein	CCNT1	Intermediate protein
MAX	Seed protein	HNRNPU	Intermediate protein
MAX	Seed protein	HIF1A	Intermediate protein
MAX	Seed protein	YY1	Intermediate protein
MAX	Seed protein	MNT	Intermediate protein
MAX	Seed protein	MYC	Seed protein
MAX	Seed protein	SNIP1	Intermediate protein
MAX	Seed protein	PIM1	Intermediate protein
HDAC3	Intermediate protein	PML	Seed protein
HDAC3	Intermediate protein	MAPK11	Intermediate protein
HDAC3	Intermediate protein	HIST1H4A	Intermediate protein
HDAC3	Intermediate protein	RELA	Intermediate protein
HDAC3	Intermediate protein	RB1	Intermediate protein
HDAC3	Intermediate protein	YY1	Intermediate protein
HDAC3	Intermediate protein	CCND1	Intermediate protein
HDAC3	Intermediate protein	MYC	Seed protein

HDAC3	Intermediate protein	E2F4	Intermediate protein
HDAC3	Intermediate protein	DAXX	Intermediate protein
HDAC3	Intermediate protein	PIN1	Intermediate protein
HDAC3	Intermediate protein	SPI1	Intermediate protein
HDAC3	Intermediate protein	CTBP1	Intermediate protein
HDAC3	Intermediate protein	HIF1A	Intermediate protein
HDAC3	Intermediate protein	GATA2	Intermediate protein
HDAC3	Intermediate protein	KAT5	Intermediate protein
HDAC3	Intermediate protein	JUN	Intermediate protein
HDAC3	Intermediate protein	NCOR1	Intermediate protein
HDAC3	Intermediate protein	SP1	Intermediate protein
HDAC3	Intermediate protein	KAT2B	Intermediate protein
HDAC3	Intermediate protein	CSNK2A1	Intermediate protein
HDAC3	Intermediate protein	HDAC3	Intermediate protein
HDAC3	Intermediate protein	HDAC7	Intermediate protein
SPI1	Intermediate protein	TBP	Intermediate protein
SPI1	Intermediate protein	JUN	Intermediate protein
SPI1	Intermediate protein	PRKCD	Intermediate protein
SPI1	Intermediate protein	CSNK2A1	Intermediate protein
SPI1	Intermediate protein	FOS	Intermediate protein

SPI1	Intermediate protein	KLF4	Seed protein
SPI1	Intermediate protein	PML	Seed protein
SPI1	Intermediate protein	CEBPA	Intermediate protein
SPI1	Intermediate protein	CEBPB	Intermediate protein
SPI1	Intermediate protein	RB1	Intermediate protein
SPI1	Intermediate protein	HDAC3	Intermediate protein
SPI1	Intermediate protein	GATA2	Intermediate protein
SPI1	Intermediate protein	MAPK8	Intermediate protein
HDAC2	Intermediate protein	PML	Seed protein
HDAC2	Intermediate protein	HIST1H4A	Intermediate protein
HDAC2	Intermediate protein	RELA	Intermediate protein
HDAC2	Intermediate protein	RB1	Intermediate protein
HDAC2	Intermediate protein	YY1	Intermediate protein
HDAC2	Intermediate protein	CCND1	Intermediate protein
HDAC2	Intermediate protein	MYC	Seed protein
HDAC2	Intermediate protein	RUVBL2	Intermediate protein
HDAC2	Intermediate protein	DAXX	Intermediate protein
HDAC2	Intermediate protein	TCF3	Seed protein
HDAC2	Intermediate protein	CTBP1	Intermediate protein
HDAC2	Intermediate protein	HIST1H3A	Intermediate protein

HDAC2	Intermediate protein	SP1	Intermediate protein
HDAC2	Intermediate protein	CSNK2A1	Intermediate protein
HDAC2	Intermediate protein	KLF4	Seed protein
HDAC2	Intermediate protein	CEBPA	Intermediate protein
HDAC2	Intermediate protein	HDAC7	Intermediate protein
HDAC2	Intermediate protein	VHL	Intermediate protein
CTBP1	Intermediate protein	KAT2B	Intermediate protein
CTBP1	Intermediate protein	SP1	Intermediate protein
CTBP1	Intermediate protein	KAT2A	Seed protein
CTBP1	Intermediate protein	TBP	Intermediate protein
CTBP1	Intermediate protein	KLF4	Seed protein
CTBP1	Intermediate protein	HDAC3	Intermediate protein
CTBP1	Intermediate protein	HDAC2	Intermediate protein
CTBP1	Intermediate protein	CTBP1	Intermediate protein
SETD7	Intermediate protein	TBP	Intermediate protein
SETD7	Intermediate protein	SIRT1	Intermediate protein
SETD7	Intermediate protein	KLF4	Seed protein
SETD7	Intermediate protein	HIST1H4A	Intermediate protein
SETD7	Intermediate protein	TAF7	Seed protein
SETD7	Intermediate protein	RELA	Intermediate protein

SETD7	Intermediate protein	RB1	Intermediate protein
SETD7	Intermediate protein	SETD7	Intermediate protein
SETD7	Intermediate protein	HIST1H3A	Intermediate protein
CUL3	Intermediate protein	PML	Seed protein
CUL3	Intermediate protein	ATF2	Seed protein
CUL3	Intermediate protein	DAXX	Intermediate protein
CUL3	Intermediate protein	CUL3	Intermediate protein
CUL3	Intermediate protein	KAT5	Intermediate protein
CUL2	Intermediate protein	KLF4	Seed protein
CUL2	Intermediate protein	KAT2A	Seed protein
CUL2	Intermediate protein	RELA	Intermediate protein
CUL2	Intermediate protein	HIF1A	Intermediate protein
CUL2	Intermediate protein	VHL	Intermediate protein
CUL2	Intermediate protein	SKP2	Intermediate protein
CUL1	Intermediate protein	PML	Seed protein
CUL1	Intermediate protein	ASF1A	Intermediate protein
CUL1	Intermediate protein	TRRAP	Intermediate protein
CUL1	Intermediate protein	CCND1	Intermediate protein
CUL1	Intermediate protein	MYC	Seed protein
CUL1	Intermediate protein	SMAD3	Intermediate protein

CUL1	Intermediate protein	CUL1	Intermediate protein
CUL1	Intermediate protein	GATA2	Intermediate protein
CUL1	Intermediate protein	SKP1	Intermediate protein
CUL1	Intermediate protein	SKP2	Intermediate protein
CUL1	Intermediate protein	CDK9	Intermediate protein
CUL1	Intermediate protein	RBL2	Intermediate protein
CUL1	Intermediate protein	CDK2	Intermediate protein
CUL1	Intermediate protein	USP37	Intermediate protein
PRKDC	Intermediate protein	JUN	Intermediate protein
PRKDC	Intermediate protein	SP1	Intermediate protein
PRKDC	Intermediate protein	KAT2A	Seed protein
PRKDC	Intermediate protein	MYC	Seed protein
PRKDC	Intermediate protein	PRKCD	Intermediate protein
PRKDC	Intermediate protein	CSNK2A1	Intermediate protein
PRKDC	Intermediate protein	H2AFX	Intermediate protein
PRKDC	Intermediate protein	HIST1H3A	Intermediate protein
PRKDC	Intermediate protein	MAPK8	Intermediate protein
PRKDC	Intermediate protein	KAT5	Intermediate protein
HIF1A	Intermediate protein	RB1	Intermediate protein
HIF1A	Intermediate protein	RELA	Intermediate protein

HIF1A	Intermediate protein	RUVBL2	Intermediate protein
HIF1A	Intermediate protein	MYC	Seed protein
HIF1A	Intermediate protein	SMAD3	Intermediate protein
HIF1A	Intermediate protein	MAX	Seed protein
HIF1A	Intermediate protein	CUL2	Intermediate protein
HIF1A	Intermediate protein	HIF1A	Intermediate protein
HIF1A	Intermediate protein	JUN	Intermediate protein
HIF1A	Intermediate protein	SP1	Intermediate protein
HIF1A	Intermediate protein	KAT2B	Intermediate protein
HIF1A	Intermediate protein	BCL2	Intermediate protein
HIF1A	Intermediate protein	CDKN2A	Intermediate protein
HIF1A	Intermediate protein	CSNK2A1	Intermediate protein
HIF1A	Intermediate protein	HDAC3	Intermediate protein
HIF1A	Intermediate protein	HDAC7	Intermediate protein
HIF1A	Intermediate protein	VHL	Intermediate protein
HIF1A	Intermediate protein	MAPK1	Intermediate protein
GATA2	Intermediate protein	HDAC3	Intermediate protein
GATA2	Intermediate protein	SPI1	Intermediate protein
GATA2	Intermediate protein	JUN	Intermediate protein
GATA2	Intermediate protein	CUL1	Intermediate protein

GATA2	Intermediate protein	PML	Seed protein
GATA2	Intermediate protein	KAT2A	Seed protein
GATA2	Intermediate protein	TCF3	Seed protein
GATA2	Intermediate protein	SKP1	Intermediate protein
HDAC7	Intermediate protein	KLF4	Seed protein
HDAC7	Intermediate protein	PML	Seed protein
HDAC7	Intermediate protein	NCOR1	Intermediate protein
HDAC7	Intermediate protein	HDAC3	Intermediate protein
HDAC7	Intermediate protein	HDAC2	Intermediate protein
HDAC7	Intermediate protein	HIF1A	Intermediate protein
HDAC7	Intermediate protein	HDAC7	Intermediate protein
HDAC7	Intermediate protein	KAT5	Intermediate protein
HIST1H3A	Intermediate protein	HIST1H4A	Intermediate protein
HIST1H3A	Intermediate protein	ASF1A	Intermediate protein
HIST1H3A	Intermediate protein	PRKDC	Intermediate protein
HIST1H3A	Intermediate protein	KAT5	Intermediate protein
HIST1H3A	Intermediate protein	SIRT1	Intermediate protein
HIST1H3A	Intermediate protein	SIRT2	Intermediate protein
HIST1H3A	Intermediate protein	NCOR1	Intermediate protein
HIST1H3A	Intermediate protein	KAT2B	Intermediate protein

HIST1H3A	Intermediate protein	KAT2A	Seed protein
HIST1H3A	Intermediate protein	H2AFX	Intermediate protein
HIST1H3A	Intermediate protein	TAF1	Seed protein
HIST1H3A	Intermediate protein	HDAC2	Intermediate protein
HIST1H3A	Intermediate protein	SETD7	Intermediate protein
MAPK8	Intermediate protein	ATF2	Seed protein
MAPK8	Intermediate protein	RB1	Intermediate protein
MAPK8	Intermediate protein	MYC	Seed protein
MAPK8	Intermediate protein	PIAS2	Intermediate protein
MAPK8	Intermediate protein	SPI1	Intermediate protein
MAPK8	Intermediate protein	PRKDC	Intermediate protein
MAPK8	Intermediate protein	JUN	Intermediate protein
MAPK8	Intermediate protein	SIRT1	Intermediate protein
MAPK8	Intermediate protein	SP1	Intermediate protein
MAPK8	Intermediate protein	BCL2	Intermediate protein
MAPK8	Intermediate protein	CDKN2A	Intermediate protein
MAPK8	Intermediate protein	H2AFX	Intermediate protein
MAPK8	Intermediate protein	CEBPA	Intermediate protein
MAPK8	Intermediate protein	MAPK8	Intermediate protein
MNT	Intermediate protein	MAX	Seed protein

MNT	Intermediate protein	MNT	Intermediate protein
MNT	Intermediate protein	MYC	Seed protein
KAT5	Intermediate protein	PML	Seed protein
KAT5	Intermediate protein	HIST1H4A	Intermediate protein
KAT5	Intermediate protein	ATF2	Seed protein
KAT5	Intermediate protein	RELA	Intermediate protein
KAT5	Intermediate protein	RB1	Intermediate protein
KAT5	Intermediate protein	TRRAP	Intermediate protein
KAT5	Intermediate protein	MYB	Intermediate protein
KAT5	Intermediate protein	MYC	Seed protein
KAT5	Intermediate protein	RUVBL2	Intermediate protein
KAT5	Intermediate protein	CUL3	Intermediate protein
KAT5	Intermediate protein	PRKDC	Intermediate protein
KAT5	Intermediate protein	HIST1H3A	Intermediate protein
KAT5	Intermediate protein	KAT5	Intermediate protein
KAT5	Intermediate protein	SIRT1	Intermediate protein
KAT5	Intermediate protein	CDKN2A	Intermediate protein
KAT5	Intermediate protein	H2AFX	Intermediate protein
KAT5	Intermediate protein	KLF4	Seed protein
KAT5	Intermediate protein	HDAC3	Intermediate protein

KAT5	Intermediate protein	HDAC7	Intermediate protein
KAT5	Intermediate protein	VHL	Intermediate protein
SNIP1	Intermediate protein	SMAD2	Intermediate protein
SNIP1	Intermediate protein	RELA	Intermediate protein
SNIP1	Intermediate protein	MAX	Seed protein
SNIP1	Intermediate protein	SMAD4	Intermediate protein
SNIP1	Intermediate protein	MYC	Seed protein
MAPK3	Intermediate protein	JUN	Intermediate protein
MAPK3	Intermediate protein	PML	Seed protein
MAPK3	Intermediate protein	SP1	Intermediate protein
MAPK3	Intermediate protein	MYC	Seed protein
VHL	Intermediate protein	RPL11	Intermediate protein
VHL	Intermediate protein	MYC	Seed protein
VHL	Intermediate protein	PRKCD	Intermediate protein
VHL	Intermediate protein	CUL2	Intermediate protein
VHL	Intermediate protein	HIF1A	Intermediate protein
VHL	Intermediate protein	KAT5	Intermediate protein
VHL	Intermediate protein	SKP2	Intermediate protein
VHL	Intermediate protein	SIRT1	Intermediate protein
VHL	Intermediate protein	KAT2B	Intermediate protein

VHL	Intermediate protein	SP1	Intermediate protein
VHL	Intermediate protein	CDKN2A	Intermediate protein
VHL	Intermediate protein	CSNK2A1	Intermediate protein
VHL	Intermediate protein	KLF4	Seed protein
VHL	Intermediate protein	HDAC2	Intermediate protein
VHL	Intermediate protein	VHL	Intermediate protein
PIM1	Intermediate protein	PIN1	Intermediate protein
PIM1	Intermediate protein	MAX	Seed protein
PIM1	Intermediate protein	MYC	Seed protein
PIM1	Intermediate protein	PIM1	Intermediate protein
MAPK1	Intermediate protein	PML	Seed protein
MAPK1	Intermediate protein	ATF2	Seed protein
MAPK1	Intermediate protein	MYC	Seed protein
MAPK1	Intermediate protein	HIF1A	Intermediate protein
MAPK1	Intermediate protein	JUN	Intermediate protein
MAPK1	Intermediate protein	SP1	Intermediate protein
MAPK1	Intermediate protein	BCL2	Intermediate protein
MAPK1	Intermediate protein	FOS	Intermediate protein
MAPK1	Intermediate protein	CEBPB	Intermediate protein
MAPK1	Intermediate protein	MAPK1	Intermediate protein

SKP1	Intermediate protein	PML	Seed protein
SKP1	Intermediate protein	CDK9	Intermediate protein
SKP1	Intermediate protein	RBL2	Intermediate protein
SKP1	Intermediate protein	CDK2	Intermediate protein
SKP1	Intermediate protein	USP37	Intermediate protein
SKP1	Intermediate protein	TRRAP	Intermediate protein
SKP1	Intermediate protein	CCND1	Intermediate protein
SKP1	Intermediate protein	MYC	Seed protein
SKP1	Intermediate protein	PPARGC 1A	Intermediate protein
SKP1	Intermediate protein	CUL1	Intermediate protein
SKP1	Intermediate protein	GATA2	Intermediate protein
SKP1	Intermediate protein	SKP2	Intermediate protein
SKP2	Intermediate protein	SIRT2	Intermediate protein
SKP2	Intermediate protein	CDK9	Intermediate protein
SKP2	Intermediate protein	CUL4A	Intermediate protein
SKP2	Intermediate protein	SP1	Intermediate protein
SKP2	Intermediate protein	RBL2	Intermediate protein
SKP2	Intermediate protein	CDK2	Intermediate protein
SKP2	Intermediate protein	RB1	Intermediate protein
SKP2	Intermediate protein	YY1	Intermediate protein

SKP2	Intermediate protein	MYC	Seed protein
SKP2	Intermediate protein	SMAD4	Intermediate protein

SKP2	Intermediate protein	DDB1	Intermediate protein
SKP2	Intermediate protein	TCF3	Seed protein
SKP2	Intermediate protein	CUL2	Intermediate protein
SKP2	Intermediate protein	CUL1	Intermediate protein
SKP2	Intermediate protein	VHL	Intermediate protein
SKP2	Intermediate protein	SKP1	Intermediate protein
SKP2	Intermediate protein	SKP2	Intermediate protein
TRIM24	Intermediate protein	PML	Seed protein
TRIM24	Intermediate protein	TAF7	Seed protein
TRIM24	Intermediate protein	TRIM24	Intermediate protein

SKP2	Intermediate protein	DDB1	Intermediate protein
SKP2	Intermediate protein	TCF3	Seed protein
SKP2	Intermediate protein	CUL2	Intermediate protein
SKP2	Intermediate protein	CUL1	Intermediate protein
SKP2	Intermediate protein	VHL	Intermediate protein
SKP2	Intermediate protein	SKP1	Intermediate protein
SKP2	Intermediate protein	SKP2	Intermediate protein
TRIM24	Intermediate protein	PML	Seed protein

TRIM24		Intermediate protein	TAF7	Seed protein
TRIM24		Intermediate protein	TRIM24	Intermediate protein

X2K

Name	Simple name	P-value	Z-score	Combined score	Targets																							
MYC_CHEA	MYC	{}	2.39E-17	0	0	RPL30	RPL3	RPL32	ATP5C1	RPL8	RPL6	RPL35	RPL23	RPL22	RPL27	RPL12	RPL11	RPS3	RPS2	RPS5	RPSA	FTL	NACA	HSP90AA1	PTP4A1	RPL37A	RPL23A	ARL6IP1
TAF7_ENCODE	TAF7	{}	7.82E-13	0	0	RPL3	RPL32	RPL6	RPL35	RPL27	RPL41	RPLP0	RPL11	RPS3	RPS2	RPSA	FTL	NACA	RPL13A	PTP4A1	RPL37A	RPL23A	RPS16	RPS19	RPS10	RPS13	RPS12	RPS26
PML_ENCODE	PML	{}	1.54E-11	0	0	FTL	ETFA	CES2	CAP1	PABPC1	EEF1G	CFL1	SLC26A2	RPL13A	RPS16	RPS19	RPL36AL	FTH1	RPS12	TXN	RPS29	RPS23	UGDH	PTGES3	RPS4X	UGP2	RPLP0	MDH2
TAF1_ENCODE	TAF1	{}	1.91E-11	0	0	ETFA	BSG	NDUFAB1	EEF1G	CFL1	TPT1	RPS16	RPS19	RPS10	RPS13	RPS12	TXN	RPS26	RPS27	RPS29	RPS20	RPS23	RPS4X	RPLP0	MDH2	COX4I1	PCBP1	HUWE1
KAT2A_ENCODE	KAT2A	{}	3.19E-11	0	0	RPL32	RPL34	RPL6	CFL1	EEF1G	RPL24	RPL27	RPL12	RPL11	PCBP1	RPS3	RPS2	NACA										
NELFE_ENCODE	NELFE	{}	3.19E-08	0	0	RPL3	ARL6IP1	RPS16	BSG	RPS13	RPL41	RPS27	RPS20	RPLP0	PABPC1	RPL12	RPS2	CFL1	RPSA	FTL								
MAX_ENCODE	MAX	{}	5.06E-07	0	0	NACA	ETFA	BSG	PABPC1	PHB2	HADHB	CFL1	SLC26A2	RPL13A	RPL36AL	RPS10	RPS13	RPS12	TXN	RPS20	RPS23	USMG5	PTGES3	ATP5F1	RPLP0	MDH2	FABP5	SLC25A5
MYC_ENCODE	MYC	{}	1.3E-06	0	0	RPLP0	NACA	RPL23A	PCBP1	PABPC1	YWHAE	RPL30	RPL3	RPL32	RPL34	PHB2	RPL6	RPL23	RPL22	RPL24	RPL27	RPL41	RPL12	RPL11	RPS3	RPS2	RPS5	RPSA
ATF2_ENCODE	ATF2	{}	4.33E-05	0	0	FTL	NACA	ETFA	CAP1	EEF1G	PHB2	CFL1	RPL13A	TPT1	RPS16	RPS19	RPS13	RPS12	SUCLG1	RPS26	RPS27	RPS29	RPS20	RPS23	PTGES3	UGP2	RPLP0	MDH2
KLF4_CHEA	KLF4	{}	7.19E-05	0	0	FABP5	LGALS3	PCBP1	ACADM	CRIP1	NDRG1	CA2	RPL8	RPL23	RPL22	RPL41	RPL12	RPS2	CFL1	RPS5	RPSA	RPL13A	KLF4	TPT1	RPS19	TXN	SGK1	F2RL1
TCF3_ENCODE	TCF3	{}	0.000396	0	0	FTL	NACA	COX4I1	HSP90AA1	LGALS1	BSG	PCBP1	CYSTM1	RGS2	PDLIM1	GNG5	RPL3	RPL35	TPT1	RPL36AL	RPS10	PFN1	RPS20	UGP2	ERBB3			
FLI1_ENCODE	FLI1	{}	0.001105	0	0	RPL34	ATP5C1	PHB2	RPL6	RPL27	RPL41	RPS3	CFL1	RPSA	NDUFB9	ARL6IP1	RPS19	RPS13	RPS27	DHRS11	CAP1							
PPARG_CHEA	PPARG	{}	0.0013	0	0	RPL27	GNE	FABP5	ETFA	ACTN4	MGST1	PCBP1	ACADM	ACAA2	HADH	ACAA1	HPGD	MXI1	VIM									
ZMIZ1_ENCODE	ZMIZ1	{}	0.002616	0	0	ATP5C1	TDP2	RPLP0	OAT	FTL	COX4I1	HSP90AA1	SLC25A5	LGALS1	CAP1	CRIP1	ACAA2	HIGD1A	NDRG1	RPS2	ARL6IP1	RPL36AL	TXN	UGDH				
CEBPD_ENCODE	CEBPD	{}	0.003652	0	0	RPL23	RPL24	RPLP0	OAT	CFL1	FTL	RPL13A	TPT1	FTH1	BSG	PFN1	PCBP1	RPS27	RPS20	UGP2	GNG5							
CHD1_ENCODE	CHD1	{}	0.007588	0	0	RPL22	CFL1	FTL	LBR	HSP90AA1	PTP4A1	TST	RPL36AL	PFN1	RPS26	ITM2B	RAC1	UGDH	VIM									
NR2C2_ENCODE	NR2C2	{}	0.010472	0	0	RPL35	RPL22	RPL27	PCBP1	TMA7	RPS26	RPS20	RPS5	NACA														
GABPA_ENCODE	GABPA	{}	0.010888	0	0	NACA	CES2	COX7C	RAC1	CAP1	PHB2	CFL1	RPS16	RPS19	RPL36AL	RPS13	RPS26	RPS27	RPS20	USMG5	TMA7	MDH2	NDUFB9	PCBP1	RPL30	RPL32	RPL34	RPL8
SPI1_ENCODE	SPI1	{}	0.013279	0	0	PKIB	TALDO1	ACTN4	RAC1	CAP1	PABPC1	PHB2	HADHB	CFL1	RPS19	RPL36AL	RPS13	NDUFB9	PCBP1	CTSS	RGS2	ACAA1	RPL23	RPL22	RPL27	RPL41	RPS3	RPL37A
E2F1_CHEA	E2F1	{}	0.014971	0	0	HSP90AA1	RPL23A	BSG	HUWE1	EEF1G	RPL3	RPL41	EPCAM	RPS2	TPT1	ARL6IP1	RPS16	PFN1	MGST1	RPS20	GNB2L1							
ELF1_ENCODE	ELF1	{}	0.016165	0	0	CES2	COX7C	CAP1	PABPC1	PHB2	CFL1	RPL13A	RPS16	RPS19	RPL36AL	RPS10	RPS12	RPS26	RPS27	RPS4X	UGP2	TMA7	MDH2	SLC25A3	COX4I1	NDUFB9	NDUFA1	PCBP1
CEBPB_ENCODE	CEBPB	{}	0.017328	0	0	RPL23	PRR13	RPL13A	PTP4A1	UGP2																		
ZNF384_ENCODE	ZNF384	{}	0.017886	0	0	RPL6	RPL24	RPL27	PRR13	RPL41	EMP1	ATP5A1	SLC25A3	PTP4A1	RPS10	PCBP1	RPS26	NCOA4	MXI1									
ETS1_ENCODE	ETS1	{}	0.023339	0	0	RPL35	FTL	RPL41	RPS4X	RPL23A																		
YY1_ENCODE	YY1	{}	0.027267	0	0	NACA	COX7C	RAC1	NDUFAB1	EEF1G	PHB2	HADHB	RPL13A	FTH1	RPS13	RPS26	RPS27	RPS29	ATP5F1	TMA7	TDP2	RPLP0	SLC25A3	HSP90AA1	NDUFB9	RPL23A	PCBP1	YWHAE

CREB1_ENCODE	CREB1	{}	0.027974	0	0	PKIB	PABPC1	PHB2	RPL13A	TPT1	RPS13	RPS26	RPS27	RPS29	USMG5	PTGES3	HADH	RPLP0	SLC25A3	TP53INP2	RPL23A	PCBP1	CYSTM1	RPL3	RPL34	RPL8	RPL23	RPL24
PPARD_CHEA	PPARD	{}	0.028276	0	0	RPS16	FTH1	HADHB	ACAA2	FTL	COX4I1	ETFA																
ZBTB33_ENCODE	ZBTB33	{}	0.028276	0	0	ATP5C1	RPL23	RPL24	RPL12	RPS2	MDH2	ETFA	RPL23A	RPS19	NCOA4	PTGES3	ATP5F1	EEF1G										
TP63_CHEA	TP63	{}	0.046839	0	0	C19ORF33	FABP5	DSP	TNFSF10	KCTD12	RAC1	HSD17B2	NDRG1	EEF1G	PDLIM1	MXI1	CHGA	S100A14	RPL41	HADHB	CHP2	LBR	KRT8	SGK1	CA12	MFSD4		
FOSL2_ENCODE	FOSL2	{}	0.052409	0	0	CYFIP1	KRT8	EPCAM	GNG5	ERBB3																		
BRCA1_ENCODE	BRCA1	{}	0.066081	0	0	ETFA	CES2	EEF1G	CFL1	TPT1	RPS16	RPS19	RPS26	RPS29	RPS20	RPLP0	MDH2	PCBP1	CYSTM1	NCOA4	RPL34	RPL35	RPL23	RPL22	RPL24	RPL27	RPL41	PRDX6
SPI1_CHEA	SPI1	{}	0.072783	0	0	FTL	NDUFB9	TALDO1	CTSS	EEF1G	RPL8	RPL6	RPL23	RPL27	PRR13	RPL41	RPS3	CFL1	ARL6IP1	RPS19	AKR1B10							
SALL4_CHEA	SALL4	{}	0.07315	0	0	RPL23	RPS20	NCOA4	PTGES3	PTP4A1	PDLIM1	VIM																
SRF_ENCODE	SRF	{}	0.087638	0	0	RPL32	RPL22	RPL24	PFN1	CAP1	CFL1																	
NFE2L2_CHEA	NFE2L2	{}	0.098799	0	0	OAT	FTL	TALDO1	PTTG1IP	CA2	PRR13	PRDX6	PTP4A1	FGL2	KLF4	FTH1	RPS12	TXN	SGK1	MPEG1								
EGR1_CHEA	EGR1	{}	0.105263	0	0	ACTN4	HADHB	HSD17B2	HSPA1A	SLC25A5	TPT1																	
STAT3_CHEA	STAT3	{}	0.109717	0	0	ARL6IP1	EMP1	PABPC1	KLF4																			
SIX5_ENCODE	SIX5	{}	0.147719	0	0	FTL	RPL23A	COX7C	PABPC1	RPL32	RPL8	PHB2	RPL6	RPL12	RPS3	CFL1	RPS13	SUCLG1	RPS26	RPS20								
BCLAF1_ENCODE	BCLAF1	{}	0.159516	0	0	MDH2	FTL	ACADM	CAP1	GNG5	PHB2	RPL41	RPL11	RPS19	RPS13	RPS12	RPS20											
TRIM28_CHEA	TRIM28	{}	0.169102	0	0	KCTD12	HSPA1A	LBR	ERBB3																			
SIN3A_ENCODE	SIN3A	{}	0.177296	0	0	RPLP0	MDH2	PABPC1	NDRG1	YWHAE	RPL30	PHB2	RPL23	RPL24	RPL41	HADHB	RPS2	CFL1	RPS5	USMG5								
EGR1_ENCODE	EGR1	{}	0.183471	0	0	RPS2	PTP4A1	SLC25A5	TPT1	ACADM	USMG5	PABPC1	GNG5															
GATA1_CHEA	GATA1	{}	0.200684	0	0	ETFA	BSG	COX7C	CAP1	NCOA4	CRIP1	LBR	AQP8	FTH1	PYY	ALDH2												
FOXA2_ENCODE	FOXA2	{}	0.221921	0	0	MT2A	KRT8	TSPAN8	SRI	TP53INP2																		
HNF4A_ENCODE	HNF4A	{}	0.226368	0	0	CLDN7	C19ORF33	TDP2	FTL	ETFA	SLC25A5	ACTN4	CES2	PCK1	NCOA4	NDUFAB1	RPL27	PRR13	HADHB	TPT1	UGDH							
SMAD4_CHEA	SMAD4	{}	0.245628	0	0	PKIB	ETFA	LBR	VDAC2	VDAC1	TSPAN8	RGS2	NDRG1															
YY1_CHEA	YY1	{}	0.251585	0	0	RPL34	RPS27	NACA	YWHAE	RPL13A																		
FOXM1_ENCODE	FOXM1	{}	0.25493	0	0	ARL6IP1	LBR																					
NANOG_CHEA	NANOG	{}	0.261121	0	0	PRR13	HSP90AA1	ACTN4	RPS23	NCOA4	SGK1	PDLIM1	VIM															
FOXP2_ENCODE	FOXP2	{}	0.273979	0	0	RPL27	NDRG1	PABPC1																				
CREB1_CHEA	CREB1	{}	0.298993	0	0	OAT	SLC25A3	TP53INP2	PABPC1	MXI1	RPL34	RPL24	RPL13A	PTP4A1	TPT1	RPS19	FTH1	RPS13	RPS27	RPS29	PTGES3	FAU						
BCL3_ENCODE	BCL3	{}	0.303825	0	0	ARL6IP1	PFN1	DHRS11	RPLP0																			
NFYB_ENCODE	NFYB	{}	0.365447	0	0	ETFA	PDCD4	LGALS1	BSG	EEF1G	CFL1	LBR	RPLP0	CYSTM1	ACAA1	RPL35	RPL41	PRDX6	GUCA2A	ARL6IP1	ALDH2	OAT	FTL	TALDO1	HIGD1A	SLC26A2	RPL13A	RPL36AL
SOX2_CHEA	SOX2	{}	0.390104	0	0	FTL	PDLIM1	VIM	RPL3	RPS19	VDAC1	RPS26	RPS23	ABCG2														
UBTF_ENCODE	UBTF	{}	0.392328	0	0	RAC1	MXI1	CFL1	LBR	ALDH9A1	TPT1	RPL36AL	UGDH	COX4I1	TP53INP2	PTTG1IP	PCBP1	CYSTM1	CAPN2	CYFIP1	PTP4A1	VDAC1	ITM2B					
SP2_ENCODE	SP2	{}	0.430016	0	0	RPLP0	SLC25A3	NDUFAB1	PRR13	RPL41	RPL11	SLC26A2	RPL37A	RPL36AL	RPS20	UGP2												

NFYA_ENCODE	NFYA	{}	0.431621	0	0	OAT	FTL	ETFA	PDCD4	LGALS1	HIGD1A	SLC26A2	LBR	RPL13A	RPL36AL	USMG5	UGP2	TMA7	RPLP0	SLC25A3	RPL23A	ACAA1	RPL27	PRR13	RPS2	RPS5	RPSA	PTP4A1
ERG_CHEA	ERG	{}	0.432739	0	0	RPL22	NDRG1																					
TCF3_CHEA	TCF3	{}	0.445679	0	0	FTL	HSP90AA1	ACTN4	PDLIM1	MXI1	VIM	RPS19	RPS26	RPS29	RPS23	SGK1												
FOS_ENCODE	FOS	{}	0.47109	0	0	PRR13	RPLP0	SLC26A2	LBR	CAPN2	UGP2	VIM																
BHLHE40_ENCODE	BHLHE40	{}	0.473397	0	0	BSG	PFN1	FTL	SLC25A5																			
IRF1_ENCODE	IRF1	{}	0.502114	0	0	MT2A	RPS12	TDP2																				
GATA2_CHEA	GATA2	{}	0.528076	0	0	PDCD4	TNFSF10	ACTN4	PTTG1IP	NCOA4	CRIP1	CAPN2	MXI1															
HDAC2_ENCODE	HDAC2	{}	0.533385	0	0	RPS19	ERBB3																					
NFIC_ENCODE	NFIC	{}	0.54146	0	0	MT2A	ACAA2	CFL1																				
VDR_CHEA	VDR	{}	0.54147	0	0	PABPC1																						
RELA_ENCODE	RELA	{}	0.547839	0	0	RPL37A	RPS19	PFN1	TXN	PCBP1																		
CTCF_ENCODE	CTCF	{}	0.554608	0	0	GCG	NDUFAB1	CA2	SLC26A2	PARM1	TPT1	RPS19	RPS26	RPS29	RPS20	RPS4X	RPLP0	SLC25A5	NCOA4	RPL32	RPL27	RPL11	ERBB3					
STAT3_ENCODE	STAT3	{}	0.60757	0	0	RPL24	RPL27	HADHB	PTP4A1	PDCD4	MT2A	TXN																
TP53_CHEA	TP53	{}	0.631549	0	0	RPS19	PTP4A1	VIM																				
RUNX1_CHEA	RUNX1	{}	0.669848	0	0	TDP2	FTL	SRI	LGALS3	LGALS2	LGALS1	PTTG1IP	ACADM	NCOA4	LBR	FTH1	SUCLG1											
STAT5A_ENCODE	STAT5A	{}	0.686988	0	0	FTH1	TXN																					
ATF3_ENCODE	ATF3	{}	0.688375	0	0	RPL23																						
ZKSCAN1_ENCODE	ZKSCAN1	{}	0.706836	0	0	HIGD1A																						
IRF8_CHEA	IRF8	{}	0.709806	0	0	CTSS																						
RCOR1_ENCODE	RCOR1	{}	0.721199	0	0	CYFIP1	TST	LGALS1	RPS26	RAC1	NDUFAB1																	
NRF1_ENCODE	NRF1	{}	0.731062	0	0	PKIB	PDCD4	BSG	ACTN4	EEF1G	GNG5	RPL13A	RPS16	RPS12	RPS29	PTGES3	TDP2	PTTG1IP	RPS5	RPL37A	PFN1	FAU						
POU5F1_CHEA	POU5F1	{}	0.745035	0	0	SGK1	HSP90AA1																					
REST_ENCODE	REST	{}	0.748819	0	0	CHGB	CHGA	SST																				
ESR1_CHEA	ESR1	{}	0.792688	0	0	CA12																						
E2F6_ENCODE	E2F6	{}	0.805972	0	0	ETFA	PDCD4	BSG	LBR	TPT1	RPS13	RPS20	PTTG1IP	YWHAE	PFN1	ATP5A1	TALDO1	KCTD12	EPCAM	SLC26A2	RPL36AL	SUCLG1	PTGES3	HADH	ETHE1	TMA7	TDP2	FABP5
MYOD1_ENCODE	MYOD1	{}	0.816586	0	0	CMBL																						
PBX3_ENCODE	PBX3	{}	0.836917	0	0	RPLP0	FTL	NACA	PRR13	RPL41	SLC26A2	RPL36AL	PFN1	RPS26	UGP2													
TCF7L2_ENCODE	TCF7L2	{}	0.846129	0	0	FTL	DSP	PCBP1	RPS27																			
USF2_ENCODE	USF2	{}	0.864136	0	0	BSG	PCBP1	RPL23	CFL1	SLC26A2	RPL13A	ATP5F1																
AR_CHEA	AR	{}	0.872716	0	0	FABP5	ETFA	LGALS3	SPINK5	NDRG1	CMBL	CA12	UGP2															
FOXA1_ENCODE	FOXA1	{}	0.876895	0	0	KRT8																						

EZH2_CHEA	EZH2	{}	0.911311	0	0	ITLN1																						
RFX5_ENCODE	RFX5	{}	0.92507	0	0	PTP4A1	PDCD4	HIGD1A																				
SP1_ENCODE	SP1	{}	0.931022	0	0	RPLP0	RPS2	ARL6IP1	RPS19																			
E2F4_ENCODE	E2F4	{}	0.932415	0	0	RPS16	BSG	RPS20	PTGES3																			
RAD21_ENCODE	RAD21	{}	0.948027	0	0	SLC25A5	NCOA4	GCG	CA2	RPL27	SLC26A2	PARM1	HEPACAM2															
REST_CHEA	REST	{}	0.952251	0	0	SST	LGALS3	ACTN4	CHGA	CHGB	PARM1	SGK1	GNB2L1															
SMC3_ENCODE	SMC3	{}	0.959232	0	0	GCG	S100A14	RPL11	SLC26A2	ITLN1	HEPACAM2	ERBB3																
ZBTB7A_ENCODE	ZBTB7A	{}	0.965736	0	0	FTL	PDCD4	LGALS1	GNG5	TST	FTH1	UGP2	TP53INP2	ACADM	NDRG1	YWHAE	RPL12	PFN1	ALDH2	ERBB3								
IRF3_ENCODE	IRF3	{}	0.966092	0	0	RPLP0	SLC26A2	UGP2																				
EZH2_ENCODE	EZH2	{}	0.968634	0	0	CHP2																						
USF1_ENCODE	USF1	{}	0.979595	0	0	BSG	PCBP1	RPL23	PRDX6	CFL1	SLC26A2	RPL13A	ATP5F1															
SUZ12_CHEA	SUZ12	{}	0.99961	0	0	PKIB	MFSD4	RGS2	NDRG1	CHGB	HPGD																	
CSNK2A1	4.64E-18	0	0	ATF2	SPI1	DAXX	CSNK2A1	PPARGC1A	JUN	NELFE	FOS	SIRT1	YY1	PIM1	MSH2	MAX	PIN1	RELA	CCND1	VDR	TCF3	VHL	TAF7	RB1	TRRAP	MYC	MYB	PRKDC
CDK4	1.73E-17	0	0	RB1	ATF2	TRRAP	CCND1	MYC	MYB	SKP2	MAX	SMAD2	JUN	SMAD3	SP1	CDK2	BCL2	TCF3	RBL2	RBL1	CEBPA	CEBPB	CDKN2A					
MAPK14	3.30E-17	0	0	ATF2	PPARGC1A	JUN	NELFE	FOS	YY1	PIM1	MSH2	SP1	RELA	CCND1	TCF3	VHL	RB1	TRRAP	HNRNPU	MYC	MYB	PRKDC	HIF1A	NCOR1	CDKN2A	CDK2	BCL2	CEBPA
CDK1	2.03E-16	0	0	ATF2	CSNK2A1	GATA2	PPARGC1A	JUN	NELFE	FOS	SIRT1	SIRT2	CCNT1	PIM1	MSH2	SP1	E2F4	RELA	CCND1	TCF3	VHL	TAF1	RB1	TRRAP	HNRNPU	MYC	MYB	PRKDC
MAPK1	1.03E-13	0	0	RB1	ATF2	MYC	MYB	CSNK2A1	HIF1A	JUN	FOS	CDKN2A	PIM1	SP1	CDK2	BCL2	CEBPA	CEBPB	KLF4	PML	SKP2	HIST1H3A	TUBB	RELA	CCND1	SMAD2	SMAD4	SMAD3
ERK1	1.11E-11	0	0	ATF2	KAT5	MYC	PRKCD	HIF1A	SMAD2	JUN	SMAD4	SMAD3	SP1	BCL2	TCF3	FOS	CEBPB	RELA	MAPK8									
GSK3B	7.80E-11	0	0	ATF2	GATA2	JUN	FOS	MSH2	SP1	TRIM24	RELA	TCF3	NCOR1	CDKN2A	BCL2	CEBPA	CEBPB	PML	KAT5	TUBB	RBL2	RBL1	MAPK8	MAPK1	DAXX	SIRT1	CCNT1	PIM1
JNK1	1.51E-10	0	0	ATF2	MYC	FOS	SIRT1	HIF1A	RELA	MAPK8	SMAD2	JUN	SMAD3	SP1	BCL2													
HIPK2	1.66E-10	0	0	MYC	MYB	CTBP1	PRKDC	HIF1A	SMAD2	SMAD3	BCL2	SIRT1	NCOR1	CEBPB	RELA	MAPK8	PML											
MAPK3	2.09E-10	0	0	ATF2	MYC	MYB	JUN	FOS	CCND1	SMAD2	SMAD4	SMAD3	SP1	CDK2	BCL2	CEBPA	CEBPB	MAPK8	E2F4	MAPK3	KLF4	PML						
CDK2	2.42E-10	0	0	ATF2	JUN	FOS	MSH2	SP1	E2F4	TCF3	HNRNPU	PRKDC	CDKN2A	BCL2	CEBPB	HDAC2	PML	KAT5	TUBB	H2AFX	RBL2	RBL1	MAPK8	MAPK1	DAXX	NELFE	SIRT2	CCNT1
AKT1	3.50E-10	0	0	TRRAP	MYC	CSNK2A1	GATA2	HIST1H3A	PPARGC1A	JUN	FOS	NCOR1	CDKN2A	GTF2F1	PIAS2	SMAD4	SMAD3	SP1	CDK2	BCL2	TCF3	MAPK1						
ERK2	6.87E-10	0	0	RB1	ATF2	MYC	MYB	SMAD2	JUN	SMAD4	SMAD3	SP1	BCL2	TCF3	FOS	CEBPB	RELA											
MAPK8	1.39E-09	0	0	ATF2	MYC	MYB	CTBP1	JUN	TUBB	FOS	SIRT1	CDKN2A	CCND1	SP1	BCL2	H2AFX	RBL2	MAPK1	KLF4									
DNAPK	1.47E-09	0	0	TRRAP	KAT5	PRKCD	PRKDC	SMAD2	JUN	SMAD4	SP1	TCF3	H2AFX	RELA	MAPK8													
JNK2	2.81E-09	0	0	RB1	ATF2	PRKDC	RELA	MYC	SMAD2	JUN	FOS																	
CK2ALPHA	1.72E-08	0	0	RB1	ATF2	SPI1	MYC	MYB	JUN	RELA	YY1	MAX	VDR	SP1	HDAC2	HDAC3	PML	TAF1										
ABL1	1.36E-07	0	0	RB1	SPI1	CCND1	KAT5	MYC	PRKCD	JUN	BCL2	FOS	DDB1	CEBPB	HDAC2													
ATM	1.65E-07	0	0	RB1	ATF2	HNRNPU	KAT5	DAXX	PRKDC	HIF1A	MSH2	SP1	CDK2	H2AFX	RBL2	RBL1	VHL											

RPS6KA3	1.86E-07	0	0	TRRAP	MYC	MYB	PRKDC	HIST1H3A	JUN	TUBB	FOS	KAT2B	CDKN2A	CCND1	SMAD3	BCL2	RBL2	MAPK3									
CDC2	9.51E-07	0	0	RB1	KAT5	MYC	SKP2	CSNK2A1	PRKDC	JUN	FOS	NCOR1	RELA	CCND1	BCL2	RBL2	RBL1										
RAF1	9.85E-07	0	0	RB1	MYC	MYB	PRKCD	BCL2	PIN1	FOS	RBL2	MAPK1	MAPK3														
CSNK2A2	1.74E-06	0	0	TRRAP	CCND1	MYC	JUN	CDK2	BCL2	NELFE	TUBB	FOS	RPL11														
CK2-A	6.95E-06	0	0	SPI1	MYB	HDAC2	MAX	RELA	JUN	VDR																	
PRKDC	1.8E-05	0	0	TRRAP	HNRNPU	MYC	SKP2	PRKDC	H2AFX	RELA	MAPK8																
GSK3BETA	1.83E-05	0	0	CCND1	MYC	PPARGC1A	JUN	SMAD3	BCL2	PIN1	RBL2	CEBPB															
PRKCA	2.25E-05	0	0	ATF2	SPI1	JUN	FOS	PIM1	RELA	MNT	CCND1	VDR	RB1	MYC	MYB	NCOR1	CDKN2A	CDK9	BCL2	CEBPB	KLF4	HIST1H3A	TUBB	SMAD3	RBL2	MAPK1	
MAPK11	3.43E-05	0	0	ATF2	PPARGC1A	JUN	BCL2	FOS	CEBPB	PML	MAPK11																
ATR	3.63E-05	0	0	DAXX	MSH2	PRKDC	CDK9	SP1	H2AFX	CDKN2A	PML																
PRKACA	5.36E-05	0	0	SPI1	PPARGC1A	JUN	FOS	DDB1	PIN1	RELA	CCND1	VDR	RB1	MYC	MYB	HIF1A	NCOR1	CDKN2A	BCL2	CEBPB	HDAC7	KLF4	TUBB	SMAD4	SMAD3	RBL2	MAPK1
SRC	6.1E-05	0	0	RB1	SPI1	MYC	MYB	PRKCD	JUN	FOS	BCL2	CEBPB	E2F4	KLF4	TUBB	CCND1	SMAD3	MAPK1									
MAP3K7	6.9E-05	0	0	HNRNPU	RUVBL2	SMAD3	HIST1H4A	TUBB	HDAC7	E2F4																	
MAP2K1	0.000106	0	0	CCND1	MYC	FOS	MAPK8	MAPK1	MAPK3																		
PRKCD	0.00011	0	0	PRKCD	PRKDC	HIST1H3A	JUN	FOS	MAPK8	MAPK1	MAPK3																
MAP2K3	0.000157	0	0	MAPK3	SMAD2	SMAD4	SMAD3	MAPK11																			
GSK-3-B	0.000248	0	0	CEBPB	RELA	CCND1	MYC																				
MAPK10	0.000283	0	0	MYC	FOS	JUN	CDKN2A	BCL2																			
MAPK9	0.000315	0	0	ATF2	CCND1	MYC	H2AFX	FOS	JUN																		
CHEK1	0.000336	0	0	RB1	MYC	SKP2	HIST1H3A	NELFE	TUBB	FOS	H2AFX	E2F4															
CDK9	0.000486	0	0	RB1	CCNT1	SMAD3	CDK9																				
TGFBR2	0.000898	0	0	MYC	SMAD2	SMAD3	PML	SP1																			
RPS6KA1	0.001403	0	0	FOS	CEBPB	MAPK1	MAPK3	JUN																			
PKBALPHA	0.001439	0	0	MYC	SKP2	CTBP1	PRKDC	PPARGC1A	RELA	SMAD2	SMAD3																
PKCEPSILON	0.001479	0	0	PRKCD	MAPK1	MAPK3	SP1	BCL2																			
PRKCZ	0.001638	0	0	RELA	MAPK1	MAPK3	SP1	PIN1																			
CHUK	0.001723	0	0	CCND1	MYC	TUBB	NCOR1	RELA																			
GSK3A	0.00185	0	0	CCND1	MYC	CEBPA	CEBPB																				
IKK-A	0.001922	0	0	CCND1	RELA																						
PKCTHETA	0.001981	0	0	PRKCD	MAPK8	MAPK1	MAPK3																				
IKBKB	0.002193	0	0	HNRNPU	TUBB	FOS	RELA	CDKN2A																			

PBK	0.002224	0	0	HIST1H3A	E2F4	JUN			
PIM1	0.002261	0	0	MYC	SKP2	RELA	HIST1H3A		
VRK1	0.00274	0	0	ATF2	HIST1H3A	JUN			
MELK	0.003023	0	0	SMAD2	SMAD4	SMAD3			
RSK3	0.003253	0	0	MAPK1	MAPK3				
CDK6	0.003642	0	0	RB1	CEBPA	RBL2			
CSNK1E	0.003653	0	0	CCND1	MYC	TUBB	CDKN2A		
PKD1	0.003653	0	0	MYB	HDAC7	MAPK8	JUN		
CK2A2	0.00386	0	0	MYC	HDAC2	RELA	JUN		
DNA-PK	0.00404	0	0	JUN	PRKDC				
CDK3	0.004333	0	0	RB1	JUN	CDK2			
AURKB	0.004647	0	0	RB1	YY1	PRKDC	HIST1H3A	PIN1	NELFE
LYN	0.005085	0	0	SPI1	YY1	CCND1	MYC	CSNK2A1	PRKCD
RET	0.00686	0	0	MAPK8	MAPK1	MAPK3			
CDK7	0.007315	0	0	GTF2F1	CDK9	CDK2	TAF3		
PAK2	0.007644	0	0	MYC	SMAD2	JUN	HIST1H4A		
PLK3	0.007861	0	0	ATF2	HIF1A	JUN			
BRD2	0.007965	0	0	RB1	RBL2				
ADRBK1	0.007965	0	0	PRKCD	MAPK3				
TBK1	0.008688	0	0	RUVBL2	TUBB	RPL11	RELA		
IKBKE	0.009056	0	0	SKP1	TUBB	CUL1	RELA		
ACTR2B	0.010372	0	0	SMAD2	SMAD3				
PKCIOTA	0.010722	0	0	RELA	CCND1	RUVBL2			
MAPK12	0.011048	0	0	ATF2	CCND1	FOS	JUN		
EGFR	0.012125	0	0	CCND1	PRKCD	PRKDC	HIST1H3A	JUN	FOS
CHEK2	0.014115	0	0	RB1	VHL	PML			
GSK3ALPHA	0.015623	0	0	CCND1	MYC	JUN			
JAK2	0.01641	0	0	HIST1H3A	MAPK1	MAPK3			
CSNK2B	0.019225	0	0	FOS	RUVBL2				
CDKN1B	0.019467	0	0	CDK2					
BAZ1B	0.019467	0	0	H2AFX					

TRIM33	0.019467	0	0	SMAD4				
HASPIN	0.019467	0	0	HIST1H3A				
PCNA	0.019467	0	0	CDK2				
TGM2	0.019467	0	0	HIST1H3A				
EP400	0.019467	0	0	TRRAP				
LYK5	0.019467	0	0	SMAD4				
HIPK1	0.022684	0	0	DAXX	MYB			
RPS6KA4	0.026378	0	0	RELA	HIST1H3A			
MAP3K8	0.028651	0	0	PIM1	MAPK1	MAPK3		
GTF2F1	0.02906	0	0	GTF2F1				
PRKCQ	0.029748	0	0	SPI1	SIRT2	HIST1H3A		
MOK	0.030297	0	0	MYC	JUN			
PAK1	0.03126	0	0	CCND1	PRKCD	CTBP1	HIST1H3A	
CDK8	0.034433	0	0	HIST1H3A	SMAD3			
UHMK1	0.036578	0	0	SMAD2	SMAD4			
MAPK7	0.036578	0	0	FOS	PML			
MAP3K20	0.03856	0	0	HIST1H3A				
EEF2K	0.038774	0	0	MAPK8	MAPK11			
MAPK13	0.039307	0	0	ATF2	FOS	JUN		
MAP3K14	0.040003	0	0	HNRNPU	RUVBL2	TUBB	RPL11	
BARK1	0.043249	0	0	PRKCD	MAPK3	PIN1		
DAPK1	0.043311	0	0	MAPK3	PIN1			
FYN	0.044112	0	0	SPI1	PRKCD	FOS	SIRT2	CCND1
INSR	0.044605	0	0	CCND1	FOS	JUN		
NPM/ALK	0.047969	0	0	MSH2				
AURORAA	0.050241	0	0	CCND1	NCOR1	HDAC3		
MAP3K5	0.050467	0	0	MAPK8	DAXX			
AURKA	0.051702	0	0	HIST1H3A	VHL	PIN1		
PRKCB	0.05614	0	0	ATF2	HIST1H3A	JUN	CCND1	BCL2
CK1ALPHA	0.056208	0	0	RB1	FOS	JUN		
DYRK2	0.060613	0	0	MYC	JUN			

MAP3K1	0.062473	0	0	RUVBL2	HIST1H4A	TUBB	DDB1	
PDGFRBETA	0.06325	0	0	MAX	PRKCD			
MAP2K2	0.065926	0	0	MAPK1	MAPK3			
DYRK1A	0.065926	0	0	CCND1	SIRT1			
RPS6KA5	0.065926	0	0	RELA	HIST1H3A			
NIK	0.066513	0	0	CEBPB				
HSPB8	0.066513	0	0	MAPK3				
RIPK1	0.06864	0	0	TUBB	PML			
PKC-B	0.075652	0	0	ATF2				
RSK-1	0.075652	0	0	CEBPB				
NLK	0.076995	0	0	MYB	SMAD4			
IKKBETA	0.079849	0	0	RELA	FOS			
LCK	0.084656	0	0	SPI1	PRKCD	MAPK1	MAPK3	
PRKCI	0.085654	0	0	DDB1	SP1			
PRKAA1	0.090696	0	0	RB1	CTBP1	SIRT2		
RSK-2	0.093664	0	0	FOS				
MAP2K4	0.094592	0	0	DAXX	MAPK8			
RIPK3	0.100434	0	0	RUVBL2	DAXX	TUBB		
MTOR	0.102431	0	0	MYC	JUN	TAF3		
PKC-Z	0.10254	0	0	RELA				
CAMKIV	0.10254	0	0	RELA				
CAMKIIBETA	0.103786	0	0	FOS	PPARGC1A			
JNK3	0.11133	0	0	JUN				
MAP3K3	0.115025	0	0	RUVBL2	HIST1H4A	TUBB	DDB1	
CDK5	0.116657	0	0	RB1	MYC	HIF1A	BCL2	
TRKB	0.120034	0	0	FOS				
AKT2	0.122854	0	0	HIST1H3A	PPARGC1A			
CAMK2A	0.129773	0	0	CCND1	MYC	SMAD2		
PLK1	0.142998	0	0	HNRNPU	MYC	CCNT1	YY1	PIN1
PKCALPHA	0.143187	0	0	MYC	PRKCD	BCL2		
SRM	0.145647	0	0	RUVBL2				

BTK	0.146068	0	0	FOS	JUN
RPS6KB1	0.149457	0	0	PPARGC1A	CDKN2A
TAF1	0.15402	0	0	TAF7	
PRKD1	0.166626	0	0	HDAC7	JUN
SRPK3	0.170524	0	0	SIRT2	
PTK7	0.170524	0	0	DDB1	
PDK-1	0.178656	0	0	PRKCD	
TIE1	0.178656	0	0	MAPK11	
PKCETA	0.178656	0	0	PRKCD	
MINK1	0.178656	0	0	SMAD2	
CDK20	0.186709	0	0	CDK2	
CDK18	0.186709	0	0	SIRT2	
DMPK	0.186709	0	0	SP1	
PKN3	0.186709	0	0	GATA2	
PKA-A	0.186709	0	0	RELA	
NTRK3	0.186709	0	0	FOS	
MARK4	0.186709	0	0	MAPK11	
PRKCE	0.191196	0	0	ATF2	MAPK3
PIM3	0.194684	0	0	PML	
MAP3K4	0.194684	0	0	TRRAP	
TLK2	0.194684	0	0	ASF1A	
GSG2	0.194684	0	0	HIST1H3A	
MAPK15	0.194684	0	0	JUN	
CSNK1A1	0.194747	0	0	RELA	VHL
MAP3K6	0.202583	0	0	MAPK8	
MAP3K10	0.202583	0	0	TCF3	
TLK1	0.202583	0	0	HIST1H3A	
MAPK6	0.202583	0	0	DDB1	
WEE1	0.202583	0	0	CDK2	
FRK	0.210404	0	0	FOS	
IKKALPHA	0.210404	0	0	RELA	

CSNK1G1	0.21 0404	0	0	RELA	
MAP3K11	0.21 0404	0	0	PIN1	
DYRK3	0.21 0404	0	0	SIRT1	
AURKC	0.21 0404	0	0	HIST1H3A	
PAK5	0.21 815	0	0	TCF3	
MYLK	0.22 5821	0	0	MAPK 3	
NEK1	0.22 5821	0	0	VHL	
PIM2	0.22 5821	0	0	MYC	
ICK	0.22 5821	0	0	SIRT2	
MARK3	0.22 5821	0	0	DDB1	
RPS6KA2	0.23 3418	0	0	MAPK 3	
MKNK1	0.23 3418	0	0	MAPK 3	
AKT3	0.24 8391	0	0	PRKC D	
PKBGAM MA	0.24 8391	0	0	RELA	
TNK2	0.24 8391	0	0	HIST1H4A	
DYRK1B	0.25 5769	0	0	CCND 1	
BMX	0.25 5769	0	0	PIM1	
LKB1	0.25 5769	0	0	PPARGC1A	
CAMK4	0.25 5769	0	0	RELA	
STK3	0.25 5769	0	0	HIST1H3A	
GRK2	0.25 5769	0	0	SMAD 2	
PRKACB	0.26 3075	0	0	FOS	
JAK1	0.26 3075	0	0	HIST1H3A	
CAMK2D	0.26 3075	0	0	SMAD 4	
PKN1	0.27 0311	0	0	HIST1H3A	
NEK6	0.27 0311	0	0	RBL2	
CAMK1	0.27 0311	0	0	HDAC 7	
TTK	0.27 4252	0	0	CDK2	E2F4
MAPKAPK 5	0.27 7476	0	0	MAPK 11	
YES1	0.27 7476	0	0	PRKC D	
STK4	0.28 4572	0	0	H2AF X	

BMPR1B	0.284572	0	0	SMAD2	
ZAP70	0.284572	0	0	MAPK11	
CSK	0.291599	0	0	PML	
PDGFRB	0.291599	0	0	PRKCD	
ERBB2	0.299692	0	0	CCND1	NCOR1
PKG1CGKI	0.305449	0	0	SP1	
PKAALPHA	0.31056	0	0	FOS	RELA
ALK	0.312274	0	0	RELA	
ARAF	0.319032	0	0	PRKCD	
MARK2	0.319032	0	0	HDAC7	
STK11	0.332353	0	0	SMAD4	
MAP2K7	0.332353	0	0	MAPK8	
PRKAA2	0.332353	0	0	PPARGC1A	
HCK	0.332353	0	0	SPI1	
ABL2	0.338916	0	0	CEBPB	
RIPK2	0.358227	0	0	TUBB	
MAP2K6	0.358227	0	0	MAPK11	
CSNK1D	0.364539	0	0	HIF1A	
PAK3	0.37079	0	0	CCND1	
PKBBETA	0.37698	0	0	RELA	
IGF1R	0.41866	0	0	FOS	
ABL	0.435669	0	0	CDK2	
PKAGAMMA	0.441228	0	0	RELA	
CAMKIIALPHA	0.452186	0	0	CEBPB	
TGFBR1	0.452186	0	0	SMAD2	
SGK1	0.478659	0	0	MAPK1	
PDHK1	0.503869	0	0	PRKCD	
ROCK1	0.513613	0	0	HIST1H3A	
PKCGAMMA	0.518414	0	0	PRKCD	
AURORAB	0.537153	0	0	RB1	

KEA

name	pvalue	zscore	combinedScore	enrichedSubstrates																	
CSNK2A1	4.64E-18	0	0	ATF2	SPI1	DAXX	CSNK2A1	PPARGC1A	JUN	NELFE	FOS	SIRT1	YY1	PIM1	MSH2	MAX	PIN1	RELA	CCND1	VDR	TCF3
CDK4	1.73E-17	0	0	RB1	ATF2	TRRAP	CCND1	MYC	MYB	SKP2	MAX	SMAD2	JUN	SMAD3	SP1	CDK2	BCL2	TCF3	RBL2	RBL1	CEBPA
MAPK14	3.30E-17	0	0	ATF2	PPARGC1A	JUN	NELFE	FOS	YY1	PIM1	MSH2	SP1	RELA	CCND1	TCF3	VHL	RB1	TRRAP	HNRNPU	MYC	MYB
CDK1	2.03E-16	0	0	ATF2	CSNK2A1	GATA2	PPARGC1A	JUN	NELFE	FOS	SIRT1	SIRT2	CCNT1	PIM1	MSH2	SP1	E2F4	RELA	CCND1	TCF3	VHL
MAPK1	1.03E-13	0	0	RB1	ATF2	MYC	MYB	CSNK2A1	HIF1A	JUN	FOS	CDKN2A	PIM1	SP1	CDK2	BCL2	CEBPA	CEBPB	KLF4	PML	SKP2
ERK1	1.11E-11	0	0	ATF2	KAT5	MYC	PRKCD	HIF1A	SMAD2	JUN	SMAD4	SMAD3	SP1	BCL2	TCF3	FOS	CEBPB	RELA	MAPK8		
GSK3B	7.80E-11	0	0	ATF2	GATA2	JUN	FOS	MSH2	SP1	TRIM24	RELA	TCF3	NCOR1	CDKN2A	BCL2	CEBPA	CEBPB	PML	KAT5	TUBB	RBL2
JNK1	1.51E-10	0	0	ATF2	MYC	FOS	SIRT1	HIF1A	RELA	MAPK8	SMAD2	JUN	SMAD3	SP1	BCL2						
HIPK2	1.66E-10	0	0	MYC	MYB	CTBP1	PRKDC	HIF1A	SMAD2	SMAD3	BCL2	SIRT1	NCOR1	CEBPB	RELA	MAPK8	PML				
MAPK3	2.09E-10	0	0	ATF2	MYC	MYB	JUN	FOS	CCND1	SMAD2	SMAD4	SMAD3	SP1	CDK2	BCL2	CEBPA	CEBPB	MAPK8	E2F4	MAPK3	KLF4
CDK2	2.42E-10	0	0	ATF2	JUN	FOS	MSH2	SP1	E2F4	TCF3	HNRNPU	PRKDC	CDKN2A	BCL2	CEBPB	HDAC2	PML	KAT5	TUBB	H2AFX	RBL2
AKT1	3.50E-10	0	0	TRRAP	MYC	CSNK2A1	GATA2	HIST1H3A	PPARGC1A	JUN	FOS	NCOR1	CDKN2A	GTF2F1	PIAS2	SMAD4	SMAD3	SP1	CDK2	BCL2	TCF3
ERK2	6.87E-10	0	0	RB1	ATF2	MYC	MYB	SMAD2	JUN	SMAD4	SMAD3	SP1	BCL2	TCF3	FOS	CEBPB	RELA				
MAPK8	1.39E-09	0	0	ATF2	MYC	MYB	CTBP1	JUN	TUBB	FOS	SIRT1	CDKN2A	CCND1	SP1	BCL2	H2AFX	RBL2	MAPK1	KLF4		
DNAPK	1.47E-09	0	0	TRRAP	KAT5	PRKCD	PRKDC	SMAD2	JUN	SMAD4	SP1	TCF3	H2AFX	RELA	MAPK8						
JNK2	2.81E-09	0	0	RB1	ATF2	PRKDC	RELA	MYC	SMAD2	JUN	FOS										
CK2ALPHA	1.72E-08	0	0	RB1	ATF2	SPI1	MYC	MYB	JUN	RELA	YY1	MAX	VDR	SP1	HDAC2	HDAC3	PML	TAF1			
ABL1	1.36E-07	0	0	RB1	SPI1	CCND1	KAT5	MYC	PRKCD	JUN	BCL2	FOS	DDB1	CEBPB	HDAC2						
ATM	1.65E-07	0	0	RB1	ATF2	HNRNPU	KAT5	DAXX	PRKDC	HIF1A	MSH2	SP1	CDK2	H2AFX	RBL2	RBL1	VHL				
RPS6KA3	1.86E-07	0	0	TRRAP	MYC	MYB	PRKDC	HIST1H3A	JUN	TUBB	FOS	KAT2B	CDKN2A	CCND1	SMAD3	BCL2	RBL2	MAPK3			
CDC2	9.51E-07	0	0	RB1	KAT5	MYC	SKP2	CSNK2A1	PRKDC	JUN	FOS	NCOR1	RELA	CCND1	BCL2	RBL2	RBL1				
RAF1	9.85E-07	0	0	RB1	MYC	MYB	PRKCD	BCL2	PIN1	FOS	RBL2	MAPK1	MAPK3								
CSNK2A2	1.74E-06	0	0	TRRAP	CCND1	MYC	JUN	CDK2	BCL2	NELFE	TUBB	FOS	RPL11								
CK2-A	6.95E-06	0	0	SPI1	MYB	HDAC2	MAX	RELA	JUN	VDR											
PRKDC	1.8E-05	0	0	TRRAP	HNRNPU	MYC	SKP2	PRKDC	H2AFX	RELA	MAPK8										
GSK3BETA	1.83E-05	0	0	CCND1	MYC	PPARGC1A	JUN	SMAD3	BCL2	PIN1	RBL2	CEBPB									
PRKCA	2.25E-05	0	0	ATF2	SPI1	JUN	FOS	PIM1	RELA	MNT	CCND1	VDR	RB1	MYC	MYB	NCOR1	CDKN2A	CDK9	BCL2	CEBPB	KLF4
MAPK11	3.43E-05	0	0	ATF2	PPARGC1A	JUN	BCL2	FOS	CEBPB	PML	MAPK11										
ATR	3.63E-05	0	0	DAXX	MSH2	PRKDC	CDK9	SP1	H2AFX	CDKN2A	PML										

PRKACA	5.36E-05	0	0	SPI1	PPARGC1A	JUN	FOS	DDB1	PIN1	RELA	CCND1	VDR	RB1	MYC	MYB	HIF1A	NCOR1	CDKN2A	BCL2	CEBPB	HDAC7
SRC	6.1E-05	0	0	RB1	SPI1	MYC	MYB	PRKCD	JUN	FOS	BCL2	CEBPB	E2F4	KLF4	TUBB	CCND1	SMAD3	MAPK1			
MAP3K7	6.9E-05	0	0	HNRNPU	RUVBL2	SMAD3	HIST1H4A	TUBB	HDAC7	E2F4											
MAP2K1	0.000106	0	0	CCND1	MYC	FOS	MAPK8	MAPK1	MAPK3												
PRKCD	0.00011	0	0	PRKCD	PRKDC	HIST1H3A	JUN	FOS	MAPK8	MAPK1	MAPK3										
MAP2K3	0.000157	0	0	MAPK3	SMAD2	SMAD4	SMAD3	MAPK11													
GSK-3-B	0.000248	0	0	CEBPB	RELA	CCND1	MYC														
MAPK10	0.000283	0	0	MYC	FOS	JUN	CDKN2A	BCL2													
MAPK9	0.000315	0	0	ATF2	CCND1	MYC	H2AFX	FOS	JUN												
CHEK1	0.000336	0	0	RB1	MYC	SKP2	HIST1H3A	NELFE	TUBB	FOS	H2AFX	E2F4									
CDK9	0.000486	0	0	RB1	CCNT1	SMAD3	CDK9														
TGFBR2	0.000898	0	0	MYC	SMAD2	SMAD3	PML	SP1													
RPS6KA1	0.001403	0	0	FOS	CEBPB	MAPK1	MAPK3	JUN													
PKBALPHA	0.001439	0	0	MYC	SKP2	CTBP1	PRKDC	PPARGC1A	RELA	SMAD2	SMAD3										
PKCEPSILON	0.001479	0	0	PRKCD	MAPK1	MAPK3	SP1	BCL2													
PRKCZ	0.001638	0	0	RELA	MAPK1	MAPK3	SP1	PIN1													
CHUK	0.001723	0	0	CCND1	MYC	TUBB	NCOR1	RELA													
GSK3A	0.00185	0	0	CCND1	MYC	CEBPA	CEBPB														
IKK-A	0.001922	0	0	CCND1	RELA																
PKCTHETA	0.001981	0	0	PRKCD	MAPK8	MAPK1	MAPK3														
IKBKB	0.002193	0	0	HNRNPU	TUBB	FOS	RELA	CDKN2A													
PBK	0.002224	0	0	HIST1H3A	E2F4	JUN															
PIM1	0.002261	0	0	MYC	SKP2	RELA	HIST1H3A														
VRK1	0.00274	0	0	ATF2	HIST1H3A	JUN															
MELK	0.003023	0	0	SMAD2	SMAD4	SMAD3															
RSK3	0.003253	0	0	MAPK1	MAPK3																
CDK6	0.003642	0	0	RB1	CEBPA	RBL2															
CSNK1E	0.003653	0	0	CCND1	MYC	TUBB	CDKN2A														
PKD1	0.003653	0	0	MYB	HDAC7	MAPK8	JUN														
CK2A2	0.00386	0	0	MYC	HDAC2	RELA	JUN														
DNA-PK	0.00404	0	0	JUN	PRKDC																
CDK3	0.004333	0	0	RB1	JUN	CDK2															

AURKB	0.004647	0	0	RB1	YY1	PRKDC	HIST1H3A	PIN1	NELFE
LYN	0.005085	0	0	SPI1	YY1	CCND1	MYC	CSNK2A1	PRKCD
RET	0.00686	0	0	MAPK8	MAPK1	MAPK3			
CDK7	0.007315	0	0	GTF2F1	CDK9	CDK2	TAF3		
PAK2	0.007644	0	0	MYC	SMAD2	JUN	HIST1H4A		
PLK3	0.007861	0	0	ATF2	HIF1A	JUN			
BRD2	0.007965	0	0	RB1	RBL2				
ADRBK1	0.007965	0	0	PRKCD	MAPK3				
TBK1	0.008688	0	0	RUVBL2	TUBB	RPL11	RELA		
IKBKE	0.009056	0	0	SKP1	TUBB	CUL1	RELA		
ACTR2B	0.010372	0	0	SMAD2	SMAD3				
PKCIOTA	0.010722	0	0	RELA	CCND1	RUVBL2			
MAPK12	0.011048	0	0	ATF2	CCND1	FOS	JUN		
EGFR	0.012125	0	0	CCND1	PRKCD	PRKDC	HIST1H3A	JUN	FOS
CHEK2	0.014115	0	0	RB1	VHL	PML			
GSK3ALPHA	0.015623	0	0	CCND1	MYC	JUN			
JAK2	0.01641	0	0	HIST1H3A	MAPK1	MAPK3			
CSNK2B	0.019225	0	0	FOS	RUVBL2				
CDKN1B	0.019467	0	0	CDK2					
BAZ1B	0.019467	0	0	H2AFX					
TRIM33	0.019467	0	0	SMAD4					
HASPIN	0.019467	0	0	HIST1H3A					
PCNA	0.019467	0	0	CDK2					
TGM2	0.019467	0	0	HIST1H3A					
EP400	0.019467	0	0	TRRAP					
LYK5	0.019467	0	0	SMAD4					
HIPK1	0.022684	0	0	DAXX	MYB				
RPS6KA4	0.026378	0	0	RELA	HIST1H3A				
MAP3K8	0.028651	0	0	PIM1	MAPK1	MAPK3			
GTF2F1	0.02906	0	0	GTF2F1					
PRKCQ	0.029748	0	0	SPI1	SIRT2	HIST1H3A			
MOK	0.030297	0	0	MYC	JUN				

PAK1	0.03126	0	0	CCND1	PRKCD	CTBP1	HIST1H3A	
CDK8	0.034433	0	0	HIST1H3A	SMAD3			
UHMK1	0.036578	0	0	SMAD2	SMAD4			
MAPK7	0.036578	0	0	FOS	PML			
MAP3K20	0.03856	0	0	HIST1H3A				
EEF2K	0.038774	0	0	MAPK8	MAPK11			
MAPK13	0.039307	0	0	ATF2	FOS	JUN		
MAP3K14	0.040003	0	0	HNRNPU	RUVBL2	TUBB	RPL11	
BARK1	0.043249	0	0	PRKCD	MAPK3	PIN1		
DAPK1	0.043311	0	0	MAPK3	PIN1			
FYN	0.044112	0	0	SPI1	PRKCD	FOS	SIRT2	CCND1
INSR	0.044605	0	0	CCND1	FOS	JUN		
NPM/ALK	0.047969	0	0	MSH2				
AURORAA	0.050241	0	0	CCND1	NCOR1	HDAC3		
MAP3K5	0.050467	0	0	MAPK8	DAXX			
AURKA	0.051702	0	0	HIST1H3A	VHL	PIN1		
PRKCB	0.05614	0	0	ATF2	HIST1H3A	JUN	CCND1	BCL2
CK1ALPHA	0.056208	0	0	RB1	FOS	JUN		
DYRK2	0.060613	0	0	MYC	JUN			
MAP3K1	0.062473	0	0	RUVBL2	HIST1H4A	TUBB	DDB1	
PDGFRBETA	0.06325	0	0	MAX	PRKCD			
MAP2K2	0.065926	0	0	MAPK1	MAPK3			
DYRK1A	0.065926	0	0	CCND1	SIRT1			
RPS6KA5	0.065926	0	0	RELA	HIST1H3A			
NIK	0.066513	0	0	CEBPB				
HSPB8	0.066513	0	0	MAPK3				
RIPK1	0.06864	0	0	TUBB	PML			
PKC-B	0.075652	0	0	ATF2				
RSK-1	0.075652	0	0	CEBPB				
NLK	0.076995	0	0	MYB	SMAD4			
IKKBETA	0.079849	0	0	RELA	FOS			
LCK	0.084656	0	0	SPI1	PRKCD	MAPK1	MAPK3	

PRKCI	0.085654	0	0	DDB1	SP1			
PRKAA1	0.090696	0	0	RB1	CTBP1	SIRT2		
RSK-2	0.093664	0	0	FOS				
MAP2K4	0.094592	0	0	DAXX	MAPK8			
RIPK3	0.100434	0	0	RUVBL2	DAXX	TUBB		
MTOR	0.102431	0	0	MYC	JUN	TAF3		
PKC-Z	0.10254	0	0	RELA				
CAMKIV	0.10254	0	0	RELA				
CAMKIIBETA	0.103786	0	0	FOS	PPARGC1A			
JNK3	0.11133	0	0	JUN				
MAP3K3	0.115025	0	0	RUVBL2	HIST1H4A	TUBB	DDB1	
CDK5	0.116657	0	0	RB1	MYC	HIF1A	BCL2	
TRKB	0.120034	0	0	FOS				
AKT2	0.122854	0	0	HIST1H3A	PPARGC1A			
CAMK2A	0.129773	0	0	CCND1	MYC	SMAD2		
PLK1	0.142998	0	0	HNRNPU	MYC	CCNT1	YY1	PIN1
PKCALPHA	0.143187	0	0	MYC	PRKCD	BCL2		
SRM	0.145647	0	0	RUVBL2				
BTK	0.146068	0	0	FOS	JUN			
RPS6KB1	0.149457	0	0	PPARGC1A	CDKN2A			
TAF1	0.15402	0	0	TAF7				
PRKD1	0.166626	0	0	HDAC7	JUN			
SRPK3	0.170524	0	0	SIRT2				
PTK7	0.170524	0	0	DDB1				
PDK-1	0.178656	0	0	PRKCD				
TIE1	0.178656	0	0	MAPK11				
PKCETA	0.178656	0	0	PRKCD				
MINK1	0.178656	0	0	SMAD2				
CDK20	0.186709	0	0	CDK2				
CDK18	0.186709	0	0	SIRT2				
DMPK	0.186709	0	0	SP1				
PKN3	0.186709	0	0	GATA2				

PKA-A	0.186709	0	0	RELA	
NTRK3	0.186709	0	0	FOS	
MARK4	0.186709	0	0	MAPK11	
PRKCE	0.191196	0	0	ATF2	MAPK3
PIM3	0.194684	0	0	PML	
MAP3K4	0.194684	0	0	TRRAP	
TLK2	0.194684	0	0	ASF1A	
GSG2	0.194684	0	0	HIST1H3A	
MAPK15	0.194684	0	0	JUN	
CSNK1A1	0.194747	0	0	RELA	VHL
MAP3K6	0.202583	0	0	MAPK8	
MAP3K10	0.202583	0	0	TCF3	
TLK1	0.202583	0	0	HIST1H3A	
MAPK6	0.202583	0	0	DDB1	
WEE1	0.202583	0	0	CDK2	
FRK	0.210404	0	0	FOS	
IKKALPHA	0.210404	0	0	RELA	
CSNK1G1	0.210404	0	0	RELA	
MAP3K11	0.210404	0	0	PIN1	
DYRK3	0.210404	0	0	SIRT1	
AURKC	0.210404	0	0	HIST1H3A	
PAK5	0.21815	0	0	TCF3	
MYLK	0.225821	0	0	MAPK3	
NEK1	0.225821	0	0	VHL	
PIM2	0.225821	0	0	MYC	
ICK	0.225821	0	0	SIRT2	
MARK3	0.225821	0	0	DDB1	
RPS6KA2	0.233418	0	0	MAPK3	
MKNK1	0.233418	0	0	MAPK3	
AKT3	0.248391	0	0	PRKCD	
PKBGAMMA	0.248391	0	0	RELA	

TNK2	0.248391	0	0	HIST1H4A	
DYRK1B	0.255769	0	0	CCND1	
BMX	0.255769	0	0	PIM1	
LKB1	0.255769	0	0	PPARGC1A	
CAMK4	0.255769	0	0	RELA	
STK3	0.255769	0	0	HIST1H3A	
GRK2	0.255769	0	0	SMAD2	
PRKACB	0.263075	0	0	FOS	
JAK1	0.263075	0	0	HIST1H3A	
CAMK2D	0.263075	0	0	SMAD4	
PKN1	0.270311	0	0	HIST1H3A	
NEK6	0.270311	0	0	RBL2	
CAMK1	0.270311	0	0	HDAC7	
TTK	0.274252	0	0	CDK2	E2F4
MAPKAPK5	0.277476	0	0	MAPK11	
YES1	0.277476	0	0	PRKCD	
STK4	0.284572	0	0	H2AFX	
BMPR1B	0.284572	0	0	SMAD2	
ZAP70	0.284572	0	0	MAPK11	
CSK	0.291599	0	0	PML	
PDGFRB	0.291599	0	0	PRKCD	
ERBB2	0.299692	0	0	CCND1	NCOR1
PKG1CGKI	0.305449	0	0	SP1	
PKAALPHA	0.31056	0	0	FOS	RELA
ALK	0.312274	0	0	RELA	
ARAF	0.319032	0	0	PRKCD	
MARK2	0.319032	0	0	HDAC7	
STK11	0.332353	0	0	SMAD4	
MAP2K7	0.332353	0	0	MAPK8	
PRKAA2	0.332353	0	0	PPARGC1A	
HCK	0.332353	0	0	SPI1	

ABL2	0.338916	0	0	CEBPB
RIPK2	0.358227	0	0	TUBB
MAP2K6	0.358227	0	0	MAPK11
CSNK1D	0.364539	0	0	HIF1A
PAK3	0.37079	0	0	CCND1
PKBBETA	0.37698	0	0	RELA
IGF1R	0.41866	0	0	FOS
ABL	0.435669	0	0	CDK2
PKAGAMMA	0.441228	0	0	RELA
CAMKIIALPHA	0.452186	0	0	CEBPB
TGFBR1	0.452186	0	0	SMAD2
SGK1	0.478659	0	0	MAPK1
PDHK1	0.503869	0	0	PRKCD
ROCK1	0.513613	0	0	HIST1H3A
PKCGAMMA	0.518414	0	0	PRKCD
AURORAB	0.537153	0	0	RB1

Supplementary Table 6

CMap co-upregulated genes

Term	Overlap	P-value	Adjusted P-value	Old P-value	Old Adjusted P-value	Z-score	Combined Score	Genes
camptothecin-3887	8/100	1.69E-06	0.000752	1.15E-05	0.005105	-1.70405	22.64764	ID1;ENC1;CD14;SOX9;FOS;S100A11;ETS2;CTGF
neostigmine bromide-5335	8/100	1.69E-06	0.000752	1.15E-05	0.005105	-1.70333	22.63807	LGALS3BP;IFITM1;SERPINA1;IFI27;CEACAM6;IFI6;ISG15;TSPAN1
emetine-2801	8/100	1.69E-06	0.000752	1.15E-05	0.005105	-1.68926	22.45107	IL32;ID1;TXNIP;ID3;SOX9;FOS;IER2;CTGF
remoxipride-5443	8/100	1.69E-06	0.000752	1.15E-05	0.005105	-1.68865	22.44292	C3;IFITM1;IFI27;PTPRO;IFI6;SECTM1;ISG15;ITM2C
cephaeline-5247	8/100	1.69E-06	0.000752	1.15E-05	0.005105	-1.685	22.39448	C3;ID1;TXNIP;ID3;SOX9;FOS;IER2;CTGF
cephaeline-5612	8/100	1.69E-06	0.000752	1.15E-05	0.005105	-1.67616	22.27703	IL32;ID1;TXNIP;ID3;FOS;IER2;CTGF;PSMB9
emetine-4827	8/100	1.69E-06	0.000752	1.15E-05	0.005105	-1.67254	22.22886	C3;ID1;TXNIP;ID3;FOS;PHLDA1;IER2;PSMB9
thioridazine-1068	8/100	1.69E-06	0.000752	1.15E-05	0.005105	-1.66472	22.12498	JUND;S100P;SOX9;FOS;TGFBI;PHLDA1;IER2;ETS2
thioridazine-4164	8/100	1.69E-06	0.000752	1.15E-05	0.005105	-1.65358	21.97687	IFI27;CEACAM5;IFI6;ISG15;SOX9;FOS;LYZ;PHLDA1
lynestrenol-5355	7/100	1.85E-05	0.002963	9.17E-05	0.01468	-1.71008	18.63452	IFITM1;SERPINA1;IFI27;IFI6;TFF2;SOX9;PHLDA1
cisapride-5305	7/100	1.85E-05	0.002963	9.17E-05	0.01468	-1.69033	18.41939	COL1A1;RCN1;IFITM1;IFI27;IFI6;S100A4;S100A11

propofol-5306	7/100	1.85E-05	0.002963	9.17E-05	0.01468	-1.68689	18.3819	LGALS3BP;IFITM1;IFI27;IFI6;AP1S1;S100A4;ISG15
cephaeline-3290	7/100	1.85E-05	0.002963	9.17E-05	0.01468	-1.685	18.36122	IL32;ID1;TXNIP;ID3;IER2;CTGF;PSMB9
puromycin-5310	7/100	1.85E-05	0.002963	9.17E-05	0.01468	-1.68148	18.32294	IFNGR1;ID1;ENC1;TXNIP;ID3;PHLDA1;CTGF
ursolic acid-7181	7/100	1.85E-05	0.002963	9.17E-05	0.01468	-1.67246	18.22459	IFITM1;IFI27;CEACAM5;MMP3;IFI6;ISG15;ITM2B
anisomycin-5364	7/100	1.85E-05	0.002963	9.17E-05	0.01468	-1.66496	18.1429	ID1;TXNIP;ID3;FOS;PHLDA1;IER2;CTGF
felodipine-848	7/100	1.85E-05	0.002963	9.17E-05	0.01468	-1.65327	18.01551	IFITM1;TXNIP;TFF2;FOS;PHLDA1;IER2;ETS2
tretinoin-1049	7/100	1.85E-05	0.002963	9.17E-05	0.01468	-1.65282	18.01065	FXYD3;CEACAM6;ID1;S100P;SOX9;TGFBI;PHLDA1
8-azaguanine-1791	7/100	1.85E-05	0.002963	9.17E-05	0.01468	-1.64866	17.96522	CCL20;CXCL1;FOS;PHLDA1;IER2;ETS2;CTGF
azacyclonol-1937	7/100	1.85E-05	0.002963	9.17E-05	0.01468	-1.64521	17.9277	JUND;CCL20;ISG15;CXCL1;FOS;IER2;CTGF
sirolimus-1646	7/100	1.85E-05	0.002963	9.17E-05	0.01468	-1.63855	17.85515	IFITM1;SERPINA1;IFI27;ID1;IFI6;TXNIP;ISG15
digoxigenin-5640	7/100	1.85E-05	0.002963	9.17E-05	0.01468	-1.63661	17.83395	ID1;TXNIP;ID3;SOX9;FOS;IER2;CTGF
mitoxantrone-5354	7/100	1.85E-05	0.002963	9.17E-05	0.01468	-1.63573	17.82437	LGALS3BP;IFITM1;IFI27;CEACAM6;S100A6;IFI6;ISG15
digoxigenin-5275	7/100	1.85E-05	0.002963	9.17E-05	0.01468	-1.63346	17.79963	JUND;TXNIP;ID3;FOS;PHLDA1;IER2;CTGF
clonidine-6814	7/100	1.85E-05	0.002963	9.17E-05	0.01468	-1.62494	17.70683	LGALS3BP;IFITM1;IFI27;IFI6;ISG15;SOX9;ITM2C
astemizole-6807	6/100	0.000175	0.010945	0.000644	0.040262	-1.69977	14.70349	TXNIP;SOX9;FOS;PHLDA1;IER2;ETS2
benzethonium chloride-7207	6/100	0.000175	0.010945	0.000644	0.040262	-1.68154	14.54578	COL1A1;SERPINA1;SPINK1;CEACAM6;CEACAM5;PHLDA1
hexetidine-3318	6/100	0.000175	0.010945	0.000644	0.040262	-1.68029	14.53501	JUND;SOX9;FOS;PHLDA1;IER2;ETS2
glycocholic acid-5316	6/100	0.000175	0.010945	0.000644	0.040262	-1.67831	14.51789	BMP4;IFITM1;IFI6;S100A4;ISG15;TSPAN1
ergocalciferol-5346	6/100	0.000175	0.010945	0.000644	0.040262	-1.67323	14.47389	BMP4;IFITM1;SERPINA1;IFI27;IFI6;ISG15
digitoxigenin-3275	6/100	0.000175	0.010945	0.000644	0.040262	-1.67156	14.45944	COL1A1;ID1;TXNIP;ID3;IER2;CTGF
mefloquine-2210	6/100	0.000175	0.010945	0.000644	0.040262	-1.66785	14.42736	IL32;TXNIP;SOX9;FOS;IER2;ETS2
camptothecin-2321	6/100	0.000175	0.010945	0.000644	0.040262	-1.66035	14.36247	ID1;SOX9;FOS;IER2;ETS2;CTGF
chlorcyclizine-4367	6/100	0.000175	0.010945	0.000644	0.040262	-1.65826	14.34445	IFITM1;JUND;IFI27;IFI6;ISG15;FOS
helveticoside-3770	6/100	0.000175	0.010945	0.000644	0.040262	-1.65609	14.32568	JUND;TXNIP;ID3;FOS;ETS2;CTGF
CP-320650-01-4379	6/100	0.000175	0.010945	0.000644	0.040262	-1.65255	14.295	COL1A1;IFITM1;SERPINA1;IFI27;IFI6;ISG15
sirolimus-1667	6/100	0.000175	0.010945	0.000644	0.040262	-1.65197	14.28998	COL1A1;IFITM1;ID1;IFI6;TXNIP;ISG15
ceforanide-5351	6/100	0.000175	0.010945	0.000644	0.040262	-1.65082	14.28007	IFITM1;IFI27;CEACAM6;IFI6;S100A4;ISG15
netilmicin-6005	6/100	0.000175	0.010945	0.000644	0.040262	-1.64906	14.26487	COL1A1;IFITM1;IFI27;CEACAM6;IFI6;ISG15
bemegride-5014	6/100	0.000175	0.010945	0.000644	0.040262	-1.64754	14.25164	LGALS3BP;BMP4;IFITM1;IFI27;IFI6;ISG15
fulvestrant-1043	6/100	0.000175	0.010945	0.000644	0.040262	-1.64422	14.22294	JUND;ID1;ENC1;TXNIP;ID3;ITM2B
lanatoside C-6048	6/100	0.000175	0.010945	0.000644	0.040262	-1.63987	14.18531	ID1;TXNIP;ID3;FOS;IER2;CTGF
cicloheximide-6220	6/100	0.000175	0.010945	0.000644	0.040262	-1.63626	14.15413	ID1;ENC1;TXNIP;ID3;PHLDA1;IER2
(+)-chelidonine-1786	6/100	0.000175	0.010945	0.000644	0.040262	-1.63566	14.14892	SLC12A2;CCL20;ACSL1;IFNGR1;FOS;ETS2
helveticoside-6047	6/100	0.000175	0.010945	0.000644	0.040262	-1.63559	14.14832	ID1;TXNIP;ID3;FOS;IER2;CTGF
azacitidine-4128	6/100	0.000175	0.010945	0.000644	0.040262	-1.63411	14.13552	COL1A1;SPINK1;IFI6;TXNIP;PHLDA1;CTGF
tacrine-5297	6/100	0.000175	0.010945	0.000644	0.040262	-1.63239	14.12064	LGALS3BP;IFITM1;IFI27;IFI6;S100A4;ISG15
perhexiline-5501	6/100	0.000175	0.010945	0.000644	0.040262	-1.62902	14.09147	PTPRO;IFI6;SOX9;FOS;PHLDA1;IER2

tretinoin-849	6/100	0.000175	0.010945	0.000644	0.040262	-1.62511	14.05762	FXYD3;ID1;SOX9;TGFBI;ATP1B1;PHLDA1
helveticoside-3945	6/100	0.000175	0.010945	0.000644	0.040262	-1.61913	14.00592	ID1;TXNIP;ID3;FOS;IER2;CTGF
triprolidine-7248	6/100	0.000175	0.010945	0.000644	0.040262	-1.61815	13.99745	IFITM1;IFI6;ISG15;CXCL1;S100A11;CTGF
helveticoside-2192	6/100	0.000175	0.010945	0.000644	0.040262	-1.61748	13.99167	PLCB4;CCL20;TXNIP;ID3;FOS;PHLDA1
strophanthidin-6087	6/100	0.000175	0.010945	0.000644	0.040262	-1.61426	13.96376	ID1;TXNIP;ID3;FOS;IER2;CTGF
H-7-5936	6/100	0.000175	0.010945	0.000644	0.040262	-1.6139	13.96069	IFITM1;SPINK1;PLP2;GAPDH;ACTB;DCN
sanguinarine-2927	6/100	0.000175	0.010945	0.000644	0.040262	-1.61207	13.94483	TSPAN13;MMP1;ID1;HSPB1;ISG15;ATP1B1
cyclopentolate-6214	6/100	0.000175	0.010945	0.000644	0.040262	-1.60809	13.91044	IFITM1;IFI27;IFI6;TFF2;ISG15;DCN
trichostatin A-4072	6/100	0.000175	0.010945	0.000644	0.040262	-1.60439	13.87838	BMP4;CCL20;MMP1;FOS;CTGF;PSMB9
podophyllotoxin-2540	6/100	0.000175	0.010945	0.000644	0.040262	-1.60208	13.85844	ANXA2;CCL20;PTPRO;ID3;TIMP1;PHLDA1
strophanthidin-5826	6/100	0.000175	0.010945	0.000644	0.040262	-1.59875	13.82961	JUND;TXNIP;ID3;FOS;ETS2;CTGF
metoprolol-6846	6/100	0.000175	0.010945	0.000644	0.040262	-1.59864	13.82865	PIGR;IFITM1;IFI27;IFI6;TFF2;ISG15
helveticoside-3851	6/100	0.000175	0.010945	0.000644	0.040262	-1.59396	13.7882	ID1;TXNIP;ID3;FOS;IER2;CTGF
lysergol-6621	6/100	0.000175	0.010945	0.000644	0.040262	-1.5817	13.68215	IFITM1;JUND;TFF3;IFI6;TGFBI;PSMB9
kawain-2299	6/100	0.000175	0.010945	0.000644	0.040262	-1.58074	13.67387	COL1A2;SPINK1;SLPI;IFI27;CEACAM5;AP1S1
terfenadine-1381	6/100	0.000175	0.010945	0.000644	0.040262	-1.57138	13.5929	ANXA2;CCL20;SPP1;IFI6;ISG15;PHLDA1
astemizole-2211	5/100	0.001403	0.036926	0.003903	0.102731	-1.67192	10.9833	TXNIP;SOX9;FOS;PHLDA1;IER2
bisacodyl-5677	5/100	0.001403	0.036926	0.003903	0.102731	-1.63936	10.76938	COL1A1;SOX9;FOS;OLFM4;PHLDA1
anisomycin-2658	5/100	0.001403	0.036926	0.003903	0.102731	-1.62711	10.68895	ID1;TXNIP;ID3;SOX9;IER2
3-hydroxy-DL-kynurenine-5276	5/100	0.001403	0.036926	0.003903	0.102731	-1.62039	10.64476	COL1A1;IFITM1;IFI27;IFI6;ISG15
azacyclonol-1520	5/100	0.001403	0.036926	0.003903	0.102731	-1.62004	10.6425	TXNIP;FOS;PHLDA1;IER2;ETS2
fendiline-3190	5/100	0.001403	0.036926	0.003903	0.102731	-1.61845	10.63202	JUND;SPINK1;SOX9;FOS;IER2
bisoprolol-5348	5/100	0.001403	0.036926	0.003903	0.102731	-1.61682	10.62132	IFITM1;SERPINA1;CEACAM6;IFI6;S100A4
8-azaguanine-1833	5/100	0.001403	0.036926	0.003903	0.102731	-1.61503	10.60956	CCL20;TXNIP;ID3;TIMP1;PHLDA1
CP-690334-01-3823	5/100	0.001403	0.036926	0.003903	0.102731	-1.61349	10.59943	IL32;JUND;MMP1;OLFM4;CTGF
brinzolamide-5016	5/100	0.001403	0.036926	0.003903	0.102731	-1.60977	10.57499	BMP4;IFITM1;IFI27;AP1S1;ISG15
megestrol-5013	5/100	0.001403	0.036926	0.003903	0.102731	-1.60947	10.57303	IFITM1;IFI27;MMP3;IFI6;ISG15
cephaeline-2429	5/100	0.001403	0.036926	0.003903	0.102731	-1.60766	10.56116	CCL20;TXNIP;ID3;FOS;ETS2
niflumic acid-5490	5/100	0.001403	0.036926	0.003903	0.102731	-1.60764	10.56101	IFITM1;IFI27;IFI6;ISG15;DCN
alprostadil-6571	5/100	0.001403	0.036926	0.003903	0.102731	-1.60679	10.55545	HSP90AB1;SYPL1;AP1S1;GAPDH;ACTB
fluspirilene-6463	5/100	0.001403	0.036926	0.003903	0.102731	-1.60496	10.54344	SOX9;FOS;PHLDA1;IER2;CTGF
clorgiline-5005	5/100	0.001403	0.036926	0.003903	0.102731	-1.60248	10.52712	BMP4;IFITM1;IFI6;ISG15;DCN
fendiline-7188	5/100	0.001403	0.036926	0.003903	0.102731	-1.60117	10.51849	IFI6;FOS;PHLDA1;IER2;ETS2
genistein-1176	5/100	0.001403	0.036926	0.003903	0.102731	-1.60042	10.5136	APP;CCL20;ID3;FOS;PHLDA1
alvespimycin-6933	5/100	0.001403	0.036926	0.003903	0.102731	-1.59792	10.49714	IFITM1;IFI27;IFI6;ISG15;SOX9
ethisterone-3783	5/100	0.001403	0.036926	0.003903	0.102731	-1.59692	10.4906	COL1A1;IFITM1;IFI27;IFI6;DUOX2
cicloheximide-5743	5/100	0.001403	0.036926	0.003903	0.102731	-1.59584	10.48352	TXNIP;ID3;PHLDA1;ETS2;CTGF

dinoprostone-6586	5/100	0.001403	0.036926	0.003903	0.102731	-1.59504	10.47824	IGFBP2;ID1;AP1S1;S100P;ETS2
carbinoxamine-6786	5/100	0.001403	0.036926	0.003903	0.102731	-1.59382	10.47025	IFITM1;IFI27;IFI6;ISG15;SOX9
cicloheximide-3464	5/100	0.001403	0.036926	0.003903	0.102731	-1.5925	10.46158	ID1;ENC1;TXNIP;ID3;SOX9
hesperidin-5313	5/100	0.001403	0.036926	0.003903	0.102731	-1.59074	10.45002	BMP4;IFITM1;IFI27;IFI6;ISG15
suloctidil-2651	5/100	0.001403	0.036926	0.003903	0.102731	-1.58922	10.44003	SOX9;FOS;PHLDA1;IER2;ETS2
clomipramine-1566	5/100	0.001403	0.036926	0.003903	0.102731	-1.58895	10.43821	RPS14P3;PIGR;CCL20;CTSD;LY6E
prasterone-5019	5/100	0.001403	0.036926	0.003903	0.102731	-1.58859	10.43588	IFITM1;IFI6;ISG15;SOX9;CTSE
BW-B70C-1132	5/100	0.001403	0.036926	0.003903	0.102731	-1.58806	10.43239	ISG15;SOX9;FOS;PHLDA1;ETS2
dexibuprofen-3094	5/100	0.001403	0.036926	0.003903	0.102731	-1.58624	10.42042	PIGR;IFITM1;GPX2;SPARC;AP1S1
terfenadine-6823	5/100	0.001403	0.036926	0.003903	0.102731	-1.58414	10.40662	TXNIP;SOX9;FOS;PHLDA1;IER2
disulfiram-2053	5/100	0.001403	0.036926	0.003903	0.102731	-1.58404	10.40598	APP;IFI6;HSPB1;FOS;CTGF
thioridazine-1010	5/100	0.001403	0.036926	0.003903	0.102731	-1.58387	10.40487	JUND;SOX9;FOS;PHLDA1;ETS2
cimetidine-4144	5/100	0.001403	0.036926	0.003903	0.102731	-1.58243	10.3954	IFITM1;IFI27;CEACAM5;IFI6;ISG15
skimmianine-6242	5/100	0.001403	0.036926	0.003903	0.102731	-1.57809	10.36689	BMP4;IFITM1;IFI27;IFI6;ISG15
menadione-2439	5/100	0.001403	0.036926	0.003903	0.102731	-1.57625	10.35479	NQO1;ANXA2;HSPB1;ATP1B1;PHLDA1
glipizide-4991	5/100	0.001403	0.036926	0.003903	0.102731	-1.5748	10.34529	IFITM1;IFI27;CEACAM5;IFI6;SECTM1
clonidine-1555	5/100	0.001403	0.036926	0.003903	0.102731	-1.57374	10.33833	ANXA2;CCL20;QPCT;TIMP1;PHLDA1
co-dergocrine mesilate-2136	5/100	0.001403	0.036926	0.003903	0.102731	-1.57316	10.33453	ANXA2;CCL20;ID3;ITGAV;TIMP1
nimesulide-2275	5/100	0.001403	0.036926	0.003903	0.102731	-1.57135	10.32264	IFITM1;SPARC;IFI27;IFI6;ISG15
lycorine-4365	5/100	0.001403	0.036926	0.003903	0.102731	-1.57116	10.32138	IFNGR1;ENC1;TXNIP;SOX9;PHLDA1
felodipine-965	5/100	0.001403	0.036926	0.003903	0.102731	-1.57084	10.31925	TXNIP;FOS;PHLDA1;IER2;ETS2
LY-294002-6995	5/100	0.001403	0.036926	0.003903	0.102731	-1.57019	10.31497	IFITM1;IFI27;IFI6;TXNIP;ISG15
mephentermine-4707	5/100	0.001403	0.036926	0.003903	0.102731	-1.56821	10.30201	LGALS3BP;IFITM1;IFI27;IFI6;ISG15
diloxanide-5025	5/100	0.001403	0.036926	0.003903	0.102731	-1.56808	10.30113	IFITM1;IFI27;IFI6;AP1S1;ISG15
terfenadine-2227	5/100	0.001403	0.036926	0.003903	0.102731	-1.5672	10.29536	JUND;SOX9;FOS;PHLDA1;IER2
etoposide-1626	5/100	0.001403	0.036926	0.003903	0.102731	-1.56569	10.28545	CCL20;IFI6;ISG15;CD14;PHLDA1
mebendazole-4694	5/100	0.001403	0.036926	0.003903	0.102731	-1.56565	10.28516	JUND;SOX9;TGFBI;PHLDA1;CTGF
myricetin-1334	5/100	0.001403	0.036926	0.003903	0.102731	-1.56467	10.27876	NQO1;JUND;SPP1;IFI6;PHLDA1
digitoxigenin-4801	5/100	0.001403	0.036926	0.003903	0.102731	-1.56338	10.27025	TXNIP;ID3;FOS;IER2;CTGF
mefloquine-6205	5/100	0.001403	0.036926	0.003903	0.102731	-1.56239	10.26375	TXNIP;SOX9;FOS;PHLDA1;IER2
thioridazine-5227	5/100	0.001403	0.036926	0.003903	0.102731	-1.56211	10.2619	S100P;SOX9;FOS;PHLDA1;ETS2
levonorgestrel-3406	5/100	0.001403	0.036926	0.003903	0.102731	-1.5615	10.25794	TFF2;SOX9;PHLDA1;DCN;ETS2
mifepristone-7183	5/100	0.001403	0.036926	0.003903	0.102731	-1.55947	10.24457	IFITM1;CEACAM5;IFI6;TXNIP;ISG15
colforsin-913	5/100	0.001403	0.036926	0.003903	0.102731	-1.55722	10.22978	TMPRSS3;FOS;PHLDA1;ETS2;CTGF
N6-methyladenosine-2626	5/100	0.001403	0.036926	0.003903	0.102731	-1.55708	10.2289	COL1A1;SERPINA1;SLPI;CEACAM5;TGFBI
mefenamic acid-5534	5/100	0.001403	0.036926	0.003903	0.102731	-1.55482	10.21402	IFITM1;CEACAM6;IFI6;ISG15;DCN
lovastatin-4978	5/100	0.001403	0.036926	0.003903	0.102731	-1.55387	10.20779	BMP4;SPINK1;AP1S1;TFF2;FOS

fulvestrant-1076	5/100	0.001403	0.036926	0.003903	0.102731	-1.55323	10.20359	CEACAM6;ID1;ENC1;ID3;IER2
pirlindole-5497	5/100	0.001403	0.036926	0.003903	0.102731	-1.55275	10.20044	COL1A1;CEACAM5;TMPRSS3;PTPRO;DUOX2
proscillaridin-7340	5/100	0.001403	0.036926	0.003903	0.102731	-1.54817	10.17037	ID1;TXNIP;ID3;FOS;IER2
mefexamide-2121	5/100	0.001403	0.036926	0.003903	0.102731	-1.54563	10.15366	APP;JUND;IFI6;TSPAN1;S100A11
thioridazine-1486	5/100	0.001403	0.036926	0.003903	0.102731	-1.54489	10.14878	AP1S1;S100P;SOX9;FOS;PHLDA1
prochlorperazine-2675	5/100	0.001403	0.036926	0.003903	0.102731	-1.54447	10.14605	ANXA2;CCL20;SPP1;TFF2;PHLDA1
ouabain-2656	5/100	0.001403	0.036926	0.003903	0.102731	-1.54438	10.14545	COL1A1;ID1;TXNIP;ID3;IER2
amodiaquine-1570	5/100	0.001403	0.036926	0.003903	0.102731	-1.5439	10.14232	RPS14P3;RCN1;CCL20;IFI6;PTMA
5155877-6574	5/100	0.001403	0.036926	0.003903	0.102731	-1.54347	10.13947	APP;HSP90AB1;ID1;GAPDH;ACTB
trichostatin A-5987	5/100	0.001403	0.036926	0.003903	0.102731	-1.54267	10.13423	IFI27;IFI6;TXNIP;SOX9;ITM2C
Gly-His-Lys-6570	5/100	0.001403	0.036926	0.003903	0.102731	-1.54099	10.12321	HSP90AB1;SYPL1;AP1S1;GAPDH;ACTB
metformin-1816	5/100	0.001403	0.036926	0.003903	0.102731	-1.53852	10.10695	IFITM1;IFI27;CCL20;IFI6;AP1S1
piperlongumine-2757	5/100	0.001403	0.036926	0.003903	0.102731	-1.53705	10.09728	ID1;TFF2;FOS;ETS2;CTGF
ouabain-5026	5/100	0.001403	0.036926	0.003903	0.102731	-1.5343	10.07922	TXNIP;ID3;FOS;IER2;CTGF
trichostatin A-2208	5/100	0.001403	0.036926	0.003903	0.102731	-1.53297	10.07051	TSPAN13;TCN1;IGFBP2;ID3;CKB
zuclopenthixol-7356	5/100	0.001403	0.036926	0.003903	0.102731	-1.5292	10.04575	CEACAM5;IFI6;SOX9;PHLDA1;S100A11
prochlorperazine-1053	5/100	0.001403	0.036926	0.003903	0.102731	-1.52465	10.01581	S100P;SOX9;TGFBI;PHLDA1;ETS2
puromycin-3310	5/100	0.001403	0.036926	0.003903	0.102731	-1.51889	9.977998	IFNGR1;ID1;TXNIP;ID3;CTGF
practolol-1587	5/100	0.001403	0.036926	0.003903	0.102731	-1.5184	9.974771	CCL20;CEACAM5;SPP1;IFI6;CKB
tolbutamide-3886	5/100	0.001403	0.036926	0.003903	0.102731	-1.51832	9.974244	COL1A1;IFITM1;SLPI;IFI27;IFI6
midecamycin-5345	5/100	0.001403	0.036926	0.003903	0.102731	-1.51826	9.973851	IFITM1;IFI27;IFI6;ISG15;ETS2
thioridazine-2690	5/100	0.001403	0.036926	0.003903	0.102731	-1.51269	9.937247	ANXA2;CCL20;CEACAM5;SPP1;PHLDA1
trichostatin A-1835	5/100	0.001403	0.036926	0.003903	0.102731	-1.50471	9.884847	APP;TSPAN13;TCN1;ID3;ATP1B1
primaquine-1343	5/100	0.001403	0.036926	0.003903	0.102731	-1.50467	9.884599	NQO1;APP;CCL20;PHLDA1;ETS2
tretinoin-6243	5/100	0.001403	0.036926	0.003903	0.102731	-1.50383	9.87907	CEACAM6;ID1;SOX9;TGFBI;PHLDA1
puromycin-6711	5/100	0.001403	0.036926	0.003903	0.102731	-1.49786	9.839859	RNF43;TXNIP;ID3;CXCL1;TGFBI
thalidomide-7288	5/100	0.001403	0.036926	0.003903	0.102731	-1.49329	9.809794	C3;APP;SERPINA1;MMP3;DCN
tanespimycin-6185	5/100	0.001403	0.036926	0.003903	0.102731	-1.48945	9.784598	COL1A1;GPX2;COL1A2;HSPB1;TGFBI
nortriptyline-7422	5/100	0.001403	0.036926	0.003903	0.102731	-1.4867	9.766504	IFITM1;IFI6;ISG15;PHLDA1;ETS2
trichostatin A-3688	5/100	0.001403	0.036926	0.003903	0.102731	-1.47134	9.66563	BMP4;APP;MMP1;CTGF;PSMB9
acetohexamide-1829	4/100	0.009292	0.10653	0.019932	0.228502	-1.56036	7.300214	APP;IFITM1;IFI27;IFI6
altizide-6829	4/100	0.009292	0.10653	0.019932	0.228502	-1.55192	7.260744	IFITM1;IFI27;IFI6;ISG15
amantadine-4222	4/100	0.009292	0.10653	0.019932	0.228502	-1.53623	7.187334	IFITM1;LUM;CEACAM6;IFI6
15-delta prostaglandin J2-2691	4/100	0.009292	0.10653	0.019932	0.228502	-1.52866	7.151918	NQO1;TSPAN13;HSPB1;ATP1B1
bepridil-5674	4/100	0.009292	0.10653	0.019932	0.228502	-1.52636	7.141161	SOX9;FOS;PHLDA1;IER2
bumetanide-5542	4/100	0.009292	0.10653	0.019932	0.228502	-1.52478	7.133774	IFITM1;IFI6;ISG15;DCN
0175029-0000-4716	4/100	0.009292	0.10653	0.019932	0.228502	-1.52215	7.121439	IFI27;IFNGR1;IFI6;TXNIP

alvespimycin-1051	4/100	0.009292	0.10653	0.019932	0.228502	-1.51915	7.10743	APP;HSP90AB1;TXNIP;AP1S1
dequalinium chloride-5396	4/100	0.009292	0.10653	0.019932	0.228502	-1.51827	7.103301	COL1A1;APP;TXNIP;SOX9
canadine-4138	4/100	0.009292	0.10653	0.019932	0.228502	-1.51466	7.086425	SLPI;IFI27;IFI6;DCN
beta-escin-4544	4/100	0.009292	0.10653	0.019932	0.228502	-1.51131	7.070741	CCL20;CXCL1;FOS;ETS2
beta-escin-4364	4/100	0.009292	0.10653	0.019932	0.228502	-1.50935	7.061564	CEACAM5;AP1S1;SOX9;PHLDA1
1,4-chrysenequinone-7139	4/100	0.009292	0.10653	0.019932	0.228502	-1.50757	7.053243	MMP1;HSPB1;FOS;CTGF
5255229-949	4/100	0.009292	0.10653	0.019932	0.228502	-1.50745	7.052688	SOX9;FOS;PHLDA1;ETS2
4-hydroxyphenazone-4175	4/100	0.009292	0.10653	0.019932	0.228502	-1.50404	7.03672	PIGR;IFITM1;IFI27;IFI6
aminophenazone-1376	4/100	0.009292	0.10653	0.019932	0.228502	-1.50388	7.035977	SERPINA1;ENC1;CKB;DCN
disulfiram-2215	4/100	0.009292	0.10653	0.019932	0.228502	-1.50248	7.029439	ID1;ID3;FOS;CTGF
celastrol-887	4/100	0.009292	0.10653	0.019932	0.228502	-1.50166	7.025608	ID1;ID3;FOS;CTGF
(-)-isoprenaline-4495	4/100	0.009292	0.10653	0.019932	0.228502	-1.49967	7.0163	CEACAM6;TFF1;S100P;CD24
chlorpromazine-5493	4/100	0.009292	0.10653	0.019932	0.228502	-1.49893	7.012833	C3;TMPRSS3;FOS;PHLDA1
alfaxalone-5451	4/100	0.009292	0.10653	0.019932	0.228502	-1.49824	7.009596	COL1A1;PIGR;CEACAM5;IFI6
azacitidine-4010	4/100	0.009292	0.10653	0.019932	0.228502	-1.49719	7.004688	IL32;JUND;CEACAM6;TXNIP
bephenium hydroxynaphthoate-3089	4/100	0.009292	0.10653	0.019932	0.228502	-1.49567	6.997572	NQO1;IFITM1;CEACAM6;MMP1
cefotaxime-2072	4/100	0.009292	0.10653	0.019932	0.228502	-1.49373	6.988502	JUND;CCL20;IFI6;AP1S1
diprophylline-5482	4/100	0.009292	0.10653	0.019932	0.228502	-1.49312	6.985633	IFITM1;TMPRSS3;IFI6;ISG15
cetirizine-2468	4/100	0.009292	0.10653	0.019932	0.228502	-1.49298	6.985008	MMP1;TMPRSS3;TFF2;S100A11
diethylstilbestrol-3895	4/100	0.009292	0.10653	0.019932	0.228502	-1.4923	6.981823	SOX9;FOS;PHLDA1;CTGF
brompheniramine-4013	4/100	0.009292	0.10653	0.019932	0.228502	-1.49096	6.975557	PIGR;TSPAN8;CEACAM6;AP1S1
decamethonium bromide-4174	4/100	0.009292	0.10653	0.019932	0.228502	-1.48916	6.967112	IFITM1;IFI27;IFI6;ISG15
5253409-844	4/100	0.009292	0.10653	0.019932	0.228502	-1.48592	6.95195	ID1;ID3;FOS;ETS2
cefoxitin-6796	4/100	0.009292	0.10653	0.019932	0.228502	-1.48543	6.949652	IFITM1;IFI27;IFI6;ISG15
butamben-6266	4/100	0.009292	0.10653	0.019932	0.228502	-1.48515	6.948338	TMPRSS3;IFI6;ISG15;SOX9
dexamethasone-5797	4/100	0.009292	0.10653	0.019932	0.228502	-1.48505	6.947898	RNF43;IFITM1;SPP1;IFI6
16,16-dimethylprostaglandin E2-6562	4/100	0.009292	0.10653	0.019932	0.228502	-1.48311	6.938808	CEACAM5;TFF2;S100P;CD24
dienestrol-3448	4/100	0.009292	0.10653	0.019932	0.228502	-1.48215	6.93432	COL1A1;SERPINA1;FOS;PHLDA1
digoxigenin-3060	4/100	0.009292	0.10653	0.019932	0.228502	-1.48067	6.92738	COL1A1;TXNIP;ID3;FOS
cefuroxime-6261	4/100	0.009292	0.10653	0.019932	0.228502	-1.4801	6.924743	BMP4;APP;SLPI;ETS2
felodipine-3295	4/100	0.009292	0.10653	0.019932	0.228502	-1.47936	6.921256	SOX9;FOS;PHLDA1;IER2
betamethasone-1590	4/100	0.009292	0.10653	0.019932	0.228502	-1.47855	6.917491	CCL20;ACSL1;IFNGR1;PTMA
flufenamic acid-2104	4/100	0.009292	0.10653	0.019932	0.228502	-1.47759	6.912986	BMP4;JUND;IFI6;AP1S1
digoxin-2423	4/100	0.009292	0.10653	0.019932	0.228502	-1.47751	6.91262	TSPAN13;CCL20;TXNIP;FOS
dobutamine-1589	4/100	0.009292	0.10653	0.019932	0.228502	-1.47751	6.912604	IFITM1;ITGAV;ISG15;TIMP1
epiandrosterone-5687	4/100	0.009292	0.10653	0.019932	0.228502	-1.47661	6.908415	COL1A1;IFITM1;IFI27;ISG15

fenoterol-6331	4/100	0.009292	0.10653	0.019932	0.228502	-1.4743	6.897591	COL1A1;S100P;TGFBI;CD24
aminophylline-5395	4/100	0.009292	0.10653	0.019932	0.228502	-1.47193	6.886489	COL1A1;CEACAM5;MMP3;DCN
biperiden-5279	4/100	0.009292	0.10653	0.019932	0.228502	-1.47114	6.882826	C3;TCN1;CEACAM6;CEACAM5
betamethasone-5328	4/100	0.009292	0.10653	0.019932	0.228502	-1.47089	6.881625	BMP4;SERPINA1;CEACAM6;S100A4
digoxin-3283	4/100	0.009292	0.10653	0.019932	0.228502	-1.46867	6.871244	ID1;TXNIP;ID3;IER2
diethylstilbestrol-3429	4/100	0.009292	0.10653	0.019932	0.228502	-1.46797	6.867972	COL1A1;TMPRSS3;SOX9;PHLDA1
dextromethorphan-5401	4/100	0.009292	0.10653	0.019932	0.228502	-1.46378	6.84839	IFITM1;IFI27;IFI6;ISG15
iproniazid-2125	4/100	0.009292	0.10653	0.019932	0.228502	-1.46227	6.841328	BMP4;APP;IFI6;FOS
flutamide-3885	4/100	0.009292	0.10653	0.019932	0.228502	-1.46129	6.836724	IFITM1;TCN1;IFI6;AP1S1
emetine-2145	4/100	0.009292	0.10653	0.019932	0.228502	-1.45905	6.826241	CCL20;TXNIP;FOS;PHLDA1
lidoflazine-6278	4/100	0.009292	0.10653	0.019932	0.228502	-1.45868	6.824534	IFITM1;CEACAM6;IFI6;DCN
cytochalasin B-1122	4/100	0.009292	0.10653	0.019932	0.228502	-1.45829	6.822666	ANXA3;ID1;ID3;PHLDA1
fluphenazine-5234	4/100	0.009292	0.10653	0.019932	0.228502	-1.4577	6.819922	IFI27;IFI6;SOX9;PHLDA1
digoxigenin-4680	4/100	0.009292	0.10653	0.019932	0.228502	-1.45563	6.810256	TXNIP;ID3;FOS;ETS2
furaltadone-3838	4/100	0.009292	0.10653	0.019932	0.228502	-1.4543	6.804016	COL1A1;IFITM1;IFI6;CTGF
adenosine phosphate-1622	4/100	0.009292	0.10653	0.019932	0.228502	-1.45338	6.799694	CCL20;TXNIP;FOS;PHLDA1
lanatoside C-4328	4/100	0.009292	0.10653	0.019932	0.228502	-1.4521	6.793739	JUND;TXNIP;FOS;ETS2
betonicine-4767	4/100	0.009292	0.10653	0.019932	0.228502	-1.45101	6.788644	COL1A1;SPARC;IFI27;IFI6
fludrocortisone-3977	4/100	0.009292	0.10653	0.019932	0.228502	-1.44722	6.770919	IFITM1;IFI27;IFI6;ISG15
digoxigenin-3397	4/100	0.009292	0.10653	0.019932	0.228502	-1.44658	6.767894	ID1;TXNIP;ID3;FOS
fulvestrant-1663	4/100	0.009292	0.10653	0.019932	0.228502	-1.44658	6.767887	RNF43;ID1;ENC1;ID3
griseofulvin-4687	4/100	0.009292	0.10653	0.019932	0.228502	-1.44399	6.755793	IFITM1;IFI27;MMP3;IFI6
idoxuridine-1480	4/100	0.009292	0.10653	0.019932	0.228502	-1.4428	6.750205	C3;CEACAM5;ATP1B1;TSPAN1
eucatropine-3841	4/100	0.009292	0.10653	0.019932	0.228502	-1.44254	6.749011	PIGR;TCN1;CTSE;ITM2C
isotretinoin-7438	4/100	0.009292	0.10653	0.019932	0.228502	-1.4424	6.748366	GPX2;SOX9;TGFBI;PHLDA1
etidronic acid-4387	4/100	0.009292	0.10653	0.019932	0.228502	-1.44196	6.746308	SPARC;IFI27;IFI6;ITM2C
fluoxetine-5356	4/100	0.009292	0.10653	0.019932	0.228502	-1.44141	6.743694	LGALS3BP;JUND;FOS;PHLDA1
bupivacaine-7435	4/100	0.009292	0.10653	0.019932	0.228502	-1.44087	6.741211	BMP4;CEACAM6;TMPRSS3;S100A6
oxaprozin-3794	4/100	0.009292	0.10653	0.019932	0.228502	-1.43767	6.726238	IFITM1;IFI27;CEACAM5;IFI6
lynestrenol-1537	4/100	0.009292	0.10653	0.019932	0.228502	-1.43612	6.718954	SOX9;FOS;PHLDA1;ETS2
fluphenazine-1075	4/100	0.009292	0.10653	0.019932	0.228502	-1.43436	6.710747	JUND;SOX9;PHLDA1;ETS2
cinoxacin-6257	4/100	0.009292	0.10653	0.019932	0.228502	-1.43428	6.710339	COL1A1;IFITM1;IFI6;ISG15
GW-8510-7067	4/100	0.009292	0.10653	0.019932	0.228502	-1.4336	6.707189	APP;CXCL1;FOS;CD24
protriptyline-5438	4/100	0.009292	0.10653	0.019932	0.228502	-1.4333	6.705776	IFITM1;IFI27;IFI6;ISG15
florfenicol-5300	4/100	0.009292	0.10653	0.019932	0.228502	-1.43273	6.703107	AP1S1;S100A4;CTSE;PTMA
digitoxigenin-1339	4/100	0.009292	0.10653	0.019932	0.228502	-1.43227	6.700953	CCL20;TXNIP;FOS;ATP1B1
metoprolol-4508	4/100	0.009292	0.10653	0.019932	0.228502	-1.43226	6.700917	BMP4;IFITM1;JUND;DCN

kinetin-6813	4/100	0.009292	0.10653	0.019932	0.228502	-1.43148	6.697238	IFITM1;IFI27;IFI6;ISG15
lycorine-6051	4/100	0.009292	0.10653	0.019932	0.228502	-1.42921	6.686645	ENC1;TXNIP;SOX9;PHLDA1
clenbuterol-1613	4/100	0.009292	0.10653	0.019932	0.228502	-1.42508	6.6673	CCL20;ISG15;TIMP1;S100A10
ivermectin-7206	4/100	0.009292	0.10653	0.019932	0.228502	-1.42458	6.664974	SPINK1;IFI6;TXNIP;PHLDA1
isopropamide iodide-6781	4/100	0.009292	0.10653	0.019932	0.228502	-1.42415	6.66296	RNF43;IFI27;CEACAM6;IFI6
pimozide-6780	4/100	0.009292	0.10653	0.019932	0.228502	-1.42366	6.660676	TXNIP;FOS;PHLDA1;IER2
labetalol-1550	4/100	0.009292	0.10653	0.019932	0.228502	-1.42226	6.654098	CCL20;SPP1;IFI6;S100A10
lasalocid-4985	4/100	0.009292	0.10653	0.019932	0.228502	-1.42182	6.652049	BMP4;JUND;SOX9;PHLDA1
suloctidil-6675	4/100	0.009292	0.10653	0.019932	0.228502	-1.42066	6.64664	PIGR;CCL20;CXCL1;FOS
ellipticine-2758	4/100	0.009292	0.10653	0.019932	0.228502	-1.42064	6.646551	IFI6;SOX9;FOS;CTGF
rottlerin-941	4/100	0.009292	0.10653	0.019932	0.228502	-1.41951	6.641254	TXNIP;FOS;PHLDA1;ETS2
GW-8510-7080	4/100	0.009292	0.10653	0.019932	0.228502	-1.41878	6.637848	APP;GPX2;SERPINA1;S100P
isotretinoin-7315	4/100	0.009292	0.10653	0.019932	0.228502	-1.4177	6.63279	C3;SPARC;QPCT;DCN
metampicillin-2123	4/100	0.009292	0.10653	0.019932	0.228502	-1.41678	6.62846	GPX2;CCL20;MMP3;AP1S1
halcinonide-4703	4/100	0.009292	0.10653	0.019932	0.228502	-1.41626	6.626049	IFI27;MMP3;IFI6;ITM2C
lanatoside C-2193	4/100	0.009292	0.10653	0.019932	0.228502	-1.41212	6.606694	CCL20;TXNIP;ID3;FOS
isosorbide-2183	4/100	0.009292	0.10653	0.019932	0.228502	-1.41183	6.605325	CCL20;TCN1;TFF2;CTSE
lorglumide-6456	4/100	0.009292	0.10653	0.019932	0.228502	-1.41055	6.599341	TCN1;MMP3;DUOX2;DCN
isotretinoin-2407	4/100	0.009292	0.10653	0.019932	0.228502	-1.40896	6.591875	NQO1;CCL20;ID1;PHLDA1
mimosine-5302	4/100	0.009292	0.10653	0.019932	0.228502	-1.40797	6.587241	JUND;S100A4;CD24;S100A11
danazol-1954	4/100	0.009292	0.10653	0.019932	0.228502	-1.40633	6.579583	SERPINA1;ID1;AP1S1;CD24
haloperidol-1539	4/100	0.009292	0.10653	0.019932	0.228502	-1.40612	6.578591	BMP4;RCN1;CEACAM5;MMP3
tamoxifen-6768	4/100	0.009292	0.10653	0.019932	0.228502	-1.40328	6.565332	CEACAM6;FOS;PHLDA1;ITM2C
kawain-4693	4/100	0.009292	0.10653	0.019932	0.228502	-1.39886	6.544634	IFITM1;IFI27;IFI6;ISG15
sulfaphenazole-1836	4/100	0.009292	0.10653	0.019932	0.228502	-1.3979	6.540167	SLC12A2;FXYD3;ENC1;PHLDA1
lanatoside C-3771	4/100	0.009292	0.10653	0.019932	0.228502	-1.39772	6.539319	JUND;TXNIP;FOS;CTGF
naftidrofuryl-5287	4/100	0.009292	0.10653	0.019932	0.228502	-1.39361	6.520084	IFITM1;IFI6;ISG15;CD24
naftifine-7273	4/100	0.009292	0.10653	0.019932	0.228502	-1.39338	6.519017	APP;CEACAM6;AP1S1;CTGF
ketoprofen-3729	4/100	0.009292	0.10653	0.019932	0.228502	-1.39333	6.518759	APP;MMP3;AP1S1;SLCO5A1
mometasone-1746	4/100	0.009292	0.10653	0.019932	0.228502	-1.39315	6.517949	CCL20;ACSL1;IFNGR1;TXNIP
mefloquine-1364	4/100	0.009292	0.10653	0.019932	0.228502	-1.39191	6.512106	RPS14P3;SPP1;IFI6;ISG15
thiamine-1744	4/100	0.009292	0.10653	0.019932	0.228502	-1.39097	6.507723	IFITM3;APP;IFITM1;CKB
prenylamine-2886	4/100	0.009292	0.10653	0.019932	0.228502	-1.38998	6.503106	SOX9;FOS;IER2;ETS2
midodrine-2087	4/100	0.009292	0.10653	0.019932	0.228502	-1.38989	6.502653	BMP4;APP;TSPAN8;CCL20
nocodazole-2239	4/100	0.009292	0.10653	0.019932	0.228502	-1.38977	6.50209	RPL28;PHLDA1;S100A11;ETS2
pentetic acid-5264	4/100	0.009292	0.10653	0.019932	0.228502	-1.38877	6.49742	COL1A1;PIGR;IFITM1;IFI6
oxantel-5338	4/100	0.009292	0.10653	0.019932	0.228502	-1.38816	6.494576	RNF43;IFITM1;CEACAM6;S100A4

hydrocotarnine-6827	4/100	0.009292	0.10653	0.019932	0.228502	-1.388	6.493826	IFITM1;IFI27;IFI6;ISG15
methotrexate-2041	4/100	0.009292	0.10653	0.019932	0.228502	-1.38694	6.488867	NQO1;APP;FOS;ITM2B
MS-275-7084	4/100	0.009292	0.10653	0.019932	0.228502	-1.38602	6.484585	MMP1;ENC1;ETS2;CTGF
lanatoside C-3963	4/100	0.009292	0.10653	0.019932	0.228502	-1.38559	6.482538	TXNIP;FOS;IER2;CTGF
prochlorperazine-5010	4/100	0.009292	0.10653	0.019932	0.228502	-1.38459	6.477894	IFITM1;IFI27;IFI6;ISG15
monorden-953	4/100	0.009292	0.10653	0.019932	0.228502	-1.38097	6.46094	COL1A1;BMP4;GPX2;ETS2
pivmecillinam-3535	4/100	0.009292	0.10653	0.019932	0.228502	-1.37895	6.451472	MMP3;S100A6;AP1S1;S100A11
miconazole-1477	4/100	0.009292	0.10653	0.019932	0.228502	-1.37773	6.445773	FOS;PHLDA1;IER2;ETS2
pyrvinium-5439	4/100	0.009292	0.10653	0.019932	0.228502	-1.37766	6.445444	JUND;ID3;S100P;ETS2
phenoxybenzamine-5248	4/100	0.009292	0.10653	0.019932	0.228502	-1.37592	6.437332	SOX9;FOS;ETS2;CTGF
merbromin-2577	4/100	0.009292	0.10653	0.019932	0.228502	-1.3745	6.430669	APP;IFITM1;GPX2;SPP1
suloctidil-5021	4/100	0.009292	0.10653	0.019932	0.228502	-1.37323	6.424751	SOX9;FOS;PHLDA1;IER2
quinpirole-2977	4/100	0.009292	0.10653	0.019932	0.228502	-1.37203	6.419117	ANXA2;ID3;TIMP1;PHLDA1
etofenamate-4108	4/100	0.009292	0.10653	0.019932	0.228502	-1.37015	6.410303	C3;SPINK1;FOS;PHLDA1
lanatoside C-3852	4/100	0.009292	0.10653	0.019932	0.228502	-1.37009	6.410039	TXNIP;FOS;IER2;CTGF
sisomicin-2493	4/100	0.009292	0.10653	0.019932	0.228502	-1.36912	6.405519	GPX2;CEACAM5;MMP3;TFF2
methantheliniun bromide-6254	4/100	0.009292	0.10653	0.019932	0.228502	-1.36601	6.390941	BMP4;IFITM1;IFI6;ISG15
spiramycin-2558	4/100	0.009292	0.10653	0.019932	0.228502	-1.36546	6.38839	PIGR;IFITM1;CCL20;IGFBP2
selegiline-4146	4/100	0.009292	0.10653	0.019932	0.228502	-1.36535	6.387885	IFITM1;IFI27;IFI6;ISG15
PNU-0293363-6573	4/100	0.009292	0.10653	0.019932	0.228502	-1.36326	6.378103	APP;HSP90AB1;GAPDH;ACTB
tanespimycin-1167	4/100	0.009292	0.10653	0.019932	0.228502	-1.36292	6.376492	APP;HSP90AB1;CCL20;HSPB1
pramocaine-2198	4/100	0.009292	0.10653	0.019932	0.228502	-1.3624	6.374052	APP;TCN1;CEACAM6;TFF2
proglumide-3972	4/100	0.009292	0.10653	0.019932	0.228502	-1.36135	6.369146	COL1A1;C3;IFI27;IFI6
trichostatin A-3395	4/100	0.009292	0.10653	0.019932	0.228502	-1.3613	6.368936	TXNIP;FOS;ITM2C;PSMB9
thiocolchicoside-5520	4/100	0.009292	0.10653	0.019932	0.228502	-1.35877	6.357059	IFITM1;IFI6;S100A4;ISG15
piromidic acid-2996	4/100	0.009292	0.10653	0.019932	0.228502	-1.35564	6.342447	SPINK1;TFF2;CD24;DCN
staurosporine-312	4/100	0.009292	0.10653	0.019932	0.228502	-1.35537	6.341162	APP;S100A6;AP1S1;CTGF
ouabain-1302	4/100	0.009292	0.10653	0.019932	0.228502	-1.35524	6.34056	CCL20;TXNIP;FOS;ATP1B1
STOCK1N-28457-6906	4/100	0.009292	0.10653	0.019932	0.228502	-1.35348	6.332323	APP;S100A4;PTMA;PHLDA1
syrosingopine-5733	4/100	0.009292	0.10653	0.019932	0.228502	-1.35255	6.327955	LUM;CEACAM5;S100P;CD24
thalidomide-5990	4/100	0.009292	0.10653	0.019932	0.228502	-1.35203	6.325539	CEACAM6;MMP3;SECTM1;DCN
trichostatin A-3791	4/100	0.009292	0.10653	0.019932	0.228502	-1.34938	6.313163	LUM;MMP1;CTGF;PSMB9
thioridazine-1655	4/100	0.009292	0.10653	0.019932	0.228502	-1.34801	6.306745	SOX9;FOS;PHLDA1;ETS2
phenoxybenzamine-5613	4/100	0.009292	0.10653	0.019932	0.228502	-1.34796	6.306525	SOX9;FOS;PHLDA1;ETS2
trichostatin A-2268	4/100	0.009292	0.10653	0.019932	0.228502	-1.34639	6.299162	IFI6;TXNIP;ITM2C;PSMB9
oxetacaine-4829	4/100	0.009292	0.10653	0.019932	0.228502	-1.34582	6.296474	TMPRSS3;MMP3;IFI6;PHLDA1
vanoxerine-3240	4/100	0.009292	0.10653	0.019932	0.228502	-1.34072	6.272644	IFI27;IFI6;FOS;IER2

rescinnamine-2785	4/100	0.009292	0.10653	0.019932	0.228502	-1.34006	6.26953	IFITM1;IFI27;IFI6;ISG15
proscillaridin-4404	4/100	0.009292	0.10653	0.019932	0.228502	-1.33802	6.260005	TXNIP;FOS;IER2;CTGF
tretinoin-6931	4/100	0.009292	0.10653	0.019932	0.228502	-1.33795	6.259675	ID1;SOX9;TGFBI;PHLDA1
sitosterol-4073	4/100	0.009292	0.10653	0.019932	0.228502	-1.33738	6.257	SERPINA1;CEACAM5;SLCO5A1;FOS
tretinoin-966	4/100	0.009292	0.10653	0.019932	0.228502	-1.33725	6.256381	ID1;SOX9;TGFBI;PHLDA1
trichostatin A-5065	4/100	0.009292	0.10653	0.019932	0.228502	-1.33553	6.248332	BMP4;MMP1;CTGF;PSMB9
trichostatin A-6454	4/100	0.009292	0.10653	0.019932	0.228502	-1.33343	6.238539	IFI6;TXNIP;ITM2C;PSMB9
STOCK1N-35696-6577	4/100	0.009292	0.10653	0.019932	0.228502	-1.33208	6.232218	BMP4;TXNIP;AP1S1;SECTM1
proadifen-7165	4/100	0.009292	0.10653	0.019932	0.228502	-1.33105	6.227373	CEACAM6;QPCT;SOX9;FOS
trichostatin A-4302	4/100	0.009292	0.10653	0.019932	0.228502	-1.33095	6.226925	BMP4;LUM;CTGF;PSMB9
tretinoin-991	4/100	0.009292	0.10653	0.019932	0.228502	-1.32804	6.213328	ID1;SOX9;TGFBI;PHLDA1
trichostatin A-2375	4/100	0.009292	0.10653	0.019932	0.228502	-1.32567	6.202206	TSPAN13;CKB;ATP1B1;CD24
suloctidil-1297	4/100	0.009292	0.10653	0.019932	0.228502	-1.32424	6.195514	IFITM1;IFI6;ISG15;LY6E
trifluoperazine-1649	4/100	0.009292	0.10653	0.019932	0.228502	-1.3213	6.181784	SOX9;FOS;PHLDA1;ETS2
trichostatin A-1612	4/100	0.009292	0.10653	0.019932	0.228502	-1.32063	6.178641	TSPAN13;CCL20;ATP1B1;ITM2C
tanespimycin-1206	4/100	0.009292	0.10653	0.019932	0.228502	-1.32032	6.177186	APP;HSP90AB1;HSPB1;CTGF
tretinoin-6170	4/100	0.009292	0.10653	0.019932	0.228502	-1.31966	6.17408	CCL20;ID1;TIMP1;PHLDA1
trifluoperazine-910	4/100	0.009292	0.10653	0.019932	0.228502	-1.319	6.171013	COL1A1;SOX9;FOS;PHLDA1
trifluoperazine-5221	4/100	0.009292	0.10653	0.019932	0.228502	-1.31331	6.144386	IFITM1;IFI6;SOX9;PHLDA1
trichostatin A-5981	4/100	0.009292	0.10653	0.019932	0.228502	-1.31316	6.143669	MMP1;FOS;CTGF;PSMB9
trichostatin A-2247	4/100	0.009292	0.10653	0.019932	0.228502	-1.30833	6.121079	IFI6;TXNIP;ITM2C;PSMB9
trihexyphenidyl-2158	4/100	0.009292	0.10653	0.019932	0.228502	-1.30819	6.120429	APP;IFITM1;IFI6;ID3
trichostatin A-3746	4/100	0.009292	0.10653	0.019932	0.228502	-1.30763	6.117832	CCL20;MMP1;FOS;CTGF
tretinoin-6971	4/100	0.009292	0.10653	0.019932	0.228502	-1.30595	6.109971	ID1;SOX9;TGFBI;PHLDA1
tanespimycin-521	4/100	0.009292	0.10653	0.019932	0.228502	-1.30497	6.105371	IFITM3;HSP90AB1;CKS2;S100A4
tretinoin-2671	4/100	0.009292	0.10653	0.019932	0.228502	-1.30452	6.103252	ACSL1;ID1;IFI6;TIMP1
thiethylperazine-3576	4/100	0.009292	0.10653	0.019932	0.228502	-1.30411	6.101361	IFITM1;IFI27;IFI6;ISG15
trifluoperazine-1165	4/100	0.009292	0.10653	0.019932	0.228502	-1.30385	6.100141	JUND;QPCT;SPP1;ATP1B1
tropicamide-3619	4/100	0.009292	0.10653	0.019932	0.228502	-1.30355	6.098746	PIGR;IFITM1;IFI27;IFI6
thioridazine-1171	4/100	0.009292	0.10653	0.019932	0.228502	-1.30191	6.091065	CCL20;ISG15;CD14;PHLDA1
trichostatin A-7387	4/100	0.009292	0.10653	0.019932	0.228502	-1.29938	6.079242	CCL20;MMP1;CTGF;PSMB9
trichostatin A-6143	4/100	0.009292	0.10653	0.019932	0.228502	-1.29802	6.072877	TSPAN13;QPCT;ID3;ATP1B1
tretinoin-1636	4/100	0.009292	0.10653	0.019932	0.228502	-1.2959	6.062916	ID1;SOX9;TGFBI;PHLDA1
trichostatin A-1153	4/100	0.009292	0.10653	0.019932	0.228502	-1.29453	6.056516	TSPAN13;CCL20;ID3;ATP1B1
trichostatin A-6820	4/100	0.009292	0.10653	0.019932	0.228502	-1.28568	6.015123	TXNIP;CTGF;ITM2C;PSMB9
tretinoin-1548	4/100	0.009292	0.10653	0.019932	0.228502	-1.28533	6.013481	CCL20;ACSL1;ID1;TIMP1
valproic acid-1155	4/100	0.009292	0.10653	0.019932	0.228502	-1.2793	5.985283	TSPAN13;QPCT;MMP3;LY6E

trichostatin A-1971	4/100	0.009292	0.10653	0.019932	0.228502	-1.27838	5.98096	TSPAN13;CKB;ATP1B1;ITM2C
tobramycin-4081	4/100	0.009292	0.10653	0.019932	0.228502	-1.27676	5.973399	IL32;C3;SERPINA1;TSPAN8
trichostatin A-4348	4/100	0.009292	0.10653	0.019932	0.228502	-1.27268	5.954282	PIGR;IFITM1;IFI6;PHLDA1
tanespimycin-6166	4/100	0.009292	0.10653	0.019932	0.228502	-1.26761	5.930576	PIGR;HSP90AB1;HSPB1;TGFBI
trifluoperazine-2684	4/100	0.009292	0.10653	0.019932	0.228502	-1.26607	5.923374	ANXA2;CCL20;TCN1;SPP1
vinburnine-2781	4/100	0.009292	0.10653	0.019932	0.228502	-1.26404	5.913895	COL1A1;CEACAM5;AP1S1;TFF2
0175029-0000-4713	3/100	0.049183	0.248146	0.083039	0.418966	-1.3416	4.041183	IFI6;CD24;S100A11
atracurium besilate-7477	3/100	0.049183	0.248146	0.083039	0.418966	-1.32297	3.985084	IFITM1;S100A6;S100A4
15-delta prostaglandin J2-1172	3/100	0.049183	0.248146	0.083039	0.418966	-1.31287	3.954654	NQO1;ANXA2;ID3
(+)-chelidonine-6236	3/100	0.049183	0.248146	0.083039	0.418966	-1.31243	3.953327	IFI6;AP1S1;TGFBI
15-delta prostaglandin J2-1231	3/100	0.049183	0.248146	0.083039	0.418966	-1.30855	3.941635	HSPB1;FOS;CTGF
5252917-944	3/100	0.049183	0.248146	0.083039	0.418966	-1.3	3.915886	GPX2;TGFBI;PHLDA1
(-)-isoprenaline-6149	3/100	0.049183	0.248146	0.083039	0.418966	-1.29873	3.912056	CCL20;ID3;PHLDA1
alpha-estradiol-5570	3/100	0.049183	0.248146	0.083039	0.418966	-1.29558	3.902579	COL1A1;IFI6;OLFM4
15-delta prostaglandin J2-1011	3/100	0.049183	0.248146	0.083039	0.418966	-1.29239	3.892959	RCN1;GPX2;ETS2
albendazole-1547	3/100	0.049183	0.248146	0.083039	0.418966	-1.29148	3.890205	TPI1;ANXA2;CCL20
altretamine-6467	3/100	0.049183	0.248146	0.083039	0.418966	-1.28909	3.883027	BMP4;IFI6;PDZK1IP1
5707885-6385	3/100	0.049183	0.248146	0.083039	0.418966	-1.28812	3.880103	PTPRO;CTSE;PHLDA1
ambroxol-6719	3/100	0.049183	0.248146	0.083039	0.418966	-1.2861	3.874018	TPI1;MMP3;ITM2B
0175029-0000-3694	3/100	0.049183	0.248146	0.083039	0.418966	-1.28302	3.864746	APP;SPINK1;DUOX2
ampyrone-6845	3/100	0.049183	0.248146	0.083039	0.418966	-1.28202	3.861728	IFITM1;IFI27;IFI6
alimemazine-5881	3/100	0.049183	0.248146	0.083039	0.418966	-1.27837	3.850729	JUND;TSPAN8;SPP1
antimycin A-5472	3/100	0.049183	0.248146	0.083039	0.418966	-1.27704	3.84673	C3;CTSE;TSPAN1
acebutolol-1993	3/100	0.049183	0.248146	0.083039	0.418966	-1.2764	3.84478	CEACAM5;CKB;LY6E
0179445-0000-3630	3/100	0.049183	0.248146	0.083039	0.418966	-1.27571	3.842717	PIGR;IFI6;TXNIP
5248896-838	3/100	0.049183	0.248146	0.083039	0.418966	-1.27523	3.841274	IFITM1;IFI6;ISG15
acebutolol-4976	3/100	0.049183	0.248146	0.083039	0.418966	-1.27441	3.838802	BMP4;CEACAM5;AP1S1
(-)-MK-801-5003	3/100	0.049183	0.248146	0.083039	0.418966	-1.27093	3.828306	MMP3;IFI6;CTGF
bepridil-2629	3/100	0.049183	0.248146	0.083039	0.418966	-1.26927	3.823318	MMP12;FOS;IER2
6-benzylaminopurine-3623	3/100	0.049183	0.248146	0.083039	0.418966	-1.26797	3.819399	SERPINA1;CTSE;DCN
acenocoumarol-1394	3/100	0.049183	0.248146	0.083039	0.418966	-1.26777	3.81881	RPS14P3;GPX2;AP1S1
atropine methonitrate-6495	3/100	0.049183	0.248146	0.083039	0.418966	-1.26697	3.816399	PIGR;CEACAM5;DCN
atovaquone-4786	3/100	0.049183	0.248146	0.083039	0.418966	-1.26644	3.814776	COL1A1;SPINK1;PHLDA1
0317956-0000-3969	3/100	0.049183	0.248146	0.083039	0.418966	-1.26445	3.808789	SERPINA1;IFI27;SECTM1
benzethonium chloride-6070	3/100	0.049183	0.248146	0.083039	0.418966	-1.26254	3.80305	TXNIP;FOS;PHLDA1
acepromazine-2769	3/100	0.049183	0.248146	0.083039	0.418966	-1.26216	3.801905	C3;TCN1;PDZK1IP1
alvespimycin-1154	3/100	0.049183	0.248146	0.083039	0.418966	-1.25904	3.792506	APP;HSP90AB1;HSPB1

alverine-2110	3/100	0.049183	0.248146	0.083039	0.418966	-1.25766	3.788337	APP;IFI6;AP1S1
benfluorex-6727	3/100	0.049183	0.248146	0.083039	0.418966	-1.25622	3.784015	IL32;IFI27;FOS
6-azathymine-2466	3/100	0.049183	0.248146	0.083039	0.418966	-1.25618	3.783897	GPX2;COL1A2;MMP3
alpha-estradiol-122	3/100	0.049183	0.248146	0.083039	0.418966	-1.25605	3.783506	PIGR;GPX2;CEACAM5
cantharidin-3075	3/100	0.049183	0.248146	0.083039	0.418966	-1.25599	3.783314	FOS;ATP1B1;IER2
3-hydroxy-DL-kynurenine-1300	3/100	0.049183	0.248146	0.083039	0.418966	-1.2553	3.78122	SLC12A2;APP;ITM2B
bezafibrate-2630	3/100	0.049183	0.248146	0.083039	0.418966	-1.25505	3.780484	C3;TCN1;CEACAM5
AG-028671-6587	3/100	0.049183	0.248146	0.083039	0.418966	-1.25155	3.769928	APP;TXNIP;ACTB
clomipramine-4487	3/100	0.049183	0.248146	0.083039	0.418966	-1.25146	3.769662	IFITM1;CEACAM5;DCN
blebbistatin-837	3/100	0.049183	0.248146	0.083039	0.418966	-1.25126	3.769063	TMPRSS3;TFF2;DCN
calycanthine-6221	3/100	0.049183	0.248146	0.083039	0.418966	-1.25097	3.768197	TMPRSS3;IFI6;ITM2B
3-hydroxy-DL-kynurenine-2654	3/100	0.049183	0.248146	0.083039	0.418966	-1.2509	3.767969	COL1A1;TCN1;CTSE
calmidazolium-486	3/100	0.049183	0.248146	0.083039	0.418966	-1.25029	3.766137	ID1;ID3;FOS
buflomedil-3274	3/100	0.049183	0.248146	0.083039	0.418966	-1.2497	3.764378	RCN1;S100A6;DCN
5162773-892	3/100	0.049183	0.248146	0.083039	0.418966	-1.24738	3.757375	COL1A1;ENC1;PHLDA1
alvespimycin-1213	3/100	0.049183	0.248146	0.083039	0.418966	-1.24677	3.755544	HSPB1;LYZ;CTGF
antazoline-3173	3/100	0.049183	0.248146	0.083039	0.418966	-1.24492	3.74998	MMP3;TFF2;LYZ
adenosine phosphate-6760	3/100	0.049183	0.248146	0.083039	0.418966	-1.24443	3.748502	SERPINA1;LUM;S100P
amiloride-3990	3/100	0.049183	0.248146	0.083039	0.418966	-1.24414	3.747617	C3;IFI27;IFI6
bisacodyl-3297	3/100	0.049183	0.248146	0.083039	0.418966	-1.24232	3.742148	ID1;TXNIP;SOX9
astemizole-1365	3/100	0.049183	0.248146	0.083039	0.418966	-1.24127	3.738971	ANXA2;CCL20;SPP1
alvespimycin-1638	3/100	0.049183	0.248146	0.083039	0.418966	-1.23754	3.727733	BMP4;SLPI;TXNIP
bergenin-7224	3/100	0.049183	0.248146	0.083039	0.418966	-1.2374	3.727321	IFI6;DCN;CTGF
chlorpropamide-5391	3/100	0.049183	0.248146	0.083039	0.418966	-1.23693	3.725889	COL1A1;IFITM1;IFI6
clenbuterol-5266	3/100	0.049183	0.248146	0.083039	0.418966	-1.23649	3.72456	COL1A1;SOX9;DCN
butyl hydroxybenzoate-5608	3/100	0.049183	0.248146	0.083039	0.418966	-1.23612	3.723471	COL1A1;PIGR;DUOX2
ceftazidime-5473	3/100	0.049183	0.248146	0.083039	0.418966	-1.23557	3.721792	COL1A2;SERPINA1;CEACAM5
danazol-5315	3/100	0.049183	0.248146	0.083039	0.418966	-1.2348	3.719486	SERPINA1;IFI6;SOX9
chenodeoxycholic acid-6012	3/100	0.049183	0.248146	0.083039	0.418966	-1.23368	3.716123	TCN1;SECTM1;SOX9
arachidonyltrifluoromethane-594	3/100	0.049183	0.248146	0.083039	0.418966	-1.23295	3.713921	SLPI;CEACAM5;TXNIP
betonicine-3745	3/100	0.049183	0.248146	0.083039	0.418966	-1.23292	3.713826	COL1A1;PIGR;CEACAM5
chloroquine-2869	3/100	0.049183	0.248146	0.083039	0.418966	-1.23214	3.711484	IFITM1;AP1S1;ITM2C
astemizole-4471	3/100	0.049183	0.248146	0.083039	0.418966	-1.23195	3.710904	JUND;S100P;FOS
chlorcyclizine-3893	3/100	0.049183	0.248146	0.083039	0.418966	-1.23042	3.706284	SERPINA1;FOS;CTSE
arecoline-6322	3/100	0.049183	0.248146	0.083039	0.418966	-1.22877	3.701331	IFI6;AP1S1;PSMB9
bretylium tosilate-5020	3/100	0.049183	0.248146	0.083039	0.418966	-1.22852	3.700562	APP;AP1S1;CTGF
anisomycin-1304	3/100	0.049183	0.248146	0.083039	0.418966	-1.22565	3.691925	CCL20;FOS;PHLDA1

BAS-012416453-6880	3/100	0.049183	0.248146	0.083039	0.418966	-1.22382	3.686414	APP;JUND;IFI6
ampicillin-5408	3/100	0.049183	0.248146	0.083039	0.418966	-1.22376	3.686243	COL1A1;IFI6;CTGF
corticosterone-3244	3/100	0.049183	0.248146	0.083039	0.418966	-1.22254	3.682558	ID1;ENC1;ID3
alpha-estradiol-1048	3/100	0.049183	0.248146	0.083039	0.418966	-1.22217	3.681433	TMPRSS3;PTMA;ETS2
amitriptyline-5453	3/100	0.049183	0.248146	0.083039	0.418966	-1.22194	3.680736	C3;FOS;S100A11
canadine-2163	3/100	0.049183	0.248146	0.083039	0.418966	-1.22135	3.678962	IGFBP2;IFI6;S100A11
ciclopirox-3317	3/100	0.049183	0.248146	0.083039	0.418966	-1.2204	3.676119	SOX9;FOS;PGM1
alprostadil-4099	3/100	0.049183	0.248146	0.083039	0.418966	-1.21714	3.666285	BMP4;C3;S100P
doxorubicin-3291	3/100	0.049183	0.248146	0.083039	0.418966	-1.21561	3.661685	COL1A1;AP1S1;S100A11
ciprofibrate-3561	3/100	0.049183	0.248146	0.083039	0.418966	-1.21539	3.661011	COL1A1;C3;S100A11
clioquinol-5623	3/100	0.049183	0.248146	0.083039	0.418966	-1.2131	3.654128	FOS;CTSE;PHLDA1
denatonium benzoate-6502	3/100	0.049183	0.248146	0.083039	0.418966	-1.21294	3.653649	RNF43;MMP3;TFF2
cicloheximide-2723	3/100	0.049183	0.248146	0.083039	0.418966	-1.21072	3.646956	TXNIP;ID3;PHLDA1
chlorpromazine-5214	3/100	0.049183	0.248146	0.083039	0.418966	-1.21022	3.645452	RNF43;APP;OLFM4
bupropion-6256	3/100	0.049183	0.248146	0.083039	0.418966	-1.20821	3.6394	IFITM1;IFI6;ISG15
CP-690334-01-3826	3/100	0.049183	0.248146	0.083039	0.418966	-1.2075	3.637242	PIGR;JUND;CTSE
diazoxide-2052	3/100	0.049183	0.248146	0.083039	0.418966	-1.20738	3.636898	BMP4;FOS;OLFM4
cytisine-1766	3/100	0.049183	0.248146	0.083039	0.418966	-1.20658	3.634491	IFITM3;APP;AP1S1
diclofenac-333	3/100	0.049183	0.248146	0.083039	0.418966	-1.20648	3.634186	IFITM1;SERPINA1;AP1S1
butacaine-2728	3/100	0.049183	0.248146	0.083039	0.418966	-1.20581	3.632159	GPX2;CEACAM5;SECTM1
crotamiton-3050	3/100	0.049183	0.248146	0.083039	0.418966	-1.20454	3.628345	NQO1;IFI6;CD24
anisomycin-6764	3/100	0.049183	0.248146	0.083039	0.418966	-1.20428	3.627556	TXNIP;FOS;CTGF
cyproheptadine-5340	3/100	0.049183	0.248146	0.083039	0.418966	-1.20271	3.622833	BMP4;FOS;PHLDA1
citiolone-3836	3/100	0.049183	0.248146	0.083039	0.418966	-1.20082	3.617123	IFITM1;TCN1;IFI6
clemizole-2301	3/100	0.049183	0.248146	0.083039	0.418966	-1.20075	3.616911	COL1A1;CEACAM5;PHLDA1
captopril-4410	3/100	0.049183	0.248146	0.083039	0.418966	-1.19781	3.608071	COL1A1;APP;ITM2C
flavoxate-7405	3/100	0.049183	0.248146	0.083039	0.418966	-1.19425	3.597339	IFITM1;SPINK1;ID1
colchicine-1598	3/100	0.049183	0.248146	0.083039	0.418966	-1.19396	3.596473	ANXA2;CCL20;PHLDA1
cefotiam-5361	3/100	0.049183	0.248146	0.083039	0.418966	-1.1922	3.591163	SERPINA1;CEACAM6;ETS2
diphenhydramine-1708	3/100	0.049183	0.248146	0.083039	0.418966	-1.19203	3.590651	BMP4;APP;TXNIP
epirizole-5995	3/100	0.049183	0.248146	0.083039	0.418966	-1.19122	3.588224	IFITM1;IFI27;IFI6
econazole-6008	3/100	0.049183	0.248146	0.083039	0.418966	-1.19	3.584526	CEACAM6;FOS;PHLDA1
chlorhexidine-2025	3/100	0.049183	0.248146	0.083039	0.418966	-1.18956	3.583212	NQO1;APP;ITM2B
cloperastine-4271	3/100	0.049183	0.248146	0.083039	0.418966	-1.18435	3.567528	JUND;CEACAM5;PHLDA1
clorsulon-7264	3/100	0.049183	0.248146	0.083039	0.418966	-1.18174	3.55966	APP;AP1S1;ITM2B
cloperastine-4732	3/100	0.049183	0.248146	0.083039	0.418966	-1.18138	3.558561	FOS;PHLDA1;IER2
beta-escin-3807	3/100	0.049183	0.248146	0.083039	0.418966	-1.18127	3.558239	JUND;TSPAN8;FOS

clomifene-1269	3/100	0.049183	0.248146	0.083039	0.418966	-1.17954	3.553027	AP1S1;ATP1B1;ITM2B
dacarbazine-6816	3/100	0.049183	0.248146	0.083039	0.418966	-1.17754	3.546995	TFF2;SOX9;DCN
colchicine-630	3/100	0.049183	0.248146	0.083039	0.418966	-1.17607	3.542573	JUND;CXCL1;CTGF
decamethonium bromide-4094	3/100	0.049183	0.248146	0.083039	0.418966	-1.1755	3.540864	APP;SERPINA1;CEACAM5
estradiol-5905	3/100	0.049183	0.248146	0.083039	0.418966	-1.17431	3.537269	TCN1;TFF2;ETS2
famprofazone-4309	3/100	0.049183	0.248146	0.083039	0.418966	-1.17047	3.5257	IL32;COL1A2;PHLDA1
clindamycin-7172	3/100	0.049183	0.248146	0.083039	0.418966	-1.17002	3.524351	RCN1;CEACAM6;CEACAM5
CP-319743-7491	3/100	0.049183	0.248146	0.083039	0.418966	-1.16944	3.522611	JUND;CEACAM6;IFI6
flunisolide-2168	3/100	0.049183	0.248146	0.083039	0.418966	-1.16939	3.522468	CCL20;TFF2;CD24
cyclobenzaprine-1332	3/100	0.049183	0.248146	0.083039	0.418966	-1.16871	3.520398	ANXA3;TCN1;IFI6
cytisine-2759	3/100	0.049183	0.248146	0.083039	0.418966	-1.16683	3.51473	COL1A1;IFI6;TFF2
famprofazone-3834	3/100	0.049183	0.248146	0.083039	0.418966	-1.16652	3.513814	COL1A1;IFITM1;IFI6
clopamide-5402	3/100	0.049183	0.248146	0.083039	0.418966	-1.16608	3.512473	C3;MMP3;SOX9
etodolac-1407	3/100	0.049183	0.248146	0.083039	0.418966	-1.16597	3.512156	RPS14P3;TPI1;PTMA
dicoumarol-3941	3/100	0.049183	0.248146	0.083039	0.418966	-1.1641	3.506522	IFITM1;IFI27;IFI6
dicycloverine-4405	3/100	0.049183	0.248146	0.083039	0.418966	-1.1602	3.494758	RCN1;FOS;ETS2
disulfiram-1369	3/100	0.049183	0.248146	0.083039	0.418966	-1.15535	3.480153	NQO1;HSPB1;S100A10
CP-645525-01-7522	3/100	0.049183	0.248146	0.083039	0.418966	-1.15343	3.474392	RNF43;SOX9;TSPAN1
hycanthone-4630	3/100	0.049183	0.248146	0.083039	0.418966	-1.1506	3.465842	IFITM1;IFI27;IFI6
etodolac-2091	3/100	0.049183	0.248146	0.083039	0.418966	-1.1497	3.46315	COL1A2;SERPINA1;IFI6
cotinine-1929	3/100	0.049183	0.248146	0.083039	0.418966	-1.14965	3.463008	AP1S1;ETS2;LY6E
enoxacin-1597	3/100	0.049183	0.248146	0.083039	0.418966	-1.14781	3.457458	APP;PHLDA1;ITM2B
flecainide-3418	3/100	0.049183	0.248146	0.083039	0.418966	-1.14748	3.456465	C3;RNF43;ITM2C
fluorometholone-6247	3/100	0.049183	0.248146	0.083039	0.418966	-1.14737	3.456138	COL1A1;IFI6;S100P
cefoperazone-1627	3/100	0.049183	0.248146	0.083039	0.418966	-1.14706	3.455204	CCL20;SPP1;CKB
dihydroergotamine-1398	3/100	0.049183	0.248146	0.083039	0.418966	-1.14612	3.452373	ANXA2;CCL20;TIMP1
dihydroergocristine-1745	3/100	0.049183	0.248146	0.083039	0.418966	-1.14597	3.451895	CCL20;TIMP1;FOS
denatonium benzoate-5480	3/100	0.049183	0.248146	0.083039	0.418966	-1.14489	3.448646	IFI27;IFI6;TGFBI
fenofibrate-7474	3/100	0.049183	0.248146	0.083039	0.418966	-1.14453	3.447573	MMP7;MMP3;TSPAN1
dinoprostone-6547	3/100	0.049183	0.248146	0.083039	0.418966	-1.14389	3.445643	AP1S1;TFF1;S100P
ginkgolide A-3260	3/100	0.049183	0.248146	0.083039	0.418966	-1.14371	3.44511	IFITM1;IFI27;IFI6
digitoxigenin-4217	3/100	0.049183	0.248146	0.083039	0.418966	-1.14336	3.444034	TXNIP;FOS;CTGF
diethylstilbestrol-4369	3/100	0.049183	0.248146	0.083039	0.418966	-1.14292	3.442732	FOS;PHLDA1;CTGF
diclofenamide-5286	3/100	0.049183	0.248146	0.083039	0.418966	-1.14204	3.440073	BMP4;JUND;S100A4
furaltadone-2554	3/100	0.049183	0.248146	0.083039	0.418966	-1.14163	3.438833	COL1A2;ID3;DCN
daunorubicin-4983	3/100	0.049183	0.248146	0.083039	0.418966	-1.14145	3.438284	SPINK1;MMP3;S100A11
emetine-4243	3/100	0.049183	0.248146	0.083039	0.418966	-1.14126	3.437724	ENC1;TXNIP;ID3

digoxin-5324	3/100	0.049183	0.248146	0.083039	0.418966	-1.13967	3.432925	TXNIP;FOS;IER2
etomidate-2958	3/100	0.049183	0.248146	0.083039	0.418966	-1.13773	3.427093	TCN1;MMP3;ATP1B1
estradiol-6200	3/100	0.049183	0.248146	0.083039	0.418966	-1.1366	3.423691	COL1A1;GPX2;TFF2
fisetin-579	3/100	0.049183	0.248146	0.083039	0.418966	-1.13576	3.421139	APP;CDH3;S100A11
fluvastatin-5290	3/100	0.049183	0.248146	0.083039	0.418966	-1.13384	3.415375	APP;IFITM1;IFI6
doxycycline-3479	3/100	0.049183	0.248146	0.083039	0.418966	-1.13103	3.406907	IFI6;SOX9;ITM2C
etynodiol-3102	3/100	0.049183	0.248146	0.083039	0.418966	-1.13024	3.404522	MMP7;CCL20;ACSL1
gentamicin-7237	3/100	0.049183	0.248146	0.083039	0.418966	-1.13004	3.403918	SERPINA1;CEACAM6;AP1S1
hexamethonium bromide-4965	3/100	0.049183	0.248146	0.083039	0.418966	-1.12684	3.394297	BMP4;SPINK1;TFF2
HC toxin-909	3/100	0.049183	0.248146	0.083039	0.418966	-1.125	3.388756	TXNIP;ID3;ITM2C
geldanamycin-5225	3/100	0.049183	0.248146	0.083039	0.418966	-1.12458	3.387477	IFITM1;IFI27;IFI6
famotidine-6665	3/100	0.049183	0.248146	0.083039	0.418966	-1.12369	3.384793	QPCT;FOS;S100A11
finasteride-3641	3/100	0.049183	0.248146	0.083039	0.418966	-1.12365	3.384667	C3;IFI27;IFI6
fenoprofen-3714	3/100	0.049183	0.248146	0.083039	0.418966	-1.12226	3.380483	IL32;SPARC;SLCO5A1
enilconazole-6518	3/100	0.049183	0.248146	0.083039	0.418966	-1.12183	3.379191	RNF43;SOX9;OLFM4
fenspiride-2106	3/100	0.049183	0.248146	0.083039	0.418966	-1.1209	3.376389	COL1A1;RNF43;IFITM1
fulvestrant-985	3/100	0.049183	0.248146	0.083039	0.418966	-1.12086	3.376259	RNF43;TPI1;ID1
guaifenesin-3897	3/100	0.049183	0.248146	0.083039	0.418966	-1.11693	3.364422	SLPI;SPP1;PSMB9
fluvoxamine-3995	3/100	0.049183	0.248146	0.083039	0.418966	-1.11653	3.363227	IL32;BMP4;JUND
guanabenz-2045	3/100	0.049183	0.248146	0.083039	0.418966	-1.11476	3.35789	CEACAM5;QPCT;ITM2B
flumetasone-2551	3/100	0.049183	0.248146	0.083039	0.418966	-1.11389	3.355287	CCL20;ACSL1;DCN
etiocholanolone-3742	3/100	0.049183	0.248146	0.083039	0.418966	-1.1124	3.350787	IFITM1;MMP3;IFI6
fenbendazole-3888	3/100	0.049183	0.248146	0.083039	0.418966	-1.112	3.349594	AP1S1;PHLDA1;ETS2
homochlorcyclizine-5998	3/100	0.049183	0.248146	0.083039	0.418966	-1.1112	3.347169	CEACAM5;QPCT;PHLDA1
fluorometholone-5771	3/100	0.049183	0.248146	0.083039	0.418966	-1.10973	3.342754	RNF43;MMP7;SPARC
isotretinoin-6017	3/100	0.049183	0.248146	0.083039	0.418966	-1.10933	3.341547	SLPI;TGFBI;PHLDA1
fluvastatin-3370	3/100	0.049183	0.248146	0.083039	0.418966	-1.10575	3.330745	IFITM1;IFI27;IFI6
geldanamycin-1169	3/100	0.049183	0.248146	0.083039	0.418966	-1.10565	3.330453	HSP90AB1;HSPB1;ATP1B1
furaltadone-4313	3/100	0.049183	0.248146	0.083039	0.418966	-1.10469	3.327553	JUND;MMP3;SPP1
homatropine-5477	3/100	0.049183	0.248146	0.083039	0.418966	-1.10353	3.32408	CEACAM6;CEACAM5;MMP3
dinoprost-2446	3/100	0.049183	0.248146	0.083039	0.418966	-1.10198	3.319387	CCL20;ID3;FOS
isoconazole-2218	3/100	0.049183	0.248146	0.083039	0.418966	-1.10189	3.319131	COL1A1;SOX9;FOS
haloperidol-5241	3/100	0.049183	0.248146	0.083039	0.418966	-1.10096	3.316325	IFI27;IFI6;AP1S1
flupentixol-5307	3/100	0.049183	0.248146	0.083039	0.418966	-1.09922	3.311085	APP;S100A4;PHLDA1
hyoscyamine-2108	3/100	0.049183	0.248146	0.083039	0.418966	-1.09748	3.305853	RNF43;JUND;LUM
fulvestrant-5926	3/100	0.049183	0.248146	0.083039	0.418966	-1.09725	3.305156	RNF43;ID1;SECTM1
lobeline-5784	3/100	0.049183	0.248146	0.083039	0.418966	-1.09691	3.304116	CEACAM6;CEACAM5;TMPRSS3

irinotecan-7535	3/100	0.049183	0.248146	0.083039	0.418966	-1.09608	3.301644	JUND;IFI6;FOS
gabexate-2937	3/100	0.049183	0.248146	0.083039	0.418966	-1.09384	3.294888	CCL20;ACSL1;ID1
fosfosal-4823	3/100	0.049183	0.248146	0.083039	0.418966	-1.09274	3.291554	COL1A1;MMP3;IFI6
hyoscyamine-5099	3/100	0.049183	0.248146	0.083039	0.418966	-1.09144	3.287661	SPINK1;QPCT;S100A11
genistein-1660	3/100	0.049183	0.248146	0.083039	0.418966	-1.09082	3.285785	AP1S1;CTSD;PHLDA1
lithocholic acid-3433	3/100	0.049183	0.248146	0.083039	0.418966	-1.09018	3.28386	LGALS3BP;MMP3;DCN
ivermectin-2213	3/100	0.049183	0.248146	0.083039	0.418966	-1.08872	3.279444	COL1A1;TXNIP;PHLDA1
fusaric acid-1308	3/100	0.049183	0.248146	0.083039	0.418966	-1.08793	3.277086	PLCB4;CTSH;PHLDA1
isoconazole-7211	3/100	0.049183	0.248146	0.083039	0.418966	-1.08756	3.275957	COL1A1;FOS;ITM2C
helveticoside-4327	3/100	0.049183	0.248146	0.083039	0.418966	-1.08707	3.274482	TXNIP;FOS;ETS2
hydrocortisone-5284	3/100	0.049183	0.248146	0.083039	0.418966	-1.08659	3.273053	QPCT;IFI6;S100A4
lomefloxacin-4745	3/100	0.049183	0.248146	0.083039	0.418966	-1.08658	3.273024	COL1A1;S100A4;DUOX2
imipenem-7294	3/100	0.049183	0.248146	0.083039	0.418966	-1.08542	3.269519	IFITM1;IFI27;IFI6
loperamide-5632	3/100	0.049183	0.248146	0.083039	0.418966	-1.08441	3.266485	IFI6;FOS;PDZK1IP1
hexestrol-6252	3/100	0.049183	0.248146	0.083039	0.418966	-1.08259	3.260995	SOX9;FOS;PHLDA1
hexetidine-5420	3/100	0.049183	0.248146	0.083039	0.418966	-1.08175	3.258471	S100P;FOS;PHLDA1
lovastatin-6633	3/100	0.049183	0.248146	0.083039	0.418966	-1.08033	3.254185	RNF43;TFF3;CD24
hydroflumethiazide-1809	3/100	0.049183	0.248146	0.083039	0.418966	-1.08006	3.253374	APP;ENC1;AP1S1
lithocholic acid-3899	3/100	0.049183	0.248146	0.083039	0.418966	-1.07992	3.25294	COL1A1;IFI6;CTSE
fulvestrant-5931	3/100	0.049183	0.248146	0.083039	0.418966	-1.07975	3.25245	COL1A2;SERPINA1;IFI6
loxapine-5293	3/100	0.049183	0.248146	0.083039	0.418966	-1.0782	3.247759	BMP4;APP;TXNIP
MG-262-7063	3/100	0.049183	0.248146	0.083039	0.418966	-1.07419	3.235682	ID3;S100P;ETS2
lithocholic acid-2571	3/100	0.049183	0.248146	0.083039	0.418966	-1.0731	3.232409	CCL20;ACSL1;CD24
geldanamycin-6946	3/100	0.049183	0.248146	0.083039	0.418966	-1.07273	3.231284	HSP90AB1;IFI6;SOX9
genistein-1073	3/100	0.049183	0.248146	0.083039	0.418966	-1.06663	3.212915	PHLDA1;S100A11;ITM2B
GW-8510-7062	3/100	0.049183	0.248146	0.083039	0.418966	-1.06565	3.209974	S100A6;TFF3;S100A11
letrozole-7336	3/100	0.049183	0.248146	0.083039	0.418966	-1.065	3.208001	COL1A1;IFI27;SOX9
naproxen-5457	3/100	0.049183	0.248146	0.083039	0.418966	-1.06466	3.206972	C3;SLPI;TFF2
ifosfamide-6279	3/100	0.049183	0.248146	0.083039	0.418966	-1.06411	3.205327	BMP4;APP;CEACAM5
furaltadone-3756	3/100	0.049183	0.248146	0.083039	0.418966	-1.06259	3.20074	IL32;PIGR;CEACAM5
idazoxan-3088	3/100	0.049183	0.248146	0.083039	0.418966	-1.06193	3.198775	NQO1;CEACAM6;AP1S1
mebendazole-7370	3/100	0.049183	0.248146	0.083039	0.418966	-1.06155	3.197628	IL32;RPL28;ETS2
isopropamide iodide-2720	3/100	0.049183	0.248146	0.083039	0.418966	-1.06022	3.193611	APP;GPX2;ID3
mebendazole-2338	3/100	0.049183	0.248146	0.083039	0.418966	-1.05939	3.1911	ANXA2;CCL20;TIMP1
meptazinol-4188	3/100	0.049183	0.248146	0.083039	0.418966	-1.05743	3.185198	FABP1;LUM;PROM1
ketotifen-3200	3/100	0.049183	0.248146	0.083039	0.418966	-1.05504	3.178006	PIGR;SPARC;DCN
kanamycin-5686	3/100	0.049183	0.248146	0.083039	0.418966	-1.05434	3.175907	COL1A1;MMP3;AP1S1

monorden-6938	3/100	0.049183	0.248146	0.083039	0.418966	-1.05148	3.167295	BMP4;PHLDA1;ETS2
mecamylamine-7023	3/100	0.049183	0.248146	0.083039	0.418966	-1.05101	3.165858	TCN1;AP1S1;ETS2
methocarbamol-2111	3/100	0.049183	0.248146	0.083039	0.418966	-1.05025	3.163573	IFI27;IFI6;DCN
metronidazole-4141	3/100	0.049183	0.248146	0.083039	0.418966	-1.0493	3.160708	COL1A1;AP1S1;SECTM1
methylergometrine-1607	3/100	0.049183	0.248146	0.083039	0.418966	-1.04906	3.16	ANXA2;CCL20;TIMP1
perphenazine-1540	3/100	0.049183	0.248146	0.083039	0.418966	-1.04884	3.159331	COL1A1;JUND;SOX9
nalidixic acid-4691	3/100	0.049183	0.248146	0.083039	0.418966	-1.04396	3.144621	SPARC;SLPI;DCN
methapyrilene-6644	3/100	0.049183	0.248146	0.083039	0.418966	-1.04384	3.144278	CD24;DCN;CTGF
idazoxan-5347	3/100	0.049183	0.248146	0.083039	0.418966	-1.04162	3.137588	SERPINA1;IFI27;CEACAM6
monensin-3443	3/100	0.049183	0.248146	0.083039	0.418966	-1.03939	3.130855	SOX9;PHLDA1;S100A11
metergoline-6744	3/100	0.049183	0.248146	0.083039	0.418966	-1.03926	3.130474	C3;CD24;S100A11
methylbenzethonium chloride-3943	3/100	0.049183	0.248146	0.083039	0.418966	-1.03893	3.129471	COL1A1;FOS;PHLDA1
meteneprost-7504	3/100	0.049183	0.248146	0.083039	0.418966	-1.03803	3.126779	APP;TPI1;TFF2
molindone-4199	3/100	0.049183	0.248146	0.083039	0.418966	-1.03785	3.12624	IFITM1;COL1A2;LUM
N-acetylmuramic acid-4406	3/100	0.049183	0.248146	0.083039	0.418966	-1.03676	3.12295	COL1A1;PIGR;DCN
meclofenamic acid-7280	3/100	0.049183	0.248146	0.083039	0.418966	-1.0362	3.121246	MMP3;IFI6;S100A11
lycorine-3808	3/100	0.049183	0.248146	0.083039	0.418966	-1.03608	3.120902	CCL20;TXNIP;ETS2
mepenzolate bromide-4304	3/100	0.049183	0.248146	0.083039	0.418966	-1.03489	3.117315	IL32;TFF3;PHLDA1
niflumic acid-7430	3/100	0.049183	0.248146	0.083039	0.418966	-1.03274	3.110822	COL1A1;BMP4;IFITM1
levonorgestrel-2547	3/100	0.049183	0.248146	0.083039	0.418966	-1.03178	3.107939	IFITM1;GPX2;CCL20
lomefloxacin-3620	3/100	0.049183	0.248146	0.083039	0.418966	-1.03127	3.106412	MMP12;IFI27;IFI6
mianserin-2068	3/100	0.049183	0.248146	0.083039	0.418966	-1.03071	3.104721	CCL20;IFI6;TXNIP
nystatin-4807	3/100	0.049183	0.248146	0.083039	0.418966	-1.03039	3.103743	COL1A1;SPINK1;S100A4
metamizole sodium-3835	3/100	0.049183	0.248146	0.083039	0.418966	-1.02914	3.100006	COL1A1;TCN1;PHLDA1
naproxen-2533	3/100	0.049183	0.248146	0.083039	0.418966	-1.02907	3.099793	APP;MMP3;ATP1B1
isocorydine-4505	3/100	0.049183	0.248146	0.083039	0.418966	-1.02715	3.094006	IFITM1;IFI27;IFI6
pimethixene-7426	3/100	0.049183	0.248146	0.083039	0.418966	-1.0257	3.089644	ID3;FOS;PHLDA1
nocodazole-7145	3/100	0.049183	0.248146	0.083039	0.418966	-1.02196	3.078372	GPX2;ETS2;CTGF
nitrendipine-3087	3/100	0.049183	0.248146	0.083039	0.418966	-1.02098	3.07542	NQO1;IFITM1;TFF2
methylprednisolone-1567	3/100	0.049183	0.248146	0.083039	0.418966	-1.01989	3.072128	CCL20;ACSL1;TCN1
ouabain-6680	3/100	0.049183	0.248146	0.083039	0.418966	-1.01967	3.071462	TXNIP;FOS;ETS2
monorden-325	3/100	0.049183	0.248146	0.083039	0.418966	-1.01921	3.07009	APP;CEACAM5;ITM2B
LY-294002-1239	3/100	0.049183	0.248146	0.083039	0.418966	-1.01913	3.069847	SPINK1;S100A11;ITM2B
monorden-544	3/100	0.049183	0.248146	0.083039	0.418966	-1.01728	3.06427	PIGR;HSP90AB1;HSPB1
minocycline-1135	3/100	0.049183	0.248146	0.083039	0.418966	-1.01623	3.061114	IFITM1;ID3;ISG15
MG-262-7068	3/100	0.049183	0.248146	0.083039	0.418966	-1.01362	3.053248	HSPB1;FOS;CTGF

niclosamide-4136	3/100	0.049183	0.248146	0.083039	0.418966	-1.01288	3.051002	JUND;TGFBI;PHLDA1
nomifensine-2062	3/100	0.049183	0.248146	0.083039	0.418966	-1.01274	3.05059	BMP4;TSPAN8;CCL20
piperacillin-3420	3/100	0.049183	0.248146	0.083039	0.418966	-1.01258	3.050119	MMP3;AP1S1;ITM2C
meticrane-1792	3/100	0.049183	0.248146	0.083039	0.418966	-1.01143	3.046654	COL1A1;AP1S1;TFF2
MG-262-7079	3/100	0.049183	0.248146	0.083039	0.418966	-1.01131	3.046277	ID3;HSPB1;CTGF
monorden-836	3/100	0.049183	0.248146	0.083039	0.418966	-1.00953	3.040928	TFF2;SOX9;DCN
nilutamide-5362	3/100	0.049183	0.248146	0.083039	0.418966	-1.00747	3.034731	IFITM1;CEACAM6;AP1S1
pargyline-2102	3/100	0.049183	0.248146	0.083039	0.418966	-1.00393	3.024066	IFI6;CD24;DUOX2
oxedrine-6156	3/100	0.049183	0.248146	0.083039	0.418966	-1.00364	3.023166	NQO1;ID3;ISG15
pimozide-7132	3/100	0.049183	0.248146	0.083039	0.418966	-1.00285	3.020809	SPARC;FXYD3;TSPAN1
oxyphenbutazone-3582	3/100	0.049183	0.248146	0.083039	0.418966	-1.00273	3.020448	GPX2;DCN;ETS2
nitrofurantoin-4697	3/100	0.049183	0.248146	0.083039	0.418966	-1.00222	3.018903	MMP3;IFI6;TFF2
piperlongumine-1764	3/100	0.049183	0.248146	0.083039	0.418966	-1.0019	3.017946	CKS2;HSPB1;ATP1B1
orciprenaline-2485	3/100	0.049183	0.248146	0.083039	0.418966	-0.99996	3.012085	CCL20;ID3;ATP1B1
monobenzone-5312	3/100	0.049183	0.248146	0.083039	0.418966	-0.99833	3.007172	BMP4;TMPRSS3;S100A4
oxamic acid-439	3/100	0.049183	0.248146	0.083039	0.418966	-0.99799	3.00616	IFITM1;SLPI;CKB
nordihydroguaiaretic acid-5583	3/100	0.049183	0.248146	0.083039	0.418966	-0.99712	3.003541	COL1A1;IFI6;TSPAN1
propofol-3386	3/100	0.049183	0.248146	0.083039	0.418966	-0.99501	2.997183	TXNIP;FOS;ETS2
pioglitazone-5925	3/100	0.049183	0.248146	0.083039	0.418966	-0.99339	2.992291	CEACAM5;SECTM1;SOX9
Prestwick-692-2165	3/100	0.049183	0.248146	0.083039	0.418966	-0.99328	2.991982	CEACAM6;IFI6;PHLDA1
phthalylsulfathiazole-3371	3/100	0.049183	0.248146	0.083039	0.418966	-0.99228	2.988963	TCN1;MMP3;SPP1
MS-275-7074	3/100	0.049183	0.248146	0.083039	0.418966	-0.99224	2.988839	MMP1;ENC1;CTGF
nadolol-3020	3/100	0.049183	0.248146	0.083039	0.418966	-0.99167	2.987123	PIGR;APP;FXYD3
nocodazole-6793	3/100	0.049183	0.248146	0.083039	0.418966	-0.99155	2.986769	PHLDA1;ETS2;CTGF
parbendazole-3799	3/100	0.049183	0.248146	0.083039	0.418966	-0.98868	2.978126	JUND;CCL20;LUM
prochlorperazine-5212	3/100	0.049183	0.248146	0.083039	0.418966	-0.98715	2.973522	SOX9;PHLDA1;ETS2
phenformin-4283	3/100	0.049183	0.248146	0.083039	0.418966	-0.98393	2.963816	JUND;AP1S1;TSPAN1
pizotifen-5072	3/100	0.049183	0.248146	0.083039	0.418966	-0.98347	2.962431	IL32;CEACAM5;CXCL1
pregnenolone-2856	3/100	0.049183	0.248146	0.083039	0.418966	-0.98345	2.962375	JUND;TMPRSS3;PHLDA1
Prestwick-685-4705	3/100	0.049183	0.248146	0.083039	0.418966	-0.98291	2.960742	AP1S1;SOX9;OLFM4
oxybenzone-5410	3/100	0.049183	0.248146	0.083039	0.418966	-0.98284	2.960516	COL1A1;IFITM1;IFI6
perphenazine-5698	3/100	0.049183	0.248146	0.083039	0.418966	-0.9818	2.957403	SOX9;OLFM4;PHLDA1
Prestwick-967-4833	3/100	0.049183	0.248146	0.083039	0.418966	-0.98151	2.956521	BMP4;CEACAM5;MMP3
niclosamide-1998	3/100	0.049183	0.248146	0.083039	0.418966	-0.98007	2.95218	CCL20;PTPRO;SPP1
prednisolone-5526	3/100	0.049183	0.248146	0.083039	0.418966	-0.97847	2.947376	IFITM1;IFI6;ISG15
metitepine-6312	3/100	0.049183	0.248146	0.083039	0.418966	-0.97646	2.94132	FXYD3;CEACAM6;IFI6
puromycin-2448	3/100	0.049183	0.248146	0.083039	0.418966	-0.97502	2.936963	TXNIP;HSPB1;ISG15

oxaprozin-863	3/100	0.049183	0.248146	0.083039	0.418966	-0.97478	2.93624	LGALS3BP;IFITM1;TXNIP
myosmine-3634	3/100	0.049183	0.248146	0.083039	0.418966	-0.97474	2.936134	COL1A2;IFI6;DCN
myricetin-3270	3/100	0.049183	0.248146	0.083039	0.418966	-0.97388	2.933548	IFITM1;IFI27;IFI6
Prestwick-1082-7027	3/100	0.049183	0.248146	0.083039	0.418966	-0.97211	2.928196	APP;S100A6;SOX9
Prestwick-664-3613	3/100	0.049183	0.248146	0.083039	0.418966	-0.97136	2.925943	IFITM1;IFI27;IFI6
promazine-2173	3/100	0.049183	0.248146	0.083039	0.418966	-0.96782	2.915279	COL1A1;NQO1;MMP3
prenylamine-5489	3/100	0.049183	0.248146	0.083039	0.418966	-0.96584	2.909316	FOS;PHLDA1;IER2
perphenazine-1956	3/100	0.049183	0.248146	0.083039	0.418966	-0.96539	2.907957	JUND;MMP1;ETS2
pilocarpine-5341	3/100	0.049183	0.248146	0.083039	0.418966	-0.96335	2.901811	BMP4;SERPINA1;S100A4
roxarsone-5051	3/100	0.049183	0.248146	0.083039	0.418966	-0.96109	2.895016	IL32;SPP1;PSMB9
myosmine-2199	3/100	0.049183	0.248146	0.083039	0.418966	-0.95948	2.890174	COL1A2;TCN1;IGFBP2
oxamniquine-4124	3/100	0.049183	0.248146	0.083039	0.418966	-0.95897	2.888632	SPINK1;IFI27;IFI6
quinidine-6267	3/100	0.049183	0.248146	0.083039	0.418966	-0.95827	2.886522	BMP4;APP;TSPAN1
primaquine-4845	3/100	0.049183	0.248146	0.083039	0.418966	-0.95782	2.88517	SPINK1;FOS;CTGF
resveratrol-662	3/100	0.049183	0.248146	0.083039	0.418966	-0.95636	2.880775	CD24;ITM2B;CTGF
procarbazine-3533	3/100	0.049183	0.248146	0.083039	0.418966	-0.95412	2.874009	C3;IFI27;DCN
Prestwick-864-4113	3/100	0.049183	0.248146	0.083039	0.418966	-0.95067	2.863622	SPARC;DCN;ITM2C
Prestwick-674-3716	3/100	0.049183	0.248146	0.083039	0.418966	-0.94988	2.861242	IL32;APP;MMP3
prenylamine-1737	3/100	0.049183	0.248146	0.083039	0.418966	-0.94907	2.858793	IFITM1;IFI6;ISG15
pyrazinamide-2839	3/100	0.049183	0.248146	0.083039	0.418966	-0.94789	2.855255	COL1A1;PIGR;CEACAM5
ribostamycin-3444	3/100	0.049183	0.248146	0.083039	0.418966	-0.94774	2.854802	COL1A1;SOX9;LYZ
tamoxifen-2212	3/100	0.049183	0.248146	0.083039	0.418966	-0.94694	2.852399	IL32;FOS;IER2
PHA-00745360-4381	3/100	0.049183	0.248146	0.083039	0.418966	-0.9463	2.850449	IFITM1;IFI27;IFI6
scriptaid-6896	3/100	0.049183	0.248146	0.083039	0.418966	-0.94518	2.847093	ACSL1;MMP1;CTGF
Prestwick-675-2187	3/100	0.049183	0.248146	0.083039	0.418966	-0.94477	2.845862	MMP7;IGFBP2;TFF2
probucol-5626	3/100	0.049183	0.248146	0.083039	0.418966	-0.94401	2.843554	MMP3;IFI6;PHLDA1
piperidolate-6772	3/100	0.049183	0.248146	0.083039	0.418966	-0.94348	2.841954	IFITM1;TCN1;ITM2C
rosiglitazone-5230	3/100	0.049183	0.248146	0.083039	0.418966	-0.94074	2.833721	IFI6;TSPAN1;ITM2C
pyrvinium-978	3/100	0.049183	0.248146	0.083039	0.418966	-0.93827	2.826272	JUND;PHLDA1;ETS2
salsolidin-2463	3/100	0.049183	0.248146	0.083039	0.418966	-0.93822	2.826109	COL1A1;GPX2;CTSE
quinethazone-3793	3/100	0.049183	0.248146	0.083039	0.418966	-0.93748	2.823879	IFITM1;LUM;DCN
racecadotril-6231	3/100	0.049183	0.248146	0.083039	0.418966	-0.93578	2.818776	IFITM1;IFI27;IFI6
sirolimus-1148	3/100	0.049183	0.248146	0.083039	0.418966	-0.9355	2.817914	CCL20;PHLDA1;LY6E
rosiglitazone-1658	3/100	0.049183	0.248146	0.083039	0.418966	-0.93063	2.803263	RNF43;SERPINA1;CTSD
tanespimycin-1159	3/100	0.049183	0.248146	0.083039	0.418966	-0.92875	2.797591	HSP90AB1;HSPB1;ATP1B1
terconazole-4407	3/100	0.049183	0.248146	0.083039	0.418966	-0.92628	2.790154	SPARC;SECTM1;DCN
tanespimycin-1147	3/100	0.049183	0.248146	0.083039	0.418966	-0.92515	2.786744	APP;HSP90AB1;HSPB1

tetrahydroalstonine-2748	3/100	0.049183	0.248146	0.083039	0.418966	-0.92389	2.782949	COL1A1;SLPI;IFI6
pirenzepine-2071	3/100	0.049183	0.248146	0.083039	0.418966	-0.92313	2.780665	SERPINA1;TSPAN8;CCL20
strophanthidin-2525	3/100	0.049183	0.248146	0.083039	0.418966	-0.92306	2.78046	CCL20;TXNIP;FOS
scriptaid-6919	3/100	0.049183	0.248146	0.083039	0.418966	-0.92291	2.780016	MMP1;S100P;CTGF
pyrvinium-2957	3/100	0.049183	0.248146	0.083039	0.418966	-0.92271	2.779414	CCL20;ID3;FOS
sulfadimidine-3847	3/100	0.049183	0.248146	0.083039	0.418966	-0.92057	2.77296	IFITM1;IFI27;IFI6
sodium phenylbutyrate-502	3/100	0.049183	0.248146	0.083039	0.418966	-0.92025	2.77199	IFITM1;GPX2;PDZK1IP1
sirolimus-4466	3/100	0.049183	0.248146	0.083039	0.418966	-0.92003	2.771323	MMP7;TMPRSS3;ID3
staurosporine-423	3/100	0.049183	0.248146	0.083039	0.418966	-0.91753	2.763811	COL1A1;ISG15;ETS2
spironolactone-6255	3/100	0.049183	0.248146	0.083039	0.418966	-0.91699	2.762184	IFI6;ISG15;DCN
pramocaine-3894	3/100	0.049183	0.248146	0.083039	0.418966	-0.91585	2.758736	IFITM1;IFI27;IFI6
salbutamol-7376	3/100	0.049183	0.248146	0.083039	0.418966	-0.91442	2.75442	TCN1;S100P;CD24
ritodrine-2635	3/100	0.049183	0.248146	0.083039	0.418966	-0.91427	2.753971	C3;SPINK1;CEACAM5
sulfadimidine-4322	3/100	0.049183	0.248146	0.083039	0.418966	-0.91229	2.748004	C3;JUND;SERPINA1
Prestwick-642-2160	3/100	0.049183	0.248146	0.083039	0.418966	-0.912	2.747154	NQO1;APP;LCN2
sulfapyridine-7151	3/100	0.049183	0.248146	0.083039	0.418966	-0.91083	2.743618	APP;IFITM1;IFI6
prenylamine-5070	3/100	0.049183	0.248146	0.083039	0.418966	-0.91063	2.743003	JUND;TFF3;FOS
rosiglitazone-2693	3/100	0.049183	0.248146	0.083039	0.418966	-0.90974	2.740331	ENC1;PHLDA1;DCN
quinisocaine-4791	3/100	0.049183	0.248146	0.083039	0.418966	-0.90857	2.736816	CEACAM5;FOS;PHLDA1
theobromine-2995	3/100	0.049183	0.248146	0.083039	0.418966	-0.90844	2.736424	PIGR;MMP3;DCN
ritodrine-1280	3/100	0.049183	0.248146	0.083039	0.418966	-0.90665	2.731022	AP1S1;ITGAV;ITM2B
tanespimycin-1166	3/100	0.049183	0.248146	0.083039	0.418966	-0.90477	2.725358	APP;HSP90AB1;HSPB1
tanespimycin-1226	3/100	0.049183	0.248146	0.083039	0.418966	-0.90313	2.720432	SERPINA1;HSPB1;CTGF
tanespimycin-1218	3/100	0.049183	0.248146	0.083039	0.418966	-0.90147	2.715431	APP;HSPB1;CTGF
talampicillin-2954	3/100	0.049183	0.248146	0.083039	0.418966	-0.90084	2.713531	NQO1;IGFBP2;DSG2
sulfametoxydiazine-2712	3/100	0.049183	0.248146	0.083039	0.418966	-0.90045	2.712359	CEACAM5;CD24;DCN
tretinoin-3165	3/100	0.049183	0.248146	0.083039	0.418966	-0.90015	2.711436	ID1;SOX9;TGFBI
sulfadiazine-5523	3/100	0.049183	0.248146	0.083039	0.418966	-0.90011	2.711315	IFITM1;IFI6;DCN
sirolimus-6958	3/100	0.049183	0.248146	0.083039	0.418966	-0.89857	2.706691	CEACAM6;TXNIP;SECTM1
sulpiride-4389	3/100	0.049183	0.248146	0.083039	0.418966	-0.89709	2.702239	TCN1;MMP3;SPP1
sirolimus-987	3/100	0.049183	0.248146	0.083039	0.418966	-0.89652	2.700526	COL1A1;MMP1;TXNIP
trichostatin A-5572	3/100	0.049183	0.248146	0.083039	0.418966	-0.89605	2.699108	IFI6;TXNIP;ITM2C
tetryzoline-2507	3/100	0.049183	0.248146	0.083039	0.418966	-0.89096	2.683759	SLCO1B3;TIMP1;DCN
thiamphenicol-1826	3/100	0.049183	0.248146	0.083039	0.418966	-0.88987	2.68048	BMP4;PTPRO;AP1S1
thiamphenicol-1704	3/100	0.049183	0.248146	0.083039	0.418966	-0.88857	2.676577	BMP4;C3;GPX2
tetrandrine-7178	3/100	0.049183	0.248146	0.083039	0.418966	-0.88565	2.667772	JUND;ACSL1;IFI6
stachydrine-2743	3/100	0.049183	0.248146	0.083039	0.418966	-0.88477	2.665113	APP;COL1A2;SLPI

sirolimus-5927	3/100	0.049183	0.248146	0.083039	0.418966	-0.88438	2.663932	COL1A2;SECTM1;SOX9
thapsigargin-7103	3/100	0.049183	0.248146	0.083039	0.418966	-0.88426	2.66359	TXNIP;PHLDA1;ETS2
tretinoin-384	3/100	0.049183	0.248146	0.083039	0.418966	-0.88343	2.66109	IFITM1;SOX9;PHLDA1
trichostatin A-4184	3/100	0.049183	0.248146	0.083039	0.418966	-0.8832	2.660378	MMP1;MMP3;CTGF
terguride-3096	3/100	0.049183	0.248146	0.083039	0.418966	-0.88312	2.66016	NQO1;IFITM1;FXYD3
tribenoside-2946	3/100	0.049183	0.248146	0.083039	0.418966	-0.88306	2.659956	CCL20;ISG15;ATP1B1
trichostatin A-3872	3/100	0.049183	0.248146	0.083039	0.418966	-0.88124	2.654485	IFITM1;IFI27;IFI6
theobromine-4958	3/100	0.049183	0.248146	0.083039	0.418966	-0.88066	2.65274	SPINK1;MMP3;TFF2
rescinnamine-2130	3/100	0.049183	0.248146	0.083039	0.418966	-0.88013	2.651137	CCL20;CEACAM5;PHLDA1
ticlopidine-4074	3/100	0.049183	0.248146	0.083039	0.418966	-0.87996	2.650638	C3;PIGR;CEACAM5
STOCK1N-35874-6583	3/100	0.049183	0.248146	0.083039	0.418966	-0.87945	2.649089	HSP90AB1;AP1S1;DCN
trichostatin A-2084	3/100	0.049183	0.248146	0.083039	0.418966	-0.87926	2.648505	CCL20;MMP1;CTGF
tretinoin-5571	3/100	0.049183	0.248146	0.083039	0.418966	-0.8784	2.645936	SOX9;TGFBI;PHLDA1
trichostatin A-1234	3/100	0.049183	0.248146	0.083039	0.418966	-0.87828	2.645566	CCL20;MMP1;CTGF
trichostatin A-4665	3/100	0.049183	0.248146	0.083039	0.418966	-0.87804	2.64485	BMP4;CTGF;PSMB9
thioridazine-5590	3/100	0.049183	0.248146	0.083039	0.418966	-0.87804	2.644832	COL1A1;FOS;PHLDA1
trifluoperazine-5584	3/100	0.049183	0.248146	0.083039	0.418966	-0.87757	2.643428	SOX9;FOS;PHLDA1
thioridazine-4454	3/100	0.049183	0.248146	0.083039	0.418966	-0.87613	2.639103	JUND;S100P;FOS
thioperamide-3392	3/100	0.049183	0.248146	0.083039	0.418966	-0.87543	2.636996	CEACAM5;TXNIP;PHLDA1
terconazole-2484	3/100	0.049183	0.248146	0.083039	0.418966	-0.87534	2.636725	IFITM1;MMP7;AP1S1
trichlormethiazide-2998	3/100	0.049183	0.248146	0.083039	0.418966	-0.87437	2.633798	GPX2;COL1A2;DCN
trichostatin A-2993	3/100	0.049183	0.248146	0.083039	0.418966	-0.87378	2.631999	TSPAN13;ID3;ATP1B1
trichostatin A-6439	3/100	0.049183	0.248146	0.083039	0.418966	-0.87348	2.631101	APP;MMP1;CTGF
trichostatin A-3462	3/100	0.049183	0.248146	0.083039	0.418966	-0.87288	2.629302	TXNIP;SOX9;PSMB9
trichostatin A-6085	3/100	0.049183	0.248146	0.083039	0.418966	-0.87219	2.627215	IFI6;TXNIP;ITM2C
xylazine-4066	3/100	0.049183	0.248146	0.083039	0.418966	-0.87127	2.62446	BMP4;APP;TSPAN8
tanespimycin-5585	3/100	0.049183	0.248146	0.083039	0.418966	-0.86921	2.618256	COL1A1;PIGR;IFI6
trichostatin A-3868	3/100	0.049183	0.248146	0.083039	0.418966	-0.86912	2.617983	TXNIP;ITM2C;PSMB9
trichostatin A-2694	3/100	0.049183	0.248146	0.083039	0.418966	-0.8687	2.616719	TSPAN13;IFI6;ATP1B1
troleandomycin-1885	3/100	0.049183	0.248146	0.083039	0.418966	-0.86684	2.611115	NPM1;MMP7;DCN
troglitazone-1070	3/100	0.049183	0.248146	0.083039	0.418966	-0.86506	2.60574	SOX9;PHLDA1;IER2
trichostatin A-1561	3/100	0.049183	0.248146	0.083039	0.418966	-0.86494	2.605371	TSPAN13;CCL20;QPCT
STOCK1N-35215-6427	3/100	0.049183	0.248146	0.083039	0.418966	-0.86396	2.602446	CXCL1;PROM1;ETS2
trichostatin A-1400	3/100	0.049183	0.248146	0.083039	0.418966	-0.86272	2.598688	TSPAN13;CKB;ATP1B1
trichostatin A-4565	3/100	0.049183	0.248146	0.083039	0.418966	-0.86235	2.597594	BMP4;CTGF;PSMB9
trichostatin A-2523	3/100	0.049183	0.248146	0.083039	0.418966	-0.86099	2.593475	TSPAN13;ID3;ATP1B1
trichostatin A-2035	3/100	0.049183	0.248146	0.083039	0.418966	-0.86093	2.593302	TSPAN13;ATP1B1;ITM2C

tiaprofenic acid-2492	3/100	0.049183	0.248146	0.083039	0.418966	-0.86053	2.592096	GPX2;CEACAM6;MMP3
trichostatin A-1951	3/100	0.049183	0.248146	0.083039	0.418966	-0.85999	2.590474	CCL20;MMP1;CTGF
trichostatin A-4458	3/100	0.049183	0.248146	0.083039	0.418966	-0.85866	2.586475	MMP1;FOS;CTGF
trichostatin A-2474	3/100	0.049183	0.248146	0.083039	0.418966	-0.85852	2.586057	TSPAN13;ID3;ATP1B1
trichostatin A-3243	3/100	0.049183	0.248146	0.083039	0.418966	-0.85602	2.578528	IFI6;TXNIP;ITM2C
trichostatin A-6546	3/100	0.049183	0.248146	0.083039	0.418966	-0.85507	2.575669	MMP1;TXNIP;CTGF
valproic acid-5237	3/100	0.049183	0.248146	0.083039	0.418966	-0.85452	2.574003	COL1A1;APP;ITM2C
verteporfin-6817	3/100	0.049183	0.248146	0.083039	0.418966	-0.85338	2.570572	RNF43;IFITM1;ISG15
trichostatin A-7047	3/100	0.049183	0.248146	0.083039	0.418966	-0.84986	2.559972	MMP1;FOS;CTGF
trifluoperazine-1004	3/100	0.049183	0.248146	0.083039	0.418966	-0.84798	2.554287	RCN1;FOS;PHLDA1
trichostatin A-873	3/100	0.049183	0.248146	0.083039	0.418966	-0.84759	2.553134	TXNIP;FOS;ITM2C
trichostatin A-6874	3/100	0.049183	0.248146	0.083039	0.418966	-0.8475	2.552844	TXNIP;CTGF;PSMB9
viomycin-7278	3/100	0.049183	0.248146	0.083039	0.418966	-0.84551	2.546855	IFI6;AP1S1;ITM2B
wortmannin-869	3/100	0.049183	0.248146	0.083039	0.418966	-0.84481	2.544738	TXNIP;FOS;TGFBI
zaprinast-5349	3/100	0.049183	0.248146	0.083039	0.418966	-0.84346	2.540687	SERPINA1;CEACAM6;ETS2
tretinoin-1152	3/100	0.049183	0.248146	0.083039	0.418966	-0.84234	2.537323	CCL20;ID1;TIMP1
vidarabine-7203	3/100	0.049183	0.248146	0.083039	0.418966	-0.83867	2.526254	CEACAM5;MMP3;IFI6
trichostatin A-6709	3/100	0.049183	0.248146	0.083039	0.418966	-0.838	2.524242	MMP1;CTGF;PSMB9
trichostatin A-4237	3/100	0.049183	0.248146	0.083039	0.418966	-0.8376	2.523024	MMP1;CTGF;PSMB9
vorinostat-4444	3/100	0.049183	0.248146	0.083039	0.418966	-0.83665	2.520181	MMP1;FOS;CTGF
trimethadione-4086	3/100	0.049183	0.248146	0.083039	0.418966	-0.83628	2.519045	RNF43;SERPINA1;TMPRSS3
trichostatin A-5625	3/100	0.049183	0.248146	0.083039	0.418966	-0.83572	2.517373	TXNIP;ITM2C;PSMB9
trichostatin A-5940	3/100	0.049183	0.248146	0.083039	0.418966	-0.83547	2.516617	BMP4;MMP1;CTGF
vorinostat-5580	3/100	0.049183	0.248146	0.083039	0.418966	-0.83113	2.503534	IFI6;TXNIP;ISG15
vancomycin-2498	3/100	0.049183	0.248146	0.083039	0.418966	-0.82958	2.498888	GPX2;COL1A2;MMP3
trichostatin A-6951	3/100	0.049183	0.248146	0.083039	0.418966	-0.82532	2.48604	TXNIP;ISG15;SOX9
trichostatin A-6879	3/100	0.049183	0.248146	0.083039	0.418966	-0.82481	2.484501	IFI6;SOX9;ITM2C
trimipramine-4163	3/100	0.049183	0.248146	0.083039	0.418966	-0.82359	2.480825	FOS;PHLDA1;IER2
trimethoprim-3678	3/100	0.049183	0.248146	0.083039	0.418966	-0.82261	2.477864	IL32;SPARC;SLCO5A1
wortmannin-977	3/100	0.049183	0.248146	0.083039	0.418966	-0.82057	2.471742	TXNIP;S100P;FOS
valproic acid-5582	3/100	0.049183	0.248146	0.083039	0.418966	-0.81809	2.464266	COL1A1;TCN1;IFI6
triflusal-7451	3/100	0.049183	0.248146	0.083039	0.418966	-0.81767	2.463005	APP;IFITM1;PHLDA1
trifluoperazine-6984	3/100	0.049183	0.248146	0.083039	0.418966	-0.8171	2.461287	MMP3;FOS;PHLDA1
trichostatin A-6886	3/100	0.049183	0.248146	0.083039	0.418966	-0.81694	2.46079	TXNIP;AP1S1;CTGF
vinblastine-7556	3/100	0.049183	0.248146	0.083039	0.418966	-0.81658	2.459703	TPI1;IFI6;CTGF
zomepirac-4479	3/100	0.049183	0.248146	0.083039	0.418966	-0.81327	2.44974	JUND;QPCT;CTSE
trichostatin A-3058	3/100	0.049183	0.248146	0.083039	0.418966	-0.813	2.44892	TSPAN13;ID3;ATP1B1

valproic acid-1150	3/100	0.049183	0.248146	0.083039	0.418966	-0.81134	2.443939	TSPAN13;QPCT;ATP1B1
vorinostat-2680	3/100	0.049183	0.248146	0.083039	0.418966	-0.80795	2.433707	TSPAN13;IFI6;ATP1B1
valproic acid-1163	3/100	0.049183	0.248146	0.083039	0.418966	-0.80782	2.433315	APP;CCL20;CEACAM6
wortmannin-6202	3/100	0.049183	0.248146	0.083039	0.418966	-0.79399	2.391659	IFITM1;GPX2;ISG15
zuclopenthixol-4843	3/100	0.049183	0.248146	0.083039	0.418966	-0.78408	2.361806	APP;IFI6;PHLDA1
withaferin A-3902	3/100	0.049183	0.248146	0.083039	0.418966	-0.77539	2.335643	PHLDA1;ETS2;CTGF
xamoterol-5363	3/100	0.049183	0.248146	0.083039	0.418966	-0.76705	2.310529	IFITM1;IFI27;IFI6
(-)-atenolol-6444	2/100	0.197972	0.408923	0.269513	0.556697	-0.83689	1.35546	FABP1;PIGR
(+)-isoprenaline-3046	2/100	0.197972	0.408923	0.269513	0.556697	-0.82117	1.329992	ID3;TIMP1
(-)-atenolol-5325	2/100	0.197972	0.408923	0.269513	0.556697	-0.8206	1.329069	BMP4;CEACAM6
1,4-chrysenequinone-1773	2/100	0.197972	0.408923	0.269513	0.556697	-0.81783	1.324577	NQO1;HSPB1
5149715-890	2/100	0.197972	0.408923	0.269513	0.556697	-0.81195	1.315056	RNF43;TPI1
11-deoxy-16,16-dimethylprostaglandin E2-7533	2/100	0.197972	0.408923	0.269513	0.556697	-0.80903	1.310327	TMPRSS3;PHLDA1
5182598-976	2/100	0.197972	0.408923	0.269513	0.556697	-0.80617	1.305702	GPX2;ETS2
0173570-0000-4712	2/100	0.197972	0.408923	0.269513	0.556697	-0.80076	1.296929	PHLDA1;ETS2
(+)-chelidonine-5760	2/100	0.197972	0.408923	0.269513	0.556697	-0.79956	1.294994	APP;JUND
alpha-ergocryptine-3817	2/100	0.197972	0.408923	0.269513	0.556697	-0.7978	1.292138	JUND;CCL20
15-delta prostaglandin J2-1656	2/100	0.197972	0.408923	0.269513	0.556697	-0.79624	1.289619	AP1S1;ETS2
5279552-843	2/100	0.197972	0.408923	0.269513	0.556697	-0.79564	1.288639	RNF43;TMPRSS3
15-delta prostaglandin J2-5228	2/100	0.197972	0.408923	0.269513	0.556697	-0.79544	1.288324	GPX2;ETS2
2,6-dimethylpiperidine-4363	2/100	0.197972	0.408923	0.269513	0.556697	-0.79512	1.287798	IFI27;IFI6
0198306-0000-7069	2/100	0.197972	0.408923	0.269513	0.556697	-0.79422	1.286347	APP;ITM2B
16,16-dimethylprostaglandin E2-6597	2/100	0.197972	0.408923	0.269513	0.556697	-0.7936	1.285344	APP;SOX9
0317956-0000-3777	2/100	0.197972	0.408923	0.269513	0.556697	-0.79232	1.28326	IL32;IFITM1
4-hydroxyphenazone-4095	2/100	0.197972	0.408923	0.269513	0.556697	-0.79179	1.282413	PIGR;CEACAM5
0316684-0000-7052	2/100	0.197972	0.408923	0.269513	0.556697	-0.78707	1.274761	COL1A1;APP
15-delta prostaglandin J2-6190	2/100	0.197972	0.408923	0.269513	0.556697	-0.78649	1.273823	NQO1;CTSE
16-phenyltetranorprostaglandin E2-7509	2/100	0.197972	0.408923	0.269513	0.556697	-0.78576	1.272645	S100P;CD24
0225151-0000-6384	2/100	0.197972	0.408923	0.269513	0.556697	-0.7835	1.268977	APP;PTPRO
0173570-0000-3693	2/100	0.197972	0.408923	0.269513	0.556697	-0.78332	1.268695	IL32;COL1A2
5140203-908	2/100	0.197972	0.408923	0.269513	0.556697	-0.78289	1.267993	APP;TMPRSS3
adiphenine-1872	2/100	0.197972	0.408923	0.269513	0.556697	-0.78192	1.266427	TSPAN13;ANXA3
AG-013608-6400	2/100	0.197972	0.408923	0.269513	0.556697	-0.78166	1.266002	JUND;TGFBI
AG-012559-6889	2/100	0.197972	0.408923	0.269513	0.556697	-0.77982	1.263024	IFI27;AP1S1
0317956-0000-3858	2/100	0.197972	0.408923	0.269513	0.556697	-0.77905	1.261767	COL1A1;TXNIP

3-nitropropionic acid-6402	2/100	0.197972	0.408923	0.269513	0.556697	-0.77754	1.259327	MMP3;IFI6
12,13-EODE-1108	2/100	0.197972	0.408923	0.269513	0.556697	-0.77625	1.257235	TGFBI;ITM2B
abamectin-5864	2/100	0.197972	0.408923	0.269513	0.556697	-0.77562	1.256212	COL1A1;CEACAM5
AG-013608-5944	2/100	0.197972	0.408923	0.269513	0.556697	-0.77513	1.255417	CEACAM5;ITM2C
5707885-6438	2/100	0.197972	0.408923	0.269513	0.556697	-0.77361	1.252963	APP;ETS2
5253409-961	2/100	0.197972	0.408923	0.269513	0.556697	-0.77316	1.252234	FOS;PHLDA1
15(S)-15-methylprostaglandin E2-7494	2/100	0.197972	0.408923	0.269513	0.556697	-0.77225	1.250762	JUND;CEACAM6
5109870-904	2/100	0.197972	0.408923	0.269513	0.556697	-0.77199	1.250342	FOS;PGM1
5182598-868	2/100	0.197972	0.408923	0.269513	0.556697	-0.77174	1.249929	CEACAM5;ETS2
acetylsalicylic acid-2664	2/100	0.197972	0.408923	0.269513	0.556697	-0.77096	1.248678	NQO1;MMP3
5224221-839	2/100	0.197972	0.408923	0.269513	0.556697	-0.77086	1.248512	ID3;ETS2
10-methoxyharmalan-1743	2/100	0.197972	0.408923	0.269513	0.556697	-0.76958	1.246443	IFITM3;APP
(-)-catechin-1101	2/100	0.197972	0.408923	0.269513	0.556697	-0.76927	1.245937	APP;JUND
3-acetamidocoumarin-2941	2/100	0.197972	0.408923	0.269513	0.556697	-0.76867	1.244969	IGFBP2;PHLDA1
AH-23848-6903	2/100	0.197972	0.408923	0.269513	0.556697	-0.76672	1.241802	APP;PTMA
aceclofenac-2117	2/100	0.197972	0.408923	0.269513	0.556697	-0.76667	1.241725	BMP4;APP
alfuzosin-1586	2/100	0.197972	0.408923	0.269513	0.556697	-0.76164	1.233582	SPP1;ITM2B
0316684-0000-7098	2/100	0.197972	0.408923	0.269513	0.556697	-0.75886	1.229068	PHLDA1;ITM2B
aciclovir-5643	2/100	0.197972	0.408923	0.269513	0.556697	-0.7587	1.228809	IFI6;TXNIP
5279552-960	2/100	0.197972	0.408923	0.269513	0.556697	-0.75708	1.226185	JUND;S100A11
alimemazine-7235	2/100	0.197972	0.408923	0.269513	0.556697	-0.75529	1.223297	RNF43;IFI6
acacetin-3849	2/100	0.197972	0.408923	0.269513	0.556697	-0.75251	1.218785	PIGR;IFITM1
acenocoumarol-2240	2/100	0.197972	0.408923	0.269513	0.556697	-0.75209	1.218113	COL1A1;DUOX2
5155877-6569	2/100	0.197972	0.408923	0.269513	0.556697	-0.74831	1.21198	APP;IFNGR1
adipiodone-5510	2/100	0.197972	0.408923	0.269513	0.556697	-0.74803	1.211537	PIGR;IFI6
acetylsalicylic acid-6924	2/100	0.197972	0.408923	0.269513	0.556697	-0.74642	1.208932	SECTM1;ETS2
acetylsalicylic acid-5201	2/100	0.197972	0.408923	0.269513	0.556697	-0.7457	1.207754	IFI27;IFI6
alvespimycin-6973	2/100	0.197972	0.408923	0.269513	0.556697	-0.74568	1.207719	BMP4;IFI6
aciclovir-1960	2/100	0.197972	0.408923	0.269513	0.556697	-0.74432	1.205524	TCN1;CD24
alpha-estradiol-5207	2/100	0.197972	0.408923	0.269513	0.556697	-0.74277	1.20302	COL1A1;TSPAN1
apomorphine-5283	2/100	0.197972	0.408923	0.269513	0.556697	-0.74198	1.201738	IFITM1;IFI6
alsterpaullone-7051	2/100	0.197972	0.408923	0.269513	0.556697	-0.74039	1.19916	APP;IFITM1
acemetacin-6361	2/100	0.197972	0.408923	0.269513	0.556697	-0.73904	1.196976	IFITM1;GPX2
abamectin-6081	2/100	0.197972	0.408923	0.269513	0.556697	-0.73878	1.196549	C3;TXNIP
azacyclonol-5398	2/100	0.197972	0.408923	0.269513	0.556697	-0.73772	1.194835	COL1A1;ITM2C
alprenolol-1571	2/100	0.197972	0.408923	0.269513	0.556697	-0.73511	1.190611	IFITM1;CCL20

articaine-7272	2/100	0.197972	0.408923	0.269513	0.556697	-0.73493	1.190313	SERPINA1;CEACAM5
acetylsalicylic acid-1629	2/100	0.197972	0.408923	0.269513	0.556697	-0.73471	1.189957	SECTM1;ETS2
alprostadil-6576	2/100	0.197972	0.408923	0.269513	0.556697	-0.73459	1.189769	S100A11;ACTB
acetazolamide-1686	2/100	0.197972	0.408923	0.269513	0.556697	-0.7345	1.189617	BMP4;PHLDA1
AH-23848-6885	2/100	0.197972	0.408923	0.269513	0.556697	-0.73391	1.188661	CEACAM6;AP1S1
aciclovir-2044	2/100	0.197972	0.408923	0.269513	0.556697	-0.7327	1.186707	CEACAM5;ITM2B
alpha-estradiol-6930	2/100	0.197972	0.408923	0.269513	0.556697	-0.7325	1.186384	SOX9;PHLDA1
atracurium besilate-1702	2/100	0.197972	0.408923	0.269513	0.556697	-0.73163	1.184974	BMP4;C3
acacetin-3942	2/100	0.197972	0.408923	0.269513	0.556697	-0.73079	1.183611	IFI27;IFI6
albendazole-7164	2/100	0.197972	0.408923	0.269513	0.556697	-0.72791	1.178949	RCN1;CEACAM5
alvespimycin-5573	2/100	0.197972	0.408923	0.269513	0.556697	-0.7265	1.176661	COL1A1;SOX9
alpha-ergocryptine-2572	2/100	0.197972	0.408923	0.269513	0.556697	-0.72415	1.172849	GPX2;CCL20
apomorphine-6683	2/100	0.197972	0.408923	0.269513	0.556697	-0.7227	1.170505	PIGR;COL1A2
amoxicillin-6285	2/100	0.197972	0.408923	0.269513	0.556697	-0.72236	1.169954	CDH3;CEACAM5
aminophenazone-2060	2/100	0.197972	0.408923	0.269513	0.556697	-0.7218	1.169046	JUND;CCL20
alvespimycin-2673	2/100	0.197972	0.408923	0.269513	0.556697	-0.71958	1.165449	HSP90AB1;HSPB1
atractyloside-7393	2/100	0.197972	0.408923	0.269513	0.556697	-0.71906	1.164617	AP1S1;DCN
alexidine-4721	2/100	0.197972	0.408923	0.269513	0.556697	-0.7183	1.163376	SOX9;PHLDA1
alpha-yohimbine-2778	2/100	0.197972	0.408923	0.269513	0.556697	-0.71715	1.161515	APP;CEACAM5
alprostadil-2938	2/100	0.197972	0.408923	0.269513	0.556697	-0.71426	1.156845	ID3;TIMP1
apomorphine-1923	2/100	0.197972	0.408923	0.269513	0.556697	-0.71371	1.15595	TPI1;CEACAM6
altretamine-3090	2/100	0.197972	0.408923	0.269513	0.556697	-0.7132	1.155125	IFITM1;CEACAM6
adiphenine-1709	2/100	0.197972	0.408923	0.269513	0.556697	-0.71295	1.154709	BMP4;S100A11
artemisinin-7007	2/100	0.197972	0.408923	0.269513	0.556697	-0.71003	1.149986	SLCO1B3;TFF2
antazoline-7128	2/100	0.197972	0.408923	0.269513	0.556697	-0.70957	1.149248	APP;CEACAM5
altretamine-4627	2/100	0.197972	0.408923	0.269513	0.556697	-0.7095	1.149134	MMP7;DCN
aminoglutethimide-2390	2/100	0.197972	0.408923	0.269513	0.556697	-0.70695	1.145	TCN1;PHLDA1
alvespimycin-6172	2/100	0.197972	0.408923	0.269513	0.556697	-0.70558	1.142778	HSPB1;TGFBI
aztreonam-5535	2/100	0.197972	0.408923	0.269513	0.556697	-0.70466	1.141296	COL1A1;IFITM1
amoxapine-4996	2/100	0.197972	0.408923	0.269513	0.556697	-0.70431	1.140715	RNF43;AP1S1
androsterone-1296	2/100	0.197972	0.408923	0.269513	0.556697	-0.70309	1.138746	AP1S1;ITM2B
azathioprine-5627	2/100	0.197972	0.408923	0.269513	0.556697	-0.70175	1.136583	TCN1;CEACAM5
alclometasone-2532	2/100	0.197972	0.408923	0.269513	0.556697	-0.70114	1.135588	CCL20;ACSL1
azacitidine-3348	2/100	0.197972	0.408923	0.269513	0.556697	-0.69897	1.132075	SPINK1;TXNIP
buspirone-5343	2/100	0.197972	0.408923	0.269513	0.556697	-0.69819	1.130809	SERPINA1;TFF2
azathioprine-5262	2/100	0.197972	0.408923	0.269513	0.556697	-0.69649	1.128062	C3;DCN
aminohippuric acid-6294	2/100	0.197972	0.408923	0.269513	0.556697	-0.69492	1.125511	CEACAM6;CEACAM5

azacyclonol-6298	2/100	0.197972	0.408923	0.269513	0.556697	-0.69451	1.124852	IFI6;ISG15
benfluorex-1266	2/100	0.197972	0.408923	0.269513	0.556697	-0.69373	1.123588	GPX2;IFI6
abamectin-2519	2/100	0.197972	0.408923	0.269513	0.556697	-0.69323	1.122778	IFITM1;IGFBP2
bethanechol-5539	2/100	0.197972	0.408923	0.269513	0.556697	-0.69272	1.121957	IFITM1;IFI6
atropine oxide-6812	2/100	0.197972	0.408923	0.269513	0.556697	-0.69175	1.120378	IFITM1;SECTM1
atractyloside-4717	2/100	0.197972	0.408923	0.269513	0.556697	-0.69173	1.120355	TFF2;SECTM1
bepridil-4613	2/100	0.197972	0.408923	0.269513	0.556697	-0.69104	1.119238	C3;CTSE
beclometasone-3001	2/100	0.197972	0.408923	0.269513	0.556697	-0.6903	1.118028	PIGR;DCN
astemizole-2049	2/100	0.197972	0.408923	0.269513	0.556697	-0.68931	1.116436	CCL20;IFI6
benperidol-4781	2/100	0.197972	0.408923	0.269513	0.556697	-0.689	1.115934	COL1A2;CEACAM5
BCB000040-7493	2/100	0.197972	0.408923	0.269513	0.556697	-0.68888	1.115733	PIGR;IFI6
androsterone-2650	2/100	0.197972	0.408923	0.269513	0.556697	-0.68643	1.111761	PIGR;ITM2C
ampyrone-2086	2/100	0.197972	0.408923	0.269513	0.556697	-0.68623	1.111447	CCL20;IFI6
amiprilose-4000	2/100	0.197972	0.408923	0.269513	0.556697	-0.68556	1.11035	IL32;TSPAN8
BAS-012416453-6876	2/100	0.197972	0.408923	0.269513	0.556697	-0.68539	1.110072	CEACAM6;QPCT
betahistine-2833	2/100	0.197972	0.408923	0.269513	0.556697	-0.68344	1.106918	SLPI;AP1S1
BCB000040-7559	2/100	0.197972	0.408923	0.269513	0.556697	-0.68055	1.102236	LGALS3BP;APP
betaxolol-1592	2/100	0.197972	0.408923	0.269513	0.556697	-0.67608	1.095002	AP1S1;PTMA
atropine-2761	2/100	0.197972	0.408923	0.269513	0.556697	-0.67606	1.094966	COL1A1;MMP3
brompheniramine-1335	2/100	0.197972	0.408923	0.269513	0.556697	-0.67418	1.091923	IFITM3;PLCB4
asiaticoside-2943	2/100	0.197972	0.408923	0.269513	0.556697	-0.67019	1.085461	TCN1;AP1S1
benzathine benzylpenicillin-7359	2/100	0.197972	0.408923	0.269513	0.556697	-0.66909	1.083679	SECTM1;PHLDA1
benzamil-3738	2/100	0.197972	0.408923	0.269513	0.556697	-0.66733	1.080826	PIGR;CEACAM5
benzylpenicillin-6839	2/100	0.197972	0.408923	0.269513	0.556697	-0.66533	1.077589	CTSE;DCN
betulinic acid-4181	2/100	0.197972	0.408923	0.269513	0.556697	-0.66495	1.076979	PIGR;CEACAM5
benzethonium chloride-2508	2/100	0.197972	0.408923	0.269513	0.556697	-0.66492	1.076929	COL1A2;DCN
benzonatate-6334	2/100	0.197972	0.408923	0.269513	0.556697	-0.6627	1.073323	IFI6;TGFBI
atovaquone-4201	2/100	0.197972	0.408923	0.269513	0.556697	-0.66105	1.070662	IFITM1;MMP3
berberine-6791	2/100	0.197972	0.408923	0.269513	0.556697	-0.6584	1.066365	CTSE;PHLDA1
buspirone-2637	2/100	0.197972	0.408923	0.269513	0.556697	-0.65829	1.066179	RNF43;AP1S1
atropine oxide-4476	2/100	0.197972	0.408923	0.269513	0.556697	-0.65826	1.066143	JUND;MMP3
cefapirin-6790	2/100	0.197972	0.408923	0.269513	0.556697	-0.65758	1.065038	IFI27;MMP3
azaperone-5877	2/100	0.197972	0.408923	0.269513	0.556697	-0.65756	1.065004	CEACAM5;IFI6
captopril-1988	2/100	0.197972	0.408923	0.269513	0.556697	-0.65692	1.063965	ENC1;PTMA
bisacodyl-4616	2/100	0.197972	0.408923	0.269513	0.556697	-0.65665	1.063531	FOS;CTSE
bupropion-1564	2/100	0.197972	0.408923	0.269513	0.556697	-0.65661	1.063463	TPI1;CCL20
cefotaxime-2235	2/100	0.197972	0.408923	0.269513	0.556697	-0.65477	1.060484	COL1A1;RNF43

brinzolamide-3230	2/100	0.197972	0.408923	0.269513	0.556697	-0.65426	1.059655	SPINK1;DCN
azlocillin-3468	2/100	0.197972	0.408923	0.269513	0.556697	-0.65403	1.05928	AP1S1;SOX9
alpha-estradiol-1635	2/100	0.197972	0.408923	0.269513	0.556697	-0.6535	1.058431	TMPRSS3;ETS2
calcium folinate-4725	2/100	0.197972	0.408923	0.269513	0.556697	-0.65344	1.058339	SERPINA1;IFI6
biotin-3289	2/100	0.197972	0.408923	0.269513	0.556697	-0.65292	1.057497	AP1S1;PDZK1IP1
betonicine-2207	2/100	0.197972	0.408923	0.269513	0.556697	-0.65247	1.056762	GPX2;TCN1
butyl hydroxybenzoate-5245	2/100	0.197972	0.408923	0.269513	0.556697	-0.65054	1.053641	COL1A1;C3
bicuculline-4397	2/100	0.197972	0.408923	0.269513	0.556697	-0.65006	1.05285	PIGR;ITM2C
bendroflumethiazide-3934	2/100	0.197972	0.408923	0.269513	0.556697	-0.64947	1.051903	COL1A1;C3
benzonatate-1801	2/100	0.197972	0.408923	0.269513	0.556697	-0.64835	1.050081	AP1S1;ITM2B
bezafibrate-4999	2/100	0.197972	0.408923	0.269513	0.556697	-0.6483	1.049999	SPINK1;MMP3
carbarsone-1313	2/100	0.197972	0.408923	0.269513	0.556697	-0.64675	1.047489	IFITM3;ATP1B1
beta-escin-6050	2/100	0.197972	0.408923	0.269513	0.556697	-0.64668	1.047382	TCN1;PHLDA1
brompheniramine-3271	2/100	0.197972	0.408923	0.269513	0.556697	-0.64372	1.042584	IFI6;TFF2
biotin-6689	2/100	0.197972	0.408923	0.269513	0.556697	-0.64089	1.038011	JUND;TSPAN1
carbinoxamine-3466	2/100	0.197972	0.408923	0.269513	0.556697	-0.64071	1.037716	SERPINA1;CD24
carbamazepine-5093	2/100	0.197972	0.408923	0.269513	0.556697	-0.63914	1.035177	BMP4;S100A11
butoconazole-6288	2/100	0.197972	0.408923	0.269513	0.556697	-0.63749	1.032493	CEACAM6;IFI6
ceforanide-3309	2/100	0.197972	0.408923	0.269513	0.556697	-0.63669	1.03121	S100A11;DCN
beta-escin-3890	2/100	0.197972	0.408923	0.269513	0.556697	-0.63425	1.027248	FOS;PHLDA1
betazole-1812	2/100	0.197972	0.408923	0.269513	0.556697	-0.63284	1.024972	MMP3;AP1S1
betazole-1854	2/100	0.197972	0.408923	0.269513	0.556697	-0.62991	1.020219	ID3;TIMP1
calmidazolium-906	2/100	0.197972	0.408923	0.269513	0.556697	-0.62896	1.018677	FOS;TGFBI
bromperidol-1723	2/100	0.197972	0.408923	0.269513	0.556697	-0.62798	1.017104	IFITM3;APP
bumetanide-5117	2/100	0.197972	0.408923	0.269513	0.556697	-0.62727	1.01594	IL32;IFI6
camptothecin-4541	2/100	0.197972	0.408923	0.269513	0.556697	-0.62672	1.015055	CD14;FOS
bromocriptine-2007	2/100	0.197972	0.408923	0.269513	0.556697	-0.62627	1.014329	CCL20;TIMP1
biperiden-5644	2/100	0.197972	0.408923	0.269513	0.556697	-0.62606	1.013979	IFI27;IFI6
betulinic acid-4101	2/100	0.197972	0.408923	0.269513	0.556697	-0.62028	1.004624	FABP1;APP
brompheniramine-4131	2/100	0.197972	0.408923	0.269513	0.556697	-0.61772	1.000481	COL1A1;SPARC
carteolol-4176	2/100	0.197972	0.408923	0.269513	0.556697	-0.6176	1.000284	IFI27;IFI6
carbimazole-3299	2/100	0.197972	0.408923	0.269513	0.556697	-0.61675	0.9989	SERPINA1;IFI6
carbamazepine-1683	2/100	0.197972	0.408923	0.269513	0.556697	-0.61626	0.99811	IFITM1;IFI6
celecoxib-922	2/100	0.197972	0.408923	0.269513	0.556697	-0.61467	0.995538	TXNIP;FOS
bromopride-2182	2/100	0.197972	0.408923	0.269513	0.556697	-0.61423	0.994822	TCN1;SOX9
carmustine-6883	2/100	0.197972	0.408923	0.269513	0.556697	-0.61014	0.988202	CEACAM6;AP1S1
cefamandole-3436	2/100	0.197972	0.408923	0.269513	0.556697	-0.61003	0.988024	SERPINA1;ITM2C

captopril-1907	2/100	0.197972	0.408923	0.269513	0.556697	-0.60805	0.984813	TPI1;TGFBI
budesonide-1716	2/100	0.197972	0.408923	0.269513	0.556697	-0.60741	0.983788	ACSL1;IFNGR1
ceftazidime-5054	2/100	0.197972	0.408923	0.269513	0.556697	-0.60697	0.983069	IFITM1;DCN
cefotaxime-5830	2/100	0.197972	0.408923	0.269513	0.556697	-0.60628	0.981954	GPX2;ETS2
chlortalidone-6800	2/100	0.197972	0.408923	0.269513	0.556697	-0.60539	0.980504	CEACAM5;SOX9
brinzolamide-1615	2/100	0.197972	0.408923	0.269513	0.556697	-0.60391	0.97811	IFITM3;AP1S1
cefalexin-5250	2/100	0.197972	0.408923	0.269513	0.556697	-0.60312	0.976834	IFITM1;IFI6
clofazimine-3239	2/100	0.197972	0.408923	0.269513	0.556697	-0.60123	0.973766	C3;DCN
carbimazole-2437	2/100	0.197972	0.408923	0.269513	0.556697	-0.60005	0.971865	NQO1;CEACAM5
cefalexin-2628	2/100	0.197972	0.408923	0.269513	0.556697	-0.60005	0.971864	C3;CEACAM5
chlorambucil-3869	2/100	0.197972	0.408923	0.269513	0.556697	-0.59829	0.969013	DCN;CTGF
calcium pantothenate-3248	2/100	0.197972	0.408923	0.269513	0.556697	-0.59797	0.968488	C3;S100A6
chlorambucil-3788	2/100	0.197972	0.408923	0.269513	0.556697	-0.59709	0.96707	IL32;PIGR
cisapride-6706	2/100	0.197972	0.408923	0.269513	0.556697	-0.59621	0.965637	C3;OLFM4
citiolone-2176	2/100	0.197972	0.408923	0.269513	0.556697	-0.59464	0.963089	FXYD3;CD24
carbimazole-5399	2/100	0.197972	0.408923	0.269513	0.556697	-0.59423	0.962438	TMPRSS3;S100A11
ciclopirox-2456	2/100	0.197972	0.408923	0.269513	0.556697	-0.59303	0.960491	FOS;PHLDA1
cefadroxil-4080	2/100	0.197972	0.408923	0.269513	0.556697	-0.59295	0.960355	IL32;APP
cefixime-1310	2/100	0.197972	0.408923	0.269513	0.556697	-0.59179	0.958484	TSPAN13;LY6E
cloperastine-3408	2/100	0.197972	0.408923	0.269513	0.556697	-0.58981	0.955281	SOX9;PHLDA1
ciclacillin-3882	2/100	0.197972	0.408923	0.269513	0.556697	-0.58935	0.954538	COL1A1;TCN1
chlorogenic acid-4142	2/100	0.197972	0.408923	0.269513	0.556697	-0.58895	0.953882	COL1A1;CEACAM5
ciclopirox-5023	2/100	0.197972	0.408923	0.269513	0.556697	-0.58751	0.951542	FOS;PGM1
clebopride-6311	2/100	0.197972	0.408923	0.269513	0.556697	-0.58676	0.950338	C3;IGFBP2
clioquinol-4663	2/100	0.197972	0.408923	0.269513	0.556697	-0.58629	0.949578	COL1A1;IFITM1
cefotetan-3997	2/100	0.197972	0.408923	0.269513	0.556697	-0.58519	0.94779	COL1A1;C3
cefoxitin-3477	2/100	0.197972	0.408923	0.269513	0.556697	-0.58383	0.945595	IFITM1;IFI6
chlorpromazine-1700	2/100	0.197972	0.408923	0.269513	0.556697	-0.58349	0.945031	JUND;ITM2B
cefalotin-2517	2/100	0.197972	0.408923	0.269513	0.556697	-0.58162	0.942009	COL1A2;CD24
CP-320650-01-3905	2/100	0.197972	0.408923	0.269513	0.556697	-0.57925	0.938174	S100A11;ITM2B
cefixime-4567	2/100	0.197972	0.408923	0.269513	0.556697	-0.57826	0.936563	IL32;MMP12
cinchocaine-1469	2/100	0.197972	0.408923	0.269513	0.556697	-0.5778	0.935824	C3;PTPRO
ciprofloxacin-6700	2/100	0.197972	0.408923	0.269513	0.556697	-0.57676	0.934133	C3;MMP3
chloropyramine-4589	2/100	0.197972	0.408923	0.269513	0.556697	-0.57675	0.934119	BMP4;CXCL1
cyclopenthiazide-4813	2/100	0.197972	0.408923	0.269513	0.556697	-0.57627	0.93334	IFI6;ITM2C
clomifene-6648	2/100	0.197972	0.408923	0.269513	0.556697	-0.57571	0.932437	FOS;DUOX2
chlorpromazine-1158	2/100	0.197972	0.408923	0.269513	0.556697	-0.57426	0.930085	JUND;MMP3

cefamandole-7394	2/100	0.197972	0.408923	0.269513	0.556697	-0.57399	0.929659	IL32;TSPAN8
cefazolin-3686	2/100	0.197972	0.408923	0.269513	0.556697	-0.57351	0.928879	IL32;C3
conessine-2792	2/100	0.197972	0.408923	0.269513	0.556697	-0.57271	0.927578	COL1A1;ITM2C
colforsin-7055	2/100	0.197972	0.408923	0.269513	0.556697	-0.5722	0.926755	TMPRSS3;ETS2
cephaeline-4651	2/100	0.197972	0.408923	0.269513	0.556697	-0.56728	0.918781	TXNIP;ID3
bucladesine-3483	2/100	0.197972	0.408923	0.269513	0.556697	-0.56706	0.918421	PIGR;SOX9
clomifene-2624	2/100	0.197972	0.408923	0.269513	0.556697	-0.56654	0.917582	FOS;S100A11
chlorprothixene-2627	2/100	0.197972	0.408923	0.269513	0.556697	-0.56548	0.915874	FOS;IER2
clotrimazole-905	2/100	0.197972	0.408923	0.269513	0.556697	-0.56366	0.912922	SOX9;PHLDA1
chloropyrazine-6148	2/100	0.197972	0.408923	0.269513	0.556697	-0.56163	0.909635	TGFBI;PROM1
chlorpromazine-6936	2/100	0.197972	0.408923	0.269513	0.556697	-0.56126	0.909038	SERPINA1;CEACAM6
clozapine-5265	2/100	0.197972	0.408923	0.269513	0.556697	-0.56054	0.907873	AP1S1;DCN
chloramphenicol-5047	2/100	0.197972	0.408923	0.269513	0.556697	-0.56034	0.90755	COL1A1;CEACAM6
chlorzoxazone-2100	2/100	0.197972	0.408923	0.269513	0.556697	-0.5594	0.906021	BMP4;AP1S1
clioquinol-3084	2/100	0.197972	0.408923	0.269513	0.556697	-0.55709	0.902286	MMP1;PHLDA1
clomipramine-6825	2/100	0.197972	0.408923	0.269513	0.556697	-0.55572	0.900056	FOS;PHLDA1
deferoxamine-3417	2/100	0.197972	0.408923	0.269513	0.556697	-0.55352	0.896504	RNF43;S100A4
chlorprothixene-6692	2/100	0.197972	0.408923	0.269513	0.556697	-0.55191	0.893884	JUND;FOS
chlorprothixene-5291	2/100	0.197972	0.408923	0.269513	0.556697	-0.55078	0.892067	FOS;IER2
CP-645525-01-7515	2/100	0.197972	0.408923	0.269513	0.556697	-0.54927	0.889609	ACSL1;SOX9
clotrimazole-3166	2/100	0.197972	0.408923	0.269513	0.556697	-0.54788	0.887358	APP;TFF2
clozapine-2644	2/100	0.197972	0.408923	0.269513	0.556697	-0.54726	0.886366	COL1A1;RNF43
dapsone-5498	2/100	0.197972	0.408923	0.269513	0.556697	-0.54575	0.883907	TMPRSS3;S100A4
ciprofloxacin-2022	2/100	0.197972	0.408923	0.269513	0.556697	-0.54535	0.883272	C3;LY6E
demecarium bromide-6269	2/100	0.197972	0.408923	0.269513	0.556697	-0.54531	0.883198	BMP4;ETS2
dextromethorphan-6300	2/100	0.197972	0.408923	0.269513	0.556697	-0.54378	0.880726	FXYD3;IFI6
corynanthine-2786	2/100	0.197972	0.408923	0.269513	0.556697	-0.54025	0.874999	CEACAM5;SECTM1
clobetasol-6095	2/100	0.197972	0.408923	0.269513	0.556697	-0.53905	0.873057	DCN;ITM2C
colistin-4212	2/100	0.197972	0.408923	0.269513	0.556697	-0.53753	0.8706	CEACAM6;MMP3
co-dergocrine mesilate-4071	2/100	0.197972	0.408923	0.269513	0.556697	-0.53531	0.867001	BMP4;SLCO5A1
cyclic adenosine monophosphate-5533	2/100	0.197972	0.408923	0.269513	0.556697	-0.53361	0.864257	COL1A1;AP1S1
cyproterone-6806	2/100	0.197972	0.408923	0.269513	0.556697	-0.53278	0.862902	TFF2;CTSE
delsoline-2513	2/100	0.197972	0.408923	0.269513	0.556697	-0.52995	0.858328	IFITM1;PHLDA1
dehydrocholic acid-2023	2/100	0.197972	0.408923	0.269513	0.556697	-0.52928	0.857239	GPX2;AP1S1
copper sulfate-459	2/100	0.197972	0.408923	0.269513	0.556697	-0.52819	0.855466	CDH3;CTGF
clofazimine-1624	2/100	0.197972	0.408923	0.269513	0.556697	-0.52734	0.854103	IFITM3;CCL20

cyproheptadine-2021	2/100	0.197972	0.408923	0.269513	0.556697	-0.52556	0.851211	APP;GPX2
clomipramine-3182	2/100	0.197972	0.408923	0.269513	0.556697	-0.52554	0.851184	COL1A1;MMP3
equilin-5620	2/100	0.197972	0.408923	0.269513	0.556697	-0.52429	0.849151	PHLDA1;DUOX2
convolamine-1779	2/100	0.197972	0.408923	0.269513	0.556697	-0.52381	0.848372	GPX2;PTMA
corbadrine-2710	2/100	0.197972	0.408923	0.269513	0.556697	-0.52078	0.843466	ID3;PHLDA1
cortisone-7416	2/100	0.197972	0.408923	0.269513	0.556697	-0.52012	0.842398	SECTM1;PHLDA1
dapsone-1827	2/100	0.197972	0.408923	0.269513	0.556697	-0.51937	0.841192	IFI6;AP1S1
dexamethasone-123	2/100	0.197972	0.408923	0.269513	0.556697	-0.51918	0.840887	GPX2;CEACAM5
clorgiline-1604	2/100	0.197972	0.408923	0.269513	0.556697	-0.51636	0.836309	RPS14P3;ANXA3
dexverapamil-164	2/100	0.197972	0.408923	0.269513	0.556697	-0.51604	0.835788	GPX2;SLPI
desipramine-1596	2/100	0.197972	0.408923	0.269513	0.556697	-0.51509	0.834263	CCL20;PTMA
debrisoquine-6688	2/100	0.197972	0.408923	0.269513	0.556697	-0.51397	0.832437	JUND;AP1S1
dihydrostreptomycin-6228	2/100	0.197972	0.408923	0.269513	0.556697	-0.51313	0.831084	COL1A1;TFF2
dihydroergotamine-2081	2/100	0.197972	0.408923	0.269513	0.556697	-0.51263	0.830274	CCL20;IFI6
difenidol-7406	2/100	0.197972	0.408923	0.269513	0.556697	-0.51205	0.829327	IFITM1;GPX2
diclofenac-5861	2/100	0.197972	0.408923	0.269513	0.556697	-0.5106	0.826984	TSPAN8;DCN
dienestrol-6208	2/100	0.197972	0.408923	0.269513	0.556697	-0.51027	0.826455	FOS;PHLDA1
dimenhydrinate-2400	2/100	0.197972	0.408923	0.269513	0.556697	-0.50908	0.82452	GPX2;CD24
deferoxamine-3760	2/100	0.197972	0.408923	0.269513	0.556697	-0.50864	0.823808	PDZK1IP1;DUOX2
dantrolene-2329	2/100	0.197972	0.408923	0.269513	0.556697	-0.50818	0.82306	CEACAM5;IFI6
diperodon-6836	2/100	0.197972	0.408923	0.269513	0.556697	-0.50733	0.821689	COL1A1;TFF2
digoxin-6724	2/100	0.197972	0.408923	0.269513	0.556697	-0.50633	0.820063	TXNIP;FOS
corticosterone-4145	2/100	0.197972	0.408923	0.269513	0.556697	-0.50431	0.816801	IFI27;IFI6
diphenylpyraline-3743	2/100	0.197972	0.408923	0.269513	0.556697	-0.5039	0.816131	IL32;COL1A2
dexpanthenol-7455	2/100	0.197972	0.408923	0.269513	0.556697	-0.50389	0.816123	TFF2;SECTM1
cycloserine-6139	2/100	0.197972	0.408923	0.269513	0.556697	-0.50335	0.815246	MMP7;PHLDA1
dacarbazine-1762	2/100	0.197972	0.408923	0.269513	0.556697	-0.50186	0.812825	IFITM3;SLC12A2
cyclic adenosine monophosphate-3531	2/100	0.197972	0.408923	0.269513	0.556697	-0.5016	0.812402	C3;S100A11
dihydrostreptomycin-2237	2/100	0.197972	0.408923	0.269513	0.556697	-0.50125	0.811834	COL1A1;AP1S1
deptropine-5543	2/100	0.197972	0.408923	0.269513	0.556697	-0.50102	0.811475	IFITM1;SPINK1
dexamethasone-1396	2/100	0.197972	0.408923	0.269513	0.556697	-0.50077	0.811058	CCL20;ACSL1
deferoxamine-573	2/100	0.197972	0.408923	0.269513	0.556697	-0.49972	0.809357	SOX9;PGM1
cycloserine-3562	2/100	0.197972	0.408923	0.269513	0.556697	-0.49938	0.808816	COL1A2;AP1S1
CP-863187-7553	2/100	0.197972	0.408923	0.269513	0.556697	-0.49906	0.808292	ENC1;TXNIP
desoxycortone-3099	2/100	0.197972	0.408923	0.269513	0.556697	-0.49864	0.807619	GPX2;FXYD3
dihydrostreptomycin-2074	2/100	0.197972	0.408923	0.269513	0.556697	-0.49653	0.804196	BMP4;IFI6

diltiazem-2032	2/100	0.197972	0.408923	0.269513	0.556697	-0.49597	0.803287	NQO1;GPX2
demecarium bromide-2773	2/100	0.197972	0.408923	0.269513	0.556697	-0.49497	0.801668	COL1A1;PDZK1IP1
ellipticine-6253	2/100	0.197972	0.408923	0.269513	0.556697	-0.48987	0.793415	SOX9;CTGF
dihydroergocristine-7034	2/100	0.197972	0.408923	0.269513	0.556697	-0.48571	0.786677	COL1A1;SOX9
cyanocobalamin-4395	2/100	0.197972	0.408923	0.269513	0.556697	-0.48467	0.784995	COL1A1;ITM2C
diphenylpyraline-2205	2/100	0.197972	0.408923	0.269513	0.556697	-0.48438	0.784509	TFF2;PDZK1IP1
dinoprost-3308	2/100	0.197972	0.408923	0.269513	0.556697	-0.48388	0.783708	PHLDA1;DCN
doxylamine-4819	2/100	0.197972	0.408923	0.269513	0.556697	-0.48349	0.783073	CEACAM5;MMP3
demeclocycline-3404	2/100	0.197972	0.408923	0.269513	0.556697	-0.483	0.782287	RNF43;SECTM1
diclofenamide-3027	2/100	0.197972	0.408923	0.269513	0.556697	-0.48211	0.780845	PIGR;FXYD3
diazoxide-7168	2/100	0.197972	0.408923	0.269513	0.556697	-0.48112	0.77924	IFITM1;ITM2B
dizocilpine-2232	2/100	0.197972	0.408923	0.269513	0.556697	-0.47803	0.774238	TCN1;S100A6
dobutamine-6286	2/100	0.197972	0.408923	0.269513	0.556697	-0.47779	0.773836	S100P;CD24
diphenylpyraline-6061	2/100	0.197972	0.408923	0.269513	0.556697	-0.47712	0.772766	QPCT;IFI6
diazoxide-5810	2/100	0.197972	0.408923	0.269513	0.556697	-0.47708	0.772699	IFI6;AP1S1
dosulepin-2864	2/100	0.197972	0.408923	0.269513	0.556697	-0.47496	0.769255	COL1A1;MMP3
diphenhydramine-1871	2/100	0.197972	0.408923	0.269513	0.556697	-0.47466	0.76878	CCL20;PHLDA1
disulfiram-6210	2/100	0.197972	0.408923	0.269513	0.556697	-0.47258	0.76541	BMP4;PHLDA1
dimenhydrinate-5450	2/100	0.197972	0.408923	0.269513	0.556697	-0.47205	0.764542	SLPI;IFI6
diphenhydramine-7318	2/100	0.197972	0.408923	0.269513	0.556697	-0.47188	0.764275	JUND;SERPINA1
dipyridamole-2017	2/100	0.197972	0.408923	0.269513	0.556697	-0.47107	0.762953	ISG15;FOS
doxorubicin-5671	2/100	0.197972	0.408923	0.269513	0.556697	-0.47034	0.761778	S100A6;S100A11
doxazosin-3363	2/100	0.197972	0.408923	0.269513	0.556697	-0.46839	0.758622	MMP3;DCN
droperidol-5690	2/100	0.197972	0.408923	0.269513	0.556697	-0.46628	0.755209	COL1A2;TCN1
dosulepin-7284	2/100	0.197972	0.408923	0.269513	0.556697	-0.46385	0.751259	AP1S1;ITM2B
dosulepin-5986	2/100	0.197972	0.408923	0.269513	0.556697	-0.46261	0.749254	APP;SECTM1
drofenine-3455	2/100	0.197972	0.408923	0.269513	0.556697	-0.46177	0.747901	IFITM1;IFI6
etacrynic acid-3181	2/100	0.197972	0.408923	0.269513	0.556697	-0.46	0.745035	CEACAM5;PHLDA1
doxazosin-3024	2/100	0.197972	0.408923	0.269513	0.556697	-0.4594	0.744056	PIGR;IFI6
ellipticine-1765	2/100	0.197972	0.408923	0.269513	0.556697	-0.4592	0.743732	IFITM3;ATP1B1
dioxybenzone-3101	2/100	0.197972	0.408923	0.269513	0.556697	-0.45784	0.741533	NQO1;TFF2
diflunisal-4794	2/100	0.197972	0.408923	0.269513	0.556697	-0.45762	0.741183	COL1A1;BMP4
eldeline-2171	2/100	0.197972	0.408923	0.269513	0.556697	-0.45692	0.740039	CEACAM6;TFF2
domperidone-4640	2/100	0.197972	0.408923	0.269513	0.556697	-0.45467	0.736391	CXCL1;CTGF
econazole-7427	2/100	0.197972	0.408923	0.269513	0.556697	-0.45463	0.736335	FOS;PHLDA1
ethotoin-4545	2/100	0.197972	0.408923	0.269513	0.556697	-0.45055	0.729724	APP;AP1S1
dirithromycin-7446	2/100	0.197972	0.408923	0.269513	0.556697	-0.44993	0.72872	SLPI;DCN

ebselen-3458	2/100	0.197972	0.408923	0.269513	0.556697	-0.44854	0.726466	GPX2;QPCT
erythromycin-1510	2/100	0.197972	0.408923	0.269513	0.556697	-0.44849	0.726388	COL1A1;FOS
estradiol-1021	2/100	0.197972	0.408923	0.269513	0.556697	-0.44612	0.72255	APP;TMPRSS3
dizocilpine-2069	2/100	0.197972	0.408923	0.269513	0.556697	-0.44448	0.719888	CCL20;IFI6
estradiol-5238	2/100	0.197972	0.408923	0.269513	0.556697	-0.44112	0.714454	MMP12;COL1A1
diphenylpyraline-4765	2/100	0.197972	0.408923	0.269513	0.556697	-0.43925	0.711428	COL1A1;SPARC
ethoxyquin-2559	2/100	0.197972	0.408923	0.269513	0.556697	-0.43914	0.711251	COL1A2;DCN
epitiostanol-4204	2/100	0.197972	0.408923	0.269513	0.556697	-0.43851	0.710218	IFITM1;IFI6
estropipate-6808	2/100	0.197972	0.408923	0.269513	0.556697	-0.4356	0.705504	TFF2;SOX9
fenbufen-3721	2/100	0.197972	0.408923	0.269513	0.556697	-0.43468	0.704018	IL32;APP
diphenhydramine-1830	2/100	0.197972	0.408923	0.269513	0.556697	-0.43413	0.703137	CCL20;PHLDA1
fenbufen-4743	2/100	0.197972	0.408923	0.269513	0.556697	-0.43392	0.702793	COL1A1;IFI6
econazole-7305	2/100	0.197972	0.408923	0.269513	0.556697	-0.43249	0.700467	IFITM1;IFI6
estradiol-1149	2/100	0.197972	0.408923	0.269513	0.556697	-0.43238	0.700302	APP;GPX2
drofenine-7129	2/100	0.197972	0.408923	0.269513	0.556697	-0.42999	0.696421	APP;TSPAN1
enoxacin-5251	2/100	0.197972	0.408923	0.269513	0.556697	-0.42963	0.695843	COL1A1;PHLDA1
equilin-3039	2/100	0.197972	0.408923	0.269513	0.556697	-0.42949	0.695622	COL1A1;TCN1
epivincamine-2775	2/100	0.197972	0.408923	0.269513	0.556697	-0.429	0.694821	MMP3;AP1S1
edrophonium chloride-1936	2/100	0.197972	0.408923	0.269513	0.556697	-0.42847	0.69396	AP1S1;LY6E
enalapril-7265	2/100	0.197972	0.408923	0.269513	0.556697	-0.42833	0.693736	SPINK1;CEACAM6
dydrogesterone-2811	2/100	0.197972	0.408923	0.269513	0.556697	-0.42807	0.693323	PTPRO;ITM2C
erythromycin-1928	2/100	0.197972	0.408923	0.269513	0.556697	-0.42643	0.690658	IFITM1;IFI6
erythromycin-5329	2/100	0.197972	0.408923	0.269513	0.556697	-0.42627	0.690407	IFITM1;CEACAM6
doxycycline-2737	2/100	0.197972	0.408923	0.269513	0.556697	-0.42574	0.689538	CTSE;OLFM4
ethambutol-1481	2/100	0.197972	0.408923	0.269513	0.556697	-0.42536	0.688927	CEACAM5;FOS
dobutamine-5386	2/100	0.197972	0.408923	0.269513	0.556697	-0.42418	0.687016	C3;APP
flupentixol-2643	2/100	0.197972	0.408923	0.269513	0.556697	-0.42388	0.686534	C3;DCN
flavoxate-5427	2/100	0.197972	0.408923	0.269513	0.556697	-0.42228	0.68394	COL1A1;CXCL1
equilin-3377	2/100	0.197972	0.408923	0.269513	0.556697	-0.4212	0.682181	AP1S1;PHLDA1
ellipticine-5779	2/100	0.197972	0.408923	0.269513	0.556697	-0.42114	0.682095	BMP4;FOS
equilin-5255	2/100	0.197972	0.408923	0.269513	0.556697	-0.41937	0.679221	C3;SOX9
flunisolide-3747	2/100	0.197972	0.408923	0.269513	0.556697	-0.41627	0.674211	SPINK1;IFI27
epitiostanol-2922	2/100	0.197972	0.408923	0.269513	0.556697	-0.41626	0.674193	TSPAN8;IGFBP2
estradiol-2668	2/100	0.197972	0.408923	0.269513	0.556697	-0.41441	0.671198	MMP3;DCN
etifenin-3998	2/100	0.197972	0.408923	0.269513	0.556697	-0.41393	0.670409	TSPAN8;CEACAM6
etifenin-4117	2/100	0.197972	0.408923	0.269513	0.556697	-0.41359	0.66986	COL1A1;PSMB9
estradiol-5318	2/100	0.197972	0.408923	0.269513	0.556697	-0.41346	0.669651	JUND;S100A4

fenoprofen-4274	2/100	0.197972	0.408923	0.269513	0.556697	-0.41248	0.668072	IL32;PHLDA1
flumequine-5529	2/100	0.197972	0.408923	0.269513	0.556697	-0.41045	0.664772	IFITM1;IFI6
eucatropine-4316	2/100	0.197972	0.408923	0.269513	0.556697	-0.40862	0.66181	IL32;SERPINA1
estropipate-2506	2/100	0.197972	0.408923	0.269513	0.556697	-0.40723	0.659556	SPINK1;CD24
F0447-0125-6396	2/100	0.197972	0.408923	0.269513	0.556697	-0.40609	0.657722	SLPI;SECTM1
estradiol-5205	2/100	0.197972	0.408923	0.269513	0.556697	-0.40604	0.657639	COL1A1;C3
estradiol-1079	2/100	0.197972	0.408923	0.269513	0.556697	-0.40516	0.656202	PHLDA1;ETS2
ethotoin-2196	2/100	0.197972	0.408923	0.269513	0.556697	-0.40365	0.653762	IFITM1;CD24
etofenamate-7327	2/100	0.197972	0.408923	0.269513	0.556697	-0.40044	0.648568	CEACAM5;TFF2
fenspiride-6001	2/100	0.197972	0.408923	0.269513	0.556697	-0.40014	0.648076	PHLDA1;ITM2C
etofylline-5048	2/100	0.197972	0.408923	0.269513	0.556697	-0.39871	0.645769	MMP3;PSMB9
etofylline-5467	2/100	0.197972	0.408923	0.269513	0.556697	-0.39845	0.645346	SOX9;PHLDA1
felodipine-5294	2/100	0.197972	0.408923	0.269513	0.556697	-0.39675	0.642587	PHLDA1;IER2
fluphenazine-1017	2/100	0.197972	0.408923	0.269513	0.556697	-0.39463	0.639162	SOX9;PHLDA1
ethionamide-4418	2/100	0.197972	0.408923	0.269513	0.556697	-0.39447	0.638901	SECTM1;ITM2C
fludroxycortide-2184	2/100	0.197972	0.408923	0.269513	0.556697	-0.39429	0.638597	CCL20;ACSL1
ethisterone-3864	2/100	0.197972	0.408923	0.269513	0.556697	-0.39355	0.637399	COL1A1;CEACAM5
fluphenazine-2697	2/100	0.197972	0.408923	0.269513	0.556697	-0.39116	0.633536	COL1A1;IFI6
ethambutol-1900	2/100	0.197972	0.408923	0.269513	0.556697	-0.38964	0.631072	SERPINA1;CEACAM5
flumequine-2276	2/100	0.197972	0.408923	0.269513	0.556697	-0.38884	0.629775	PIGR;IFI6
famotidine-2029	2/100	0.197972	0.408923	0.269513	0.556697	-0.38786	0.628196	C3;AP1S1
famotidine-5011	2/100	0.197972	0.408923	0.269513	0.556697	-0.38723	0.627168	MMP3;CTSE
flecainide-4318	2/100	0.197972	0.408923	0.269513	0.556697	-0.38713	0.627007	IL32;RNF43
etamsylate-7335	2/100	0.197972	0.408923	0.269513	0.556697	-0.38581	0.624873	IFI27;MMP3
finasteride-6062	2/100	0.197972	0.408923	0.269513	0.556697	-0.38552	0.624398	IFI6;DCN
etodolac-2254	2/100	0.197972	0.408923	0.269513	0.556697	-0.38502	0.62359	TCN1;LYZ
gibberellic acid-7330	2/100	0.197972	0.408923	0.269513	0.556697	-0.38386	0.621719	SECTM1;PHLDA1
gliquidone-6505	2/100	0.197972	0.408923	0.269513	0.556697	-0.38306	0.620412	TMPRSS3;MMP3
fluphenazine-1237	2/100	0.197972	0.408923	0.269513	0.556697	-0.38289	0.620146	CCL20;FOS
fluphenazine-6954	2/100	0.197972	0.408923	0.269513	0.556697	-0.38249	0.619499	SOX9;PHLDA1
fluphenazine-5880	2/100	0.197972	0.408923	0.269513	0.556697	-0.38136	0.617657	MMP1;AP1S1
etomidate-7460	2/100	0.197972	0.408923	0.269513	0.556697	-0.38103	0.617136	COL1A1;ETS2
fludrocortisone-2368	2/100	0.197972	0.408923	0.269513	0.556697	-0.37957	0.614758	ACSL1;SPP1
fluocinonide-3933	2/100	0.197972	0.408923	0.269513	0.556697	-0.37944	0.614557	FOS;DCN
etacrynic acid-1565	2/100	0.197972	0.408923	0.269513	0.556697	-0.37928	0.614298	NQO1;ANXA2
etodolac-7246	2/100	0.197972	0.408923	0.269513	0.556697	-0.37907	0.61395	GPX2;AP1S1
fludrocortisone-282	2/100	0.197972	0.408923	0.269513	0.556697	-0.3769	0.610439	GPX2;TCN1

flumetasone-3712	2/100	0.197972	0.408923	0.269513	0.556697	-0.37528	0.607814	APP;CCL20
flunixin-3411	2/100	0.197972	0.408923	0.269513	0.556697	-0.37464	0.606778	TMPRSS3;AP1S1
etilefrine-4590	2/100	0.197972	0.408923	0.269513	0.556697	-0.3745	0.606559	S100P;CD24
etofylline-2093	2/100	0.197972	0.408923	0.269513	0.556697	-0.37353	0.604984	TSPAN8;CCL20
flucytosine-6690	2/100	0.197972	0.408923	0.269513	0.556697	-0.37304	0.604186	CEACAM5;MMP3
fenbendazole-3805	2/100	0.197972	0.408923	0.269513	0.556697	-0.36823	0.596396	MMP12;IFITM1
fluphenazine-4461	2/100	0.197972	0.408923	0.269513	0.556697	-0.36679	0.594057	SERPINA1;FOS
fursultiamine-4975	2/100	0.197972	0.408923	0.269513	0.556697	-0.36673	0.593961	BMP4;SERPINA1
fulvestrant-1630	2/100	0.197972	0.408923	0.269513	0.556697	-0.36156	0.585597	RNF43;ID1
fulvestrant-4462	2/100	0.197972	0.408923	0.269513	0.556697	-0.35879	0.581107	PIGR;MMP3
fluphenazine-5597	2/100	0.197972	0.408923	0.269513	0.556697	-0.35747	0.578968	COL1A1;C3
florfenicol-3083	2/100	0.197972	0.408923	0.269513	0.556697	-0.35727	0.57865	COL1A1;TFF2
fulvestrant-6965	2/100	0.197972	0.408923	0.269513	0.556697	-0.35455	0.574247	IFITM1;ID1
flunarizine-7252	2/100	0.197972	0.408923	0.269513	0.556697	-0.35452	0.574185	CEACAM6;ITM2B
gentamicin-2245	2/100	0.197972	0.408923	0.269513	0.556697	-0.3535	0.572534	C3;ITM2C
flurbiprofen-6472	2/100	0.197972	0.408923	0.269513	0.556697	-0.35338	0.572345	RNF43;TCN1
foliosidine-3739	2/100	0.197972	0.408923	0.269513	0.556697	-0.35321	0.57207	COL1A1;SLCO5A1
fluvoxamine-4114	2/100	0.197972	0.408923	0.269513	0.556697	-0.35262	0.571109	SPINK1;CTSE
gibberellic acid-4234	2/100	0.197972	0.408923	0.269513	0.556697	-0.3519	0.569942	IL32;SLCO5A1
gibberellic acid-2910	2/100	0.197972	0.408923	0.269513	0.556697	-0.35138	0.569114	COL1A2;IGFBP2
fluphenazine-6196	2/100	0.197972	0.408923	0.269513	0.556697	-0.34998	0.566841	COL1A1;GPX2
genistein-5232	2/100	0.197972	0.408923	0.269513	0.556697	-0.34669	0.561513	APP;AP1S1
foliosidine-4295	2/100	0.197972	0.408923	0.269513	0.556697	-0.34624	0.560783	SPP1;CD24
fulvestrant-6165	2/100	0.197972	0.408923	0.269513	0.556697	-0.34595	0.560312	CFI;TFF2
fluticasone-2928	2/100	0.197972	0.408923	0.269513	0.556697	-0.34041	0.551331	CCL20;ACSL1
flunisolide-3828	2/100	0.197972	0.408923	0.269513	0.556697	-0.33985	0.550424	COL1A1;TCN1
harmaline-2805	2/100	0.197972	0.408923	0.269513	0.556697	-0.33971	0.550204	COL1A1;MMP3
furosemide-1580	2/100	0.197972	0.408923	0.269513	0.556697	-0.33961	0.550042	CCL20;IFI6
fulvestrant-6997	2/100	0.197972	0.408923	0.269513	0.556697	-0.33847	0.548196	COL1A1;ID1
guanethidine-3171	2/100	0.197972	0.408923	0.269513	0.556697	-0.33735	0.546382	FOS;DUOX2
glimepiride-2809	2/100	0.197972	0.408923	0.269513	0.556697	-0.33568	0.543682	SECTM1;FOS
fursultiamine-6630	2/100	0.197972	0.408923	0.269513	0.556697	-0.33489	0.542392	COL1A1;MMP3
fluorometholone-2509	2/100	0.197972	0.408923	0.269513	0.556697	-0.33142	0.536773	CCL20;CEACAM5
fulvestrant-7096	2/100	0.197972	0.408923	0.269513	0.556697	-0.32986	0.534259	CTSD;ITM2B
guaifenesin-4371	2/100	0.197972	0.408923	0.269513	0.556697	-0.32866	0.532303	C3;AP1S1
fluphenazine-490	2/100	0.197972	0.408923	0.269513	0.556697	-0.32864	0.532268	COL1A2;DPEP1
gemfibrozil-2113	2/100	0.197972	0.408923	0.269513	0.556697	-0.32715	0.529855	APP;CCL20

harmalol-2892	2/100	0.197972	0.408923	0.269513	0.556697	-0.3267	0.529126	COL1A1;FOS
H-7-5941	2/100	0.197972	0.408923	0.269513	0.556697	-0.32664	0.529029	APP;IFI6
fulvestrant-6925	2/100	0.197972	0.408923	0.269513	0.556697	-0.32593	0.527884	SERPINA1;DCN
fulvestrant-7539	2/100	0.197972	0.408923	0.269513	0.556697	-0.32573	0.527567	FXYD3;IFI6
geldanamycin-1228	2/100	0.197972	0.408923	0.269513	0.556697	-0.32509	0.52652	HSPB1;CTGF
fulvestrant-2698	2/100	0.197972	0.408923	0.269513	0.556697	-0.32392	0.524629	COL1A1;PRSS23
gossypol-3637	2/100	0.197972	0.408923	0.269513	0.556697	-0.32308	0.523272	TXNIP;FOS
glycocholic acid-3315	2/100	0.197972	0.408923	0.269513	0.556697	-0.32268	0.522627	IFI27;IFI6
genistein-4459	2/100	0.197972	0.408923	0.269513	0.556697	-0.32113	0.520106	IFI27;CEACAM5
glycocholic acid-2454	2/100	0.197972	0.408923	0.269513	0.556697	-0.32089	0.519723	TFF2;CTSE
fosfosal-2997	2/100	0.197972	0.408923	0.269513	0.556697	-0.32031	0.518791	IGFBP2;DCN
hesperidin-6714	2/100	0.197972	0.408923	0.269513	0.556697	-0.31835	0.515603	MMP3;SLCO5A1
genistein-5595	2/100	0.197972	0.408923	0.269513	0.556697	-0.31802	0.515067	TCN1;SPP1
harmine-7209	2/100	0.197972	0.408923	0.269513	0.556697	-0.31675	0.513013	CEACAM6;SOX9
geldanamycin-1653	2/100	0.197972	0.408923	0.269513	0.556697	-0.31394	0.508465	BMP4;SOX9
geldanamycin-2688	2/100	0.197972	0.408923	0.269513	0.556697	-0.31364	0.507977	COL1A2;HSPB1
fulvestrant-2665	2/100	0.197972	0.408923	0.269513	0.556697	-0.31311	0.507115	ENC1;IFI6
hexylcaine-6244	2/100	0.197972	0.408923	0.269513	0.556697	-0.31166	0.50478	BMP4;APP
hemicholinium-5339	2/100	0.197972	0.408923	0.269513	0.556697	-0.31022	0.502441	BMP4;CEACAM6
heliotrine-2180	2/100	0.197972	0.408923	0.269513	0.556697	-0.31007	0.502203	TCN1;TFF2
furosemide-3197	2/100	0.197972	0.408923	0.269513	0.556697	-0.30701	0.497237	COL1A2;ITM2C
genistein-6194	2/100	0.197972	0.408923	0.269513	0.556697	-0.30654	0.496487	COL1A1;COL1A2
glipizide-2008	2/100	0.197972	0.408923	0.269513	0.556697	-0.30606	0.495708	IFITM3;TCN1
glibenclamide-3163	2/100	0.197972	0.408923	0.269513	0.556697	-0.30423	0.492747	PIGR;CD14
gabapentin-7229	2/100	0.197972	0.408923	0.269513	0.556697	-0.30167	0.488601	COL1A1;CTSE
fusidic acid-1293	2/100	0.197972	0.408923	0.269513	0.556697	-0.2992	0.484587	AP1S1;ATP1B1
gabexate-7357	2/100	0.197972	0.408923	0.269513	0.556697	-0.29716	0.481285	COL1A1;SERPINA1
gelsemine-4097	2/100	0.197972	0.408923	0.269513	0.556697	-0.29639	0.480035	BMP4;APP
GW-8510-7085	2/100	0.197972	0.408923	0.269513	0.556697	-0.29296	0.474493	C3;MMP1
guanfacine-2634	2/100	0.197972	0.408923	0.269513	0.556697	-0.29022	0.470042	COL1A1;PSMB9
geldanamycin-5588	2/100	0.197972	0.408923	0.269513	0.556697	-0.28916	0.468328	BMP4;SOX9
hycanthone-1614	2/100	0.197972	0.408923	0.269513	0.556697	-0.28658	0.46415	CKB;PTMA
guanethidine-5731	2/100	0.197972	0.408923	0.269513	0.556697	-0.28648	0.463993	FXYD3;DUOX2
genistein-2695	2/100	0.197972	0.408923	0.269513	0.556697	-0.28518	0.461888	CCL20;PHLDA1
indapamide-2322	2/100	0.197972	0.408923	0.269513	0.556697	-0.28509	0.461734	SLPI;SPP1
galantamine-4186	2/100	0.197972	0.408923	0.269513	0.556697	-0.28481	0.46129	BMP4;IFITM1
indapamide-2361	2/100	0.197972	0.408923	0.269513	0.556697	-0.28262	0.45774	CEACAM5;CKB

IC-86621-7543	2/100	0.197972	0.408923	0.269513	0.556697	-0.28249	0.457533	C3;IFITM1
gliquidone-3126	2/100	0.197972	0.408923	0.269513	0.556697	-0.28142	0.455795	IFITM1;PHLDA1
heptaminol-1866	2/100	0.197972	0.408923	0.269513	0.556697	-0.28124	0.455501	TCN1;ENC1
haloperidol-5273	2/100	0.197972	0.408923	0.269513	0.556697	-0.27897	0.451836	COL1A1;AP1S1
hydrochlorothiazide-1487	2/100	0.197972	0.408923	0.269513	0.556697	-0.27783	0.449983	S100A6;FOS
haloperidol-6163	2/100	0.197972	0.408923	0.269513	0.556697	-0.27518	0.445694	COL1A1;TFF2
hexestrol-2515	2/100	0.197972	0.408923	0.269513	0.556697	-0.27507	0.445507	TFF2;DCN
gliclazide-1720	2/100	0.197972	0.408923	0.269513	0.556697	-0.27506	0.445496	QPCT;DSG2
H-89-6878	2/100	0.197972	0.408923	0.269513	0.556697	-0.27504	0.445471	IFI6;AP1S1
hesperetin-1947	2/100	0.197972	0.408923	0.269513	0.556697	-0.27477	0.445019	CD24;ITM2C
hydrocotarnine-2765	2/100	0.197972	0.408923	0.269513	0.556697	-0.2737	0.443289	AP1S1;TFF2
hexamethonium bromide-1982	2/100	0.197972	0.408923	0.269513	0.556697	-0.27366	0.443234	IFITM3;LY6E
haloperidol-4468	2/100	0.197972	0.408923	0.269513	0.556697	-0.27309	0.442308	IFITM1;JUND
imipramine-5440	2/100	0.197972	0.408923	0.269513	0.556697	-0.27226	0.44096	CEACAM6;SECTM1
hemicholinium-6739	2/100	0.197972	0.408923	0.269513	0.556697	-0.27194	0.440442	COL1A2;DCN
guanadrel-3698	2/100	0.197972	0.408923	0.269513	0.556697	-0.26736	0.433028	IL32;SLCO5A1
iloprost-496	2/100	0.197972	0.408923	0.269513	0.556697	-0.2664	0.431472	TPI1;PDZK1IP1
guanadrel-2575	2/100	0.197972	0.408923	0.269513	0.556697	-0.26554	0.430081	NQO1;IFITM1
hexestrol-6077	2/100	0.197972	0.408923	0.269513	0.556697	-0.26443	0.428284	FOS;PHLDA1
heliotrine-4739	2/100	0.197972	0.408923	0.269513	0.556697	-0.26329	0.426435	COL1A1;ITM2C
HNMPA-(AM)3-583	2/100	0.197972	0.408923	0.269513	0.556697	-0.26246	0.425091	JUND;COL1A2
hexylcaine-2708	2/100	0.197972	0.408923	0.269513	0.556697	-0.26215	0.424594	APP;DCN
hydrocortisone-6684	2/100	0.197972	0.408923	0.269513	0.556697	-0.26113	0.422929	COL1A2;OLFM4
haloperidol-2663	2/100	0.197972	0.408923	0.269513	0.556697	-0.25922	0.419841	NQO1;IFI6
isocorydine-6843	2/100	0.197972	0.408923	0.269513	0.556697	-0.2592	0.419812	IFI6;PHLDA1
hydroquinine-2767	2/100	0.197972	0.408923	0.269513	0.556697	-0.2588	0.419168	COL1A1;AP1S1
hydrastine hydrochloride-1740	2/100	0.197972	0.408923	0.269513	0.556697	-0.25866	0.418927	APP;GPX2
IC-86621-7513	2/100	0.197972	0.408923	0.269513	0.556697	-0.2574	0.416889	AP1S1;PHLDA1
iproniazid-2288	2/100	0.197972	0.408923	0.269513	0.556697	-0.25726	0.416673	RCN1;IFI27
idoxuridine-1980	2/100	0.197972	0.408923	0.269513	0.556697	-0.25646	0.415365	NQO1;FOS
homatropine-1848	2/100	0.197972	0.408923	0.269513	0.556697	-0.25388	0.41119	COL1A1;TCN1
isometheptene-5502	2/100	0.197972	0.408923	0.269513	0.556697	-0.25336	0.410356	CEACAM6;IFI6
heptaminol-1825	2/100	0.197972	0.408923	0.269513	0.556697	-0.25302	0.409798	AP1S1;ITM2B
hymecromone-4623	2/100	0.197972	0.408923	0.269513	0.556697	-0.25043	0.405603	COL1A1;SPARC
iopromide-2739	2/100	0.197972	0.408923	0.269513	0.556697	-0.24979	0.404576	COL1A1;CEACAM6
iocetamic acid-3022	2/100	0.197972	0.408923	0.269513	0.556697	-0.24919	0.403603	SLCO1B3;DCN
hydrocotarnine-1772	2/100	0.197972	0.408923	0.269513	0.556697	-0.24899	0.403279	IFITM3;MMP1

iohexol-2461	2/100	0.197972	0.408923	0.269513	0.556697	-0.24342	0.394256	CEACAM5;TFF2
harman-4584	2/100	0.197972	0.408923	0.269513	0.556697	-0.24298	0.393541	SPINK1;DCN
hydroflumethiazide-7259	2/100	0.197972	0.408923	0.269513	0.556697	-0.24173	0.391509	SERPINA1;SPP1
iopanoic acid-3527	2/100	0.197972	0.408923	0.269513	0.556697	-0.24163	0.391345	C3;SPARC
isradipine-6347	2/100	0.197972	0.408923	0.269513	0.556697	-0.23739	0.384483	IFI6;AP1S1
ionomycin-979	2/100	0.197972	0.408923	0.269513	0.556697	-0.23622	0.382582	PHLDA1;ETS2
idoxuridine-4200	2/100	0.197972	0.408923	0.269513	0.556697	-0.23619	0.382546	IFITM1;CEACAM6
hycanthone-5691	2/100	0.197972	0.408923	0.269513	0.556697	-0.23588	0.382043	PTPRO;AP1S1
hydralazine-4746	2/100	0.197972	0.408923	0.269513	0.556697	-0.23475	0.380209	IFI6;CTSE
levothyroxine sodium-1312	2/100	0.197972	0.408923	0.269513	0.556697	-0.23154	0.375009	TCN1;ATP1B1
imipramine-1807	2/100	0.197972	0.408923	0.269513	0.556697	-0.22962	0.371905	CCL20;AP1S1
isoxicam-1820	2/100	0.197972	0.408923	0.269513	0.556697	-0.22648	0.366809	COL1A1;AP1S1
ifenprodil-7404	2/100	0.197972	0.408923	0.269513	0.556697	-0.22542	0.365098	RNF43;IFITM1
levobunolol-3354	2/100	0.197972	0.408923	0.269513	0.556697	-0.21913	0.354916	TCN1;SECTM1
labetalol-6809	2/100	0.197972	0.408923	0.269513	0.556697	-0.21828	0.35354	SOX9;ITM2C
lansoprazole-6009	2/100	0.197972	0.408923	0.269513	0.556697	-0.21807	0.353188	COL1A1;CEACAM6
iopamidol-7189	2/100	0.197972	0.408923	0.269513	0.556697	-0.21756	0.352364	SOX9;ITM2B
ioversol-3365	2/100	0.197972	0.408923	0.269513	0.556697	-0.21719	0.351773	MMP3;IFI6
ketanserin-4995	2/100	0.197972	0.408923	0.269513	0.556697	-0.21717	0.351741	MMP3;SECTM1
isocarboxazid-3424	2/100	0.197972	0.408923	0.269513	0.556697	-0.21586	0.349609	CFI;MMP3
leflunomide-5884	2/100	0.197972	0.408923	0.269513	0.556697	-0.21584	0.349587	GPX2;TSPAN8
leflunomide-7238	2/100	0.197972	0.408923	0.269513	0.556697	-0.21483	0.347939	CEACAM5;IFI6
isoflupredone-1873	2/100	0.197972	0.408923	0.269513	0.556697	-0.21253	0.344221	CCL20;ACSL1
imipenem-5997	2/100	0.197972	0.408923	0.269513	0.556697	-0.21168	0.342836	CEACAM6;IFI6
ketotifen-1583	2/100	0.197972	0.408923	0.269513	0.556697	-0.21134	0.342293	IFITM1;IFI6
levamisole-7450	2/100	0.197972	0.408923	0.269513	0.556697	-0.21089	0.341556	ENC1;S100A4
iopamidol-5832	2/100	0.197972	0.408923	0.269513	0.556697	-0.21032	0.340643	IFITM1;IFI6
khellin-6641	2/100	0.197972	0.408923	0.269513	0.556697	-0.20918	0.338787	COL1A1;AP1S1
josamycin-2034	2/100	0.197972	0.408923	0.269513	0.556697	-0.20838	0.337496	AP1S1;CD24
irinotecan-7530	2/100	0.197972	0.408923	0.269513	0.556697	-0.20828	0.337342	ID3;ETS2
isocorydine-2780	2/100	0.197972	0.408923	0.269513	0.556697	-0.20769	0.336388	RCN1;AP1S1
melatonin-5393	2/100	0.197972	0.408923	0.269513	0.556697	-0.2073	0.335753	COL1A1;S100A11
isoniazid-2083	2/100	0.197972	0.408923	0.269513	0.556697	-0.20539	0.332648	IFI6;AP1S1
ioversol-5326	2/100	0.197972	0.408923	0.269513	0.556697	-0.20404	0.330476	CEACAM6;S100A4
labetalol-4473	2/100	0.197972	0.408923	0.269513	0.556697	-0.20232	0.327676	IFITM1;DCN
levamisole-2094	2/100	0.197972	0.408923	0.269513	0.556697	-0.20073	0.325107	CCL20;FOS
isocorydine-1787	2/100	0.197972	0.408923	0.269513	0.556697	-0.20022	0.324287	GPX2;AP1S1

loperamide-2033	2/100	0.197972	0.408923	0.269513	0.556697	-0.19928	0.322766	NQO1;CTSE
luteolin-3379	2/100	0.197972	0.408923	0.269513	0.556697	-0.19875	0.321906	IFI6;ETS2
lasalocid-3021	2/100	0.197972	0.408923	0.269513	0.556697	-0.19875	0.321896	FXYD3;SPP1
L-methionine sulfoximine-4070	2/100	0.197972	0.408923	0.269513	0.556697	-0.19858	0.321627	BMP4;APP
levomepromazine-4723	2/100	0.197972	0.408923	0.269513	0.556697	-0.19841	0.321355	SLPI;MMP3
kanamycin-1609	2/100	0.197972	0.408923	0.269513	0.556697	-0.19592	0.317319	TPI1;CCL20
ketoprofen-4751	2/100	0.197972	0.408923	0.269513	0.556697	-0.1958	0.317122	IFI27;IFI6
karakoline-3638	2/100	0.197972	0.408923	0.269513	0.556697	-0.19558	0.316764	CEACAM5;IFI6
isoflupredone-1832	2/100	0.197972	0.408923	0.269513	0.556697	-0.19353	0.313449	CCL20;AP1S1
lactobionic acid-3246	2/100	0.197972	0.408923	0.269513	0.556697	-0.19302	0.312619	COL1A1;TXNIP
L-methionine sulfoximine-2470	2/100	0.197972	0.408923	0.269513	0.556697	-0.19211	0.311146	MMP3;S100A11
LY-294002-5224	2/100	0.197972	0.408923	0.269513	0.556697	-0.19025	0.30813	TXNIP;TFF2
levobunolol-4134	2/100	0.197972	0.408923	0.269513	0.556697	-0.18958	0.307049	COL1A1;BMP4
lynestrenol-2037	2/100	0.197972	0.408923	0.269513	0.556697	-0.18954	0.306978	GPX2;CKB
lumicolchicine-3254	2/100	0.197972	0.408923	0.269513	0.556697	-0.18819	0.304804	C3;AP1S1
lidocaine-4421	2/100	0.197972	0.408923	0.269513	0.556697	-0.18716	0.303134	APP;SECTM1
medrysone-2544	2/100	0.197972	0.408923	0.269513	0.556697	-0.18483	0.29935	IFITM1;CCL20
lisuride-1962	2/100	0.197972	0.408923	0.269513	0.556697	-0.18443	0.298714	CEACAM6;PHLDA1
lorglumide-3079	2/100	0.197972	0.408923	0.269513	0.556697	-0.18362	0.297399	COL1A2;FXYD3
LY-294002-5937	2/100	0.197972	0.408923	0.269513	0.556697	-0.17798	0.288256	TXNIP;FOS
lovastatin-2854	2/100	0.197972	0.408923	0.269513	0.556697	-0.17756	0.287585	SPARC;FOS
maprotiline-5022	2/100	0.197972	0.408923	0.269513	0.556697	-0.17741	0.287338	SOX9;PHLDA1
LY-294002-6945	2/100	0.197972	0.408923	0.269513	0.556697	-0.17707	0.286782	IFI6;TXNIP
LY-294002-5587	2/100	0.197972	0.408923	0.269513	0.556697	-0.17671	0.286201	COL1A1;TXNIP
LY-294002-6935	2/100	0.197972	0.408923	0.269513	0.556697	-0.17664	0.286097	IFI6;S100A4
LY-294002-1065	2/100	0.197972	0.408923	0.269513	0.556697	-0.17598	0.285021	TXNIP;TGFBI
loracarbef-5073	2/100	0.197972	0.408923	0.269513	0.556697	-0.17226	0.278999	IL32;COL1A1
LY-294002-5233	2/100	0.197972	0.408923	0.269513	0.556697	-0.17084	0.276699	IFI6;TXNIP
methylergometrine-3222	2/100	0.197972	0.408923	0.269513	0.556697	-0.16882	0.27343	C3;DCN
loxapine-6694	2/100	0.197972	0.408923	0.269513	0.556697	-0.16772	0.271644	IL32;SLCO5A1
levobunolol-3015	2/100	0.197972	0.408923	0.269513	0.556697	-0.16762	0.271478	FXYD3;DCN
maprotiline-6676	2/100	0.197972	0.408923	0.269513	0.556697	-0.16647	0.269622	C3;DUOX2
levomepromazine-3440	2/100	0.197972	0.408923	0.269513	0.556697	-0.16596	0.268795	S100A4;S100A11
LY-294002-6956	2/100	0.197972	0.408923	0.269513	0.556697	-0.16419	0.265923	TXNIP;S100A11
levopropoxyphene-2980	2/100	0.197972	0.408923	0.269513	0.556697	-0.1635	0.264814	IFITM3;ATP1B1
melatonin-3293	2/100	0.197972	0.408923	0.269513	0.556697	-0.16285	0.26376	AP1S1;ITM2C
mafenide-2287	2/100	0.197972	0.408923	0.269513	0.556697	-0.15982	0.258847	SPINK1;DCN

mebendazole-2300	2/100	0.197972	0.408923	0.269513	0.556697	-0.15964	0.258555	PHLDA1;ETS2
metamizole sodium-3929	2/100	0.197972	0.408923	0.269513	0.556697	-0.1563	0.253155	C3;PDZK1IP1
mafenide-5079	2/100	0.197972	0.408923	0.269513	0.556697	-0.15626	0.25308	FABP1;CEACAM6
mesalazine-3584	2/100	0.197972	0.408923	0.269513	0.556697	-0.1561	0.252829	C3;TCN1
LY-294002-328	2/100	0.197972	0.408923	0.269513	0.556697	-0.15605	0.252744	TXNIP;ITM2B
lysergol-1325	2/100	0.197972	0.408923	0.269513	0.556697	-0.15489	0.250867	GPX2;IFI6
mefenamic acid-1821	2/100	0.197972	0.408923	0.269513	0.556697	-0.15407	0.249534	IL32;SERPINA1
mestranol-4208	2/100	0.197972	0.408923	0.269513	0.556697	-0.15379	0.249089	IFITM1;MMP3
metergoline-3221	2/100	0.197972	0.408923	0.269513	0.556697	-0.15352	0.248641	SOX9;DCN
LY-294002-1177	2/100	0.197972	0.408923	0.269513	0.556697	-0.15338	0.248413	CEACAM6;FOS
meclozine-5607	2/100	0.197972	0.408923	0.269513	0.556697	-0.15271	0.247328	CEACAM5;ITM2C
lycorine-2195	2/100	0.197972	0.408923	0.269513	0.556697	-0.15264	0.247226	CCL20;PHLDA1
methoxsalen-5007	2/100	0.197972	0.408923	0.269513	0.556697	-0.15111	0.244743	SPINK1;S100A4
mebhydrolin-4211	2/100	0.197972	0.408923	0.269513	0.556697	-0.15054	0.243814	BMP4;IFITM1
memantine-7354	2/100	0.197972	0.408923	0.269513	0.556697	-0.15052	0.243792	SOX9;PHLDA1
methylbenzethonium chloride-4325	2/100	0.197972	0.408923	0.269513	0.556697	-0.1501	0.243105	S100P;FOS
methazolamide-3474	2/100	0.197972	0.408923	0.269513	0.556697	-0.14889	0.241155	AP1S1;PHLDA1
metformin-1858	2/100	0.197972	0.408923	0.269513	0.556697	-0.14575	0.23606	APP;S100A11
methotrexate-1599	2/100	0.197972	0.408923	0.269513	0.556697	-0.14513	0.23505	IFITM3;AP1S1
meticrane-5984	2/100	0.197972	0.408923	0.269513	0.556697	-0.14505	0.234931	APP;TCN1
methoxsalen-3302	2/100	0.197972	0.408923	0.269513	0.556697	-0.14502	0.234872	AP1S1;S100A11
metixene-5018	2/100	0.197972	0.408923	0.269513	0.556697	-0.14469	0.234341	SOX9;PHLDA1
mebeverine-3193	2/100	0.197972	0.408923	0.269513	0.556697	-0.14372	0.232769	CTSE;PHLDA1
metampicillin-1440	2/100	0.197972	0.408923	0.269513	0.556697	-0.14257	0.230915	IFITM3;AP1S1
mafenide-1441	2/100	0.197972	0.408923	0.269513	0.556697	-0.1412	0.228687	AP1S1;PTMA
metolazone-1932	2/100	0.197972	0.408923	0.269513	0.556697	-0.1398	0.226428	PIGR;CEACAM6
methotrexate-5419	2/100	0.197972	0.408923	0.269513	0.556697	-0.13965	0.22618	PHLDA1;CTGF
LY-294002-6198	2/100	0.197972	0.408923	0.269513	0.556697	-0.13828	0.223966	TMPRSS3;TFF2
molindone-7337	2/100	0.197972	0.408923	0.269513	0.556697	-0.13629	0.220737	COL1A1;PHLDA1
meteneprost-7557	2/100	0.197972	0.408923	0.269513	0.556697	-0.13559	0.219606	APP;CEACAM6
monorden-5947	2/100	0.197972	0.408923	0.269513	0.556697	-0.13464	0.218075	RNF43;IFI27
metformin-5068	2/100	0.197972	0.408923	0.269513	0.556697	-0.12815	0.207564	COL1A1;PSMB9
mephentermine-2563	2/100	0.197972	0.408923	0.269513	0.556697	-0.12773	0.206877	COL1A2;CCL20
monensin-4726	2/100	0.197972	0.408923	0.269513	0.556697	-0.1261	0.20424	JUND;PHLDA1
methyldopa-5272	2/100	0.197972	0.408923	0.269513	0.556697	-0.12588	0.203884	COL1A2;PHLDA1
methylprednisolone-7137	2/100	0.197972	0.408923	0.269513	0.556697	-0.12128	0.196431	IFI6;TSPAN1

meglumine-5285	2/100	0.197972	0.408923	0.269513	0.556697	-0.12066	0.195424	TMPRSS3;IFI6
metixene-2451	2/100	0.197972	0.408923	0.269513	0.556697	-0.11918	0.193029	COL1A1;PHLDA1
metoclopramide-4750	2/100	0.197972	0.408923	0.269513	0.556697	-0.11907	0.192845	CEACAM5;IFI6
minaprine-4814	2/100	0.197972	0.408923	0.269513	0.556697	-0.11881	0.192428	CEACAM5;MMP3
mometasone-2896	2/100	0.197972	0.408923	0.269513	0.556697	-0.11869	0.192241	IL32;TXNIP
methyldopa-1619	2/100	0.197972	0.408923	0.269513	0.556697	-0.11809	0.191258	CCL20;PTMA
meropenem-6141	2/100	0.197972	0.408923	0.269513	0.556697	-0.11636	0.188454	CEACAM6;MMP3
mefloquine-5724	2/100	0.197972	0.408923	0.269513	0.556697	-0.115	0.186255	JUND;FOS
metixene-6672	2/100	0.197972	0.408923	0.269513	0.556697	-0.11482	0.185966	TFF3;DCN
methapyrilene-1588	2/100	0.197972	0.408923	0.269513	0.556697	-0.11301	0.183042	PLCB4;PTMA
molsidomine-5426	2/100	0.197972	0.408923	0.269513	0.556697	-0.11274	0.182591	SOX9;ITM2C
nafcillin-3983	2/100	0.197972	0.408923	0.269513	0.556697	-0.11259	0.182348	QPCT;SPP1
methazolamide-6268	2/100	0.197972	0.408923	0.269513	0.556697	-0.11137	0.180374	IFI6;ISG15
methylergometrine-5303	2/100	0.197972	0.408923	0.269513	0.556697	-0.11075	0.17938	AP1S1;S100A4
mevalolactone-5738	2/100	0.197972	0.408923	0.269513	0.556697	-0.10922	0.176903	COL1A1;TMPRSS3
metamizole sodium-2175	2/100	0.197972	0.408923	0.269513	0.556697	-0.10821	0.175255	IFITM1;TCN1
metoclopramide-3625	2/100	0.197972	0.408923	0.269513	0.556697	-0.10729	0.173771	FOS;DCN
mexiletine-3973	2/100	0.197972	0.408923	0.269513	0.556697	-0.10493	0.169945	COL1A1;DPEP1
methocarbamol-2274	2/100	0.197972	0.408923	0.269513	0.556697	-0.10279	0.166475	SERPINA1;SPP1
mevalolactone-3459	2/100	0.197972	0.408923	0.269513	0.556697	-0.10238	0.165819	RCN1;AP1S1
monastrol-605	2/100	0.197972	0.408923	0.269513	0.556697	-0.10039	0.162602	SLPI;CEACAM5
monastrol-627	2/100	0.197972	0.408923	0.269513	0.556697	-0.09994	0.161863	NQO1;AP1S1
mifepristone-1569	2/100	0.197972	0.408923	0.269513	0.556697	-0.09979	0.161619	APP;TPI1
milrinone-7210	2/100	0.197972	0.408923	0.269513	0.556697	-0.09855	0.159608	COL1A1;ITM2C
metrifonate-1839	2/100	0.197972	0.408923	0.269513	0.556697	-0.09843	0.159424	ENC1;ID3
monorden-1644	2/100	0.197972	0.408923	0.269513	0.556697	-0.09768	0.158199	SERPINA1;SOX9
minoxidil-4216	2/100	0.197972	0.408923	0.269513	0.556697	-0.09422	0.152598	JUND;LYZ
N-acetyl-L-aspartic acid-4007	2/100	0.197972	0.408923	0.269513	0.556697	-0.09182	0.148716	SERPINA1;CEACAM6
monorden-1057	2/100	0.197972	0.408923	0.269513	0.556697	-0.09031	0.146271	BMP4;TXNIP
monastrol-614	2/100	0.197972	0.408923	0.269513	0.556697	-0.08922	0.144508	IFITM3;S100A11
metitepine-5413	2/100	0.197972	0.408923	0.269513	0.556697	-0.08902	0.144175	CEACAM6;FOS
monensin-2580	2/100	0.197972	0.408923	0.269513	0.556697	-0.08434	0.136602	CTSE;PHLDA1
nicergoline-2220	2/100	0.197972	0.408923	0.269513	0.556697	-0.0832	0.134761	C3;TFF2
myosmine-4759	2/100	0.197972	0.408923	0.269513	0.556697	-0.08205	0.132896	COL1A1;DCN
naltrexone-2209	2/100	0.197972	0.408923	0.269513	0.556697	-0.0816	0.132157	COL1A1;CEACAM5
monorden-493	2/100	0.197972	0.408923	0.269513	0.556697	-0.07897	0.1279	HSPB1;ITM2B
N6-methyladenosine-5332	2/100	0.197972	0.408923	0.269513	0.556697	-0.0785	0.127139	CEACAM6;TXNIP

monorden-1219	2/100	0.197972	0.408923	0.269513	0.556697	-0.07809	0.126478	HSPB1;CTGF
napelline-6824	2/100	0.197972	0.408923	0.269513	0.556697	-0.07659	0.124048	COL1A1;PDZK1IP1
monorden-1160	2/100	0.197972	0.408923	0.269513	0.556697	-0.0765	0.123908	HSP90AB1;HSPB1
minocycline-5496	2/100	0.197972	0.408923	0.269513	0.556697	-0.07626	0.123511	PTPRO;DCN
nisoxetine-5516	2/100	0.197972	0.408923	0.269513	0.556697	-0.07327	0.118667	IFITM1;TFF2
midecamycin-6745	2/100	0.197972	0.408923	0.269513	0.556697	-0.07249	0.117407	PIGR;PROM1
mitoxantrone-3232	2/100	0.197972	0.408923	0.269513	0.556697	-0.07237	0.117207	SOX9;CTGF
minoxidil-1914	2/100	0.197972	0.408923	0.269513	0.556697	-0.07093	0.11488	GPX2;TGFBI
oxedrine-6798	2/100	0.197972	0.408923	0.269513	0.556697	-0.07058	0.114309	CEACAM6;PHLDA1
nabumetone-6487	2/100	0.197972	0.408923	0.269513	0.556697	-0.07034	0.11393	CEACAM5;OLFM4
nicergoline-6251	2/100	0.197972	0.408923	0.269513	0.556697	-0.07002	0.113405	MMP3;LYZ
moxonidine-4084	2/100	0.197972	0.408923	0.269513	0.556697	-0.06693	0.108395	IL32;BMP4
nicardipine-1600	2/100	0.197972	0.408923	0.269513	0.556697	-0.06666	0.107961	APP;CCL20
natamycin-7167	2/100	0.197972	0.408923	0.269513	0.556697	-0.06607	0.107011	TFF2;ETS2
monastrol-668	2/100	0.197972	0.408923	0.269513	0.556697	-0.06475	0.104875	TSPAN8;DCN
metronidazole-4023	2/100	0.197972	0.408923	0.269513	0.556697	-0.06396	0.103588	COL1A2;FOS
nicardipine-5397	2/100	0.197972	0.408923	0.269513	0.556697	-0.0618	0.1001	APP;IFI6
N-acetylmuramic acid-4582	2/100	0.197972	0.408923	0.269513	0.556697	-0.06166	0.099864	CEACAM5;AP1S1
nefopam-4752	2/100	0.197972	0.408923	0.269513	0.556697	-0.06142	0.099472	COL1A1;CEACAM5
netilmicin-2963	2/100	0.197972	0.408923	0.269513	0.556697	-0.06121	0.099145	IGFBP2;ATP1B1
monorden-999	2/100	0.197972	0.408923	0.269513	0.556697	-0.06043	0.097873	APP;GPX2
monorden-2679	2/100	0.197972	0.408923	0.269513	0.556697	-0.05934	0.09611	APP;HSPB1
naloxone-2006	2/100	0.197972	0.408923	0.269513	0.556697	-0.05915	0.095796	COL1A1;GPX2
nalbuphine-1379	2/100	0.197972	0.408923	0.269513	0.556697	-0.05872	0.095104	ENC1;LY6E
nomifensine-7217	2/100	0.197972	0.408923	0.269513	0.556697	-0.05782	0.093644	IFITM1;IFI6
orlistat-6383	2/100	0.197972	0.408923	0.269513	0.556697	-0.05725	0.092718	MMP3;PHLDA1
nystatin-4223	2/100	0.197972	0.408923	0.269513	0.556697	-0.05649	0.091491	IFITM1;JUND
nicergoline-2058	2/100	0.197972	0.408923	0.269513	0.556697	-0.05632	0.091213	TSPAN8;CCL20
monorden-6178	2/100	0.197972	0.408923	0.269513	0.556697	-0.05573	0.090254	HSPB1;TGFBI
norfloxacin-2090	2/100	0.197972	0.408923	0.269513	0.556697	-0.05566	0.090142	RNF43;APP
nifedipine-1856	2/100	0.197972	0.408923	0.269513	0.556697	-0.05488	0.088893	CEACAM5;S100A11
naphazoline-1966	2/100	0.197972	0.408923	0.269513	0.556697	-0.05478	0.08872	S100A6;CKB
naloxone-5243	2/100	0.197972	0.408923	0.269513	0.556697	-0.0532	0.086156	AP1S1;PHLDA1
naproxen-1828	2/100	0.197972	0.408923	0.269513	0.556697	-0.053	0.085835	AP1S1;PSMB9
nifenazone-2122	2/100	0.197972	0.408923	0.269513	0.556697	-0.05052	0.081822	JUND;TSPAN8
nortriptyline-7300	2/100	0.197972	0.408923	0.269513	0.556697	-0.05017	0.081249	JUND;DCN
pargyline-7016	2/100	0.197972	0.408923	0.269513	0.556697	-0.04801	0.077757	IFI27;IFI6

norethisterone-5055	2/100	0.197972	0.408923	0.269513	0.556697	-0.04764	0.077153	IFITM1;IFI6
naproxen-1706	2/100	0.197972	0.408923	0.269513	0.556697	-0.04673	0.075686	BMP4;AP1S1
noscapine-5851	2/100	0.197972	0.408923	0.269513	0.556697	-0.04488	0.072697	PIGR;DCN
norfloxacin-2253	2/100	0.197972	0.408923	0.269513	0.556697	-0.04481	0.072574	PROM1;LYZ
nimesulide-2112	2/100	0.197972	0.408923	0.269513	0.556697	-0.0415	0.067209	APP;CDH3
nifuroxazide-4835	2/100	0.197972	0.408923	0.269513	0.556697	-0.04	0.064781	COL1A1;IFI6
ofloxacin-2340	2/100	0.197972	0.408923	0.269513	0.556697	-0.03697	0.059885	FXYD3;S100A6
nizatidine-3047	2/100	0.197972	0.408923	0.269513	0.556697	-0.0352	0.057003	COL1A2;TCN1
nilutamide-6763	2/100	0.197972	0.408923	0.269513	0.556697	-0.0349	0.056517	PIGR;SPARC
orphenadrine-3801	2/100	0.197972	0.408923	0.269513	0.556697	-0.03313	0.05365	JUND;LUM
nordihydroguaiaretic acid-4447	2/100	0.197972	0.408923	0.269513	0.556697	-0.03231	0.052338	TFF3;PROM1
naftifine-2974	2/100	0.197972	0.408923	0.269513	0.556697	-0.02823	0.04572	IGFBP2;ATP1B1
nifuroxazide-4253	2/100	0.197972	0.408923	0.269513	0.556697	-0.02803	0.045395	SPP1;SLCO5A1
nifenazone-6016	2/100	0.197972	0.408923	0.269513	0.556697	-0.0278	0.045029	CEACAM5;ITM2C
nifedipine-7303	2/100	0.197972	0.408923	0.269513	0.556697	-0.02748	0.044507	COL1A1;APP
nortriptyline-2391	2/100	0.197972	0.408923	0.269513	0.556697	-0.02596	0.042043	IFITM1;CD24
niclosamide-1498	2/100	0.197972	0.408923	0.269513	0.556697	-0.02571	0.041636	IFI6;S100P
niclosamide-1916	2/100	0.197972	0.408923	0.269513	0.556697	-0.0256	0.041462	PTPRO;ETS2
nilutamide-6481	2/100	0.197972	0.408923	0.269513	0.556697	-0.02511	0.040663	MMP3;PROM1
oxamniquine-7344	2/100	0.197972	0.408923	0.269513	0.556697	-0.0249	0.040321	S100A4;S100A11
oxybutynin-7126	2/100	0.197972	0.408923	0.269513	0.556697	-0.0245	0.039682	GPX2;IFI6
nomegestrol-6362	2/100	0.197972	0.408923	0.269513	0.556697	-0.02337	0.037848	COL1A1;AP1S1
oxolamine-4969	2/100	0.197972	0.408923	0.269513	0.556697	-0.02296	0.037188	BMP4;SPINK1
nystatin-2500	2/100	0.197972	0.408923	0.269513	0.556697	-0.02047	0.033161	SPP1;PDZK1IP1
nordihydroguaiaretic acid-6942	2/100	0.197972	0.408923	0.269513	0.556697	-0.01902	0.030806	IFI6;TSPAN1
norfloxacin-5985	2/100	0.197972	0.408923	0.269513	0.556697	-0.01813	0.029366	APP;SLPI
nifuroxazide-2850	2/100	0.197972	0.408923	0.269513	0.556697	-0.01709	0.027671	TXNIP;CTGF
oxaprozin-971	2/100	0.197972	0.408923	0.269513	0.556697	-0.01578	0.025564	TXNIP;FOS
nipecotic acid-7296	2/100	0.197972	0.408923	0.269513	0.556697	-0.01255	0.020334	SERPINA1;ENC1
pentetrazol-5508	2/100	0.197972	0.408923	0.269513	0.556697	-0.01121	0.018155	IFITM1;IFI6
orciprenaline-4831	2/100	0.197972	0.408923	0.269513	0.556697	-0.01041	0.016864	IFI27;IFI6
orciprenaline-4248	2/100	0.197972	0.408923	0.269513	0.556697	-0.00918	0.014872	TFF1;S100P
nocodazole-1393	2/100	0.197972	0.408923	0.269513	0.556697	-0.00856	0.01386	ANXA2;CCL20
oxedrine-3578	2/100	0.197972	0.408923	0.269513	0.556697	-0.00683	0.011061	PTPRO;FOS
paromomycin-4595	2/100	0.197972	0.408923	0.269513	0.556697	-0.00435	0.007049	IFITM1;IFI6
nimodipine-5421	2/100	0.197972	0.408923	0.269513	0.556697	-0.00146	0.002364	S100A11;ITM2C
orphenadrine-4359	2/100	0.197972	0.408923	0.269513	0.556697	-0.00144	0.002339	IFI27;IFI6

oxolinic acid-5519	2/100	0.197972	0.408923	0.269513	0.556697	-0.00073	0.001185	IFITM1;IFI6
ornidazole-5483	2/100	0.197972	0.408923	0.269513	0.556697	-0.00046	0.000743	CEACAM5;ITM2C
parbendazole-4535	2/100	0.197972	0.408923	0.269513	0.556697	0.001075	-0.00174	OLFM4;DUOX2
ozagrel-5983	2/100	0.197972	0.408923	0.269513	0.556697	0.005245	-0.0085	APP;SECTM1
parthenolide-5105	2/100	0.197972	0.408923	0.269513	0.556697	0.005854	-0.00948	CEACAM5;FOS
phenelzine-3802	2/100	0.197972	0.408923	0.269513	0.556697	0.009593	-0.01554	PIGR;TMPRSS3
PF-00875133-00-5967	2/100	0.197972	0.408923	0.269513	0.556697	0.011132	-0.01803	TCN1;PHLDA1
ornidazole-2109	2/100	0.197972	0.408923	0.269513	0.556697	0.011767	-0.01906	CCL20;AP1S1
orphenadrine-3883	2/100	0.197972	0.408923	0.269513	0.556697	0.012379	-0.02005	PDZK1IP1;S100A11
PF-00539745-00-5974	2/100	0.197972	0.408923	0.269513	0.556697	0.012868	-0.02084	SLPI;TFF2
pergolide-2403	2/100	0.197972	0.408923	0.269513	0.556697	0.013167	-0.02133	CCL20;TIMP1
paclitaxel-5320	2/100	0.197972	0.408923	0.269513	0.556697	0.014316	-0.02319	IFITM1;CEACAM6
PF-01378883-00-6363	2/100	0.197972	0.408923	0.269513	0.556697	0.01471	-0.02382	GPX2;CTGF
PF-00875133-00-5928	2/100	0.197972	0.408923	0.269513	0.556697	0.015387	-0.02492	C3;IFI6
NU-1025-608	2/100	0.197972	0.408923	0.269513	0.556697	0.016527	-0.02677	SLPI;CEACAM5
pentolonium-3676	2/100	0.197972	0.408923	0.269513	0.556697	0.017547	-0.02842	IL32;PSMB9
ondansetron-6270	2/100	0.197972	0.408923	0.269513	0.556697	0.018054	-0.02924	DCN;ETS2
oxolinic acid-2103	2/100	0.197972	0.408923	0.269513	0.556697	0.018934	-0.03067	JUND;TCN1
pentolonium-2305	2/100	0.197972	0.408923	0.269513	0.556697	0.019697	-0.0319	PIGR;SECTM1
PHA-00745360-3824	2/100	0.197972	0.408923	0.269513	0.556697	0.021275	-0.03446	CEACAM5;TMPRSS3
penbutolol-3534	2/100	0.197972	0.408923	0.269513	0.556697	0.026576	-0.04304	AP1S1;S100A11
pentetrazol-2092	2/100	0.197972	0.408923	0.269513	0.556697	0.028054	-0.04544	JUND;IFI6
paroxetine-3904	2/100	0.197972	0.408923	0.269513	0.556697	0.030967	-0.05016	PIGR;CTSE
phenformin-2350	2/100	0.197972	0.408923	0.269513	0.556697	0.031377	-0.05082	AP1S1;CD24
pivmecillinam-2973	2/100	0.197972	0.408923	0.269513	0.556697	0.031504	-0.05102	CEACAM6;ATP1B1
PF-00875133-00-5923	2/100	0.197972	0.408923	0.269513	0.556697	0.035839	-0.05805	SECTM1;SOX9
parthenolide-2885	2/100	0.197972	0.408923	0.269513	0.556697	0.035862	-0.05808	ID1;CTGF
phenformin-2312	2/100	0.197972	0.408923	0.269513	0.556697	0.036108	-0.05848	PTPRO;IFI6
PNU-0251126-3689	2/100	0.197972	0.408923	0.269513	0.556697	0.037377	-0.06054	COL1A2;PDZK1IP1
perhexiline-7441	2/100	0.197972	0.408923	0.269513	0.556697	0.038481	-0.06233	FOS;PHLDA1
prednisolone-2393	2/100	0.197972	0.408923	0.269513	0.556697	0.038807	-0.06285	COL1A1;CCL20
pralidoxime-5383	2/100	0.197972	0.408923	0.269513	0.556697	0.039325	-0.06369	C3;APP
picrotoxinin-2161	2/100	0.197972	0.408923	0.269513	0.556697	0.039991	-0.06477	IFI6;ID3
phenazopyridine-2537	2/100	0.197972	0.408923	0.269513	0.556697	0.041684	-0.06751	CD24;DCN
phentolamine-1138	2/100	0.197972	0.408923	0.269513	0.556697	0.04381	-0.07096	ID3;ITM2B
PHA-00851261E-4330	2/100	0.197972	0.408923	0.269513	0.556697	0.044114	-0.07145	RNF43;TMPRSS3
piracetam-5462	2/100	0.197972	0.408923	0.269513	0.556697	0.045207	-0.07322	TFF2;S100A4

PHA-00767505E-6596	2/100	0.197972	0.408923	0.269513	0.556697	0.04613	-0.07471	RCN1;AP1S1
oxymetazoline-2114	2/100	0.197972	0.408923	0.269513	0.556697	0.04722	-0.07648	RNF43;AP1S1
pepstatin-3264	2/100	0.197972	0.408923	0.269513	0.556697	0.048216	-0.07809	IFI27;IFI6
PF-00539758-00-6379	2/100	0.197972	0.408923	0.269513	0.556697	0.050687	-0.08209	COL1A1;FOS
perhexiline-2410	2/100	0.197972	0.408923	0.269513	0.556697	0.050928	-0.08248	SPP1;DCN
proxyphylline-5993	2/100	0.197972	0.408923	0.269513	0.556697	0.052475	-0.08499	SLPI;ITM2C
PHA-00767505E-6591	2/100	0.197972	0.408923	0.269513	0.556697	0.052665	-0.0853	GAPDH;ACTB
pipenzolate bromide-2719	2/100	0.197972	0.408923	0.269513	0.556697	0.052704	-0.08536	SPARC;CEACAM5
PF-00539758-00-6416	2/100	0.197972	0.408923	0.269513	0.556697	0.053475	-0.08661	COL1A1;C3
pimozide-3178	2/100	0.197972	0.408923	0.269513	0.556697	0.053651	-0.0869	FOS;IER2
pentetic acid-3049	2/100	0.197972	0.408923	0.269513	0.556697	0.053821	-0.08717	FXYD3;CD24
prazosin-826	2/100	0.197972	0.408923	0.269513	0.556697	0.053998	-0.08746	COL1A1;QPCT
PHA-00665752-6578	2/100	0.197972	0.408923	0.269513	0.556697	0.054343	-0.08802	RNF43;PHLDA1
pridinol-7214	2/100	0.197972	0.408923	0.269513	0.556697	0.054391	-0.08809	TMPRSS3;ITM2C
prednicarbate-5544	2/100	0.197972	0.408923	0.269513	0.556697	0.055856	-0.09047	PIGR;SPARC
pinacidil-5456	2/100	0.197972	0.408923	0.269513	0.556697	0.056026	-0.09074	SOX9;PDZK1IP1
prochlorperazine-2641	2/100	0.197972	0.408923	0.269513	0.556697	0.056753	-0.09192	C3;IFI6
pargyline-1418	2/100	0.197972	0.408923	0.269513	0.556697	0.058248	-0.09434	CKB;PTMA
PNU-0230031-3632	2/100	0.197972	0.408923	0.269513	0.556697	0.059826	-0.0969	MMP3;IFI6
phentolamine-3860	2/100	0.197972	0.408923	0.269513	0.556697	0.061623	-0.09981	IFITM1;IFI6
profenamine-5296	2/100	0.197972	0.408923	0.269513	0.556697	0.063003	-0.10204	QPCT;S100A4
perphenazine-2040	2/100	0.197972	0.408923	0.269513	0.556697	0.063992	-0.10364	AP1S1;FOS
piperacillin-3763	2/100	0.197972	0.408923	0.269513	0.556697	0.064669	-0.10474	IL32;C3
piretanide-6144	2/100	0.197972	0.408923	0.269513	0.556697	0.065105	-0.10545	PIGR;ID1
PF-00539745-00-5939	2/100	0.197972	0.408923	0.269513	0.556697	0.06541	-0.10594	APP;IFI6
phentolamine-3971	2/100	0.197972	0.408923	0.269513	0.556697	0.065955	-0.10682	IFI27;IFI6
Prestwick-967-4250	2/100	0.197972	0.408923	0.269513	0.556697	0.068944	-0.11166	MMP3;ENC1
Prestwick-1085-6131	2/100	0.197972	0.408923	0.269513	0.556697	0.069058	-0.11185	COL1A2;PHLDA1
Prestwick-674-2179	2/100	0.197972	0.408923	0.269513	0.556697	0.070055	-0.11346	NQO1;COL1A2
phenformin-3622	2/100	0.197972	0.408923	0.269513	0.556697	0.070813	-0.11469	IFI6;PSMB9
Prestwick-1080-3878	2/100	0.197972	0.408923	0.269513	0.556697	0.071183	-0.11529	COL1A1;ITM2B
PF-01378883-00-6368	2/100	0.197972	0.408923	0.269513	0.556697	0.071459	-0.11574	TGFBI;DCN
pindolol-6834	2/100	0.197972	0.408923	0.269513	0.556697	0.074937	-0.12137	SOX9;ITM2C
PNU-0230031-4754	2/100	0.197972	0.408923	0.269513	0.556697	0.078919	-0.12782	COL1A1;OLFM4
physostigmine-1776	2/100	0.197972	0.408923	0.269513	0.556697	0.079494	-0.12875	LGALS3BP;AP1S1
phthalylsulfathiazole-5249	2/100	0.197972	0.408923	0.269513	0.556697	0.08046	-0.13032	SPINK1;PHLDA1
Prestwick-691-4092	2/100	0.197972	0.408923	0.269513	0.556697	0.081728	-0.13237	APP;TSPAN8

pronetalol-7322	2/100	0.197972	0.408923	0.269513	0.556697	0.083247	-0.13483	SERPINA1;SECTM1
Prestwick-920-5056	2/100	0.197972	0.408923	0.269513	0.556697	0.084129	-0.13626	RNF43;COL1A2
procaine-1674	2/100	0.197972	0.408923	0.269513	0.556697	0.087375	-0.14152	C3;PHLDA1
prilocaine-2352	2/100	0.197972	0.408923	0.269513	0.556697	0.087556	-0.14181	GPX2;AP1S1
quercetin-4846	2/100	0.197972	0.408923	0.269513	0.556697	0.089052	-0.14423	PIGR;IFI6
pronetalol-4104	2/100	0.197972	0.408923	0.269513	0.556697	0.091691	-0.14851	SPINK1;SECTM1
promethazine-5317	2/100	0.197972	0.408923	0.269513	0.556697	0.091939	-0.14891	IFITM1;IFI6
Prestwick-1100-3880	2/100	0.197972	0.408923	0.269513	0.556697	0.092465	-0.14976	IFI27;CTGF
progesterone-6646	2/100	0.197972	0.408923	0.269513	0.556697	0.095331	-0.1544	PDZK1IP1;DCN
prochlorperazine-995	2/100	0.197972	0.408923	0.269513	0.556697	0.095339	-0.15441	SOX9;FOS
podophyllotoxin-5841	2/100	0.197972	0.408923	0.269513	0.556697	0.095666	-0.15494	ETS2;CTGF
pivmecillinam-6014	2/100	0.197972	0.408923	0.269513	0.556697	0.096093	-0.15563	C3;TFF2
prochlorperazine-6975	2/100	0.197972	0.408923	0.269513	0.556697	0.097677	-0.1582	FOS;PHLDA1
prazosin-3098	2/100	0.197972	0.408923	0.269513	0.556697	0.099631	-0.16137	COL1A1;SPARC
ramipril-3572	2/100	0.197972	0.408923	0.269513	0.556697	0.100401	-0.16261	SPINK1;AP1S1
resveratrol-5084	2/100	0.197972	0.408923	0.269513	0.556697	0.100475	-0.16273	COL1A1;CEACAM6
promazine-3752	2/100	0.197972	0.408923	0.269513	0.556697	0.10268	-0.1663	PIGR;SLCO5A1
ramifenazone-7233	2/100	0.197972	0.408923	0.269513	0.556697	0.10326	-0.16724	SOX9;TSPAN1
progesterone-2426	2/100	0.197972	0.408923	0.269513	0.556697	0.104117	-0.16863	CEACAM5;TFF2
remoxipride-6342	2/100	0.197972	0.408923	0.269513	0.556697	0.104406	-0.1691	MMP3;DUOX2
Prestwick-983-3141	2/100	0.197972	0.408923	0.269513	0.556697	0.104516	-0.16928	ID3;TIMP1
Prestwick-920-5475	2/100	0.197972	0.408923	0.269513	0.556697	0.105248	-0.17046	IFI6;SOX9
pyrantel-2260	2/100	0.197972	0.408923	0.269513	0.556697	0.107765	-0.17454	PIGR;ITM2C
picotamide-2233	2/100	0.197972	0.408923	0.269513	0.556697	0.109475	-0.17731	COL1A1;CEACAM5
Prestwick-864-3333	2/100	0.197972	0.408923	0.269513	0.556697	0.109859	-0.17793	COL1A1;CTSE
PHA-00767505E-6550	2/100	0.197972	0.408923	0.269513	0.556697	0.110962	-0.17972	IFITM1;SERPINA1
pridinol-3456	2/100	0.197972	0.408923	0.269513	0.556697	0.111097	-0.17994	COL1A1;TMPRSS3
quinpirole-3539	2/100	0.197972	0.408923	0.269513	0.556697	0.11275	-0.18261	CXCL1;DCN
primaquine-3279	2/100	0.197972	0.408923	0.269513	0.556697	0.114216	-0.18499	AP1S1;CTGF
ritodrine-4619	2/100	0.197972	0.408923	0.269513	0.556697	0.116478	-0.18865	SPARC;S100P
seneciphylline-4822	2/100	0.197972	0.408923	0.269513	0.556697	0.117484	-0.19028	CEACAM5;IFI6
racecadotril-2774	2/100	0.197972	0.408923	0.269513	0.556697	0.118461	-0.19186	SLPI;AP1S1
pyrantel-5513	2/100	0.197972	0.408923	0.269513	0.556697	0.118769	-0.19236	COL1A1;IFITM1
sirolimus-5581	2/100	0.197972	0.408923	0.269513	0.556697	0.118894	-0.19256	COL1A1;TXNIP
R-atenolol-4841	2/100	0.197972	0.408923	0.269513	0.556697	0.119662	-0.19381	COL1A1;DUOX2
rifabutin-3873	2/100	0.197972	0.408923	0.269513	0.556697	0.120525	-0.19521	ITM2C;PSMB9
repaglinide-5862	2/100	0.197972	0.408923	0.269513	0.556697	0.121199	-0.1963	AP1S1;DCN

proadifen-2707	2/100	0.197972	0.408923	0.269513	0.556697	0.12264	-0.19863	COL1A1;CTGF
rifabutin-4527	2/100	0.197972	0.408923	0.269513	0.556697	0.12325	-0.19962	BMP4;PSMB9
reserpine-3341	2/100	0.197972	0.408923	0.269513	0.556697	0.124088	-0.20098	TXNIP;PHLDA1
quercetin-2499	2/100	0.197972	0.408923	0.269513	0.556697	0.126207	-0.20441	CCL20;SPP1
prochlorperazine-6174	2/100	0.197972	0.408923	0.269513	0.556697	0.127106	-0.20586	COL1A1;GPX2
pyrvinium-870	2/100	0.197972	0.408923	0.269513	0.556697	0.129625	-0.20995	JUND;MMP1
S-propranolol-3523	2/100	0.197972	0.408923	0.269513	0.556697	0.129768	-0.21018	C3;SERPINA1
quipazine-5887	2/100	0.197972	0.408923	0.269513	0.556697	0.131	-0.21217	TSPAN8;AP1S1
S-propranolol-2961	2/100	0.197972	0.408923	0.269513	0.556697	0.132413	-0.21446	TCN1;PHLDA1
propoxycaine-6803	2/100	0.197972	0.408923	0.269513	0.556697	0.13246	-0.21454	C3;CEACAM6
securinine-3470	2/100	0.197972	0.408923	0.269513	0.556697	0.132543	-0.21467	CEACAM5;SOX9
saquinavir-6246	2/100	0.197972	0.408923	0.269513	0.556697	0.132567	-0.21471	APP;AP1S1
propylthiouracil-2476	2/100	0.197972	0.408923	0.269513	0.556697	0.132834	-0.21514	GPX2;LY6E
quinethazone-4351	2/100	0.197972	0.408923	0.269513	0.556697	0.134473	-0.2178	C3;CEACAM5
solasodine-4305	2/100	0.197972	0.408923	0.269513	0.556697	0.135279	-0.2191	JUND;DCN
salsolidin-4810	2/100	0.197972	0.408923	0.269513	0.556697	0.135763	-0.21989	COL1A1;CTSE
ribavirin-6018	2/100	0.197972	0.408923	0.269513	0.556697	0.137555	-0.22279	APP;TFF2
sirolimus-1632	2/100	0.197972	0.408923	0.269513	0.556697	0.138072	-0.22363	ID1;TXNIP
proscillaridin-2920	2/100	0.197972	0.408923	0.269513	0.556697	0.138585	-0.22446	TXNIP;FOS
pyrimethamine-1474	2/100	0.197972	0.408923	0.269513	0.556697	0.141748	-0.22958	IFITM1;IFI6
riboflavin-2760	2/100	0.197972	0.408923	0.269513	0.556697	0.146033	-0.23652	AP1S1;SECTM1
ronidazole-3557	2/100	0.197972	0.408923	0.269513	0.556697	0.146986	-0.23806	COL1A1;MMP3
serotonin-5633	2/100	0.197972	0.408923	0.269513	0.556697	0.147359	-0.23867	IFI27;IFI6
stachydrine-6805	2/100	0.197972	0.408923	0.269513	0.556697	0.14742	-0.23877	TMPRSS3;PHLDA1
roxarsone-3511	2/100	0.197972	0.408923	0.269513	0.556697	0.147736	-0.23928	SERPINA1;TFF2
pyridoxine-1759	2/100	0.197972	0.408923	0.269513	0.556697	0.148146	-0.23994	IFITM3;APP
sirolimus-5204	2/100	0.197972	0.408923	0.269513	0.556697	0.148267	-0.24014	COL1A1;TXNIP
ribostamycin-6765	2/100	0.197972	0.408923	0.269513	0.556697	0.151372	-0.24517	SECTM1;PHLDA1
sirolimus-6180	2/100	0.197972	0.408923	0.269513	0.556697	0.151803	-0.24587	SPINK1;AP1S1
ribavirin-7316	2/100	0.197972	0.408923	0.269513	0.556697	0.152756	-0.24741	JUND;IFI6
roxarsone-2950	2/100	0.197972	0.408923	0.269513	0.556697	0.153152	-0.24805	COL1A1;PDZK1IP1
rolitetracycline-3031	2/100	0.197972	0.408923	0.269513	0.556697	0.153572	-0.24873	NQO1;SECTM1
salbutamol-3677	2/100	0.197972	0.408923	0.269513	0.556697	0.153633	-0.24883	C3;S100P
rosiglitazone-1071	2/100	0.197972	0.408923	0.269513	0.556697	0.155886	-0.25248	APP;S100A4
(-)-isoprenaline-6833	1/100	0.561955	0.561955	0.637173	0.637173	0.440088	-0.25364	IL32
sirolimus-5239	2/100	0.197972	0.408923	0.269513	0.556697	0.156946	-0.25419	IFI6;TXNIP
scopolamine-4219	2/100	0.197972	0.408923	0.269513	0.556697	0.157816	-0.2556	JUND;LUM

SB-202190-6882	2/100	0.197972	0.408923	0.269513	0.556697	0.15863	-0.25692	CEACAM6;AP1S1
0316684-0000-7057	1/100	0.561955	0.561955	0.637173	0.637173	0.448032	-0.25822	APP
securinine-4493	2/100	0.197972	0.408923	0.269513	0.556697	0.161422	-0.26144	NQO1;JUND
0173570-0000-7389	1/100	0.561955	0.561955	0.637173	0.637173	0.458664	-0.26434	AP1S1
2,6-dimethylpiperidine-3806	1/100	0.561955	0.561955	0.637173	0.637173	0.459249	-0.26468	IL32
securinine-6831	2/100	0.197972	0.408923	0.269513	0.556697	0.163671	-0.26509	SOX9;PHLDA1
0317956-0000-3966	1/100	0.561955	0.561955	0.637173	0.637173	0.461295	-0.26586	COL1A1
11-deoxy-16,16-dimethylprostaglandin E2-7519	1/100	0.561955	0.561955	0.637173	0.637173	0.465896	-0.26851	S100P
quinisocaine-2151	2/100	0.197972	0.408923	0.269513	0.556697	0.166802	-0.27016	IFI6;PHLDA1
1,5-isoquinolinediol-543	1/100	0.561955	0.561955	0.637173	0.637173	0.469471	-0.27057	NQO1
0179445-0000-4292	1/100	0.561955	0.561955	0.637173	0.637173	0.470592	-0.27122	TSPAN1
scoulerine-1742	2/100	0.197972	0.408923	0.269513	0.556697	0.168069	-0.27221	QPCT;PHLDA1
stachydrine-1751	2/100	0.197972	0.408923	0.269513	0.556697	0.168217	-0.27245	IFITM3;PTMA
0175029-0000-3691	1/100	0.561955	0.561955	0.637173	0.637173	0.473495	-0.27289	COL1A1
11-deoxy-16,16-dimethylprostaglandin E2-7538	1/100	0.561955	0.561955	0.637173	0.637173	0.473911	-0.27313	AP1S1
4,5-dianilinophthalimide-578	1/100	0.561955	0.561955	0.637173	0.637173	0.475957	-0.27431	CDH3
(+)-isoprenaline-5009	1/100	0.561955	0.561955	0.637173	0.637173	0.475963	-0.27431	IFI27
15-delta prostaglandin J2-1069	1/100	0.561955	0.561955	0.637173	0.637173	0.478128	-0.27556	ETS2
succinylsulfathiazole-2821	2/100	0.197972	0.408923	0.269513	0.556697	0.17091	-0.27681	COL1A1;PIGR
sulconazole-4998	2/100	0.197972	0.408923	0.269513	0.556697	0.171066	-0.27706	SOX9;FOS
16,16-dimethylprostaglandin E2-6592	1/100	0.561955	0.561955	0.637173	0.637173	0.482518	-0.27809	S100P
(+)-isoprenaline-3384	1/100	0.561955	0.561955	0.637173	0.637173	0.484753	-0.27938	CTGF
3-hydroxy-DL-kynurenine-5641	1/100	0.561955	0.561955	0.637173	0.637173	0.485509	-0.27982	SPP1
(-)-MK-801-3081	1/100	0.561955	0.561955	0.637173	0.637173	0.486153	-0.28019	MMP1
2,6-dimethylpiperidine-4543	1/100	0.561955	0.561955	0.637173	0.637173	0.486437	-0.28035	JUND
SR-95639A-4977	2/100	0.197972	0.408923	0.269513	0.556697	0.173566	-0.28111	BMP4;SECTM1
sirolimus-6967	2/100	0.197972	0.408923	0.269513	0.556697	0.173596	-0.28116	TXNIP;ITM2C
3-nitropropionic acid-6367	1/100	0.561955	0.561955	0.637173	0.637173	0.48975	-0.28226	GPX2
4-hydroxyphenazone-1915	1/100	0.561955	0.561955	0.637173	0.637173	0.490005	-0.28241	CEACAM5
16-phenyltetranorprostaglandin E2-7505	1/100	0.561955	0.561955	0.637173	0.637173	0.490631	-0.28277	SERPINA1
0317956-0000-3855	1/100	0.561955	0.561955	0.637173	0.637173	0.490637	-0.28277	TCN1
0297417-0002B-6900	1/100	0.561955	0.561955	0.637173	0.637173	0.490653	-0.28278	APP
15-delta prostaglandin J2-5591	1/100	0.561955	0.561955	0.637173	0.637173	0.491306	-0.28316	GPX2
5194442-6553	1/100	0.561955	0.561955	0.637173	0.637173	0.491917	-0.28351	BMP4
S-propranolol-6343	2/100	0.197972	0.408923	0.269513	0.556697	0.17514	-0.28366	COL1A1;AP1S1

simvastatin-3340	2/100	0.197972	0.408923	0.269513	0.556697	0.175725	-0.28461	COL1A1;PIGR
15-delta prostaglandin J2-201	1/100	0.561955	0.561955	0.637173	0.637173	0.495008	-0.28529	DCN
8-azaguanine-1670	1/100	0.561955	0.561955	0.637173	0.637173	0.495667	-0.28567	IFI6
0179445-0000-3733	1/100	0.561955	0.561955	0.637173	0.637173	0.496576	-0.28619	BMP4
sparteine-2134	2/100	0.197972	0.408923	0.269513	0.556697	0.176822	-0.28639	CCL20;PHLDA1
15(S)-15-methylprostaglandin E2-7489	1/100	0.561955	0.561955	0.637173	0.637173	0.497324	-0.28662	IFI27
5194442-6594	1/100	0.561955	0.561955	0.637173	0.637173	0.497991	-0.28701	AP1S1
6-azathymine-4106	1/100	0.561955	0.561955	0.637173	0.637173	0.498198	-0.28713	SECTM1
acacetin-4324	1/100	0.561955	0.561955	0.637173	0.637173	0.498874	-0.28752	MMP3
0225151-0000-6426	1/100	0.561955	0.561955	0.637173	0.637173	0.499752	-0.28802	AP1S1
6-benzylaminopurine-2351	1/100	0.561955	0.561955	0.637173	0.637173	0.500483	-0.28845	PROM1
sirolimus-6981	2/100	0.197972	0.408923	0.269513	0.556697	0.178434	-0.289	CEACAM5;TXNIP
3-acetylcoumarin-5259	1/100	0.561955	0.561955	0.637173	0.637173	0.50166	-0.28912	DCN
sisomicin-4014	2/100	0.197972	0.408923	0.269513	0.556697	0.178782	-0.28956	TSPAN8;TSPAN1
5224221-956	1/100	0.561955	0.561955	0.637173	0.637173	0.503559	-0.29022	ETS2
15(S)-15-methylprostaglandin E2-7526	1/100	0.561955	0.561955	0.637173	0.637173	0.503598	-0.29024	IFITM1
15-delta prostaglandin J2-6948	1/100	0.561955	0.561955	0.637173	0.637173	0.504782	-0.29092	ITM2C
2-deoxy-D-glucose-344	1/100	0.561955	0.561955	0.637173	0.637173	0.506588	-0.29196	TXNIP
16-phenyltetranorprostaglandin E2-7546	1/100	0.561955	0.561955	0.637173	0.637173	0.509839	-0.29384	CD24
SC-560-6870	2/100	0.197972	0.408923	0.269513	0.556697	0.181713	-0.29431	SECTM1;ITM2C
sparteine-4391	2/100	0.197972	0.408923	0.269513	0.556697	0.181744	-0.29436	DCN;ITM2C
(-)-atenolol-3067	1/100	0.561955	0.561955	0.637173	0.637173	0.511913	-0.29503	IFITM1
3-nitropropionic acid-6372	1/100	0.561955	0.561955	0.637173	0.637173	0.51357	-0.29599	APP
3-hydroxy-DL-kynurenine-1109	1/100	0.561955	0.561955	0.637173	0.637173	0.513704	-0.29606	APP
3-acetamidocoumarin-7361	1/100	0.561955	0.561955	0.637173	0.637173	0.514749	-0.29667	SECTM1
0317956-0000-4334	1/100	0.561955	0.561955	0.637173	0.637173	0.515684	-0.29721	RNF43
acenocoumarol-5878	1/100	0.561955	0.561955	0.637173	0.637173	0.515797	-0.29727	TSPAN8
acetylsalicylic acid-6164	1/100	0.561955	0.561955	0.637173	0.637173	0.516591	-0.29773	DSG2
5152487-896	1/100	0.561955	0.561955	0.637173	0.637173	0.51791	-0.29849	COL1A1
2-aminobenzenesulfonamide-5422	1/100	0.561955	0.561955	0.637173	0.637173	0.518192	-0.29865	IFI6
15(S)-15-methylprostaglandin E2-7521	1/100	0.561955	0.561955	0.637173	0.637173	0.518255	-0.29869	RNF43
4-hydroxyphenazone-1497	1/100	0.561955	0.561955	0.637173	0.637173	0.518362	-0.29875	LGALS3BP
5151277-903	1/100	0.561955	0.561955	0.637173	0.637173	0.520038	-0.29972	ID3
sulfachlorpyridazine-4326	2/100	0.197972	0.408923	0.269513	0.556697	0.185672	-0.30072	IL32;SERPINA1
6-bromoindirubin-3'-oxime-6589	1/100	0.561955	0.561955	0.637173	0.637173	0.521914	-0.3008	AP1S1

acenocoumarol-7232	1/100	0.561955	0.561955	0.637173	0.637173	0.522142	-0.30093	IFI6
sirolimus-1059	2/100	0.197972	0.408923	0.269513	0.556697	0.186444	-0.30197	TXNIP;PTMA
abamectin-7218	1/100	0.561955	0.561955	0.637173	0.637173	0.524718	-0.30241	TXNIP
sulfadimethoxine-4724	2/100	0.197972	0.408923	0.269513	0.556697	0.186746	-0.30246	MMP3;IFI6
0179445-0000-4755	1/100	0.561955	0.561955	0.637173	0.637173	0.526789	-0.30361	COL1A1
aconitine-2776	1/100	0.561955	0.561955	0.637173	0.637173	0.52681	-0.30362	AP1S1
alfadolone-6506	1/100	0.561955	0.561955	0.637173	0.637173	0.526937	-0.30369	TCN1
5186223-885	1/100	0.561955	0.561955	0.637173	0.637173	0.527687	-0.30412	ITM2B
sulfaquinoxaline-2528	2/100	0.197972	0.408923	0.269513	0.556697	0.188007	-0.3045	PROM1;DCN
SC-58125-542	2/100	0.197972	0.408923	0.269513	0.556697	0.188526	-0.30534	PIGR;ENC1
5211181-950	1/100	0.561955	0.561955	0.637173	0.637173	0.531421	-0.30628	PHLDA1
alsterpaullone-7056	1/100	0.561955	0.561955	0.637173	0.637173	0.532464	-0.30688	APP
adiphenine-1831	1/100	0.561955	0.561955	0.637173	0.637173	0.534045	-0.30779	AP1S1
5707885-6433	1/100	0.561955	0.561955	0.637173	0.637173	0.5343	-0.30794	ACTB
15-delta prostaglandin J2-564	1/100	0.561955	0.561955	0.637173	0.637173	0.535152	-0.30843	COL1A1
serotonin-5268	2/100	0.197972	0.408923	0.269513	0.556697	0.190467	-0.30849	COL1A1;AP1S1
adenosine phosphate-5359	1/100	0.561955	0.561955	0.637173	0.637173	0.535401	-0.30857	CEACAM6
aciclovir-4683	1/100	0.561955	0.561955	0.637173	0.637173	0.536491	-0.3092	FOS
sulfabenzamide-6634	2/100	0.197972	0.408923	0.269513	0.556697	0.191376	-0.30996	RNF43;MMP3
6-benzylaminopurine-3726	1/100	0.561955	0.561955	0.637173	0.637173	0.537879	-0.31	PIGR
AG-028671-6557	1/100	0.561955	0.561955	0.637173	0.637173	0.538707	-0.31047	APP
5155877-6544	1/100	0.561955	0.561955	0.637173	0.637173	0.540041	-0.31124	GPX2
adiphenine-7037	1/100	0.561955	0.561955	0.637173	0.637173	0.541316	-0.31198	AP1S1
5286656-889	1/100	0.561955	0.561955	0.637173	0.637173	0.541608	-0.31215	COL1A1
acacetin-2189	1/100	0.561955	0.561955	0.637173	0.637173	0.541631	-0.31216	CCL20
acemetacin-2411	1/100	0.561955	0.561955	0.637173	0.637173	0.541845	-0.31228	TFF2
5255229-833	1/100	0.561955	0.561955	0.637173	0.637173	0.54443	-0.31377	PHLDA1
6-bromoindirubin-3'-oxime-7101	1/100	0.561955	0.561955	0.637173	0.637173	0.544525	-0.31383	ITM2B
AG-013608-6435	1/100	0.561955	0.561955	0.637173	0.637173	0.545002	-0.3141	PROM1
sotalol-4079	2/100	0.197972	0.408923	0.269513	0.556697	0.194007	-0.31422	IL32;APP
6-azathymine-3987	1/100	0.561955	0.561955	0.637173	0.637173	0.545604	-0.31445	CEACAM6
alpha-ergocryptine-4552	1/100	0.561955	0.561955	0.637173	0.637173	0.546197	-0.31479	COL1A2
AG-013608-5949	1/100	0.561955	0.561955	0.637173	0.637173	0.547622	-0.31561	SPINK1
adipiodone-6490	1/100	0.561955	0.561955	0.637173	0.637173	0.548055	-0.31586	DCN
acetazolamide-1808	1/100	0.561955	0.561955	0.637173	0.637173	0.548055	-0.31586	ITM2B
alfadolone-3127	1/100	0.561955	0.561955	0.637173	0.637173	0.548167	-0.31593	APP
7-aminocephalosporanic acid-3258	1/100	0.561955	0.561955	0.637173	0.637173	0.549953	-0.31696	AP1S1

aconitine-7149	1/100	0.561955	0.561955	0.637173	0.637173	0.552177	-0.31824	S100A11
alpha-estradiol-762	1/100	0.561955	0.561955	0.637173	0.637173	0.552403	-0.31837	PIGR
sirolimus-5567	2/100	0.197972	0.408923	0.269513	0.556697	0.19718	-0.31936	SPINK1;TXNIP
solasodine-3830	2/100	0.197972	0.408923	0.269513	0.556697	0.197598	-0.32004	DCN;PSMB9
AG-012559-6920	1/100	0.561955	0.561955	0.637173	0.637173	0.557744	-0.32145	COL1A1
adrenosterone-5464	1/100	0.561955	0.561955	0.637173	0.637173	0.559862	-0.32267	IFI6
aminophenazone-6818	1/100	0.561955	0.561955	0.637173	0.637173	0.561652	-0.3237	COL1A1
acenocoumarol-2077	1/100	0.561955	0.561955	0.637173	0.637173	0.562142	-0.32398	LGALS3BP
6-bromoindirubin-3'-oxime-6559	1/100	0.561955	0.561955	0.637173	0.637173	0.56266	-0.32428	C3
AG-012559-6884	1/100	0.561955	0.561955	0.637173	0.637173	0.563086	-0.32453	AP1S1
solanine-4087	2/100	0.197972	0.408923	0.269513	0.556697	0.200484	-0.32471	SPARC;SLCO5A1
alexidine-2576	1/100	0.561955	0.561955	0.637173	0.637173	0.563628	-0.32484	DCN
sitosterol-2912	2/100	0.197972	0.408923	0.269513	0.556697	0.201598	-0.32651	IGFBP2;MMP3
tanespimycin-2666	2/100	0.197972	0.408923	0.269513	0.556697	0.201615	-0.32654	HSP90AB1;HSPB1
7-aminocephalosporanic acid-4242	1/100	0.561955	0.561955	0.637173	0.637173	0.566664	-0.32659	DCN
succinylsulfathiazole-2166	2/100	0.197972	0.408923	0.269513	0.556697	0.201773	-0.3268	MMP3;IFI6
succinylsulfathiazole-4265	2/100	0.197972	0.408923	0.269513	0.556697	0.201868	-0.32695	SPINK1;DUOX2
simvastatin-4828	2/100	0.197972	0.408923	0.269513	0.556697	0.202514	-0.328	COL1A1;IFI6
aconitine-6797	1/100	0.561955	0.561955	0.637173	0.637173	0.569516	-0.32823	IFI27
alfuzosin-3203	1/100	0.561955	0.561955	0.637173	0.637173	0.570168	-0.32861	CTGF
suramin sodium-7501	2/100	0.197972	0.408923	0.269513	0.556697	0.202937	-0.32868	CTSD;ETS2
aceclofenac-7029	1/100	0.561955	0.561955	0.637173	0.637173	0.573297	-0.33041	GPX2
alpha-estradiol-4434	1/100	0.561955	0.561955	0.637173	0.637173	0.573315	-0.33042	MMP3
AG-013608-6395	1/100	0.561955	0.561955	0.637173	0.637173	0.573623	-0.3306	PHLDA1
solasodine-3749	2/100	0.197972	0.408923	0.269513	0.556697	0.204172	-0.33068	COL1A2;DCN
ajmaline-2899	1/100	0.561955	0.561955	0.637173	0.637173	0.575768	-0.33183	ITM2C
adrenosterone-6486	1/100	0.561955	0.561955	0.637173	0.637173	0.575878	-0.3319	CTGF
alpha-estradiol-1210	1/100	0.561955	0.561955	0.637173	0.637173	0.576737	-0.33239	SERPINA1
alexidine-3699	1/100	0.561955	0.561955	0.637173	0.637173	0.578115	-0.33319	S100P
aconitine-1784	1/100	0.561955	0.561955	0.637173	0.637173	0.578221	-0.33325	PTMA
amikacin-5314	1/100	0.561955	0.561955	0.637173	0.637173	0.578965	-0.33368	S100A6
alexidine-7397	1/100	0.561955	0.561955	0.637173	0.637173	0.57932	-0.33388	S100P
amikacin-3233	1/100	0.561955	0.561955	0.637173	0.637173	0.580172	-0.33437	MMP3
alpha-yohimbine-6274	1/100	0.561955	0.561955	0.637173	0.637173	0.580396	-0.3345	APP
alvespimycin-4437	1/100	0.561955	0.561955	0.637173	0.637173	0.581219	-0.33498	HSPB1
amodiaquine-3186	1/100	0.561955	0.561955	0.637173	0.637173	0.581394	-0.33508	CTSE
sulfachlorpyridazine-3944	2/100	0.197972	0.408923	0.269513	0.556697	0.207984	-0.33686	COL1A1;C3

acetylsalicylsalicylic acid-1377	1/100	0.561955	0.561955	0.637173	0.637173	0.584714	-0.33699	TPI1
adrenosterone-5045	1/100	0.561955	0.561955	0.637173	0.637173	0.584959	-0.33713	CEACAM5
alprostadil-6555	1/100	0.561955	0.561955	0.637173	0.637173	0.585475	-0.33743	S100P
alsterpaullone-7078	1/100	0.561955	0.561955	0.637173	0.637173	0.585966	-0.33771	S100A11
alpha-ergocryptine-3434	1/100	0.561955	0.561955	0.637173	0.637173	0.586045	-0.33776	TXNIP
aminoglutethimide-7463	1/100	0.561955	0.561955	0.637173	0.637173	0.587065	-0.33835	IFI6
aminohippuric acid-6453	1/100	0.561955	0.561955	0.637173	0.637173	0.587818	-0.33878	S100A11
antimycin A-5053	1/100	0.561955	0.561955	0.637173	0.637173	0.589115	-0.33953	COL1A1
alcuronium chloride-7345	1/100	0.561955	0.561955	0.637173	0.637173	0.590523	-0.34034	SOX9
albendazole-3164	1/100	0.561955	0.561955	0.637173	0.637173	0.590915	-0.34056	CD14
sulfamethoxazole-3667	2/100	0.197972	0.408923	0.269513	0.556697	0.210904	-0.34159	PIGR;APP
alprenolol-7141	1/100	0.561955	0.561955	0.637173	0.637173	0.592908	-0.34171	JUND
amiprilose-4119	1/100	0.561955	0.561955	0.637173	0.637173	0.593465	-0.34203	ITM2C
altretamine-5688	1/100	0.561955	0.561955	0.637173	0.637173	0.594622	-0.3427	DCN
alverine-1426	1/100	0.561955	0.561955	0.637173	0.637173	0.595199	-0.34303	LY6E
alprenolol-3188	1/100	0.561955	0.561955	0.637173	0.637173	0.597312	-0.34425	DUOX2
tanespimycin-6986	2/100	0.197972	0.408923	0.269513	0.556697	0.212897	-0.34481	COL1A1;BMP4
altizide-2527	1/100	0.561955	0.561955	0.637173	0.637173	0.598961	-0.3452	PRSS23
alpha-estradiol-702	1/100	0.561955	0.561955	0.637173	0.637173	0.600263	-0.34595	ENC1
ambroxol-1623	1/100	0.561955	0.561955	0.637173	0.637173	0.60175	-0.34681	TPI1
alvespimycin-5210	1/100	0.561955	0.561955	0.637173	0.637173	0.603035	-0.34755	SOX9
solasodine-2170	2/100	0.197972	0.408923	0.269513	0.556697	0.214598	-0.34757	IFITM1;PHLDA1
tenoxicam-2501	2/100	0.197972	0.408923	0.269513	0.556697	0.214727	-0.34778	MMP3;TFF2
amiloride-1970	1/100	0.561955	0.561955	0.637173	0.637173	0.603707	-0.34794	LY6E
staurosporine-508	2/100	0.197972	0.408923	0.269513	0.556697	0.214897	-0.34805	GPX2;JUND
alprenolol-6789	1/100	0.561955	0.561955	0.637173	0.637173	0.605071	-0.34872	C3
amiodarone-5253	1/100	0.561955	0.561955	0.637173	0.637173	0.605677	-0.34907	DCN
alpha-yohimbine-5800	1/100	0.561955	0.561955	0.637173	0.637173	0.606711	-0.34967	SPP1
antimycin A-2261	1/100	0.561955	0.561955	0.637173	0.637173	0.607156	-0.34992	PIGR
tanespimycin-432	2/100	0.197972	0.408923	0.269513	0.556697	0.216196	-0.35016	HSPB1;AP1S1
AG-028671-6582	1/100	0.561955	0.561955	0.637173	0.637173	0.60858	-0.35074	ETS2
tanespimycin-1225	2/100	0.197972	0.408923	0.269513	0.556697	0.216582	-0.35078	HSPB1;CTGF
sulfaguanidine-4839	2/100	0.197972	0.408923	0.269513	0.556697	0.216954	-0.35138	COL1A1;ITM2C
amitriptyline-1865	1/100	0.561955	0.561955	0.637173	0.637173	0.610492	-0.35185	APP
amiloride-4109	1/100	0.561955	0.561955	0.637173	0.637173	0.610998	-0.35214	DCN
amantadine-1344	1/100	0.561955	0.561955	0.637173	0.637173	0.61237	-0.35293	ATP1B1
staurosporine-425	2/100	0.197972	0.408923	0.269513	0.556697	0.218088	-0.35322	S100P;PHLDA1

amodiaquine-5747	1/100	0.561955	0.561955	0.637173	0.637173	0.612946	-0.35326	CEACAM5
apramycin-2914	1/100	0.561955	0.561955	0.637173	0.637173	0.613001	-0.35329	CEACAM6
aminoglutethimide-7421	1/100	0.561955	0.561955	0.637173	0.637173	0.6131	-0.35335	IFITM1
aminophylline-3036	1/100	0.561955	0.561955	0.637173	0.637173	0.613969	-0.35385	ID3
arcaine-3010	1/100	0.561955	0.561955	0.637173	0.637173	0.614753	-0.3543	FXYD3
amikacin-1618	1/100	0.561955	0.561955	0.637173	0.637173	0.61577	-0.35489	TCN1
amitriptyline-6353	1/100	0.561955	0.561955	0.637173	0.637173	0.616016	-0.35503	CD24
alverine-6345	1/100	0.561955	0.561955	0.637173	0.637173	0.616781	-0.35547	COL1A1
aminocaproic acid-3122	1/100	0.561955	0.561955	0.637173	0.637173	0.617598	-0.35594	IFITM1
sulfanilamide-3449	2/100	0.197972	0.408923	0.269513	0.556697	0.220111	-0.3565	IFI6;SOX9
terguride-4633	2/100	0.197972	0.408923	0.269513	0.556697	0.22012	-0.35651	CEACAM5;FOS
amylocaine-4089	1/100	0.561955	0.561955	0.637173	0.637173	0.618602	-0.35652	CEACAM5
terconazole-4583	2/100	0.197972	0.408923	0.269513	0.556697	0.220326	-0.35685	AP1S1;CD24
asiaticoside-7244	1/100	0.561955	0.561955	0.637173	0.637173	0.619238	-0.35689	ITM2B
amitriptyline-1701	1/100	0.561955	0.561955	0.637173	0.637173	0.621309	-0.35808	C3
atovaquone-2480	1/100	0.561955	0.561955	0.637173	0.637173	0.621419	-0.35814	S100A11
alpha-estradiol-6970	1/100	0.561955	0.561955	0.637173	0.637173	0.621838	-0.35839	ITM2C
androsterone-4635	1/100	0.561955	0.561955	0.637173	0.637173	0.624647	-0.36	COL1A1
sulfinpyrazone-6230	2/100	0.197972	0.408923	0.269513	0.556697	0.223066	-0.36128	BMP4;AP1S1
amprolium-1479	1/100	0.561955	0.561955	0.637173	0.637173	0.628904	-0.36246	CEACAM5
aminophenazone-2222	1/100	0.561955	0.561955	0.637173	0.637173	0.630889	-0.3636	CFI
SR-95531-1316	2/100	0.197972	0.408923	0.269513	0.556697	0.224575	-0.36373	TCN1;PHLDA1
suxibuzone-7163	2/100	0.197972	0.408923	0.269513	0.556697	0.224669	-0.36388	SECTM1;S100A4
alpha-estradiol-6169	1/100	0.561955	0.561955	0.637173	0.637173	0.633922	-0.36535	PIGR
amoxicillin-1265	1/100	0.561955	0.561955	0.637173	0.637173	0.634743	-0.36582	ATP1B1
sulfadimidine-3940	2/100	0.197972	0.408923	0.269513	0.556697	0.225994	-0.36603	COL1A1;SECTM1
sulfamonomethoxine-2742	2/100	0.197972	0.408923	0.269513	0.556697	0.226046	-0.36611	COL1A1;OLFM4
amrinone-6826	1/100	0.561955	0.561955	0.637173	0.637173	0.638097	-0.36776	SOX9
tanespimycin-1631	2/100	0.197972	0.408923	0.269513	0.556697	0.227664	-0.36873	APP;TXNIP
sulfanilamide-6810	2/100	0.197972	0.408923	0.269513	0.556697	0.22772	-0.36882	SPINK1;SOX9
amitriptyline-167	1/100	0.561955	0.561955	0.637173	0.637173	0.641056	-0.36946	TCN1
articaine-6517	1/100	0.561955	0.561955	0.637173	0.637173	0.641065	-0.36947	RNF43
ampicillin-2030	1/100	0.561955	0.561955	0.637173	0.637173	0.641076	-0.36947	GPX2
aminophylline-6295	1/100	0.561955	0.561955	0.637173	0.637173	0.641219	-0.36956	COL1A2
sulfacetamide-1859	2/100	0.197972	0.408923	0.269513	0.556697	0.228346	-0.36984	TCN1;ID3
amoxapine-2013	1/100	0.561955	0.561955	0.637173	0.637173	0.642155	-0.3701	TPI1
aminohippuric acid-3076	1/100	0.561955	0.561955	0.637173	0.637173	0.643501	-0.37087	CEACAM6

talampicillin-7254	2/100	0.197972	0.408923	0.269513	0.556697	0.230007	-0.37253	IFNGR1;CEACAM6
testosterone-5636	2/100	0.197972	0.408923	0.269513	0.556697	0.230365	-0.37311	FOS;ITM2C
tetrandrine-6082	2/100	0.197972	0.408923	0.269513	0.556697	0.230407	-0.37318	C3;PHLDA1
amoxicillin-2620	1/100	0.561955	0.561955	0.637173	0.637173	0.648631	-0.37383	COL1A1
arecoline-2657	1/100	0.561955	0.561955	0.637173	0.637173	0.648646	-0.37384	IFI6
tenoxicam-4102	2/100	0.197972	0.408923	0.269513	0.556697	0.230841	-0.37388	APP;SLCO5A1
thioproperazine-2073	2/100	0.197972	0.408923	0.269513	0.556697	0.231008	-0.37415	CCL20;IFI6
sulindac-5103	2/100	0.197972	0.408923	0.269513	0.556697	0.231045	-0.37421	IFITM1;S100A11
thapsigargin-7053	2/100	0.197972	0.408923	0.269513	0.556697	0.231486	-0.37492	TXNIP;SOX9
thiamazole-4372	2/100	0.197972	0.408923	0.269513	0.556697	0.231592	-0.37509	AP1S1;PHLDA1
amitriptyline-1823	1/100	0.561955	0.561955	0.637173	0.637173	0.650962	-0.37517	CCL20
amprolium-4241	1/100	0.561955	0.561955	0.637173	0.637173	0.650988	-0.37519	SLCO5A1
tanespimycin-2678	2/100	0.197972	0.408923	0.269513	0.556697	0.23179	-0.37541	HSP90AB1;HSPB1
amrinone-2724	1/100	0.561955	0.561955	0.637173	0.637173	0.652997	-0.37634	DCN
amphotericin B-6303	1/100	0.561955	0.561955	0.637173	0.637173	0.653664	-0.37673	CXCL1
amphotericin B-2441	1/100	0.561955	0.561955	0.637173	0.637173	0.65374	-0.37677	CCL20
alvespimycin-993	1/100	0.561955	0.561955	0.637173	0.637173	0.653826	-0.37682	GPX2
benserazide-5322	1/100	0.561955	0.561955	0.637173	0.637173	0.65437	-0.37714	S100A4
terguride-3082	2/100	0.197972	0.408923	0.269513	0.556697	0.233128	-0.37758	COL1A1;MMP1
atropine methonitrate-7253	1/100	0.561955	0.561955	0.637173	0.637173	0.655575	-0.37783	CEACAM6
benfluorex-5327	1/100	0.561955	0.561955	0.637173	0.637173	0.656557	-0.3784	ETS2
BCB000038-7520	1/100	0.561955	0.561955	0.637173	0.637173	0.65716	-0.37874	CD24
sulfasalazine-204	2/100	0.197972	0.408923	0.269513	0.556697	0.233885	-0.37881	CEACAM5;DCN
streptozocin-7193	2/100	0.197972	0.408923	0.269513	0.556697	0.23393	-0.37888	TSPAN1;S100A11
arcaine-4974	1/100	0.561955	0.561955	0.637173	0.637173	0.657443	-0.37891	MMP3
benfluorex-2621	1/100	0.561955	0.561955	0.637173	0.637173	0.658808	-0.37969	IER2
timolol-5280	2/100	0.197972	0.408923	0.269513	0.556697	0.234927	-0.38049	COL1A1;COL1A2
atropine oxide-2216	1/100	0.561955	0.561955	0.637173	0.637173	0.661511	-0.38125	LYZ
aztreonam-5110	1/100	0.561955	0.561955	0.637173	0.637173	0.663186	-0.38222	MMP3
antimycin A-2098	1/100	0.561955	0.561955	0.637173	0.637173	0.664492	-0.38297	SPINK1
tanespimycin-5223	2/100	0.197972	0.408923	0.269513	0.556697	0.236984	-0.38383	IFI27;IFI6
amylocaine-4169	1/100	0.561955	0.561955	0.637173	0.637173	0.667607	-0.38476	CEACAM5
artemisinin-7247	1/100	0.561955	0.561955	0.637173	0.637173	0.667756	-0.38485	CEACAM6
arcaine-3349	1/100	0.561955	0.561955	0.637173	0.637173	0.668213	-0.38511	ITM2C
sulfafurazole-4661	2/100	0.197972	0.408923	0.269513	0.556697	0.238017	-0.3855	C3;RNF43
amoxapine-1931	1/100	0.561955	0.561955	0.637173	0.637173	0.66894	-0.38553	TSPAN8
arachidonic acid-604	1/100	0.561955	0.561955	0.637173	0.637173	0.669408	-0.3858	DCN

azathioprine-1945	1/100	0.561955	0.561955	0.637173	0.637173	0.66949	-0.38585	ITM2B
AR-A014418-7070	1/100	0.561955	0.561955	0.637173	0.637173	0.670182	-0.38625	APP
anabasine-2512	1/100	0.561955	0.561955	0.637173	0.637173	0.670948	-0.38669	PIGR
baclofen-1536	1/100	0.561955	0.561955	0.637173	0.637173	0.67103	-0.38674	COL1A1
benfotiamine-4312	1/100	0.561955	0.561955	0.637173	0.637173	0.67182	-0.38719	PHLDA1
syrosingopine-1761	2/100	0.197972	0.408923	0.269513	0.556697	0.239463	-0.38784	APP;CCL20
baclofen-5414	1/100	0.561955	0.561955	0.637173	0.637173	0.674495	-0.38873	COL1A1
amprolium-1898	1/100	0.561955	0.561955	0.637173	0.637173	0.675243	-0.38917	TGFBI
tanespimycin-6926	2/100	0.197972	0.408923	0.269513	0.556697	0.240285	-0.38917	BMP4;RNF43
baclofen-6313	1/100	0.561955	0.561955	0.637173	0.637173	0.675747	-0.38946	SERPINA1
tolazoline-4844	2/100	0.197972	0.408923	0.269513	0.556697	0.241262	-0.39076	COL1A1;MMP3
azaperone-3573	1/100	0.561955	0.561955	0.637173	0.637173	0.678073	-0.3908	DCN
atropine oxide-2054	1/100	0.561955	0.561955	0.637173	0.637173	0.680349	-0.39211	IFI6
apigenin-1321	1/100	0.561955	0.561955	0.637173	0.637173	0.681515	-0.39278	CCL20
bendroflumethiazide-2555	1/100	0.561955	0.561955	0.637173	0.637173	0.681928	-0.39302	MMP3
bacampicillin-3273	1/100	0.561955	0.561955	0.637173	0.637173	0.682307	-0.39324	IFI6
arecoline-5423	1/100	0.561955	0.561955	0.637173	0.637173	0.682854	-0.39355	PDZK1IP1
benzamil-3635	1/100	0.561955	0.561955	0.637173	0.637173	0.68413	-0.39429	IFI6
tinidazole-3896	2/100	0.197972	0.408923	0.269513	0.556697	0.243753	-0.39479	IFI27;IFI6
ampicillin-6307	1/100	0.561955	0.561955	0.637173	0.637173	0.68532	-0.39497	AP1S1
bacitracin-3109	1/100	0.561955	0.561955	0.637173	0.637173	0.685477	-0.39506	NQO1
tocainide-4256	2/100	0.197972	0.408923	0.269513	0.556697	0.244835	-0.39654	MMP3;SLCO5A1
baclofen-2036	1/100	0.561955	0.561955	0.637173	0.637173	0.688151	-0.3966	AP1S1
topiramate-915	2/100	0.197972	0.408923	0.269513	0.556697	0.244968	-0.39676	SERPINA1;TMPRSS3
amprolium-4825	1/100	0.561955	0.561955	0.637173	0.637173	0.688874	-0.39702	COL1A1
sulfaphenazole-1794	2/100	0.197972	0.408923	0.269513	0.556697	0.245792	-0.39809	APP;TSPAN8
testosterone-1295	2/100	0.197972	0.408923	0.269513	0.556697	0.246021	-0.39846	APP;GPX2
bendroflumethiazide-3415	1/100	0.561955	0.561955	0.637173	0.637173	0.69197	-0.39881	TMPRSS3
benperidol-4196	1/100	0.561955	0.561955	0.637173	0.637173	0.692215	-0.39895	CEACAM6
amylocaine-1991	1/100	0.561955	0.561955	0.637173	0.637173	0.693416	-0.39964	ETS2
bemegride-3051	1/100	0.561955	0.561955	0.637173	0.637173	0.693928	-0.39993	COL1A2
atropine-1768	1/100	0.561955	0.561955	0.637173	0.637173	0.694152	-0.40006	PIGR
benfotiamine-3837	1/100	0.561955	0.561955	0.637173	0.637173	0.695887	-0.40106	DCN
atropine oxide-1370	1/100	0.561955	0.561955	0.637173	0.637173	0.697054	-0.40174	ITM2B
azapropazone-7277	1/100	0.561955	0.561955	0.637173	0.637173	0.698656	-0.40266	S100A11
testosterone-5271	2/100	0.197972	0.408923	0.269513	0.556697	0.249081	-0.40342	SOX9;DCN
atractyloside-2573	1/100	0.561955	0.561955	0.637173	0.637173	0.700326	-0.40362	CCL20

antazoline-1556	1/100	0.561955	0.561955	0.637173	0.637173	0.700819	-0.40391	CEACAM5
apomorphine-2005	1/100	0.561955	0.561955	0.637173	0.637173	0.703568	-0.40549	FOS
betonicine-6063	1/100	0.561955	0.561955	0.637173	0.637173	0.704546	-0.40605	PTPRO
benzydamine-5811	1/100	0.561955	0.561955	0.637173	0.637173	0.705008	-0.40632	COL1A1
bicuculline-2796	1/100	0.561955	0.561955	0.637173	0.637173	0.705098	-0.40637	TCN1
bergenin-3467	1/100	0.561955	0.561955	0.637173	0.637173	0.70537	-0.40653	SOX9
thioridazine-1230	2/100	0.197972	0.408923	0.269513	0.556697	0.252067	-0.40826	CCL20;CXCL1
terazosin-5831	2/100	0.197972	0.408923	0.269513	0.556697	0.252623	-0.40916	IFI6;DCN
aztreonam-2282	1/100	0.561955	0.561955	0.637173	0.637173	0.709931	-0.40916	PDZK1IP1
bethanechol-3537	1/100	0.561955	0.561955	0.637173	0.637173	0.709975	-0.40918	ITM2C
benzocaine-4808	1/100	0.561955	0.561955	0.637173	0.637173	0.711302	-0.40995	MMP3
aztreonam-1435	1/100	0.561955	0.561955	0.637173	0.637173	0.711391	-0.41	JUND
benperidol-2475	1/100	0.561955	0.561955	0.637173	0.637173	0.712037	-0.41037	S100A11
benzonatate-5435	1/100	0.561955	0.561955	0.637173	0.637173	0.713297	-0.4111	DCN
betulin-2952	1/100	0.561955	0.561955	0.637173	0.637173	0.714562	-0.41183	ATP1B1
tonzonium bromide-4617	2/100	0.197972	0.408923	0.269513	0.556697	0.254672	-0.41248	JUND;FOS
bephenium hydroxynaphthoate-5263	1/100	0.561955	0.561955	0.637173	0.637173	0.716338	-0.41285	DCN
BCB000038-7542	1/100	0.561955	0.561955	0.637173	0.637173	0.716911	-0.41318	TFF2
tolbutamide-3804	2/100	0.197972	0.408923	0.269513	0.556697	0.255315	-0.41352	IFITM1;IFI6
beclometasone-4403	1/100	0.561955	0.561955	0.637173	0.637173	0.718197	-0.41392	SECTM1
bambuterol-1582	1/100	0.561955	0.561955	0.637173	0.637173	0.718202	-0.41392	CCL20
benzamil-6056	1/100	0.561955	0.561955	0.637173	0.637173	0.718681	-0.4142	TCN1
azlocillin-5788	1/100	0.561955	0.561955	0.637173	0.637173	0.719684	-0.41478	COL1A1
benfotiamine-2177	1/100	0.561955	0.561955	0.637173	0.637173	0.720529	-0.41527	TCN1
tanespimycin-1651	2/100	0.197972	0.408923	0.269513	0.556697	0.256403	-0.41528	TXNIP;SOX9
tolnaftate-1919	2/100	0.197972	0.408923	0.269513	0.556697	0.256858	-0.41601	IFITM1;TGFBI
betazole-1690	1/100	0.561955	0.561955	0.637173	0.637173	0.723273	-0.41685	BMP4
bumetanide-2409	1/100	0.561955	0.561955	0.637173	0.637173	0.72331	-0.41687	APP
benzydamine-7169	1/100	0.561955	0.561955	0.637173	0.637173	0.723941	-0.41723	CEACAM6
azacyclonol-2020	1/100	0.561955	0.561955	0.637173	0.637173	0.723949	-0.41724	CKB
tetramisole-2849	2/100	0.197972	0.408923	0.269513	0.556697	0.257617	-0.41724	IFITM1;IFI6
thioridazine-6989	2/100	0.197972	0.408923	0.269513	0.556697	0.257665	-0.41732	SOX9;PHLDA1
benzydamine-1552	1/100	0.561955	0.561955	0.637173	0.637173	0.724599	-0.41761	PLCB4
bergenin-2726	1/100	0.561955	0.561955	0.637173	0.637173	0.725053	-0.41787	CTGF
benzamil-2200	1/100	0.561955	0.561955	0.637173	0.637173	0.728837	-0.42005	TCN1
terbutaline-5764	2/100	0.197972	0.408923	0.269513	0.556697	0.260483	-0.42189	TFF1;S100P

benfotiamine-3931	1/100	0.561955	0.561955	0.637173	0.637173	0.732397	-0.42211	COL1A1
bambuterol-7239	1/100	0.561955	0.561955	0.637173	0.637173	0.732569	-0.4222	IFI27
benzbromarone-6669	1/100	0.561955	0.561955	0.637173	0.637173	0.733279	-0.42261	DCN
betazole-5445	1/100	0.561955	0.561955	0.637173	0.637173	0.734169	-0.42313	SERPINA1
bicuculline-4574	1/100	0.561955	0.561955	0.637173	0.637173	0.734549	-0.42335	C3
bicuculline-2139	1/100	0.561955	0.561955	0.637173	0.637173	0.736225	-0.42431	IFI6
benzylpenicillin-3577	1/100	0.561955	0.561955	0.637173	0.637173	0.736273	-0.42434	QPCT
benzocaine-2822	1/100	0.561955	0.561955	0.637173	0.637173	0.738154	-0.42542	C3
berberine-2770	1/100	0.561955	0.561955	0.637173	0.637173	0.739082	-0.42596	TFF2
bisoprolol-2642	1/100	0.561955	0.561955	0.637173	0.637173	0.739382	-0.42613	QPCT
ticarcillin-7185	2/100	0.197972	0.408923	0.269513	0.556697	0.263108	-0.42614	AP1S1;SOX9
aztreonam-2118	1/100	0.561955	0.561955	0.637173	0.637173	0.740303	-0.42666	BMP4
BCB000039-7536	1/100	0.561955	0.561955	0.637173	0.637173	0.740308	-0.42666	AP1S1
budesonide-2866	1/100	0.561955	0.561955	0.637173	0.637173	0.740705	-0.42689	IFI6
baclofen-1952	1/100	0.561955	0.561955	0.637173	0.637173	0.741544	-0.42738	PHLDA1
tanespimycin-6944	2/100	0.197972	0.408923	0.269513	0.556697	0.264118	-0.42777	HSP90AB1;ITM2C
bepridil-1274	1/100	0.561955	0.561955	0.637173	0.637173	0.743312	-0.4284	TSPAN13
benzthiazide-2989	1/100	0.561955	0.561955	0.637173	0.637173	0.743394	-0.42844	TFF2
terazosin-2530	2/100	0.197972	0.408923	0.269513	0.556697	0.264721	-0.42875	OLFM4;PHLDA1
bretylium tosilate-6674	1/100	0.561955	0.561955	0.637173	0.637173	0.743977	-0.42878	AP1S1
boldine-2804	1/100	0.561955	0.561955	0.637173	0.637173	0.74435	-0.42899	MMP3
betulinic acid-3281	1/100	0.561955	0.561955	0.637173	0.637173	0.745673	-0.42976	ETS2
BCB000039-7510	1/100	0.561955	0.561955	0.637173	0.637173	0.746181	-0.43005	PIGR
calycanthine-5744	1/100	0.561955	0.561955	0.637173	0.637173	0.747607	-0.43087	TGFBI
terguride-5694	2/100	0.197972	0.408923	0.269513	0.556697	0.266263	-0.43125	COL1A1;PTPRO
canadine-4020	1/100	0.561955	0.561955	0.637173	0.637173	0.748509	-0.43139	FOS
bromopride-3719	1/100	0.561955	0.561955	0.637173	0.637173	0.751203	-0.43294	MMP3
terbutaline-6240	2/100	0.197972	0.408923	0.269513	0.556697	0.267591	-0.4334	COL1A1;OLFM4
capsaicin-4612	1/100	0.561955	0.561955	0.637173	0.637173	0.752317	-0.43359	CCL20
torasemide-5476	2/100	0.197972	0.408923	0.269513	0.556697	0.268097	-0.43422	IFI27;SOX9
butacaine-6225	1/100	0.561955	0.561955	0.637173	0.637173	0.755928	-0.43567	CEACAM5
bergenin-5870	1/100	0.561955	0.561955	0.637173	0.637173	0.756026	-0.43572	TSPAN8
betulinic acid-1345	1/100	0.561955	0.561955	0.637173	0.637173	0.756115	-0.43577	NQO1
betaxolol-5669	1/100	0.561955	0.561955	0.637173	0.637173	0.757262	-0.43644	LYZ
boldine-2148	1/100	0.561955	0.561955	0.637173	0.637173	0.757353	-0.43649	NQO1
biotin-2428	1/100	0.561955	0.561955	0.637173	0.637173	0.757977	-0.43685	IFITM1
calcium folinate-3442	1/100	0.561955	0.561955	0.637173	0.637173	0.758648	-0.43723	RNF43

bromopride-4741	1/100	0.561955	0.561955	0.637173	0.637173	0.758971	-0.43742	COL1A1
calcium folinate-3703	1/100	0.561955	0.561955	0.637173	0.637173	0.759278	-0.4376	DUOX2
theobromine-3334	2/100	0.197972	0.408923	0.269513	0.556697	0.270478	-0.43808	COL1A1;TMPRSS3
C-75-6394	1/100	0.561955	0.561955	0.637173	0.637173	0.761867	-0.43909	ITM2C
butirosin-2518	1/100	0.561955	0.561955	0.637173	0.637173	0.762033	-0.43919	IFITM1
thiamine-2894	2/100	0.197972	0.408923	0.269513	0.556697	0.271217	-0.43927	MMP3;ITM2C
benzydamine-3169	1/100	0.561955	0.561955	0.637173	0.637173	0.76257	-0.43949	MMP3
blebbistatin-954	1/100	0.561955	0.561955	0.637173	0.637173	0.763242	-0.43988	CD24
cefadroxil-3259	1/100	0.561955	0.561955	0.637173	0.637173	0.764158	-0.44041	COL1A1
trichostatin A-1535	2/100	0.197972	0.408923	0.269513	0.556697	0.272366	-0.44113	TXNIP;ITM2C
carcinine-1305	1/100	0.561955	0.561955	0.637173	0.637173	0.767792	-0.4425	IFI6
benzylpenicillin-4501	1/100	0.561955	0.561955	0.637173	0.637173	0.768258	-0.44277	MMP3
carbachol-3042	1/100	0.561955	0.561955	0.637173	0.637173	0.769764	-0.44364	CCL20
tolazoline-4262	2/100	0.197972	0.408923	0.269513	0.556697	0.274442	-0.4445	COL1A2;OLFM4
tetracycline-2080	2/100	0.197972	0.408923	0.269513	0.556697	0.274453	-0.44451	IL32;BMP4
bupropion-3180	1/100	0.561955	0.561955	0.637173	0.637173	0.772079	-0.44498	SECTM1
butamben-5792	1/100	0.561955	0.561955	0.637173	0.637173	0.772526	-0.44523	IFI6
C-75-6423	1/100	0.561955	0.561955	0.637173	0.637173	0.772985	-0.4455	PROM1
butein-607	1/100	0.561955	0.561955	0.637173	0.637173	0.77444	-0.44634	SLPI
carbamazepine-5518	1/100	0.561955	0.561955	0.637173	0.637173	0.774558	-0.4464	IFI6
thioridazine-4085	2/100	0.197972	0.408923	0.269513	0.556697	0.275699	-0.44653	BMP4;FOS
canrenoic acid-7135	1/100	0.561955	0.561955	0.637173	0.637173	0.775178	-0.44676	TSPAN1
thioproperazine-2236	2/100	0.197972	0.408923	0.269513	0.556697	0.276065	-0.44712	C3;SPINK1
cefazolin-7385	1/100	0.561955	0.561955	0.637173	0.637173	0.777086	-0.44786	IL32
tinidazole-4370	2/100	0.197972	0.408923	0.269513	0.556697	0.276663	-0.44809	C3;AP1S1
bromocriptine-1925	1/100	0.561955	0.561955	0.637173	0.637173	0.777532	-0.44812	RNF43
butacaine-5748	1/100	0.561955	0.561955	0.637173	0.637173	0.777618	-0.44817	RNF43
capsaicin-5673	1/100	0.561955	0.561955	0.637173	0.637173	0.778624	-0.44875	C3
bromocriptine-5665	1/100	0.561955	0.561955	0.637173	0.637173	0.778755	-0.44882	RNF43
calycanthine-1771	1/100	0.561955	0.561955	0.637173	0.637173	0.779156	-0.44905	CEACAM6
carisoprodol-1314	1/100	0.561955	0.561955	0.637173	0.637173	0.782923	-0.45122	FXYD3
caffeic acid-3053	1/100	0.561955	0.561955	0.637173	0.637173	0.783378	-0.45149	COL1A1
canavanine-4197	1/100	0.561955	0.561955	0.637173	0.637173	0.783932	-0.45181	IFITM1
carteolol-4096	1/100	0.561955	0.561955	0.637173	0.637173	0.785435	-0.45267	COL1A2
ticlopidine-1475	2/100	0.197972	0.408923	0.269513	0.556697	0.279511	-0.4527	C3;ETS2
butamben-6093	1/100	0.561955	0.561955	0.637173	0.637173	0.786476	-0.45327	TCN1
canavanine-2141	1/100	0.561955	0.561955	0.637173	0.637173	0.78733	-0.45376	PRSS23

thioperamide-3055	2/100	0.197972	0.408923	0.269513	0.556697	0.280251	-0.4539	COL1A2;ID3
carbamazepine-1847	1/100	0.561955	0.561955	0.637173	0.637173	0.787954	-0.45412	APP
carbachol-5342	1/100	0.561955	0.561955	0.637173	0.637173	0.78843	-0.4544	SERPINA1
thiethylperazine-5756	2/100	0.197972	0.408923	0.269513	0.556697	0.280825	-0.45483	RNF43;S100A11
bupivacaine-2404	1/100	0.561955	0.561955	0.637173	0.637173	0.791531	-0.45619	PRSS23
thiostrepton-2462	2/100	0.197972	0.408923	0.269513	0.556697	0.281792	-0.4564	NQO1;ATP1B1
chlorcyclizine-6053	1/100	0.561955	0.561955	0.637173	0.637173	0.793362	-0.45724	FOS
C-75-6428	1/100	0.561955	0.561955	0.637173	0.637173	0.793393	-0.45726	IFI6
tremorine-5799	2/100	0.197972	0.408923	0.269513	0.556697	0.282771	-0.45798	IL32;RNF43
tiratricol-2096	2/100	0.197972	0.408923	0.269513	0.556697	0.283309	-0.45886	APP;IFI6
canadine-2818	1/100	0.561955	0.561955	0.637173	0.637173	0.796561	-0.45908	BMP4
carbamazepine-1805	1/100	0.561955	0.561955	0.637173	0.637173	0.799753	-0.46092	AP1S1
todralazine-1841	2/100	0.197972	0.408923	0.269513	0.556697	0.284665	-0.46105	PLCB4;ATP1B1
carbinoxamine-7138	1/100	0.561955	0.561955	0.637173	0.637173	0.800068	-0.46111	APP
carbarsone-3250	1/100	0.561955	0.561955	0.637173	0.637173	0.801056	-0.46168	COL1A1
butyl hydroxybenzoate-3069	1/100	0.561955	0.561955	0.637173	0.637173	0.801572	-0.46197	CEACAM5
triamcinolone-5835	2/100	0.197972	0.408923	0.269513	0.556697	0.285293	-0.46207	ID1;AP1S1
butamben-2531	1/100	0.561955	0.561955	0.637173	0.637173	0.8023	-0.46239	APP
cefaclor-2843	1/100	0.561955	0.561955	0.637173	0.637173	0.80279	-0.46267	ITM2C
tetrandrine-5821	2/100	0.197972	0.408923	0.269513	0.556697	0.285708	-0.46274	IFI6;TSPAN1
cefamandole-4718	1/100	0.561955	0.561955	0.637173	0.637173	0.805946	-0.46449	DCN
carbamazepine-835	1/100	0.561955	0.561955	0.637173	0.637173	0.806155	-0.46461	FOS
thioridazine-1986	2/100	0.197972	0.408923	0.269513	0.556697	0.287727	-0.46601	CEACAM5;SPP1
carisoprodol-4955	1/100	0.561955	0.561955	0.637173	0.637173	0.808898	-0.46619	BMP4
carcinine-4225	1/100	0.561955	0.561955	0.637173	0.637173	0.809724	-0.46667	DCN
cefaclor-4967	1/100	0.561955	0.561955	0.637173	0.637173	0.812909	-0.46851	BMP4
butyl hydroxybenzoate-6446	1/100	0.561955	0.561955	0.637173	0.637173	0.81294	-0.46852	PIGR
canrenoic acid-2065	1/100	0.561955	0.561955	0.637173	0.637173	0.813393	-0.46879	CCL20
tolmetin-4167	2/100	0.197972	0.408923	0.269513	0.556697	0.289502	-0.46889	CEACAM5;ITM2C
cefotaxime-1389	1/100	0.561955	0.561955	0.637173	0.637173	0.813716	-0.46897	ENC1
chloramphenicol-1795	1/100	0.561955	0.561955	0.637173	0.637173	0.815043	-0.46974	CD24
thioperamide-5270	2/100	0.197972	0.408923	0.269513	0.556697	0.290756	-0.47092	COL1A1;DCN
calcium folinate-2579	1/100	0.561955	0.561955	0.637173	0.637173	0.817096	-0.47092	NQO1
ticlopidine-1975	2/100	0.197972	0.408923	0.269513	0.556697	0.290905	-0.47116	CEACAM5;CKB
carbamazepine-952	1/100	0.561955	0.561955	0.637173	0.637173	0.818762	-0.47188	FOS
cefazolin-3426	1/100	0.561955	0.561955	0.637173	0.637173	0.818762	-0.47188	S100A11
cefalotin-6079	1/100	0.561955	0.561955	0.637173	0.637173	0.820374	-0.47281	TCN1

tracazolate-7339	2/100	0.197972	0.408923	0.269513	0.556697	0.292151	-0.47318	COL1A1;SECTM1
cefalexin-4654	1/100	0.561955	0.561955	0.637173	0.637173	0.821854	-0.47366	CEACAM5
carbachol-3380	1/100	0.561955	0.561955	0.637173	0.637173	0.82212	-0.47382	DCN
carbarsone-3991	1/100	0.561955	0.561955	0.637173	0.637173	0.823251	-0.47447	TSPAN8
chlorogenic acid-4024	1/100	0.561955	0.561955	0.637173	0.637173	0.823568	-0.47465	TSPAN8
chlormezanone-4636	1/100	0.561955	0.561955	0.637173	0.637173	0.825333	-0.47567	COL1A1
carisoprodol-3251	1/100	0.561955	0.561955	0.637173	0.637173	0.826846	-0.47654	IFI6
trichostatin A-2566	2/100	0.197972	0.408923	0.269513	0.556697	0.29442	-0.47685	TSPAN13;ATP1B1
cefalotin-4482	1/100	0.561955	0.561955	0.637173	0.637173	0.830447	-0.47861	C3
cefaclor-6622	1/100	0.561955	0.561955	0.637173	0.637173	0.830952	-0.47891	TSPAN1
Chicago Sky Blue 6B-4971	1/100	0.561955	0.561955	0.637173	0.637173	0.83162	-0.47929	BMP4
cefmetazole-2524	1/100	0.561955	0.561955	0.637173	0.637173	0.831631	-0.4793	IGFBP2
celecoxib-206	1/100	0.561955	0.561955	0.637173	0.637173	0.831722	-0.47935	COL1A2
CAY-10397-7082	1/100	0.561955	0.561955	0.637173	0.637173	0.832281	-0.47967	APP
cefazolin-4708	1/100	0.561955	0.561955	0.637173	0.637173	0.833332	-0.48028	IFI6
cefotiam-3319	1/100	0.561955	0.561955	0.637173	0.637173	0.835456	-0.4815	AP1S1
trichostatin A-1072	2/100	0.197972	0.408923	0.269513	0.556697	0.297344	-0.48159	TXNIP;ISG15
cetirizine-4231	1/100	0.561955	0.561955	0.637173	0.637173	0.835748	-0.48167	MMP3
cefoperazone-5424	1/100	0.561955	0.561955	0.637173	0.637173	0.83592	-0.48177	APP
tolnaftate-2001	2/100	0.197972	0.408923	0.269513	0.556697	0.29763	-0.48205	CEACAM5;ITM2B
chlorphenamine-1371	1/100	0.561955	0.561955	0.637173	0.637173	0.837127	-0.48246	ITM2B
cefsulodin-4067	1/100	0.561955	0.561955	0.637173	0.637173	0.837592	-0.48273	APP
cefotetan-4116	1/100	0.561955	0.561955	0.637173	0.637173	0.838968	-0.48353	CEACAM5
cefotaxime-7186	1/100	0.561955	0.561955	0.637173	0.637173	0.839146	-0.48363	ITM2B
cefepime-5761	1/100	0.561955	0.561955	0.637173	0.637173	0.839193	-0.48366	PSMB9
trichostatin A-1471	2/100	0.197972	0.408923	0.269513	0.556697	0.298937	-0.48417	TXNIP;PSMB9
carteolol-3276	1/100	0.561955	0.561955	0.637173	0.637173	0.840859	-0.48461	CEACAM5
chlorcyclizine-3810	1/100	0.561955	0.561955	0.637173	0.637173	0.842657	-0.48565	DCN
trichostatin A-1112	2/100	0.197972	0.408923	0.269513	0.556697	0.299994	-0.48588	TXNIP;ISG15
chlorpropamide-141	1/100	0.561955	0.561955	0.637173	0.637173	0.843326	-0.48604	CEACAM5
cefepime-6237	1/100	0.561955	0.561955	0.637173	0.637173	0.845661	-0.48738	BMP4
tretinoin-5208	2/100	0.197972	0.408923	0.269513	0.556697	0.301179	-0.4878	SOX9;TGFBI
tretinoin-224	2/100	0.197972	0.408923	0.269513	0.556697	0.301715	-0.48867	SOX9;PHLDA1
cetirizine-2829	1/100	0.561955	0.561955	0.637173	0.637173	0.848494	-0.48902	SPINK1
tribenoside-6328	2/100	0.197972	0.408923	0.269513	0.556697	0.302303	-0.48962	AP1S1;TGFBI
chloramphenicol-5466	1/100	0.561955	0.561955	0.637173	0.637173	0.851016	-0.49047	IFI6
chlormezanone-5697	1/100	0.561955	0.561955	0.637173	0.637173	0.851176	-0.49056	MMP3

cefixime-4390	1/100	0.561955	0.561955	0.637173	0.637173	0.851938	-0.491	DCN
cefalonium-4245	1/100	0.561955	0.561955	0.637173	0.637173	0.853964	-0.49217	PTPRO
trichostatin A-2777	2/100	0.197972	0.408923	0.269513	0.556697	0.304511	-0.4932	TXNIP;ITM2C
chlorpromazine-6977	1/100	0.561955	0.561955	0.637173	0.637173	0.856783	-0.49379	ITM2C
chloropyramine-4414	1/100	0.561955	0.561955	0.637173	0.637173	0.856889	-0.49385	ITM2C
chlormezanone-3235	1/100	0.561955	0.561955	0.637173	0.637173	0.857677	-0.49431	DCN
carbenoxolone-4093	1/100	0.561955	0.561955	0.637173	0.637173	0.859083	-0.49512	CEACAM5
cefsulodin-2988	1/100	0.561955	0.561955	0.637173	0.637173	0.859375	-0.49529	S100A11
tobramycin-4162	2/100	0.197972	0.408923	0.269513	0.556697	0.30601	-0.49562	COL1A1;CD14
chlorambucil-4523	1/100	0.561955	0.561955	0.637173	0.637173	0.861051	-0.49625	COL1A2
chlorpromazine-6176	1/100	0.561955	0.561955	0.637173	0.637173	0.862145	-0.49688	COL1A1
ciclopirox-6677	1/100	0.561955	0.561955	0.637173	0.637173	0.863038	-0.4974	FOS
chlorhexidine-5403	1/100	0.561955	0.561955	0.637173	0.637173	0.866475	-0.49938	SPINK1
chloropyrazine-5750	1/100	0.561955	0.561955	0.637173	0.637173	0.867673	-0.50007	SLCO5A1
cefuroxime-2526	1/100	0.561955	0.561955	0.637173	0.637173	0.867758	-0.50012	MMP3
cefoxitin-7148	1/100	0.561955	0.561955	0.637173	0.637173	0.867787	-0.50013	GPX2
tretinoin-447	2/100	0.197972	0.408923	0.269513	0.556697	0.308855	-0.50023	COL1A2;CTSE
chlorpropamide-3210	1/100	0.561955	0.561955	0.637173	0.637173	0.871331	-0.50218	PIGR
cefsulodin-4148	1/100	0.561955	0.561955	0.637173	0.637173	0.872771	-0.50301	CFI
celecoxib-482	1/100	0.561955	0.561955	0.637173	0.637173	0.8737	-0.50354	CEACAM5
Chicago Sky Blue 6B-3266	1/100	0.561955	0.561955	0.637173	0.637173	0.875344	-0.50449	CEACAM5
tretinoin-390	2/100	0.197972	0.408923	0.269513	0.556697	0.311519	-0.50455	ID1;CKB
chlorpromazine-426	1/100	0.561955	0.561955	0.637173	0.637173	0.875637	-0.50466	APP
chlorphenamine-6773	1/100	0.561955	0.561955	0.637173	0.637173	0.876597	-0.50521	CEACAM5
cinchonidine-7190	1/100	0.561955	0.561955	0.637173	0.637173	0.878035	-0.50604	CD24
ciclacillin-3800	1/100	0.561955	0.561955	0.637173	0.637173	0.879406	-0.50683	TSPAN8
triamterene-1819	2/100	0.197972	0.408923	0.269513	0.556697	0.313036	-0.507	CCL20;ITM2B
trichostatin A-2794	2/100	0.197972	0.408923	0.269513	0.556697	0.313046	-0.50702	TXNIP;ITM2C
trichostatin A-5417	2/100	0.197972	0.408923	0.269513	0.556697	0.313107	-0.50712	TXNIP;ITM2C
trichostatin A-2105	2/100	0.197972	0.408923	0.269513	0.556697	0.31406	-0.50866	MMP1;CTGF
cefuroxime-5787	1/100	0.561955	0.561955	0.637173	0.637173	0.883281	-0.50906	BMP4
chlortalidone-7152	1/100	0.561955	0.561955	0.637173	0.637173	0.885066	-0.51009	S100A11
clenbuterol-5631	1/100	0.561955	0.561955	0.637173	0.637173	0.885202	-0.51017	DUOX2
chlortalidone-1581	1/100	0.561955	0.561955	0.637173	0.637173	0.885294	-0.51022	IFITM3
cinoxacin-5783	1/100	0.561955	0.561955	0.637173	0.637173	0.885529	-0.51036	BMP4
chrysin-5505	1/100	0.561955	0.561955	0.637173	0.637173	0.886052	-0.51066	ITM2C
chlorpromazine-1822	1/100	0.561955	0.561955	0.637173	0.637173	0.887339	-0.5114	CCL20

chlortalidone-3198	1/100	0.561955	0.561955	0.637173	0.637173	0.887442	-0.51146	SLPI
trichostatin A-1732	2/100	0.197972	0.408923	0.269513	0.556697	0.315819	-0.51151	QPCT;ATP1B1
trichostatin A-5336	2/100	0.197972	0.408923	0.269513	0.556697	0.316051	-0.51189	TXNIP;SOX9
chlorphenesin-1432	1/100	0.561955	0.561955	0.637173	0.637173	0.888229	-0.51192	DCN
cefuroxime-6088	1/100	0.561955	0.561955	0.637173	0.637173	0.888352	-0.51199	C3
chrysin-3106	1/100	0.561955	0.561955	0.637173	0.637173	0.888589	-0.51212	MMP1
chlorpropamide-1594	1/100	0.561955	0.561955	0.637173	0.637173	0.889948	-0.51291	TCN1
triamterene-1697	2/100	0.197972	0.408923	0.269513	0.556697	0.317446	-0.51415	BMP4;SOX9
clemizole-2339	1/100	0.561955	0.561955	0.637173	0.637173	0.893774	-0.51511	COL1A2
chlortetracycline-5360	1/100	0.561955	0.561955	0.637173	0.637173	0.893959	-0.51522	SERPINA1
trichostatin A-3227	2/100	0.197972	0.408923	0.269513	0.556697	0.318128	-0.51525	TXNIP;LYZ
chenodeoxycholic acid-2402	1/100	0.561955	0.561955	0.637173	0.637173	0.894213	-0.51537	ITM2B
cinchonine-2789	1/100	0.561955	0.561955	0.637173	0.637173	0.894374	-0.51546	MMP3
cloxacillin-7483	1/100	0.561955	0.561955	0.637173	0.637173	0.894873	-0.51575	TFF2
clebopride-5412	1/100	0.561955	0.561955	0.637173	0.637173	0.896413	-0.51663	COL1A2
clonidine-4478	1/100	0.561955	0.561955	0.637173	0.637173	0.897956	-0.51752	IFITM1
ciclosporin-4411	1/100	0.561955	0.561955	0.637173	0.637173	0.899956	-0.51867	TXNIP
cinchocaine-1889	1/100	0.561955	0.561955	0.637173	0.637173	0.90101	-0.51928	ETS2
trichostatin A-5231	2/100	0.197972	0.408923	0.269513	0.556697	0.320767	-0.51952	FOS;CTGF
trichostatin A-2330	2/100	0.197972	0.408923	0.269513	0.556697	0.320784	-0.51955	TXNIP;ITM2C
triamcinolone-1395	2/100	0.197972	0.408923	0.269513	0.556697	0.321099	-0.52006	ACSL1;TXNIP
colecalciferol-3298	1/100	0.561955	0.561955	0.637173	0.637173	0.904743	-0.52143	S100A11
cloxacillin-2289	1/100	0.561955	0.561955	0.637173	0.637173	0.905636	-0.52195	MMP3
chlorzoxazone-1416	1/100	0.561955	0.561955	0.637173	0.637173	0.905824	-0.52206	TFF1
ciclosporin-602	1/100	0.561955	0.561955	0.637173	0.637173	0.906046	-0.52218	SLPI
tranylcypromine-1417	2/100	0.197972	0.408923	0.269513	0.556697	0.32261	-0.52251	ANXA2;PTMA
trichostatin A-4710	2/100	0.197972	0.408923	0.269513	0.556697	0.322667	-0.5226	IFI6;TXNIP
chlorpromazine-1864	1/100	0.561955	0.561955	0.637173	0.637173	0.909077	-0.52393	JUND
clofilium tosylate-6830	1/100	0.561955	0.561955	0.637173	0.637173	0.912275	-0.52577	PTPRO
chlorphenamine-2217	1/100	0.561955	0.561955	0.637173	0.637173	0.912484	-0.52589	RNF43
ciclosporin-261	1/100	0.561955	0.561955	0.637173	0.637173	0.912745	-0.52605	ID3
clenbuterol-4671	1/100	0.561955	0.561955	0.637173	0.637173	0.913177	-0.52629	MMP3
citiolone-6031	1/100	0.561955	0.561955	0.637173	0.637173	0.914155	-0.52686	TCN1
trichostatin A-3993	2/100	0.197972	0.408923	0.269513	0.556697	0.325521	-0.52722	MMP1;CTGF
chlorphenesin-2115	1/100	0.561955	0.561955	0.637173	0.637173	0.915408	-0.52758	SERPINA1
cinchonidine-2772	1/100	0.561955	0.561955	0.637173	0.637173	0.917072	-0.52854	CEACAM5
trichostatin A-5945	2/100	0.197972	0.408923	0.269513	0.556697	0.326459	-0.52874	TXNIP;ITM2C

clozapine-5226	1/100	0.561955	0.561955	0.637173	0.637173	0.919193	-0.52976	APP
chlorphenesin-7472	1/100	0.561955	0.561955	0.637173	0.637173	0.920214	-0.53035	SLPI
trichostatin A-3979	2/100	0.197972	0.408923	0.269513	0.556697	0.327872	-0.53103	SOX9;PSMB9
trichostatin A-2137	2/100	0.197972	0.408923	0.269513	0.556697	0.328288	-0.5317	TSPAN13;ATP1B1
clozapine-1289	1/100	0.561955	0.561955	0.637173	0.637173	0.922648	-0.53175	APP
trichostatin A-3787	2/100	0.197972	0.408923	0.269513	0.556697	0.328454	-0.53197	MMP1;CTGF
trichostatin A-3312	2/100	0.197972	0.408923	0.269513	0.556697	0.328581	-0.53218	TXNIP;ITM2C
cinchocaine-4149	1/100	0.561955	0.561955	0.637173	0.637173	0.923764	-0.5324	IFI6
trichostatin A-1284	2/100	0.197972	0.408923	0.269513	0.556697	0.328764	-0.53248	TSPAN13;ATP1B1
clebopride-2646	1/100	0.561955	0.561955	0.637173	0.637173	0.925474	-0.53338	PDIA3
chlorprothixene-1272	1/100	0.561955	0.561955	0.637173	0.637173	0.925576	-0.53344	ATP1B1
trichostatin A-2904	2/100	0.197972	0.408923	0.269513	0.556697	0.329403	-0.53351	TSPAN13;ATP1B1
ciprofloxacin-1522	1/100	0.561955	0.561955	0.637173	0.637173	0.927293	-0.53443	AP1S1
citalopram-3820	1/100	0.561955	0.561955	0.637173	0.637173	0.928072	-0.53488	JUND
trichostatin A-4483	2/100	0.197972	0.408923	0.269513	0.556697	0.330513	-0.53531	BMP4;CTGF
cimetidine-1964	1/100	0.561955	0.561955	0.637173	0.637173	0.928875	-0.53534	GPX2
trichostatin A-5903	2/100	0.197972	0.408923	0.269513	0.556697	0.330562	-0.53539	FOS;PSMB9
clioquinol-5258	1/100	0.561955	0.561955	0.637173	0.637173	0.929269	-0.53557	COL1A1
co-dergocrine mesilate-2793	1/100	0.561955	0.561955	0.637173	0.637173	0.929438	-0.53567	RNF43
trichostatin A-3077	2/100	0.197972	0.408923	0.269513	0.556697	0.331511	-0.53693	TSPAN13;AP1S1
citiolone-4311	1/100	0.561955	0.561955	0.637173	0.637173	0.931953	-0.53712	LYZ
clidinium bromide-4499	1/100	0.561955	0.561955	0.637173	0.637173	0.932547	-0.53746	BMP4
clemastine-2412	1/100	0.561955	0.561955	0.637173	0.637173	0.932643	-0.53751	GPX2
clozapine-5630	1/100	0.561955	0.561955	0.637173	0.637173	0.932779	-0.53759	SPARC
copper sulfate-438	1/100	0.561955	0.561955	0.637173	0.637173	0.933026	-0.53773	CKB
chlormezanone-1620	1/100	0.561955	0.561955	0.637173	0.637173	0.9337	-0.53812	PTMA
cimetidine-1464	1/100	0.561955	0.561955	0.637173	0.637173	0.93437	-0.53851	APP
ciprofibrate-6218	1/100	0.561955	0.561955	0.637173	0.637173	0.934598	-0.53864	DCN
convolamine-2771	1/100	0.561955	0.561955	0.637173	0.637173	0.937759	-0.54046	AP1S1
clofibrate-263	1/100	0.561955	0.561955	0.637173	0.637173	0.938311	-0.54078	LCN2
CP-690334-01-3909	1/100	0.561955	0.561955	0.637173	0.637173	0.938801	-0.54106	IFI6
clidinium bromide-2734	1/100	0.561955	0.561955	0.637173	0.637173	0.939188	-0.54129	COL1A1
ciprofloxacin-5299	1/100	0.561955	0.561955	0.637173	0.637173	0.939454	-0.54144	S100A4
trichostatin A-2949	2/100	0.197972	0.408923	0.269513	0.556697	0.334846	-0.54233	TSPAN13;ATP1B1
trichostatin A-1306	2/100	0.197972	0.408923	0.269513	0.556697	0.334951	-0.5425	TSPAN13;ATP1B1
colchicine-644	1/100	0.561955	0.561955	0.637173	0.637173	0.941981	-0.5429	CDH3
trichostatin A-5086	2/100	0.197972	0.408923	0.269513	0.556697	0.335439	-0.54329	BMP4;CTGF

convolamine-5876	1/100	0.561955	0.561955	0.637173	0.637173	0.943337	-0.54368	COL1A2
trichostatin A-3114	2/100	0.197972	0.408923	0.269513	0.556697	0.33632	-0.54471	TSPAN13;PROM1
clopamide-3220	1/100	0.561955	0.561955	0.637173	0.637173	0.945192	-0.54475	TFF2
corticosterone-1307	1/100	0.561955	0.561955	0.637173	0.637173	0.946249	-0.54535	CCL20
ciclosporin-3267	1/100	0.561955	0.561955	0.637173	0.637173	0.947641	-0.54616	COL1A1
ciclosporin-4586	1/100	0.561955	0.561955	0.637173	0.637173	0.950422	-0.54776	AP1S1
clopamide-6301	1/100	0.561955	0.561955	0.637173	0.637173	0.950915	-0.54804	CEACAM6
trichostatin A-6434	2/100	0.197972	0.408923	0.269513	0.556697	0.338462	-0.54818	ITM2C;PSMB9
cinnarizine-3175	1/100	0.561955	0.561955	0.637173	0.637173	0.95235	-0.54887	PIGR
clozapine-1170	1/100	0.561955	0.561955	0.637173	0.637173	0.954148	-0.54991	APP
trichostatin A-1891	2/100	0.197972	0.408923	0.269513	0.556697	0.339565	-0.54997	MMP1;CTGF
trichostatin A-5908	2/100	0.197972	0.408923	0.269513	0.556697	0.340279	-0.55113	MMP1;CTGF
clobetasol-6835	1/100	0.561955	0.561955	0.637173	0.637173	0.956705	-0.55138	AP1S1
copper sulfate-500	1/100	0.561955	0.561955	0.637173	0.637173	0.958271	-0.55228	DPEP1
trichostatin A-2881	2/100	0.197972	0.408923	0.269513	0.556697	0.341479	-0.55307	ITM2C;PSMB9
coralyne-2652	1/100	0.561955	0.561955	0.637173	0.637173	0.960922	-0.55381	C3
trichostatin A-4112	2/100	0.197972	0.408923	0.269513	0.556697	0.342241	-0.5543	CTGF;PSMB9
cobalt chloride-379	1/100	0.561955	0.561955	0.637173	0.637173	0.96197	-0.55442	SPP1
colchicine-3213	1/100	0.561955	0.561955	0.637173	0.637173	0.962513	-0.55473	PHLDA1
CP-319743-7537	1/100	0.561955	0.561955	0.637173	0.637173	0.962955	-0.55498	AP1S1
CP-320650-01-3822	1/100	0.561955	0.561955	0.637173	0.637173	0.964255	-0.55573	LUM
colforsin-7059	1/100	0.561955	0.561955	0.637173	0.637173	0.966207	-0.55686	ETS2
clidinium bromide-3476	1/100	0.561955	0.561955	0.637173	0.637173	0.966485	-0.55702	CTSE
clozapine-6947	1/100	0.561955	0.561955	0.637173	0.637173	0.966622	-0.5571	SERPINA1
clindamycin-2219	1/100	0.561955	0.561955	0.637173	0.637173	0.966736	-0.55716	PSMB9
clorgiline-6659	1/100	0.561955	0.561955	0.637173	0.637173	0.967167	-0.55741	DCN
trichostatin A-6340	2/100	0.197972	0.408923	0.269513	0.556697	0.344162	-0.55742	MMP1;CTGF
trichostatin A-5511	2/100	0.197972	0.408923	0.269513	0.556697	0.344856	-0.55854	TXNIP;SOX9
cobalt chloride-383	1/100	0.561955	0.561955	0.637173	0.637173	0.970039	-0.55907	LCN2
trihexyphenidyl-4133	2/100	0.197972	0.408923	0.269513	0.556697	0.34537	-0.55937	FOS;CTGF
corbadrine-7208	1/100	0.561955	0.561955	0.637173	0.637173	0.970765	-0.55948	MMP3
cloxacillin-2126	1/100	0.561955	0.561955	0.637173	0.637173	0.970995	-0.55962	JUND
trichostatin A-7503	2/100	0.197972	0.408923	0.269513	0.556697	0.345619	-0.55978	MMP1;CTGF
trichostatin A-2835	2/100	0.197972	0.408923	0.269513	0.556697	0.346047	-0.56047	IFI6;TXNIP
trichostatin A-4632	2/100	0.197972	0.408923	0.269513	0.556697	0.346746	-0.5616	CTGF;PSMB9
clozapine-1229	1/100	0.561955	0.561955	0.637173	0.637173	0.975094	-0.56198	LYZ
cyclopentolate-3555	1/100	0.561955	0.561955	0.637173	0.637173	0.97528	-0.56209	C3

clozapine-6188	1/100	0.561955	0.561955	0.637173	0.637173	0.976209	-0.56262	GPX2
cyclizine-1731	1/100	0.561955	0.561955	0.637173	0.637173	0.976418	-0.56274	CTSE
trichostatin A-5594	2/100	0.197972	0.408923	0.269513	0.556697	0.347893	-0.56346	IFI6;TXNIP
clozapine-1654	1/100	0.561955	0.561955	0.637173	0.637173	0.979522	-0.56453	SERPINA1
cloperastine-3710	1/100	0.561955	0.561955	0.637173	0.637173	0.979779	-0.56468	COL1A1
cotinine-2011	1/100	0.561955	0.561955	0.637173	0.637173	0.980069	-0.56485	SLC12A2
trichostatin A-1421	2/100	0.197972	0.408923	0.269513	0.556697	0.348857	-0.56502	TSPAN13;ATP1B1
trichostatin A-1793	2/100	0.197972	0.408923	0.269513	0.556697	0.348995	-0.56524	MMP1;CTGF
CP-690334-01-4561	1/100	0.561955	0.561955	0.637173	0.637173	0.980857	-0.5653	MMP3
CP-944629-7544	1/100	0.561955	0.561955	0.637173	0.637173	0.981542	-0.5657	COL1A1
clozapine-4453	1/100	0.561955	0.561955	0.637173	0.637173	0.981974	-0.56594	PIGR
trichostatin A-6171	2/100	0.197972	0.408923	0.269513	0.556697	0.349723	-0.56642	TSPAN13;QPCT
trichostatin A-5935	2/100	0.197972	0.408923	0.269513	0.556697	0.349766	-0.56649	TXNIP;PSMB9
coralyne-5418	1/100	0.561955	0.561955	0.637173	0.637173	0.983407	-0.56677	APP
clotrimazole-1549	1/100	0.561955	0.561955	0.637173	0.637173	0.985756	-0.56812	LY6E
ciprofloxacin-1939	1/100	0.561955	0.561955	0.637173	0.637173	0.986167	-0.56836	ACSL1
cyclic adenosine monophosphate-5108	1/100	0.561955	0.561955	0.637173	0.637173	0.986548	-0.56858	COL1A1
CP-645525-01-7527	1/100	0.561955	0.561955	0.637173	0.637173	0.986927	-0.5688	JUND
CP-690334-01-4558	1/100	0.561955	0.561955	0.637173	0.637173	0.987023	-0.56885	CTGF
CP-320650-01-3825	1/100	0.561955	0.561955	0.637173	0.637173	0.987827	-0.56932	TSPAN8
dantrolene-3978	1/100	0.561955	0.561955	0.637173	0.637173	0.989098	-0.57005	PHLDA1
cyanocobalamin-3252	1/100	0.561955	0.561955	0.637173	0.637173	0.990113	-0.57064	AP1S1
delsoline-7212	1/100	0.561955	0.561955	0.637173	0.637173	0.9905	-0.57086	CEACAM5
cromoglicic acid-5754	1/100	0.561955	0.561955	0.637173	0.637173	0.990854	-0.57106	DUOX2
trichostatin A-2370	2/100	0.197972	0.408923	0.269513	0.556697	0.352729	-0.57129	TSPAN13;CKB
deferoxamine-3842	1/100	0.561955	0.561955	0.637173	0.637173	0.992034	-0.57174	IFITM1
CP-863187-7512	1/100	0.561955	0.561955	0.637173	0.637173	0.992038	-0.57174	BMP4
cyanocobalamin-1315	1/100	0.561955	0.561955	0.637173	0.637173	0.994435	-0.57313	FXYD3
cyclobenzaprine-4834	1/100	0.561955	0.561955	0.637173	0.637173	0.99458	-0.57321	PHLDA1
trichostatin A-5260	2/100	0.197972	0.408923	0.269513	0.556697	0.354036	-0.57341	TXNIP;ITM2C
CP-690334-01-4383	1/100	0.561955	0.561955	0.637173	0.637173	0.994994	-0.57345	IFI6
conessine-4191	1/100	0.561955	0.561955	0.637173	0.637173	0.997398	-0.57483	CEACAM6
debrisoquine-3207	1/100	0.561955	0.561955	0.637173	0.637173	0.997555	-0.57492	APP
trichostatin A-6891	2/100	0.197972	0.408923	0.269513	0.556697	0.355374	-0.57558	IFI6;TXNIP
dehydrocholic acid-4620	1/100	0.561955	0.561955	0.637173	0.637173	0.999399	-0.57599	COL1A1
CP-690334-01-3906	1/100	0.561955	0.561955	0.637173	0.637173	1.00144	-0.57716	IFI6

dapsone-1868	1/100	0.561955	0.561955	0.637173	0.637173	1.002486	-0.57777	APP
dehydrocholic acid-1523	1/100	0.561955	0.561955	0.637173	0.637173	1.003959	-0.57862	COL1A1
trichostatin A-4821	2/100	0.197972	0.408923	0.269513	0.556697	0.357732	-0.57939	IFI6;ITM2C
cyproterone-4470	1/100	0.561955	0.561955	0.637173	0.637173	1.006597	-0.58014	MMP3
dantrolene-3786	1/100	0.561955	0.561955	0.637173	0.637173	1.00842	-0.58119	SLCO5A1
trichostatin A-5950	2/100	0.197972	0.408923	0.269513	0.556697	0.359518	-0.58229	MMP1;CTGF
decamethonium bromide-7353	1/100	0.561955	0.561955	0.637173	0.637173	1.01118	-0.58278	S100A11
troleandomycin-3985	2/100	0.197972	0.408923	0.269513	0.556697	0.360193	-0.58338	IL32;LUM
trichostatin A-6609	2/100	0.197972	0.408923	0.269513	0.556697	0.360483	-0.58385	BMP4;CTGF
demeclocycline-4267	1/100	0.561955	0.561955	0.637173	0.637173	1.013186	-0.58393	JUND
CP-320650-01-4382	1/100	0.561955	0.561955	0.637173	0.637173	1.014917	-0.58493	AP1S1
dantrolene-4343	1/100	0.561955	0.561955	0.637173	0.637173	1.015338	-0.58517	ENC1
cyclopentolate-5734	1/100	0.561955	0.561955	0.637173	0.637173	1.016486	-0.58584	APP
dequalinium chloride-2631	1/100	0.561955	0.561955	0.637173	0.637173	1.017345	-0.58633	CEACAM5
cyclopenthiazide-2905	1/100	0.561955	0.561955	0.637173	0.637173	1.018062	-0.58674	CCL20
dexpanthenol-1844	1/100	0.561955	0.561955	0.637173	0.637173	1.019041	-0.58731	ENC1
dexpanthenol-1802	1/100	0.561955	0.561955	0.637173	0.637173	1.019811	-0.58775	AP1S1
trichostatin A-6736	2/100	0.197972	0.408923	0.269513	0.556697	0.363124	-0.58813	MMP1;CTGF
diclofenac-445	1/100	0.561955	0.561955	0.637173	0.637173	1.021464	-0.5887	CTSE
trichostatin A-6932	2/100	0.197972	0.408923	0.269513	0.556697	0.363611	-0.58892	IFI6;TXNIP
debrisoquine-5288	1/100	0.561955	0.561955	0.637173	0.637173	1.021836	-0.58892	APP
dicycloverine-1483	1/100	0.561955	0.561955	0.637173	0.637173	1.022409	-0.58925	FOS
deferoxamine-4317	1/100	0.561955	0.561955	0.637173	0.637173	1.022987	-0.58958	JUND
debrisoquine-1591	1/100	0.561955	0.561955	0.637173	0.637173	1.023604	-0.58994	CKB
trichostatin A-7105	2/100	0.197972	0.408923	0.269513	0.556697	0.364347	-0.59011	MMP1;CTGF
diazoxide-1368	1/100	0.561955	0.561955	0.637173	0.637173	1.025116	-0.59081	ITM2B
crotamiton-3388	1/100	0.561955	0.561955	0.637173	0.637173	1.02518	-0.59085	IFI6
diflunisal-4210	1/100	0.561955	0.561955	0.637173	0.637173	1.025699	-0.59114	IFITM1
dexibuprofen-6712	1/100	0.561955	0.561955	0.637173	0.637173	1.026211	-0.59144	DUOX2
diflorasone-2142	1/100	0.561955	0.561955	0.637173	0.637173	1.027623	-0.59225	ACSL1
trimethobenzamide-4100	2/100	0.197972	0.408923	0.269513	0.556697	0.36578	-0.59243	APP;MMP3
deferoxamine-485	1/100	0.561955	0.561955	0.637173	0.637173	1.029575	-0.59338	SOX9
CP-863187-7508	1/100	0.561955	0.561955	0.637173	0.637173	1.029889	-0.59356	SOX9
dicloxacillin-5012	1/100	0.561955	0.561955	0.637173	0.637173	1.030257	-0.59377	TFF2
desipramine-3212	1/100	0.561955	0.561955	0.637173	0.637173	1.030439	-0.59388	APP
danazol-2038	1/100	0.561955	0.561955	0.637173	0.637173	1.031214	-0.59432	MMP7
demecarium bromide-1781	1/100	0.561955	0.561955	0.637173	0.637173	1.032003	-0.59478	GPX2

demeclocycline-4728	1/100	0.561955	0.561955	0.637173	0.637173	1.032178	-0.59488	ITM2C
dexpanthenol-1680	1/100	0.561955	0.561955	0.637173	0.637173	1.033	-0.59535	BMP4
desipramine-6693	1/100	0.561955	0.561955	0.637173	0.637173	1.033446	-0.59561	JUND
cyclizine-5525	1/100	0.561955	0.561955	0.637173	0.637173	1.034462	-0.5962	SECTM1
depudecin-982	1/100	0.561955	0.561955	0.637173	0.637173	1.035258	-0.59665	TXNIP
dextromethorphan-1281	1/100	0.561955	0.561955	0.637173	0.637173	1.036987	-0.59765	CTSD
trichostatin A-5484	2/100	0.197972	0.408923	0.269513	0.556697	0.369484	-0.59843	TXNIP;SOX9
demeclocycline-3706	1/100	0.561955	0.561955	0.637173	0.637173	1.040061	-0.59942	IFI6
dipivefrine-1752	1/100	0.561955	0.561955	0.637173	0.637173	1.040933	-0.59992	PHLDA1
dextromethorphan-2636	1/100	0.561955	0.561955	0.637173	0.637173	1.041404	-0.6002	DCN
desoxycortone-5357	1/100	0.561955	0.561955	0.637173	0.637173	1.042981	-0.60111	ETS2
demeclocycline-2545	1/100	0.561955	0.561955	0.637173	0.637173	1.043184	-0.60122	DCN
daunorubicin-7525	1/100	0.561955	0.561955	0.637173	0.637173	1.043567	-0.60144	SOX9
cyclic adenosine monophosphate-2969	1/100	0.561955	0.561955	0.637173	0.637173	1.04618	-0.60295	CCL20
diethylstilbestrol-4547	1/100	0.561955	0.561955	0.637173	0.637173	1.046732	-0.60327	CEACAM5
valinomycin-5957	2/100	0.197972	0.408923	0.269513	0.556697	0.372572	-0.60343	TXNIP;SECTM1
cyclopentolate-6132	1/100	0.561955	0.561955	0.637173	0.637173	1.047918	-0.60395	TMPRSS3
dioxybenzone-5699	1/100	0.561955	0.561955	0.637173	0.637173	1.048595	-0.60434	COL1A1
dequalinium chloride-1276	1/100	0.561955	0.561955	0.637173	0.637173	1.048673	-0.60439	CEACAM6
dilazep-3665	1/100	0.561955	0.561955	0.637173	0.637173	1.048807	-0.60446	QPCT
dipivefrine-6766	1/100	0.561955	0.561955	0.637173	0.637173	1.048915	-0.60453	DCN
dicycloverine-1983	1/100	0.561955	0.561955	0.637173	0.637173	1.050609	-0.6055	CEACAM5
daunorubicin-7507	1/100	0.561955	0.561955	0.637173	0.637173	1.051397	-0.60596	SOX9
delsoline-5858	1/100	0.561955	0.561955	0.637173	0.637173	1.051981	-0.60629	AP1S1
dexamethasone-6271	1/100	0.561955	0.561955	0.637173	0.637173	1.052316	-0.60648	COL1A1
diflunisal-1990	1/100	0.561955	0.561955	0.637173	0.637173	1.052834	-0.60678	CEACAM5
trichostatin A-6784	2/100	0.197972	0.408923	0.269513	0.556697	0.375272	-0.6078	TXNIP;PSMB9
diclofenamide-3366	1/100	0.561955	0.561955	0.637173	0.637173	1.055202	-0.60815	AP1S1
triflupromazine-7466	2/100	0.197972	0.408923	0.269513	0.556697	0.375624	-0.60837	COL1A1;SLPI
diltiazem-1532	1/100	0.561955	0.561955	0.637173	0.637173	1.057227	-0.60932	CEACAM5
demecolcine-1103	1/100	0.561955	0.561955	0.637173	0.637173	1.057333	-0.60938	JUND
dexamethasone-2079	1/100	0.561955	0.561955	0.637173	0.637173	1.057839	-0.60967	CCL20
dipivefrine-2744	1/100	0.561955	0.561955	0.637173	0.637173	1.060206	-0.61103	APP
trichostatin A-7073	2/100	0.197972	0.408923	0.269513	0.556697	0.378059	-0.61232	MMP1;CTGF
diflorasone-4077	1/100	0.561955	0.561955	0.637173	0.637173	1.063231	-0.61278	ID1
dehydrocholic acid-5681	1/100	0.561955	0.561955	0.637173	0.637173	1.064091	-0.61327	MMP3

diltiazem-5309	1/100	0.561955	0.561955	0.637173	0.637173	1.064194	-0.61333	S100A4
diphemanil metilsulfate-1494	1/100	0.561955	0.561955	0.637173	0.637173	1.064365	-0.61343	ITM2C
demeclocycline-3604	1/100	0.561955	0.561955	0.637173	0.637173	1.064434	-0.61347	SECTM1
diperodon-4498	1/100	0.561955	0.561955	0.637173	0.637173	1.064643	-0.61359	MMP3
tubocurarine chloride-5449	2/100	0.197972	0.408923	0.269513	0.556697	0.379739	-0.61504	IFITM1;IFI6
trichostatin A-6064	2/100	0.197972	0.408923	0.269513	0.556697	0.379882	-0.61527	IFI6;PSMB9
desipramine-5292	1/100	0.561955	0.561955	0.637173	0.637173	1.068091	-0.61558	APP
triprolidine-2376	2/100	0.197972	0.408923	0.269513	0.556697	0.380184	-0.61576	APP;PRSS23
troglitazone-6191	2/100	0.197972	0.408923	0.269513	0.556697	0.380557	-0.61636	SECTM1;DCN
dicycloverine-4581	1/100	0.561955	0.561955	0.637173	0.637173	1.069468	-0.61637	BMP4
diethylcarbamazine-5066	1/100	0.561955	0.561955	0.637173	0.637173	1.070013	-0.61668	SERPINA1
diprophylline-1689	1/100	0.561955	0.561955	0.637173	0.637173	1.072378	-0.61805	GPX2
trifluoperazine-4448	2/100	0.197972	0.408923	0.269513	0.556697	0.381726	-0.61826	JUND;S100P
tropicamide-3722	2/100	0.197972	0.408923	0.269513	0.556697	0.381727	-0.61826	PIGR;COL1A2
dexpropranolol-6130	1/100	0.561955	0.561955	0.637173	0.637173	1.073326	-0.61859	SECTM1
docosahexaenoic acid ethyl ester-664	1/100	0.561955	0.561955	0.637173	0.637173	1.073417	-0.61865	TFF3
dicloxacillin-2445	1/100	0.561955	0.561955	0.637173	0.637173	1.073874	-0.61891	PIGR
dicloxacillin-3307	1/100	0.561955	0.561955	0.637173	0.637173	1.074498	-0.61927	PIGR
diphenylpyraline-4299	1/100	0.561955	0.561955	0.637173	0.637173	1.074918	-0.61951	CFI
trifluoperazine-5442	2/100	0.197972	0.408923	0.269513	0.556697	0.382863	-0.6201	COL1A1;SECTM1
valproic acid-1078	2/100	0.197972	0.408923	0.269513	0.556697	0.38293	-0.62021	PGM1;ITM2B
dipyridamole-4656	1/100	0.561955	0.561955	0.637173	0.637173	1.080234	-0.62258	RNF43
dl-alpha tocopherol-3256	1/100	0.561955	0.561955	0.637173	0.637173	1.080243	-0.62258	CEACAM5
dinoprost-5409	1/100	0.561955	0.561955	0.637173	0.637173	1.080257	-0.62259	S100A11
difenidol-2374	1/100	0.561955	0.561955	0.637173	0.637173	1.080474	-0.62271	APP
diethylstilbestrol-2567	1/100	0.561955	0.561955	0.637173	0.637173	1.08283	-0.62407	NQO1
vigabatrin-5415	2/100	0.197972	0.408923	0.269513	0.556697	0.385568	-0.62448	CEACAM6;MMP3
diflorasone-2798	1/100	0.561955	0.561955	0.637173	0.637173	1.083723	-0.62459	PIGR
doxazosin-4988	1/100	0.561955	0.561955	0.637173	0.637173	1.086515	-0.6262	SPINK1
diprophylline-1853	1/100	0.561955	0.561955	0.637173	0.637173	1.086944	-0.62644	TPI1
trichostatin A-6910	2/100	0.197972	0.408923	0.269513	0.556697	0.386871	-0.62659	TXNIP;SOX9
diethylcarbamazine-5485	1/100	0.561955	0.561955	0.637173	0.637173	1.089216	-0.62775	TMPRSS3
dicycloverine-1902	1/100	0.561955	0.561955	0.637173	0.637173	1.091333	-0.62897	CD24
tropine-5790	2/100	0.197972	0.408923	0.269513	0.556697	0.38862	-0.62942	IL32;DCN
trichostatin A-7236	2/100	0.197972	0.408923	0.269513	0.556697	0.389177	-0.63032	TXNIP;ITM2C
trichostatin A-6316	2/100	0.197972	0.408923	0.269513	0.556697	0.389256	-0.63045	MMP1;CTGF

diethylstilbestrol-3812	1/100	0.561955	0.561955	0.637173	0.637173	1.094461	-0.63077	JUND
droperidol-2645	1/100	0.561955	0.561955	0.637173	0.637173	1.095518	-0.63138	C3
diperodon-1575	1/100	0.561955	0.561955	0.637173	0.637173	1.096044	-0.63169	TPI1
diphemanil metilsulfate-1912	1/100	0.561955	0.561955	0.637173	0.637173	1.097226	-0.63237	ID3
dinoprost-6308	1/100	0.561955	0.561955	0.637173	0.637173	1.099452	-0.63365	CEACAM5
doxazosin-6642	1/100	0.561955	0.561955	0.637173	0.637173	1.101405	-0.63478	SPINK1
troglitazone-1012	2/100	0.197972	0.408923	0.269513	0.556697	0.392241	-0.63529	C3;FOS
disulfiram-5729	1/100	0.561955	0.561955	0.637173	0.637173	1.102882	-0.63563	FOS
dorzolamide-6142	1/100	0.561955	0.561955	0.637173	0.637173	1.104906	-0.63679	COL1A2
diethylcarbamazine-2394	1/100	0.561955	0.561955	0.637173	0.637173	1.106675	-0.63781	PIGR
enalapril-7428	1/100	0.561955	0.561955	0.637173	0.637173	1.106743	-0.63785	IFITM1
doxylamine-1893	1/100	0.561955	0.561955	0.637173	0.637173	1.107023	-0.63801	DEK
diphenhydramine-6020	1/100	0.561955	0.561955	0.637173	0.637173	1.108154	-0.63867	TGFBI
trichostatin A-7005	2/100	0.197972	0.408923	0.269513	0.556697	0.394347	-0.6387	TXNIP;PSMB9
trichostatin A-5822	2/100	0.197972	0.408923	0.269513	0.556697	0.394592	-0.63909	MMP1;CTGF
diloxanide-6679	1/100	0.561955	0.561955	0.637173	0.637173	1.109617	-0.63951	AP1S1
dioxybenzone-4638	1/100	0.561955	0.561955	0.637173	0.637173	1.109694	-0.63955	SERPINA1
dimethadione-3367	1/100	0.561955	0.561955	0.637173	0.637173	1.110217	-0.63986	DCN
trifluoperazine-7420	2/100	0.197972	0.408923	0.269513	0.556697	0.395121	-0.63995	FOS;PHLDA1
vigabatrin-6314	2/100	0.197972	0.408923	0.269513	0.556697	0.395233	-0.64013	CEACAM6;AP1S1
dropropizine-7429	1/100	0.561955	0.561955	0.637173	0.637173	1.112017	-0.64089	DCN
DL-thiorphan-6249	1/100	0.561955	0.561955	0.637173	0.637173	1.112326	-0.64107	QPCT
valproic acid-5211	2/100	0.197972	0.408923	0.269513	0.556697	0.395956	-0.6413	PIGR;SECTM1
troglitazone-2692	2/100	0.197972	0.408923	0.269513	0.556697	0.395996	-0.64137	COL1A1;PIGR
dipyridamole-1517	1/100	0.561955	0.561955	0.637173	0.637173	1.113434	-0.64171	AP1S1
dobutamine-3206	1/100	0.561955	0.561955	0.637173	0.637173	1.113692	-0.64186	PHLDA1
doxycycline-5838	1/100	0.561955	0.561955	0.637173	0.637173	1.113834	-0.64194	TSPAN8
dropropizine-5106	1/100	0.561955	0.561955	0.637173	0.637173	1.114959	-0.64259	COL1A1
diphemanil metilsulfate-4416	1/100	0.561955	0.561955	0.637173	0.637173	1.11603	-0.64321	ITM2C
diphemanil metilsulfate-1994	1/100	0.561955	0.561955	0.637173	0.637173	1.116957	-0.64374	LY6E
dinoprostone-6552	1/100	0.561955	0.561955	0.637173	0.637173	1.117756	-0.6442	TFF2
trichostatin A-7555	2/100	0.197972	0.408923	0.269513	0.556697	0.397936	-0.64451	MMP1;CTGF
eldeline-3831	1/100	0.561955	0.561955	0.637173	0.637173	1.118846	-0.64483	COL1A1
valproic acid-1181	2/100	0.197972	0.408923	0.269513	0.556697	0.398841	-0.64598	TSPAN13;QPCT
diflunisal-1908	1/100	0.561955	0.561955	0.637173	0.637173	1.122102	-0.64671	FXYD3
dipivefrine-7124	1/100	0.561955	0.561955	0.637173	0.637173	1.12437	-0.64801	S100P
doxylamine-4235	1/100	0.561955	0.561955	0.637173	0.637173	1.124699	-0.6482	COL1A2

dl-alpha tocopherol-4961	1/100	0.561955	0.561955	0.637173	0.637173	1.125113	-0.64844	COL1A2
dienestrol-5727	1/100	0.561955	0.561955	0.637173	0.637173	1.126073	-0.64899	RNF43
tyrphostin AG-825-1114	2/100	0.197972	0.408923	0.269513	0.556697	0.400999	-0.64947	ISG15;ETS2
dropropizine-2398	1/100	0.561955	0.561955	0.637173	0.637173	1.128959	-0.65066	CEACAM6
dioxybenzone-6478	1/100	0.561955	0.561955	0.637173	0.637173	1.12902	-0.65069	DUOX2
triflupromazine-1691	2/100	0.197972	0.408923	0.269513	0.556697	0.402055	-0.65118	COL1A1;JUND
trichostatin A-981	2/100	0.197972	0.408923	0.269513	0.556697	0.402293	-0.65157	TXNIP;FOS
verteporfin-3556	2/100	0.197972	0.408923	0.269513	0.556697	0.402514	-0.65192	SLPI;MMP3
vinblastine-7551	2/100	0.197972	0.408923	0.269513	0.556697	0.402772	-0.65234	COL1A1;ENC1
dl-alpha tocopherol-1320	1/100	0.561955	0.561955	0.637173	0.637173	1.1331	-0.65304	JUND
troglitazone-5592	2/100	0.197972	0.408923	0.269513	0.556697	0.403296	-0.65319	TCN1;FOS
domperidone-5701	1/100	0.561955	0.561955	0.637173	0.637173	1.135603	-0.65449	COL1A1
epirizole-7292	1/100	0.561955	0.561955	0.637173	0.637173	1.136408	-0.65495	TGFBI
equilin-4659	1/100	0.561955	0.561955	0.637173	0.637173	1.137007	-0.6553	CEACAM5
triprolidine-7008	2/100	0.197972	0.408923	0.269513	0.556697	0.404609	-0.65532	APP;ENC1
edrophonium chloride-1519	1/100	0.561955	0.561955	0.637173	0.637173	1.137479	-0.65557	S100A11
dydrogesterone-4836	1/100	0.561955	0.561955	0.637173	0.637173	1.137538	-0.6556	IFI6
estradiol-2653	1/100	0.561955	0.561955	0.637173	0.637173	1.138137	-0.65595	C3
ebselen-2717	1/100	0.561955	0.561955	0.637173	0.637173	1.138216	-0.65599	NQO1
dyclonine-7022	1/100	0.561955	0.561955	0.637173	0.637173	1.138909	-0.65639	GPX2
dropropizine-5531	1/100	0.561955	0.561955	0.637173	0.637173	1.139236	-0.65658	AP1S1
dicoumarol-3848	1/100	0.561955	0.561955	0.637173	0.637173	1.142254	-0.65832	COL1A1
dyclonine-2392	1/100	0.561955	0.561955	0.637173	0.637173	1.143752	-0.65918	ATP1B1
trimethylcolchicinic acid-2802	2/100	0.197972	0.408923	0.269513	0.556697	0.407359	-0.65977	COL1A1;PIGR
estradiol-6957	1/100	0.561955	0.561955	0.637173	0.637173	1.145779	-0.66035	TFF2
erythromycin-6729	1/100	0.561955	0.561955	0.637173	0.637173	1.146023	-0.66049	LYZ
ergocalciferol-3304	1/100	0.561955	0.561955	0.637173	0.637173	1.147132	-0.66113	COL1A1
doxepin-2384	1/100	0.561955	0.561955	0.637173	0.637173	1.148047	-0.66166	CD24
esculin-3390	1/100	0.561955	0.561955	0.637173	0.637173	1.148102	-0.66169	SPINK1
triflusal-1717	2/100	0.197972	0.408923	0.269513	0.556697	0.40871	-0.66196	IFITM3;QPCT
doxylamine-1473	1/100	0.561955	0.561955	0.637173	0.637173	1.148961	-0.66218	APP
diprophylline-5063	1/100	0.561955	0.561955	0.637173	0.637173	1.14917	-0.66231	PSMB9
valproic acid-2669	2/100	0.197972	0.408923	0.269513	0.556697	0.409119	-0.66262	TSPAN13;ATP1B1
doxepin-6337	1/100	0.561955	0.561955	0.637173	0.637173	1.149842	-0.66269	SERPINA1
dorzolamide-6259	1/100	0.561955	0.561955	0.637173	0.637173	1.149929	-0.66274	SOX9
F0447-0125-6401	1/100	0.561955	0.561955	0.637173	0.637173	1.150183	-0.66289	JUND
eldeline-4306	1/100	0.561955	0.561955	0.637173	0.637173	1.150265	-0.66294	MMP3

dl-alpha tocopherol-6616	1/100	0.561955	0.561955	0.637173	0.637173	1.152067	-0.66397	TFF3
eldeline-6026	1/100	0.561955	0.561955	0.637173	0.637173	1.152199	-0.66405	DCN
ethisterone-4340	1/100	0.561955	0.561955	0.637173	0.637173	1.152889	-0.66445	SERPINA1
drofenine-6776	1/100	0.561955	0.561955	0.637173	0.637173	1.152942	-0.66448	FOS
estradiol-2701	1/100	0.561955	0.561955	0.637173	0.637173	1.153123	-0.66458	COL1A2
estradiol-1241	1/100	0.561955	0.561955	0.637173	0.637173	1.155913	-0.66619	LYZ
dyclonine-7261	1/100	0.561955	0.561955	0.637173	0.637173	1.156142	-0.66632	AP1S1
eldeline-3925	1/100	0.561955	0.561955	0.637173	0.637173	1.157144	-0.6669	COL1A1
epitiostanol-4788	1/100	0.561955	0.561955	0.637173	0.637173	1.158203	-0.66751	COL1A1
epitiostanol-7342	1/100	0.561955	0.561955	0.637173	0.637173	1.158705	-0.6678	TMPRSS3
etiocholanolone-2204	1/100	0.561955	0.561955	0.637173	0.637173	1.160364	-0.66876	TCN1
epivincamine-1783	1/100	0.561955	0.561955	0.637173	0.637173	1.16288	-0.67021	CEACAM6
estradiol-5955	1/100	0.561955	0.561955	0.637173	0.637173	1.163355	-0.67048	SECTM1
erythromycin-2010	1/100	0.561955	0.561955	0.637173	0.637173	1.163665	-0.67066	GPX2
trichostatin A-7077	2/100	0.197972	0.408923	0.269513	0.556697	0.414161	-0.67079	MMP1;CTGF
erastin-6412	1/100	0.561955	0.561955	0.637173	0.637173	1.16391	-0.6708	APP
ebselen-5778	1/100	0.561955	0.561955	0.637173	0.637173	1.16524	-0.67157	FOS
tyloxapol-5672	2/100	0.197972	0.408923	0.269513	0.556697	0.415053	-0.67223	COL1A1;MMP3
dopamine-491	1/100	0.561955	0.561955	0.637173	0.637173	1.166408	-0.67224	IFITM1
valinomycin-5906	2/100	0.197972	0.408923	0.269513	0.556697	0.415973	-0.67372	TFF2;ETS2
esculetin-6499	1/100	0.561955	0.561955	0.637173	0.637173	1.169104	-0.67379	AP1S1
estradiol-1633	1/100	0.561955	0.561955	0.637173	0.637173	1.169504	-0.67402	TFF2
estradiol-4432	1/100	0.561955	0.561955	0.637173	0.637173	1.170538	-0.67462	CCL20
esculetin-3120	1/100	0.561955	0.561955	0.637173	0.637173	1.170968	-0.67487	MMP1
tyloxapol-6452	2/100	0.197972	0.408923	0.269513	0.556697	0.416905	-0.67523	MMP3;DUOX2
estradiol-365	1/100	0.561955	0.561955	0.637173	0.637173	1.172055	-0.67549	COL1A2
estradiol-121	1/100	0.561955	0.561955	0.637173	0.637173	1.17249	-0.67575	GPX2
erastin-6364	1/100	0.561955	0.561955	0.637173	0.637173	1.172497	-0.67575	GPX2
troglitazone-6949	2/100	0.197972	0.408923	0.269513	0.556697	0.417292	-0.67586	SOX9;S100A11
estradiol-1666	1/100	0.561955	0.561955	0.637173	0.637173	1.174588	-0.67695	COL1A1
tyloxapol-3074	2/100	0.197972	0.408923	0.269513	0.556697	0.417974	-0.67696	COL1A1;TMPRSS3
vanoxerine-4641	2/100	0.197972	0.408923	0.269513	0.556697	0.418681	-0.67811	CXCL1;FOS
ethosuximide-2280	1/100	0.561955	0.561955	0.637173	0.637173	1.176903	-0.67829	IFI6
ethosuximide-7308	1/100	0.561955	0.561955	0.637173	0.637173	1.177804	-0.67881	IFI6
ethionamide-2495	1/100	0.561955	0.561955	0.637173	0.637173	1.179402	-0.67973	COL1A1
erastin-6417	1/100	0.561955	0.561955	0.637173	0.637173	1.183081	-0.68185	AP1S1
estriol-5866	1/100	0.561955	0.561955	0.637173	0.637173	1.184745	-0.68281	AP1S1

valinomycin-5962	2/100	0.197972	0.408923	0.269513	0.556697	0.42197	-0.68344	S100P;PHLDA1
DL-PPMP-1121	1/100	0.561955	0.561955	0.637173	0.637173	1.186139	-0.68361	PHLDA1
estrone-3071	1/100	0.561955	0.561955	0.637173	0.637173	1.186385	-0.68375	IFITM1
etamsylate-4399	1/100	0.561955	0.561955	0.637173	0.637173	1.187966	-0.68466	CEACAM5
estradiol-6928	1/100	0.561955	0.561955	0.637173	0.637173	1.18874	-0.68511	SERPINA1
etanidazole-6072	1/100	0.561955	0.561955	0.637173	0.637173	1.18922	-0.68539	RNF43
etacrynic acid-5742	1/100	0.561955	0.561955	0.637173	0.637173	1.189788	-0.68571	MMP7
etiocholanolone-6060	1/100	0.561955	0.561955	0.637173	0.637173	1.189822	-0.68573	SOX9
vorinostat-1645	2/100	0.197972	0.408923	0.269513	0.556697	0.42414	-0.68695	TXNIP;ISG15
etofylline-2256	1/100	0.561955	0.561955	0.637173	0.637173	1.191978	-0.68698	PSMB9
eucatropine-3416	1/100	0.561955	0.561955	0.637173	0.637173	1.192175	-0.68709	COL1A1
valproic acid-1214	2/100	0.197972	0.408923	0.269513	0.556697	0.424506	-0.68754	AP1S1;PSMB9
estropipate-6068	1/100	0.561955	0.561955	0.637173	0.637173	1.193343	-0.68776	IFI6
ethisterone-2366	1/100	0.561955	0.561955	0.637173	0.637173	1.193884	-0.68808	SLCO1B3
ethambutol-4001	1/100	0.561955	0.561955	0.637173	0.637173	1.196196	-0.68941	CEACAM6
valproic acid-1240	2/100	0.197972	0.408923	0.269513	0.556697	0.42603	-0.69001	LYZ;PSMB9
valproic acid-6929	2/100	0.197972	0.408923	0.269513	0.556697	0.426242	-0.69036	IFI6;SOX9
etilefrine-7350	1/100	0.561955	0.561955	0.637173	0.637173	1.19791	-0.6904	SOX9
ethoxyquin-3846	1/100	0.561955	0.561955	0.637173	0.637173	1.199251	-0.69117	COL1A1
esculin-6310	1/100	0.561955	0.561955	0.637173	0.637173	1.199836	-0.69151	TSPAN1
enoxacin-5616	1/100	0.561955	0.561955	0.637173	0.637173	1.200501	-0.69189	PHLDA1
trimethylcolchicinic acid-2146	2/100	0.197972	0.408923	0.269513	0.556697	0.427218	-0.69194	PDZK1IP1;PHLDA1
flunarizine-2381	1/100	0.561955	0.561955	0.637173	0.637173	1.200827	-0.69208	APP
F0447-0125-6429	1/100	0.561955	0.561955	0.637173	0.637173	1.200961	-0.69215	AP1S1
etilefrine-2930	1/100	0.561955	0.561955	0.637173	0.637173	1.201974	-0.69274	ID3
etynodiol-6479	1/100	0.561955	0.561955	0.637173	0.637173	1.20218	-0.69286	COL1A1
ethaverine-5337	1/100	0.561955	0.561955	0.637173	0.637173	1.202378	-0.69297	CEACAM6
exemestane-165	1/100	0.561955	0.561955	0.637173	0.637173	1.204602	-0.69425	CEACAM5
ursolic acid-2230	2/100	0.197972	0.408923	0.269513	0.556697	0.42938	-0.69544	FOS;PHLDA1
valproic acid-2674	2/100	0.197972	0.408923	0.269513	0.556697	0.429493	-0.69562	TCN1;PRSS23
withaferin A-3819	2/100	0.197972	0.408923	0.269513	0.556697	0.429553	-0.69572	JUND;HSPB1
fenoprofen-2553	1/100	0.561955	0.561955	0.637173	0.637173	1.207922	-0.69617	IFITM1
vinburnine-6802	2/100	0.197972	0.408923	0.269513	0.556697	0.429909	-0.69629	IFI27;CTGF
wortmannin-7002	2/100	0.197972	0.408923	0.269513	0.556697	0.430248	-0.69684	IFITM1;CEACAM5
vorinostat-1220	2/100	0.197972	0.408923	0.269513	0.556697	0.430924	-0.69794	MMP1;CTGF
etofenamate-3989	1/100	0.561955	0.561955	0.637173	0.637173	1.211295	-0.69811	SERPINA1
valproic acid-6181	2/100	0.197972	0.408923	0.269513	0.556697	0.431197	-0.69838	COL1A1;GPX2

ethambutol-1981	1/100	0.561955	0.561955	0.637173	0.637173	1.213106	-0.69915	CEACAM5
fluorocurarine-6083	1/100	0.561955	0.561955	0.637173	0.637173	1.215862	-0.70074	SPARC
fipexide-3176	1/100	0.561955	0.561955	0.637173	0.637173	1.217212	-0.70152	SERPINA1
fenbendazole-4542	1/100	0.561955	0.561955	0.637173	0.637173	1.21841	-0.70221	JUND
valproic acid-1222	2/100	0.197972	0.408923	0.269513	0.556697	0.43374	-0.7025	IFITM1;JUND
estriol-6140	1/100	0.561955	0.561955	0.637173	0.637173	1.220048	-0.70315	TGFBI
eticlopride-5695	1/100	0.561955	0.561955	0.637173	0.637173	1.220894	-0.70364	MMP3
ethotoin-4366	1/100	0.561955	0.561955	0.637173	0.637173	1.220934	-0.70367	AP1S1
wortmannin-911	2/100	0.197972	0.408923	0.269513	0.556697	0.434643	-0.70396	TXNIP;TSPAN1
famotidine-1529	1/100	0.561955	0.561955	0.637173	0.637173	1.222502	-0.70457	CEACAM5
ethosuximide-1433	1/100	0.561955	0.561955	0.637173	0.637173	1.222962	-0.70483	IFITM3
vorinostat-6939	2/100	0.197972	0.408923	0.269513	0.556697	0.435226	-0.70491	TXNIP;SOX9
fendiline-1573	1/100	0.561955	0.561955	0.637173	0.637173	1.224279	-0.70559	CCL20
F0447-0125-6424	1/100	0.561955	0.561955	0.637173	0.637173	1.228538	-0.70805	CEACAM6
florfenicol-6701	1/100	0.561955	0.561955	0.637173	0.637173	1.228745	-0.70817	TSPAN1
fenoprofen-3612	1/100	0.561955	0.561955	0.637173	0.637173	1.230441	-0.70914	IFI6
zaprinast-3226	2/100	0.197972	0.408923	0.269513	0.556697	0.438628	-0.71042	SERPINA1;S100A11
etamivan-7260	1/100	0.561955	0.561955	0.637173	0.637173	1.232873	-0.71055	APP
estriol-3563	1/100	0.561955	0.561955	0.637173	0.637173	1.233611	-0.71097	CEACAM5
fenoprofen-4736	1/100	0.561955	0.561955	0.637173	0.637173	1.233685	-0.71101	COL1A1
wortmannin-6959	2/100	0.197972	0.408923	0.269513	0.556697	0.439067	-0.71113	IFITM1;IFI6
flucytosine-5289	1/100	0.561955	0.561955	0.637173	0.637173	1.234287	-0.71136	S100A4
flecainide-3761	1/100	0.561955	0.561955	0.637173	0.637173	1.236913	-0.71287	PTPRO
flumetasone-3410	1/100	0.561955	0.561955	0.637173	0.637173	1.23723	-0.71306	CEACAM6
flucytosine-3073	1/100	0.561955	0.561955	0.637173	0.637173	1.237488	-0.71321	FXYD3
flavoxate-6326	1/100	0.561955	0.561955	0.637173	0.637173	1.238808	-0.71397	COL1A1
fenoterol-5432	1/100	0.561955	0.561955	0.637173	0.637173	1.238877	-0.71401	RCN1
famprofazone-6029	1/100	0.561955	0.561955	0.637173	0.637173	1.239494	-0.71436	SPP1
fenoterol-2378	1/100	0.561955	0.561955	0.637173	0.637173	1.240883	-0.71516	APP
felbinac-3061	1/100	0.561955	0.561955	0.637173	0.637173	1.241435	-0.71548	COL1A1
fluspirilene-5008	1/100	0.561955	0.561955	0.637173	0.637173	1.241594	-0.71557	FOS
etamsylate-2915	1/100	0.561955	0.561955	0.637173	0.637173	1.241726	-0.71565	FXYD3
vorinostat-1161	2/100	0.197972	0.408923	0.269513	0.556697	0.442444	-0.7166	TSPAN13;ID3
vincamine-2327	2/100	0.197972	0.408923	0.269513	0.556697	0.442526	-0.71673	TCN1;PTPRO
fludroxycortide-3679	1/100	0.561955	0.561955	0.637173	0.637173	1.244147	-0.71704	IL32
xylometazoline-2107	2/100	0.197972	0.408923	0.269513	0.556697	0.442815	-0.7172	GPX2;S100A11
felodipine-2433	1/100	0.561955	0.561955	0.637173	0.637173	1.245673	-0.71792	IFITM1

flunisolide-4303	1/100	0.561955	0.561955	0.637173	0.637173	1.245723	-0.71795	PHLDA1
fluvastatin-6691	1/100	0.561955	0.561955	0.637173	0.637173	1.245965	-0.71809	JUND
flutamide-2358	1/100	0.561955	0.561955	0.637173	0.637173	1.246998	-0.71869	GPX2
fluphenazine-7234	1/100	0.561955	0.561955	0.637173	0.637173	1.249123	-0.71991	PHLDA1
wortmannin-1081	2/100	0.197972	0.408923	0.269513	0.556697	0.444595	-0.72008	IFITM1;TXNIP
flucloxacillin-5102	1/100	0.561955	0.561955	0.637173	0.637173	1.249648	-0.72021	MMP3
zidovudine-6733	2/100	0.197972	0.408923	0.269513	0.556697	0.445117	-0.72092	C3;AP1S1
W-13-440	2/100	0.197972	0.408923	0.269513	0.556697	0.445145	-0.72097	JUND;CKB
TTNPB-223	2/100	0.197972	0.408923	0.269513	0.556697	0.445418	-0.72141	SOX9;PHLDA1
flucloxacillin-6507	1/100	0.561955	0.561955	0.637173	0.637173	1.251946	-0.72154	CTSE
fenspiride-1422	1/100	0.561955	0.561955	0.637173	0.637173	1.251949	-0.72154	AP1S1
fluorocurarine-6219	1/100	0.561955	0.561955	0.637173	0.637173	1.252282	-0.72173	IFITM1
flunarizine-7412	1/100	0.561955	0.561955	0.637173	0.637173	1.252637	-0.72194	CEACAM6
felodipine-337	1/100	0.561955	0.561955	0.637173	0.637173	1.256663	-0.72426	PHLDA1
flunisolide-6023	1/100	0.561955	0.561955	0.637173	0.637173	1.256909	-0.7244	DCN
fluocinonide-3414	1/100	0.561955	0.561955	0.637173	0.637173	1.257675	-0.72484	RNF43
fluocinonide-4314	1/100	0.561955	0.561955	0.637173	0.637173	1.258001	-0.72503	JUND
fasudil-436	1/100	0.561955	0.561955	0.637173	0.637173	1.259684	-0.726	AP1S1
vorinostat-1058	2/100	0.197972	0.408923	0.269513	0.556697	0.448293	-0.72607	TXNIP;ISG15
ethotoin-3892	1/100	0.561955	0.561955	0.637173	0.637173	1.26	-0.72618	CTSE
vincamine-4341	2/100	0.197972	0.408923	0.269513	0.556697	0.448714	-0.72675	ENC1;CXCL1
yohimbine-1119	2/100	0.197972	0.408923	0.269513	0.556697	0.449053	-0.7273	APP;ID3
flufenamic acid-316	1/100	0.561955	0.561955	0.637173	0.637173	1.262177	-0.72743	IFI6
fluspirilene-3086	1/100	0.561955	0.561955	0.637173	0.637173	1.262586	-0.72767	CEACAM6
flumetasone-3610	1/100	0.561955	0.561955	0.637173	0.637173	1.263046	-0.72794	IFI27
fluphenazine-1577	1/100	0.561955	0.561955	0.637173	0.637173	1.263362	-0.72812	TXNIP
flucloxacillin-3128	1/100	0.561955	0.561955	0.637173	0.637173	1.264434	-0.72874	IFITM1
finasteride-2206	1/100	0.561955	0.561955	0.637173	0.637173	1.26552	-0.72936	GPX2
fipexide-1560	1/100	0.561955	0.561955	0.637173	0.637173	1.266593	-0.72998	IFITM1
flunisolide-3923	1/100	0.561955	0.561955	0.637173	0.637173	1.267056	-0.73025	COL1A1
flumequine-5104	1/100	0.561955	0.561955	0.637173	0.637173	1.267706	-0.73062	JUND
wortmannin-5603	2/100	0.197972	0.408923	0.269513	0.556697	0.451366	-0.73105	SPINK1;TCN1
foliosidine-6057	1/100	0.561955	0.561955	0.637173	0.637173	1.268783	-0.73124	TCN1
yohimbic acid-2803	2/100	0.197972	0.408923	0.269513	0.556697	0.451694	-0.73158	MMP3;S100A6
fulvestrant-5565	1/100	0.561955	0.561955	0.637173	0.637173	1.27045	-0.7322	ID1
fluvoxamine-2913	1/100	0.561955	0.561955	0.637173	0.637173	1.270772	-0.73239	JUND
fenoprofen-3412	1/100	0.561955	0.561955	0.637173	0.637173	1.271022	-0.73253	C3

zimeldine-1512	2/100	0.197972	0.408923	0.269513	0.556697	0.452623	-0.73308	AP1S1;SECTM1
furosemide-6841	1/100	0.561955	0.561955	0.637173	0.637173	1.274845	-0.73474	TFF2
valproic acid-1209	2/100	0.197972	0.408923	0.269513	0.556697	0.454025	-0.73535	LUM;PSMB9
valproic acid-1634	2/100	0.197972	0.408923	0.269513	0.556697	0.454951	-0.73685	C3;PGM1
flucloxacillin-5527	1/100	0.561955	0.561955	0.637173	0.637173	1.278768	-0.737	AP1S1
velnacrine-2430	2/100	0.197972	0.408923	0.269513	0.556697	0.455542	-0.73781	COL1A1;AP1S1
fluticasone-4011	1/100	0.561955	0.561955	0.637173	0.637173	1.281303	-0.73846	QPCT
flurbiprofen-3095	1/100	0.561955	0.561955	0.637173	0.637173	1.283357	-0.73964	IFITM1
folic acid-7201	1/100	0.561955	0.561955	0.637173	0.637173	1.284575	-0.74034	S100A11
zidovudine-5333	2/100	0.197972	0.408923	0.269513	0.556697	0.457119	-0.74036	SERPINA1;CEACAM6
flecainide-3843	1/100	0.561955	0.561955	0.637173	0.637173	1.284616	-0.74037	COL1A1
fulvestrant-5235	1/100	0.561955	0.561955	0.637173	0.637173	1.284736	-0.74044	ID1
fluoxetine-2453	1/100	0.561955	0.561955	0.637173	0.637173	1.285105	-0.74065	JUND
fluphenazine-6996	1/100	0.561955	0.561955	0.637173	0.637173	1.289332	-0.74309	PHLDA1
fulvestrant-1205	1/100	0.561955	0.561955	0.637173	0.637173	1.289516	-0.74319	AP1S1
furazolidone-4098	1/100	0.561955	0.561955	0.637173	0.637173	1.289591	-0.74323	SPINK1
zuclopenthixol-2936	2/100	0.197972	0.408923	0.269513	0.556697	0.45894	-0.74331	IFITM1;IGFBP2
fluocinonide-3757	1/100	0.561955	0.561955	0.637173	0.637173	1.289874	-0.7434	MMP7
zardaverine-2926	2/100	0.197972	0.408923	0.269513	0.556697	0.459013	-0.74343	ID3;TIMP1
fulvestrant-5202	1/100	0.561955	0.561955	0.637173	0.637173	1.29112	-0.74412	ID1
folic acid-2783	1/100	0.561955	0.561955	0.637173	0.637173	1.291806	-0.74451	AP1S1
galantamine-2787	1/100	0.561955	0.561955	0.637173	0.637173	1.292395	-0.74485	COL1A1
fulvestrant-6955	1/100	0.561955	0.561955	0.637173	0.637173	1.293064	-0.74524	SERPINA1
gabapentin-3472	1/100	0.561955	0.561955	0.637173	0.637173	1.293518	-0.7455	COL1A1
fluphenazine-1662	1/100	0.561955	0.561955	0.637173	0.637173	1.293519	-0.7455	JUND
withaferin A-4376	2/100	0.197972	0.408923	0.269513	0.556697	0.460984	-0.74662	PHLDA1;ETS2
flufenamic acid-1420	1/100	0.561955	0.561955	0.637173	0.637173	1.299141	-0.74874	GPX2
geldanamycin-864	1/100	0.561955	0.561955	0.637173	0.637173	1.300519	-0.74953	COL1A1
fulvestrant-6872	1/100	0.561955	0.561955	0.637173	0.637173	1.300754	-0.74967	APP
gabapentin-5875	1/100	0.561955	0.561955	0.637173	0.637173	1.301821	-0.75028	DCN
gossypol-4762	1/100	0.561955	0.561955	0.637173	0.637173	1.30343	-0.75121	TXNIP
zaprinast-6749	2/100	0.197972	0.408923	0.269513	0.556697	0.464153	-0.75176	C3;SERPINA1
fulvestrant-367	1/100	0.561955	0.561955	0.637173	0.637173	1.305001	-0.75212	COL1A2
genistein-6994	1/100	0.561955	0.561955	0.637173	0.637173	1.310772	-0.75544	S100A11
genistein-6952	1/100	0.561955	0.561955	0.637173	0.637173	1.310867	-0.7555	S100A11
furazolidone-4178	1/100	0.561955	0.561955	0.637173	0.637173	1.311363	-0.75578	CEACAM5
foliosidine-3636	1/100	0.561955	0.561955	0.637173	0.637173	1.313956	-0.75728	SERPINA1

gemfibrozil-5069	1/100	0.561955	0.561955	0.637173	0.637173	1.31429	-0.75747	JUND
galantamine-4772	1/100	0.561955	0.561955	0.637173	0.637173	1.314347	-0.7575	SPINK1
fulvestrant-1146	1/100	0.561955	0.561955	0.637173	0.637173	1.31549	-0.75816	CCL20
fulvestrant-7490	1/100	0.561955	0.561955	0.637173	0.637173	1.315984	-0.75845	COL1A1
geldanamycin-972	1/100	0.561955	0.561955	0.637173	0.637173	1.31669	-0.75885	BMP4
gliclazide-5089	1/100	0.561955	0.561955	0.637173	0.637173	1.318665	-0.75999	SPINK1
genistein-267	1/100	0.561955	0.561955	0.637173	0.637173	1.318758	-0.76004	IFI27
fusaric acid-4105	1/100	0.561955	0.561955	0.637173	0.637173	1.319789	-0.76064	DCN
fulvestrant-704	1/100	0.561955	0.561955	0.637173	0.637173	1.320807	-0.76123	PLCB4
glafenine-7018	1/100	0.561955	0.561955	0.637173	0.637173	1.321904	-0.76186	SOX9
guaifenesin-4549	1/100	0.561955	0.561955	0.637173	0.637173	1.322608	-0.76226	DCN
fulvestrant-7495	1/100	0.561955	0.561955	0.637173	0.637173	1.323642	-0.76286	S100A11
fulvestrant-7091	1/100	0.561955	0.561955	0.637173	0.637173	1.324098	-0.76312	COL1A1
gibberellic acid-4818	1/100	0.561955	0.561955	0.637173	0.637173	1.325111	-0.76371	MMP3
guaifenesin-3431	1/100	0.561955	0.561955	0.637173	0.637173	1.328197	-0.76548	AP1S1
fulvestrant-523	1/100	0.561955	0.561955	0.637173	0.637173	1.32849	-0.76565	S100A4
fulvestrant-6918	1/100	0.561955	0.561955	0.637173	0.637173	1.328521	-0.76567	CEACAM6
gramine-2143	1/100	0.561955	0.561955	0.637173	0.637173	1.330729	-0.76694	APP
galantamine-2131	1/100	0.561955	0.561955	0.637173	0.637173	1.331586	-0.76744	IFI6
genistein-1015	1/100	0.561955	0.561955	0.637173	0.637173	1.334747	-0.76926	ETS2
halofantrine-6509	1/100	0.561955	0.561955	0.637173	0.637173	1.335061	-0.76944	IFI6
gliclazide-5514	1/100	0.561955	0.561955	0.637173	0.637173	1.336203	-0.7701	IFI6
guanadrel-4720	1/100	0.561955	0.561955	0.637173	0.637173	1.338041	-0.77116	SECTM1
geldanamycin-6187	1/100	0.561955	0.561955	0.637173	0.637173	1.338059	-0.77117	HSPB1
ginkgolide A-4121	1/100	0.561955	0.561955	0.637173	0.637173	1.338265	-0.77129	SECTM1
fulvestrant-310	1/100	0.561955	0.561955	0.637173	0.637173	1.33842	-0.77138	ID3
fusidic acid-2647	1/100	0.561955	0.561955	0.637173	0.637173	1.338442	-0.77139	SLPI
Gly-His-Lys-6560	1/100	0.561955	0.561955	0.637173	0.637173	1.339994	-0.77228	GAPDH
gentamicin-2082	1/100	0.561955	0.561955	0.637173	0.637173	1.342166	-0.77354	BMP4
gabexate-4804	1/100	0.561955	0.561955	0.637173	0.637173	1.342863	-0.77394	IFI6
glimepiride-2154	1/100	0.561955	0.561955	0.637173	0.637173	1.344768	-0.77504	IFI6
haloperidol-4678	1/100	0.561955	0.561955	0.637173	0.637173	1.345719	-0.77558	COL1A1
gliquidone-6004	1/100	0.561955	0.561955	0.637173	0.637173	1.346395	-0.77597	CEACAM5
fusaric acid-3986	1/100	0.561955	0.561955	0.637173	0.637173	1.34738	-0.77654	CEACAM6
Gly-His-Lys-6575	1/100	0.561955	0.561955	0.637173	0.637173	1.348704	-0.7773	LGALS3BP
fusidic acid-5353	1/100	0.561955	0.561955	0.637173	0.637173	1.348993	-0.77747	CEACAM6
glibenclamide-1546	1/100	0.561955	0.561955	0.637173	0.637173	1.349045	-0.7775	TFF1

guaifenesin-3814	1/100	0.561955	0.561955	0.637173	0.637173	1.349298	-0.77765	JUND
xylometazoline-2270	2/100	0.197972	0.408923	0.269513	0.556697	0.480294	-0.7779	COL1A1;LYZ
glycopyrronium bromide-7386	1/100	0.561955	0.561955	0.637173	0.637173	1.35053	-0.77836	DCN
glafenine-7418	1/100	0.561955	0.561955	0.637173	0.637173	1.35135	-0.77883	CEACAM5
gemfibrozil-5488	1/100	0.561955	0.561955	0.637173	0.637173	1.352464	-0.77947	TMPRSS3
genistein-1235	1/100	0.561955	0.561955	0.637173	0.637173	1.35344	-0.78003	COL1A2
haloperidol-1628	1/100	0.561955	0.561955	0.637173	0.637173	1.354311	-0.78054	TMPRSS3
haloperidol-1041	1/100	0.561955	0.561955	0.637173	0.637173	1.355813	-0.7814	CD24
guanadrel-3438	1/100	0.561955	0.561955	0.637173	0.637173	1.358259	-0.78281	AP1S1
harmaline-4968	1/100	0.561955	0.561955	0.637173	0.637173	1.362683	-0.78536	PTPRO
gemfibrozil-1430	1/100	0.561955	0.561955	0.637173	0.637173	1.362969	-0.78552	CKB
gentamicin-5883	1/100	0.561955	0.561955	0.637173	0.637173	1.363904	-0.78606	TSPAN8
geldanamycin-1008	1/100	0.561955	0.561955	0.637173	0.637173	1.364206	-0.78624	TXNIP
vinburnine-7154	2/100	0.197972	0.408923	0.269513	0.556697	0.485991	-0.78713	CXCL1;CTGF
geldanamycin-593	1/100	0.561955	0.561955	0.637173	0.637173	1.365873	-0.7872	SLPI
gossypol-6058	1/100	0.561955	0.561955	0.637173	0.637173	1.365962	-0.78725	PHLDA1
hecogenin-7175	1/100	0.561955	0.561955	0.637173	0.637173	1.365972	-0.78726	CEACAM5
heliotrine-3717	1/100	0.561955	0.561955	0.637173	0.637173	1.366293	-0.78744	APP
harpagoside-2935	1/100	0.561955	0.561955	0.637173	0.637173	1.368316	-0.78861	IGFBP2
hesperidin-2648	1/100	0.561955	0.561955	0.637173	0.637173	1.37023	-0.78971	CEACAM5
genistein-268	1/100	0.561955	0.561955	0.637173	0.637173	1.371921	-0.79068	IFI27
harmine-1758	1/100	0.561955	0.561955	0.637173	0.637173	1.372716	-0.79114	SPP1
gossypol-2202	1/100	0.561955	0.561955	0.637173	0.637173	1.374062	-0.79192	PHLDA1
guanfacine-4660	1/100	0.561955	0.561955	0.637173	0.637173	1.375036	-0.79248	CEACAM5
genistein-703	1/100	0.561955	0.561955	0.637173	0.637173	1.375621	-0.79282	ENC1
harmaline-6623	1/100	0.561955	0.561955	0.637173	0.637173	1.377387	-0.79383	C3
H-89-6921	1/100	0.561955	0.561955	0.637173	0.637173	1.377935	-0.79415	COL1A1
haloperidol-983	1/100	0.561955	0.561955	0.637173	0.637173	1.378922	-0.79472	RNF43
gramine-3999	1/100	0.561955	0.561955	0.637173	0.637173	1.378982	-0.79475	CEACAM6
hesperetin-1531	1/100	0.561955	0.561955	0.637173	0.637173	1.379771	-0.79521	PHLDA1
guanabenz-1961	1/100	0.561955	0.561955	0.637173	0.637173	1.380998	-0.79592	LY6E
haloperidol-5604	1/100	0.561955	0.561955	0.637173	0.637173	1.381081	-0.79596	IFI6
hemicholinium-1601	1/100	0.561955	0.561955	0.637173	0.637173	1.382242	-0.79663	TPI1
zimeldine-2012	2/100	0.197972	0.408923	0.269513	0.556697	0.492056	-0.79695	NQO1;GPX2
glycopyrronium bromide-3427	1/100	0.561955	0.561955	0.637173	0.637173	1.386476	-0.79907	CEACAM5
guanethidine-1554	1/100	0.561955	0.561955	0.637173	0.637173	1.386932	-0.79934	TPI1
ikarugamycin-918	1/100	0.561955	0.561955	0.637173	0.637173	1.390212	-0.80123	JUND

hemicholinium-3216	1/100	0.561955	0.561955	0.637173	0.637173	1.39028	-0.80126	LYZ
hexestrol-5776	1/100	0.561955	0.561955	0.637173	0.637173	1.392339	-0.80245	C3
hexamethonium bromide-1482	1/100	0.561955	0.561955	0.637173	0.637173	1.394448	-0.80367	COL1A1
harmine-5855	1/100	0.561955	0.561955	0.637173	0.637173	1.394548	-0.80372	COL1A1
indapamide-4335	1/100	0.561955	0.561955	0.637173	0.637173	1.395406	-0.80422	IL32
heptaminol-6015	1/100	0.561955	0.561955	0.637173	0.637173	1.395999	-0.80456	PHLDA1
homosalate-4355	1/100	0.561955	0.561955	0.637173	0.637173	1.39756	-0.80546	CEACAM5
hecogenin-3457	1/100	0.561955	0.561955	0.637173	0.637173	1.39757	-0.80547	SOX9
hydrastine hydrochloride-2889	1/100	0.561955	0.561955	0.637173	0.637173	1.397797	-0.8056	COL1A1
haloperidol-1185	1/100	0.561955	0.561955	0.637173	0.637173	1.397881	-0.80565	APP
homatropine-5058	1/100	0.561955	0.561955	0.637173	0.637173	1.398276	-0.80587	SPP1
heliotrine-6035	1/100	0.561955	0.561955	0.637173	0.637173	1.398288	-0.80588	IFI6
haloperidol-1082	1/100	0.561955	0.561955	0.637173	0.637173	1.399038	-0.80631	ETS2
homatropine-1684	1/100	0.561955	0.561955	0.637173	0.637173	1.401142	-0.80753	S100A11
hecogenin-2716	1/100	0.561955	0.561955	0.637173	0.637173	1.401542	-0.80776	DSG2
halcinonide-2185	1/100	0.561955	0.561955	0.637173	0.637173	1.401769	-0.80789	CCL20
hydrastinine-1436	1/100	0.561955	0.561955	0.637173	0.637173	1.403535	-0.8089	PTMA
hesperidin-1294	1/100	0.561955	0.561955	0.637173	0.637173	1.406376	-0.81054	S100A11
hydrastine hydrochloride-6011	1/100	0.561955	0.561955	0.637173	0.637173	1.409891	-0.81257	ITM2C
hydroxyzine-2024	1/100	0.561955	0.561955	0.637173	0.637173	1.411465	-0.81347	GPX2
harpagoside-6636	1/100	0.561955	0.561955	0.637173	0.637173	1.412384	-0.814	MMP3
hydroxyzine-5006	1/100	0.561955	0.561955	0.637173	0.637173	1.413088	-0.81441	CEACAM5
guanfacine-1279	1/100	0.561955	0.561955	0.637173	0.637173	1.413096	-0.81441	APP
hydroxyachillin-4213	1/100	0.561955	0.561955	0.637173	0.637173	1.41311	-0.81442	IFITM1
hexylcaine-3447	1/100	0.561955	0.561955	0.637173	0.637173	1.414719	-0.81535	RCN1
hexylcaine-5768	1/100	0.561955	0.561955	0.637173	0.637173	1.414959	-0.81549	DCN
indapamide-3778	1/100	0.561955	0.561955	0.637173	0.637173	1.419208	-0.81794	PIGR
hesperetin-2031	1/100	0.561955	0.561955	0.637173	0.637173	1.419511	-0.81811	NQO1
haloperidol-6960	1/100	0.561955	0.561955	0.637173	0.637173	1.419927	-0.81835	ACSL1
hydroquinine-6263	1/100	0.561955	0.561955	0.637173	0.637173	1.419939	-0.81836	APP
iloprost-427	1/100	0.561955	0.561955	0.637173	0.637173	1.420555	-0.81871	DCN
haloperidol-1244	1/100	0.561955	0.561955	0.637173	0.637173	1.420699	-0.8188	TSPAN8
hydroflumethiazide-1851	1/100	0.561955	0.561955	0.637173	0.637173	1.421597	-0.81931	CTSH
hydrastinine-5494	1/100	0.561955	0.561955	0.637173	0.637173	1.421892	-0.81948	TFF2
hesperetin-6750	1/100	0.561955	0.561955	0.637173	0.637173	1.423795	-0.82058	LYZ
hexetidine-2457	1/100	0.561955	0.561955	0.637173	0.637173	1.423811	-0.82059	ISG15
haloperidol-6923	1/100	0.561955	0.561955	0.637173	0.637173	1.423932	-0.82066	SOX9

hydrocotarnine-4489	1/100	0.561955	0.561955	0.637173	0.637173	1.425623	-0.82163	DCN
ifenprodil-5463	1/100	0.561955	0.561955	0.637173	0.637173	1.425941	-0.82182	SOX9
homochlorcyclizine-2386	1/100	0.561955	0.561955	0.637173	0.637173	1.428988	-0.82357	SPP1
hydroquinine-5789	1/100	0.561955	0.561955	0.637173	0.637173	1.431669	-0.82512	PROM1
hydrochlorothiazide-6625	1/100	0.561955	0.561955	0.637173	0.637173	1.431985	-0.8253	MMP3
hydrocortisone-3284	1/100	0.561955	0.561955	0.637173	0.637173	1.434044	-0.82649	COL1A1
hydroxyzine-6660	1/100	0.561955	0.561955	0.637173	0.637173	1.434555	-0.82678	COL1A2
hydroxyzine-1941	1/100	0.561955	0.561955	0.637173	0.637173	1.435213	-0.82716	AP1S1
iopanoic acid-6348	1/100	0.561955	0.561955	0.637173	0.637173	1.435832	-0.82752	AP1S1
homosalate-3797	1/100	0.561955	0.561955	0.637173	0.637173	1.437052	-0.82822	IL32
imipenem-2873	1/100	0.561955	0.561955	0.637173	0.637173	1.43805	-0.8288	ITM2C
iproniazid-1442	1/100	0.561955	0.561955	0.637173	0.637173	1.440044	-0.82995	PTMA
hyoscyamine-5524	1/100	0.561955	0.561955	0.637173	0.637173	1.440468	-0.83019	MMP7
hymecromone-3383	1/100	0.561955	0.561955	0.637173	0.637173	1.441706	-0.8309	CTGF
ifosfamide-5805	1/100	0.561955	0.561955	0.637173	0.637173	1.442226	-0.8312	JUND
hydroquinine-1775	1/100	0.561955	0.561955	0.637173	0.637173	1.442535	-0.83138	TCN1
hydroflumethiazide-7019	1/100	0.561955	0.561955	0.637173	0.637173	1.442783	-0.83152	CEACAM5
idazoxan-6465	1/100	0.561955	0.561955	0.637173	0.637173	1.443707	-0.83206	BMP4
ioxaglic acid-3528	1/100	0.561955	0.561955	0.637173	0.637173	1.444183	-0.83233	COL1A1
hydralazine-3724	1/100	0.561955	0.561955	0.637173	0.637173	1.444193	-0.83234	MMP7
hycanthone-3229	1/100	0.561955	0.561955	0.637173	0.637173	1.445198	-0.83292	AP1S1
isosorbide-3720	1/100	0.561955	0.561955	0.637173	0.637173	1.446071	-0.83342	SPARC
kawain-2337	1/100	0.561955	0.561955	0.637173	0.637173	1.450494	-0.83597	COL1A1
hesperetin-5350	1/100	0.561955	0.561955	0.637173	0.637173	1.453729	-0.83783	SOX9
indometacin-262	1/100	0.561955	0.561955	0.637173	0.637173	1.455831	-0.83904	ID3
hexamethonium bromide-1901	1/100	0.561955	0.561955	0.637173	0.637173	1.455986	-0.83913	CD24
hydroxyachillin-2157	1/100	0.561955	0.561955	0.637173	0.637173	1.458294	-0.84046	IFI6
iobenguane-2878	1/100	0.561955	0.561955	0.637173	0.637173	1.459225	-0.841	COL1A1
indometacin-5468	1/100	0.561955	0.561955	0.637173	0.637173	1.46014	-0.84153	TFF2
lansoprazole-3529	1/100	0.561955	0.561955	0.637173	0.637173	1.462777	-0.84305	COL1A1
ioversol-6726	1/100	0.561955	0.561955	0.637173	0.637173	1.463382	-0.8434	DCN
ifenprodil-5044	1/100	0.561955	0.561955	0.637173	0.637173	1.464007	-0.84376	JUND
isoetarine-5812	1/100	0.561955	0.561955	0.637173	0.637173	1.464741	-0.84418	TFF1
iopamidol-2732	1/100	0.561955	0.561955	0.637173	0.637173	1.465879	-0.84484	IFI6
iodixanol-3023	1/100	0.561955	0.561955	0.637173	0.637173	1.466278	-0.84507	FXYD3
isoxsuprine-1985	1/100	0.561955	0.561955	0.637173	0.637173	1.466281	-0.84507	LY6E
imidurea-5481	1/100	0.561955	0.561955	0.637173	0.637173	1.46669	-0.8453	TSPAN1

imatinib-483	1/100	0.561955	0.561955	0.637173	0.637173	1.468144	-0.84614	CEACAM5
isoniazid-2246	1/100	0.561955	0.561955	0.637173	0.637173	1.468218	-0.84618	SPINK1
isoxsuprine-1904	1/100	0.561955	0.561955	0.637173	0.637173	1.469555	-0.84695	PIGR
karakoline-2203	1/100	0.561955	0.561955	0.637173	0.637173	1.473244	-0.84908	TFF2
isoconazole-5857	1/100	0.561955	0.561955	0.637173	0.637173	1.47651	-0.85096	COL1A2
iopamidol-3473	1/100	0.561955	0.561955	0.637173	0.637173	1.477208	-0.85136	CEACAM5
indoprofen-3007	1/100	0.561955	0.561955	0.637173	0.637173	1.479946	-0.85294	DCN
ivermectin-2051	1/100	0.561955	0.561955	0.637173	0.637173	1.480901	-0.85349	CCL20
labetalol-3167	1/100	0.561955	0.561955	0.637173	0.637173	1.482344	-0.85432	MMP3
ioxaglic acid-2966	1/100	0.561955	0.561955	0.637173	0.637173	1.483569	-0.85503	ATP1B1
indometacin-5049	1/100	0.561955	0.561955	0.637173	0.637173	1.484348	-0.85548	SLCO5A1
levcycloserine-4524	1/100	0.561955	0.561955	0.637173	0.637173	1.487357	-0.85721	COL1A1
ipratropium bromide-1769	1/100	0.561955	0.561955	0.637173	0.637173	1.488514	-0.85788	CTSE
iproniazid-6359	1/100	0.561955	0.561955	0.637173	0.637173	1.489761	-0.8586	TGFBI
isoniazid-1399	1/100	0.561955	0.561955	0.637173	0.637173	1.490646	-0.85911	ITM2B
letrozole-2916	1/100	0.561955	0.561955	0.637173	0.637173	1.490885	-0.85925	ACSL1
isoetarine-7170	1/100	0.561955	0.561955	0.637173	0.637173	1.492008	-0.85989	CEACAM5
ketanserin-3209	1/100	0.561955	0.561955	0.637173	0.637173	1.492109	-0.85995	CEACAM5
ketanserin-1593	1/100	0.561955	0.561955	0.637173	0.637173	1.492188	-0.86	PHLDA1
lidocaine-1499	1/100	0.561955	0.561955	0.637173	0.637173	1.493369	-0.86068	TMPRSS3
lasalocid-6639	1/100	0.561955	0.561955	0.637173	0.637173	1.493655	-0.86084	JUND
ionomycin-871	1/100	0.561955	0.561955	0.637173	0.637173	1.494474	-0.86132	ETS2
levomepromazine-3701	1/100	0.561955	0.561955	0.637173	0.637173	1.496161	-0.86229	CXCL1
isoxicam-1698	1/100	0.561955	0.561955	0.637173	0.637173	1.49641	-0.86243	RNF43
kawain-3670	1/100	0.561955	0.561955	0.637173	0.637173	1.49722	-0.8629	APP
leflunomide-6102	1/100	0.561955	0.561955	0.637173	0.637173	1.500227	-0.86463	C3
ivermectin-1367	1/100	0.561955	0.561955	0.637173	0.637173	1.50196	-0.86563	ITM2B
hyoscyamine-2271	1/100	0.561955	0.561955	0.637173	0.637173	1.502424	-0.8659	DCN
iproniazid-5458	1/100	0.561955	0.561955	0.637173	0.637173	1.502597	-0.866	PIGR
isradipine-5447	1/100	0.561955	0.561955	0.637173	0.637173	1.502643	-0.86602	SLPI
lansoprazole-2967	1/100	0.561955	0.561955	0.637173	0.637173	1.503169	-0.86633	TCN1
indoprofen-4832	1/100	0.561955	0.561955	0.637173	0.637173	1.505306	-0.86756	COL1A1
ketotifen-5842	1/100	0.561955	0.561955	0.637173	0.637173	1.506054	-0.86799	IFI6
isosorbide-4742	1/100	0.561955	0.561955	0.637173	0.637173	1.507665	-0.86892	COL1A1
levopropoxyphene-5083	1/100	0.561955	0.561955	0.637173	0.637173	1.508229	-0.86924	JUND
levodopa-4571	1/100	0.561955	0.561955	0.637173	0.637173	1.510918	-0.87079	S100A11
levothyroxine sodium-4069	1/100	0.561955	0.561955	0.637173	0.637173	1.511535	-0.87115	APP

kaempferol-7196	1/100	0.561955	0.561955	0.637173	0.637173	1.511947	-0.87139	SPINK1
ketorolac-6489	1/100	0.561955	0.561955	0.637173	0.637173	1.512405	-0.87165	TSPAN1
L-methionine sulfoximine-4151	1/100	0.561955	0.561955	0.637173	0.637173	1.512537	-0.87173	SECTM1
kinetin-6073	1/100	0.561955	0.561955	0.637173	0.637173	1.513208	-0.87211	PHLDA1
indometacin-2377	1/100	0.561955	0.561955	0.637173	0.637173	1.513703	-0.8724	GPX2
isoxsuprine-4205	1/100	0.561955	0.561955	0.637173	0.637173	1.515179	-0.87325	MMP3
lisinopril-5504	1/100	0.561955	0.561955	0.637173	0.637173	1.516778	-0.87417	CEACAM6
isradipine-6508	1/100	0.561955	0.561955	0.637173	0.637173	1.518988	-0.87544	TSPAN1
LM-1685-612	1/100	0.561955	0.561955	0.637173	0.637173	1.519258	-0.8756	RNF43
levamisole-1410	1/100	0.561955	0.561955	0.637173	0.637173	1.519367	-0.87566	PTMA
kaempferol-6157	1/100	0.561955	0.561955	0.637173	0.637173	1.519542	-0.87576	NQO1
ketoconazole-5685	1/100	0.561955	0.561955	0.637173	0.637173	1.520555	-0.87635	COL1A1
loperamide-1533	1/100	0.561955	0.561955	0.637173	0.637173	1.521506	-0.87689	COL1A1
isoconazole-1372	1/100	0.561955	0.561955	0.637173	0.637173	1.522045	-0.87721	PTMA
isoetarine-2711	1/100	0.561955	0.561955	0.637173	0.637173	1.522439	-0.87743	CCL20
laudanosine-7030	1/100	0.561955	0.561955	0.637173	0.637173	1.523405	-0.87799	GPX2
isocarboxazid-4706	1/100	0.561955	0.561955	0.637173	0.637173	1.523549	-0.87807	SERPINA1
ketoprofen-4286	1/100	0.561955	0.561955	0.637173	0.637173	1.524528	-0.87864	SPARC
liothyronine-4947	1/100	0.561955	0.561955	0.637173	0.637173	1.525348	-0.87911	COL1A1
LY-294002-1180	1/100	0.561955	0.561955	0.637173	0.637173	1.526364	-0.87969	CCL20
lidocaine-1999	1/100	0.561955	0.561955	0.637173	0.637173	1.526455	-0.87975	NPM1
lovastatin-2494	1/100	0.561955	0.561955	0.637173	0.637173	1.527144	-0.88014	COL1A1
lomustine-7045	1/100	0.561955	0.561955	0.637173	0.637173	1.529115	-0.88128	PHLDA1
laudanosine-2890	1/100	0.561955	0.561955	0.637173	0.637173	1.53	-0.88179	COL1A1
lidoflazine-3201	1/100	0.561955	0.561955	0.637173	0.637173	1.530275	-0.88195	PIGR
karakoline-6059	1/100	0.561955	0.561955	0.637173	0.637173	1.531101	-0.88243	PTPRO
liothyronine-3324	1/100	0.561955	0.561955	0.637173	0.637173	1.532253	-0.88309	COL1A2
isradipine-3129	1/100	0.561955	0.561955	0.637173	0.637173	1.533063	-0.88356	IFITM1
levcycloserine-3870	1/100	0.561955	0.561955	0.637173	0.637173	1.53413	-0.88417	AP1S1
loxapine-2016	1/100	0.561955	0.561955	0.637173	0.637173	1.534153	-0.88418	GPX2
lansoprazole-7306	1/100	0.561955	0.561955	0.637173	0.637173	1.53428	-0.88426	SERPINA1
LY-294002-1019	1/100	0.561955	0.561955	0.637173	0.637173	1.53575	-0.8851	TXNIP
lincomycin-5992	1/100	0.561955	0.561955	0.637173	0.637173	1.536543	-0.88556	CEACAM6
loperamide-5267	1/100	0.561955	0.561955	0.637173	0.637173	1.536724	-0.88567	FOS
ketorolac-3110	1/100	0.561955	0.561955	0.637173	0.637173	1.540252	-0.8877	NQO1
lithocholic acid-4373	1/100	0.561955	0.561955	0.637173	0.637173	1.540713	-0.88796	AP1S1
levonorgestrel-3708	1/100	0.561955	0.561955	0.637173	0.637173	1.541651	-0.88851	APP

LY-294002-1074	1/100	0.561955	0.561955	0.637173	0.637173	1.542484	-0.88899	TXNIP
LY-294002-1016	1/100	0.561955	0.561955	0.637173	0.637173	1.544032	-0.88988	TXNIP
levothyroxine sodium-3249	1/100	0.561955	0.561955	0.637173	0.637173	1.544332	-0.89005	COL1A1
LY-294002-1236	1/100	0.561955	0.561955	0.637173	0.637173	1.544589	-0.8902	MMP1
LY-294002-461	1/100	0.561955	0.561955	0.637173	0.637173	1.544681	-0.89025	PIGR
LY-294002-1168	1/100	0.561955	0.561955	0.637173	0.637173	1.544881	-0.89037	JUND
LY-294002-5213	1/100	0.561955	0.561955	0.637173	0.637173	1.546919	-0.89154	COL1A1
levopropoxyphene-5503	1/100	0.561955	0.561955	0.637173	0.637173	1.547014	-0.8916	MMP3
ketoprofen-2354	1/100	0.561955	0.561955	0.637173	0.637173	1.54717	-0.89169	FXYD3
levocabastine-7009	1/100	0.561955	0.561955	0.637173	0.637173	1.547501	-0.89188	ITM2C
LY-294002-6998	1/100	0.561955	0.561955	0.637173	0.637173	1.549405	-0.89297	TXNIP
levonorgestrel-3606	1/100	0.561955	0.561955	0.637173	0.637173	1.558614	-0.89828	COL1A1
lycorine-3891	1/100	0.561955	0.561955	0.637173	0.637173	1.563518	-0.90111	IFI6
lysergol-3261	1/100	0.561955	0.561955	0.637173	0.637173	1.565912	-0.90249	CEACAM5
luteolin-5004	1/100	0.561955	0.561955	0.637173	0.637173	1.566136	-0.90262	SOX9
LY-294002-501	1/100	0.561955	0.561955	0.637173	0.637173	1.567027	-0.90313	APP
LY-294002-1157	1/100	0.561955	0.561955	0.637173	0.637173	1.568707	-0.9041	MMP3
levopropoxyphene-3543	1/100	0.561955	0.561955	0.637173	0.637173	1.569913	-0.90479	S100A11
letrozole-4824	1/100	0.561955	0.561955	0.637173	0.637173	1.570496	-0.90513	IFI6
LY-294002-6987	1/100	0.561955	0.561955	0.637173	0.637173	1.571014	-0.90543	TXNIP
mebeverine-1576	1/100	0.561955	0.561955	0.637173	0.637173	1.571277	-0.90558	PTMA
meclofenamic acid-7038	1/100	0.561955	0.561955	0.637173	0.637173	1.571388	-0.90564	SOX9
lumicolchicine-4195	1/100	0.561955	0.561955	0.637173	0.637173	1.572282	-0.90616	TSPAN8
mepenzolate bromide-6024	1/100	0.561955	0.561955	0.637173	0.637173	1.572783	-0.90645	TCN1
lisinopril-2371	1/100	0.561955	0.561955	0.637173	0.637173	1.573913	-0.9071	IFI6
mefloquine-2048	1/100	0.561955	0.561955	0.637173	0.637173	1.576526	-0.9086	MMP1
LY-294002-5942	1/100	0.561955	0.561955	0.637173	0.637173	1.577025	-0.90889	DCN
LY-294002-1664	1/100	0.561955	0.561955	0.637173	0.637173	1.577139	-0.90896	TXNIP
LY-294002-1652	1/100	0.561955	0.561955	0.637173	0.637173	1.577201	-0.90899	TXNIP
lasalocid-3360	1/100	0.561955	0.561955	0.637173	0.637173	1.577364	-0.90909	CTGF
LY-294002-1007	1/100	0.561955	0.561955	0.637173	0.637173	1.57898	-0.91002	TXNIP
lomefloxacin-2348	1/100	0.561955	0.561955	0.637173	0.637173	1.580514	-0.9109	LY6E
LY-294002-2676	1/100	0.561955	0.561955	0.637173	0.637173	1.581797	-0.91164	IFI6
meclofenoxate-3605	1/100	0.561955	0.561955	0.637173	0.637173	1.582153	-0.91185	DCN
mebhydrolin-3269	1/100	0.561955	0.561955	0.637173	0.637173	1.582405	-0.91199	CEACAM5
lymecycline-3514	1/100	0.561955	0.561955	0.637173	0.637173	1.582683	-0.91215	ITM2C
mefexamide-2284	1/100	0.561955	0.561955	0.637173	0.637173	1.584283	-0.91308	RCN1

LY-294002-5970	1/100	0.561955	0.561955	0.637173	0.637173	1.585466	-0.91376	CTGF
LY-294002-6175	1/100	0.561955	0.561955	0.637173	0.637173	1.585989	-0.91406	CEACAM5
mafenide-5499	1/100	0.561955	0.561955	0.637173	0.637173	1.586497	-0.91435	COL1A1
LY-294002-5576	1/100	0.561955	0.561955	0.637173	0.637173	1.587614	-0.915	PIGR
luteolin-3041	1/100	0.561955	0.561955	0.637173	0.637173	1.588519	-0.91552	CCL20
lynestrenol-6756	1/100	0.561955	0.561955	0.637173	0.637173	1.590357	-0.91658	FOS
megestrol-3091	1/100	0.561955	0.561955	0.637173	0.637173	1.594128	-0.91875	IFITM1
LY-294002-5596	1/100	0.561955	0.561955	0.637173	0.637173	1.595984	-0.91982	TXNIP
LY-294002-424	1/100	0.561955	0.561955	0.637173	0.637173	1.596073	-0.91987	AP1S1
mafenide-2124	1/100	0.561955	0.561955	0.637173	0.637173	1.597384	-0.92063	IFI6
mepyramine-3184	1/100	0.561955	0.561955	0.637173	0.637173	1.598547	-0.9213	PHLDA1
lysergol-4966	1/100	0.561955	0.561955	0.637173	0.637173	1.600376	-0.92235	SPINK1
lobeline-2763	1/100	0.561955	0.561955	0.637173	0.637173	1.600398	-0.92236	COL1A1
lymecycline-2953	1/100	0.561955	0.561955	0.637173	0.637173	1.601613	-0.92306	ATP1B1
LY-294002-6953	1/100	0.561955	0.561955	0.637173	0.637173	1.605057	-0.92505	TXNIP
lomefloxacin-2310	1/100	0.561955	0.561955	0.637173	0.637173	1.605922	-0.92555	PDZK1IP1
meclofenoxate-3707	1/100	0.561955	0.561955	0.637173	0.637173	1.607158	-0.92626	DCN
maprotiline-3236	1/100	0.561955	0.561955	0.637173	0.637173	1.607746	-0.9266	SOX9
mebhydrolin-1333	1/100	0.561955	0.561955	0.637173	0.637173	1.607791	-0.92662	S100A11
meprylcaine-3544	1/100	0.561955	0.561955	0.637173	0.637173	1.608136	-0.92682	COL1A2
meclofenamic acid-2291	1/100	0.561955	0.561955	0.637173	0.637173	1.608233	-0.92688	PROM1
meclocycline-1341	1/100	0.561955	0.561955	0.637173	0.637173	1.608382	-0.92696	JUND
lobelanidine-2897	1/100	0.561955	0.561955	0.637173	0.637173	1.608741	-0.92717	IFI6
meclofenoxate-4729	1/100	0.561955	0.561955	0.637173	0.637173	1.609177	-0.92742	COL1A1
meclofenamic acid-2128	1/100	0.561955	0.561955	0.637173	0.637173	1.610171	-0.928	BMP4
meclozine-3285	1/100	0.561955	0.561955	0.637173	0.637173	1.611463	-0.92874	PIGR
mephentermine-3425	1/100	0.561955	0.561955	0.637173	0.637173	1.611597	-0.92882	TSPAN1
meclocycline-6637	1/100	0.561955	0.561955	0.637173	0.637173	1.612064	-0.92909	JUND
meclofenoxate-4268	1/100	0.561955	0.561955	0.637173	0.637173	1.613375	-0.92984	COL1A1
LY-294002-5599	1/100	0.561955	0.561955	0.637173	0.637173	1.615966	-0.93134	TXNIP
mepenzolate bromide-3829	1/100	0.561955	0.561955	0.637173	0.637173	1.616682	-0.93175	COL1A1
mercaptopurine-334	1/100	0.561955	0.561955	0.637173	0.637173	1.619346	-0.93328	IFITM1
LY-294002-1077	1/100	0.561955	0.561955	0.637173	0.637173	1.620217	-0.93379	TXNIP
merbromin-4722	1/100	0.561955	0.561955	0.637173	0.637173	1.621985	-0.9348	ITM2C
LY-294002-1661	1/100	0.561955	0.561955	0.637173	0.637173	1.622658	-0.93519	TXNIP
LY-294002-2696	1/100	0.561955	0.561955	0.637173	0.637173	1.625976	-0.9371	ATP1B1
memantine-4017	1/100	0.561955	0.561955	0.637173	0.637173	1.630392	-0.93965	AP1S1

methotrexate-3214	1/100	0.561955	0.561955	0.637173	0.637173	1.630583	-0.93976	DCN
menadione-4662	1/100	0.561955	0.561955	0.637173	0.637173	1.630711	-0.93983	FOS
lynestrenol-1953	1/100	0.561955	0.561955	0.637173	0.637173	1.632151	-0.94066	CD24
mephenytoin-5801	1/100	0.561955	0.561955	0.637173	0.637173	1.636515	-0.94318	DUOX2
mepacrine-3179	1/100	0.561955	0.561955	0.637173	0.637173	1.637347	-0.94366	SOX9
mefenamic acid-1863	1/100	0.561955	0.561955	0.637173	0.637173	1.638093	-0.94409	S100A11
metacycline-7321	1/100	0.561955	0.561955	0.637173	0.637173	1.638438	-0.94429	ITM2C
metanephrine-5334	1/100	0.561955	0.561955	0.637173	0.637173	1.638478	-0.94431	SERPINA1
mepacrine-1563	1/100	0.561955	0.561955	0.637173	0.637173	1.638677	-0.94442	QPCT
metaraminol-3669	1/100	0.561955	0.561955	0.637173	0.637173	1.639099	-0.94467	RNF43
mestranol-3346	1/100	0.561955	0.561955	0.637173	0.637173	1.63945	-0.94487	PHLDA1
mephentermine-7384	1/100	0.561955	0.561955	0.637173	0.637173	1.63998	-0.94518	IFITM1
medrysone-4727	1/100	0.561955	0.561955	0.637173	0.637173	1.641951	-0.94631	COL1A1
mephenesin-2304	1/100	0.561955	0.561955	0.637173	0.637173	1.642149	-0.94643	PIGR
mepyramine-7223	1/100	0.561955	0.561955	0.637173	0.637173	1.642684	-0.94673	SERPINA1
metanephrine-6734	1/100	0.561955	0.561955	0.637173	0.637173	1.642892	-0.94685	C3
metaraminol-2298	1/100	0.561955	0.561955	0.637173	0.637173	1.643162	-0.94701	COL1A2
metolazone-6292	1/100	0.561955	0.561955	0.637173	0.637173	1.644091	-0.94754	CEACAM6
merbromin-3700	1/100	0.561955	0.561955	0.637173	0.637173	1.644359	-0.9477	JUND
methylbenzethonium chloride-3768	1/100	0.561955	0.561955	0.637173	0.637173	1.644406	-0.94773	IL32
mephenesin-7374	1/100	0.561955	0.561955	0.637173	0.637173	1.644728	-0.94791	TCN1
mephentermine-3685	1/100	0.561955	0.561955	0.637173	0.637173	1.645225	-0.9482	PTPRO
mepenzolate bromide-2169	1/100	0.561955	0.561955	0.637173	0.637173	1.646053	-0.94868	ID3
methylbenzethonium chloride-6045	1/100	0.561955	0.561955	0.637173	0.637173	1.64769	-0.94962	PHLDA1
meropenem-7180	1/100	0.561955	0.561955	0.637173	0.637173	1.647816	-0.94969	TXNIP
metronidazole-2003	1/100	0.561955	0.561955	0.637173	0.637173	1.648098	-0.94985	PIGR
meprylcaine-5723	1/100	0.561955	0.561955	0.637173	0.637173	1.648211	-0.94992	MMP3
meglumine-3068	1/100	0.561955	0.561955	0.637173	0.637173	1.65054	-0.95126	IFITM1
mesalazine-5888	1/100	0.561955	0.561955	0.637173	0.637173	1.651005	-0.95153	JUND
merbromin-7398	1/100	0.561955	0.561955	0.637173	0.637173	1.65311	-0.95274	S100A11
metformin-5487	1/100	0.561955	0.561955	0.637173	0.637173	1.653549	-0.953	PTPRO
mepenzolate bromide-3748	1/100	0.561955	0.561955	0.637173	0.637173	1.658848	-0.95605	DUOX2
metacycline-4143	1/100	0.561955	0.561955	0.637173	0.637173	1.659357	-0.95634	CEACAM5
memantine-4135	1/100	0.561955	0.561955	0.637173	0.637173	1.659754	-0.95657	COL1A1
metergoline-5344	1/100	0.561955	0.561955	0.637173	0.637173	1.660156	-0.9568	RNF43
methylprednisolone-3183	1/100	0.561955	0.561955	0.637173	0.637173	1.664078	-0.95906	APP

metamizole sodium-6030	1/100	0.561955	0.561955	0.637173	0.637173	1.665128	-0.95967	CD14
metoclopramide-3728	1/100	0.561955	0.561955	0.637173	0.637173	1.665739	-0.96002	IL32
metaraminol-4692	1/100	0.561955	0.561955	0.637173	0.637173	1.666866	-0.96067	TFF2
metrizamide-3255	1/100	0.561955	0.561955	0.637173	0.637173	1.668659	-0.9617	AP1S1
methyldopate-7360	1/100	0.561955	0.561955	0.637173	0.637173	1.669714	-0.96231	RCN1
metergoline-1606	1/100	0.561955	0.561955	0.637173	0.637173	1.670569	-0.9628	CCL20
methacholine chloride-5773	1/100	0.561955	0.561955	0.637173	0.637173	1.670654	-0.96285	DUOX2
metampicillin-5540	1/100	0.561955	0.561955	0.637173	0.637173	1.672291	-0.9638	IFI6
mestranol-4792	1/100	0.561955	0.561955	0.637173	0.637173	1.67239	-0.96385	CEACAM5
meptazinol-4774	1/100	0.561955	0.561955	0.637173	0.637173	1.674938	-0.96532	COL1A1
methyldopate-6640	1/100	0.561955	0.561955	0.637173	0.637173	1.674946	-0.96533	TGFBI
metrifonate-5989	1/100	0.561955	0.561955	0.637173	0.637173	1.675184	-0.96546	MMP3
metformin-1694	1/100	0.561955	0.561955	0.637173	0.637173	1.67582	-0.96583	JUND
midodrine-6804	1/100	0.561955	0.561955	0.637173	0.637173	1.681884	-0.96933	MMP3
metronidazole-1503	1/100	0.561955	0.561955	0.637173	0.637173	1.683847	-0.97046	APP
meropenem-3564	1/100	0.561955	0.561955	0.637173	0.637173	1.683932	-0.97051	C3
monensin-1105	1/100	0.561955	0.561955	0.637173	0.637173	1.684246	-0.97069	PHLDA1
minaprine-1468	1/100	0.561955	0.561955	0.637173	0.637173	1.688046	-0.97288	COL1A1
molindone-2917	1/100	0.561955	0.561955	0.637173	0.637173	1.688317	-0.97303	IGFBP2
methylergometrine-6704	1/100	0.561955	0.561955	0.637173	0.637173	1.689566	-0.97375	COL1A2
methotrexate-5000	1/100	0.561955	0.561955	0.637173	0.637173	1.689971	-0.97399	MMP3
melatonin-6293	1/100	0.561955	0.561955	0.637173	0.637173	1.690858	-0.9745	TCN1
methyldopa-4677	1/100	0.561955	0.561955	0.637173	0.637173	1.691404	-0.97481	CEACAM5
methoxamine-2848	1/100	0.561955	0.561955	0.637173	0.637173	1.692944	-0.9757	RNF43
miconazole-1977	1/100	0.561955	0.561955	0.637173	0.637173	1.694017	-0.97632	CEACAM5
methotrexate-1957	1/100	0.561955	0.561955	0.637173	0.637173	1.694746	-0.97674	ETS2
mexiletine-2324	1/100	0.561955	0.561955	0.637173	0.637173	1.697513	-0.97833	DCN
methoxamine-4972	1/100	0.561955	0.561955	0.637173	0.637173	1.69855	-0.97893	BMP4
meteneprost-7500	1/100	0.561955	0.561955	0.637173	0.637173	1.698991	-0.97919	COL1A1
moracizine-7297	1/100	0.561955	0.561955	0.637173	0.637173	1.701167	-0.98044	SPP1
mifepristone-3185	1/100	0.561955	0.561955	0.637173	0.637173	1.701548	-0.98066	TSPAN8
methyldopate-2940	1/100	0.561955	0.561955	0.637173	0.637173	1.701714	-0.98075	PHLDA1
metoclopramide-4285	1/100	0.561955	0.561955	0.637173	0.637173	1.702926	-0.98145	AP1S1
metitepine-1616	1/100	0.561955	0.561955	0.637173	0.637173	1.703122	-0.98157	CCL20
methoxsalen-6661	1/100	0.561955	0.561955	0.637173	0.637173	1.703585	-0.98183	OLFM4
monorden-484	1/100	0.561955	0.561955	0.637173	0.637173	1.703821	-0.98197	HSPB1
metolazone-5392	1/100	0.561955	0.561955	0.637173	0.637173	1.704509	-0.98237	SECTM1

MG-132-1140	1/100	0.561955	0.561955	0.637173	0.637173	1.704875	-0.98258	S100P
meticrane-1834	1/100	0.561955	0.561955	0.637173	0.637173	1.709866	-0.98545	DEK
minocycline-7436	1/100	0.561955	0.561955	0.637173	0.637173	1.715152	-0.9885	COL1A1
minocycline-5077	1/100	0.561955	0.561955	0.637173	0.637173	1.71542	-0.98865	BMP4
mexiletine-3781	1/100	0.561955	0.561955	0.637173	0.637173	1.717907	-0.99009	COL1A1
metampicillin-2286	1/100	0.561955	0.561955	0.637173	0.637173	1.718474	-0.99041	RCN1
miconazole-1896	1/100	0.561955	0.561955	0.637173	0.637173	1.718586	-0.99048	CD24
miconazole-4960	1/100	0.561955	0.561955	0.637173	0.637173	1.720594	-0.99164	CEACAM5
metyrapone-4606	1/100	0.561955	0.561955	0.637173	0.637173	1.721093	-0.99192	SPARC
moroxydine-1944	1/100	0.561955	0.561955	0.637173	0.637173	1.72146	-0.99214	CEACAM5
midecamycin-1526	1/100	0.561955	0.561955	0.637173	0.637173	1.722084	-0.99249	SOX9
metyrapone-3070	1/100	0.561955	0.561955	0.637173	0.637173	1.722539	-0.99276	SPARC
moxisylyte-1804	1/100	0.561955	0.561955	0.637173	0.637173	1.723206	-0.99314	APP
mycophenolic acid-4019	1/100	0.561955	0.561955	0.637173	0.637173	1.724074	-0.99364	CEACAM6
mianserin-2231	1/100	0.561955	0.561955	0.637173	0.637173	1.72429	-0.99377	ITM2C
mexiletine-4338	1/100	0.561955	0.561955	0.637173	0.637173	1.725305	-0.99435	SPP1
minaprine-1968	1/100	0.561955	0.561955	0.637173	0.637173	1.726661	-0.99513	AP1S1
metyrapone-6447	1/100	0.561955	0.561955	0.637173	0.637173	1.727118	-0.9954	PIGR
mexiletine-3862	1/100	0.561955	0.561955	0.637173	0.637173	1.729859	-0.99698	COL1A1
mometasone-5116	1/100	0.561955	0.561955	0.637173	0.637173	1.730563	-0.99738	CCL20
N-acetyl-L-leucine-3085	1/100	0.561955	0.561955	0.637173	0.637173	1.732186	-0.99832	CEACAM5
midecamycin-2026	1/100	0.561955	0.561955	0.637173	0.637173	1.732579	-0.99854	GPX2
moxisylyte-1846	1/100	0.561955	0.561955	0.637173	0.637173	1.732722	-0.99863	TCN1
metolazone-2014	1/100	0.561955	0.561955	0.637173	0.637173	1.734365	-0.99957	PLCB4
morantel-7010	1/100	0.561955	0.561955	0.637173	0.637173	1.73536	-1.00015	MMP3
minoxidil-1496	1/100	0.561955	0.561955	0.637173	0.637173	1.738579	-1.002	SPP1
naftidrofuryl-6687	1/100	0.561955	0.561955	0.637173	0.637173	1.738684	-1.00206	CTSE
meteneprost-7552	1/100	0.561955	0.561955	0.637173	0.637173	1.739087	-1.00229	S100A4
milrinone-3552	1/100	0.561955	0.561955	0.637173	0.637173	1.739327	-1.00243	COL1A1
monocrotaline-2749	1/100	0.561955	0.561955	0.637173	0.637173	1.740081	-1.00287	AP1S1
moxisylyte-7015	1/100	0.561955	0.561955	0.637173	0.637173	1.742003	-1.00397	GPX2
N-acetyl-L-aspartic acid-3265	1/100	0.561955	0.561955	0.637173	0.637173	1.742491	-1.00426	S100A6
monensin-3704	1/100	0.561955	0.561955	0.637173	0.637173	1.745382	-1.00592	C3
monorden-5579	1/100	0.561955	0.561955	0.637173	0.637173	1.747939	-1.0074	SOX9
nafcillin-3323	1/100	0.561955	0.561955	0.637173	0.637173	1.751612	-1.00951	PIGR
monobenzone-3054	1/100	0.561955	0.561955	0.637173	0.637173	1.752655	-1.01011	PHLDA1
nabumetone-6327	1/100	0.561955	0.561955	0.637173	0.637173	1.753189	-1.01042	AP1S1

monobenzone-6713	1/100	0.561955	0.561955	0.637173	0.637173	1.754676	-1.01128	DUOX2
molsidomine-1711	1/100	0.561955	0.561955	0.637173	0.637173	1.757031	-1.01264	APP
monorden-5952	1/100	0.561955	0.561955	0.637173	0.637173	1.757044	-1.01264	HSPB1
naphazoline-6604	1/100	0.561955	0.561955	0.637173	0.637173	1.757135	-1.0127	MMP12
mitoxantrone-6755	1/100	0.561955	0.561955	0.637173	0.637173	1.759346	-1.01397	C3
naftifine-7032	1/100	0.561955	0.561955	0.637173	0.637173	1.76112	-1.01499	ETS2
monastrol-681	1/100	0.561955	0.561955	0.637173	0.637173	1.761493	-1.01521	S100P
naftidrofuryl-2622	1/100	0.561955	0.561955	0.637173	0.637173	1.765523	-1.01753	SPINK1
MK-886-264	1/100	0.561955	0.561955	0.637173	0.637173	1.765716	-1.01764	ID3
napelline-6084	1/100	0.561955	0.561955	0.637173	0.637173	1.769296	-1.0197	AP1S1
naftifine-3536	1/100	0.561955	0.561955	0.637173	0.637173	1.771984	-1.02125	AP1S1
morantel-1798	1/100	0.561955	0.561955	0.637173	0.637173	1.772467	-1.02153	AP1S1
nalidixic acid-2297	1/100	0.561955	0.561955	0.637173	0.637173	1.772821	-1.02174	SPP1
mometasone-5541	1/100	0.561955	0.561955	0.637173	0.637173	1.773295	-1.02201	APP
myricetin-4090	1/100	0.561955	0.561955	0.637173	0.637173	1.773898	-1.02236	FOS
mimosine-2638	1/100	0.561955	0.561955	0.637173	0.637173	1.774454	-1.02268	CEACAM5
moracizine-3520	1/100	0.561955	0.561955	0.637173	0.637173	1.774675	-1.0228	PHLDA1
mycophenolic acid-2857	1/100	0.561955	0.561955	0.637173	0.637173	1.77705	-1.02417	TXNIP
minaprine-1888	1/100	0.561955	0.561955	0.637173	0.637173	1.777143	-1.02423	TPI1
monocrotaline-1757	1/100	0.561955	0.561955	0.637173	0.637173	1.778462	-1.02499	IFITM3
naproxen-6794	1/100	0.561955	0.561955	0.637173	0.637173	1.779775	-1.02574	MMP3
naftidrofuryl-1267	1/100	0.561955	0.561955	0.637173	0.637173	1.781331	-1.02664	AP1S1
nafcillin-2983	1/100	0.561955	0.561955	0.637173	0.637173	1.782302	-1.0272	DCN
N6-methyladenosine-1271	1/100	0.561955	0.561955	0.637173	0.637173	1.783431	-1.02785	IFI6
monorden-449	1/100	0.561955	0.561955	0.637173	0.637173	1.7835	-1.02789	HSPB1
N6-methyladenosine-6732	1/100	0.561955	0.561955	0.637173	0.637173	1.786414	-1.02957	S100P
nafcillin-4103	1/100	0.561955	0.561955	0.637173	0.637173	1.787739	-1.03033	DCN
niclosamide-4018	1/100	0.561955	0.561955	0.637173	0.637173	1.790947	-1.03218	S100P
moracizine-2959	1/100	0.561955	0.561955	0.637173	0.637173	1.791367	-1.03242	ID1
moxisylyte-7255	1/100	0.561955	0.561955	0.637173	0.637173	1.791443	-1.03247	CTGF
nadide-5873	1/100	0.561955	0.561955	0.637173	0.637173	1.791979	-1.03278	CEACAM6
neomycin-2229	1/100	0.561955	0.561955	0.637173	0.637173	1.792431	-1.03304	SECTM1
naftopidil-7331	1/100	0.561955	0.561955	0.637173	0.637173	1.793212	-1.03349	CTSE
neomycin-2066	1/100	0.561955	0.561955	0.637173	0.637173	1.794113	-1.03401	TCN1
nordihydroguaiaretic acid-1061	1/100	0.561955	0.561955	0.637173	0.637173	1.795596	-1.03486	CD24
nilutamide-3104	1/100	0.561955	0.561955	0.637173	0.637173	1.798875	-1.03675	CEACAM6
napelline-4486	1/100	0.561955	0.561955	0.637173	0.637173	1.799818	-1.0373	DCN

moroxydine-5304	1/100	0.561955	0.561955	0.637173	0.637173	1.799953	-1.03737	IFI6
naltrexone-6241	1/100	0.561955	0.561955	0.637173	0.637173	1.802091	-1.03861	BMP4
nalbuphine-2063	1/100	0.561955	0.561955	0.637173	0.637173	1.8039	-1.03965	CCL20
naloxone-5606	1/100	0.561955	0.561955	0.637173	0.637173	1.804387	-1.03993	ITM2C
oligomycin-442	1/100	0.561955	0.561955	0.637173	0.637173	1.804445	-1.03996	S100A4
N-acetyl-L-leucine-5683	1/100	0.561955	0.561955	0.637173	0.637173	1.805332	-1.04047	COL1A2
monorden-5216	1/100	0.561955	0.561955	0.637173	0.637173	1.805537	-1.04059	IFI6
nitrendipine-336	1/100	0.561955	0.561955	0.637173	0.637173	1.809699	-1.04299	GPX2
neomycin-7221	1/100	0.561955	0.561955	0.637173	0.637173	1.811421	-1.04398	TFF2
N-phenylanthranilic acid-317	1/100	0.561955	0.561955	0.637173	0.637173	1.811987	-1.04431	IFITM1
nordihydroguaiaretic acid-1223	1/100	0.561955	0.561955	0.637173	0.637173	1.813007	-1.0449	S100A11
nicergoline-1374	1/100	0.561955	0.561955	0.637173	0.637173	1.813765	-1.04533	ENC1
novobiocin-4569	1/100	0.561955	0.561955	0.637173	0.637173	1.814205	-1.04559	PSMB9
norcyclobenzaprine-4776	1/100	0.561955	0.561955	0.637173	0.637173	1.814741	-1.0459	PHLDA1
ondansetron-3575	1/100	0.561955	0.561955	0.637173	0.637173	1.815962	-1.0466	COL1A1
naringenin-3278	1/100	0.561955	0.561955	0.637173	0.637173	1.816705	-1.04703	CTSD
naringin-2425	1/100	0.561955	0.561955	0.637173	0.637173	1.817401	-1.04743	DCN
nisoxetine-6496	1/100	0.561955	0.561955	0.637173	0.637173	1.818561	-1.0481	CD14
nicardipine-6297	1/100	0.561955	0.561955	0.637173	0.637173	1.818671	-1.04816	CEACAM6
neostigmine bromide-6735	1/100	0.561955	0.561955	0.637173	0.637173	1.819232	-1.04848	IL32
nialamide-4347	1/100	0.561955	0.561955	0.637173	0.637173	1.819456	-1.04861	PTPRO
nalidixic acid-2336	1/100	0.561955	0.561955	0.637173	0.637173	1.819862	-1.04885	AP1S1
nizatidine-6305	1/100	0.561955	0.561955	0.637173	0.637173	1.819981	-1.04892	AP1S1
nefopam-2317	1/100	0.561955	0.561955	0.637173	0.637173	1.82088	-1.04943	SPARC
naproxen-1869	1/100	0.561955	0.561955	0.637173	0.637173	1.821013	-1.04951	NQO1
nimesulide-1428	1/100	0.561955	0.561955	0.637173	0.637173	1.821232	-1.04964	NQO1
NS-398-6892	1/100	0.561955	0.561955	0.637173	0.637173	1.822982	-1.05065	IFI6
nifenazone-1439	1/100	0.561955	0.561955	0.637173	0.637173	1.823694	-1.05106	PIGR
myosmine-6055	1/100	0.561955	0.561955	0.637173	0.637173	1.823895	-1.05117	ITM2C
neostigmine bromide-3294	1/100	0.561955	0.561955	0.637173	0.637173	1.825126	-1.05188	AP1S1
nicotinic acid-6702	1/100	0.561955	0.561955	0.637173	0.637173	1.825166	-1.0519	JUND
noscapine-2745	1/100	0.561955	0.561955	0.637173	0.637173	1.82517	-1.05191	APP
norcyclobenzaprine-2830	1/100	0.561955	0.561955	0.637173	0.637173	1.826855	-1.05288	SPP1
nitrofurantoin-2303	1/100	0.561955	0.561955	0.637173	0.637173	1.828496	-1.05382	COL1A1
novobiocin-437	1/100	0.561955	0.561955	0.637173	0.637173	1.828819	-1.05401	CKB
NU-1025-313	1/100	0.561955	0.561955	0.637173	0.637173	1.829654	-1.05449	CEACAM5
naloxone-1506	1/100	0.561955	0.561955	0.637173	0.637173	1.829907	-1.05464	ENC1

natamycin-3548	1/100	0.561955	0.561955	0.637173	0.637173	1.830688	-1.05509	CEACAM5
nitrofurantoin-3674	1/100	0.561955	0.561955	0.637173	0.637173	1.831219	-1.05539	PSMB9
nordihydroguaiaretic acid-5220	1/100	0.561955	0.561955	0.637173	0.637173	1.833041	-1.05644	JUND
nimodipine-3103	1/100	0.561955	0.561955	0.637173	0.637173	1.833561	-1.05674	IGFBP2
nitrofural-3320	1/100	0.561955	0.561955	0.637173	0.637173	1.836345	-1.05835	S100A11
nordihydroguaiaretic acid-1164	1/100	0.561955	0.561955	0.637173	0.637173	1.838001	-1.0593	JUND
oleandomycin-2018	1/100	0.561955	0.561955	0.637173	0.637173	1.83858	-1.05964	IFITM3
nitrendipine-5405	1/100	0.561955	0.561955	0.637173	0.637173	1.839478	-1.06015	S100A11
nialamide-4525	1/100	0.561955	0.561955	0.637173	0.637173	1.842976	-1.06217	COL1A2
nimodipine-6480	1/100	0.561955	0.561955	0.637173	0.637173	1.843953	-1.06273	DUOX2
naringin-5666	1/100	0.561955	0.561955	0.637173	0.637173	1.845583	-1.06367	TFF2
nefopam-3627	1/100	0.561955	0.561955	0.637173	0.637173	1.847125	-1.06456	TCN1
nomifensine-1378	1/100	0.561955	0.561955	0.637173	0.637173	1.850528	-1.06652	ENC1
nordihydroguaiaretic acid-203	1/100	0.561955	0.561955	0.637173	0.637173	1.853335	-1.06814	DPEP1
noscapine-1753	1/100	0.561955	0.561955	0.637173	0.637173	1.853819	-1.06842	IFITM3
octopamine-5469	1/100	0.561955	0.561955	0.637173	0.637173	1.854016	-1.06853	MMP3
oxolinic acid-1419	1/100	0.561955	0.561955	0.637173	0.637173	1.855176	-1.0692	AP1S1
orciprenaline-2845	1/100	0.561955	0.561955	0.637173	0.637173	1.856185	-1.06978	SLPI
nisoxetine-5091	1/100	0.561955	0.561955	0.637173	0.637173	1.856242	-1.06981	MMP3
novobiocin-499	1/100	0.561955	0.561955	0.637173	0.637173	1.85694	-1.07022	DPEP1
nifedipine-603	1/100	0.561955	0.561955	0.637173	0.637173	1.858697	-1.07123	SLPI
oxyphenbutazone-6160	1/100	0.561955	0.561955	0.637173	0.637173	1.858709	-1.07124	NQO1
norfloxacin-7283	1/100	0.561955	0.561955	0.637173	0.637173	1.859634	-1.07177	JUND
nifurtimox-2908	1/100	0.561955	0.561955	0.637173	0.637173	1.861103	-1.07262	SLCO5A1
nifenazone-2285	1/100	0.561955	0.561955	0.637173	0.637173	1.861383	-1.07278	LYZ
nocodazole-2076	1/100	0.561955	0.561955	0.637173	0.637173	1.861971	-1.07312	RPL28
nipecotic acid-6500	1/100	0.561955	0.561955	0.637173	0.637173	1.865599	-1.07521	COL1A2
nomegestrol-6525	1/100	0.561955	0.561955	0.637173	0.637173	1.865931	-1.0754	COL1A1
oxetacaine-1984	1/100	0.561955	0.561955	0.637173	0.637173	1.866013	-1.07545	CEACAM5
octopamine-3112	1/100	0.561955	0.561955	0.637173	0.637173	1.866619	-1.0758	CTSE
oxybutynin-1551	1/100	0.561955	0.561955	0.637173	0.637173	1.868181	-1.0767	LGALS3BP
nitrendipine-6464	1/100	0.561955	0.561955	0.637173	0.637173	1.872613	-1.07925	COL1A1
palmatine-2795	1/100	0.561955	0.561955	0.637173	0.637173	1.872851	-1.07939	MMP3
perphenazine-4637	1/100	0.561955	0.561955	0.637173	0.637173	1.87332	-1.07966	JUND
norethisterone-5474	1/100	0.561955	0.561955	0.637173	0.637173	1.873524	-1.07977	SLPI
nialamide-3871	1/100	0.561955	0.561955	0.637173	0.637173	1.878451	-1.08261	CEACAM5
nordihydroguaiaretic acid-524	1/100	0.561955	0.561955	0.637173	0.637173	1.87859	-1.08269	PSMB9

octopamine-5050	1/100	0.561955	0.561955	0.637173	0.637173	1.879304	-1.08311	PSMB9
oxybutynin-3168	1/100	0.561955	0.561955	0.637173	0.637173	1.879505	-1.08322	MMP3
ofloxacin-3673	1/100	0.561955	0.561955	0.637173	0.637173	1.88009	-1.08356	PDZK1IP1
oxymetazoline-1431	1/100	0.561955	0.561955	0.637173	0.637173	1.8801	-1.08356	PTMA
novobiocin-4392	1/100	0.561955	0.561955	0.637173	0.637173	1.880129	-1.08358	PDZK1IP1
oleandomycin-4615	1/100	0.561955	0.561955	0.637173	0.637173	1.880212	-1.08363	CEACAM5
omeprazole-6606	1/100	0.561955	0.561955	0.637173	0.637173	1.88203	-1.08468	CTSE
oxybenzone-3092	1/100	0.561955	0.561955	0.637173	0.637173	1.882882	-1.08517	IFITM1
orlistat-6388	1/100	0.561955	0.561955	0.637173	0.637173	1.883088	-1.08529	AP1S1
oxprenolol-5871	1/100	0.561955	0.561955	0.637173	0.637173	1.883927	-1.08577	AP1S1
pararosaniline-893	1/100	0.561955	0.561955	0.637173	0.637173	1.884739	-1.08624	ID1
oxolamine-6624	1/100	0.561955	0.561955	0.637173	0.637173	1.885086	-1.08644	TSPAN1
ornidazole-1425	1/100	0.561955	0.561955	0.637173	0.637173	1.885946	-1.08693	DPEP1
noretynodrel-1860	1/100	0.561955	0.561955	0.637173	0.637173	1.889398	-1.08892	ID3
parthenolide-5530	1/100	0.561955	0.561955	0.637173	0.637173	1.890381	-1.08949	ID1
pepstatin-4206	1/100	0.561955	0.561955	0.637173	0.637173	1.891624	-1.09021	IFITM1
ondansetron-5796	1/100	0.561955	0.561955	0.637173	0.637173	1.892263	-1.09057	CEACAM6
oxytetracycline-1553	1/100	0.561955	0.561955	0.637173	0.637173	1.892674	-1.09081	CCL20
orphenadrine-2356	1/100	0.561955	0.561955	0.637173	0.637173	1.896668	-1.09311	COL1A1
oxybuprocaine-1476	1/100	0.561955	0.561955	0.637173	0.637173	1.89863	-1.09424	CEACAM6
oxybutynin-6770	1/100	0.561955	0.561955	0.637173	0.637173	1.899425	-1.0947	FOS
nitrofural-5321	1/100	0.561955	0.561955	0.637173	0.637173	1.899807	-1.09492	S100A6
nordihydroguaiaretic acid-6182	1/100	0.561955	0.561955	0.637173	0.637173	1.900823	-1.09551	GPX2
NS-398-6911	1/100	0.561955	0.561955	0.637173	0.637173	1.902448	-1.09644	SOX9
paromomycin-4420	1/100	0.561955	0.561955	0.637173	0.637173	1.902749	-1.09662	COL1A1
NS-398-6897	1/100	0.561955	0.561955	0.637173	0.637173	1.903206	-1.09688	IFITM1
pentamidine-4396	1/100	0.561955	0.561955	0.637173	0.637173	1.904116	-1.09741	ITM2C
orlistat-6415	1/100	0.561955	0.561955	0.637173	0.637173	1.904376	-1.09756	C3
orlistat-6905	1/100	0.561955	0.561955	0.637173	0.637173	1.904812	-1.09781	PTMA
pempidine-6027	1/100	0.561955	0.561955	0.637173	0.637173	1.90745	-1.09933	SECTM1
omeprazole-2467	1/100	0.561955	0.561955	0.637173	0.637173	1.908175	-1.09975	TCN1
PHA-00846566E-7046	1/100	0.561955	0.561955	0.637173	0.637173	1.909247	-1.10036	JUND
noretynodrel-1818	1/100	0.561955	0.561955	0.637173	0.637173	1.909278	-1.10038	AP1S1
pentoxyverine-2623	1/100	0.561955	0.561955	0.637173	0.637173	1.909347	-1.10042	COL1A2
oxolinic acid-5094	1/100	0.561955	0.561955	0.637173	0.637173	1.909611	-1.10057	CEACAM5
pancuronium bromide-2909	1/100	0.561955	0.561955	0.637173	0.637173	1.912311	-1.10213	COL1A2
papaverine-6245	1/100	0.561955	0.561955	0.637173	0.637173	1.912635	-1.10232	ETS2

papaverine-1755	1/100	0.561955	0.561955	0.637173	0.637173	1.91371	-1.10294	IFITM3
palmatine-4957	1/100	0.561955	0.561955	0.637173	0.637173	1.913833	-1.10301	IFI6
orphenadrine-4537	1/100	0.561955	0.561955	0.637173	0.637173	1.91623	-1.10439	DUOX2
nordihydroguaiaretic acid-415	1/100	0.561955	0.561955	0.637173	0.637173	1.916423	-1.1045	GPX2
oxantel-6738	1/100	0.561955	0.561955	0.637173	0.637173	1.917556	-1.10515	IL32
pempidine-3926	1/100	0.561955	0.561955	0.637173	0.637173	1.918403	-1.10564	CTSE
paracetamol-3364	1/100	0.561955	0.561955	0.637173	0.637173	1.923017	-1.1083	PDZK1IP1
PHA-00851261E-3965	1/100	0.561955	0.561955	0.637173	0.637173	1.923486	-1.10857	COL1A1
PHA-00846566E-7086	1/100	0.561955	0.561955	0.637173	0.637173	1.923526	-1.10859	AP1S1
pentoxifylline-6021	1/100	0.561955	0.561955	0.637173	0.637173	1.929893	-1.11226	APP
PHA-00745360-4559	1/100	0.561955	0.561955	0.637173	0.637173	1.929922	-1.11228	CEACAM5
oxytetracycline-5772	1/100	0.561955	0.561955	0.637173	0.637173	1.930375	-1.11254	CEACAM6
nordihydroguaiaretic acid-2683	1/100	0.561955	0.561955	0.637173	0.637173	1.931571	-1.11323	COL1A2
PF-00562151-00-6912	1/100	0.561955	0.561955	0.637173	0.637173	1.931687	-1.1133	PHLDA1
PHA-00745360-4562	1/100	0.561955	0.561955	0.637173	0.637173	1.933698	-1.11445	SERPINA1
paclitaxel-640	1/100	0.561955	0.561955	0.637173	0.637173	1.933913	-1.11458	CEACAM5
penbutolol-2972	1/100	0.561955	0.561955	0.637173	0.637173	1.935073	-1.11525	PHLDA1
phenacetin-2832	1/100	0.561955	0.561955	0.637173	0.637173	1.938963	-1.11749	PHLDA1
pentamidine-639	1/100	0.561955	0.561955	0.637173	0.637173	1.939448	-1.11777	CEACAM5
orphenadrine-2318	1/100	0.561955	0.561955	0.637173	0.637173	1.941411	-1.1189	PHLDA1
PHA-00745360-3910	1/100	0.561955	0.561955	0.637173	0.637173	1.941801	-1.11913	IFI27
pergolide-7434	1/100	0.561955	0.561955	0.637173	0.637173	1.942816	-1.11971	PHLDA1
pentolonium-2343	1/100	0.561955	0.561955	0.637173	0.637173	1.94325	-1.11996	CKB
paromomycin-3356	1/100	0.561955	0.561955	0.637173	0.637173	1.944478	-1.12067	SECTM1
PF-00539745-00-5979	1/100	0.561955	0.561955	0.637173	0.637173	1.945689	-1.12137	AP1S1
PHA-00851261E-3857	1/100	0.561955	0.561955	0.637173	0.637173	1.947198	-1.12224	CTSE
pempidine-4307	1/100	0.561955	0.561955	0.637173	0.637173	1.947207	-1.12224	PHLDA1
phenacetin-3992	1/100	0.561955	0.561955	0.637173	0.637173	1.94827	-1.12285	QPCT
phensuximide-5097	1/100	0.561955	0.561955	0.637173	0.637173	1.948645	-1.12307	OLFM4
phenazopyridine-6234	1/100	0.561955	0.561955	0.637173	0.637173	1.950332	-1.12404	MMP3
paracetamol-6284	1/100	0.561955	0.561955	0.637173	0.637173	1.954133	-1.12623	MMP7
pentoxifylline-2127	1/100	0.561955	0.561955	0.637173	0.637173	1.955003	-1.12673	JUND
pempidine-3832	1/100	0.561955	0.561955	0.637173	0.637173	1.955176	-1.12683	COL1A1
pentamidine-2473	1/100	0.561955	0.561955	0.637173	0.637173	1.956559	-1.12763	TFF2
parthenolide-1736	1/100	0.561955	0.561955	0.637173	0.637173	1.958467	-1.12873	NQO1
PF-00562151-00-5917	1/100	0.561955	0.561955	0.637173	0.637173	1.960988	-1.13018	PHLDA1
pheneticillin-5763	1/100	0.561955	0.561955	0.637173	0.637173	1.964683	-1.13231	TMPRSS3

phenelzine-2357	1/100	0.561955	0.561955	0.637173	0.637173	1.968293	-1.13439	DCN
phenindione-2868	1/100	0.561955	0.561955	0.637173	0.637173	1.969135	-1.13488	PROM1
PHA-00851261E-4333	1/100	0.561955	0.561955	0.637173	0.637173	1.969492	-1.13508	RNF43
phenoxybenzamine-4652	1/100	0.561955	0.561955	0.637173	0.637173	1.969996	-1.13537	FOS
phenylpropanolamine-3217	1/100	0.561955	0.561955	0.637173	0.637173	1.970255	-1.13552	LYZ
oxyphenbutazone-6844	1/100	0.561955	0.561955	0.637173	0.637173	1.970313	-1.13556	SOX9
pepstatin-1328	1/100	0.561955	0.561955	0.637173	0.637173	1.970731	-1.1358	LY6E
phenindione-1718	1/100	0.561955	0.561955	0.637173	0.637173	1.97142	-1.1362	APP
PF-01378883-00-6410	1/100	0.561955	0.561955	0.637173	0.637173	1.971831	-1.13643	PSMB9
PF-00562151-00-6868	1/100	0.561955	0.561955	0.637173	0.637173	1.973363	-1.13732	PHLDA1
ozagrel-2942	1/100	0.561955	0.561955	0.637173	0.637173	1.975354	-1.13846	ATP1B1
pheniramine-1992	1/100	0.561955	0.561955	0.637173	0.637173	1.978354	-1.14019	ETS2
paclitaxel-1959	1/100	0.561955	0.561955	0.637173	0.637173	1.981066	-1.14175	PIGR
pilocarpine-2438	1/100	0.561955	0.561955	0.637173	0.637173	1.981898	-1.14223	IFITM1
pheneticillin-6239	1/100	0.561955	0.561955	0.637173	0.637173	1.982073	-1.14234	ETS2
PF-01378883-00-6405	1/100	0.561955	0.561955	0.637173	0.637173	1.983093	-1.14292	IFI6
PHA-00851261E-3776	1/100	0.561955	0.561955	0.637173	0.637173	1.983312	-1.14305	IL32
prasterone-6474	1/100	0.561955	0.561955	0.637173	0.637173	1.983814	-1.14334	SOX9
pipenzolate bromide-6821	1/100	0.561955	0.561955	0.637173	0.637173	1.984619	-1.1438	ITM2C
PHA-00851261E-3968	1/100	0.561955	0.561955	0.637173	0.637173	1.985508	-1.14431	SECTM1
piperidolate-3551	1/100	0.561955	0.561955	0.637173	0.637173	1.986818	-1.14507	DCN
pergolide-7271	1/100	0.561955	0.561955	0.637173	0.637173	1.987048	-1.1452	ITM2B
phenanthridinone-1115	1/100	0.561955	0.561955	0.637173	0.637173	1.98759	-1.14551	JUND
phenazone-1989	1/100	0.561955	0.561955	0.637173	0.637173	1.989019	-1.14634	GPX2
pentetic acid-4669	1/100	0.561955	0.561955	0.637173	0.637173	1.98998	-1.14689	SPARC
phenoxybenzamine-6451	1/100	0.561955	0.561955	0.637173	0.637173	1.990917	-1.14743	ETS2
parbendazole-4357	1/100	0.561955	0.561955	0.637173	0.637173	1.991376	-1.1477	IFI6
PHA-00745360-4384	1/100	0.561955	0.561955	0.637173	0.637173	1.992099	-1.14811	SECTM1
phthalylsulfathiazole-3033	1/100	0.561955	0.561955	0.637173	0.637173	1.99226	-1.14821	CD24
pramocaine-3811	1/100	0.561955	0.561955	0.637173	0.637173	1.992393	-1.14828	JUND
phenformin-21	1/100	0.561955	0.561955	0.637173	0.637173	1.994265	-1.14936	AP1S1
piracetam-1710	1/100	0.561955	0.561955	0.637173	0.637173	1.994511	-1.1495	APP
pilocarpine-6741	1/100	0.561955	0.561955	0.637173	0.637173	1.996342	-1.15056	AP1S1
pioglitazone-7528	1/100	0.561955	0.561955	0.637173	0.637173	1.997812	-1.15141	TFF2
pindolol-2075	1/100	0.561955	0.561955	0.637173	0.637173	1.998204	-1.15163	CCL20
phenacetin-2471	1/100	0.561955	0.561955	0.637173	0.637173	1.998532	-1.15182	CEACAM5
phensuximide-5522	1/100	0.561955	0.561955	0.637173	0.637173	1.99868	-1.15191	IFITM1

pimethixene-2395	1/100	0.561955	0.561955	0.637173	0.637173	1.998991	-1.15209	ATP1B1
pentoxyverine-1268	1/100	0.561955	0.561955	0.637173	0.637173	1.999572	-1.15242	TSPAN13
pizotifen-5491	1/100	0.561955	0.561955	0.637173	0.637173	2.002163	-1.15391	IFI27
pheniramine-4130	1/100	0.561955	0.561955	0.637173	0.637173	2.002933	-1.15436	COL1A1
pepstatin-4790	1/100	0.561955	0.561955	0.637173	0.637173	2.003558	-1.15472	DCN
piroxicam-2089	1/100	0.561955	0.561955	0.637173	0.637173	2.003922	-1.15493	S100A11
pipenzolate bromide-4484	1/100	0.561955	0.561955	0.637173	0.637173	2.005624	-1.15591	BMP4
pirenzepine-2234	1/100	0.561955	0.561955	0.637173	0.637173	2.006638	-1.15649	COL1A1
picotamide-6787	1/100	0.561955	0.561955	0.637173	0.637173	2.007417	-1.15694	C3
phenacetin-4111	1/100	0.561955	0.561955	0.637173	0.637173	2.008504	-1.15757	COL1A1
phentolamine-3779	1/100	0.561955	0.561955	0.637173	0.637173	2.009559	-1.15818	IFITM1
pirinixic acid-481	1/100	0.561955	0.561955	0.637173	0.637173	2.009562	-1.15818	MMP3
piracetam-5043	1/100	0.561955	0.561955	0.637173	0.637173	2.00969	-1.15825	IL32
phenindione-7289	1/100	0.561955	0.561955	0.637173	0.637173	2.011532	-1.15931	SPP1
piretanide-4490	1/100	0.561955	0.561955	0.637173	0.637173	2.015808	-1.16178	BMP4
piromidic acid-3335	1/100	0.561955	0.561955	0.637173	0.637173	2.016181	-1.16199	SECTM1
piretanide-3567	1/100	0.561955	0.561955	0.637173	0.637173	2.017316	-1.16265	IFI6
phenelzine-4538	1/100	0.561955	0.561955	0.637173	0.637173	2.020222	-1.16432	DCN
phentolamine-4336	1/100	0.561955	0.561955	0.637173	0.637173	2.021041	-1.16479	SERPINA1
pirlindole-3140	1/100	0.561955	0.561955	0.637173	0.637173	2.022139	-1.16543	IGFBP2
pheniramine-4012	1/100	0.561955	0.561955	0.637173	0.637173	2.024572	-1.16683	LUM
picrotoxinin-4260	1/100	0.561955	0.561955	0.637173	0.637173	2.026235	-1.16779	OLFM4
piribedil-3512	1/100	0.561955	0.561955	0.637173	0.637173	2.026393	-1.16788	PHLDA1
Prestwick-1082-3530	1/100	0.561955	0.561955	0.637173	0.637173	2.027596	-1.16857	C3
pivampicillin-5046	1/100	0.561955	0.561955	0.637173	0.637173	2.028985	-1.16937	COL1A1
PNU-0251126-7390	1/100	0.561955	0.561955	0.637173	0.637173	2.029406	-1.16961	CEACAM5
PNU-0230031-4291	1/100	0.561955	0.561955	0.637173	0.637173	2.031332	-1.17072	TFF3
piribedil-5434	1/100	0.561955	0.561955	0.637173	0.637173	2.031361	-1.17074	SLPI
Prestwick-665-7380	1/100	0.561955	0.561955	0.637173	0.637173	2.031511	-1.17083	PSMB9
pioglitazone-7506	1/100	0.561955	0.561955	0.637173	0.637173	2.033334	-1.17188	SOX9
picotamide-1387	1/100	0.561955	0.561955	0.637173	0.637173	2.033849	-1.17218	TCN1
picrotoxinin-4842	1/100	0.561955	0.561955	0.637173	0.637173	2.034064	-1.1723	COL1A1
podophyllotoxin-6103	1/100	0.561955	0.561955	0.637173	0.637173	2.037215	-1.17412	S100A11
phenylpropanolamine-5298	1/100	0.561955	0.561955	0.637173	0.637173	2.038382	-1.17479	S100A4
PNU-0230031-3629	1/100	0.561955	0.561955	0.637173	0.637173	2.038718	-1.17498	IFI27
prednicarbate-5119	1/100	0.561955	0.561955	0.637173	0.637173	2.03935	-1.17535	CEACAM5
piperacetazine-5834	1/100	0.561955	0.561955	0.637173	0.637173	2.039576	-1.17548	JUND

pinacidil-7437	1/100	0.561955	0.561955	0.637173	0.637173	2.040658	-1.1761	COL1A1
pioglitazone-5972	1/100	0.561955	0.561955	0.637173	0.637173	2.042115	-1.17694	SLPI
PNU-0230031-3732	1/100	0.561955	0.561955	0.637173	0.637173	2.04348	-1.17773	IL32
Prestwick-664-6033	1/100	0.561955	0.561955	0.637173	0.637173	2.044876	-1.17853	CTSE
pivampicillin-2945	1/100	0.561955	0.561955	0.637173	0.637173	2.045014	-1.17861	IFI6
prednisone-1478	1/100	0.561955	0.561955	0.637173	0.637173	2.047112	-1.17982	COL1A1
piracetam-2861	1/100	0.561955	0.561955	0.637173	0.637173	2.04735	-1.17996	AP1S1
prasterone-6673	1/100	0.561955	0.561955	0.637173	0.637173	2.047678	-1.18015	BMP4
piperine-4830	1/100	0.561955	0.561955	0.637173	0.637173	2.049721	-1.18132	COL1A1
pirinixic acid-495	1/100	0.561955	0.561955	0.637173	0.637173	2.053001	-1.18321	CEACAM6
primidone-3402	1/100	0.561955	0.561955	0.637173	0.637173	2.053701	-1.18362	TCN1
Prestwick-972-7266	1/100	0.561955	0.561955	0.637173	0.637173	2.055794	-1.18482	BMP4
pirlindole-6519	1/100	0.561955	0.561955	0.637173	0.637173	2.056858	-1.18544	RNF43
prazosin-5416	1/100	0.561955	0.561955	0.637173	0.637173	2.056962	-1.1855	PIGR
Prestwick-665-4704	1/100	0.561955	0.561955	0.637173	0.637173	2.057158	-1.18561	TCN1
picotamide-2070	1/100	0.561955	0.561955	0.637173	0.637173	2.057521	-1.18582	CCL20
praziquantel-1572	1/100	0.561955	0.561955	0.637173	0.637173	2.058073	-1.18614	CCL20
Prestwick-1083-6357	1/100	0.561955	0.561955	0.637173	0.637173	2.058296	-1.18626	COL1A1
piperacillin-4320	1/100	0.561955	0.561955	0.637173	0.637173	2.059935	-1.18721	TSPAN1
prednisolone-5101	1/100	0.561955	0.561955	0.637173	0.637173	2.059975	-1.18723	BMP4
piperacetazine-6152	1/100	0.561955	0.561955	0.637173	0.637173	2.060432	-1.1875	AP1S1
pindolol-4496	1/100	0.561955	0.561955	0.637173	0.637173	2.061895	-1.18834	IFITM1
pirenperone-2455	1/100	0.561955	0.561955	0.637173	0.637173	2.062562	-1.18872	S100A11
piromidic acid-4398	1/100	0.561955	0.561955	0.637173	0.637173	2.06602	-1.19072	DCN
Prestwick-1082-7267	1/100	0.561955	0.561955	0.637173	0.637173	2.067509	-1.19157	TSPAN1
Prestwick-972-6511	1/100	0.561955	0.561955	0.637173	0.637173	2.067793	-1.19174	CEACAM5
Prestwick-559-2877	1/100	0.561955	0.561955	0.637173	0.637173	2.067837	-1.19176	CEACAM5
PNU-0251126-7388	1/100	0.561955	0.561955	0.637173	0.637173	2.067859	-1.19178	AP1S1
Prestwick-1100-3798	1/100	0.561955	0.561955	0.637173	0.637173	2.067883	-1.19179	IFITM1
Prestwick-1103-7317	1/100	0.561955	0.561955	0.637173	0.637173	2.068653	-1.19223	COL1A1
proadifen-3446	1/100	0.561955	0.561955	0.637173	0.637173	2.069388	-1.19266	SOX9
pirinixic acid-464	1/100	0.561955	0.561955	0.637173	0.637173	2.071195	-1.1937	PIGR
Prestwick-967-7346	1/100	0.561955	0.561955	0.637173	0.637173	2.07157	-1.19392	PHLDA1
Prestwick-642-2815	1/100	0.561955	0.561955	0.637173	0.637173	2.074822	-1.19579	PIGR
Prestwick-665-3681	1/100	0.561955	0.561955	0.637173	0.637173	2.076311	-1.19665	PIGR
prednisone-1978	1/100	0.561955	0.561955	0.637173	0.637173	2.078135	-1.1977	ENC1
pizotifen-6513	1/100	0.561955	0.561955	0.637173	0.637173	2.078693	-1.19802	MMP3

probucol-5261	1/100	0.561955	0.561955	0.637173	0.637173	2.079816	-1.19867	C3
piroxicam-2252	1/100	0.561955	0.561955	0.637173	0.637173	2.081931	-1.19989	CEACAM5
Prestwick-1100-4356	1/100	0.561955	0.561955	0.637173	0.637173	2.082288	-1.20009	AP1S1
Prestwick-1084-3546	1/100	0.561955	0.561955	0.637173	0.637173	2.083634	-1.20087	COL1A1
Prestwick-675-7381	1/100	0.561955	0.561955	0.637173	0.637173	2.085021	-1.20167	IL32
Prestwick-1083-2976	1/100	0.561955	0.561955	0.637173	0.637173	2.085755	-1.20209	TFF2
Prestwick-682-4984	1/100	0.561955	0.561955	0.637173	0.637173	2.086343	-1.20243	PIGR
PNU-0251126-4714	1/100	0.561955	0.561955	0.637173	0.637173	2.087271	-1.20296	IFI6
pramocaine-6054	1/100	0.561955	0.561955	0.637173	0.637173	2.088529	-1.20369	COL1A1
Prestwick-1084-6767	1/100	0.561955	0.561955	0.637173	0.637173	2.089271	-1.20412	C3
Prestwick-642-4419	1/100	0.561955	0.561955	0.637173	0.637173	2.094435	-1.20709	ITM2C
prochlorperazine-1156	1/100	0.561955	0.561955	0.637173	0.637173	2.098254	-1.20929	CD14
pregnenolone-4218	1/100	0.561955	0.561955	0.637173	0.637173	2.098965	-1.2097	CEACAM6
Prestwick-689-5816	1/100	0.561955	0.561955	0.637173	0.637173	2.09984	-1.21021	TSPAN8
Prestwick-981-7464	1/100	0.561955	0.561955	0.637173	0.637173	2.100885	-1.21081	S100A4
Prestwick-675-6042	1/100	0.561955	0.561955	0.637173	0.637173	2.10107	-1.21092	SECTM1
Prestwick-682-2164	1/100	0.561955	0.561955	0.637173	0.637173	2.103394	-1.21226	IFI6
primidone-5323	1/100	0.561955	0.561955	0.637173	0.637173	2.104426	-1.21285	IFI6
prochlorperazine-5575	1/100	0.561955	0.561955	0.637173	0.637173	2.105225	-1.21331	TCN1
Prestwick-1103-6019	1/100	0.561955	0.561955	0.637173	0.637173	2.106501	-1.21405	CEACAM5
Prestwick-920-3118	1/100	0.561955	0.561955	0.637173	0.637173	2.106592	-1.2141	NQO1
PNU-0251126-3692	1/100	0.561955	0.561955	0.637173	0.637173	2.106648	-1.21413	SPP1
Prestwick-664-2178	1/100	0.561955	0.561955	0.637173	0.637173	2.10684	-1.21424	CD24
Prestwick-1103-3540	1/100	0.561955	0.561955	0.637173	0.637173	2.107823	-1.21481	S100A11
Prestwick-1080-4354	1/100	0.561955	0.561955	0.637173	0.637173	2.108359	-1.21512	OLFM4
Prestwick-972-3132	1/100	0.561955	0.561955	0.637173	0.637173	2.108493	-1.2152	IGFBP2
Prestwick-665-2186	1/100	0.561955	0.561955	0.637173	0.637173	2.112083	-1.21726	IFITM1
Prestwick-984-2903	1/100	0.561955	0.561955	0.637173	0.637173	2.112768	-1.21766	MMP3
promazine-3927	1/100	0.561955	0.561955	0.637173	0.637173	2.11312	-1.21786	PDZK1IP1
Prestwick-864-3994	1/100	0.561955	0.561955	0.637173	0.637173	2.113134	-1.21787	TSPAN8
primidone-3065	1/100	0.561955	0.561955	0.637173	0.637173	2.113973	-1.21835	PIGR
protoveratrine A-6618	1/100	0.561955	0.561955	0.637173	0.637173	2.116644	-1.21989	PIGR
prednisolone-265	1/100	0.561955	0.561955	0.637173	0.637173	2.116663	-1.2199	IFI27
prilocaine-3727	1/100	0.561955	0.561955	0.637173	0.637173	2.117781	-1.22055	APP
pramocaine-4368	1/100	0.561955	0.561955	0.637173	0.637173	2.118224	-1.2208	AP1S1
propranolol-5358	1/100	0.561955	0.561955	0.637173	0.637173	2.118824	-1.22115	IFI6
prilocaine-2314	1/100	0.561955	0.561955	0.637173	0.637173	2.120643	-1.2222	CEACAM5

Prestwick-983-7480	1/100	0.561955	0.561955	0.637173	0.637173	2.122724	-1.2234	MMP3
Prestwick-682-6638	1/100	0.561955	0.561955	0.637173	0.637173	2.123331	-1.22375	OLFM4
prednisone-4577	1/100	0.561955	0.561955	0.637173	0.637173	2.123884	-1.22407	MMP3
proadifen-5807	1/100	0.561955	0.561955	0.637173	0.637173	2.123903	-1.22408	JUND
probucol-1608	1/100	0.561955	0.561955	0.637173	0.637173	2.130512	-1.22789	CCL20
pridinol-2715	1/100	0.561955	0.561955	0.637173	0.637173	2.131408	-1.2284	CEACAM6
prochlorperazine-1215	1/100	0.561955	0.561955	0.637173	0.637173	2.131812	-1.22863	SPINK1
probenecid-4185	1/100	0.561955	0.561955	0.637173	0.637173	2.132412	-1.22898	IFITM1
prilocaine-3624	1/100	0.561955	0.561955	0.637173	0.637173	2.13319	-1.22943	CTGF
protoveratrine A-4963	1/100	0.561955	0.561955	0.637173	0.637173	2.134228	-1.23003	MMP3
Prestwick-857-3016	1/100	0.561955	0.561955	0.637173	0.637173	2.13691	-1.23157	GPX2
procyclidine-4817	1/100	0.561955	0.561955	0.637173	0.637173	2.137649	-1.232	IFI6
procainamide-5663	1/100	0.561955	0.561955	0.637173	0.637173	2.137963	-1.23218	MMP3
pyrimethamine-4194	1/100	0.561955	0.561955	0.637173	0.637173	2.138126	-1.23227	MMP3
proglumide-4337	1/100	0.561955	0.561955	0.637173	0.637173	2.138274	-1.23236	SERPINA1
Prestwick-692-4424	1/100	0.561955	0.561955	0.637173	0.637173	2.139545	-1.23309	SECTM1
procainamide-1263	1/100	0.561955	0.561955	0.637173	0.637173	2.140863	-1.23385	ATP1B1
propidium iodide-6104	1/100	0.561955	0.561955	0.637173	0.637173	2.143122	-1.23515	ITM2C
propidium iodide-6277	1/100	0.561955	0.561955	0.637173	0.637173	2.149602	-1.23889	IFI6
propantheline bromide-3352	1/100	0.561955	0.561955	0.637173	0.637173	2.150397	-1.23935	PIGR
Prestwick-689-7173	1/100	0.561955	0.561955	0.637173	0.637173	2.150941	-1.23966	SECTM1
Prestwick-689-6076	1/100	0.561955	0.561955	0.637173	0.637173	2.151375	-1.23991	FOS
proguanil-2944	1/100	0.561955	0.561955	0.637173	0.637173	2.153175	-1.24095	TCN1
propofol-3048	1/100	0.561955	0.561955	0.637173	0.637173	2.156751	-1.24301	CEACAM5
propidium iodide-2541	1/100	0.561955	0.561955	0.637173	0.637173	2.15858	-1.24406	IGFBP2
Prestwick-857-6635	1/100	0.561955	0.561955	0.637173	0.637173	2.159233	-1.24444	DCN
Prestwick-984-4948	1/100	0.561955	0.561955	0.637173	0.637173	2.160578	-1.24521	MMP3
raloxifene-2738	1/100	0.561955	0.561955	0.637173	0.637173	2.160692	-1.24528	PHLDA1
Prestwick-1080-4532	1/100	0.561955	0.561955	0.637173	0.637173	2.162798	-1.24649	RNF43
pseudopelletierine-1774	1/100	0.561955	0.561955	0.637173	0.637173	2.164541	-1.2475	PTPRO
procaine-1796	1/100	0.561955	0.561955	0.637173	0.637173	2.165314	-1.24794	AP1S1
procaine-5430	1/100	0.561955	0.561955	0.637173	0.637173	2.165707	-1.24817	SERPINA1
propoxycaine-6161	1/100	0.561955	0.561955	0.637173	0.637173	2.165748	-1.24819	PIGR
retrorsine-6601	1/100	0.561955	0.561955	0.637173	0.637173	2.168422	-1.24973	MMP12
procarbazine-5452	1/100	0.561955	0.561955	0.637173	0.637173	2.168962	-1.25005	COL1A2
propafenone-6336	1/100	0.561955	0.561955	0.637173	0.637173	2.170376	-1.25086	TGFBI
promazine-4308	1/100	0.561955	0.561955	0.637173	0.637173	2.17059	-1.25098	JUND

protriptyline-6338	1/100	0.561955	0.561955	0.637173	0.637173	2.172184	-1.2519	COL1A1
pyrantel-2097	1/100	0.561955	0.561955	0.637173	0.637173	2.172418	-1.25204	FOS
proguanil-5506	1/100	0.561955	0.561955	0.637173	0.637173	2.173412	-1.25261	AP1S1
quinethazone-4529	1/100	0.561955	0.561955	0.637173	0.637173	2.173937	-1.25291	JUND
ribostamycin-2705	1/100	0.561955	0.561955	0.637173	0.637173	2.174851	-1.25344	DCN
procarbazine-2971	1/100	0.561955	0.561955	0.637173	0.637173	2.174858	-1.25344	JUND
raloxifene-6235	1/100	0.561955	0.561955	0.637173	0.637173	2.175587	-1.25386	JUND
propylthiouracil-4076	1/100	0.561955	0.561955	0.637173	0.637173	2.17572	-1.25394	RNF43
propantheline bromide-4798	1/100	0.561955	0.561955	0.637173	0.637173	2.177002	-1.25468	COL1A1
procainamide-2618	1/100	0.561955	0.561955	0.637173	0.637173	2.177902	-1.2552	IFI6
pronetalol-2902	1/100	0.561955	0.561955	0.637173	0.637173	2.179108	-1.25589	TFF2
procyclidine-2991	1/100	0.561955	0.561955	0.637173	0.637173	2.180158	-1.2565	PIGR
probucol-4666	1/100	0.561955	0.561955	0.637173	0.637173	2.18554	-1.2596	CEACAM5
pyrimethamine-4779	1/100	0.561955	0.561955	0.637173	0.637173	2.18681	-1.26033	IFI6
promethazine-6717	1/100	0.561955	0.561955	0.637173	0.637173	2.192528	-1.26363	CXCL1
quinpirole-456	1/100	0.561955	0.561955	0.637173	0.637173	2.194978	-1.26504	SLPI
propidium iodide-5803	1/100	0.561955	0.561955	0.637173	0.637173	2.195236	-1.26519	TGFBI
protriptyline-3119	1/100	0.561955	0.561955	0.637173	0.637173	2.195277	-1.26521	CTSE
proxymetacaine-3113	1/100	0.561955	0.561955	0.637173	0.637173	2.196141	-1.26571	COL1A2
propofol-6707	1/100	0.561955	0.561955	0.637173	0.637173	2.196171	-1.26573	FOS
pyrazinamide-6617	1/100	0.561955	0.561955	0.637173	0.637173	2.197642	-1.26657	COL1A2
ranitidine-1404	1/100	0.561955	0.561955	0.637173	0.637173	2.19774	-1.26663	PTMA
prochlorperazine-1286	1/100	0.561955	0.561955	0.637173	0.637173	2.19935	-1.26756	AP1S1
profenamine-6697	1/100	0.561955	0.561955	0.637173	0.637173	2.199404	-1.26759	CXCL1
prochlorperazine-1640	1/100	0.561955	0.561955	0.637173	0.637173	2.201135	-1.26859	APP
quinethazone-3875	1/100	0.561955	0.561955	0.637173	0.637173	2.201299	-1.26868	IFI6
pseudopelletierine-2766	1/100	0.561955	0.561955	0.637173	0.637173	2.201923	-1.26904	TFF2
proglumide-2363	1/100	0.561955	0.561955	0.637173	0.637173	2.202787	-1.26954	TFF2
R-atenolol-4259	1/100	0.561955	0.561955	0.637173	0.637173	2.204457	-1.2705	DUOX2
pyrvinium-6339	1/100	0.561955	0.561955	0.637173	0.637173	2.20563	-1.27118	S100P
rosiglitazone-1233	1/100	0.561955	0.561955	0.637173	0.637173	2.205808	-1.27128	CCL20
pyrimethamine-1974	1/100	0.561955	0.561955	0.637173	0.637173	2.206293	-1.27156	CEACAM5
quercetin-4264	1/100	0.561955	0.561955	0.637173	0.637173	2.20703	-1.27199	LYZ
resveratrol-622	1/100	0.561955	0.561955	0.637173	0.637173	2.208555	-1.27286	IFNGR1
promethazine-6477	1/100	0.561955	0.561955	0.637173	0.637173	2.208578	-1.27288	MMP3
rofecoxib-205	1/100	0.561955	0.561955	0.637173	0.637173	2.213815	-1.2759	CEACAM6
repaglinide-6135	1/100	0.561955	0.561955	0.637173	0.637173	2.214179	-1.27611	CCL20

rofecoxib-463	1/100	0.561955	0.561955	0.637173	0.637173	2.21437	-1.27622	PIGR
pyrithyldione-2740	1/100	0.561955	0.561955	0.637173	0.637173	2.215761	-1.27702	CEACAM5
pyrithyldione-3482	1/100	0.561955	0.561955	0.637173	0.637173	2.21699	-1.27773	IFI6
quinpirole-7481	1/100	0.561955	0.561955	0.637173	0.637173	2.217279	-1.27789	IFITM1
propantheline bromide-3013	1/100	0.561955	0.561955	0.637173	0.637173	2.217409	-1.27797	MMP3
ricinine-2505	1/100	0.561955	0.561955	0.637173	0.637173	2.217697	-1.27813	CEACAM5
raloxifene-388	1/100	0.561955	0.561955	0.637173	0.637173	2.218924	-1.27884	LCN2
prochlorperazine-4439	1/100	0.561955	0.561955	0.637173	0.637173	2.219027	-1.2789	JUND
proxyphylline-6494	1/100	0.561955	0.561955	0.637173	0.637173	2.219605	-1.27923	COL1A1
rilmenidine-5532	1/100	0.561955	0.561955	0.637173	0.637173	2.221129	-1.28011	IFI6
raubasine-5459	1/100	0.561955	0.561955	0.637173	0.637173	2.224247	-1.28191	SPARC
raloxifene-202	1/100	0.561955	0.561955	0.637173	0.637173	2.224866	-1.28226	TPI1
raloxifene-376	1/100	0.561955	0.561955	0.637173	0.637173	2.228084	-1.28412	TGFBI
raloxifene-3480	1/100	0.561955	0.561955	0.637173	0.637173	2.230725	-1.28564	RNF43
retrorsine-4946	1/100	0.561955	0.561955	0.637173	0.637173	2.230765	-1.28566	BMP4
pyrazinamide-4962	1/100	0.561955	0.561955	0.637173	0.637173	2.230842	-1.28571	CEACAM5
rilmenidine-6512	1/100	0.561955	0.561955	0.637173	0.637173	2.232157	-1.28647	AP1S1
riboflavin-4485	1/100	0.561955	0.561955	0.637173	0.637173	2.233586	-1.28729	JUND
rosiglitazone-6950	1/100	0.561955	0.561955	0.637173	0.637173	2.233809	-1.28742	SERPINA1
rolitetracycline-5331	1/100	0.561955	0.561955	0.637173	0.637173	2.234325	-1.28772	GPX2
reserpine-4203	1/100	0.561955	0.561955	0.637173	0.637173	2.235695	-1.28851	TSPAN8
ranitidine-5425	1/100	0.561955	0.561955	0.637173	0.637173	2.235854	-1.2886	COL1A1
pyridoxine-5813	1/100	0.561955	0.561955	0.637173	0.637173	2.236481	-1.28896	AP1S1
pseudopelletierine-5828	1/100	0.561955	0.561955	0.637173	0.637173	2.23713	-1.28933	DCN
rimexolone-3516	1/100	0.561955	0.561955	0.637173	0.637173	2.239081	-1.29046	IFI27
retrorsine-2784	1/100	0.561955	0.561955	0.637173	0.637173	2.239947	-1.29096	COL1A1
remoxipride-3124	1/100	0.561955	0.561955	0.637173	0.637173	2.24043	-1.29123	PIGR
ramifenazone-5879	1/100	0.561955	0.561955	0.637173	0.637173	2.241571	-1.29189	C3
saquinavir-3549	1/100	0.561955	0.561955	0.637173	0.637173	2.24214	-1.29222	BMP4
riboflavin-6822	1/100	0.561955	0.561955	0.637173	0.637173	2.245848	-1.29436	SOX9
R-atenolol-2855	1/100	0.561955	0.561955	0.637173	0.637173	2.246512	-1.29474	SOX9
rifampicin-4008	1/100	0.561955	0.561955	0.637173	0.637173	2.24724	-1.29516	CEACAM6
ribavirin-3142	1/100	0.561955	0.561955	0.637173	0.637173	2.248367	-1.29581	CCL20
repaglinide-7216	1/100	0.561955	0.561955	0.637173	0.637173	2.25195	-1.29787	TFF2
pyridoxine-2751	1/100	0.561955	0.561955	0.637173	0.637173	2.253992	-1.29905	AP1S1
rolitetracycline-3369	1/100	0.561955	0.561955	0.637173	0.637173	2.255123	-1.2997	SPINK1
remoxipride-6503	1/100	0.561955	0.561955	0.637173	0.637173	2.255379	-1.29985	MMP3

rimexolone-2955	1/100	0.561955	0.561955	0.637173	0.637173	2.255948	-1.30018	CCL20
scopolamine N-oxide-6335	1/100	0.561955	0.561955	0.637173	0.637173	2.257459	-1.30105	SERPINA1
SB-202190-7054	1/100	0.561955	0.561955	0.637173	0.637173	2.258583	-1.3017	IFITM1
SB-203580-7066	1/100	0.561955	0.561955	0.637173	0.637173	2.258969	-1.30192	APP
SB-203580-6894	1/100	0.561955	0.561955	0.637173	0.637173	2.259209	-1.30206	APP
SC-19220-7060	1/100	0.561955	0.561955	0.637173	0.637173	2.260036	-1.30253	COL1A1
riboflavin-1767	1/100	0.561955	0.561955	0.637173	0.637173	2.260549	-1.30283	IFITM3
sanguinarine-4168	1/100	0.561955	0.561955	0.637173	0.637173	2.261535	-1.3034	FOS
quinisocaine-2807	1/100	0.561955	0.561955	0.637173	0.637173	2.262666	-1.30405	CTSE
rosiglitazone-1174	1/100	0.561955	0.561955	0.637173	0.637173	2.265882	-1.3059	AP1S1
santonin-3795	1/100	0.561955	0.561955	0.637173	0.637173	2.266381	-1.30619	PIGR
pyrvinium-3518	1/100	0.561955	0.561955	0.637173	0.637173	2.269064	-1.30774	ID3
riluzole-2295	1/100	0.561955	0.561955	0.637173	0.637173	2.269171	-1.3078	SECTM1
riluzole-3666	1/100	0.561955	0.561955	0.637173	0.637173	2.269999	-1.30828	APP
resveratrol-958	1/100	0.561955	0.561955	0.637173	0.637173	2.2701	-1.30833	TXNIP
sirolimus-5602	1/100	0.561955	0.561955	0.637173	0.637173	2.270198	-1.30839	TXNIP
rilmenidine-3133	1/100	0.561955	0.561955	0.637173	0.637173	2.270272	-1.30843	PHLDA1
sirolimus-1022	1/100	0.561955	0.561955	0.637173	0.637173	2.271246	-1.309	TXNIP
seneciphylline-2797	1/100	0.561955	0.561955	0.637173	0.637173	2.272269	-1.30958	DCN
quipazine-1789	1/100	0.561955	0.561955	0.637173	0.637173	2.273188	-1.31011	FOS
S-propranolol-5444	1/100	0.561955	0.561955	0.637173	0.637173	2.27334	-1.3102	S100A11
ronidazole-6134	1/100	0.561955	0.561955	0.637173	0.637173	2.273664	-1.31039	TMPRSS3
sirolimus-5218	1/100	0.561955	0.561955	0.637173	0.637173	2.273836	-1.31049	TXNIP
rofecoxib-166	1/100	0.561955	0.561955	0.637173	0.637173	2.275034	-1.31118	TSPAN1
scopolamine N-oxide-2262	1/100	0.561955	0.561955	0.637173	0.637173	2.275082	-1.31121	CEACAM5
sirolimus-6927	1/100	0.561955	0.561955	0.637173	0.637173	2.276751	-1.31217	TXNIP
risperidone-3508	1/100	0.561955	0.561955	0.637173	0.637173	2.276874	-1.31224	AP1S1
sirolimus-402	1/100	0.561955	0.561955	0.637173	0.637173	2.278249	-1.31303	IFI27
santonin-4353	1/100	0.561955	0.561955	0.637173	0.637173	2.27827	-1.31304	COL1A1
semustine-7540	1/100	0.561955	0.561955	0.637173	0.637173	2.278778	-1.31334	RCN1
salbutamol-2306	1/100	0.561955	0.561955	0.637173	0.637173	2.279032	-1.31348	PHLDA1
salbutamol-2344	1/100	0.561955	0.561955	0.637173	0.637173	2.280713	-1.31445	CD24
simvastatin-3002	1/100	0.561955	0.561955	0.637173	0.637173	2.281787	-1.31507	PIGR
selegiline-2826	1/100	0.561955	0.561955	0.637173	0.637173	2.281873	-1.31512	PSMB9
riluzole-2334	1/100	0.561955	0.561955	0.637173	0.637173	2.281999	-1.31519	TFF2
selegiline-4065	1/100	0.561955	0.561955	0.637173	0.637173	2.282403	-1.31543	IL32
rimexolone-5092	1/100	0.561955	0.561955	0.637173	0.637173	2.282704	-1.3156	SPINK1

rimexolone-5517	1/100	0.561955	0.561955	0.637173	0.637173	2.284624	-1.31671	IFITM1
scopolamine N-oxide-2099	1/100	0.561955	0.561955	0.637173	0.637173	2.285078	-1.31697	SPINK1
sisomicin-4132	1/100	0.561955	0.561955	0.637173	0.637173	2.285334	-1.31711	COL1A1
rosiglitazone-5593	1/100	0.561955	0.561955	0.637173	0.637173	2.285696	-1.31732	ITM2C
sirolimus-1080	1/100	0.561955	0.561955	0.637173	0.637173	2.285734	-1.31734	TXNIP
resveratrol-1715	1/100	0.561955	0.561955	0.637173	0.637173	2.288113	-1.31872	PHLDA1
ribavirin-6521	1/100	0.561955	0.561955	0.637173	0.637173	2.288495	-1.31894	COL1A1
raloxifene-5759	1/100	0.561955	0.561955	0.637173	0.637173	2.291934	-1.32092	RNF43
sirolimus-1001	1/100	0.561955	0.561955	0.637173	0.637173	2.291973	-1.32094	TXNIP
SB-203580-7061	1/100	0.561955	0.561955	0.637173	0.637173	2.29316	-1.32163	ETS2
rolipram-3072	1/100	0.561955	0.561955	0.637173	0.637173	2.29437	-1.32232	IFITM1
rosiglitazone-369	1/100	0.561955	0.561955	0.637173	0.637173	2.295838	-1.32317	ENC1
selegiline-2465	1/100	0.561955	0.561955	0.637173	0.637173	2.296691	-1.32366	PHLDA1
SB-203580-6899	1/100	0.561955	0.561955	0.637173	0.637173	2.29686	-1.32376	ITM2B
spiramycin-3419	1/100	0.561955	0.561955	0.637173	0.637173	2.29742	-1.32408	C3
SB-203580-6915	1/100	0.561955	0.561955	0.637173	0.637173	2.298775	-1.32486	APP
risperidone-2947	1/100	0.561955	0.561955	0.637173	0.637173	2.299447	-1.32525	IGFBP2
seneciphylline-4238	1/100	0.561955	0.561955	0.637173	0.637173	2.300071	-1.32561	ENC1
scriptaid-6901	1/100	0.561955	0.561955	0.637173	0.637173	2.300642	-1.32594	APP
spironolactone-2226	1/100	0.561955	0.561955	0.637173	0.637173	2.301939	-1.32668	COL1A1
sirolimus-4431	1/100	0.561955	0.561955	0.637173	0.637173	2.302287	-1.32688	PIGR
SC-19220-7095	1/100	0.561955	0.561955	0.637173	0.637173	2.304306	-1.32805	CTGF
santonin-3877	1/100	0.561955	0.561955	0.637173	0.637173	2.307441	-1.32986	COL1A1
securinine-2729	1/100	0.561955	0.561955	0.637173	0.637173	2.307905	-1.33012	NQO1
sirolimus-7001	1/100	0.561955	0.561955	0.637173	0.637173	2.308301	-1.33035	TXNIP
salsolidin-4226	1/100	0.561955	0.561955	0.637173	0.637173	2.310164	-1.33142	APP
spiramycin-3938	1/100	0.561955	0.561955	0.637173	0.637173	2.311362	-1.33212	PDZK1IP1
salsolinol-4816	1/100	0.561955	0.561955	0.637173	0.637173	2.311567	-1.33223	COL1A1
rosiglitazone-6992	1/100	0.561955	0.561955	0.637173	0.637173	2.312078	-1.33253	TMPRSS3
semustine-7492	1/100	0.561955	0.561955	0.637173	0.637173	2.314013	-1.33364	JUND
sirolimus-1162	1/100	0.561955	0.561955	0.637173	0.637173	2.315045	-1.33424	CD24
skimmianine-5766	1/100	0.561955	0.561955	0.637173	0.637173	2.315984	-1.33478	TGFBI
scopolamine-3018	1/100	0.561955	0.561955	0.637173	0.637173	2.31614	-1.33487	IFI6
rosiglitazone-6192	1/100	0.561955	0.561955	0.637173	0.637173	2.318302	-1.33612	COL1A1
scopolamine N-oxide-5436	1/100	0.561955	0.561955	0.637173	0.637173	2.318341	-1.33614	IFI6
sodium phenylbutyrate-407	1/100	0.561955	0.561955	0.637173	0.637173	2.318788	-1.33639	PTMA
salsolinol-2791	1/100	0.561955	0.561955	0.637173	0.637173	2.321332	-1.33786	COL1A2

scopolamine N-oxide-1415	1/100	0.561955	0.561955	0.637173	0.637173	2.321494	-1.33796	CEACAM5
sodium phenylbutyrate-408	1/100	0.561955	0.561955	0.637173	0.637173	2.323709	-1.33923	TXNIP
spaglumic acid-2962	1/100	0.561955	0.561955	0.637173	0.637173	2.324153	-1.33949	NQO1
sparteine-2790	1/100	0.561955	0.561955	0.637173	0.637173	2.325284	-1.34014	AP1S1
sirolimus-5932	1/100	0.561955	0.561955	0.637173	0.637173	2.329933	-1.34282	IFI6
SB-202190-6909	1/100	0.561955	0.561955	0.637173	0.637173	2.331812	-1.3439	PHLDA1
sulfadoxine-3547	1/100	0.561955	0.561955	0.637173	0.637173	2.332257	-1.34416	CEACAM5
simvastatin-4244	1/100	0.561955	0.561955	0.637173	0.637173	2.337366	-1.3471	S100P
sertaconazole-6811	1/100	0.561955	0.561955	0.637173	0.637173	2.337371	-1.34711	RNF43
spiradoline-3818	1/100	0.561955	0.561955	0.637173	0.637173	2.339337	-1.34824	IFITM1
sirolimus-921	1/100	0.561955	0.561955	0.637173	0.637173	2.344447	-1.35118	COL1A1
spiperone-5777	1/100	0.561955	0.561955	0.637173	0.637173	2.345586	-1.35184	S100P
sirolimus-6940	1/100	0.561955	0.561955	0.637173	0.637173	2.346156	-1.35217	TXNIP
sulfadimidine-2560	1/100	0.561955	0.561955	0.637173	0.637173	2.351498	-1.35525	IFITM1
spaglumic acid-7465	1/100	0.561955	0.561955	0.637173	0.637173	2.353491	-1.3564	COL1A1
sodium phenylbutyrate-341	1/100	0.561955	0.561955	0.637173	0.637173	2.353606	-1.35646	JUND
sirolimus-5975	1/100	0.561955	0.561955	0.637173	0.637173	2.355957	-1.35782	TXNIP
sirolimus-1207	1/100	0.561955	0.561955	0.637173	0.637173	2.356292	-1.35801	SPINK1
sulfathiazole-1963	1/100	0.561955	0.561955	0.637173	0.637173	2.356759	-1.35828	IFITM3
spiramycin-4319	1/100	0.561955	0.561955	0.637173	0.637173	2.356882	-1.35835	COL1A2
sodium phenylbutyrate-434	1/100	0.561955	0.561955	0.637173	0.637173	2.357473	-1.35869	CEACAM5
spectinomycin-4773	1/100	0.561955	0.561955	0.637173	0.637173	2.35822	-1.35912	COL1A1
SR-95639A-3272	1/100	0.561955	0.561955	0.637173	0.637173	2.358415	-1.35923	COL1A1
sulfamethoxypyridazine-2550	1/100	0.561955	0.561955	0.637173	0.637173	2.358912	-1.35952	DCN
scoulerine-5111	1/100	0.561955	0.561955	0.637173	0.637173	2.35974	-1.36	HSPB1
sirolimus-2702	1/100	0.561955	0.561955	0.637173	0.637173	2.360427	-1.36039	OLFM4
skimmianine-2504	1/100	0.561955	0.561955	0.637173	0.637173	2.360473	-1.36042	PHLDA1
spironolactone-2064	1/100	0.561955	0.561955	0.637173	0.637173	2.360902	-1.36067	IFI6
sulconazole-3373	1/100	0.561955	0.561955	0.637173	0.637173	2.361182	-1.36083	FOS
scopoletin-3131	1/100	0.561955	0.561955	0.637173	0.637173	2.361587	-1.36106	CEACAM6
STOCK1N-35874-6561	1/100	0.561955	0.561955	0.637173	0.637173	2.363043	-1.3619	AP1S1
sulmazole-4127	1/100	0.561955	0.561955	0.637173	0.637173	2.36397	-1.36243	IFI6
solanine-2152	1/100	0.561955	0.561955	0.637173	0.637173	2.36522	-1.36316	PROM1
skimmianine-6066	1/100	0.561955	0.561955	0.637173	0.637173	2.366429	-1.36385	IFI6
sulfacetamide-1695	1/100	0.561955	0.561955	0.637173	0.637173	2.366973	-1.36417	GPX2
succinylsulfathiazole-4847	1/100	0.561955	0.561955	0.637173	0.637173	2.368258	-1.36491	MMP3
STOCK1N-35215-6380	1/100	0.561955	0.561955	0.637173	0.637173	2.368594	-1.3651	COL1A1

seneciphylline-2140	1/100	0.561955	0.561955	0.637173	0.637173	2.369851	-1.36582	CEACAM6
solanine-2808	1/100	0.561955	0.561955	0.637173	0.637173	2.369864	-1.36583	COL1A1
sulfametoxydiazine-3453	1/100	0.561955	0.561955	0.637173	0.637173	2.372438	-1.36732	COL1A1
sulfafurazole-1603	1/100	0.561955	0.561955	0.637173	0.637173	2.37407	-1.36826	CCL20
spectinomycin-3327	1/100	0.561955	0.561955	0.637173	0.637173	2.374426	-1.36846	SPARC
SR-95531-4820	1/100	0.561955	0.561955	0.637173	0.637173	2.374778	-1.36866	MMP3
solasodine-3924	1/100	0.561955	0.561955	0.637173	0.637173	2.375348	-1.36899	COL1A1
SR-95639A-6632	1/100	0.561955	0.561955	0.637173	0.637173	2.376221	-1.3695	RNF43
sulfachlorpyridazine-2191	1/100	0.561955	0.561955	0.637173	0.637173	2.376423	-1.36961	CEACAM6
sulfamerazine-3718	1/100	0.561955	0.561955	0.637173	0.637173	2.378415	-1.37076	SLCO5A1
sirolimus-326	1/100	0.561955	0.561955	0.637173	0.637173	2.378602	-1.37087	ITM2B
sulfacetamide-1817	1/100	0.561955	0.561955	0.637173	0.637173	2.380699	-1.37208	AP1S1
syrosingopine-6213	1/100	0.561955	0.561955	0.637173	0.637173	2.382447	-1.37308	TXNIP
sitosterol-7332	1/100	0.561955	0.561955	0.637173	0.637173	2.384267	-1.37413	CEACAM5
sulindac-5528	1/100	0.561955	0.561955	0.637173	0.637173	2.385128	-1.37463	COL1A1
sulfabenzamide-2159	1/100	0.561955	0.561955	0.637173	0.637173	2.391279	-1.37817	DCN
suprofen-3343	1/100	0.561955	0.561955	0.637173	0.637173	2.392468	-1.37886	SECTM1
spiperone-1559	1/100	0.561955	0.561955	0.637173	0.637173	2.394628	-1.3801	PIGR
sulindac-1857	1/100	0.561955	0.561955	0.637173	0.637173	2.396742	-1.38132	MMP3
tacrine-6698	1/100	0.561955	0.561955	0.637173	0.637173	2.399015	-1.38263	CTSE
sulfamethoxazole-7366	1/100	0.561955	0.561955	0.637173	0.637173	2.399505	-1.38291	TXNIP
STOCK1N-28457-6869	1/100	0.561955	0.561955	0.637173	0.637173	2.400141	-1.38328	SERPINA1
sirolimus-1045	1/100	0.561955	0.561955	0.637173	0.637173	2.401548	-1.38409	TXNIP
sulfafurazole-5257	1/100	0.561955	0.561955	0.637173	0.637173	2.402012	-1.38436	C3
tanespimycin-4430	1/100	0.561955	0.561955	0.637173	0.637173	2.402143	-1.38444	HSPB1
sulfasalazine-5446	1/100	0.561955	0.561955	0.637173	0.637173	2.403083	-1.38498	PHLDA1
sulfacetamide-6349	1/100	0.561955	0.561955	0.637173	0.637173	2.40625	-1.3868	IGFBP2
sulfadoxine-7205	1/100	0.561955	0.561955	0.637173	0.637173	2.406408	-1.38689	SOX9
sulfadiazine-1852	1/100	0.561955	0.561955	0.637173	0.637173	2.407065	-1.38727	ITM2B
sulfanilamide-4474	1/100	0.561955	0.561955	0.637173	0.637173	2.407577	-1.38757	MMP3
sulmazole-2153	1/100	0.561955	0.561955	0.637173	0.637173	2.408434	-1.38806	APP
sulfasalazine-2882	1/100	0.561955	0.561955	0.637173	0.637173	2.411458	-1.3898	SPINK1
sulfadimethoxine-3702	1/100	0.561955	0.561955	0.637173	0.637173	2.413747	-1.39112	COL1A1
syrosingopine-2753	1/100	0.561955	0.561955	0.637173	0.637173	2.415665	-1.39223	TXNIP
tetrahydroalstonine-6209	1/100	0.561955	0.561955	0.637173	0.637173	2.416746	-1.39285	AP1S1
spectinomycin-4187	1/100	0.561955	0.561955	0.637173	0.637173	2.417244	-1.39314	IFI27
sulfamerazine-2181	1/100	0.561955	0.561955	0.637173	0.637173	2.420465	-1.395	TFF2

streptozocin-2535	1/100	0.561955	0.561955	0.637173	0.637173	2.420526	-1.39503	MMP3
suprofen-3005	1/100	0.561955	0.561955	0.637173	0.637173	2.420946	-1.39527	COL1A1
sulfamethoxazole-4690	1/100	0.561955	0.561955	0.637173	0.637173	2.421055	-1.39534	SERPINA1
sulfamethizole-6272	1/100	0.561955	0.561955	0.637173	0.637173	2.425929	-1.39814	CEACAM6
STOCK1N-35696-6564	1/100	0.561955	0.561955	0.637173	0.637173	2.426266	-1.39834	ID3
sulfamethizole-6099	1/100	0.561955	0.561955	0.637173	0.637173	2.426412	-1.39842	DCN
sulmazole-4009	1/100	0.561955	0.561955	0.637173	0.637173	2.427797	-1.39922	CEACAM6
tanespimycin-1006	1/100	0.561955	0.561955	0.637173	0.637173	2.427867	-1.39926	BMP4
sulfaquinoxaline-6788	1/100	0.561955	0.561955	0.637173	0.637173	2.429549	-1.40023	IFI27
SR-95531-3253	1/100	0.561955	0.561955	0.637173	0.637173	2.430204	-1.40061	AP1S1
sulfaphenazole-1673	1/100	0.561955	0.561955	0.637173	0.637173	2.431216	-1.40119	S100A11
suramin sodium-7529	1/100	0.561955	0.561955	0.637173	0.637173	2.43472	-1.40321	BMP4
sulfafurazole-3218	1/100	0.561955	0.561955	0.637173	0.637173	2.435151	-1.40346	FOS
thiostrepton-4385	1/100	0.561955	0.561955	0.637173	0.637173	2.436091	-1.404	DCN
sulfametoxydiazine-6212	1/100	0.561955	0.561955	0.637173	0.637173	2.436669	-1.40433	ETS2
sulfamonomethoxine-3484	1/100	0.561955	0.561955	0.637173	0.637173	2.437068	-1.40456	COL1A1
sulfadimidine-3765	1/100	0.561955	0.561955	0.637173	0.637173	2.437136	-1.4046	IL32
SR-95639A-1336	1/100	0.561955	0.561955	0.637173	0.637173	2.43791	-1.40505	APP
tanespimycin-4442	1/100	0.561955	0.561955	0.637173	0.637173	2.43874	-1.40553	HSPB1
tamoxifen-2050	1/100	0.561955	0.561955	0.637173	0.637173	2.439597	-1.40602	COL1A2
tenoxicam-2860	1/100	0.561955	0.561955	0.637173	0.637173	2.43973	-1.4061	PIGR
tetracycline-2243	1/100	0.561955	0.561955	0.637173	0.637173	2.439921	-1.40621	SLPI
strophanthidin-7182	1/100	0.561955	0.561955	0.637173	0.637173	2.442221	-1.40753	SECTM1
sulfanilamide-2709	1/100	0.561955	0.561955	0.637173	0.637173	2.443042	-1.40801	CEACAM6
tanespimycin-6184	1/100	0.561955	0.561955	0.637173	0.637173	2.443738	-1.40841	HSPB1
theophylline-2986	1/100	0.561955	0.561955	0.637173	0.637173	2.444465	-1.40883	FXYD3
tanespimycin-6978	1/100	0.561955	0.561955	0.637173	0.637173	2.444916	-1.40909	BMP4
tacrolimus-378	1/100	0.561955	0.561955	0.637173	0.637173	2.446401	-1.40994	IFITM2
thioridazine-417	1/100	0.561955	0.561955	0.637173	0.637173	2.447031	-1.41031	JUND
suprofen-4123	1/100	0.561955	0.561955	0.637173	0.637173	2.447908	-1.41081	SECTM1
sulfathiazole-4183	1/100	0.561955	0.561955	0.637173	0.637173	2.448257	-1.41101	IFITM1
tanespimycin-916	1/100	0.561955	0.561955	0.637173	0.637173	2.448411	-1.4111	COL1A1
tanespimycin-986	1/100	0.561955	0.561955	0.637173	0.637173	2.448554	-1.41118	COL1A2
tanespimycin-5222	1/100	0.561955	0.561955	0.637173	0.637173	2.450268	-1.41217	SOX9
sulfamethoxypyridazine-4733	1/100	0.561955	0.561955	0.637173	0.637173	2.451464	-1.41286	COL1A1
tanespimycin-5958	1/100	0.561955	0.561955	0.637173	0.637173	2.452553	-1.41349	HSPB1
sulindac sulfide-308	1/100	0.561955	0.561955	0.637173	0.637173	2.45363	-1.41411	ID3

sulfamerazine-3616	1/100	0.561955	0.561955	0.637173	0.637173	2.455333	-1.41509	CEACAM5
tanespimycin-2685	1/100	0.561955	0.561955	0.637173	0.637173	2.456592	-1.41582	HSPB1
sulindac-1815	1/100	0.561955	0.561955	0.637173	0.637173	2.45809	-1.41668	AP1S1
thalidomide-2095	1/100	0.561955	0.561955	0.637173	0.637173	2.458988	-1.4172	DCN
sulfasalazine-1733	1/100	0.561955	0.561955	0.637173	0.637173	2.459751	-1.41764	CTSE
sulfapyridine-6799	1/100	0.561955	0.561955	0.637173	0.637173	2.46382	-1.41998	ITM2C
tiaprofenic acid-2852	1/100	0.561955	0.561955	0.637173	0.637173	2.466348	-1.42144	ITM2C
terconazole-2844	1/100	0.561955	0.561955	0.637173	0.637173	2.467238	-1.42195	PROM1
tetracycline-5757	1/100	0.561955	0.561955	0.637173	0.637173	2.468422	-1.42263	MMP3
timolol-6483	1/100	0.561955	0.561955	0.637173	0.637173	2.46844	-1.42264	S100A4
tetracaine-2888	1/100	0.561955	0.561955	0.637173	0.637173	2.469955	-1.42352	ITM2C
sulfamethoxypyridazine-3609	1/100	0.561955	0.561955	0.637173	0.637173	2.470403	-1.42378	SECTM1
tetraethylenepentamine-412	1/100	0.561955	0.561955	0.637173	0.637173	2.471426	-1.42437	IFITM3
tiapride-2292	1/100	0.561955	0.561955	0.637173	0.637173	2.47239	-1.42492	PIGR
thioproperazine-6265	1/100	0.561955	0.561955	0.637173	0.637173	2.472745	-1.42513	DCN
thioguanosine-4989	1/100	0.561955	0.561955	0.637173	0.637173	2.473704	-1.42568	TXNIP
tanespimycin-505	1/100	0.561955	0.561955	0.637173	0.637173	2.474143	-1.42593	HSPB1
tiratricol-1412	1/100	0.561955	0.561955	0.637173	0.637173	2.475239	-1.42656	TSPAN13
tanespimycin-947	1/100	0.561955	0.561955	0.637173	0.637173	2.475848	-1.42691	BMP4
tanespimycin-5919	1/100	0.561955	0.561955	0.637173	0.637173	2.479491	-1.42901	HSPB1
tanespimycin-450	1/100	0.561955	0.561955	0.637173	0.637173	2.479665	-1.42911	HSPB1
tanespimycin-6177	1/100	0.561955	0.561955	0.637173	0.637173	2.479859	-1.42923	HSPB1
tracazolate-4964	1/100	0.561955	0.561955	0.637173	0.637173	2.480285	-1.42947	CEACAM5
tanespimycin-5203	1/100	0.561955	0.561955	0.637173	0.637173	2.481966	-1.43044	SOX9
tetraethylenepentamine-457	1/100	0.561955	0.561955	0.637173	0.637173	2.481966	-1.43044	CDH3
thioridazine-422	1/100	0.561955	0.561955	0.637173	0.637173	2.482385	-1.43068	ENC1
tanespimycin-2686	1/100	0.561955	0.561955	0.637173	0.637173	2.482602	-1.43081	HSPB1
talampicillin-7014	1/100	0.561955	0.561955	0.637173	0.637173	2.483762	-1.43148	GPX2
tanespimycin-1005	1/100	0.561955	0.561955	0.637173	0.637173	2.483879	-1.43154	BMP4
tanespimycin-1044	1/100	0.561955	0.561955	0.637173	0.637173	2.48412	-1.43168	SOX9
terazosin-7187	1/100	0.561955	0.561955	0.637173	0.637173	2.485637	-1.43256	CEACAM6
tanespimycin-5566	1/100	0.561955	0.561955	0.637173	0.637173	2.48621	-1.43289	COL1A1
testosterone-4676	1/100	0.561955	0.561955	0.637173	0.637173	2.486448	-1.43302	MMP3
thioridazine-1905	1/100	0.561955	0.561955	0.637173	0.637173	2.487163	-1.43344	ETS2
tiapride-3663	1/100	0.561955	0.561955	0.637173	0.637173	2.487678	-1.43373	DCN
tanespimycin-5953	1/100	0.561955	0.561955	0.637173	0.637173	2.489043	-1.43452	TXNIP
suramin sodium-7524	1/100	0.561955	0.561955	0.637173	0.637173	2.4901	-1.43513	SECTM1

terguride-6473	1/100	0.561955	0.561955	0.637173	0.637173	2.490415	-1.43531	BMP4
tetraethylenepentamine-498	1/100	0.561955	0.561955	0.637173	0.637173	2.491819	-1.43612	DPEP1
theobromine-6613	1/100	0.561955	0.561955	0.637173	0.637173	2.493021	-1.43681	MMP7
thiocolchicoside-2875	1/100	0.561955	0.561955	0.637173	0.637173	2.493461	-1.43707	IFI6
thioguanosine-2619	1/100	0.561955	0.561955	0.637173	0.637173	2.494935	-1.43791	TXNIP
tetroquinone-4078	1/100	0.561955	0.561955	0.637173	0.637173	2.495022	-1.43797	SERPINA1
terguride-6299	1/100	0.561955	0.561955	0.637173	0.637173	2.495546	-1.43827	AP1S1
tanespimycin-5215	1/100	0.561955	0.561955	0.637173	0.637173	2.496352	-1.43873	BMP4
tanespimycin-6966	1/100	0.561955	0.561955	0.637173	0.637173	2.496502	-1.43882	BMP4
tanespimycin-1063	1/100	0.561955	0.561955	0.637173	0.637173	2.496946	-1.43907	BMP4
tinidazole-3430	1/100	0.561955	0.561955	0.637173	0.637173	2.500456	-1.4411	S100A6
tocainide-7351	1/100	0.561955	0.561955	0.637173	0.637173	2.501454	-1.44167	SECTM1
thiocolchicoside-5095	1/100	0.561955	0.561955	0.637173	0.637173	2.502023	-1.442	BMP4
thiamine-7479	1/100	0.561955	0.561955	0.637173	0.637173	2.502861	-1.44248	ENC1
terguride-6459	1/100	0.561955	0.561955	0.637173	0.637173	2.504151	-1.44323	COL1A1
thapsigargin-7100	1/100	0.561955	0.561955	0.637173	0.637173	2.504605	-1.44349	TXNIP
tanespimycin-6943	1/100	0.561955	0.561955	0.637173	0.637173	2.50611	-1.44436	S100A11
thioproperazine-5791	1/100	0.561955	0.561955	0.637173	0.637173	2.506301	-1.44447	JUND
sulpiride-1967	1/100	0.561955	0.561955	0.637173	0.637173	2.50656	-1.44461	CD24
tolazamide-2482	1/100	0.561955	0.561955	0.637173	0.637173	2.507092	-1.44492	APP
tinidazole-3813	1/100	0.561955	0.561955	0.637173	0.637173	2.508545	-1.44576	JUND
testosterone-2649	1/100	0.561955	0.561955	0.637173	0.637173	2.510907	-1.44712	MMP3
terbutaline-1585	1/100	0.561955	0.561955	0.637173	0.637173	2.511303	-1.44735	CCL20
tolfenamic acid-2120	1/100	0.561955	0.561955	0.637173	0.637173	2.511324	-1.44736	BMP4
ticlopidine-4155	1/100	0.561955	0.561955	0.637173	0.637173	2.512542	-1.44806	FOS
tanespimycin-6937	1/100	0.561955	0.561955	0.637173	0.637173	2.513086	-1.44838	SOX9
terbutaline-3202	1/100	0.561955	0.561955	0.637173	0.637173	2.513863	-1.44882	DCN
thiocolchicoside-1726	1/100	0.561955	0.561955	0.637173	0.637173	2.514142	-1.44898	QPCT
tiletamine-6013	1/100	0.561955	0.561955	0.637173	0.637173	2.515	-1.44948	SPARC
thiamazole-2570	1/100	0.561955	0.561955	0.637173	0.637173	2.520784	-1.45281	CCL20
tocainide-4838	1/100	0.561955	0.561955	0.637173	0.637173	2.522445	-1.45377	IFI6
tolazamide-2842	1/100	0.561955	0.561955	0.637173	0.637173	2.522569	-1.45384	IFI6
tinidazole-2568	1/100	0.561955	0.561955	0.637173	0.637173	2.523331	-1.45428	FABP1
triamcinolone-2078	1/100	0.561955	0.561955	0.637173	0.637173	2.523715	-1.4545	CCL20
trichostatin A-4388	1/100	0.561955	0.561955	0.637173	0.637173	2.525998	-1.45582	ITM2C
tomatidine-2746	1/100	0.561955	0.561955	0.637173	0.637173	2.526145	-1.4559	PIGR
thiamphenicol-1867	1/100	0.561955	0.561955	0.637173	0.637173	2.526455	-1.45608	PTPRO

thioguanosine-6643	1/100	0.561955	0.561955	0.637173	0.637173	2.527241	-1.45653	FOS
thiamphenicol-7274	1/100	0.561955	0.561955	0.637173	0.637173	2.527321	-1.45658	IFI6
trichostatin A-332	1/100	0.561955	0.561955	0.637173	0.637173	2.531996	-1.45927	IFITM1
tolbutamide-4540	1/100	0.561955	0.561955	0.637173	0.637173	2.532287	-1.45944	SPARC
tomelukast-222	1/100	0.561955	0.561955	0.637173	0.637173	2.53253	-1.45958	PHLDA1
tomatidine-1754	1/100	0.561955	0.561955	0.637173	0.637173	2.533639	-1.46022	TCN1
tiabendazole-2479	1/100	0.561955	0.561955	0.637173	0.637173	2.534078	-1.46047	GPX2
todralazine-1799	1/100	0.561955	0.561955	0.637173	0.637173	2.534298	-1.4606	APP
tiratricol-2259	1/100	0.561955	0.561955	0.637173	0.637173	2.53946	-1.46358	C3
tolmetin-4088	1/100	0.561955	0.561955	0.637173	0.637173	2.540098	-1.46394	RNF43
tiapride-4686	1/100	0.561955	0.561955	0.637173	0.637173	2.541256	-1.46461	ITM2B
ticarcillin-5829	1/100	0.561955	0.561955	0.637173	0.637173	2.541623	-1.46482	IFI6
tetramisole-4412	1/100	0.561955	0.561955	0.637173	0.637173	2.541968	-1.46502	SECTM1
trichostatin A-2450	1/100	0.561955	0.561955	0.637173	0.637173	2.544091	-1.46624	ATP1B1
tobramycin-2841	1/100	0.561955	0.561955	0.637173	0.637173	2.546071	-1.46739	COL1A1
torasemide-2956	1/100	0.561955	0.561955	0.637173	0.637173	2.548463	-1.46876	ATP1B1
torasemide-5057	1/100	0.561955	0.561955	0.637173	0.637173	2.548815	-1.46897	COL1A1
trichostatin A-1637	1/100	0.561955	0.561955	0.637173	0.637173	2.548924	-1.46903	TXNIP
tranexamic acid-6238	1/100	0.561955	0.561955	0.637173	0.637173	2.549393	-1.4693	BMP4
tonzonium bromide-3080	1/100	0.561955	0.561955	0.637173	0.637173	2.550006	-1.46965	IFITM1
tolfenamic acid-6354	1/100	0.561955	0.561955	0.637173	0.637173	2.553377	-1.4716	GPX2
tranexamic acid-5762	1/100	0.561955	0.561955	0.637173	0.637173	2.554372	-1.47217	TGFBI
timolol-5645	1/100	0.561955	0.561955	0.637173	0.637173	2.5571	-1.47374	CEACAM5
trichostatin A-5441	1/100	0.561955	0.561955	0.637173	0.637173	2.558667	-1.47465	ITM2C
trichostatin A-4526	1/100	0.561955	0.561955	0.637173	0.637173	2.560237	-1.47555	NQO1
trichostatin A-5017	1/100	0.561955	0.561955	0.637173	0.637173	2.561737	-1.47641	SOX9
triamcinolone-2241	1/100	0.561955	0.561955	0.637173	0.637173	2.561785	-1.47644	COL1A1
tonzonium bromide-5678	1/100	0.561955	0.561955	0.637173	0.637173	2.561919	-1.47652	MMP3
tranylcypromine-2264	1/100	0.561955	0.561955	0.637173	0.637173	2.562483	-1.47684	CEACAM5
trichostatin A-1014	1/100	0.561955	0.561955	0.637173	0.637173	2.564083	-1.47777	TXNIP
tracazolate-2919	1/100	0.561955	0.561955	0.637173	0.637173	2.566119	-1.47894	COL1A1
tremorine-6273	1/100	0.561955	0.561955	0.637173	0.637173	2.567805	-1.47991	ETS2
trichostatin A-3510	1/100	0.561955	0.561955	0.637173	0.637173	2.568665	-1.48041	TXNIP
trichostatin A-1212	1/100	0.561955	0.561955	0.637173	0.637173	2.568786	-1.48048	CTGF
trichostatin A-7324	1/100	0.561955	0.561955	0.637173	0.637173	2.56972	-1.48102	TXNIP
trichostatin A-2639	1/100	0.561955	0.561955	0.637173	0.637173	2.570635	-1.48154	TXNIP
tolfenamic acid-1437	1/100	0.561955	0.561955	0.637173	0.637173	2.572095	-1.48238	FOS

tranexamic acid-2085	1/100	0.561955	0.561955	0.637173	0.637173	2.572913	-1.48286	PSMB9
trichostatin A-4768	1/100	0.561955	0.561955	0.637173	0.637173	2.575061	-1.48409	ITM2C
triamcinolone-7192	1/100	0.561955	0.561955	0.637173	0.637173	2.578042	-1.48581	CEACAM6
trichostatin A-1785	1/100	0.561955	0.561955	0.637173	0.637173	2.581099	-1.48757	TCN1
trichostatin A-4436	1/100	0.561955	0.561955	0.637173	0.637173	2.582811	-1.48856	CTGF
tolmetin-3009	1/100	0.561955	0.561955	0.637173	0.637173	2.583477	-1.48894	FXYD3
thioridazine-6189	1/100	0.561955	0.561955	0.637173	0.637173	2.583968	-1.48923	GPX2
tribenoside-5429	1/100	0.561955	0.561955	0.637173	0.637173	2.584704	-1.48965	PSMB9
trichostatin A-6493	1/100	0.561955	0.561955	0.637173	0.637173	2.585027	-1.48984	TXNIP
trichostatin A-3566	1/100	0.561955	0.561955	0.637173	0.637173	2.585732	-1.49024	IFI27
tolazamide-4003	1/100	0.561955	0.561955	0.637173	0.637173	2.58583	-1.4903	IL32
trichostatin A-5882	1/100	0.561955	0.561955	0.637173	0.637173	2.587976	-1.49154	CTGF
trichostatin A-4344	1/100	0.561955	0.561955	0.637173	0.637173	2.588533	-1.49186	CTGF
trichostatin A-2672	1/100	0.561955	0.561955	0.637173	0.637173	2.588544	-1.49186	ATP1B1
tridihexethyl-5067	1/100	0.561955	0.561955	0.637173	0.637173	2.589217	-1.49225	PSMB9
trichostatin A-5802	1/100	0.561955	0.561955	0.637173	0.637173	2.590417	-1.49294	CTGF
tranylcypromine-5996	1/100	0.561955	0.561955	0.637173	0.637173	2.590577	-1.49304	QPCT
tremorine-3196	1/100	0.561955	0.561955	0.637173	0.637173	2.590892	-1.49322	DCN
trichostatin A-7179	1/100	0.561955	0.561955	0.637173	0.637173	2.591569	-1.49361	TXNIP
tolazoline-2000	1/100	0.561955	0.561955	0.637173	0.637173	2.591694	-1.49368	ITM2B
trichostatin A-1659	1/100	0.561955	0.561955	0.637173	0.637173	2.59363	-1.4948	TXNIP
trichostatin A-5308	1/100	0.561955	0.561955	0.637173	0.637173	2.596992	-1.49673	TXNIP
trichostatin A-1672	1/100	0.561955	0.561955	0.637173	0.637173	2.598978	-1.49788	TXNIP
tremorine-1579	1/100	0.561955	0.561955	0.637173	0.637173	2.599264	-1.49804	ENC1
trichostatin A-4770	1/100	0.561955	0.561955	0.637173	0.637173	2.600164	-1.49856	PSMB9
trazodone-7452	1/100	0.561955	0.561955	0.637173	0.637173	2.602061	-1.49966	TCN1
trichostatin A-3428	1/100	0.561955	0.561955	0.637173	0.637173	2.604811	-1.50124	TXNIP
trichostatin A-448	1/100	0.561955	0.561955	0.637173	0.637173	2.604847	-1.50126	CTGF
tridihexethyl-3526	1/100	0.561955	0.561955	0.637173	0.637173	2.605132	-1.50143	AP1S1
trichostatin A-3332	1/100	0.561955	0.561955	0.637173	0.637173	2.605516	-1.50165	IFI6
triflupromazine-1855	1/100	0.561955	0.561955	0.637173	0.637173	2.606738	-1.50235	PFN1
trifluoperazine-1224	1/100	0.561955	0.561955	0.637173	0.637173	2.60801	-1.50308	SPINK1
trichlormethiazide-4783	1/100	0.561955	0.561955	0.637173	0.637173	2.608177	-1.50318	IFI6
trichostatin A-4954	1/100	0.561955	0.561955	0.637173	0.637173	2.609513	-1.50395	CTGF
trichostatin A-6579	1/100	0.561955	0.561955	0.637173	0.637173	2.611084	-1.50486	TXNIP
trichostatin A-6972	1/100	0.561955	0.561955	0.637173	0.637173	2.611361	-1.50501	TXNIP
trichlormethiazide-3337	1/100	0.561955	0.561955	0.637173	0.637173	2.612623	-1.50574	SECTM1

trichostatin A-6276	1/100	0.561955	0.561955	0.637173	0.637173	2.6147	-1.50694	TXNIP
trichostatin A-5693	1/100	0.561955	0.561955	0.637173	0.637173	2.615162	-1.50721	PSMB9
troleandomycin-1965	1/100	0.561955	0.561955	0.637173	0.637173	2.617283	-1.50843	TPI1
trimethylcolchicinic acid-4202	1/100	0.561955	0.561955	0.637173	0.637173	2.617908	-1.50879	MMP3
trichostatin A-6671	1/100	0.561955	0.561955	0.637173	0.637173	2.618715	-1.50925	BMP4
trifluridine-6136	1/100	0.561955	0.561955	0.637173	0.637173	2.619435	-1.50967	TGFBI
trifluoperazine-2389	1/100	0.561955	0.561955	0.637173	0.637173	2.621815	-1.51104	CEACAM5
urapidil-6696	1/100	0.561955	0.561955	0.637173	0.637173	2.622477	-1.51142	QPCT
trichostatin A-7499	1/100	0.561955	0.561955	0.637173	0.637173	2.623599	-1.51207	TXNIP
trichostatin A-1175	1/100	0.561955	0.561955	0.637173	0.637173	2.625207	-1.51299	TSPAN13
trichostatin A-5745	1/100	0.561955	0.561955	0.637173	0.637173	2.630458	-1.51602	CTGF
tropicamide-4280	1/100	0.561955	0.561955	0.637173	0.637173	2.631752	-1.51677	PIGR
trichostatin A-6193	1/100	0.561955	0.561955	0.637173	0.637173	2.634049	-1.51809	TSPAN13
ursodeoxycholic acid-3105	1/100	0.561955	0.561955	0.637173	0.637173	2.634229	-1.51819	CD24
tolbutamide-2359	1/100	0.561955	0.561955	0.637173	0.637173	2.634295	-1.51823	DCN
tropicamide-4744	1/100	0.561955	0.561955	0.637173	0.637173	2.634334	-1.51825	COL1A1
trichostatin A-7043	1/100	0.561955	0.561955	0.637173	0.637173	2.636371	-1.51943	TXNIP
tomatidine-7166	1/100	0.561955	0.561955	0.637173	0.637173	2.636427	-1.51946	ITM2B
troglitazone-1173	1/100	0.561955	0.561955	0.637173	0.637173	2.637151	-1.51988	CTSH
trichostatin A-6993	1/100	0.561955	0.561955	0.637173	0.637173	2.637313	-1.51997	TXNIP
tranexamic acid-1401	1/100	0.561955	0.561955	0.637173	0.637173	2.639628	-1.52131	ITM2B
troglitazone-4456	1/100	0.561955	0.561955	0.637173	0.637173	2.639747	-1.52137	FOS
tyloxapol-4611	1/100	0.561955	0.561955	0.637173	0.637173	2.641459	-1.52236	COL1A1
trichostatin A-992	1/100	0.561955	0.561955	0.637173	0.637173	2.642723	-1.52309	TXNIP
troglitazone-1232	1/100	0.561955	0.561955	0.637173	0.637173	2.644968	-1.52438	C3
tropicamide-2347	1/100	0.561955	0.561955	0.637173	0.637173	2.646871	-1.52548	TSPAN8
trimethobenzamide-2002	1/100	0.561955	0.561955	0.637173	0.637173	2.647099	-1.52561	ITM2B
trichostatin A-1050	1/100	0.561955	0.561955	0.637173	0.637173	2.647516	-1.52585	TXNIP
trioxysalen-5736	1/100	0.561955	0.561955	0.637173	0.637173	2.647905	-1.52608	TFF3
troglitazone-431	1/100	0.561955	0.561955	0.637173	0.637173	2.648688	-1.52653	DPEP1
trichostatin A-7136	1/100	0.561955	0.561955	0.637173	0.637173	2.648854	-1.52662	CTGF
tyrphostin AG-1478-1141	1/100	0.561955	0.561955	0.637173	0.637173	2.64985	-1.5272	ETS2
trichostatin A-7245	1/100	0.561955	0.561955	0.637173	0.637173	2.650379	-1.5275	CTGF
trimethylcolchicinic acid-4787	1/100	0.561955	0.561955	0.637173	0.637173	2.651498	-1.52815	COL1A1
trichostatin A-3643	1/100	0.561955	0.561955	0.637173	0.637173	2.651798	-1.52832	IFI6
valproic acid-4438	1/100	0.561955	0.561955	0.637173	0.637173	2.653195	-1.52913	JUND
trichostatin A-2721	1/100	0.561955	0.561955	0.637173	0.637173	2.653501	-1.5293	IFI6

trimethadione-2486	1/100	0.561955	0.561955	0.637173	0.637173	2.654757	-1.53003	PIGR
valproic acid-4464	1/100	0.561955	0.561955	0.637173	0.637173	2.657108	-1.53138	COL1A1
triflupromazine-1813	1/100	0.561955	0.561955	0.637173	0.637173	2.65998	-1.53304	AP1S1
trichostatin A-7550	1/100	0.561955	0.561955	0.637173	0.637173	2.661147	-1.53371	TXNIP
trichostatin A-7407	1/100	0.561955	0.561955	0.637173	0.637173	2.661753	-1.53406	TXNIP
valproic acid-5219	1/100	0.561955	0.561955	0.637173	0.637173	2.662152	-1.53429	ITM2C
urapidil-3078	1/100	0.561955	0.561955	0.637173	0.637173	2.662712	-1.53461	COL1A1
valproic acid-4433	1/100	0.561955	0.561955	0.637173	0.637173	2.663958	-1.53533	COL1A2
valproic acid-347	1/100	0.561955	0.561955	0.637173	0.637173	2.664118	-1.53542	PIGR
urapidil-5295	1/100	0.561955	0.561955	0.637173	0.637173	2.665461	-1.53619	S100A4
troglitazone-370	1/100	0.561955	0.561955	0.637173	0.637173	2.666415	-1.53674	ENC1
trichlormethiazide-4198	1/100	0.561955	0.561955	0.637173	0.637173	2.668616	-1.53801	MMP3
valproic acid-5569	1/100	0.561955	0.561955	0.637173	0.637173	2.669053	-1.53826	ITM2C
triflusal-2867	1/100	0.561955	0.561955	0.637173	0.637173	2.671624	-1.53975	COL1A2
troglitazone-1657	1/100	0.561955	0.561955	0.637173	0.637173	2.672416	-1.5402	AP1S1
Trolox C-6007	1/100	0.561955	0.561955	0.637173	0.637173	2.673669	-1.54092	CEACAM6
trichostatin A-3177	1/100	0.561955	0.561955	0.637173	0.637173	2.676964	-1.54282	TXNIP
tubocurarine chloride-2887	1/100	0.561955	0.561955	0.637173	0.637173	2.677314	-1.54303	MMP3
trimethobenzamide-1502	1/100	0.561955	0.561955	0.637173	0.637173	2.677516	-1.54314	COL1A1
trifluoperazine-6341	1/100	0.561955	0.561955	0.637173	0.637173	2.677783	-1.5433	S100P
velnacrine-3292	1/100	0.561955	0.561955	0.637173	0.637173	2.67807	-1.54346	S100A11
trimethobenzamide-1920	1/100	0.561955	0.561955	0.637173	0.637173	2.678463	-1.54369	PHLDA1
vinblastine-7517	1/100	0.561955	0.561955	0.637173	0.637173	2.681443	-1.54541	C3
valproic acid-409	1/100	0.561955	0.561955	0.637173	0.637173	2.685965	-1.54801	TSPAN13
trichostatin A-5976	1/100	0.561955	0.561955	0.637173	0.637173	2.686327	-1.54822	TXNIP
wortmannin-2703	1/100	0.561955	0.561955	0.637173	0.637173	2.686616	-1.54839	PHLDA1
ursodeoxycholic acid-6484	1/100	0.561955	0.561955	0.637173	0.637173	2.689115	-1.54983	PHLDA1
valproic acid-458	1/100	0.561955	0.561955	0.637173	0.637173	2.689781	-1.55021	CDH3
valproic acid-345	1/100	0.561955	0.561955	0.637173	0.637173	2.692351	-1.55169	TXNIP
Trolox C-1734	1/100	0.561955	0.561955	0.637173	0.637173	2.693071	-1.55211	CKB
tropine-6264	1/100	0.561955	0.561955	0.637173	0.637173	2.693706	-1.55247	BMP4
trichostatin A-7285	1/100	0.561955	0.561955	0.637173	0.637173	2.694075	-1.55269	CTGF
tropine-6147	1/100	0.561955	0.561955	0.637173	0.637173	2.69476	-1.55308	PIGR
troglitazone-462	1/100	0.561955	0.561955	0.637173	0.637173	2.696155	-1.55388	PIGR
trimipramine-4083	1/100	0.561955	0.561955	0.637173	0.637173	2.698706	-1.55535	CEACAM5
valproic acid-1047	1/100	0.561955	0.561955	0.637173	0.637173	2.698787	-1.5554	PGM1
valproic acid-994	1/100	0.561955	0.561955	0.637173	0.637173	2.698833	-1.55543	APP

vincamine-3865	1/100	0.561955	0.561955	0.637173	0.637173	2.701493	-1.55696	COL1A1
valproic acid-2700	1/100	0.561955	0.561955	0.637173	0.637173	2.702037	-1.55727	COL1A2
zuclopenthixol-4261	1/100	0.561955	0.561955	0.637173	0.637173	2.70238	-1.55747	CEACAM5
trimetazidine-5060	1/100	0.561955	0.561955	0.637173	0.637173	2.702963	-1.55781	COL1A1
wortmannin-1668	1/100	0.561955	0.561955	0.637173	0.637173	2.704901	-1.55893	TXNIP
vitexin-4413	1/100	0.561955	0.561955	0.637173	0.637173	2.707927	-1.56067	ITM2C
valproic acid-6941	1/100	0.561955	0.561955	0.637173	0.637173	2.708352	-1.56091	S100A4
valproic acid-348	1/100	0.561955	0.561955	0.637173	0.637173	2.708354	-1.56092	PIGR
valproic acid-4446	1/100	0.561955	0.561955	0.637173	0.637173	2.708882	-1.56122	MMP7
valproic acid-410	1/100	0.561955	0.561955	0.637173	0.637173	2.710645	-1.56224	TSPAN13
verapamil-1509	1/100	0.561955	0.561955	0.637173	0.637173	2.713417	-1.56383	TCN1
ursolic acid-2067	1/100	0.561955	0.561955	0.637173	0.637173	2.714157	-1.56426	BMP4
valproic acid-629	1/100	0.561955	0.561955	0.637173	0.637173	2.714848	-1.56466	ATP1B1
verapamil-2009	1/100	0.561955	0.561955	0.637173	0.637173	2.71676	-1.56576	APP
W-13-643	1/100	0.561955	0.561955	0.637173	0.637173	2.717283	-1.56606	TCN1
tropine-3569	1/100	0.561955	0.561955	0.637173	0.637173	2.718611	-1.56683	S100A4
zimeldine-1930	1/100	0.561955	0.561955	0.637173	0.637173	2.719945	-1.5676	APP
vinpocetine-3174	1/100	0.561955	0.561955	0.637173	0.637173	2.720878	-1.56813	MMP3
xylazine-2788	1/100	0.561955	0.561955	0.637173	0.637173	2.72196	-1.56876	MMP3
vitexin-4588	1/100	0.561955	0.561955	0.637173	0.637173	2.724798	-1.57039	COL1A1
vinpocetine-7213	1/100	0.561955	0.561955	0.637173	0.637173	2.725355	-1.57071	SERPINA1
vinburnine-1788	1/100	0.561955	0.561955	0.637173	0.637173	2.725815	-1.57098	SLPI
vigabatrin-2452	1/100	0.561955	0.561955	0.637173	0.637173	2.726862	-1.57158	ISG15
vorinostat-1000	1/100	0.561955	0.561955	0.637173	0.637173	2.727825	-1.57214	TXNIP
valproic acid-433	1/100	0.561955	0.561955	0.637173	0.637173	2.728866	-1.57274	CEACAM5
valproic acid-497	1/100	0.561955	0.561955	0.637173	0.637173	2.732099	-1.5746	DPEP1
vanoxerine-1625	1/100	0.561955	0.561955	0.637173	0.637173	2.735668	-1.57666	CKB
zidovudine-1595	1/100	0.561955	0.561955	0.637173	0.637173	2.735975	-1.57683	CCL20
vanoxerine-5702	1/100	0.561955	0.561955	0.637173	0.637173	2.736552	-1.57717	FOS
vorinostat-6179	1/100	0.561955	0.561955	0.637173	0.637173	2.740922	-1.57969	TSPAN13
vorinostat-6980	1/100	0.561955	0.561955	0.637173	0.637173	2.741314	-1.57991	TXNIP
verapamil-1927	1/100	0.561955	0.561955	0.637173	0.637173	2.741524	-1.58003	ITM2B
valinomycin-5911	1/100	0.561955	0.561955	0.637173	0.637173	2.74182	-1.5802	S100P
wortmannin-1023	1/100	0.561955	0.561955	0.637173	0.637173	2.745222	-1.58216	RNF43
wortmannin-389	1/100	0.561955	0.561955	0.637173	0.637173	2.745892	-1.58255	TCN1
yohimbine-6777	1/100	0.561955	0.561955	0.637173	0.637173	2.750276	-1.58508	C3
valproic acid-6934	1/100	0.561955	0.561955	0.637173	0.637173	2.750536	-1.58523	PGM1

vincamine-2367	1/100	0.561955	0.561955	0.637173	0.637173	2.754687	-1.58762	CKB
zomepirac-3454	1/100	0.561955	0.561955	0.637173	0.637173	2.755304	-1.58797	SERPINA1
yohimbic acid-4082	1/100	0.561955	0.561955	0.637173	0.637173	2.7556	-1.58814	COL1A1
vidarabine-3445	1/100	0.561955	0.561955	0.637173	0.637173	2.756962	-1.58893	COL1A1
zimeldine-4609	1/100	0.561955	0.561955	0.637173	0.637173	2.762787	-1.59229	FABP1
vancomycin-4598	1/100	0.561955	0.561955	0.637173	0.637173	2.770899	-1.59696	SERPINA1
vincamine-3784	1/100	0.561955	0.561955	0.637173	0.637173	2.774843	-1.59923	IL32
zidovudine-3211	1/100	0.561955	0.561955	0.637173	0.637173	2.78847	-1.60709	C3
zoxazolamine-5390	1/100	0.561955	0.561955	0.637173	0.637173	2.78922	-1.60752	COL1A1
valproic acid-6199	1/100	0.561955	0.561955	0.637173	0.637173	2.794192	-1.61039	TSPAN13
valproic acid-6168	1/100	0.561955	0.561955	0.637173	0.637173	2.811883	-1.62058	TFF2

CMap co-downregulated genes

Term	Overlap	P-value	Adjusted P-value	Old P-value	Old Adjusted P-value	Z-score	Combined Score	Genes
omeprazole-4951	7/100	1.85E-05	0.068619	0.000387	0.724338	-1.69591	18.4802	IFITM2;IFI27;IFI6;S100A4;ISG15;PLP2;TSPAN1
apramycin-4959	6/100	0.000175	0.157541	0.002152	0.724338	-1.67175	14.4611	IFITM1;IFI27;QPCT;IFI6;S100A4;ISG15
ambroxol-6719	6/100	0.000175	0.157541	0.002152	0.724338	-1.63342	14.12953	SLPI;ID1;S100A4;PFN1;TSPAN1;LY6E
verteporfin-3556	6/100	0.000175	0.157541	0.002152	0.724338	-1.58507	13.71131	PDIA3;SLC12A2;CDH3;ITGAV;DSG2;ATP1A1
crotamiton-3388	5/100	0.001403	0.157541	0.010351	0.724338	-1.68334	11.05833	RNF43;GPX2;SLPI;AP1S1;TSPAN1
chlormezanone-4636	5/100	0.001403	0.157541	0.010351	0.724338	-1.67843	11.02605	IL32;BMP4;SPINK1;OLFM4;DUOX2
cyclopenthiazide-7325	5/100	0.001403	0.157541	0.010351	0.724338	-1.67083	10.97612	LGALS3BP;BMP4;IFI6;S100A4;ISG15
ethisterone-3975	5/100	0.001403	0.157541	0.010351	0.724338	-1.6679	10.95687	BMP4;SLPI;IFI6;AP1S1;TFF2
SC-58125-542	5/100	0.001403	0.157541	0.010351	0.724338	-1.66701	10.95103	IFITM3;C3;GPX2;NDUFA4;CTSE
dl-alpha tocopherol-4961	5/100	0.001403	0.157541	0.010351	0.724338	-1.65677	10.88378	GPX2;IFI27;IFI6;ISG15;TSPAN1
sirolimus-5239	5/100	0.001403	0.157541	0.010351	0.724338	-1.65297	10.85884	BMP4;RNF43;MMP3;SECTM1;SOX9
16,16-dimethylprostaglandin E2-6562	5/100	0.001403	0.157541	0.010351	0.724338	-1.64474	10.80471	LGALS3BP;GPX2;MMP1;PLP2;CTGF
nitrofural-6721	5/100	0.001403	0.157541	0.010351	0.724338	-1.6445	10.80318	TCN1;ID1;PFN1;TSPAN1;LY6E
acetazolamide-1850	5/100	0.001403	0.157541	0.010351	0.724338	-1.64363	10.79742	C3;SLPI;QPCT;CTSD;LY6E
carisoprodol-4955	5/100	0.001403	0.157541	0.010351	0.724338	-1.64346	10.79634	IFITM1;IFI27;IFI6;S100A4;ISG15
pioglitazone-6893	5/100	0.001403	0.157541	0.010351	0.724338	-1.6414	10.78279	BMP4;C3;AP1S1;CXCL1;CTGF
monensin-7402	5/100	0.001403	0.157541	0.010351	0.724338	-1.63899	10.76696	ID1;IFI6;ID3;SECTM1;CXCL1
moxonidine-2923	5/100	0.001403	0.157541	0.010351	0.724338	-1.63754	10.75745	TSPAN13;TCN1;IFI6;CTSD;LY6E
naringin-5666	5/100	0.001403	0.157541	0.010351	0.724338	-1.63663	10.75146	LGALS3BP;IFI27;QPCT;IFI6;S100A4

lansoprazole-7306	5/100	0.001403	0.157541	0.010351	0.724338	-1.63602	10.74748	LGALS3BP;IGFBP2;IFI6;TSPAN1;CTSD
nefopam-4752	5/100	0.001403	0.157541	0.010351	0.724338	-1.6349	10.74009	ID1;IFI6;ID3;AP1S1;TSPAN1
amikacin-6715	5/100	0.001403	0.157541	0.010351	0.724338	-1.63409	10.73477	SLPI;ID1;IFI6;TSPAN1;LY6E
benfluorex-5327	5/100	0.001403	0.157541	0.010351	0.724338	-1.63147	10.71758	ID1;S100A6;MIF;TSPAN1;CTSD
mebeverine-6795	5/100	0.001403	0.157541	0.010351	0.724338	-1.62916	10.7024	LGALS3BP;RCN1;GPX2;IFI6;LY6E
tanespimycin-1218	5/100	0.001403	0.157541	0.010351	0.724338	-1.62864	10.69898	IL32;BMP4;IFI6;IER2;LY6E
karakoline-3638	5/100	0.001403	0.157541	0.010351	0.724338	-1.62368	10.66639	C3;GPX2;SPINK1;SLPI;ITM2C
myricetin-4170	5/100	0.001403	0.157541	0.010351	0.724338	-1.60736	10.55918	SLPI;TCN1;MMP3;IFI6;AP1S1
midecamycin-2026	5/100	0.001403	0.157541	0.010351	0.724338	-1.60705	10.55714	GPI;CBX3;PARK7;PABPC1;ZWINT
CP-863187-7512	5/100	0.001403	0.157541	0.010351	0.724338	-1.60559	10.54755	SLCO1B3;IFI6;ID3;TSPAN1;CTGF
estradiol-2653	5/100	0.001403	0.157541	0.010351	0.724338	-1.60081	10.51613	BMP4;RCN1;ID3;SECTM1;IER2
thioperamide-4675	5/100	0.001403	0.157541	0.010351	0.724338	-1.597	10.49111	IL32;SPINK1;IFI6;TGFBI;OLFM4
sulfathiazole-4769	5/100	0.001403	0.157541	0.010351	0.724338	-1.59627	10.48631	IGFBP2;ID1;S100A6;IFI6;MIF
solanine-4166	5/100	0.001403	0.157541	0.010351	0.724338	-1.58247	10.3957	SLPI;TCN1;IFI6;AP1S1;TFF2
carbamazepine-919	4/100	0.009292	0.260892	0.042102	0.724338	-1.66275	7.779272	BMP4;RNF43;TMPRSS3;IER2
ciclopirox-6677	4/100	0.009292	0.260892	0.042102	0.724338	-1.65119	7.725196	MMP3;CXCL1;TGFBI;HIF1A
beclometasone-4403	4/100	0.009292	0.260892	0.042102	0.724338	-1.64417	7.692335	SLPI;IFI27;IFI6;AP1S1
decamethonium bromide-7353	4/100	0.009292	0.260892	0.042102	0.724338	-1.64234	7.683773	LGALS3BP;IFI6;S100A4;ISG15
ethoxyquin-3846	4/100	0.009292	0.260892	0.042102	0.724338	-1.63708	7.659163	SLPI;MMP3;IFI6;TFF2
clofilium tosylate-6830	4/100	0.009292	0.260892	0.042102	0.724338	-1.6357	7.652699	IFITM1;GPX2;IFI27;IFI6
alprenolol-6789	4/100	0.009292	0.260892	0.042102	0.724338	-1.62626	7.608534	RCN1;GPX2;S100A6;IFI6
butyl hydroxybenzoate-5245	4/100	0.009292	0.260892	0.042102	0.724338	-1.62522	7.60367	PDIA3;SLPI;IFI27;ID3
metampicillin-2286	4/100	0.009292	0.260892	0.042102	0.724338	-1.62437	7.599709	C3;SLPI;MMP3;SECTM1
11-deoxy-16,16-dimethylprostaglandin E2-7519	4/100	0.009292	0.260892	0.042102	0.724338	-1.62247	7.590815	IFITM1;CDH3;AP1S1;CTGF
adiphenine-1831	4/100	0.009292	0.260892	0.042102	0.724338	-1.62208	7.588991	TUBA1C;GPX2;IFI27;IFI6
biotin-3289	4/100	0.009292	0.260892	0.042102	0.724338	-1.62079	7.582955	RPS14P3;RNF43;SECTM1;S100A4
15-delta prostaglandin J2-4455	4/100	0.009292	0.260892	0.042102	0.724338	-1.61646	7.562696	RNF43;IFI6;CXCL1;PSMB9
iobenguane-6002	4/100	0.009292	0.260892	0.042102	0.724338	-1.61499	7.555834	LGALS3BP;BMP4;IFI6;S100A4
ethaverine-3375	4/100	0.009292	0.260892	0.042102	0.724338	-1.61491	7.555433	PDIA3;C3;TMPRSS3;AP1S1
genistein-5232	4/100	0.009292	0.260892	0.042102	0.724338	-1.61291	7.546079	BMP4;ID1;ID3;TSPAN1
canrenoic acid-6783	4/100	0.009292	0.260892	0.042102	0.724338	-1.6122	7.542767	IFITM1;IFI6;ISG15;TSPAN1
fosfosal-2997	4/100	0.009292	0.260892	0.042102	0.724338	-1.61037	7.53421	SLPI;ISG15;CKB;LY6E
3-acetamidocoumarin-7361	4/100	0.009292	0.260892	0.042102	0.724338	-1.60923	7.528881	IFITM1;SPINK1;IFI6;TSPAN1
nafcillin-4103	4/100	0.009292	0.260892	0.042102	0.724338	-1.60827	7.524378	BMP4;GPX2;S100A6;IFI6
dicloxacillin-3307	4/100	0.009292	0.260892	0.042102	0.724338	-1.60599	7.513708	BMP4;RNF43;TFF2;S100A4
aminophylline-5395	4/100	0.009292	0.260892	0.042102	0.724338	-1.6037	7.502998	BMP4;IFI27;IFI6;ISG15
meclozine-3285	4/100	0.009292	0.260892	0.042102	0.724338	-1.60318	7.500555	PDIA3;ID3;TFF2;SECTM1

acebutolol-1993	4/100	0.009292	0.260892	0.042102	0.724338	-1.60021	7.486662	C3;SLC12A2;TMEM123;SLPI
CP-320650-01-4382	4/100	0.009292	0.260892	0.042102	0.724338	-1.60003	7.485825	IFI27;TCN1;MMP3;SECTM1
cefsulodin-4148	4/100	0.009292	0.260892	0.042102	0.724338	-1.5994	7.482886	BMP4;MMP3;IFI6;AP1S1
LY-294002-361	4/100	0.009292	0.260892	0.042102	0.724338	-1.59775	7.475156	IFITM3;IFITM2;SLPI;HSPB1
dipivefrine-7124	4/100	0.009292	0.260892	0.042102	0.724338	-1.59735	7.473303	IGFBP2;CTSD;CTGF;LY6E
chlorcyclizine-3810	4/100	0.009292	0.260892	0.042102	0.724338	-1.59713	7.472237	IL32;MMP3;AP1S1;FOS
(-)-atenolol-3067	4/100	0.009292	0.260892	0.042102	0.724338	-1.59457	7.460279	IFITM3;TSPAN13;ANXA3;ISG15
fusidic acid-5353	4/100	0.009292	0.260892	0.042102	0.724338	-1.59358	7.45567	GPX2;TMPRSS3;IFI6;ISG15
ornidazole-2272	4/100	0.009292	0.260892	0.042102	0.724338	-1.59242	7.450228	RNF43;SLPI;TFF2;SECTM1
flurbiprofen-5269	4/100	0.009292	0.260892	0.042102	0.724338	-1.59117	7.444386	IFI27;IFI6;ID3;TGFBI
apigenin-4401	4/100	0.009292	0.260892	0.042102	0.724338	-1.59058	7.441637	BMP4;IFI27;ID1;IFI6
colchicine-4614	4/100	0.009292	0.260892	0.042102	0.724338	-1.58971	7.437558	TUBA1A;SPINK1;TUBB;IFI6
gramine-4118	4/100	0.009292	0.260892	0.042102	0.724338	-1.58907	7.434546	S100A6;TFF2;S100A4;ISG15
chlorzoxazone-2263	4/100	0.009292	0.260892	0.042102	0.724338	-1.58802	7.42963	C3;SECTM1;TGFBI;TSPAN1
acebutolol-4976	4/100	0.009292	0.260892	0.042102	0.724338	-1.58692	7.424505	RCN1;IFI27;IFI6;ISG15
hydralazine-4746	4/100	0.009292	0.260892	0.042102	0.724338	-1.58662	7.423071	PDIA3;AP1S1;S100A4;TSPAN1
(+)-isoprenaline-3384	4/100	0.009292	0.260892	0.042102	0.724338	-1.58494	7.415203	BMP4;C3;SLPI;AP1S1
aminophenazone-2222	4/100	0.009292	0.260892	0.042102	0.724338	-1.58241	7.403407	BMP4;GPX2;AP1S1;SECTM1
ioversol-3365	4/100	0.009292	0.260892	0.042102	0.724338	-1.58201	7.401504	PDIA3;SLPI;AP1S1;SECTM1
genistein-1073	4/100	0.009292	0.260892	0.042102	0.724338	-1.58189	7.400945	IFNGR2;ID1;ISG15;MIF
minaprine-4814	4/100	0.009292	0.260892	0.042102	0.724338	-1.57764	7.381061	GPX2;ID1;TSPAN1;CTSD
methyldopa-5272	4/100	0.009292	0.260892	0.042102	0.724338	-1.57739	7.379899	BMP4;SLPI;IFI27;TFF2
daunorubicin-4983	4/100	0.009292	0.260892	0.042102	0.724338	-1.57597	7.373269	ID1;TXNIP;ID3;IER2
theobromine-4958	4/100	0.009292	0.260892	0.042102	0.724338	-1.57429	7.365402	IFITM1;IFI27;IFI6;ISG15
fisetin-579	4/100	0.009292	0.260892	0.042102	0.724338	-1.5729	7.358883	ID1;CKS2;SOX9;IER2
medrysone-4266	4/100	0.009292	0.260892	0.042102	0.724338	-1.57224	7.355802	RPS14P3;TSPAN8;MMP1;IFI6
betaxolol-3208	4/100	0.009292	0.260892	0.042102	0.724338	-1.57065	7.348358	TMPRSS3;SECTM1;S100A4;LYZ
isoflupredone-1873	4/100	0.009292	0.260892	0.042102	0.724338	-1.56862	7.338861	RCN1;ANXA3;QPCT;IFI6
LY-294002-6976	4/100	0.009292	0.260892	0.042102	0.724338	-1.56755	7.333888	IFNGR2;TFF3;S100A4;TSPAN1
cefuroxime-2526	4/100	0.009292	0.260892	0.042102	0.724338	-1.56701	7.331329	TSPAN13;CCL20;ANXA3;QPCT
methyldopa-5637	4/100	0.009292	0.260892	0.042102	0.724338	-1.56576	7.325494	BMP4;RNF43;SLPI;TMPRSS3
pyrazinamide-4962	4/100	0.009292	0.260892	0.042102	0.724338	-1.56413	7.317884	IFI27;IFI6;ISG15;TSPAN1
methotrexate-5419	4/100	0.009292	0.260892	0.042102	0.724338	-1.56313	7.313198	BMP4;IFI27;CKS2;TXNIP
iocetamic acid-4425	4/100	0.009292	0.260892	0.042102	0.724338	-1.5625	7.310248	C3;IFI27;IFI6;AP1S1
paclitaxel-6720	4/100	0.009292	0.260892	0.042102	0.724338	-1.5625	7.31024	ID1;S100A4;PFN1;LY6E
chloropyramine-4589	4/100	0.009292	0.260892	0.042102	0.724338	-1.56204	7.30807	C3;GPX2;SOX9;TSPAN1
nialamide-3871	4/100	0.009292	0.260892	0.042102	0.724338	-1.56182	7.307071	C3;TFF2;SECTM1;ITM2C

retrorsine-4946	4/100	0.009292	0.260892	0.042102	0.724338	-1.56068	7.301742	GPX2;IFI27;IFI6;ISG15
nitrofural-2459	4/100	0.009292	0.260892	0.042102	0.724338	-1.5566	7.282625	GPX2;MMP3;IFI6;ATP1B1
naringin-3286	4/100	0.009292	0.260892	0.042102	0.724338	-1.55643	7.281835	PDIA3;SLPI;TFF2;TSPAN1
PHA-00851261E-3854	4/100	0.009292	0.260892	0.042102	0.724338	-1.55641	7.281749	RCN1;SLPI;IFI6;SECTM1
vinblastine-7556	4/100	0.009292	0.260892	0.042102	0.724338	-1.55584	7.279069	TUBA1C;TUBA1B;TUBA1A;TUBB
Prestwick-1080-4354	4/100	0.009292	0.260892	0.042102	0.724338	-1.5495	7.249434	ID1;IFI6;CTSD;LY6E
pancuronium bromide-4393	4/100	0.009292	0.260892	0.042102	0.724338	-1.54869	7.245616	RNF43;IFITM1;IFI6;ISG15
nifedipine-6006	4/100	0.009292	0.260892	0.042102	0.724338	-1.54796	7.242215	BMP4;IFITM1;IFI6;S100A4
ganciclovir-5389	4/100	0.009292	0.260892	0.042102	0.724338	-1.54714	7.23839	BMP4;IFI27;IFI6;TGFBI
parthenolide-5530	4/100	0.009292	0.260892	0.042102	0.724338	-1.54573	7.231775	LGALS3BP;IFNGR2;ENC1;TXNIP
oxetacaine-1903	4/100	0.009292	0.260892	0.042102	0.724338	-1.54417	7.224459	CDH3;TSPAN13;ANXA3;SECTM1
tetroquinone-4159	4/100	0.009292	0.260892	0.042102	0.724338	-1.54103	7.209809	SLPI;TCN1;IFI6;AP1S1
tanespimycin-2685	4/100	0.009292	0.260892	0.042102	0.724338	-1.54003	7.205106	IGFBP2;CD14;IER2;LY6E
naltrexone-2209	4/100	0.009292	0.260892	0.042102	0.724338	-1.53958	7.203017	GPX2;AP1S1;SECTM1;S100A4
papaverine-6245	4/100	0.009292	0.260892	0.042102	0.724338	-1.53957	7.202943	PDIA3;TXNIP;ID3;AP1S1
lorglumide-5254	4/100	0.009292	0.260892	0.042102	0.724338	-1.53924	7.201401	GPX2;ID1;IFI6;TSPAN1
doxorubicin-5671	4/100	0.009292	0.260892	0.042102	0.724338	-1.53785	7.194906	ID1;TXNIP;ID3;IER2
Prestwick-1084-6767	4/100	0.009292	0.260892	0.042102	0.724338	-1.53757	7.193599	RCN1;GPX2;IFI6;ETS2
paroxetine-3904	4/100	0.009292	0.260892	0.042102	0.724338	-1.53568	7.184755	C3;RCN1;QPCT;ITM2C
papaverine-1755	4/100	0.009292	0.260892	0.042102	0.724338	-1.53105	7.163095	SPP1;TXNIP;HSPB1;CD14
norcyclobenzaprine-4776	4/100	0.009292	0.260892	0.042102	0.724338	-1.53103	7.162986	QPCT;ID1;ENC1;IFI6
puromycin-6711	4/100	0.009292	0.260892	0.042102	0.724338	-1.52761	7.147024	LGALS3BP;GPX2;TUBB;TSPAN1
tretinoin-5208	4/100	0.009292	0.260892	0.042102	0.724338	-1.52619	7.14034	BMP4;TMPRSS3;S100A4;ISG15
tracazolate-7339	4/100	0.009292	0.260892	0.042102	0.724338	-1.52322	7.126445	BMP4;IFITM1;IFI6;TSPAN1
orphenadrine-3883	4/100	0.009292	0.260892	0.042102	0.724338	-1.51822	7.10307	RCN1;TFF2;SECTM1;ITM2C
protriptyline-6338	4/100	0.009292	0.260892	0.042102	0.724338	-1.51803	7.1022	TCN1;ID3;TSPAN1;CTSD
nitrendipine-5405	4/100	0.009292	0.260892	0.042102	0.724338	-1.5177	7.100648	RNF43;QPCT;IFI6;ISG15
quinethazone-3875	4/100	0.009292	0.260892	0.042102	0.724338	-1.5131	7.079126	C3;QPCT;TFF2;ITM2C
mifepristone-3185	4/100	0.009292	0.260892	0.042102	0.724338	-1.51284	7.077893	TMPRSS3;S100A4;LYZ;PGM1
piperlongumine-1764	4/100	0.009292	0.260892	0.042102	0.724338	-1.51276	7.077543	CCL20;IFNGR2;SPP1;CD14
rosiglitazone-4457	4/100	0.009292	0.260892	0.042102	0.724338	-1.51103	7.069443	BMP4;IFI6;CXCL1;CTGF
orlistat-6905	4/100	0.009292	0.260892	0.042102	0.724338	-1.51076	7.06815	ID1;PLP2;TSPAN1;CTSD
primidone-6723	4/100	0.009292	0.260892	0.042102	0.724338	-1.50986	7.063981	TCN1;ID1;CTSD;LY6E
pioglitazone-7528	4/100	0.009292	0.260892	0.042102	0.724338	-1.50953	7.062408	RNF43;ID3;CXCL1;CTGF
tobramycin-4162	4/100	0.009292	0.260892	0.042102	0.724338	-1.50432	7.038059	RNF43;TCN1;IFI6;AP1S1
tretinoin-1211	4/100	0.009292	0.260892	0.042102	0.724338	-1.49389	6.989251	IL32;BMP4;IFI27;IFI6
xamoterol-3401	4/100	0.009292	0.260892	0.042102	0.724338	-1.49358	6.987795	PIGR;RNF43;RCN1;ITM2C

tribenoside-3507	4/100	0.009292	0.260892	0.042102	0.724338	-1.47705	6.91044	LGALS3BP;BMP4;GPX2;S100A4
trichostatin A-6709	4/100	0.009292	0.260892	0.042102	0.724338	-1.47081	6.881263	LGALS3BP;IFNGR2;ID1;S100A4
3-hydroxy-DL-kynurenine-4681	3/100	0.049183	0.38132	0.140496	0.724338	-1.57163	4.734094	SPINK1;OLFM4;DUOX2
5155877-6549	3/100	0.049183	0.38132	0.140496	0.724338	-1.52383	4.590114	LGALS3BP;IFI27;TSPAN1
azathioprine-5262	3/100	0.049183	0.38132	0.140496	0.724338	-1.52334	4.588629	BMP4;SLPI;SOX9
3-acetylcoumarin-4664	3/100	0.049183	0.38132	0.140496	0.724338	-1.52125	4.582339	IL32;PDIA3;TGFBI
15-delta prostaglandin J2-5228	3/100	0.049183	0.38132	0.140496	0.724338	-1.51645	4.567879	BMP4;RNF43;IFI6
5707885-6438	3/100	0.049183	0.38132	0.140496	0.724338	-1.51546	4.564895	MMP3;TXNIP;AP1S1
16-phenyltetranorprostaglandin E2-7546	3/100	0.049183	0.38132	0.140496	0.724338	-1.51535	4.564547	BMP4;IFI6;CTGF
bepridil-2629	3/100	0.049183	0.38132	0.140496	0.724338	-1.51448	4.56193	PDIA3;LGALS3BP;BMP4
acacetin-3942	3/100	0.049183	0.38132	0.140496	0.724338	-1.51298	4.557415	ID1;AP1S1;TGFBI
0179445-0000-4758	3/100	0.049183	0.38132	0.140496	0.724338	-1.51231	4.555393	RCN1;IFI27;IFI6
berberine-2770	3/100	0.049183	0.38132	0.140496	0.724338	-1.51121	4.552091	GPX2;TMPRSS3;TXNIP
1,5-isoquinolinediol-543	3/100	0.049183	0.38132	0.140496	0.724338	-1.51009	4.548716	IFITM3;HSPB1;DYNLL1
0317956-0000-3855	3/100	0.049183	0.38132	0.140496	0.724338	-1.50932	4.546396	C3;RCN1;MMP3
alclometasone-5752	3/100	0.049183	0.38132	0.140496	0.724338	-1.50698	4.539344	C3;IFITM1;IFI6
albendazole-3164	3/100	0.049183	0.38132	0.140496	0.724338	-1.50606	4.536589	BMP4;AP1S1;LYZ
(+)-isoprenaline-6663	3/100	0.049183	0.38132	0.140496	0.724338	-1.50606	4.536577	C3;CTGF;PSMB9
5194442-6594	3/100	0.049183	0.38132	0.140496	0.724338	-1.50412	4.530744	BMP4;IFI6;TGFBI
bendroflumethiazide-3758	3/100	0.049183	0.38132	0.140496	0.724338	-1.50382	4.529823	GPX2;TSPAN8;MMP3
alvespimycin-6172	3/100	0.049183	0.38132	0.140496	0.724338	-1.50365	4.529307	SLPI;CTSD;LY6E
butoconazole-5388	3/100	0.049183	0.38132	0.140496	0.724338	-1.50058	4.520078	BMP4;IFI27;AP1S1
6-bromoindirubin-3'-oxime-7106	3/100	0.049183	0.38132	0.140496	0.724338	-1.50019	4.518897	ANXA3;AP1S1;CTGF
amoxapine-1513	3/100	0.049183	0.38132	0.140496	0.724338	-1.49997	4.518233	BMP4;TFF2;S100A4
alpha-estradiol-5207	3/100	0.049183	0.38132	0.140496	0.724338	-1.49773	4.511496	LGALS3BP;QPCT;ID3
butoconazole-3288	3/100	0.049183	0.38132	0.140496	0.724338	-1.49731	4.510233	RPS14P3;TFF2;SECTM1
betonicine-3642	3/100	0.049183	0.38132	0.140496	0.724338	-1.49549	4.504744	SPINK1;MMP3;ITM2C
0317956-0000-3966	3/100	0.049183	0.38132	0.140496	0.724338	-1.49523	4.503946	QPCT;IFI6;AP1S1
3-acetylcoumarin-5259	3/100	0.049183	0.38132	0.140496	0.724338	-1.49402	4.500298	RNF43;IFI27;IFI6
(-)-catechin-1101	3/100	0.049183	0.38132	0.140496	0.724338	-1.49131	4.492155	S100P;TGFBI;PGM1
alclometasone-6094	3/100	0.049183	0.38132	0.140496	0.724338	-1.49067	4.490208	BMP4;QPCT;MMP3
cefotetan-4116	3/100	0.049183	0.38132	0.140496	0.724338	-1.48957	4.486904	RCN1;GPX2;IFI6
AG-013608-5949	3/100	0.049183	0.38132	0.140496	0.724338	-1.48882	4.484661	PROM1;CTGF;PSMB9
2,6-dimethylpiperidine-3889	3/100	0.049183	0.38132	0.140496	0.724338	-1.48756	4.480857	PDIA3;RCN1;QPCT
6-bromoindirubin-3'-oxime-7101	3/100	0.049183	0.38132	0.140496	0.724338	-1.48657	4.477882	TMPRSS3;MMP3;PLP2
6-azathymine-4106	3/100	0.049183	0.38132	0.140496	0.724338	-1.48612	4.476507	BMP4;QPCT;TFF2
antazoline-6775	3/100	0.049183	0.38132	0.140496	0.724338	-1.48517	4.473644	RCN1;TFF2;S100A4

clenbuterol-5266	3/100	0.049183	0.38132	0.140496	0.724338	-1.48474	4.472345	SLPI;TFF2;S100A4
buspirone-5343	3/100	0.049183	0.38132	0.140496	0.724338	-1.48301	4.467159	GPX2;IFI6;ISG15
alcuronium chloride-7345	3/100	0.049183	0.38132	0.140496	0.724338	-1.48129	4.461965	IFITM1;IFI6;AP1S1
arecoline-2657	3/100	0.049183	0.38132	0.140496	0.724338	-1.48082	4.460536	RCN1;TFF2;SECTM1
betonicine-3745	3/100	0.049183	0.38132	0.140496	0.724338	-1.48048	4.459529	C3;IFI6;PSMB9
AG-013608-6440	3/100	0.049183	0.38132	0.140496	0.724338	-1.47814	4.45248	C3;MMP3;CTGF
5666823-609	3/100	0.049183	0.38132	0.140496	0.724338	-1.47812	4.452402	GPX2;TCN1;DPEP1
estradiol-5238	3/100	0.049183	0.38132	0.140496	0.724338	-1.47782	4.451498	BMP4;QPCT;ID3
amiodarone-5253	3/100	0.049183	0.38132	0.140496	0.724338	-1.47481	4.442444	LGALS3BP;SLPI;SECTM1
bromperidol-2872	3/100	0.049183	0.38132	0.140496	0.724338	-1.47456	4.441687	BMP4;TMPRSS3;LY6E
crotamiton-5689	3/100	0.049183	0.38132	0.140496	0.724338	-1.47327	4.437795	GPX2;ID3;S100A4
alpha-estradiol-1635	3/100	0.049183	0.38132	0.140496	0.724338	-1.47245	4.435321	ID1;ID3;TGFBI
deferoxamine-3760	3/100	0.049183	0.38132	0.140496	0.724338	-1.47085	4.43051	BMP4;TCN1;MMP3
clozapine-5226	3/100	0.049183	0.38132	0.140496	0.724338	-1.4705	4.429466	RNF43;MMP3;SECTM1
carbenoxolone-4173	3/100	0.049183	0.38132	0.140496	0.724338	-1.46952	4.426505	TCN1;AP1S1;TFF2
aminocaproic acid-3122	3/100	0.049183	0.38132	0.140496	0.724338	-1.46801	4.421976	TSPAN13;TCN1;LY6E
dicycloverine-4405	3/100	0.049183	0.38132	0.140496	0.724338	-1.46781	4.421348	RNF43;IFI6;TFF2
bupropion-3180	3/100	0.049183	0.38132	0.140496	0.724338	-1.4649	4.412579	BMP4;S100A4;LYZ
chlorhexidine-5403	3/100	0.049183	0.38132	0.140496	0.724338	-1.46472	4.412042	RNF43;IFITM1;IFI6
3-aminobenzamide-590	3/100	0.049183	0.38132	0.140496	0.724338	-1.46273	4.406069	GPX2;ID3;DPEP1
cefoperazone-5424	3/100	0.049183	0.38132	0.140496	0.724338	-1.46197	4.403764	IFITM1;IFI27;IFI6
antazoline-1556	3/100	0.049183	0.38132	0.140496	0.724338	-1.46194	4.403684	IFITM3;SLPI;ANXA3
3-hydroxy-DL-kynurenine-1109	3/100	0.049183	0.38132	0.140496	0.724338	-1.46096	4.400722	IGFBP2;TGFBI;PRSS23
betahistine-4956	3/100	0.049183	0.38132	0.140496	0.724338	-1.46042	4.39911	IFITM3;IFI27;TSPAN1
AH-23848-6903	3/100	0.049183	0.38132	0.140496	0.724338	-1.45948	4.396272	PLP2;TSPAN1;CTSD
benzathine benzylpenicillin-2939	3/100	0.049183	0.38132	0.140496	0.724338	-1.45813	4.392196	ID3;ISG15;CTSD
ciprofloxacin-5299	3/100	0.049183	0.38132	0.140496	0.724338	-1.45789	4.391468	IFI6;ISG15;TSPAN1
atractyloside-7393	3/100	0.049183	0.38132	0.140496	0.724338	-1.45553	4.384365	CDH3;MMP1;IGFBP2
cefapirin-6790	3/100	0.049183	0.38132	0.140496	0.724338	-1.4555	4.384289	BMP4;RCN1;SECTM1
5194442-6558	3/100	0.049183	0.38132	0.140496	0.724338	-1.45537	4.383902	RNF43;PLP2;TSPAN1
0173570-0000-7389	3/100	0.049183	0.38132	0.140496	0.724338	-1.45521	4.383413	CKS2;TGFBI;CTGF
chloroquine-7012	3/100	0.049183	0.38132	0.140496	0.724338	-1.45468	4.381821	BMP4;IFITM1;TSPAN1
androsterone-4635	3/100	0.049183	0.38132	0.140496	0.724338	-1.45059	4.369504	BMP4;OLFM4;DUOX2
alvespimycin-1213	3/100	0.049183	0.38132	0.140496	0.724338	-1.45053	4.369321	CCL20;ID1;IER2
carbamazepine-5518	3/100	0.049183	0.38132	0.140496	0.724338	-1.44918	4.365245	RNF43;GPX2;TGFBI
clomipramine-4487	3/100	0.049183	0.38132	0.140496	0.724338	-1.44901	4.364717	TUBA1A;IFI6;ID3
chlorambucil-4523	3/100	0.049183	0.38132	0.140496	0.724338	-1.44583	4.355164	CDH3;TGFBI;TSPAN1

cetirizine-4231	3/100	0.049183	0.38132	0.140496	0.724338	-1.44401	4.349674	GPX2;IFI6;PSMB9
alvespimycin-6973	3/100	0.049183	0.38132	0.140496	0.724338	-1.4427	4.345722	ID1;ID3;IER2
cyanocobalamin-4395	3/100	0.049183	0.38132	0.140496	0.724338	-1.43659	4.327324	IFI27;IFI6;AP1S1
colecalciferol-3298	3/100	0.049183	0.38132	0.140496	0.724338	-1.43624	4.326262	RNF43;IFI6;TFF2
cefotiam-3319	3/100	0.049183	0.38132	0.140496	0.724338	-1.43543	4.32381	C3;RPS14P3;IFI6
4,5-dianilinophthalimide-624	3/100	0.049183	0.38132	0.140496	0.724338	-1.43244	4.314829	TMEM123;ID3;TGFBI
chlorphenesin-2279	3/100	0.049183	0.38132	0.140496	0.724338	-1.43136	4.311558	C3;GPX2;SLPI
dehydrocholic acid-4620	3/100	0.049183	0.38132	0.140496	0.724338	-1.43101	4.310495	IL32;OLFM4;DUOX2
cefotaxime-2235	3/100	0.049183	0.38132	0.140496	0.724338	-1.42992	4.307225	BMP4;C3;AP1S1
cisapride-2443	3/100	0.049183	0.38132	0.140496	0.724338	-1.42941	4.305676	GPX2;IFI6;LY6E
brinzolamide-1615	3/100	0.049183	0.38132	0.140496	0.724338	-1.42927	4.305257	SLC12A2;TUBA1A;IFI6
deferoxamine-3936	3/100	0.049183	0.38132	0.140496	0.724338	-1.42892	4.30422	QPCT;MMP3;TFF2
dobutamine-5386	3/100	0.049183	0.38132	0.140496	0.724338	-1.42856	4.303124	IFI27;QPCT;IFI6
cloxacillin-2289	3/100	0.049183	0.38132	0.140496	0.724338	-1.42753	4.300015	BMP4;C3;RNF43
dexibuprofen-6712	3/100	0.049183	0.38132	0.140496	0.724338	-1.42668	4.297458	ID1;S100A4;TSPAN1
chlorphenamine-6773	3/100	0.049183	0.38132	0.140496	0.724338	-1.42402	4.289469	BMP4;IFI6;SECTM1
Chicago Sky Blue 6B-4971	3/100	0.049183	0.38132	0.140496	0.724338	-1.42232	4.284323	IFI27;IFI6;ISG15
chlorphenesin-2115	3/100	0.049183	0.38132	0.140496	0.724338	-1.42148	4.281794	TSPAN8;CCL20;ITM2C
ciclosporin-1331	3/100	0.049183	0.38132	0.140496	0.724338	-1.42145	4.281717	CCL20;SPP1;CD14
dihydroergotamine-2244	3/100	0.049183	0.38132	0.140496	0.724338	-1.42087	4.279979	BMP4;AP1S1;TFF2
desipramine-3212	3/100	0.049183	0.38132	0.140496	0.724338	-1.42058	4.279093	GPX2;SECTM1;LYZ
calmidazolium-486	3/100	0.049183	0.38132	0.140496	0.724338	-1.42057	4.279072	IFITM3;CDH3;DPEP1
cefixime-4390	3/100	0.049183	0.38132	0.140496	0.724338	-1.42035	4.278395	LGALS3BP;RNF43;IFI6
diltiazem-2032	3/100	0.049183	0.38132	0.140496	0.724338	-1.41992	4.277099	TUBA1A;ITGAV;CTSD
diloxanide-3399	3/100	0.049183	0.38132	0.140496	0.724338	-1.41802	4.27137	PDIA3;RNF43;QPCT
ethosuximide-2280	3/100	0.049183	0.38132	0.140496	0.724338	-1.41635	4.266339	C3;RNF43;SLPI
droperidol-5690	3/100	0.049183	0.38132	0.140496	0.724338	-1.41475	4.261518	IFI27;IFI6;S100A4
colforsin-913	3/100	0.049183	0.38132	0.140496	0.724338	-1.41281	4.255689	BMP4;ID1;TXNIP
finasteride-4766	3/100	0.049183	0.38132	0.140496	0.724338	-1.40988	4.246877	SPINK1;IFI27;IFI6
colforsin-783	3/100	0.049183	0.38132	0.140496	0.724338	-1.4092	4.244801	CSTB;DEK;ITM2C
clofibrate-263	3/100	0.049183	0.38132	0.140496	0.724338	-1.40879	4.243565	IFI27;MMP3;DSG2
captopril-4410	3/100	0.049183	0.38132	0.140496	0.724338	-1.40762	4.240057	C3;MMP3;AP1S1
digoxigenin-3397	3/100	0.049183	0.38132	0.140496	0.724338	-1.40752	4.239743	BMP4;C3;SECTM1
etamsylate-4399	3/100	0.049183	0.38132	0.140496	0.724338	-1.40597	4.235093	IFI27;IFI6;AP1S1
dexpropranolol-3553	3/100	0.049183	0.38132	0.140496	0.724338	-1.40499	4.232124	LGALS3BP;GPX2;S100A4
cobalt chloride-383	3/100	0.049183	0.38132	0.140496	0.724338	-1.40486	4.231742	GPX2;PDZK1IP1;TSPAN1
celastrol-887	3/100	0.049183	0.38132	0.140496	0.724338	-1.40205	4.223285	S100A6;TXNIP;CTSD

edrophonium chloride-1519	3/100	0.049183	0.38132	0.140496	0.724338	-1.40164	4.222031	C3;TFF2;S100A4
flunisolide-3828	3/100	0.049183	0.38132	0.140496	0.724338	-1.4005	4.218609	GPX2;IFI6;S100A4
diphenhydramine-6020	3/100	0.049183	0.38132	0.140496	0.724338	-1.39916	4.214586	IFITM1;IFI6;AP1S1
deptropine-5543	3/100	0.049183	0.38132	0.140496	0.724338	-1.39844	4.212412	LGALS3BP;GPX2;IFNGR2
eucatropine-3759	3/100	0.049183	0.38132	0.140496	0.724338	-1.39705	4.208205	TCN1;TGFBI;PSMB9
felbinac-3398	3/100	0.049183	0.38132	0.140496	0.724338	-1.39581	4.204471	C3;QPCT;ITM2C
caffeic acid-5352	3/100	0.049183	0.38132	0.140496	0.724338	-1.39498	4.201981	IFITM1;IFI6;ISG15
CP-690334-01-3823	3/100	0.049183	0.38132	0.140496	0.724338	-1.39408	4.199257	RNF43;MMP3;CXCL1
furosemide-6841	3/100	0.049183	0.38132	0.140496	0.724338	-1.39394	4.19884	GPX2;IFI27;IFI6
ciclosporin-4411	3/100	0.049183	0.38132	0.140496	0.724338	-1.39197	4.1929	BMP4;IFI6;AP1S1
daunorubicin-7511	3/100	0.049183	0.38132	0.140496	0.724338	-1.39027	4.187778	ID1;TSPAN1;PHLDA1
dehydrocholic acid-1523	3/100	0.049183	0.38132	0.140496	0.724338	-1.38974	4.186184	C3;RNF43;TFF2
doxylamine-4819	3/100	0.049183	0.38132	0.140496	0.724338	-1.38821	4.181587	BMP4;GPX2;S100A6
epitiostanol-4788	3/100	0.049183	0.38132	0.140496	0.724338	-1.3878	4.180345	LGALS3BP;ID1;TSPAN1
dexamethasone-255	3/100	0.049183	0.38132	0.140496	0.724338	-1.38706	4.178138	ID3;S100A4;TGFBI
hydralazine-4282	3/100	0.049183	0.38132	0.140496	0.724338	-1.38417	4.169412	RPS14P3;MMP3;IFI6
estriol-3563	3/100	0.049183	0.38132	0.140496	0.724338	-1.38368	4.167936	GPX2;ID1;ID3
ganciclovir-3368	3/100	0.049183	0.38132	0.140496	0.724338	-1.38246	4.164275	PDIA3;AP1S1;SECTM1
flufenamic acid-2267	3/100	0.049183	0.38132	0.140496	0.724338	-1.38244	4.164202	GPX2;SLPI;TFF2
fenoprofen-4736	3/100	0.049183	0.38132	0.140496	0.724338	-1.3802	4.157446	TMPRSS3;IFI6;AP1S1
dimethadione-5668	3/100	0.049183	0.38132	0.140496	0.724338	-1.37991	4.156583	BMP4;IFI27;IFNGR2
estradiol-1666	3/100	0.049183	0.38132	0.140496	0.724338	-1.37898	4.153798	BMP4;ID3;TSPAN1
epirizole-7292	3/100	0.049183	0.38132	0.140496	0.724338	-1.37629	4.145692	TCN1;IFI6;ETS2
iopromide-6842	3/100	0.049183	0.38132	0.140496	0.724338	-1.37442	4.140063	IFITM1;IFI27;IFI6
famotidine-1529	3/100	0.049183	0.38132	0.140496	0.724338	-1.37174	4.131964	RNF43;TFF2;S100A4
deferoxamine-460	3/100	0.049183	0.38132	0.140496	0.724338	-1.37061	4.128565	CTSE;PRSS23;HIF1A
lithocholic acid-3899	3/100	0.049183	0.38132	0.140496	0.724338	-1.37045	4.128104	RCN1;TFF2;ITM2C
diphemanil metilsulfate-4416	3/100	0.049183	0.38132	0.140496	0.724338	-1.36903	4.123814	SPINK1;IFI27;AP1S1
eticlopride-3393	3/100	0.049183	0.38132	0.140496	0.724338	-1.36784	4.120223	PDIA3;QPCT;AP1S1
finasteride-2206	3/100	0.049183	0.38132	0.140496	0.724338	-1.36708	4.117939	PLCB4;TSPAN13;IFI6
doxazosin-4988	3/100	0.049183	0.38132	0.140496	0.724338	-1.36604	4.11481	PDIA3;IFI27;IFI6
lansoprazole-6009	3/100	0.049183	0.38132	0.140496	0.724338	-1.36418	4.109212	IFITM1;GPX2;IFI6
lomefloxacin-4281	3/100	0.049183	0.38132	0.140496	0.724338	-1.36284	4.105153	GPX2;MMP3;IFI6
fluorocurarine-2521	3/100	0.049183	0.38132	0.140496	0.724338	-1.36235	4.103681	SLC12A2;ANXA3;QPCT
fludrocortisone-3977	3/100	0.049183	0.38132	0.140496	0.724338	-1.36101	4.099661	SPINK1;SLPI;AP1S1
dequalinium chloride-1276	3/100	0.049183	0.38132	0.140496	0.724338	-1.35988	4.096249	C3;SPP1;TXNIP
epitiostanol-7342	3/100	0.049183	0.38132	0.140496	0.724338	-1.35911	4.093945	BMP4;ENC1;IFI6

dioxybenzone-3101	3/100	0.049183	0.38132	0.140496	0.724338	-1.35815	4.091052	SLPI;TCN1;IFI6
demecarium bromide-6269	3/100	0.049183	0.38132	0.140496	0.724338	-1.35657	4.086279	TFF2;SECTM1;PGM1
dimethadione-3367	3/100	0.049183	0.38132	0.140496	0.724338	-1.35573	4.083743	PDIA3;GPX2;AP1S1
flupentixol-2643	3/100	0.049183	0.38132	0.140496	0.724338	-1.35514	4.081979	PDIA3;BMP4;RCN1
eticlopride-5695	3/100	0.049183	0.38132	0.140496	0.724338	-1.35376	4.077828	BMP4;IFI27;S100A4
estrone-6647	3/100	0.049183	0.38132	0.140496	0.724338	-1.35356	4.07722	IFI6;TGFBI;PSMB9
harmaline-4968	3/100	0.049183	0.38132	0.140496	0.724338	-1.3533	4.076423	IFI27;IFI6;ISG15
ketorolac-5988	3/100	0.049183	0.38132	0.140496	0.724338	-1.34457	4.050142	RNF43;IFI6;S100A4
fusaric acid-4105	3/100	0.049183	0.38132	0.140496	0.724338	-1.34259	4.044169	BMP4;S100A4;LY6E
hexetidine-3318	3/100	0.049183	0.38132	0.140496	0.724338	-1.34242	4.043658	PDIA3;C3;IFI6
fluticasone-7348	3/100	0.049183	0.38132	0.140496	0.724338	-1.34149	4.040859	RNF43;IFITM1;IFI6
guanabenz-4642	3/100	0.049183	0.38132	0.140496	0.724338	-1.34124	4.040102	BMP4;SPINK1;OLFM4
gabexate-2937	3/100	0.049183	0.38132	0.140496	0.724338	-1.34123	4.040086	ANXA3;TCN1;IFI6
glafenine-7418	3/100	0.049183	0.38132	0.140496	0.724338	-1.33987	4.035985	ID1;ID3;AP1S1
gibberellic acid-7330	3/100	0.049183	0.38132	0.140496	0.724338	-1.33861	4.032196	BMP4;IFITM1;IFI6
epiandrosterone-3306	3/100	0.049183	0.38132	0.140496	0.724338	-1.33768	4.029387	RPS14P3;RNF43;SECTM1
fluorometholone-6071	3/100	0.049183	0.38132	0.140496	0.724338	-1.33508	4.021549	SLPI;TFF2;S100A4
fluvoxamine-7333	3/100	0.049183	0.38132	0.140496	0.724338	-1.33243	4.013575	IFITM1;ISG15;IER2
liothyronine-4947	3/100	0.049183	0.38132	0.140496	0.724338	-1.32985	4.005806	IFI27;IFI6;ISG15
fluspirilene-3086	3/100	0.049183	0.38132	0.140496	0.724338	-1.32841	4.001443	SLPI;ATP1B1;LY6E
erastin-6412	3/100	0.049183	0.38132	0.140496	0.724338	-1.32799	4.000199	QPCT;CD14;PROM1
H-7-5963	3/100	0.049183	0.38132	0.140496	0.724338	-1.32763	3.999121	ID1;ID3;IER2
gliclazide-5514	3/100	0.049183	0.38132	0.140496	0.724338	-1.32597	3.994094	TMPRSS3;QPCT;TSPAN1
isoflupredone-1832	3/100	0.049183	0.38132	0.140496	0.724338	-1.32557	3.992888	TUBA1C;IFI27;PHLDA1
pirenperone-5274	3/100	0.049183	0.38132	0.140496	0.724338	-1.32542	3.992458	MMP3;TFF2;SECTM1
naltrexone-6241	3/100	0.049183	0.38132	0.140496	0.724338	-1.32453	3.98976	GPX2;SECTM1;TSPAN1
naproxen-6794	3/100	0.049183	0.38132	0.140496	0.724338	-1.32452	3.989743	BMP4;IFI6;AP1S1
diperodon-1575	3/100	0.049183	0.38132	0.140496	0.724338	-1.32295	3.985019	ENC1;ISG15;LYZ
ioxaglic acid-7470	3/100	0.049183	0.38132	0.140496	0.724338	-1.32274	3.984377	GPX2;TMPRSS3;AP1S1
fulvestrant-4462	3/100	0.049183	0.38132	0.140496	0.724338	-1.32208	3.982384	BMP4;RNF43;IFI6
flumequine-1429	3/100	0.049183	0.38132	0.140496	0.724338	-1.32159	3.980902	RPS14P3;TUBA1A;LYZ
ethionamide-4418	3/100	0.049183	0.38132	0.140496	0.724338	-1.32044	3.977444	IFI27;QPCT;IFI6
lithocholic acid-4373	3/100	0.049183	0.38132	0.140496	0.724338	-1.31907	3.973336	MMP3;TFF2;ITM2C
lycorine-6051	3/100	0.049183	0.38132	0.140496	0.724338	-1.31657	3.965806	BMP4;GPX2;MMP3
LY-294002-1239	3/100	0.049183	0.38132	0.140496	0.724338	-1.31558	3.962796	TSPAN8;CCL20;PSMB9
metamizole sodium-3929	3/100	0.049183	0.38132	0.140496	0.724338	-1.31549	3.96254	SPINK1;IFI6;AP1S1
ketoprofen-2354	3/100	0.049183	0.38132	0.140496	0.724338	-1.3141	3.958352	SLC12A2;TSPAN13;LY6E

lorglumide-6456	3/100	0.049183	0.38132	0.140496	0.724338	-1.31376	3.957325	ENC1;SECTM1;TSPAN1
flufenamic acid-5478	3/100	0.049183	0.38132	0.140496	0.724338	-1.3125	3.953543	RNF43;GPX2;IFNGR2
fulvestrant-6918	3/100	0.049183	0.38132	0.140496	0.724338	-1.31249	3.953489	IFI6;PLP2;TSPAN1
pancuronium bromide-7329	3/100	0.049183	0.38132	0.140496	0.724338	-1.31119	3.949581	BMP4;IFITM1;GPX2
isosorbide-2183	3/100	0.049183	0.38132	0.140496	0.724338	-1.31046	3.947377	C3;IFI6;LY6E
fulvestrant-1663	3/100	0.049183	0.38132	0.140496	0.724338	-1.3095	3.944485	BMP4;TMPRSS3;PRSS23
loxapine-1516	3/100	0.049183	0.38132	0.140496	0.724338	-1.30641	3.935193	GPX2;TFF2;S100A4
geldanamycin-611	3/100	0.049183	0.38132	0.140496	0.724338	-1.30636	3.935039	TCN1;ID1;IER2
piperacetazine-6152	3/100	0.049183	0.38132	0.140496	0.724338	-1.30505	3.931077	TSPAN13;CCL20;TCN1
doxorubicin-4610	3/100	0.049183	0.38132	0.140496	0.724338	-1.30359	3.92668	ID1;ID3;IER2
indometacin-7409	3/100	0.049183	0.38132	0.140496	0.724338	-1.30288	3.924548	AP1S1;CKB;TSPAN1
isradipine-6347	3/100	0.049183	0.38132	0.140496	0.724338	-1.30283	3.924399	CDH3;TSPAN8;TSPAN1
monorden-953	3/100	0.049183	0.38132	0.140496	0.724338	-1.29904	3.912982	ID1;PGM1;IER2
N-acetyl-L-leucine-5683	3/100	0.049183	0.38132	0.140496	0.724338	-1.29812	3.910224	LGALS3BP;IFI27;IFI6
methoxamine-4972	3/100	0.049183	0.38132	0.140496	0.724338	-1.29781	3.90927	RNF43;IFI27;S100A4
gallamine triethiodide-1375	3/100	0.049183	0.38132	0.140496	0.724338	-1.29759	3.908608	SLC12A2;TMEM123;ITGAV
geldanamycin-1228	3/100	0.049183	0.38132	0.140496	0.724338	-1.29538	3.901973	IL32;BMP4;IER2
perphenazine-5698	3/100	0.049183	0.38132	0.140496	0.724338	-1.29394	3.897625	BMP4;IFI27;TMPRSS3
meptazinol-4188	3/100	0.049183	0.38132	0.140496	0.724338	-1.2936	3.896617	ID1;S100A4;TSPAN1
nabumetone-3108	3/100	0.049183	0.38132	0.140496	0.724338	-1.29339	3.895958	GPX2;PLCB4;TSPAN13
estradiol-5318	3/100	0.049183	0.38132	0.140496	0.724338	-1.29324	3.895509	IFI6;ID3;PGM1
hexylcaine-6244	3/100	0.049183	0.38132	0.140496	0.724338	-1.29265	3.89374	TFF2;SECTM1;S100A4
nitrofural-3320	3/100	0.049183	0.38132	0.140496	0.724338	-1.29212	3.892143	C3;IFI6;TSPAN1
lansoprazole-2967	3/100	0.049183	0.38132	0.140496	0.724338	-1.29119	3.889355	QPCT;SPP1;FOS
metformin-4	3/100	0.049183	0.38132	0.140496	0.724338	-1.28633	3.87469	LCN2;PDZK1IP1;TSPAN1
fluphenazine-3194	3/100	0.049183	0.38132	0.140496	0.724338	-1.28467	3.869706	BMP4;GPX2;LYZ
oxybutynin-3168	3/100	0.049183	0.38132	0.140496	0.724338	-1.28444	3.868995	BMP4;GPX2;LYZ
nordihydroguaiaretic acid-6942	3/100	0.049183	0.38132	0.140496	0.724338	-1.28329	3.865538	LGALS3BP;IFNGR2;TGFBI
meclozine-4646	3/100	0.049183	0.38132	0.140496	0.724338	-1.28236	3.862746	IL32;CDH3;TSPAN1
metacycline-4143	3/100	0.049183	0.38132	0.140496	0.724338	-1.28139	3.859827	TCN1;AP1S1;TSPAN1
niridazole-5682	3/100	0.049183	0.38132	0.140496	0.724338	-1.27952	3.854194	BMP4;IFI27;ISG15
pioglitazone-5977	3/100	0.049183	0.38132	0.140496	0.724338	-1.27763	3.8485	BMP4;CXCL1;CTGF
meptazinol-4774	3/100	0.049183	0.38132	0.140496	0.724338	-1.27562	3.842454	LGALS3BP;ID1;S100A6
monocrotaline-6771	3/100	0.049183	0.38132	0.140496	0.724338	-1.27343	3.835844	RCN1;IFI6;TSPAN1
paroxetine-4378	3/100	0.049183	0.38132	0.140496	0.724338	-1.27062	3.827378	C3;SPINK1;ITM2C
mebendazole-7370	3/100	0.049183	0.38132	0.140496	0.724338	-1.26981	3.824951	TUBA1A;MMP1;TUBB
ribostamycin-6765	3/100	0.049183	0.38132	0.140496	0.724338	-1.26816	3.819972	RCN1;IFI27;IFI6

oxprenolol-5871	3/100	0.049183	0.38132	0.140496	0.724338	-1.26671	3.815591	TUBA1A;MMP1;S100A4
phenindione-7289	3/100	0.049183	0.38132	0.140496	0.724338	-1.26559	3.812231	PDIA3;IGFBP2;IFI6
phentolamine-3971	3/100	0.049183	0.38132	0.140496	0.724338	-1.26423	3.808147	SLPI;MMP3;AP1S1
pilocarpine-3300	3/100	0.049183	0.38132	0.140496	0.724338	-1.26404	3.80755	RNF43;SLPI;IFI6
naringenin-4422	3/100	0.049183	0.38132	0.140496	0.724338	-1.2632	3.805039	RNF43;MMP3;AP1S1
pilocarpine-2438	3/100	0.049183	0.38132	0.140496	0.724338	-1.26292	3.80419	GPX2;IFI6;ISG15
mexiletine-3973	3/100	0.049183	0.38132	0.140496	0.724338	-1.26187	3.801022	AP1S1;CTGF;ITM2C
phenformin-4747	3/100	0.049183	0.38132	0.140496	0.724338	-1.26161	3.800254	IFI6;TXNIP;TSPAN1
methotrexate-3214	3/100	0.049183	0.38132	0.140496	0.724338	-1.26149	3.799886	BMP4;CKS2;TXNIP
pimozide-3178	3/100	0.049183	0.38132	0.140496	0.724338	-1.26088	3.798045	LGALS3BP;BMP4;LYZ
mifepristone-5827	3/100	0.049183	0.38132	0.140496	0.724338	-1.25924	3.793104	CDH3;CXCL1;CTGF
hydrocotarnine-1772	3/100	0.049183	0.38132	0.140496	0.724338	-1.25729	3.787223	C3;GPI;TUBA1A
luteolin-5004	3/100	0.049183	0.38132	0.140496	0.724338	-1.25586	3.782932	ID1;ID3;S100A4
harmine-1758	3/100	0.049183	0.38132	0.140496	0.724338	-1.25494	3.780136	TUBA1A;TXNIP;S100P
PF-00539745-00-5974	3/100	0.049183	0.38132	0.140496	0.724338	-1.25291	3.77405	SPINK1;SECTM1;ITM2C
piretanide-6828	3/100	0.049183	0.38132	0.140496	0.724338	-1.25259	3.773065	IFI27;IFI6;TSPAN1
indapamide-3970	3/100	0.049183	0.38132	0.140496	0.724338	-1.25216	3.771785	SLPI;AP1S1;ITM2C
pipenzolate bromide-6821	3/100	0.049183	0.38132	0.140496	0.724338	-1.25187	3.770914	IFI27;TMPRSS3;IFI6
pentamidine-4396	3/100	0.049183	0.38132	0.140496	0.724338	-1.25054	3.766911	IFITM1;IFI27;AP1S1
piromidic acid-4398	3/100	0.049183	0.38132	0.140496	0.724338	-1.24889	3.761926	IFI27;IFI6;AP1S1
PHA-00745360-3907	3/100	0.049183	0.38132	0.140496	0.724338	-1.24801	3.75926	QPCT;TFF2;ITM2C
Prestwick-857-4980	3/100	0.049183	0.38132	0.140496	0.724338	-1.24593	3.753016	IFI27;IFI6;ISG15
papaverine-2747	3/100	0.049183	0.38132	0.140496	0.724338	-1.24544	3.751545	PDIA3;RNF43;TMPRSS3
imipenem-1724	3/100	0.049183	0.38132	0.140496	0.724338	-1.2454	3.751409	RPS14P3;ISG15;MEST
nifurtimox-4953	3/100	0.049183	0.38132	0.140496	0.724338	-1.24504	3.750322	IFI27;IFI6;ISG15
piperacillin-3845	3/100	0.049183	0.38132	0.140496	0.724338	-1.2445	3.748703	RCN1;TFF2;S100A4
Prestwick-1080-3878	3/100	0.049183	0.38132	0.140496	0.724338	-1.24409	3.747467	BMP4;RCN1;ITM2C
PF-00562151-00-6912	3/100	0.049183	0.38132	0.140496	0.724338	-1.24306	3.744369	LGALS3BP;GPI;PLP2
Prestwick-664-4737	3/100	0.049183	0.38132	0.140496	0.724338	-1.24226	3.741968	GPX2;AP1S1;TFF2
prochlorperazine-5212	3/100	0.049183	0.38132	0.140496	0.724338	-1.24051	3.736694	RNF43;GPX2;QPCT
sulfafurazole-5257	3/100	0.049183	0.38132	0.140496	0.724338	-1.23961	3.733976	SLPI;IFI27;S100A4
Prestwick-967-7346	3/100	0.049183	0.38132	0.140496	0.724338	-1.23909	3.732404	LGALS3BP;IFI6;AP1S1
procyclidine-4817	3/100	0.049183	0.38132	0.140496	0.724338	-1.2387	3.731242	TFF2;DSG2;ETS2
sotalol-7338	3/100	0.049183	0.38132	0.140496	0.724338	-1.23658	3.724849	GPX2;IFI6;S100A4
prednisolone-5526	3/100	0.049183	0.38132	0.140496	0.724338	-1.23649	3.724586	TMPRSS3;S100A6;TGFBI
piperacetazine-3574	3/100	0.049183	0.38132	0.140496	0.724338	-1.23638	3.724236	LGALS3BP;RNF43;SECTM1
mitoxantrone-5354	3/100	0.049183	0.38132	0.140496	0.724338	-1.23632	3.724069	TXNIP;ID3;IER2

nicotinic acid-3381	3/100	0.049183	0.38132	0.140496	0.724338	-1.2362	3.723709	PDIA3;C3;SECTM1
parthenolide-5105	3/100	0.049183	0.38132	0.140496	0.724338	-1.23608	3.723328	IFI6;CXCL1;PHLDA1
PNU-0293363-6563	3/100	0.049183	0.38132	0.140496	0.724338	-1.23565	3.722043	TSPAN8;CKS2;CTGF
progesterone-3287	3/100	0.049183	0.38132	0.140496	0.724338	-1.23172	3.710205	PDIA3;RPS14P3;SECTM1
nocodazole-1393	3/100	0.049183	0.38132	0.140496	0.724338	-1.2317	3.710138	SPP1;HSPB1;MEST
mitoxantrone-6755	3/100	0.049183	0.38132	0.140496	0.724338	-1.23136	3.709114	ID1;IER2;CTGF
nocodazole-7145	3/100	0.049183	0.38132	0.140496	0.724338	-1.2304	3.706231	TUBA1A;MMP1;TUBB
PF-00875133-00-5967	3/100	0.049183	0.38132	0.140496	0.724338	-1.22895	3.70185	BMP4;RCN1;IFI6
Prestwick-675-3682	3/100	0.049183	0.38132	0.140496	0.724338	-1.22862	3.700878	C3;MMP3;IFI6
sparteine-4391	3/100	0.049183	0.38132	0.140496	0.724338	-1.22693	3.69578	IFI27;IFI6;AP1S1
sulfamethoxazole-4690	3/100	0.049183	0.38132	0.140496	0.724338	-1.2242	3.687564	RCN1;QPCT;TSPAN1
probucol-5261	3/100	0.049183	0.38132	0.140496	0.724338	-1.22129	3.678783	GPX2;IFI27;S100A4
telenzepine-5521	3/100	0.049183	0.38132	0.140496	0.724338	-1.21949	3.673367	LGALS3BP;IFNGR2;TSPAN1
riluzole-2295	3/100	0.049183	0.38132	0.140496	0.724338	-1.21768	3.667909	GPX2;TFF2;TSPAN1
propafenone-6336	3/100	0.049183	0.38132	0.140496	0.724338	-1.21517	3.660366	CDH3;ID1;TFF2
stachydrine-6805	3/100	0.049183	0.38132	0.140496	0.724338	-1.21487	3.659459	BMP4;IFI27;IFI6
sulindac-1693	3/100	0.049183	0.38132	0.140496	0.724338	-1.21364	3.655752	PDIA3;TMPRSS3;ID3
salbutamol-3677	3/100	0.049183	0.38132	0.140496	0.724338	-1.21311	3.654144	TSPAN8;MMP3;CTGF
piroxicam-1405	3/100	0.049183	0.38132	0.140496	0.724338	-1.20989	3.644434	IFITM3;C3;HSPB1
sulfabenzamide-4979	3/100	0.049183	0.38132	0.140496	0.724338	-1.20893	3.641543	IFI27;IFI6;ISG15
pyrithyldione-6801	3/100	0.049183	0.38132	0.140496	0.724338	-1.20657	3.634442	RCN1;GPX2;SECTM1
thiamazole-4372	3/100	0.049183	0.38132	0.140496	0.724338	-1.20647	3.634146	SLPI;IFI27;ITM2C
sulfaphenazole-1836	3/100	0.049183	0.38132	0.140496	0.724338	-1.20516	3.630204	CCL20;ANXA3;QPCT
sulmazole-4009	3/100	0.049183	0.38132	0.140496	0.724338	-1.20473	3.628893	MMP3;S100A4;TSPAN1
promethazine-6717	3/100	0.049183	0.38132	0.140496	0.724338	-1.20394	3.626518	ID1;TSPAN1;CTSD
tetramisole-4412	3/100	0.049183	0.38132	0.140496	0.724338	-1.20372	3.625873	IFI27;IFI6;AP1S1
profenamine-3376	3/100	0.049183	0.38132	0.140496	0.724338	-1.20212	3.621043	PDIA3;C3;SECTM1
tanespimycin-4450	3/100	0.049183	0.38132	0.140496	0.724338	-1.2021	3.620982	BMP4;ID1;PSMB9
Prestwick-1085-6250	3/100	0.049183	0.38132	0.140496	0.724338	-1.2014	3.61888	IGFBP2;TFF3;PGM1
Prestwick-664-6033	3/100	0.049183	0.38132	0.140496	0.724338	-1.19859	3.610399	BMP4;GPX2;MMP3
tanespimycin-4442	3/100	0.049183	0.38132	0.140496	0.724338	-1.19544	3.600933	BMP4;ID1;ID3
podophyllotoxin-5841	3/100	0.049183	0.38132	0.140496	0.724338	-1.19521	3.60023	TUBA1A;TSPAN8;TUBB
tanespimycin-6978	3/100	0.049183	0.38132	0.140496	0.724338	-1.192	3.590549	LGALS3BP;ID1;IER2
serotonin-5268	3/100	0.049183	0.38132	0.140496	0.724338	-1.19141	3.588788	BMP4;SLPI;PGM1
propantheline bromide-3352	3/100	0.049183	0.38132	0.140496	0.724338	-1.19037	3.585655	GPX2;PLP2;TSPAN1
zardaverine-7347	3/100	0.049183	0.38132	0.140496	0.724338	-1.18785	3.578058	LGALS3BP;BMP4;IFI6
procaine-1796	3/100	0.049183	0.38132	0.140496	0.724338	-1.187	3.575497	IL32;SECTM1;LY6E

protriptyline-6498	3/100	0.049183	0.38132	0.140496	0.724338	-1.18661	3.574321	GPX2;IFITM2;TFF2
prochlorperazine-6174	3/100	0.049183	0.38132	0.140496	0.724338	-1.18622	3.573146	RCN1;ANXA3;TCN1
tanespimycin-6944	3/100	0.049183	0.38132	0.140496	0.724338	-1.18582	3.571939	IFNGR2;ID1;IER2
valproic acid-5206	3/100	0.049183	0.38132	0.140496	0.724338	-1.18572	3.571635	RNF43;TMPRSS3;MMP3
rosiglitazone-1233	3/100	0.049183	0.38132	0.140496	0.724338	-1.1834	3.564657	BMP4;TSPAN8;SOX9
wortmannin-5240	3/100	0.049183	0.38132	0.140496	0.724338	-1.18225	3.561187	BMP4;GPX2;SECTM1
tiabendazole-4402	3/100	0.049183	0.38132	0.140496	0.724338	-1.18131	3.558362	IFI27;IFI6;AP1S1
Prestwick-1084-3546	3/100	0.049183	0.38132	0.140496	0.724338	-1.18042	3.555665	S100A6;S100A4;MIF
puromycin-5310	3/100	0.049183	0.38132	0.140496	0.724338	-1.17798	3.548335	LGALS3BP;TUBB;PRSS23
STOCK1N-28457-6906	3/100	0.049183	0.38132	0.140496	0.724338	-1.17705	3.545518	PDIA3;ID1;PLP2
todralazine-1677	3/100	0.049183	0.38132	0.140496	0.724338	-1.17615	3.542818	CDH3;S100A4;LY6E
tanespimycin-1225	3/100	0.049183	0.38132	0.140496	0.724338	-1.17604	3.542477	BMP4;SECTM1;IER2
scriptaid-6919	3/100	0.049183	0.38132	0.140496	0.724338	-1.1758	3.541761	CXCL1;PLP2;TSPAN1
tanespimycin-6943	3/100	0.049183	0.38132	0.140496	0.724338	-1.17463	3.538252	ID1;TSPAN1;IER2
terconazole-4407	3/100	0.049183	0.38132	0.140496	0.724338	-1.1726	3.53211	IFITM1;SPINK1;AP1S1
SR-95531-4820	3/100	0.049183	0.38132	0.140496	0.724338	-1.17221	3.530942	ID1;S100A6;TSPAN1
sulmazole-2153	3/100	0.049183	0.38132	0.140496	0.724338	-1.17082	3.526772	GPX2;SLPI;TFF2
zoxazolamine-5390	3/100	0.049183	0.38132	0.140496	0.724338	-1.16796	3.518161	IFITM1;IFI27;IFI6
troglitazone-4456	3/100	0.049183	0.38132	0.140496	0.724338	-1.16706	3.515427	ID3;CXCL1;CTGF
valproic acid-2700	3/100	0.049183	0.38132	0.140496	0.724338	-1.16686	3.514822	C3;SLPI;LY6E
sulindac-168	3/100	0.049183	0.38132	0.140496	0.724338	-1.16681	3.514691	PIGR;S100P;TGFBI
tribenoside-6328	3/100	0.049183	0.38132	0.140496	0.724338	-1.16662	3.5141	ID1;S100A4;TSPAN1
tanespimycin-2666	3/100	0.049183	0.38132	0.140496	0.724338	-1.16658	3.513985	SLC12A2;CD14;IER2
tretinoin-3165	3/100	0.049183	0.38132	0.140496	0.724338	-1.16584	3.511762	BMP4;ID3;LYZ
withaferin A-4376	3/100	0.049183	0.38132	0.140496	0.724338	-1.16224	3.500903	RNF43;TXNIP;ITM2C
yohimbine-6777	3/100	0.049183	0.38132	0.140496	0.724338	-1.16199	3.500167	RCN1;GPX2;IFI6
xylazine-4147	3/100	0.049183	0.38132	0.140496	0.724338	-1.16191	3.499911	RPS14P3;IFI6;AP1S1
tranexamic acid-2248	3/100	0.049183	0.38132	0.140496	0.724338	-1.16168	3.49922	BMP4;C3;AP1S1
tinidazole-3896	3/100	0.049183	0.38132	0.140496	0.724338	-1.15803	3.488221	RNF43;RCN1;TMPRSS3
vincamine-3976	3/100	0.049183	0.38132	0.140496	0.724338	-1.15777	3.487466	BMP4;AP1S1;ITM2C
tanespimycin-428	3/100	0.049183	0.38132	0.140496	0.724338	-1.15556	3.480791	ID1;MMP3;IER2
valproic acid-1639	3/100	0.049183	0.38132	0.140496	0.724338	-1.15527	3.479922	LGALS3BP;S100A4;SOX9
tanespimycin-505	3/100	0.049183	0.38132	0.140496	0.724338	-1.15484	3.478626	ID1;ID3;IER2
thiostrepton-4385	3/100	0.049183	0.38132	0.140496	0.724338	-1.15224	3.470806	IFITM1;IFI6;AP1S1
tetraethylenepentamine-457	3/100	0.049183	0.38132	0.140496	0.724338	-1.15122	3.467718	IGFBP2;SECTM1;PRSS23
valproic acid-410	3/100	0.049183	0.38132	0.140496	0.724338	-1.15107	3.467256	IFNGR2;CD14;CTSE
salsolidin-4810	3/100	0.049183	0.38132	0.140496	0.724338	-1.14615	3.452443	LGALS3BP;ID1;S100A6

trichostatin A-2949	3/100	0.049183	0.38132	0.140496	0.724338	-1.14446	3.447352	RCN1;ISG15;CD14
zidovudine-1595	3/100	0.049183	0.38132	0.140496	0.724338	-1.1434	3.444164	IFITM3;FOS;PABPC1
zomepirac-4479	3/100	0.049183	0.38132	0.140496	0.724338	-1.14327	3.443777	C3;IFI6;TSPAN1
thioperamide-5270	3/100	0.049183	0.38132	0.140496	0.724338	-1.1419	3.439659	RNF43;IFI27;S100A4
tanespimycin-521	3/100	0.049183	0.38132	0.140496	0.724338	-1.1416	3.438758	ID1;DPEP1;TGFBI
vigabatrin-2452	3/100	0.049183	0.38132	0.140496	0.724338	-1.13813	3.428278	GPX2;IFI6;LY6E
tretinoin-1636	3/100	0.049183	0.38132	0.140496	0.724338	-1.13324	3.413575	PDIA3;ID3;PLP2
tanespimycin-432	3/100	0.049183	0.38132	0.140496	0.724338	-1.13038	3.404941	PIGR;ID1;ID3
tetraethylenepentamine-412	3/100	0.049183	0.38132	0.140496	0.724338	-1.12153	3.378298	PIGR;GPX2;CTSE
tanespimycin-6937	3/100	0.049183	0.38132	0.140496	0.724338	-1.11653	3.363236	IFNGR2;ID1;IER2
withaferin A-4554	3/100	0.049183	0.38132	0.140496	0.724338	-1.1129	3.352309	TXNIP;CXCL1;SOX9
vitexin-4413	3/100	0.049183	0.38132	0.140496	0.724338	-1.11169	3.34864	IFI27;QPCT;IFI6
trichostatin A-6193	3/100	0.049183	0.38132	0.140496	0.724338	-1.10957	3.342273	RCN1;IFNGR2;CD14
vorinostat-2680	3/100	0.049183	0.38132	0.140496	0.724338	-1.10318	3.323018	RCN1;IFNGR2;CD14
verteporfin-6133	3/100	0.049183	0.38132	0.140496	0.724338	-1.07434	3.236146	ANXA3;SPP1;ITGAV
(-)-isoprenaline-4495	2/100	0.197972	0.467314	0.369318	0.724338	-1.1546	1.870031	IFI6;CTGF
3-hydroxy-DL-kynurenine-5276	2/100	0.197972	0.467314	0.369318	0.724338	-1.15264	1.866844	TMPRSS3;SECTM1
acetylsalicylic acid-5201	2/100	0.197972	0.467314	0.369318	0.724338	-1.1505	1.863386	BMP4;MMP3
0317956-0000-4334	2/100	0.197972	0.467314	0.369318	0.724338	-1.14996	1.862516	TGFBI;ITM2C
15(S)-15-methylprostaglandin E2-7494	2/100	0.197972	0.467314	0.369318	0.724338	-1.14967	1.862047	C3;RNF43
acebutolol-1493	2/100	0.197972	0.467314	0.369318	0.724338	-1.13864	1.844183	RNF43;TGFBI
(+)-chelidonine-2779	2/100	0.197972	0.467314	0.369318	0.724338	-1.13717	1.8418	BMP4;PGM1
2,6-dimethylpiperidine-3806	2/100	0.197972	0.467314	0.369318	0.724338	-1.13636	1.840484	TSPAN8;IFI6
0173570-0000-7391	2/100	0.197972	0.467314	0.369318	0.724338	-1.13622	1.840256	CDH3;CTSD
aciclovir-5278	2/100	0.197972	0.467314	0.369318	0.724338	-1.13421	1.837003	IFI27;IFI6
5253409-844	2/100	0.197972	0.467314	0.369318	0.724338	-1.13397	1.836607	BMP4;GPX2
3-nitropropionic acid-6402	2/100	0.197972	0.467314	0.369318	0.724338	-1.13334	1.835585	QPCT;ITM2C
aceclofenac-2117	2/100	0.197972	0.467314	0.369318	0.724338	-1.13154	1.832682	CCL20;TCN1
4-hydroxyphenazone-4175	2/100	0.197972	0.467314	0.369318	0.724338	-1.12383	1.820185	C3;TCN1
(+/-)-catechin-3351	2/100	0.197972	0.467314	0.369318	0.724338	-1.12379	1.820123	BMP4;C3
6-bromoindirubin-3'-oxime-6559	2/100	0.197972	0.467314	0.369318	0.724338	-1.12374	1.820049	CXCL1;CTGF
5279552-843	2/100	0.197972	0.467314	0.369318	0.724338	-1.12145	1.816335	BMP4;AP1S1
15(S)-15-methylprostaglandin E2-7526	2/100	0.197972	0.467314	0.369318	0.724338	-1.12065	1.815047	RNF43;TCN1
0198306-0000-7069	2/100	0.197972	0.467314	0.369318	0.724338	-1.11816	1.811003	IFI6;TSPAN1
alpha-yohimbine-5800	2/100	0.197972	0.467314	0.369318	0.724338	-1.11745	1.809853	MMP3;PSMB9
5255229-949	2/100	0.197972	0.467314	0.369318	0.724338	-1.11585	1.807261	BMP4;TMPRSS3
(+)-chelidonine-1786	2/100	0.197972	0.467314	0.369318	0.724338	-1.1135	1.803465	IFITM2;TUBA1A

4,5-dianilinophthalimide-578	2/100	0.197972	0.467314	0.369318	0.724338	-1.11174	1.800608	TMEM123;ITM2C
0198306-0000-7099	2/100	0.197972	0.467314	0.369318	0.724338	-1.1113	1.799898	TMPRSS3;MMP3
0173570-0000-3690	2/100	0.197972	0.467314	0.369318	0.724338	-1.1109	1.799243	MMP3;CTGF
aciclovir-4683	2/100	0.197972	0.467314	0.369318	0.724338	-1.10971	1.797321	OLFM4;DUOX2
aciclovir-1543	2/100	0.197972	0.467314	0.369318	0.724338	-1.10882	1.795885	TUBA1A;CTSD
0175029-0000-4713	2/100	0.197972	0.467314	0.369318	0.724338	-1.10766	1.794008	ID1;ID3
6-benzylaminopurine-2351	2/100	0.197972	0.467314	0.369318	0.724338	-1.10299	1.786438	TSPAN13;LY6E
3-acetamidocoumarin-4426	2/100	0.197972	0.467314	0.369318	0.724338	-1.10186	1.784604	IFI27;AP1S1
altretamine-4627	2/100	0.197972	0.467314	0.369318	0.724338	-1.10113	1.783432	SPINK1;OLFM4
5211181-950	2/100	0.197972	0.467314	0.369318	0.724338	-1.10075	1.78281	IFITM1;TFF3
5255229-833	2/100	0.197972	0.467314	0.369318	0.724338	-1.1004	1.782248	TMPRSS3;S100A4
adipiodone-3111	2/100	0.197972	0.467314	0.369318	0.724338	-1.09933	1.780502	ISG15;LY6E
abamectin-2519	2/100	0.197972	0.467314	0.369318	0.724338	-1.0963	1.775601	GPX2;CTSD
11-deoxy-16,16-dimethylprostaglandin E2-7538	2/100	0.197972	0.467314	0.369318	0.724338	-1.09488	1.7733	TCN1;CTGF
acetylsalicylic acid-6164	2/100	0.197972	0.467314	0.369318	0.724338	-1.09467	1.77296	CCL20;QPCT
altretamine-6467	2/100	0.197972	0.467314	0.369318	0.724338	-1.09436	1.772463	TMPRSS3;TSPAN1
alvespimycin-5573	2/100	0.197972	0.467314	0.369318	0.724338	-1.09376	1.771494	ID1;IER2
acacetin-3767	2/100	0.197972	0.467314	0.369318	0.724338	-1.09325	1.77067	BMP4;CTGF
astemizole-6807	2/100	0.197972	0.467314	0.369318	0.724338	-1.09142	1.767698	IFI27;S100A4
adiphenine-1872	2/100	0.197972	0.467314	0.369318	0.724338	-1.08719	1.760853	C3;LY6E
(+)-chelidonine-5760	2/100	0.197972	0.467314	0.369318	0.724338	-1.08689	1.760369	TUBA1A;TUBB
alimemazine-2736	2/100	0.197972	0.467314	0.369318	0.724338	-1.08668	1.760014	IGFBP2;CTSD
0173570-0000-3693	2/100	0.197972	0.467314	0.369318	0.724338	-1.08667	1.760009	MMP3;IFI6
AG-013608-6435	2/100	0.197972	0.467314	0.369318	0.724338	-1.08556	1.758212	RNF43;S100A4
16-phenyltetranorprostaglandin E2-7509	2/100	0.197972	0.467314	0.369318	0.724338	-1.08538	1.75792	IFI6;CTGF
AG-013608-5904	2/100	0.197972	0.467314	0.369318	0.724338	-1.08249	1.753235	GPX2;DUOX2
adiphenine-7037	2/100	0.197972	0.467314	0.369318	0.724338	-1.08161	1.751802	BMP4;PGM1
altretamine-3090	2/100	0.197972	0.467314	0.369318	0.724338	-1.07839	1.746601	IFI6;LY6E
alvespimycin-6933	2/100	0.197972	0.467314	0.369318	0.724338	-1.07839	1.746587	ID1;IER2
acacetin-4324	2/100	0.197972	0.467314	0.369318	0.724338	-1.07469	1.740603	TGFBI;CTGF
0317956-0000-3969	2/100	0.197972	0.467314	0.369318	0.724338	-1.07439	1.740123	AP1S1;CTGF
amphotericin B-5404	2/100	0.197972	0.467314	0.369318	0.724338	-1.074	1.739491	GPX2;IFI6
azathioprine-2028	2/100	0.197972	0.467314	0.369318	0.724338	-1.07341	1.738524	TUBA1A;GGH
acetylsalicylsalicylic acid-1377	2/100	0.197972	0.467314	0.369318	0.724338	-1.07339	1.738503	SLC12A2;PIGR
AG-012559-6920	2/100	0.197972	0.467314	0.369318	0.724338	-1.07226	1.73666	PLP2;TSPAN1
5252917-944	2/100	0.197972	0.467314	0.369318	0.724338	-1.07121	1.734966	BMP4;TSPAN1
alvespimycin-5210	2/100	0.197972	0.467314	0.369318	0.724338	-1.07115	1.734869	ID1;IER2

alimemazine-5881	2/100	0.197972	0.467314	0.369318	0.724338	-1.06917	1.731662	ID1;TSPAN1
aztreonam-5535	2/100	0.197972	0.467314	0.369318	0.724338	-1.06915	1.731624	RNF43;TSPAN1
acacetin-3849	2/100	0.197972	0.467314	0.369318	0.724338	-1.06894	1.731285	C3;MMP3
AG-013608-6400	2/100	0.197972	0.467314	0.369318	0.724338	-1.06844	1.730481	TFF2;CTGF
adenosine phosphate-5359	2/100	0.197972	0.467314	0.369318	0.724338	-1.06512	1.725098	ID1;ID3
aminophylline-3374	2/100	0.197972	0.467314	0.369318	0.724338	-1.06292	1.721546	C3;AP1S1
alpha-estradiol-1048	2/100	0.197972	0.467314	0.369318	0.724338	-1.06173	1.719604	PGM1;IER2
bacampicillin-3273	2/100	0.197972	0.467314	0.369318	0.724338	-1.06039	1.717447	PDIA3;RNF43
articaine-6517	2/100	0.197972	0.467314	0.369318	0.724338	-1.06038	1.717426	IFNGR2;TFF2
alvespimycin-4437	2/100	0.197972	0.467314	0.369318	0.724338	-1.06007	1.716924	C3;ID1
alpha-ergocryptine-3817	2/100	0.197972	0.467314	0.369318	0.724338	-1.05619	1.710637	RNF43;CXCL1
aminohippuric acid-3076	2/100	0.197972	0.467314	0.369318	0.724338	-1.0552	1.709036	ANXA3;CTSD
alpha-yohimbine-6274	2/100	0.197972	0.467314	0.369318	0.724338	-1.05346	1.706215	TFF2;S100A4
aciclovir-2044	2/100	0.197972	0.467314	0.369318	0.724338	-1.05284	1.70522	PIGR;PLP2
amoxapine-6650	2/100	0.197972	0.467314	0.369318	0.724338	-1.05231	1.704359	ID1;MMP3
alfuzosin-3203	2/100	0.197972	0.467314	0.369318	0.724338	-1.05131	1.702729	BMP4;LYZ
arecoline-6322	2/100	0.197972	0.467314	0.369318	0.724338	-1.04665	1.695195	GPX2;TSPAN1
amitriptyline-6353	2/100	0.197972	0.467314	0.369318	0.724338	-1.04654	1.695005	TSPAN8;IGFBP2
bemegride-5014	2/100	0.197972	0.467314	0.369318	0.724338	-1.04637	1.694736	RCN1;TSPAN1
azaperone-6151	2/100	0.197972	0.467314	0.369318	0.724338	-1.04633	1.694674	CCL20;CTSD
anabasine-6074	2/100	0.197972	0.467314	0.369318	0.724338	-1.04601	1.694153	SLPI;TFF2
aminophenazone-1376	2/100	0.197972	0.467314	0.369318	0.724338	-1.04551	1.693341	ITGAV;ISG15
alvespimycin-1051	2/100	0.197972	0.467314	0.369318	0.724338	-1.04379	1.690556	ID1;PSME2
allantoin-5471	2/100	0.197972	0.467314	0.369318	0.724338	-1.04378	1.690533	QPCT;IFI6
amitriptyline-1701	2/100	0.197972	0.467314	0.369318	0.724338	-1.04334	1.689834	TMPRSS3;SECTM1
amphotericin B-6303	2/100	0.197972	0.467314	0.369318	0.724338	-1.03887	1.682592	CDH3;TSPAN8
aminohippuric acid-5394	2/100	0.197972	0.467314	0.369318	0.724338	-1.03809	1.681315	IFI27;IFI6
amiodarone-3296	2/100	0.197972	0.467314	0.369318	0.724338	-1.03736	1.680142	PDIA3;BMP4
ascorbic acid-3225	2/100	0.197972	0.467314	0.369318	0.724338	-1.03592	1.677807	BMP4;LYZ
betamethasone-6728	2/100	0.197972	0.467314	0.369318	0.724338	-1.03403	1.674751	IFITM1;GPX2
apramycin-2914	2/100	0.197972	0.467314	0.369318	0.724338	-1.03363	1.674098	TCN1;ITGAV
amylocaine-4169	2/100	0.197972	0.467314	0.369318	0.724338	-1.03361	1.674062	SPINK1;AP1S1
benzonatate-5435	2/100	0.197972	0.467314	0.369318	0.724338	-1.03287	1.67287	GPX2;IFI6
apigenin-3257	2/100	0.197972	0.467314	0.369318	0.724338	-1.03285	1.672844	ID1;ATP1B1
benfotiamine-4312	2/100	0.197972	0.467314	0.369318	0.724338	-1.03242	1.672143	GPX2;IFI6
benzylpenicillin-6839	2/100	0.197972	0.467314	0.369318	0.724338	-1.03169	1.670961	IFI27;IFI6
bacampicillin-4417	2/100	0.197972	0.467314	0.369318	0.724338	-1.03037	1.668825	IFI6;AP1S1

antimycin A-1414	2/100	0.197972	0.467314	0.369318	0.724338	-1.03033	1.668758	TXNIP;DYNLL1
atovaquone-2480	2/100	0.197972	0.467314	0.369318	0.724338	-1.02966	1.667675	SPP1;TXNIP
bacitracin-3109	2/100	0.197972	0.467314	0.369318	0.724338	-1.02792	1.664858	IFITM3;IFI6
amphotericin B-2441	2/100	0.197972	0.467314	0.369318	0.724338	-1.02784	1.664723	IFI6;ATP1B1
bicuculline-4397	2/100	0.197972	0.467314	0.369318	0.724338	-1.02775	1.664577	IFI27;S100A4
alpha-estradiol-702	2/100	0.197972	0.467314	0.369318	0.724338	-1.02636	1.66233	IFI6;ID3
allantoin-1842	2/100	0.197972	0.467314	0.369318	0.724338	-1.02587	1.661536	IFITM3;LY6E
adenosine phosphate-1622	2/100	0.197972	0.467314	0.369318	0.724338	-1.02235	1.655822	IFITM3;HSPB1
ampyrone-1402	2/100	0.197972	0.467314	0.369318	0.724338	-1.02206	1.655352	IFITM3;ISG15
alvespimycin-1638	2/100	0.197972	0.467314	0.369318	0.724338	-1.0218	1.654933	ID1;IER2
arachidonic acid-441	2/100	0.197972	0.467314	0.369318	0.724338	-1.01887	1.6502	MMP7;TCN1
biotin-2428	2/100	0.197972	0.467314	0.369318	0.724338	-1.01785	1.648536	GPX2;CKB
albendazole-1547	2/100	0.197972	0.467314	0.369318	0.724338	-1.01718	1.647465	TUBA1A;MEST
calcium folinate-2579	2/100	0.197972	0.467314	0.369318	0.724338	-1.01502	1.643954	TSPAN13;IGFBP2
alpha-ergocryptine-3900	2/100	0.197972	0.467314	0.369318	0.724338	-1.01478	1.643568	SECTM1;ITM2C
alverine-1426	2/100	0.197972	0.467314	0.369318	0.724338	-1.01345	1.641418	RPS14P3;TMEM123
BCB000039-7510	2/100	0.197972	0.467314	0.369318	0.724338	-1.01067	1.636908	TSPAN1;CTGF
atropine oxide-1370	2/100	0.197972	0.467314	0.369318	0.724338	-1.00898	1.634176	IFITM3;SLC12A2
bephenium hydroxynaphthoate-5263	2/100	0.197972	0.467314	0.369318	0.724338	-1.00827	1.633032	IFI27;IFI6
bretylium tosilate-6674	2/100	0.197972	0.467314	0.369318	0.724338	-1.0075	1.63178	TGFBI;PSMB9
betulinic acid-4101	2/100	0.197972	0.467314	0.369318	0.724338	-1.00663	1.630377	IFI6;AP1S1
carbarsone-4110	2/100	0.197972	0.467314	0.369318	0.724338	-1.00562	1.628734	IFI6;S100A4
benzthiazide-4952	2/100	0.197972	0.467314	0.369318	0.724338	-1.00356	1.6254	IFI27;TSPAN1
benserazide-6482	2/100	0.197972	0.467314	0.369318	0.724338	-1.00348	1.625272	QPCT;SECTM1
azacyclonol-2020	2/100	0.197972	0.467314	0.369318	0.724338	-1.00216	1.623127	IFITM3;C3
alvespimycin-993	2/100	0.197972	0.467314	0.369318	0.724338	-1.00128	1.621704	ID1;IER2
alsterpaullone-7078	2/100	0.197972	0.467314	0.369318	0.724338	-1.0012	1.621573	ID3;CTGF
ambroxol-1623	2/100	0.197972	0.467314	0.369318	0.724338	-1.00115	1.621487	IFITM3;RPS14P3
atractyloside-2573	2/100	0.197972	0.467314	0.369318	0.724338	-0.99832	1.616917	IGFBP2;OLFM4
amprolium-1979	2/100	0.197972	0.467314	0.369318	0.724338	-0.99824	1.616787	ISG15;LYZ
benperidol-4196	2/100	0.197972	0.467314	0.369318	0.724338	-0.99703	1.614814	GPX2;TGFBI
bromopride-3719	2/100	0.197972	0.467314	0.369318	0.724338	-0.99604	1.613216	RNF43;PSMB9
betaxolol-4608	2/100	0.197972	0.467314	0.369318	0.724338	-0.99561	1.61252	IGFBP2;AP1S1
betamethasone-5328	2/100	0.197972	0.467314	0.369318	0.724338	-0.9956	1.612498	IFI6;ITGAV
baclofen-5414	2/100	0.197972	0.467314	0.369318	0.724338	-0.99444	1.610627	IFI27;QPCT
benzydamine-3169	2/100	0.197972	0.467314	0.369318	0.724338	-0.99337	1.608893	LGALS3BP;LYZ
cefixime-3247	2/100	0.197972	0.467314	0.369318	0.724338	-0.98993	1.603325	PDIA3;SECTM1

benserazide-6722	2/100	0.197972	0.467314	0.369318	0.724338	-0.98984	1.603171	LGALS3BP;ID1
aztreonam-1435	2/100	0.197972	0.467314	0.369318	0.724338	-0.98895	1.601735	RPS14P3;TUBA1A
carbamazepine-1847	2/100	0.197972	0.467314	0.369318	0.724338	-0.98878	1.601456	IFITM3;C3
bufexamac-5090	2/100	0.197972	0.467314	0.369318	0.724338	-0.98659	1.597911	IFI6;CTGF
bupivacaine-2404	2/100	0.197972	0.467314	0.369318	0.724338	-0.98593	1.596836	IFITM3;SPP1
BCB000038-7547	2/100	0.197972	0.467314	0.369318	0.724338	-0.9843	1.594197	BMP4;GPX2
BCB000038-7542	2/100	0.197972	0.467314	0.369318	0.724338	-0.98263	1.591495	IFI6;S100A4
bupivacaine-7435	2/100	0.197972	0.467314	0.369318	0.724338	-0.98062	1.588243	PDIA3;AP1S1
benzethonium chloride-2508	2/100	0.197972	0.467314	0.369318	0.724338	-0.98059	1.588199	C3;CCL20
boldine-4004	2/100	0.197972	0.467314	0.369318	0.724338	-0.98025	1.587638	IFI6;S100A4
calcium pantothenate-4775	2/100	0.197972	0.467314	0.369318	0.724338	-0.97936	1.586202	QPCT;IFI6
amprolium-4825	2/100	0.197972	0.467314	0.369318	0.724338	-0.97825	1.584399	GPX2;CKB
betazole-1812	2/100	0.197972	0.467314	0.369318	0.724338	-0.97632	1.581271	IL32;LGALS3BP
butein-607	2/100	0.197972	0.467314	0.369318	0.724338	-0.97396	1.577454	TCN1;ID3
benzamil-3635	2/100	0.197972	0.467314	0.369318	0.724338	-0.9739	1.577359	QPCT;TFF2
boldine-2148	2/100	0.197972	0.467314	0.369318	0.724338	-0.97373	1.577085	TCN1;TFF2
baclofen-2036	2/100	0.197972	0.467314	0.369318	0.724338	-0.9735	1.576705	PDIA3;PLP2
carbenoxolone-4093	2/100	0.197972	0.467314	0.369318	0.724338	-0.97217	1.574565	LUM;IFI6
bendroflumethiazide-2555	2/100	0.197972	0.467314	0.369318	0.724338	-0.97193	1.574175	IFITM3;CKB
amylocaine-1991	2/100	0.197972	0.467314	0.369318	0.724338	-0.97151	1.573485	IFITM3;IFITM2
bromopride-3617	2/100	0.197972	0.467314	0.369318	0.724338	-0.97107	1.572773	RPS14P3;SLPI
cefotiam-5361	2/100	0.197972	0.467314	0.369318	0.724338	-0.96939	1.570048	IFI6;IER2
budesonide-2866	2/100	0.197972	0.467314	0.369318	0.724338	-0.96619	1.564878	PDIA3;C3
baclofen-1952	2/100	0.197972	0.467314	0.369318	0.724338	-0.96563	1.563966	TMEM123;ITGAV
bemegride-3051	2/100	0.197972	0.467314	0.369318	0.724338	-0.96369	1.560818	CCL20;IGFBP2
betaxolol-1592	2/100	0.197972	0.467314	0.369318	0.724338	-0.95986	1.554619	IFNGR2;IFI6
ceforanide-2447	2/100	0.197972	0.467314	0.369318	0.724338	-0.95708	1.550113	GPX2;IFI6
butamben-6266	2/100	0.197972	0.467314	0.369318	0.724338	-0.95628	1.548829	TFF2;S100A4
biperiden-2460	2/100	0.197972	0.467314	0.369318	0.724338	-0.95614	1.548599	GPX2;IFI6
calcium pantothenate-3248	2/100	0.197972	0.467314	0.369318	0.724338	-0.95532	1.547262	ISG15;PGM1
carbimazole-3299	2/100	0.197972	0.467314	0.369318	0.724338	-0.95495	1.546662	TMPRSS3;TFF2
chloropyramine-4414	2/100	0.197972	0.467314	0.369318	0.724338	-0.95464	1.546171	IFI27;IFI6
carbachol-5342	2/100	0.197972	0.467314	0.369318	0.724338	-0.95423	1.545509	TFF3;IFI6
chlorpromazine-5214	2/100	0.197972	0.467314	0.369318	0.724338	-0.95342	1.544184	GPX2;AP1S1
canadine-4020	2/100	0.197972	0.467314	0.369318	0.724338	-0.95234	1.542446	MMP3;S100A6
ceforanide-3309	2/100	0.197972	0.467314	0.369318	0.724338	-0.95226	1.542311	BMP4;TFF2
cefaclor-4967	2/100	0.197972	0.467314	0.369318	0.724338	-0.95109	1.540423	IFI27;S100A4

beta-escin-3890	2/100	0.197972	0.467314	0.369318	0.724338	-0.94886	1.536801	RCN1;GPX2
cefalexin-5615	2/100	0.197972	0.467314	0.369318	0.724338	-0.94643	1.532865	TMPRSS3;TFF2
cinnarizine-3175	2/100	0.197972	0.467314	0.369318	0.724338	-0.94425	1.529329	BMP4;LYZ
canadine-2163	2/100	0.197972	0.467314	0.369318	0.724338	-0.94264	1.526732	GPX2;TFF2
bufexamac-5515	2/100	0.197972	0.467314	0.369318	0.724338	-0.94079	1.523735	IFNGR2;TMPRSS3
budesonide-1716	2/100	0.197972	0.467314	0.369318	0.724338	-0.93771	1.51874	ANXA3;DYNLL1
chlorpromazine-1158	2/100	0.197972	0.467314	0.369318	0.724338	-0.93697	1.517553	IFITM3;IFI6
chlorcyclizine-2197	2/100	0.197972	0.467314	0.369318	0.724338	-0.93666	1.51704	IFI6;LY6E
capsaicin-3372	2/100	0.197972	0.467314	0.369318	0.724338	-0.93587	1.51576	BMP4;AP1S1
chloropyrazine-6148	2/100	0.197972	0.467314	0.369318	0.724338	-0.93363	1.512139	CCL20;TCN1
cefmetazole-6086	2/100	0.197972	0.467314	0.369318	0.724338	-0.93347	1.51187	SLPI;TFF2
chlormezanone-5697	2/100	0.197972	0.467314	0.369318	0.724338	-0.93199	1.509487	GPX2;QPCT
carcinine-4809	2/100	0.197972	0.467314	0.369318	0.724338	-0.93171	1.509028	ID1;TSPAN1
betazole-1854	2/100	0.197972	0.467314	0.369318	0.724338	-0.9301	1.50642	C3;ANXA3
cefalexin-2628	2/100	0.197972	0.467314	0.369318	0.724338	-0.92986	1.506037	BMP4;S100A4
capsaicin-5673	2/100	0.197972	0.467314	0.369318	0.724338	-0.92965	1.505683	BMP4;TMPRSS3
chlortetracycline-5360	2/100	0.197972	0.467314	0.369318	0.724338	-0.92962	1.505644	TUBA1A;IFI6
chlorphenesin-1432	2/100	0.197972	0.467314	0.369318	0.724338	-0.92958	1.505573	RPS14P3;TUBA1A
cetirizine-4815	2/100	0.197972	0.467314	0.369318	0.724338	-0.92807	1.503138	S100A4;CTSD
clidinium bromide-6837	2/100	0.197972	0.467314	0.369318	0.724338	-0.92682	1.501112	IFI27;IFI6
carbachol-3380	2/100	0.197972	0.467314	0.369318	0.724338	-0.92545	1.498888	SLPI;SECTM1
calcium pantothenate-1311	2/100	0.197972	0.467314	0.369318	0.724338	-0.92481	1.497846	IFITM3;C3
clenbuterol-4671	2/100	0.197972	0.467314	0.369318	0.724338	-0.92458	1.497471	IL32;CTGF
cefotiam-2458	2/100	0.197972	0.467314	0.369318	0.724338	-0.924	1.496545	GPX2;MMP3
cefadroxil-4161	2/100	0.197972	0.467314	0.369318	0.724338	-0.92374	1.496124	MMP3;AP1S1
carbamazepine-1683	2/100	0.197972	0.467314	0.369318	0.724338	-0.92291	1.494781	TMPRSS3;S100A4
clorgiline-3219	2/100	0.197972	0.467314	0.369318	0.724338	-0.92237	1.493895	SECTM1;LYZ
cefepime-6159	2/100	0.197972	0.467314	0.369318	0.724338	-0.92193	1.493193	GPX2;CCL20
carmustine-6914	2/100	0.197972	0.467314	0.369318	0.724338	-0.92165	1.492738	PLP2;TSPAN1
bufexamac-2382	2/100	0.197972	0.467314	0.369318	0.724338	-0.92115	1.491929	TSPAN13;SLPI
chlorpromazine-6176	2/100	0.197972	0.467314	0.369318	0.724338	-0.92091	1.491529	TSPAN13;CTSD
cefalotin-6819	2/100	0.197972	0.467314	0.369318	0.724338	-0.91986	1.489838	IFI27;IFI6
cinchonidine-7190	2/100	0.197972	0.467314	0.369318	0.724338	-0.91887	1.488233	LGALS3BP;TSPAN1
biperiden-5644	2/100	0.197972	0.467314	0.369318	0.724338	-0.91815	1.487068	C3;RCN1
chlorambucil-4345	2/100	0.197972	0.467314	0.369318	0.724338	-0.9178	1.486499	BMP4;IFI27
cinchocaine-4149	2/100	0.197972	0.467314	0.369318	0.724338	-0.91777	1.486446	BMP4;AP1S1
chlorhexidine-1525	2/100	0.197972	0.467314	0.369318	0.724338	-0.91648	1.484362	BMP4;TFF2

clorgiline-6659	2/100	0.197972	0.467314	0.369318	0.724338	-0.91496	1.48189	C3;TGFBI
brompheniramine-4131	2/100	0.197972	0.467314	0.369318	0.724338	-0.91469	1.481464	TCN1;MMP3
clioquinol-5258	2/100	0.197972	0.467314	0.369318	0.724338	-0.91381	1.480043	BMP4;IFI27
clenbuterol-5631	2/100	0.197972	0.467314	0.369318	0.724338	-0.91338	1.479332	QPCT;TFF2
bromocriptine-2007	2/100	0.197972	0.467314	0.369318	0.724338	-0.91317	1.479002	SPP1;HSPB1
chlormezanone-3235	2/100	0.197972	0.467314	0.369318	0.724338	-0.912	1.4771	SECTM1;LYZ
clofazimine-3239	2/100	0.197972	0.467314	0.369318	0.724338	-0.91108	1.475613	SECTM1;LYZ
ciclopirox-3317	2/100	0.197972	0.467314	0.369318	0.724338	-0.91083	1.475206	RNF43;CKS2
clonidine-3172	2/100	0.197972	0.467314	0.369318	0.724338	-0.9084	1.471272	GPX2;LYZ
clindamycin-2219	2/100	0.197972	0.467314	0.369318	0.724338	-0.90804	1.470698	AP1S1;TFF2
chlorcyclizine-3893	2/100	0.197972	0.467314	0.369318	0.724338	-0.90699	1.46899	GPX2;SECTM1
chlortalidone-6800	2/100	0.197972	0.467314	0.369318	0.724338	-0.90697	1.468949	IFI27;IFI6
clozapine-1229	2/100	0.197972	0.467314	0.369318	0.724338	-0.90598	1.467353	TSPAN8;PGM1
citiolone-3930	2/100	0.197972	0.467314	0.369318	0.724338	-0.90585	1.467136	IFI6;TFF2
chlorpromazine-419	2/100	0.197972	0.467314	0.369318	0.724338	-0.90565	1.466824	GPX2;ID3
citiolone-4311	2/100	0.197972	0.467314	0.369318	0.724338	-0.90386	1.463928	RPS14P3;IFI6
co-dergocrine mesilate-4152	2/100	0.197972	0.467314	0.369318	0.724338	-0.90363	1.463554	BMP4;AP1S1
chloramphenicol-1837	2/100	0.197972	0.467314	0.369318	0.724338	-0.90179	1.460561	IFITM3;CCL20
carteolol-4176	2/100	0.197972	0.467314	0.369318	0.724338	-0.8978	1.454104	AP1S1;TFF2
clopamide-3220	2/100	0.197972	0.467314	0.369318	0.724338	-0.89721	1.453156	SECTM1;LYZ
coralyne-5418	2/100	0.197972	0.467314	0.369318	0.724338	-0.89708	1.452942	IFI27;IFI6
ciclopirox-5023	2/100	0.197972	0.467314	0.369318	0.724338	-0.897	1.452817	IFI27;CKS2
chloropyrazine-6227	2/100	0.197972	0.467314	0.369318	0.724338	-0.89439	1.448578	LGALS3BP;IFNGR2
clopamide-5402	2/100	0.197972	0.467314	0.369318	0.724338	-0.89387	1.447743	IFI27;IFI6
cloperastine-4271	2/100	0.197972	0.467314	0.369318	0.724338	-0.88943	1.440548	IFI6;S100A4
cefotaxime-2072	2/100	0.197972	0.467314	0.369318	0.724338	-0.88914	1.440072	IL32;IGFBP2
chlorpropamide-3210	2/100	0.197972	0.467314	0.369318	0.724338	-0.88863	1.439252	BMP4;LYZ
clemizole-2301	2/100	0.197972	0.467314	0.369318	0.724338	-0.8881	1.438398	GPX2;TFF2
clofazimine-4682	2/100	0.197972	0.467314	0.369318	0.724338	-0.88805	1.438317	TXNIP;OLFM4
colchicine-5675	2/100	0.197972	0.467314	0.369318	0.724338	-0.88751	1.437438	BMP4;TMPRSS3
chlorpromazine-426	2/100	0.197972	0.467314	0.369318	0.724338	-0.88265	1.42956	CDH3;MMP3
clozapine-1170	2/100	0.197972	0.467314	0.369318	0.724338	-0.87911	1.423826	TSPAN13;PGM1
clotrimazole-3166	2/100	0.197972	0.467314	0.369318	0.724338	-0.87674	1.419993	GPX2;LYZ
clorsulon-7025	2/100	0.197972	0.467314	0.369318	0.724338	-0.87633	1.419325	IFITM1;TSPAN1
chlorpromazine-2677	2/100	0.197972	0.467314	0.369318	0.724338	-0.87573	1.418361	ANXA3;CKB
chlorpropamide-1594	2/100	0.197972	0.467314	0.369318	0.724338	-0.87488	1.416978	SLC12A2;IFI6
cyproterone-3545	2/100	0.197972	0.467314	0.369318	0.724338	-0.87343	1.414641	LGALS3BP;S100A6

cyclizine-5525	2/100	0.197972	0.467314	0.369318	0.724338	-0.87325	1.414348	LGALS3BP;QPCT
clomipramine-6825	2/100	0.197972	0.467314	0.369318	0.724338	-0.87284	1.413675	IFI27;IFI6
cyproterone-6806	2/100	0.197972	0.467314	0.369318	0.724338	-0.87188	1.412125	BMP4;IFI6
corbadrine-5854	2/100	0.197972	0.467314	0.369318	0.724338	-0.87026	1.409497	MMP1;CTGF
dacarbazine-6816	2/100	0.197972	0.467314	0.369318	0.724338	-0.87009	1.409227	IFI27;IFI6
conessine-4191	2/100	0.197972	0.467314	0.369318	0.724338	-0.86548	1.40175	GPX2;TGFBI
chlorpropamide-6291	2/100	0.197972	0.467314	0.369318	0.724338	-0.86227	1.396561	CDH3;CTSD
clenbuterol-3228	2/100	0.197972	0.467314	0.369318	0.724338	-0.8615	1.395315	SECTM1;LYZ
demeclocycline-3604	2/100	0.197972	0.467314	0.369318	0.724338	-0.85963	1.392282	SLPI;S100A4
demeclocycline-4267	2/100	0.197972	0.467314	0.369318	0.724338	-0.85923	1.391635	RPS14P3;S100A4
dequalinium chloride-5396	2/100	0.197972	0.467314	0.369318	0.724338	-0.85887	1.39105	IFI27;ISG15
clozapine-1009	2/100	0.197972	0.467314	0.369318	0.724338	-0.8575	1.388838	BMP4;TSPAN1
CP-690334-01-3826	2/100	0.197972	0.467314	0.369318	0.724338	-0.85639	1.387031	IL32;FOS
clidinium bromide-4499	2/100	0.197972	0.467314	0.369318	0.724338	-0.85405	1.383254	PDIA3;RNF43
corticosterone-4145	2/100	0.197972	0.467314	0.369318	0.724338	-0.85291	1.381398	C3;AP1S1
dexpropranolol-6130	2/100	0.197972	0.467314	0.369318	0.724338	-0.85245	1.380659	PDIA3;ANXA3
clofazimine-5277	2/100	0.197972	0.467314	0.369318	0.724338	-0.85142	1.378991	MMP3;TXNIP
conessine-4777	2/100	0.197972	0.467314	0.369318	0.724338	-0.85133	1.378836	CDH3;IFI6
colecalciferol-5002	2/100	0.197972	0.467314	0.369318	0.724338	-0.84553	1.369452	SLPI;S100A4
decitabine-920	2/100	0.197972	0.467314	0.369318	0.724338	-0.84328	1.365796	RNF43;IGFBP2
CP-320650-01-3908	2/100	0.197972	0.467314	0.369318	0.724338	-0.8423	1.364211	LGALS3BP;ITGAV
cyclopenthiazide-2905	2/100	0.197972	0.467314	0.369318	0.724338	-0.84226	1.364153	TSPAN13;IFI6
crotamiton-4628	2/100	0.197972	0.467314	0.369318	0.724338	-0.84128	1.362563	IL32;SPINK1
diethylstilbestrol-4547	2/100	0.197972	0.467314	0.369318	0.724338	-0.84053	1.361341	C3;CDH3
diphenylpyraline-3743	2/100	0.197972	0.467314	0.369318	0.724338	-0.84	1.360494	C3;PSMB9
clozapine-5265	2/100	0.197972	0.467314	0.369318	0.724338	-0.83914	1.359091	IFI27;IFI6
demeclocycline-2545	2/100	0.197972	0.467314	0.369318	0.724338	-0.83819	1.357566	SLC12A2;TSPAN13
cortisone-7458	2/100	0.197972	0.467314	0.369318	0.724338	-0.83714	1.355855	TMPRSS3;TSPAN1
dacarbazine-2754	2/100	0.197972	0.467314	0.369318	0.724338	-0.83689	1.355457	BMP4;SOX9
cyproheptadine-2021	2/100	0.197972	0.467314	0.369318	0.724338	-0.83675	1.355229	GGH;LYZ
colchicine-630	2/100	0.197972	0.467314	0.369318	0.724338	-0.83659	1.35497	TUBA1A;TUBB
cytisine-1766	2/100	0.197972	0.467314	0.369318	0.724338	-0.83443	1.351466	C3;CCL20
CP-645525-01-7522	2/100	0.197972	0.467314	0.369318	0.724338	-0.83337	1.349744	SLPI;ITM2C
diltiazem-6710	2/100	0.197972	0.467314	0.369318	0.724338	-0.83198	1.347501	LGALS3BP;SLPI
CP-944629-7549	2/100	0.197972	0.467314	0.369318	0.724338	-0.83185	1.347283	BMP4;TSPAN1
dapsone-5078	2/100	0.197972	0.467314	0.369318	0.724338	-0.82952	1.343513	TSPAN8;IFI6
diphenhydramine-1708	2/100	0.197972	0.467314	0.369318	0.724338	-0.82532	1.336715	SECTM1;ETS2

cotinine-1929	2/100	0.197972	0.467314	0.369318	0.724338	-0.82439	1.335213	TMEM123;PRSS23
debrisoquine-3207	2/100	0.197972	0.467314	0.369318	0.724338	-0.82365	1.334004	SECTM1;LYZ
dicycloverine-4581	2/100	0.197972	0.467314	0.369318	0.724338	-0.82354	1.333839	C3;TSPAN1
ebselen-3458	2/100	0.197972	0.467314	0.369318	0.724338	-0.82271	1.332492	TMPRSS3;PGM1
CP-319743-7537	2/100	0.197972	0.467314	0.369318	0.724338	-0.82262	1.332338	MMP1;CTGF
CP-319743-7491	2/100	0.197972	0.467314	0.369318	0.724338	-0.82259	1.332289	RNF43;CTGF
dienestrol-3448	2/100	0.197972	0.467314	0.369318	0.724338	-0.82124	1.330106	ID3;TGFBI
debrisoquine-5288	2/100	0.197972	0.467314	0.369318	0.724338	-0.81997	1.328053	IFI6;ISG15
epirizole-1803	2/100	0.197972	0.467314	0.369318	0.724338	-0.81913	1.326684	TUBA1C;IFI6
demecarium bromide-2773	2/100	0.197972	0.467314	0.369318	0.724338	-0.81908	1.326615	BMP4;RNF43
CP-320650-01-3825	2/100	0.197972	0.467314	0.369318	0.724338	-0.81899	1.326461	SLCO5A1;FOS
dicycloverine-1483	2/100	0.197972	0.467314	0.369318	0.724338	-0.81891	1.326339	BMP4;RNF43
diperodon-6836	2/100	0.197972	0.467314	0.369318	0.724338	-0.81857	1.32578	IFI27;IFI6
clorgiline-1604	2/100	0.197972	0.467314	0.369318	0.724338	-0.81745	1.323968	IFITM3;FOS
colforsin-7104	2/100	0.197972	0.467314	0.369318	0.724338	-0.81685	1.323004	MMP1;CTGF
droperidol-2645	2/100	0.197972	0.467314	0.369318	0.724338	-0.81632	1.322135	BMP4;ETS2
denatonium benzoate-3123	2/100	0.197972	0.467314	0.369318	0.724338	-0.81553	1.320858	TCN1;IFI6
dinoprostone-6590	2/100	0.197972	0.467314	0.369318	0.724338	-0.81275	1.316361	TGFBI;TSPAN1
etiocholanolone-4764	2/100	0.197972	0.467314	0.369318	0.724338	-0.81234	1.315699	IFI27;IFI6
dequalinium chloride-2631	2/100	0.197972	0.467314	0.369318	0.724338	-0.81202	1.315178	TMPRSS3;S100A4
CP-944629-7497	2/100	0.197972	0.467314	0.369318	0.724338	-0.81182	1.314855	SPINK1;SLPI
cyproheptadine-5340	2/100	0.197972	0.467314	0.369318	0.724338	-0.81138	1.31413	IFITM1;IFI6
dinoprostone-6547	2/100	0.197972	0.467314	0.369318	0.724338	-0.80587	1.305205	MMP1;CTGF
epiandrosterone-2444	2/100	0.197972	0.467314	0.369318	0.724338	-0.8058	1.3051	IFI6;LY6E
dicloxacillin-2445	2/100	0.197972	0.467314	0.369318	0.724338	-0.80547	1.304559	ITGAV;FOS
diflunisal-1990	2/100	0.197972	0.467314	0.369318	0.724338	-0.80483	1.303531	ISG15;ITM2C
dobutamine-3206	2/100	0.197972	0.467314	0.369318	0.724338	-0.80468	1.303292	BMP4;LYZ
dinoprostone-6586	2/100	0.197972	0.467314	0.369318	0.724338	-0.80398	1.302148	C3;CTGF
epirizole-5995	2/100	0.197972	0.467314	0.369318	0.724338	-0.80166	1.298399	AP1S1;S100A4
dilazep-2333	2/100	0.197972	0.467314	0.369318	0.724338	-0.80166	1.298391	TSPAN13;SPP1
econazole-6008	2/100	0.197972	0.467314	0.369318	0.724338	-0.80098	1.297296	RNF43;IFI6
dipyridamole-5252	2/100	0.197972	0.467314	0.369318	0.724338	-0.80092	1.297197	IFI6;TSPAN1
dosulepin-1713	2/100	0.197972	0.467314	0.369318	0.724338	-0.79873	1.293646	GPX2;ISG15
dilazep-3665	2/100	0.197972	0.467314	0.369318	0.724338	-0.79732	1.291362	MMP3;CXCL1
diphenhydramine-1871	2/100	0.197972	0.467314	0.369318	0.724338	-0.79509	1.28775	ANXA3;LY6E
felbinac-4639	2/100	0.197972	0.467314	0.369318	0.724338	-0.7949	1.287448	TGFBI;OLFM4
dinoprost-3308	2/100	0.197972	0.467314	0.369318	0.724338	-0.79369	1.285492	RNF43;SECTM1

dipivefrine-6766	2/100	0.197972	0.467314	0.369318	0.724338	-0.79321	1.284701	S100A6;IFI6
etodolac-2091	2/100	0.197972	0.467314	0.369318	0.724338	-0.78873	1.277445	IL32;TCN1
dicloxacillin-6666	2/100	0.197972	0.467314	0.369318	0.724338	-0.78851	1.277099	MMP3;TGFBI
dilazep-7364	2/100	0.197972	0.467314	0.369318	0.724338	-0.78849	1.277064	IGFBP2;CXCL1
desoxycortone-5357	2/100	0.197972	0.467314	0.369318	0.724338	-0.78835	1.276838	ID1;ISG15
dinoprostone-6552	2/100	0.197972	0.467314	0.369318	0.724338	-0.78704	1.27471	IFI27;TSPAN1
enoxacin-5251	2/100	0.197972	0.467314	0.369318	0.724338	-0.78296	1.268109	RNF43;SLPI
dropropizine-7429	2/100	0.197972	0.467314	0.369318	0.724338	-0.78272	1.267723	S100A4;TGFBI
enilconazole-6518	2/100	0.197972	0.467314	0.369318	0.724338	-0.77555	1.25611	GPX2;TFF2
etamsylate-2915	2/100	0.197972	0.467314	0.369318	0.724338	-0.77507	1.255327	QPCT;ISG15
eldeline-3925	2/100	0.197972	0.467314	0.369318	0.724338	-0.77443	1.254292	IFI6;AP1S1
emetine-2801	2/100	0.197972	0.467314	0.369318	0.724338	-0.77433	1.254124	RPS14P3;TMEM123
estradiol-1208	2/100	0.197972	0.467314	0.369318	0.724338	-0.7724	1.25101	IL32;CCL20
esculin-5411	2/100	0.197972	0.467314	0.369318	0.724338	-0.77216	1.250615	IFI27;S100A4
ellipticine-6253	2/100	0.197972	0.467314	0.369318	0.724338	-0.7712	1.249068	ID3;IER2
estradiol-6928	2/100	0.197972	0.467314	0.369318	0.724338	-0.7702	1.24744	LGALS3BP;IGFBP2
demecolcine-1103	2/100	0.197972	0.467314	0.369318	0.724338	-0.7698	1.246786	IFITM2;LY6E
fluoxetine-5356	2/100	0.197972	0.467314	0.369318	0.724338	-0.76901	1.245516	ID1;IFI6
econazole-2396	2/100	0.197972	0.467314	0.369318	0.724338	-0.76838	1.244498	TSPAN13;CKB
fenbufen-2308	2/100	0.197972	0.467314	0.369318	0.724338	-0.76598	1.240601	C3;S100A6
flavoxate-6326	2/100	0.197972	0.467314	0.369318	0.724338	-0.76551	1.239844	PDIA3;IFITM1
fenbufen-4743	2/100	0.197972	0.467314	0.369318	0.724338	-0.76359	1.236733	PDIA3;GPX2
estradiol-6200	2/100	0.197972	0.467314	0.369318	0.724338	-0.763	1.235782	CCL20;MMP3
equilin-5255	2/100	0.197972	0.467314	0.369318	0.724338	-0.76077	1.232173	IFI27;IFI6
diflorasone-2142	2/100	0.197972	0.467314	0.369318	0.724338	-0.75904	1.229366	CD14;CKB
etilefrine-7350	2/100	0.197972	0.467314	0.369318	0.724338	-0.75811	1.227862	TMPRSS3;IFI6
etoposide-3241	2/100	0.197972	0.467314	0.369318	0.724338	-0.75711	1.226237	BMP4;LYZ
estradiol-1021	2/100	0.197972	0.467314	0.369318	0.724338	-0.75602	1.224474	BMP4;ID1
fenbendazole-2360	2/100	0.197972	0.467314	0.369318	0.724338	-0.75523	1.2232	PLCB4;TUBB
ethoxyquin-4321	2/100	0.197972	0.467314	0.369318	0.724338	-0.75423	1.221577	C3;IFI6
ethaverine-5337	2/100	0.197972	0.467314	0.369318	0.724338	-0.75423	1.221569	IFI6;TXNIP
etoposide-6681	2/100	0.197972	0.467314	0.369318	0.724338	-0.75357	1.220502	IFI6;TGFBI
felodipine-848	2/100	0.197972	0.467314	0.369318	0.724338	-0.75183	1.217685	BMP4;AP1S1
estradiol-7000	2/100	0.197972	0.467314	0.369318	0.724338	-0.75144	1.217052	IFNGR2;ID1
emetine-4827	2/100	0.197972	0.467314	0.369318	0.724338	-0.75056	1.215628	TMEM123;GPX2
estradiol-6957	2/100	0.197972	0.467314	0.369318	0.724338	-0.75002	1.214754	RPS14P3;S100A4
dipivefrine-1752	2/100	0.197972	0.467314	0.369318	0.724338	-0.74761	1.210856	IFITM2;SPP1

etifenin-2838	2/100	0.197972	0.467314	0.369318	0.724338	-0.74379	1.204668	IGFBP2;ID3
ellipticine-2758	2/100	0.197972	0.467314	0.369318	0.724338	-0.74173	1.201336	ID3;IER2
exisulind-309	2/100	0.197972	0.467314	0.369318	0.724338	-0.74136	1.200737	LCN2;DSG2
dobutamine-1589	2/100	0.197972	0.467314	0.369318	0.724338	-0.74081	1.199842	IFITM3;HSPB1
doxorubicin-3291	2/100	0.197972	0.467314	0.369318	0.724338	-0.73981	1.198227	ID3;IER2
flunixin-4273	2/100	0.197972	0.467314	0.369318	0.724338	-0.73795	1.195211	RPS14P3;IFI6
etidronic acid-4387	2/100	0.197972	0.467314	0.369318	0.724338	-0.73519	1.190743	C3;AP1S1
estriol-6140	2/100	0.197972	0.467314	0.369318	0.724338	-0.73462	1.189808	TSPAN13;ANXA3
fenbendazole-4542	2/100	0.197972	0.467314	0.369318	0.724338	-0.73284	1.186927	TUBB;TGFBI
estradiol-1299	2/100	0.197972	0.467314	0.369318	0.724338	-0.73136	1.18453	C3;S100A6
flunixin-4735	2/100	0.197972	0.467314	0.369318	0.724338	-0.7308	1.183632	PDIA3;IFI6
furaltadone-3932	2/100	0.197972	0.467314	0.369318	0.724338	-0.72959	1.18166	QPCT;IFI6
flumetasone-4272	2/100	0.197972	0.467314	0.369318	0.724338	-0.72868	1.180192	RPS14P3;IFI6
dyclonine-2392	2/100	0.197972	0.467314	0.369318	0.724338	-0.72845	1.179823	C3;CKB
fursultiamine-4975	2/100	0.197972	0.467314	0.369318	0.724338	-0.72746	1.178223	IFI27;IFI6
felodipine-3295	2/100	0.197972	0.467314	0.369318	0.724338	-0.72375	1.172206	BMP4;C3
fenspiride-2269	2/100	0.197972	0.467314	0.369318	0.724338	-0.72369	1.172118	SLPI;PGM1
doxycycline-1113	2/100	0.197972	0.467314	0.369318	0.724338	-0.72281	1.170686	IFNGR2;CTSD
flucytosine-5289	2/100	0.197972	0.467314	0.369318	0.724338	-0.72272	1.170541	RPS14P3;AP1S1
epivincamine-1783	2/100	0.197972	0.467314	0.369318	0.724338	-0.72209	1.169521	TUBA1A;PSMB9
ethotoin-3892	2/100	0.197972	0.467314	0.369318	0.724338	-0.72158	1.168686	C3;TGFBI
genistein-1660	2/100	0.197972	0.467314	0.369318	0.724338	-0.72009	1.166284	GPX2;TSPAN1
fluoxetine-3314	2/100	0.197972	0.467314	0.369318	0.724338	-0.71924	1.164902	SLPI;IFI6
flutamide-2358	2/100	0.197972	0.467314	0.369318	0.724338	-0.71899	1.164495	C3;SLC12A2
flucloxacillin-5527	2/100	0.197972	0.467314	0.369318	0.724338	-0.71871	1.164047	GPX2;ETS2
erastin-6364	2/100	0.197972	0.467314	0.369318	0.724338	-0.71861	1.163881	CTSE;LYZ
F0447-0125-6429	2/100	0.197972	0.467314	0.369318	0.724338	-0.71822	1.16325	SECTM1;SOX9
fluorocurarine-5741	2/100	0.197972	0.467314	0.369318	0.724338	-0.71812	1.163092	GPX2;IFI6
ethosuximide-1433	2/100	0.197972	0.467314	0.369318	0.724338	-0.71606	1.159756	RPS14P3;TMEM123
flunisolide-2168	2/100	0.197972	0.467314	0.369318	0.724338	-0.71572	1.159195	QPCT;IFI6
ethionamide-2495	2/100	0.197972	0.467314	0.369318	0.724338	-0.71455	1.157307	SLPI;IFI6
fluvastatin-6691	2/100	0.197972	0.467314	0.369318	0.724338	-0.71427	1.156859	CXCL1;CTGF
glibenclamide-3163	2/100	0.197972	0.467314	0.369318	0.724338	-0.71354	1.155674	S100A4;LYZ
glafenine-7257	2/100	0.197972	0.467314	0.369318	0.724338	-0.7116	1.15253	ID3;TSPAN1
foliosidine-4295	2/100	0.197972	0.467314	0.369318	0.724338	-0.70887	1.148116	MMP3;TGFBI
flunisolide-3747	2/100	0.197972	0.467314	0.369318	0.724338	-0.70831	1.147206	BMP4;MMP3
ethoxyquin-3421	2/100	0.197972	0.467314	0.369318	0.724338	-0.70826	1.147115	BMP4;S100A4

etomidate-2958	2/100	0.197972	0.467314	0.369318	0.724338	-0.70695	1.144994	PLCB4;IFI6
fluvoxamine-4114	2/100	0.197972	0.467314	0.369318	0.724338	-0.70674	1.144665	MMP3;IFI6
ethotoin-4366	2/100	0.197972	0.467314	0.369318	0.724338	-0.70672	1.144618	SLPI;ITM2C
felodipine-965	2/100	0.197972	0.467314	0.369318	0.724338	-0.70611	1.143641	BMP4;TSPAN1
fluphenazine-5880	2/100	0.197972	0.467314	0.369318	0.724338	-0.70584	1.143205	CDH3;S100A4
fulvestrant-6867	2/100	0.197972	0.467314	0.369318	0.724338	-0.70558	1.142777	SPINK1;IFI6
fluphenazine-5234	2/100	0.197972	0.467314	0.369318	0.724338	-0.70444	1.140926	MMP3;SECTM1
fenoterol-6331	2/100	0.197972	0.467314	0.369318	0.724338	-0.7038	1.139902	TSPAN1;CTGF
gelsemine-4177	2/100	0.197972	0.467314	0.369318	0.724338	-0.70336	1.139186	BMP4;TCN1
fusaric acid-3245	2/100	0.197972	0.467314	0.369318	0.724338	-0.70234	1.137536	GPX2;TSPAN1
flucytosine-6450	2/100	0.197972	0.467314	0.369318	0.724338	-0.70103	1.135403	IFNGR2;QPCT
finasteride-4300	2/100	0.197972	0.467314	0.369318	0.724338	-0.70091	1.135212	CDH3;MMP3
furazolidone-4178	2/100	0.197972	0.467314	0.369318	0.724338	-0.70079	1.135026	C3;AP1S1
etofylline-1409	2/100	0.197972	0.467314	0.369318	0.724338	-0.70022	1.134103	ITGAV;LYZ
etodolac-7246	2/100	0.197972	0.467314	0.369318	0.724338	-0.69867	1.13158	CDH3;TGFBI
fluphenazine-1178	2/100	0.197972	0.467314	0.369318	0.724338	-0.69256	1.121693	CKB;CTSD
geldanamycin-5588	2/100	0.197972	0.467314	0.369318	0.724338	-0.69146	1.119905	ID1;IER2
foliosidine-6057	2/100	0.197972	0.467314	0.369318	0.724338	-0.68952	1.116774	BMP4;MMP3
fenoprofen-2553	2/100	0.197972	0.467314	0.369318	0.724338	-0.68918	1.116214	C3;ISG15
geldanamycin-5225	2/100	0.197972	0.467314	0.369318	0.724338	-0.68751	1.113508	ID1;IER2
flupentixol-5307	2/100	0.197972	0.467314	0.369318	0.724338	-0.6873	1.113176	IFI6;AP1S1
genistein-1235	2/100	0.197972	0.467314	0.369318	0.724338	-0.68619	1.111372	CCL20;TGFBI
gliquidone-6004	2/100	0.197972	0.467314	0.369318	0.724338	-0.68598	1.111034	IFITM1;IFI6
glafenine-7018	2/100	0.197972	0.467314	0.369318	0.724338	-0.68102	1.102997	ID1;S100A4
famprofazone-4309	2/100	0.197972	0.467314	0.369318	0.724338	-0.67973	1.100911	RPS14P3;IFI6
haloperidol-5241	2/100	0.197972	0.467314	0.369318	0.724338	-0.67865	1.09916	GPX2;QPCT
fulvestrant-704	2/100	0.197972	0.467314	0.369318	0.724338	-0.67801	1.098119	PIGR;IFI6
gibberellic acid-2910	2/100	0.197972	0.467314	0.369318	0.724338	-0.67789	1.097937	IFI6;ID3
genistein-6194	2/100	0.197972	0.467314	0.369318	0.724338	-0.67719	1.096796	ANXA3;SPP1
glycocholic acid-5316	2/100	0.197972	0.467314	0.369318	0.724338	-0.67573	1.094427	AP1S1;TGFBI
fulvestrant-6872	2/100	0.197972	0.467314	0.369318	0.724338	-0.67515	1.093496	TMPRSS3;AP1S1
geldanamycin-1653	2/100	0.197972	0.467314	0.369318	0.724338	-0.67481	1.092938	ID1;IER2
H-89-6921	2/100	0.197972	0.467314	0.369318	0.724338	-0.67419	1.091933	BMP4;IFI6
flecainide-3937	2/100	0.197972	0.467314	0.369318	0.724338	-0.67418	1.091926	SPINK1;IFI6
geldanamycin-1066	2/100	0.197972	0.467314	0.369318	0.724338	-0.67322	1.090363	ID1;IER2
fulvestrant-5964	2/100	0.197972	0.467314	0.369318	0.724338	-0.67231	1.088892	TMPRSS3;TSPAN1
geldanamycin-4452	2/100	0.197972	0.467314	0.369318	0.724338	-0.66855	1.08281	BMP4;ID1

genistein-638	2/100	0.197972	0.467314	0.369318	0.724338	-0.66431	1.075943	CDH3;S100A4
geldanamycin-864	2/100	0.197972	0.467314	0.369318	0.724338	-0.66425	1.075846	ID1;IER2
guanadrel-4720	2/100	0.197972	0.467314	0.369318	0.724338	-0.66422	1.075788	SPINK1;S100A4
haloperidol-4678	2/100	0.197972	0.467314	0.369318	0.724338	-0.66375	1.075023	SPINK1;TGFBI
geldanamycin-6946	2/100	0.197972	0.467314	0.369318	0.724338	-0.66332	1.074337	ID1;IER2
heliotrine-3615	2/100	0.197972	0.467314	0.369318	0.724338	-0.66027	1.069392	SLPI;ITM2C
glycocholic acid-3315	2/100	0.197972	0.467314	0.369318	0.724338	-0.65958	1.068278	RNF43;SLPI
iohexol-3322	2/100	0.197972	0.467314	0.369318	0.724338	-0.65859	1.06667	PDIA3;MMP3
gliquidone-3126	2/100	0.197972	0.467314	0.369318	0.724338	-0.6581	1.06588	GPX2;IFI6
fulvestrant-5598	2/100	0.197972	0.467314	0.369318	0.724338	-0.65713	1.064312	SLPI;TMPRSS3
guanadrel-7396	2/100	0.197972	0.467314	0.369318	0.724338	-0.65535	1.061422	IGFBP2;TGFBI
hydrastinine-5494	2/100	0.197972	0.467314	0.369318	0.724338	-0.65503	1.060913	GPX2;IFNGR2
guanadrel-2575	2/100	0.197972	0.467314	0.369318	0.724338	-0.65492	1.060723	ANXA3;QPCT
homatropine-1806	2/100	0.197972	0.467314	0.369318	0.724338	-0.65426	1.059658	IL32;IFI6
geldanamycin-593	2/100	0.197972	0.467314	0.369318	0.724338	-0.65302	1.057652	ID1;IER2
gemfibrozil-2277	2/100	0.197972	0.467314	0.369318	0.724338	-0.65287	1.057412	BMP4;TFF2
gliquidone-7301	2/100	0.197972	0.467314	0.369318	0.724338	-0.65275	1.057218	CXCL1;CTGF
guanethidine-3171	2/100	0.197972	0.467314	0.369318	0.724338	-0.64914	1.05136	S100A4;LYZ
harpagoside-2935	2/100	0.197972	0.467314	0.369318	0.724338	-0.64459	1.043999	IFI6;LY6E
hycanthone-3229	2/100	0.197972	0.467314	0.369318	0.724338	-0.64302	1.041448	BMP4;LYZ
gossypol-6058	2/100	0.197972	0.467314	0.369318	0.724338	-0.64173	1.039363	BMP4;MMP3
hesperidin-2648	2/100	0.197972	0.467314	0.369318	0.724338	-0.64166	1.039246	BMP4;RCN1
hydroxyzine-6660	2/100	0.197972	0.467314	0.369318	0.724338	-0.64049	1.037358	MMP3;TGFBI
haloperidol-5563	2/100	0.197972	0.467314	0.369318	0.724338	-0.63922	1.035307	SLPI;S100A4
hycanthone-4630	2/100	0.197972	0.467314	0.369318	0.724338	-0.63792	1.033202	IL32;OLFM4
glycopyrronium bromide-4709	2/100	0.197972	0.467314	0.369318	0.724338	-0.63758	1.032644	RCN1;TMPRSS3
hydroquinine-6263	2/100	0.197972	0.467314	0.369318	0.724338	-0.63654	1.030959	PDIA3;TSPAN1
geldanamycin-6187	2/100	0.197972	0.467314	0.369318	0.724338	-0.63602	1.030113	ID3;CD14
genistein-2695	2/100	0.197972	0.467314	0.369318	0.724338	-0.63531	1.02897	SLPI;MEST
fulvestrant-6925	2/100	0.197972	0.467314	0.369318	0.724338	-0.63474	1.028039	IFNGR2;PRSS23
gossypol-3637	2/100	0.197972	0.467314	0.369318	0.724338	-0.63364	1.026262	BMP4;ITM2C
geldanamycin-1008	2/100	0.197972	0.467314	0.369318	0.724338	-0.63328	1.025676	ID1;IER2
guaifenesin-3897	2/100	0.197972	0.467314	0.369318	0.724338	-0.6316	1.022955	QPCT;TFF2
ionomycin-979	2/100	0.197972	0.467314	0.369318	0.724338	-0.63112	1.022188	BMP4;TFF3
hyoscyamine-2271	2/100	0.197972	0.467314	0.369318	0.724338	-0.62815	1.01737	RNF43;SECTM1
H-7-5936	2/100	0.197972	0.467314	0.369318	0.724338	-0.62694	1.015414	ID3;IER2
hesperidin-6714	2/100	0.197972	0.467314	0.369318	0.724338	-0.62675	1.015105	ID1;TSPAN1

harmol-7320	2/100	0.197972	0.467314	0.369318	0.724338	-0.62625	1.014293	MMP1;TSPAN1
isotretinoin-2407	2/100	0.197972	0.467314	0.369318	0.724338	-0.62532	1.012794	PLCB4;ANXA3
ketoconazole-4624	2/100	0.197972	0.467314	0.369318	0.724338	-0.62468	1.011755	IL32;OLFM4
heliotrine-2180	2/100	0.197972	0.467314	0.369318	0.724338	-0.62247	1.008175	IFI6;CTSD
imipramine-1849	2/100	0.197972	0.467314	0.369318	0.724338	-0.6193	1.00304	IFITM2;LY6E
imipramine-1685	2/100	0.197972	0.467314	0.369318	0.724338	-0.61869	1.002055	CDH3;S100A4
hexetidine-2457	2/100	0.197972	0.467314	0.369318	0.724338	-0.61772	1.000472	MMP3;IFI6
glibenclamide-5849	2/100	0.197972	0.467314	0.369318	0.724338	-0.61626	0.998115	CXCL1;CTGF
glipizide-1508	2/100	0.197972	0.467314	0.369318	0.724338	-0.61584	0.997436	BMP4;TFF2
harmol-1750	2/100	0.197972	0.467314	0.369318	0.724338	-0.61558	0.997014	ISG15;S100P
IC-86621-7543	2/100	0.197972	0.467314	0.369318	0.724338	-0.61552	0.996909	RNF43;IFI6
H-7-5941	2/100	0.197972	0.467314	0.369318	0.724338	-0.61426	0.99488	ID1;SOX9
iopromide-4504	2/100	0.197972	0.467314	0.369318	0.724338	-0.61202	0.991249	BMP4;RNF43
hydroxyzine-5006	2/100	0.197972	0.467314	0.369318	0.724338	-0.61198	0.991174	IFI27;ISG15
glycocholic acid-6716	2/100	0.197972	0.467314	0.369318	0.724338	-0.60937	0.986952	IFI6;TSPAN1
imipramine-5440	2/100	0.197972	0.467314	0.369318	0.724338	-0.60933	0.986893	RNF43;IFI6
hemicholinium-1601	2/100	0.197972	0.467314	0.369318	0.724338	-0.60906	0.986447	SLC12A2;S100P
hydrochlorothiazide-1906	2/100	0.197972	0.467314	0.369318	0.724338	-0.60768	0.98422	CKB;ITM2C
indapamide-3778	2/100	0.197972	0.467314	0.369318	0.724338	-0.60727	0.983547	BMP4;IFI6
kaempferol-3579	2/100	0.197972	0.467314	0.369318	0.724338	-0.60553	0.980731	BMP4;RNF43
isocarboxazid-7383	2/100	0.197972	0.467314	0.369318	0.724338	-0.60449	0.979048	SECTM1;TGFBI
ketanserin-4995	2/100	0.197972	0.467314	0.369318	0.724338	-0.6041	0.978426	IFI27;IFI6
homosalate-4355	2/100	0.197972	0.467314	0.369318	0.724338	-0.6036	0.977611	SPINK1;IFI27
hemicholinium-5339	2/100	0.197972	0.467314	0.369318	0.724338	-0.6028	0.976309	IFI6;TSPAN1
josamycin-1534	2/100	0.197972	0.467314	0.369318	0.724338	-0.60259	0.975968	BMP4;TFF2
hydralazine-3724	2/100	0.197972	0.467314	0.369318	0.724338	-0.59672	0.966468	RNF43;PSMB9
iproniazid-6359	2/100	0.197972	0.467314	0.369318	0.724338	-0.59533	0.964214	PDIA3;CDH3
liothyronine-3324	2/100	0.197972	0.467314	0.369318	0.724338	-0.59507	0.963789	LGALS3BP;BMP4
GW-8510-7085	2/100	0.197972	0.467314	0.369318	0.724338	-0.59441	0.962724	ID1;SOX9
ioversol-5326	2/100	0.197972	0.467314	0.369318	0.724338	-0.59237	0.959426	IFI6;TSPAN1
isocarboxazid-2562	2/100	0.197972	0.467314	0.369318	0.724338	-0.5912	0.957527	TSPAN13;LY6E
hexamethonium bromide-4965	2/100	0.197972	0.467314	0.369318	0.724338	-0.59078	0.956839	IFI27;IFI6
hexamethonium bromide-1982	2/100	0.197972	0.467314	0.369318	0.724338	-0.58917	0.954234	TMEM123;LYZ
isoxicam-7268	2/100	0.197972	0.467314	0.369318	0.724338	-0.58913	0.954172	CDH3;MMP1
levcycloserine-3870	2/100	0.197972	0.467314	0.369318	0.724338	-0.58823	0.952713	C3;ITM2C
hydrastinine-5075	2/100	0.197972	0.467314	0.369318	0.724338	-0.58255	0.943515	LGALS3BP;IFI6
josamycin-4631	2/100	0.197972	0.467314	0.369318	0.724338	-0.58224	0.943021	OLFM4;DUOX2

HNMPA-(AM)3-583	2/100	0.197972	0.467314	0.369318	0.724338	-0.5812	0.941324	CDH3;CTGF
isoxicam-1862	2/100	0.197972	0.467314	0.369318	0.724338	-0.5791	0.937932	C3;LY6E
iohexol-4643	2/100	0.197972	0.467314	0.369318	0.724338	-0.57476	0.930904	IL32;OLFM4
idoxuridine-1899	2/100	0.197972	0.467314	0.369318	0.724338	-0.57447	0.930424	IFI6;NOP10
leflunomide-6102	2/100	0.197972	0.467314	0.369318	0.724338	-0.57446	0.930406	MMP3;TFF2
indometacin-2377	2/100	0.197972	0.467314	0.369318	0.724338	-0.57325	0.928446	ISG15;CKB
indapamide-2361	2/100	0.197972	0.467314	0.369318	0.724338	-0.57298	0.928023	C3;LY6E
isoflupredone-5545	2/100	0.197972	0.467314	0.369318	0.724338	-0.57208	0.926561	RNF43;GPX2
hesperetin-5350	2/100	0.197972	0.467314	0.369318	0.724338	-0.57144	0.92552	IFI6;ISG15
leflunomide-7238	2/100	0.197972	0.467314	0.369318	0.724338	-0.56917	0.921853	GPX2;S100A4
iloprost-427	2/100	0.197972	0.467314	0.369318	0.724338	-0.5691	0.921726	CDH3;TGFBI
hydroxyzine-1941	2/100	0.197972	0.467314	0.369318	0.724338	-0.56785	0.919712	GPX2;IGFBP2
loperamide-5267	2/100	0.197972	0.467314	0.369318	0.724338	-0.56729	0.918803	IFI27;MMP3
levocabastine-3509	2/100	0.197972	0.467314	0.369318	0.724338	-0.56678	0.917969	S100A4;ETS2
kanamycin-1609	2/100	0.197972	0.467314	0.369318	0.724338	-0.56657	0.917635	ITGAV;FOS
iopanoic acid-5448	2/100	0.197972	0.467314	0.369318	0.724338	-0.56579	0.916372	IFITM1;TGFBI
karakoline-6059	2/100	0.197972	0.467314	0.369318	0.724338	-0.56458	0.914409	BMP4;MMP3
lumicolchicine-3254	2/100	0.197972	0.467314	0.369318	0.724338	-0.56447	0.91423	GPX2;SECTM1
ketoconazole-1285	2/100	0.197972	0.467314	0.369318	0.724338	-0.56078	0.908251	SPP1;CTSD
ketoprofen-3729	2/100	0.197972	0.467314	0.369318	0.724338	-0.56076	0.908223	BMP4;IFI6
ketorolac-6489	2/100	0.197972	0.467314	0.369318	0.724338	-0.55888	0.905179	GPX2;ID1
L-methionine sulfoximine-4151	2/100	0.197972	0.467314	0.369318	0.724338	-0.55846	0.904496	AP1S1;TFF2
isoxsuprine-4789	2/100	0.197972	0.467314	0.369318	0.724338	-0.55812	0.903956	IFNGR2;QPCT
levobunolol-3354	2/100	0.197972	0.467314	0.369318	0.724338	-0.55808	0.903887	RNF43;TFF2
isoconazole-2056	2/100	0.197972	0.467314	0.369318	0.724338	-0.55756	0.903034	IGFBP2;ITM2C
ketotifen-3200	2/100	0.197972	0.467314	0.369318	0.724338	-0.55739	0.902765	GPX2;LYZ
kanamycin-3224	2/100	0.197972	0.467314	0.369318	0.724338	-0.55704	0.902206	BMP4;LYZ
ivermectin-1367	2/100	0.197972	0.467314	0.369318	0.724338	-0.55581	0.900199	IFITM3;PIGR
lomefloxacin-2310	2/100	0.197972	0.467314	0.369318	0.724338	-0.55393	0.897155	TMPRSS3;TFF2
lisuride-1545	2/100	0.197972	0.467314	0.369318	0.724338	-0.55134	0.89297	TFF2;S100A4
lovastatin-2854	2/100	0.197972	0.467314	0.369318	0.724338	-0.54935	0.889743	TFF2;S100A4
levodopa-4394	2/100	0.197972	0.467314	0.369318	0.724338	-0.54926	0.889601	IFI27;IFI6
LY-294002-4463	2/100	0.197972	0.467314	0.369318	0.724338	-0.54597	0.884274	C3;CXCL1
lovastatin-4978	2/100	0.197972	0.467314	0.369318	0.724338	-0.54572	0.883869	IFI27;IFI6
lomefloxacin-2348	2/100	0.197972	0.467314	0.369318	0.724338	-0.54431	0.881588	TSPAN13;QPCT
ivermectin-7206	2/100	0.197972	0.467314	0.369318	0.724338	-0.54193	0.87772	BMP4;S100A4
levcycloserine-4346	2/100	0.197972	0.467314	0.369318	0.724338	-0.54096	0.876151	SLPI;IFI27

levonorgestrel-3406	2/100	0.197972	0.467314	0.369318	0.724338	-0.54036	0.875183	LGALS3BP;CDH3
levonorgestrel-4269	2/100	0.197972	0.467314	0.369318	0.724338	-0.5381	0.871529	RPS14P3;IFI6
ketanserin-1593	2/100	0.197972	0.467314	0.369318	0.724338	-0.53762	0.870743	RPS14P3;ITGAV
isoetarine-5812	2/100	0.197972	0.467314	0.369318	0.724338	-0.53734	0.870297	LGALS3BP;CTGF
lactobionic acid-4950	2/100	0.197972	0.467314	0.369318	0.724338	-0.53682	0.869447	IFI27;IFI6
loperamide-5632	2/100	0.197972	0.467314	0.369318	0.724338	-0.53506	0.866596	SLPI;TFF2
lidocaine-4421	2/100	0.197972	0.467314	0.369318	0.724338	-0.5344	0.86553	MMP3;AP1S1
lomustine-7050	2/100	0.197972	0.467314	0.369318	0.724338	-0.53407	0.864994	C3;TXNIP
ketotifen-1583	2/100	0.197972	0.467314	0.369318	0.724338	-0.53231	0.862148	SLPI;MEST
mafenide-2287	2/100	0.197972	0.467314	0.369318	0.724338	-0.52545	0.851034	SECTM1;TSPAN1
loperamide-4672	2/100	0.197972	0.467314	0.369318	0.724338	-0.5253	0.850784	GPX2;IFI6
lidocaine-1499	2/100	0.197972	0.467314	0.369318	0.724338	-0.52367	0.848157	RNF43;ISG15
LY-294002-5965	2/100	0.197972	0.467314	0.369318	0.724338	-0.52292	0.846936	TMPRSS3;SECTM1
LY-294002-4460	2/100	0.197972	0.467314	0.369318	0.724338	-0.5217	0.844955	BMP4;CXCL1
mefloquine-2210	2/100	0.197972	0.467314	0.369318	0.724338	-0.51917	0.840858	BMP4;AP1S1
hyoscyamine-1424	2/100	0.197972	0.467314	0.369318	0.724338	-0.5188	0.840269	IFITM3;PFN1
lithocholic acid-3816	2/100	0.197972	0.467314	0.369318	0.724338	-0.51742	0.838033	C3;FOS
lidoflazine-3201	2/100	0.197972	0.467314	0.369318	0.724338	-0.51706	0.837442	RNF43;LYZ
melatonin-5393	2/100	0.197972	0.467314	0.369318	0.724338	-0.51579	0.835395	IFI27;TGFBI
LY-294002-6935	2/100	0.197972	0.467314	0.369318	0.724338	-0.51506	0.8342	LGALS3BP;BMP4
lynestrenol-6756	2/100	0.197972	0.467314	0.369318	0.724338	-0.51325	0.831271	GPX2;IFI6
metacycline-7321	2/100	0.197972	0.467314	0.369318	0.724338	-0.51254	0.830118	ISG15;TSPAN1
metampicillin-1440	2/100	0.197972	0.467314	0.369318	0.724338	-0.51225	0.829653	SLC12A2;SLPI
mebendazole-3671	2/100	0.197972	0.467314	0.369318	0.724338	-0.51095	0.827555	TUBA1A;MMP3
lobeline-5784	2/100	0.197972	0.467314	0.369318	0.724338	-0.51052	0.826854	IFI6;PSMB9
mebhydrolin-4795	2/100	0.197972	0.467314	0.369318	0.724338	-0.50993	0.825899	IFNGR2;QPCT
memantine-7354	2/100	0.197972	0.467314	0.369318	0.724338	-0.50897	0.824349	BMP4;IFI6
melatonin-6293	2/100	0.197972	0.467314	0.369318	0.724338	-0.5058	0.819204	PDIA3;LGALS3BP
mephentermine-7384	2/100	0.197972	0.467314	0.369318	0.724338	-0.50398	0.816259	AP1S1;CTSD
levocabastine-2948	2/100	0.197972	0.467314	0.369318	0.724338	-0.50391	0.816149	IFI6;PGM1
LY-294002-5224	2/100	0.197972	0.467314	0.369318	0.724338	-0.5015	0.812251	TMPRSS3;ETS2
luteolin-6658	2/100	0.197972	0.467314	0.369318	0.724338	-0.50085	0.811191	ID3;CTGF
mephenesin-3675	2/100	0.197972	0.467314	0.369318	0.724338	-0.50076	0.811039	MMP3;IFI6
lidocaine-1999	2/100	0.197972	0.467314	0.369318	0.724338	-0.50018	0.810107	SLPI;ITGAV
lobeline-1770	2/100	0.197972	0.467314	0.369318	0.724338	-0.4999	0.809651	CCL20;HSPB1
metanephrine-1515	2/100	0.197972	0.467314	0.369318	0.724338	-0.49683	0.804686	TMPRSS3;TFF2
LY-294002-5587	2/100	0.197972	0.467314	0.369318	0.724338	-0.49626	0.803765	BMP4;TMPRSS3

mafenide-5079	2/100	0.197972	0.467314	0.369318	0.724338	-0.49583	0.803054	GPX2;IFI6
LY-294002-1227	2/100	0.197972	0.467314	0.369318	0.724338	-0.49178	0.796495	IL32;CCL20
lycorine-3808	2/100	0.197972	0.467314	0.369318	0.724338	-0.4915	0.79605	BMP4;ID1
LY-294002-6175	2/100	0.197972	0.467314	0.369318	0.724338	-0.49127	0.795673	TSPAN13;ANXA3
LY-294002-6198	2/100	0.197972	0.467314	0.369318	0.724338	-0.49003	0.79366	CCL20;ID3
meclocycline-4982	2/100	0.197972	0.467314	0.369318	0.724338	-0.48912	0.792187	IFI6;S100A4
meticrane-5984	2/100	0.197972	0.467314	0.369318	0.724338	-0.48908	0.792131	BMP4;IFI6
metyrapone-6447	2/100	0.197972	0.467314	0.369318	0.724338	-0.48884	0.791736	PDIA3;TSPAN1
LY-294002-5233	2/100	0.197972	0.467314	0.369318	0.724338	-0.48851	0.791209	BMP4;TMPRSS3
melatonin-3293	2/100	0.197972	0.467314	0.369318	0.724338	-0.48661	0.788127	S100A6;IFI6
meclofenoxate-4729	2/100	0.197972	0.467314	0.369318	0.724338	-0.48459	0.78485	PDIA3;IFI6
LY-294002-1077	2/100	0.197972	0.467314	0.369318	0.724338	-0.48325	0.782689	IFNGR2;ISG15
meptazinol-7326	2/100	0.197972	0.467314	0.369318	0.724338	-0.48319	0.782592	LGALS3BP;ISG15
meclozine-5244	2/100	0.197972	0.467314	0.369318	0.724338	-0.48228	0.781119	GPX2;IFI27
methapyrilene-4990	2/100	0.197972	0.467314	0.369318	0.724338	-0.48169	0.780168	TMPRSS3;S100A4
levothyroxine sodium-4150	2/100	0.197972	0.467314	0.369318	0.724338	-0.48004	0.777492	TCN1;AP1S1
metformin-3	2/100	0.197972	0.467314	0.369318	0.724338	-0.47975	0.777022	TCN1;ID3
methanthelinium bromide-6137	2/100	0.197972	0.467314	0.369318	0.724338	-0.47897	0.775757	GPX2;TSPAN13
mesoridazine-7017	2/100	0.197972	0.467314	0.369318	0.724338	-0.47881	0.775489	S100A4;TSPAN1
mefloquine-6205	2/100	0.197972	0.467314	0.369318	0.724338	-0.47878	0.775442	ENC1;AP1S1
metergoline-5344	2/100	0.197972	0.467314	0.369318	0.724338	-0.47842	0.774867	IFI6;TSPAN1
lycorine-4365	2/100	0.197972	0.467314	0.369318	0.724338	-0.47788	0.773986	LGALS3BP;IFITM1
meclofenoxate-4268	2/100	0.197972	0.467314	0.369318	0.724338	-0.47736	0.773145	RPS14P3;IFI6
methocarbamol-7467	2/100	0.197972	0.467314	0.369318	0.724338	-0.47672	0.772109	LGALS3BP;AP1S1
mesoridazine-2874	2/100	0.197972	0.467314	0.369318	0.724338	-0.47574	0.770521	LGALS3BP;C3
metamizole sodium-2175	2/100	0.197972	0.467314	0.369318	0.724338	-0.47464	0.768749	TSPAN13;IFI6
LY-294002-4440	2/100	0.197972	0.467314	0.369318	0.724338	-0.47425	0.768103	MMP1;IFI6
mefexamide-2121	2/100	0.197972	0.467314	0.369318	0.724338	-0.47272	0.765625	TSPAN8;SECTM1
mecamylamine-7023	2/100	0.197972	0.467314	0.369318	0.724338	-0.47126	0.763264	BMP4;TSPAN1
LY-294002-4451	2/100	0.197972	0.467314	0.369318	0.724338	-0.4711	0.763013	C3;CXCL1
LY-294002-6186	2/100	0.197972	0.467314	0.369318	0.724338	-0.47089	0.762664	ANXA3;S100P
minoxidil-1496	2/100	0.197972	0.467314	0.369318	0.724338	-0.46762	0.75738	LGALS3BP;BMP4
mefexamide-2284	2/100	0.197972	0.467314	0.369318	0.724338	-0.46744	0.757087	SLPI;AP1S1
meteneprost-7557	2/100	0.197972	0.467314	0.369318	0.724338	-0.46581	0.754438	MMP1;CTGF
LY-294002-1065	2/100	0.197972	0.467314	0.369318	0.724338	-0.46571	0.754271	CKB;CTSD
metoclopramide-3728	2/100	0.197972	0.467314	0.369318	0.724338	-0.4655	0.753937	BMP4;PSMB9
meclofenamic acid-2128	2/100	0.197972	0.467314	0.369318	0.724338	-0.46536	0.753718	CCL20;CTGF

mometasone-2896	2/100	0.197972	0.467314	0.369318	0.724338	-0.46511	0.7533	GPX2;SECTM1
metergoline-1606	2/100	0.197972	0.467314	0.369318	0.724338	-0.46494	0.753036	IFITM3;SLC12A2
methyldopa-3234	2/100	0.197972	0.467314	0.369318	0.724338	-0.46335	0.75045	TGFBI;LYZ
methylprednisolone-1567	2/100	0.197972	0.467314	0.369318	0.724338	-0.4632	0.750208	IFI6;CTSH
methyldopate-4986	2/100	0.197972	0.467314	0.369318	0.724338	-0.46289	0.749711	IFI27;IFI6
metixene-3313	2/100	0.197972	0.467314	0.369318	0.724338	-0.45895	0.743332	IFI6;SECTM1
metitepine-5413	2/100	0.197972	0.467314	0.369318	0.724338	-0.45803	0.741845	IFI27;QPCT
molsidomine-2862	2/100	0.197972	0.467314	0.369318	0.724338	-0.45735	0.740734	GPX2;TFF2
maprotiline-3236	2/100	0.197972	0.467314	0.369318	0.724338	-0.45508	0.737056	BMP4;LYZ
milrinone-3552	2/100	0.197972	0.467314	0.369318	0.724338	-0.45487	0.736724	TMPRSS3;S100A4
metolazone-5392	2/100	0.197972	0.467314	0.369318	0.724338	-0.45476	0.736537	IFI27;IFI6
miconazole-4960	2/100	0.197972	0.467314	0.369318	0.724338	-0.45281	0.733386	IFI27;IFI6
monorden-5579	2/100	0.197972	0.467314	0.369318	0.724338	-0.45273	0.733249	SLPI;ID1
methylprednisolone-3183	2/100	0.197972	0.467314	0.369318	0.724338	-0.4504	0.729478	GPX2;LYZ
minaprine-1468	2/100	0.197972	0.467314	0.369318	0.724338	-0.45013	0.729039	BMP4;RNF43
norfloxacin-5985	2/100	0.197972	0.467314	0.369318	0.724338	-0.44641	0.723023	IFI6;S100A4
metaraminol-7368	2/100	0.197972	0.467314	0.369318	0.724338	-0.44526	0.721149	CDH3;MMP1
metixene-6672	2/100	0.197972	0.467314	0.369318	0.724338	-0.44519	0.721037	MMP3;TGFBI
metitepine-3231	2/100	0.197972	0.467314	0.369318	0.724338	-0.44371	0.718642	BMP4;LYZ
meteneprost-7500	2/100	0.197972	0.467314	0.369318	0.724338	-0.44298	0.717456	SLPI;IFI6
minocycline-5496	2/100	0.197972	0.467314	0.369318	0.724338	-0.44249	0.716675	GPX2;ISG15
mexiletine-2364	2/100	0.197972	0.467314	0.369318	0.724338	-0.44228	0.716338	ANXA3;ISG15
methapyrilene-1588	2/100	0.197972	0.467314	0.369318	0.724338	-0.44066	0.713712	IFITM3;SLC12A2
mesalazine-124	2/100	0.197972	0.467314	0.369318	0.724338	-0.43956	0.711923	TMEM123;ID3
methoxsalen-6661	2/100	0.197972	0.467314	0.369318	0.724338	-0.4389	0.710861	C3;PSMB9
nilutamide-6481	2/100	0.197972	0.467314	0.369318	0.724338	-0.43545	0.705273	TMPRSS3;CTSE
meticrane-1834	2/100	0.197972	0.467314	0.369318	0.724338	-0.43377	0.702542	CCL20;ISG15
meropenem-5824	2/100	0.197972	0.467314	0.369318	0.724338	-0.43344	0.702012	TSPAN1;CTSD
nadolol-4021	2/100	0.197972	0.467314	0.369318	0.724338	-0.43172	0.699223	MMP3;IFI6
moroxydine-1527	2/100	0.197972	0.467314	0.369318	0.724338	-0.42973	0.695997	BMP4;TFF2
methylergometrine-6704	2/100	0.197972	0.467314	0.369318	0.724338	-0.42893	0.694711	ID1;CTSD
MG-262-7079	2/100	0.197972	0.467314	0.369318	0.724338	-0.4285	0.694006	SOX9;PHLDA1
monorden-999	2/100	0.197972	0.467314	0.369318	0.724338	-0.42644	0.690675	ID1;IER2
nalidixic acid-3668	2/100	0.197972	0.467314	0.369318	0.724338	-0.42548	0.689114	TSPAN8;MMP3
naftidrofuryl-2622	2/100	0.197972	0.467314	0.369318	0.724338	-0.42312	0.685302	PDIA3;BMP4
N6-methyladenosine-5332	2/100	0.197972	0.467314	0.369318	0.724338	-0.42278	0.684745	TMPRSS3;S100A6
nimesulide-7024	2/100	0.197972	0.467314	0.369318	0.724338	-0.42208	0.683617	BMP4;TSPAN1

MG-262-7068	2/100	0.197972	0.467314	0.369318	0.724338	-0.42199	0.683461	SOX9;PHLDA1
napelline-6824	2/100	0.197972	0.467314	0.369318	0.724338	-0.42188	0.683296	IFI27;PGM1
naltrexone-1363	2/100	0.197972	0.467314	0.369318	0.724338	-0.42183	0.683202	PDIA3;IFITM3
nizatidine-3385	2/100	0.197972	0.467314	0.369318	0.724338	-0.41894	0.678525	SLPI;PGM1
monobenzone-3391	2/100	0.197972	0.467314	0.369318	0.724338	-0.4185	0.677819	C3;SECTM1
monastrol-596	2/100	0.197972	0.467314	0.369318	0.724338	-0.41841	0.677677	RNF43;CTSD
novobiocin-4392	2/100	0.197972	0.467314	0.369318	0.724338	-0.4166	0.674741	SPINK1;IFI27
pancuronium bromide-2909	2/100	0.197972	0.467314	0.369318	0.724338	-0.41586	0.673541	TCN1;ID3
moracizine-7297	2/100	0.197972	0.467314	0.369318	0.724338	-0.41463	0.671552	IFI6;CTSD
methazolamide-5794	2/100	0.197972	0.467314	0.369318	0.724338	-0.4128	0.66858	MMP3;IFI6
mometasone-1746	2/100	0.197972	0.467314	0.369318	0.724338	-0.41124	0.66605	ISG15;CKB
molsidomine-6325	2/100	0.197972	0.467314	0.369318	0.724338	-0.41039	0.664674	CDH3;FXYD3
midecamycin-1943	2/100	0.197972	0.467314	0.369318	0.724338	-0.40874	0.662001	S100A6;ITGAV
N-acetyl-L-leucine-6462	2/100	0.197972	0.467314	0.369318	0.724338	-0.40843	0.661503	IFNGR2;SECTM1
niflumic acid-5490	2/100	0.197972	0.467314	0.369318	0.724338	-0.4072	0.659518	GPX2;QPCT
nimodipine-5421	2/100	0.197972	0.467314	0.369318	0.724338	-0.40713	0.659393	IFI27;TFF2
mometasone-5541	2/100	0.197972	0.467314	0.369318	0.724338	-0.40578	0.657221	RNF43;GPX2
nicergoline-6251	2/100	0.197972	0.467314	0.369318	0.724338	-0.40555	0.656838	LGALS3BP;PGM1
oxyphenbutazone-6844	2/100	0.197972	0.467314	0.369318	0.724338	-0.40472	0.6555	IFI27;IFI6
monorden-5952	2/100	0.197972	0.467314	0.369318	0.724338	-0.40207	0.651208	CTSE;PROM1
mexiletine-4338	2/100	0.197972	0.467314	0.369318	0.724338	-0.40101	0.649489	C3;CDH3
mifepristone-1569	2/100	0.197972	0.467314	0.369318	0.724338	-0.40023	0.648218	SLPI;FOS
naphazoline-4949	2/100	0.197972	0.467314	0.369318	0.724338	-0.39894	0.646136	IFI27;QPCT
naloxone-4645	2/100	0.197972	0.467314	0.369318	0.724338	-0.39555	0.640639	TGFBI;ITM2C
nitrofural-5321	2/100	0.197972	0.467314	0.369318	0.724338	-0.39415	0.638381	IFI6;ISG15
niclosamide-1916	2/100	0.197972	0.467314	0.369318	0.724338	-0.39313	0.636729	ID1;SOX9
paclitaxel-640	2/100	0.197972	0.467314	0.369318	0.724338	-0.39027	0.632092	TCN1;DPEP1
monastrol-605	2/100	0.197972	0.467314	0.369318	0.724338	-0.38989	0.631483	TCN1;MMP3
monorden-5947	2/100	0.197972	0.467314	0.369318	0.724338	-0.38852	0.629264	LGALS3BP;TSPAN1
minaprine-1888	2/100	0.197972	0.467314	0.369318	0.724338	-0.38785	0.628177	TMEM123;PRSS23
oleandomycin-4615	2/100	0.197972	0.467314	0.369318	0.724338	-0.38706	0.626901	TGFBI;OLFM4
nicardipine-3215	2/100	0.197972	0.467314	0.369318	0.724338	-0.38684	0.626536	GPX2;LYZ
metolazone-2014	2/100	0.197972	0.467314	0.369318	0.724338	-0.38497	0.623517	ITGAV;DYNLL1
moxisylyte-7255	2/100	0.197972	0.467314	0.369318	0.724338	-0.38413	0.622154	TGFBI;ITM2C
nordihydroguaiaretic acid-1223	2/100	0.197972	0.467314	0.369318	0.724338	-0.38378	0.621587	TSPAN8;TGFBI
metolazone-6292	2/100	0.197972	0.467314	0.369318	0.724338	-0.3836	0.621292	TSPAN8;TCN1
oxedrine-3578	2/100	0.197972	0.467314	0.369318	0.724338	-0.38089	0.616905	SECTM1;S100A4

ouabain-6680	2/100	0.197972	0.467314	0.369318	0.724338	-0.38023	0.615832	C3;PSMB9
MG-132-1140	2/100	0.197972	0.467314	0.369318	0.724338	-0.37916	0.614093	ISG15;IER2
PF-00562151-00-5959	2/100	0.197972	0.467314	0.369318	0.724338	-0.37796	0.612154	IL32;BMP4
nefopam-2355	2/100	0.197972	0.467314	0.369318	0.724338	-0.37774	0.611792	C3;TSPAN13
nifenazone-6016	2/100	0.197972	0.467314	0.369318	0.724338	-0.37483	0.607079	IFI6;LY6E
orphenadrine-3801	2/100	0.197972	0.467314	0.369318	0.724338	-0.37466	0.606807	BMP4;RNF43
monobenzone-6713	2/100	0.197972	0.467314	0.369318	0.724338	-0.37393	0.605633	GPX2;IFI6
nalidixic acid-4691	2/100	0.197972	0.467314	0.369318	0.724338	-0.37278	0.603759	RCN1;SPINK1
pentoxyverine-5610	2/100	0.197972	0.467314	0.369318	0.724338	-0.37195	0.602425	BMP4;ISG15
norfloxacin-2253	2/100	0.197972	0.467314	0.369318	0.724338	-0.37162	0.601891	SLPI;SECTM1
norethisterone-2383	2/100	0.197972	0.467314	0.369318	0.724338	-0.37162	0.60188	C3;TSPAN13
niflumic acid-7430	2/100	0.197972	0.467314	0.369318	0.724338	-0.3709	0.600727	S100A4;PGM1
mycophenolic acid-4019	2/100	0.197972	0.467314	0.369318	0.724338	-0.37021	0.599604	MMP3;CXCL1
parbendazole-3881	2/100	0.197972	0.467314	0.369318	0.724338	-0.36801	0.596043	C3;GPX2
napelline-2522	2/100	0.197972	0.467314	0.369318	0.724338	-0.36745	0.59513	ANXA3;ISG15
monorden-1057	2/100	0.197972	0.467314	0.369318	0.724338	-0.36625	0.593187	ID1;IER2
nystatin-4807	2/100	0.197972	0.467314	0.369318	0.724338	-0.36599	0.592776	QPCT;DSG2
nicardipine-1600	2/100	0.197972	0.467314	0.369318	0.724338	-0.36428	0.589997	SLC12A2;PIGR
paromomycin-3356	2/100	0.197972	0.467314	0.369318	0.724338	-0.36421	0.589888	BMP4;TFF2
oxamniquine-7344	2/100	0.197972	0.467314	0.369318	0.724338	-0.36418	0.589835	TMPRSS3;IFI6
PHA-00851261E-3857	2/100	0.197972	0.467314	0.369318	0.724338	-0.36364	0.588966	SLPI;IFI6
nipecotic acid-6500	2/100	0.197972	0.467314	0.369318	0.724338	-0.36323	0.588291	LGALS3BP;GPX2
paracetamol-5384	2/100	0.197972	0.467314	0.369318	0.724338	-0.36315	0.588174	IFI27;IFI6
pheneticillin-6105	2/100	0.197972	0.467314	0.369318	0.724338	-0.36151	0.58552	CEACAM5;TSPAN1
nordihydroguaiaretic acid-6182	2/100	0.197972	0.467314	0.369318	0.724338	-0.35862	0.580825	CCL20;TCN1
nizatidine-5406	2/100	0.197972	0.467314	0.369318	0.724338	-0.35769	0.579326	IFITM1;IFI27
pentolonium-3676	2/100	0.197972	0.467314	0.369318	0.724338	-0.35612	0.576778	MMP3;IFI6
oxetacaine-4829	2/100	0.197972	0.467314	0.369318	0.724338	-0.35314	0.571959	ID1;TGFBI
nomifensine-5863	2/100	0.197972	0.467314	0.369318	0.724338	-0.35182	0.569824	PDIA3;TCN1
nomegestrol-5461	2/100	0.197972	0.467314	0.369318	0.724338	-0.35181	0.569804	PDIA3;GPX2
phenelzine-3802	2/100	0.197972	0.467314	0.369318	0.724338	-0.3509	0.568328	IL32;SLCO5A1
paclitaxel-1542	2/100	0.197972	0.467314	0.369318	0.724338	-0.35047	0.56764	TMPRSS3;TFF2
oleandomycin-1935	2/100	0.197972	0.467314	0.369318	0.724338	-0.34906	0.565353	GPX2;PRSS23
oxolamine-4969	2/100	0.197972	0.467314	0.369318	0.724338	-0.34899	0.565234	IFI27;IFI6
nocodazole-621	2/100	0.197972	0.467314	0.369318	0.724338	-0.34661	0.561379	TUBA1A;TUBB
omeprazole-2828	2/100	0.197972	0.467314	0.369318	0.724338	-0.34614	0.560613	TMPRSS3;TFF2
noretynodrel-1860	2/100	0.197972	0.467314	0.369318	0.724338	-0.34378	0.55679	C3;ANXA3

oxybutynin-6770	2/100	0.197972	0.467314	0.369318	0.724338	-0.34292	0.555402	IFI6;S100A4
pilocarpine-5341	2/100	0.197972	0.467314	0.369318	0.724338	-0.34118	0.552585	GPX2;IFI6
PHA-00851261E-3776	2/100	0.197972	0.467314	0.369318	0.724338	-0.33909	0.549195	MMP3;PSMB9
NU-1025-313	2/100	0.197972	0.467314	0.369318	0.724338	-0.33894	0.548956	IFI27;ISG15
paclitaxel-1959	2/100	0.197972	0.467314	0.369318	0.724338	-0.33669	0.545315	TSPAN13;CBX3
NS-398-6892	2/100	0.197972	0.467314	0.369318	0.724338	-0.33649	0.544992	C3;AP1S1
PHA-00846566E-7081	2/100	0.197972	0.467314	0.369318	0.724338	-0.33634	0.544752	TMPRSS3;S100A4
oxybutynin-1551	2/100	0.197972	0.467314	0.369318	0.724338	-0.33614	0.544419	SLPI;CKB
pepstatin-4790	2/100	0.197972	0.467314	0.369318	0.724338	-0.33598	0.544158	IFI6;TSPAN1
novobiocin-437	2/100	0.197972	0.467314	0.369318	0.724338	-0.33557	0.543507	TCN1;TSPAN1
octopamine-5469	2/100	0.197972	0.467314	0.369318	0.724338	-0.33507	0.542686	RNF43;QPCT
pentetic acid-3387	2/100	0.197972	0.467314	0.369318	0.724338	-0.33502	0.542615	PDIA3;AP1S1
nifedipine-7303	2/100	0.197972	0.467314	0.369318	0.724338	-0.33421	0.5413	IFI6;ID3
prasterone-6474	2/100	0.197972	0.467314	0.369318	0.724338	-0.33421	0.541294	SECTM1;TSPAN1
pentetrazol-2092	2/100	0.197972	0.467314	0.369318	0.724338	-0.33356	0.540251	IL32;CCL20
PHA-00745360-3824	2/100	0.197972	0.467314	0.369318	0.724338	-0.33344	0.540053	MMP3;SLCO5A1
ofloxacin-2340	2/100	0.197972	0.467314	0.369318	0.724338	-0.32965	0.533916	SLC12A2;QPCT
PHA-00851261E-3968	2/100	0.197972	0.467314	0.369318	0.724338	-0.32634	0.528552	QPCT;AP1S1
phthalylsulfathiazole-5249	2/100	0.197972	0.467314	0.369318	0.724338	-0.32633	0.528528	IFI6;CTSD
phenacetin-4111	2/100	0.197972	0.467314	0.369318	0.724338	-0.3262	0.528319	MMP3;TFF2
pararosaniline-893	2/100	0.197972	0.467314	0.369318	0.724338	-0.32554	0.527262	LGALS3BP;BMP4
PNU-0251126-4711	2/100	0.197972	0.467314	0.369318	0.724338	-0.32493	0.526267	BMP4;SPINK1
pindolol-6834	2/100	0.197972	0.467314	0.369318	0.724338	-0.32451	0.525586	IFI6;S100A4
pentoxyverine-1268	2/100	0.197972	0.467314	0.369318	0.724338	-0.32351	0.52396	CCL20;ANXA3
oxybenzone-6469	2/100	0.197972	0.467314	0.369318	0.724338	-0.32325	0.52355	GPX2;SECTM1
paromomycin-4420	2/100	0.197972	0.467314	0.369318	0.724338	-0.32124	0.520287	AP1S1;S100A4
phenoxybenzamine-5248	2/100	0.197972	0.467314	0.369318	0.724338	-0.31932	0.517175	IFI6;TXNIP
niclosamide-1998	2/100	0.197972	0.467314	0.369318	0.724338	-0.31908	0.516786	IFITM3;DYNLL1
pentoxyverine-2623	2/100	0.197972	0.467314	0.369318	0.724338	-0.31864	0.51608	TMPRSS3;S100A4
nystatin-2500	2/100	0.197972	0.467314	0.369318	0.724338	-0.31585	0.511568	TSPAN13;IFI6
phthalylsulfathiazole-3371	2/100	0.197972	0.467314	0.369318	0.724338	-0.31393	0.508446	PDIA3;AP1S1
picotamide-2233	2/100	0.197972	0.467314	0.369318	0.724338	-0.31267	0.506406	C3;S100A4
pralidoxime-6443	2/100	0.197972	0.467314	0.369318	0.724338	-0.3118	0.504999	PDIA3;TSPAN1
phenoxybenzamine-4652	2/100	0.197972	0.467314	0.369318	0.724338	-0.31169	0.504816	ID1;SOX9
pralidoxime-5383	2/100	0.197972	0.467314	0.369318	0.724338	-0.30797	0.498801	IFI27;IFI6
pizotifen-3134	2/100	0.197972	0.467314	0.369318	0.724338	-0.30455	0.493255	GPX2;TSPAN13
piroxicam-2252	2/100	0.197972	0.467314	0.369318	0.724338	-0.30308	0.490875	SLPI;SECTM1

pioglitazone-7088	2/100	0.197972	0.467314	0.369318	0.724338	-0.30208	0.489262	TCN1;CTGF
PHA-00851261E-3965	2/100	0.197972	0.467314	0.369318	0.724338	-0.30179	0.488789	SPINK1;AP1S1
Prestwick-642-4419	2/100	0.197972	0.467314	0.369318	0.724338	-0.30023	0.486256	IFITM1;IFI6
picrotoxinin-4842	2/100	0.197972	0.467314	0.369318	0.724338	-0.29897	0.484214	RCN1;SLPI
phenacetin-2832	2/100	0.197972	0.467314	0.369318	0.724338	-0.29807	0.482761	C3;TFF2
phenelzine-3884	2/100	0.197972	0.467314	0.369318	0.724338	-0.29779	0.482308	BMP4;QPCT
pirenperone-3316	2/100	0.197972	0.467314	0.369318	0.724338	-0.29647	0.480175	C3;RPS14P3
phenazopyridine-2537	2/100	0.197972	0.467314	0.369318	0.724338	-0.29568	0.478895	C3;TSPAN13
PHA-00745360-3910	2/100	0.197972	0.467314	0.369318	0.724338	-0.29456	0.477082	RPS14P3;SECTM1
pizotifen-5072	2/100	0.197972	0.467314	0.369318	0.724338	-0.29212	0.473131	GPX2;TUBA1A
Prestwick-1085-3554	2/100	0.197972	0.467314	0.369318	0.724338	-0.29212	0.473123	GPX2;TMPRSS3
Prestwick-1084-6125	2/100	0.197972	0.467314	0.369318	0.724338	-0.29082	0.471017	GPX2;TSPAN13
pivmecillinam-2973	2/100	0.197972	0.467314	0.369318	0.724338	-0.28912	0.468268	SPP1;PGM1
PNU-0230031-4757	2/100	0.197972	0.467314	0.369318	0.724338	-0.28846	0.4672	GPX2;IFI6
Prestwick-664-3613	2/100	0.197972	0.467314	0.369318	0.724338	-0.28825	0.466857	RNF43;ITM2C
prazosin-5416	2/100	0.197972	0.467314	0.369318	0.724338	-0.28795	0.466372	IFI27;IFI6
parthenolide-1736	2/100	0.197972	0.467314	0.369318	0.724338	-0.28667	0.4643	SPP1;CD14
pirinixic acid-487	2/100	0.197972	0.467314	0.369318	0.724338	-0.28514	0.46183	ID3;DPEP1
pioglitazone-5972	2/100	0.197972	0.467314	0.369318	0.724338	-0.28443	0.460679	FOS;ITM2C
Prestwick-1083-6357	2/100	0.197972	0.467314	0.369318	0.724338	-0.28326	0.458772	CDH3;TSPAN1
prilocaine-4749	2/100	0.197972	0.467314	0.369318	0.724338	-0.28247	0.457492	QPCT;TFF2
PNU-0230031-3629	2/100	0.197972	0.467314	0.369318	0.724338	-0.28221	0.457071	ID1;ITM2C
PF-00539758-00-6416	2/100	0.197972	0.467314	0.369318	0.724338	-0.28142	0.455799	TFF2;CD14
phenylpropanolamine-5298	2/100	0.197972	0.467314	0.369318	0.724338	-0.27997	0.453456	IFI6;ID3
piribedil-6333	2/100	0.197972	0.467314	0.369318	0.724338	-0.27948	0.452649	LGALS3BP;TSPAN1
piperacetazine-5834	2/100	0.197972	0.467314	0.369318	0.724338	-0.2774	0.449288	CXCL1;TSPAN1
PHA-00665752-6578	2/100	0.197972	0.467314	0.369318	0.724338	-0.27707	0.448751	PLP2;CTSD
pivmecillinam-7312	2/100	0.197972	0.467314	0.369318	0.724338	-0.27656	0.447927	IFI6;TGFBI
PHA-00846566E-7086	2/100	0.197972	0.467314	0.369318	0.724338	-0.27623	0.447391	GPX2;IGFBP2
PNU-0230031-4754	2/100	0.197972	0.467314	0.369318	0.724338	-0.2758	0.4467	IFI6;TFF2
pioglitazone-7083	2/100	0.197972	0.467314	0.369318	0.724338	-0.27356	0.443066	S100A4;TSPAN1
pimozide-1562	2/100	0.197972	0.467314	0.369318	0.724338	-0.26747	0.433204	S100A6;HSPB1
proadifen-2707	2/100	0.197972	0.467314	0.369318	0.724338	-0.26706	0.432539	CKB;LY6E
Prestwick-665-2186	2/100	0.197972	0.467314	0.369318	0.724338	-0.26591	0.430684	IFI6;CKB
pyrimethamine-4779	2/100	0.197972	0.467314	0.369318	0.724338	-0.26562	0.430201	ID1;LY6E
pramocaine-4368	2/100	0.197972	0.467314	0.369318	0.724338	-0.26461	0.428564	IFI27;PGM1
prilocaine-2314	2/100	0.197972	0.467314	0.369318	0.724338	-0.26385	0.427341	C3;TFF2

pentetrazol-1408	2/100	0.197972	0.467314	0.369318	0.724338	-0.26301	0.425974	IFITM3;PFN1
Prestwick-981-6504	2/100	0.197972	0.467314	0.369318	0.724338	-0.26248	0.425116	RCN1;SECTM1
prednicarbate-5544	2/100	0.197972	0.467314	0.369318	0.724338	-0.26191	0.424196	RNF43;QPCT
prazosin-3098	2/100	0.197972	0.467314	0.369318	0.724338	-0.26044	0.421814	IFI6;LY6E
prednisone-4400	2/100	0.197972	0.467314	0.369318	0.724338	-0.25971	0.420636	IFITM1;IFI27
praziquantel-3189	2/100	0.197972	0.467314	0.369318	0.724338	-0.2589	0.41932	BMP4;LYZ
Prestwick-675-2187	2/100	0.197972	0.467314	0.369318	0.724338	-0.2584	0.418518	CTSD;LY6E
Prestwick-1083-3538	2/100	0.197972	0.467314	0.369318	0.724338	-0.25771	0.417395	LGALS3BP;BMP4
primidone-3065	2/100	0.197972	0.467314	0.369318	0.724338	-0.25762	0.417251	IGFBP2;TFF2
propofol-3048	2/100	0.197972	0.467314	0.369318	0.724338	-0.2535	0.410569	CCL20;IGFBP2
proxymetacaine-5433	2/100	0.197972	0.467314	0.369318	0.724338	-0.25078	0.406173	IFI6;S100A4
probucol-3223	2/100	0.197972	0.467314	0.369318	0.724338	-0.25043	0.405608	GPX2;LYZ
Prestwick-1103-6019	2/100	0.197972	0.467314	0.369318	0.724338	-0.24978	0.404549	IFITM1;IFI6
Prestwick-682-4984	2/100	0.197972	0.467314	0.369318	0.724338	-0.24886	0.403062	IFI27;IFI6
Prestwick-665-6041	2/100	0.197972	0.467314	0.369318	0.724338	-0.2458	0.398109	GPX2;S100A4
Prestwick-685-4705	2/100	0.197972	0.467314	0.369318	0.724338	-0.24397	0.395147	TMPRSS3;S100A4
pyrvinium-6339	2/100	0.197972	0.467314	0.369318	0.724338	-0.24217	0.392222	CDH3;TSPAN1
procainamide-2618	2/100	0.197972	0.467314	0.369318	0.724338	-0.24152	0.391172	PDIA3;TGFBI
Prestwick-981-7464	2/100	0.197972	0.467314	0.369318	0.724338	-0.23946	0.38783	GPX2;TMPRSS3
quinpirole-7481	2/100	0.197972	0.467314	0.369318	0.724338	-0.23907	0.387209	LGALS3BP;AP1S1
piperine-4247	2/100	0.197972	0.467314	0.369318	0.724338	-0.2374	0.384494	SECTM1;PSMB9
propoxycaine-6161	2/100	0.197972	0.467314	0.369318	0.724338	-0.23723	0.384225	TSPAN13;CCL20
Prestwick-920-3118	2/100	0.197972	0.467314	0.369318	0.724338	-0.23646	0.382977	SLC12A2;TCN1
protoveratrine A-4963	2/100	0.197972	0.467314	0.369318	0.724338	-0.23507	0.38072	IFI27;IFI6
procarbazine-5452	2/100	0.197972	0.467314	0.369318	0.724338	-0.23427	0.379431	TMPRSS3;IFI6
prilocaine-2352	2/100	0.197972	0.467314	0.369318	0.724338	-0.23404	0.379066	QPCT;ITGAV
SB-202190-7054	2/100	0.197972	0.467314	0.369318	0.724338	-0.23344	0.378092	RNF43;FOS
probucol-592	2/100	0.197972	0.467314	0.369318	0.724338	-0.23192	0.375624	GPX2;DPEP1
procyclidine-3330	2/100	0.197972	0.467314	0.369318	0.724338	-0.23045	0.373238	LGALS3BP;BMP4
quinethazone-4351	2/100	0.197972	0.467314	0.369318	0.724338	-0.22972	0.372057	SLPI;IFI27
Prestwick-674-2179	2/100	0.197972	0.467314	0.369318	0.724338	-0.229	0.370887	SLPI;LY6E
racecadotril-5755	2/100	0.197972	0.467314	0.369318	0.724338	-0.22752	0.368497	GPX2;IFI6
procainamide-4602	2/100	0.197972	0.467314	0.369318	0.724338	-0.22698	0.367626	IGFBP2;TSPAN1
roxarsone-5470	2/100	0.197972	0.467314	0.369318	0.724338	-0.22461	0.36379	LGALS3BP;RNF43
profenamine-5296	2/100	0.197972	0.467314	0.369318	0.724338	-0.22414	0.363026	CEACAM6;AP1S1
pronetalol-7322	2/100	0.197972	0.467314	0.369318	0.724338	-0.22399	0.362774	BMP4;ISG15
pyrantel-5513	2/100	0.197972	0.467314	0.369318	0.724338	-0.22177	0.359178	TMPRSS3;TSPAN1

promazine-2173	2/100	0.197972	0.467314	0.369318	0.724338	-0.21887	0.354486	IFITM3;LY6E
puromycin-3310	2/100	0.197972	0.467314	0.369318	0.724338	-0.21575	0.349431	RPS14P3;PRSS23
raloxifene-5759	2/100	0.197972	0.467314	0.369318	0.724338	-0.21182	0.343072	IFI6;ID3
Prestwick-675-7381	2/100	0.197972	0.467314	0.369318	0.724338	-0.21156	0.342651	LGALS3BP;MMP1
proxymetacaine-6332	2/100	0.197972	0.467314	0.369318	0.724338	-0.21135	0.342302	PDIA3;ID1
Prestwick-984-4948	2/100	0.197972	0.467314	0.369318	0.724338	-0.21113	0.341949	IFI27;IFI6
raloxifene-3480	2/100	0.197972	0.467314	0.369318	0.724338	-0.20923	0.33888	TMPRSS3;S100A4
Prestwick-983-3141	2/100	0.197972	0.467314	0.369318	0.724338	-0.20362	0.329782	IFITM3;ANXA3
quinidine-3191	2/100	0.197972	0.467314	0.369318	0.724338	-0.20327	0.329221	TMPRSS3;LYZ
promazine-4308	2/100	0.197972	0.467314	0.369318	0.724338	-0.20056	0.324834	IFI6;SOX9
sitosterol-7332	2/100	0.197972	0.467314	0.369318	0.724338	-0.20039	0.324551	LGALS3BP;BMP4
quinethazone-3793	2/100	0.197972	0.467314	0.369318	0.724338	-0.19801	0.3207	C3;FOS
solasodine-4305	2/100	0.197972	0.467314	0.369318	0.724338	-0.19512	0.316018	IFITM1;IFI6
rilmenidine-5532	2/100	0.197972	0.467314	0.369318	0.724338	-0.19483	0.315549	RNF43;TMPRSS3
ranitidine-5425	2/100	0.197972	0.467314	0.369318	0.724338	-0.19443	0.314898	S100A4;TGFBI
SC-560-6865	2/100	0.197972	0.467314	0.369318	0.724338	-0.19147	0.310118	C3;SPINK1
spiramycin-3762	2/100	0.197972	0.467314	0.369318	0.724338	-0.191	0.309346	BMP4;MMP3
rolitetracycline-3369	2/100	0.197972	0.467314	0.369318	0.724338	-0.19075	0.308947	SLPI;SECTM1
propylthiouracil-4157	2/100	0.197972	0.467314	0.369318	0.724338	-0.19046	0.308478	AP1S1;TFF2
pyrvinium-978	2/100	0.197972	0.467314	0.369318	0.724338	-0.19036	0.308307	BMP4;AP1S1
resveratrol-5509	2/100	0.197972	0.467314	0.369318	0.724338	-0.18922	0.306459	RNF43;TGFBI
ribostamycin-2705	2/100	0.197972	0.467314	0.369318	0.724338	-0.18889	0.305924	TSPAN13;QPCT
succinylsulfathiazole-2166	2/100	0.197972	0.467314	0.369318	0.724338	-0.18866	0.305567	CTSD;LY6E
ranitidine-6324	2/100	0.197972	0.467314	0.369318	0.724338	-0.18836	0.305082	PDIA3;S100A4
resveratrol-1715	2/100	0.197972	0.467314	0.369318	0.724338	-0.18752	0.303709	TMEM123;SPP1
rifampicin-4126	2/100	0.197972	0.467314	0.369318	0.724338	-0.18715	0.303112	IFITM1;TCN1
raloxifene-6235	2/100	0.197972	0.467314	0.369318	0.724338	-0.18631	0.301748	IFNGR2;ISG15
ranitidine-2251	2/100	0.197972	0.467314	0.369318	0.724338	-0.1863	0.30173	RNF43;TFF2
raubasine-5459	2/100	0.197972	0.467314	0.369318	0.724338	-0.18477	0.29926	TMPRSS3;TGFBI
ramipril-6150	2/100	0.197972	0.467314	0.369318	0.724338	-0.18309	0.296541	TSPAN13;ANXA3
riboflavin-2760	2/100	0.197972	0.467314	0.369318	0.724338	-0.18225	0.29518	GPX2;SOX9
sitosterol-2912	2/100	0.197972	0.467314	0.369318	0.724338	-0.18194	0.294668	TCN1;ID3
semustine-7545	2/100	0.197972	0.467314	0.369318	0.724338	-0.18012	0.291727	CDH3;CTGF
retrorsine-6601	2/100	0.197972	0.467314	0.369318	0.724338	-0.17874	0.28949	ID1;PSMB9
securinine-4493	2/100	0.197972	0.467314	0.369318	0.724338	-0.17816	0.28855	ENC1;CTGF
pyrantel-1413	2/100	0.197972	0.467314	0.369318	0.724338	-0.17777	0.287914	IFITM3;PFN1
roxarsone-2950	2/100	0.197972	0.467314	0.369318	0.724338	-0.1771	0.286835	RCN1;PLCB4

streptomycin-3195	2/100	0.197972	0.467314	0.369318	0.724338	-0.17606	0.285144	S100A4;LYZ
rilmenidine-5107	2/100	0.197972	0.467314	0.369318	0.724338	-0.17598	0.28502	GPX2;IFI6
sirolimus-5204	2/100	0.197972	0.467314	0.369318	0.724338	-0.17501	0.283445	RNF43;IFI6
racecadotril-1782	2/100	0.197972	0.467314	0.369318	0.724338	-0.17414	0.282038	NOP10;ZWINT
santonin-3877	2/100	0.197972	0.467314	0.369318	0.724338	-0.17321	0.280544	C3;RNF43
SB-203580-6915	2/100	0.197972	0.467314	0.369318	0.724338	-0.17098	0.276926	AP1S1;SECTM1
scopolamine N-oxide-6335	2/100	0.197972	0.467314	0.369318	0.724338	-0.17058	0.276279	TSPAN8;SECTM1
pyrimethamine-1974	2/100	0.197972	0.467314	0.369318	0.724338	-0.16978	0.274975	C3;CKS2
sirolimus-7001	2/100	0.197972	0.467314	0.369318	0.724338	-0.16963	0.27474	BMP4;GPX2
risperidone-2947	2/100	0.197972	0.467314	0.369318	0.724338	-0.16825	0.272509	C3;IFI6
ricinine-5725	2/100	0.197972	0.467314	0.369318	0.724338	-0.16721	0.27082	GPX2;TFF2
ritodrine-1280	2/100	0.197972	0.467314	0.369318	0.724338	-0.16522	0.267588	IFITM3;SLPI
sertaconazole-6811	2/100	0.197972	0.467314	0.369318	0.724338	-0.1644	0.26626	IFI27;IFI6
rosiglitazone-5230	2/100	0.197972	0.467314	0.369318	0.724338	-0.16387	0.265402	RCN1;MMP3
sulfafurazole-4661	2/100	0.197972	0.467314	0.369318	0.724338	-0.16292	0.263876	IL32;SPINK1
tanespimycin-1005	2/100	0.197972	0.467314	0.369318	0.724338	-0.16121	0.261104	ID1;IER2
sirolimus-6967	2/100	0.197972	0.467314	0.369318	0.724338	-0.15922	0.257885	LGALS3BP;S100A4
sulfadoxine-3547	2/100	0.197972	0.467314	0.369318	0.724338	-0.15906	0.257617	PDIA3;LGALS3BP
sotalol-4079	2/100	0.197972	0.467314	0.369318	0.724338	-0.15795	0.255818	LUM;IFI6
sirolimus-6958	2/100	0.197972	0.467314	0.369318	0.724338	-0.15716	0.254547	IFNGR2;S100A4
securinine-2729	2/100	0.197972	0.467314	0.369318	0.724338	-0.1547	0.25056	ENC1;PSMB9
spiramycin-3938	2/100	0.197972	0.467314	0.369318	0.724338	-0.15022	0.243306	BMP4;AP1S1
solasodine-3749	2/100	0.197972	0.467314	0.369318	0.724338	-0.14998	0.242918	TCN1;MMP3
SR-95531-3253	2/100	0.197972	0.467314	0.369318	0.724338	-0.14977	0.242577	RPS14P3;TGFBI
sirolimus-1080	2/100	0.197972	0.467314	0.369318	0.724338	-0.14928	0.241774	IGFBP2;PGM1
sulpiride-1467	2/100	0.197972	0.467314	0.369318	0.724338	-0.14655	0.23735	GPX2;TSPAN1
SC-58125-254	2/100	0.197972	0.467314	0.369318	0.724338	-0.14594	0.236361	MMP3;SPP1
sulfinpyrazone-1574	2/100	0.197972	0.467314	0.369318	0.724338	-0.14501	0.234863	SLPI;CTSH
sulfafurazole-3218	2/100	0.197972	0.467314	0.369318	0.724338	-0.14489	0.234671	LYZ;TSPAN1
rofecoxib-166	2/100	0.197972	0.467314	0.369318	0.724338	-0.14465	0.234281	APP;DSG2
S-propranolol-2961	2/100	0.197972	0.467314	0.369318	0.724338	-0.1441	0.233396	IFI6;LY6E
sulfadimidine-3765	2/100	0.197972	0.467314	0.369318	0.724338	-0.1434	0.232248	BMP4;RNF43
semustine-7492	2/100	0.197972	0.467314	0.369318	0.724338	-0.14322	0.231965	BMP4;CTGF
SC-560-6913	2/100	0.197972	0.467314	0.369318	0.724338	-0.14285	0.231361	PLP2;TSPAN1
sparteine-2790	2/100	0.197972	0.467314	0.369318	0.724338	-0.14235	0.230549	PDIA3;GPX2
sirolimus-1162	2/100	0.197972	0.467314	0.369318	0.724338	-0.14203	0.230037	TSPAN13;IFI6
skimmianine-6066	2/100	0.197972	0.467314	0.369318	0.724338	-0.14052	0.227597	AP1S1;TFF2

sirolimus-1183	2/100	0.197972	0.467314	0.369318	0.724338	-0.13735	0.222454	IFI6;CKB
SR-95639A-4977	2/100	0.197972	0.467314	0.369318	0.724338	-0.13711	0.222066	IFI27;ISG15
sulfamerazine-4740	2/100	0.197972	0.467314	0.369318	0.724338	-0.13358	0.216358	PDIA3;IFI6
sulindac-5528	2/100	0.197972	0.467314	0.369318	0.724338	-0.13343	0.216099	GPX2;TMPRSS3
sulfafurazole-1603	2/100	0.197972	0.467314	0.369318	0.724338	-0.13021	0.210897	SLC12A2;SLPI
resveratrol-622	2/100	0.197972	0.467314	0.369318	0.724338	-0.13013	0.210767	ID1;CKS2
STOCK1N-35874-6583	2/100	0.197972	0.467314	0.369318	0.724338	-0.12904	0.209004	C3;ENC1
STOCK1N-35696-6564	2/100	0.197972	0.467314	0.369318	0.724338	-0.12902	0.208973	GPX2;IFI6
talampicillin-7254	2/100	0.197972	0.467314	0.369318	0.724338	-0.12854	0.208188	LCN2;CTSD
sulfamethoxypyridazine-3609	2/100	0.197972	0.467314	0.369318	0.724338	-0.12588	0.203875	RPS14P3;ITM2C
tanespimycin-5203	2/100	0.197972	0.467314	0.369318	0.724338	-0.12412	0.201029	ID1;IER2
sulfadiazine-5523	2/100	0.197972	0.467314	0.369318	0.724338	-0.12358	0.200156	RNF43;TSPAN1
sirolimus-362	2/100	0.197972	0.467314	0.369318	0.724338	-0.12322	0.199576	IFITM3;RCN1
sulfasalazine-6346	2/100	0.197972	0.467314	0.369318	0.724338	-0.12248	0.198365	CDH3;TSPAN8
resveratrol-595	2/100	0.197972	0.467314	0.369318	0.724338	-0.12216	0.197848	RNF43;CKS2
tanespimycin-4430	2/100	0.197972	0.467314	0.369318	0.724338	-0.12182	0.197311	ID1;ID3
solanine-2152	2/100	0.197972	0.467314	0.369318	0.724338	-0.11966	0.193807	C3;CTSD
tanespimycin-1056	2/100	0.197972	0.467314	0.369318	0.724338	-0.11897	0.19269	ID1;IER2
rofecoxib-251	2/100	0.197972	0.467314	0.369318	0.724338	-0.11789	0.190934	IFI27;SPP1
sulfaquinoxaline-6788	2/100	0.197972	0.467314	0.369318	0.724338	-0.11784	0.190853	BMP4;RCN1
staurosporine-508	2/100	0.197972	0.467314	0.369318	0.724338	-0.11643	0.188578	ID3;CXCL1
tanespimycin-5958	2/100	0.197972	0.467314	0.369318	0.724338	-0.11495	0.186182	IL32;IFI6
terbutaline-5764	2/100	0.197972	0.467314	0.369318	0.724338	-0.11378	0.184286	GPX2;CTGF
suloctidil-2651	2/100	0.197972	0.467314	0.369318	0.724338	-0.11282	0.182722	BMP4;RCN1
terguride-4633	2/100	0.197972	0.467314	0.369318	0.724338	-0.10956	0.177442	SPINK1;OLFM4
suxibuzone-5806	2/100	0.197972	0.467314	0.369318	0.724338	-0.10863	0.175939	PDIA3;ITM2C
tanespimycin-5223	2/100	0.197972	0.467314	0.369318	0.724338	-0.10749	0.174102	ID1;IER2
tanespimycin-5586	2/100	0.197972	0.467314	0.369318	0.724338	-0.10723	0.17368	ID1;IER2
tamoxifen-2212	2/100	0.197972	0.467314	0.369318	0.724338	-0.10641	0.172339	TMPRSS3;PRSS23
sulfanilamide-6810	2/100	0.197972	0.467314	0.369318	0.724338	-0.1047	0.169581	IFI27;IFI6
sulfamethoxazole-2335	2/100	0.197972	0.467314	0.369318	0.724338	-0.10256	0.166116	C3;QPCT
tanespimycin-5222	2/100	0.197972	0.467314	0.369318	0.724338	-0.10104	0.163644	ID1;IER2
staurosporine-312	2/100	0.197972	0.467314	0.369318	0.724338	-0.10008	0.162099	ID1;CKS2
tetroquinone-4078	2/100	0.197972	0.467314	0.369318	0.724338	-0.10006	0.162066	LUM;IFI6
tanespimycin-1044	2/100	0.197972	0.467314	0.369318	0.724338	-0.09742	0.157779	ID1;IER2
tanespimycin-4449	2/100	0.197972	0.467314	0.369318	0.724338	-0.09619	0.155788	ID1;TGFBI
sulfadimethoxine-7400	2/100	0.197972	0.467314	0.369318	0.724338	-0.09457	0.153165	IFI6;TGFBI

tanespimycin-381	2/100	0.197972	0.467314	0.369318	0.724338	-0.0934	0.151281	PIGR;IER2
testosterone-2649	2/100	0.197972	0.467314	0.369318	0.724338	-0.09266	0.150072	PDIA3;BMP4
sulfadiazine-1688	2/100	0.197972	0.467314	0.369318	0.724338	-0.09259	0.149956	IGFBP2;S100A4
tanespimycin-5585	2/100	0.197972	0.467314	0.369318	0.724338	-0.09252	0.149852	ID1;IER2
tanespimycin-6926	2/100	0.197972	0.467314	0.369318	0.724338	-0.09112	0.147578	ID1;IER2
sulindac-1857	2/100	0.197972	0.467314	0.369318	0.724338	-0.09032	0.146287	C3;LY6E
tanespimycin-5566	2/100	0.197972	0.467314	0.369318	0.724338	-0.08814	0.142752	ID1;IER2
tanespimycin-1063	2/100	0.197972	0.467314	0.369318	0.724338	-0.08657	0.140206	ID1;IER2
tanespimycin-1064	2/100	0.197972	0.467314	0.369318	0.724338	-0.08625	0.139695	ID1;IER2
terbutaline-3202	2/100	0.197972	0.467314	0.369318	0.724338	-0.08384	0.135787	GPX2;LYZ
tanespimycin-5578	2/100	0.197972	0.467314	0.369318	0.724338	-0.08381	0.135735	ID1;IER2
tanespimycin-5914	2/100	0.197972	0.467314	0.369318	0.724338	-0.08293	0.134321	ID1;TFF2
tanespimycin-6985	2/100	0.197972	0.467314	0.369318	0.724338	-0.08267	0.13389	ID1;IER2
terfenadine-6823	2/100	0.197972	0.467314	0.369318	0.724338	-0.08246	0.133556	IFI27;IFI6
tanespimycin-5215	2/100	0.197972	0.467314	0.369318	0.724338	-0.08228	0.133263	ID1;IER2
testosterone-5271	2/100	0.197972	0.467314	0.369318	0.724338	-0.0807	0.130697	IFI27;IFI6
testosterone-1295	2/100	0.197972	0.467314	0.369318	0.724338	-0.07826	0.126757	ANXA3;ISG15
sulfaphenazole-5507	2/100	0.197972	0.467314	0.369318	0.724338	-0.07791	0.126185	LGALS3BP;RNF43
tocainide-4838	2/100	0.197972	0.467314	0.369318	0.724338	-0.07538	0.122092	RCN1;SECTM1
tanespimycin-5953	2/100	0.197972	0.467314	0.369318	0.724338	-0.07276	0.117847	ID1;IER2
suramin sodium-7524	2/100	0.197972	0.467314	0.369318	0.724338	-0.07184	0.116362	SLPI;S100A4
tanespimycin-450	2/100	0.197972	0.467314	0.369318	0.724338	-0.07038	0.113991	ID1;ID3
tetracaine-2888	2/100	0.197972	0.467314	0.369318	0.724338	-0.06846	0.110888	TFF2;SECTM1
thioproperazine-2236	2/100	0.197972	0.467314	0.369318	0.724338	-0.06679	0.108173	GPX2;AP1S1
thiamphenicol-7274	2/100	0.197972	0.467314	0.369318	0.724338	-0.06499	0.105258	MMP1;TGFBI
tanespimycin-831	2/100	0.197972	0.467314	0.369318	0.724338	-0.06275	0.101629	ID1;IER2
thioridazine-5921	2/100	0.197972	0.467314	0.369318	0.724338	-0.06209	0.100564	IL32;RNF43
tanespimycin-1206	2/100	0.197972	0.467314	0.369318	0.724338	-0.06059	0.098131	CCL20;IER2
tanespimycin-1643	2/100	0.197972	0.467314	0.369318	0.724338	-0.05992	0.097053	ID1;IER2
tinidazole-4370	2/100	0.197972	0.467314	0.369318	0.724338	-0.05989	0.097001	MMP3;ITM2C
tolmetin-4167	2/100	0.197972	0.467314	0.369318	0.724338	-0.05789	0.093754	TCN1;AP1S1
tenoxicam-4182	2/100	0.197972	0.467314	0.369318	0.724338	-0.05774	0.093515	C3;AP1S1
tiapride-3663	2/100	0.197972	0.467314	0.369318	0.724338	-0.05582	0.090406	C3;TSPAN8
tanespimycin-6966	2/100	0.197972	0.467314	0.369318	0.724338	-0.05494	0.088985	ID1;IER2
tanespimycin-947	2/100	0.197972	0.467314	0.369318	0.724338	-0.05436	0.088036	ID1;IER2
tanespimycin-986	2/100	0.197972	0.467314	0.369318	0.724338	-0.05401	0.087472	ID1;IER2
timolol-5280	2/100	0.197972	0.467314	0.369318	0.724338	-0.05258	0.085161	MMP3;IFI6

tetracycline-6233	2/100	0.197972	0.467314	0.369318	0.724338	-0.05213	0.084424	LGALS3BP;S100A4
tanespimycin-1651	2/100	0.197972	0.467314	0.369318	0.724338	-0.05148	0.08338	ID1;IER2
trichostatin A-1175	2/100	0.197972	0.467314	0.369318	0.724338	-0.04979	0.080638	IFNGR2;CD14
tanespimycin-916	2/100	0.197972	0.467314	0.369318	0.724338	-0.04932	0.079884	ID1;IER2
testosterone-5636	2/100	0.197972	0.467314	0.369318	0.724338	-0.04892	0.079226	TFF2;S100A4
tetracaine-7473	2/100	0.197972	0.467314	0.369318	0.724338	-0.04858	0.078685	LGALS3BP;TMPRSS3
thiamphenicol-1826	2/100	0.197972	0.467314	0.369318	0.724338	-0.04852	0.078584	GPX2;IFI27
tanespimycin-1631	2/100	0.197972	0.467314	0.369318	0.724338	-0.0481	0.077898	ID1;IER2
terazosin-7187	2/100	0.197972	0.467314	0.369318	0.724338	-0.0465	0.075313	LGALS3BP;IFNGR2
todralazine-5087	2/100	0.197972	0.467314	0.369318	0.724338	-0.04635	0.075062	LGALS3BP;TSPAN1
thioridazine-5227	2/100	0.197972	0.467314	0.369318	0.724338	-0.04443	0.071964	BMP4;IFI6
tiratricol-7011	2/100	0.197972	0.467314	0.369318	0.724338	-0.04371	0.070802	BMP4;S100A4
tetraethylenepentamine-574	2/100	0.197972	0.467314	0.369318	0.724338	-0.04283	0.069372	PIGR;PDZK1IP1
tracazolate-4964	2/100	0.197972	0.467314	0.369318	0.724338	-0.04019	0.065098	IFI27;IFI6
terbutaline-6240	2/100	0.197972	0.467314	0.369318	0.724338	-0.03923	0.063543	IFNGR2;TGFBI
tanespimycin-6986	2/100	0.197972	0.467314	0.369318	0.724338	-0.03867	0.062636	ID1;IER2
thiamazole-3898	2/100	0.197972	0.467314	0.369318	0.724338	-0.03623	0.058685	C3;SECTM1
timolol-4685	2/100	0.197972	0.467314	0.369318	0.724338	-0.0357	0.057827	IL32;OLFM4
timolol-6483	2/100	0.197972	0.467314	0.369318	0.724338	-0.03557	0.057618	PDIA3;TMPRSS3
trichostatin A-2450	2/100	0.197972	0.467314	0.369318	0.724338	-0.03481	0.056381	GPX2;IFI6
terguride-5694	2/100	0.197972	0.467314	0.369318	0.724338	-0.03276	0.053058	IFITM1;IFI27
tiratricol-1412	2/100	0.197972	0.467314	0.369318	0.724338	-0.03114	0.050432	PDIA3;CTSD
ticlopidine-1895	2/100	0.197972	0.467314	0.369318	0.724338	-0.02988	0.048396	ITGAV;ITM2C
triprolidine-7008	2/100	0.197972	0.467314	0.369318	0.724338	-0.02931	0.047469	IFNGR2;AP1S1
tolmetin-3347	2/100	0.197972	0.467314	0.369318	0.724338	-0.02816	0.045617	BMP4;TFF2
thiamazole-3815	2/100	0.197972	0.467314	0.369318	0.724338	-0.02811	0.045532	C3;PSMB9
trimethadione-4165	2/100	0.197972	0.467314	0.369318	0.724338	-0.02491	0.040345	GPX2;AP1S1
trichlormethiazide-2998	2/100	0.197972	0.467314	0.369318	0.724338	-0.02421	0.039203	ANXA3;LY6E
valproic acid-5237	2/100	0.197972	0.467314	0.369318	0.724338	-0.02371	0.0384	TMPRSS3;MMP3
trichostatin A-3058	2/100	0.197972	0.467314	0.369318	0.724338	-0.02228	0.036083	GPX2;TFF2
trichostatin A-2375	2/100	0.197972	0.467314	0.369318	0.724338	-0.02132	0.034527	IFITM3;CD14
trimethoprim-3678	2/100	0.197972	0.467314	0.369318	0.724338	-0.01827	0.029584	BMP4;MMP3
tanespimycin-998	2/100	0.197972	0.467314	0.369318	0.724338	-0.01691	0.027381	ID1;IER2
trichostatin A-3872	2/100	0.197972	0.467314	0.369318	0.724338	-0.01643	0.026611	C3;TMPRSS3
torasemide-5476	2/100	0.197972	0.467314	0.369318	0.724338	-0.01643	0.02661	LGALS3BP;IFI6
trichlormethiazide-3337	2/100	0.197972	0.467314	0.369318	0.724338	-0.01598	0.025875	BMP4;RNF43
tretinoin-1152	2/100	0.197972	0.467314	0.369318	0.724338	-0.01408	0.022801	PLCB4;ANXA3

trichostatin A-2694	2/100	0.197972	0.467314	0.369318	0.724338	-0.01386	0.022452	IFNGR2;CD14
thapsigargin-7100	2/100	0.197972	0.467314	0.369318	0.724338	-0.01312	0.021253	BMP4;MMP3
trimethoprim-7377	2/100	0.197972	0.467314	0.369318	0.724338	-0.01303	0.02111	IGFBP2;TGFBI
tetracaine-1739	2/100	0.197972	0.467314	0.369318	0.724338	-0.01216	0.019691	GPX2;CTSD
trichostatin A-3566	2/100	0.197972	0.467314	0.369318	0.724338	-0.00808	0.013088	LGALS3BP;RNF43
tranylcypromine-1417	2/100	0.197972	0.467314	0.369318	0.724338	-0.00661	0.010712	RPS14P3;S100P
tretinoin-2671	2/100	0.197972	0.467314	0.369318	0.724338	-0.00625	0.01012	PLCB4;ANXA3
valdecoxib-6403	2/100	0.197972	0.467314	0.369318	0.724338	-0.00528	0.008549	RCN1;QPCT
valproic acid-6941	2/100	0.197972	0.467314	0.369318	0.724338	-0.00368	0.00596	LGALS3BP;IFITM1
valproic acid-5219	2/100	0.197972	0.467314	0.369318	0.724338	-0.00343	0.005556	IFI6;ISG15
tocainide-7351	2/100	0.197972	0.467314	0.369318	0.724338	-0.00258	0.004182	GPX2;IFI6
trifluridine-6136	2/100	0.197972	0.467314	0.369318	0.724338	-0.00173	0.002805	CCL20;IFI6
16,16-dimethylprostaglandin E2-6597	1/100	0.561955	0.561955	0.724338	0.724338	0.000253	-0.00015	IFI6
trichostatin A-1421	2/100	0.197972	0.467314	0.369318	0.724338	0.00158	-0.00256	RPS14P3;CD14
valproic acid-5600	2/100	0.197972	0.467314	0.369318	0.724338	0.001653	-0.00268	RPS14P3;SECTM1
valproic acid-6199	2/100	0.197972	0.467314	0.369318	0.724338	0.002209	-0.00358	SPP1;CD14
trioxysalen-2516	2/100	0.197972	0.467314	0.369318	0.724338	0.003943	-0.00639	CCL20;ANXA3
trichostatin A-5017	2/100	0.197972	0.467314	0.369318	0.724338	0.004348	-0.00704	ID1;TSPAN1
vinblastine-7551	2/100	0.197972	0.467314	0.369318	0.724338	0.006554	-0.01062	TUBB;IFI6
tranylcypromine-7293	2/100	0.197972	0.467314	0.369318	0.724338	0.007094	-0.01149	PDIA3;IFI6
ursodeoxycholic acid-3105	2/100	0.197972	0.467314	0.369318	0.724338	0.009503	-0.01539	IFI6;LY6E
verapamil-5387	2/100	0.197972	0.467314	0.369318	0.724338	0.009736	-0.01577	IFI27;IFI6
trichostatin A-1835	2/100	0.197972	0.467314	0.369318	0.724338	0.01007	-0.01631	IFITM3;ISG15
0198306-0000-7064	1/100	0.561955	0.561955	0.724338	0.724338	0.034658	-0.01997	PRSS23
0317956-0000-3777	1/100	0.561955	0.561955	0.724338	0.724338	0.035042	-0.0202	RNF43
vanoxerine-5702	2/100	0.197972	0.467314	0.369318	0.724338	0.013591	-0.02201	QPCT;S100A4
trichostatin A-1400	2/100	0.197972	0.467314	0.369318	0.724338	0.013617	-0.02206	IFITM3;CD14
0173570-0000-4712	1/100	0.561955	0.561955	0.724338	0.724338	0.040074	-0.0231	SPINK1
trichostatin A-2035	2/100	0.197972	0.467314	0.369318	0.724338	0.015022	-0.02433	RPS14P3;CD14
(+)-chelidonine-6236	1/100	0.561955	0.561955	0.724338	0.724338	0.043	-0.02478	TUBB
15-delta prostaglandin J2-6948	1/100	0.561955	0.561955	0.724338	0.724338	0.04446	-0.02562	IFNGR2
tropicamide-4744	2/100	0.197972	0.467314	0.369318	0.724338	0.01588	-0.02572	IFI6;TFF2
trichostatin A-6171	2/100	0.197972	0.467314	0.369318	0.724338	0.015902	-0.02576	CCL20;ID1
trimipramine-4163	2/100	0.197972	0.467314	0.369318	0.724338	0.016461	-0.02666	TCN1;AP1S1
triamcinolone-2241	2/100	0.197972	0.467314	0.369318	0.724338	0.016629	-0.02693	AP1S1;TSPAN1
ursolic acid-5825	2/100	0.197972	0.467314	0.369318	0.724338	0.016664	-0.02699	LGALS3BP;CDH3
vanoxerine-3240	2/100	0.197972	0.467314	0.369318	0.724338	0.017348	-0.0281	BMP4;GPX2

tretinoin-991	2/100	0.197972	0.467314	0.369318	0.724338	0.018626	-0.03017	BMP4;S100A4
tolazoline-2000	2/100	0.197972	0.467314	0.369318	0.724338	0.019052	-0.03086	IFITM3;HSPB1
0175029-0000-3691	1/100	0.561955	0.561955	0.724338	0.724338	0.053701	-0.03095	SOX9
5186223-885	1/100	0.561955	0.561955	0.724338	0.724338	0.054047	-0.03115	RNF43
(-)-MK-801-3081	1/100	0.561955	0.561955	0.724338	0.724338	0.054317	-0.0313	LY6E
tolnaftate-2001	2/100	0.197972	0.467314	0.369318	0.724338	0.019902	-0.03223	IFITM3;S100A10
velnacrine-6651	2/100	0.197972	0.467314	0.369318	0.724338	0.020037	-0.03245	MMP3;SECTM1
troglitazone-5229	2/100	0.197972	0.467314	0.369318	0.724338	0.02103	-0.03406	BMP4;IFI6
2,6-dimethylpiperidine-6049	1/100	0.561955	0.561955	0.724338	0.724338	0.059645	-0.03438	MMP3
(+/-)-catechin-4837	1/100	0.561955	0.561955	0.724338	0.724338	0.059979	-0.03457	GPX2
0175029-0000-3694	1/100	0.561955	0.561955	0.724338	0.724338	0.060082	-0.03463	CTGF
0316684-0000-7057	1/100	0.561955	0.561955	0.724338	0.724338	0.062708	-0.03614	CTGF
2-aminobenzenesulfonamide-6321	1/100	0.561955	0.561955	0.724338	0.724338	0.063512	-0.0366	LY6E
valproic acid-4446	2/100	0.197972	0.467314	0.369318	0.724338	0.023498	-0.03806	BMP4;IFI6
5186324-900	1/100	0.561955	0.561955	0.724338	0.724338	0.069608	-0.04012	BMP4
10-methoxyharmalan-5455	1/100	0.561955	0.561955	0.724338	0.724338	0.069958	-0.04032	IFI6
0316684-0000-7098	1/100	0.561955	0.561955	0.724338	0.724338	0.071088	-0.04097	AP1S1
10-methoxyharmalan-1743	1/100	0.561955	0.561955	0.724338	0.724338	0.07125	-0.04106	ISG15
0179445-0000-3633	1/100	0.561955	0.561955	0.724338	0.724338	0.071419	-0.04116	GPX2
15-delta prostaglandin J2-6990	1/100	0.561955	0.561955	0.724338	0.724338	0.072416	-0.04174	TSPAN1
triprolidine-7408	2/100	0.197972	0.467314	0.369318	0.724338	0.026147	-0.04235	ENC1;S100A4
3-acetamidocoumarin-4601	1/100	0.561955	0.561955	0.724338	0.724338	0.074484	-0.04293	GPX2
(-)-atenolol-5325	1/100	0.561955	0.561955	0.724338	0.724338	0.076902	-0.04432	IFI6
trichostatin A-6222	2/100	0.197972	0.467314	0.369318	0.724338	0.027755	-0.04495	IFNGR2;ISG15
3-acetylcoumarin-3382	1/100	0.561955	0.561955	0.724338	0.724338	0.080553	-0.04643	PDIA3
0317956-0000-4331	1/100	0.561955	0.561955	0.724338	0.724338	0.08208	-0.04731	TGFBI
thioridazine-5916	2/100	0.197972	0.467314	0.369318	0.724338	0.029989	-0.04857	PIGR;TFF2
tropicamide-3619	2/100	0.197972	0.467314	0.369318	0.724338	0.030194	-0.0489	RNF43;S100A4
5230742-862	1/100	0.561955	0.561955	0.724338	0.724338	0.086268	-0.04972	BMP4
5114445-901	1/100	0.561955	0.561955	0.724338	0.724338	0.088153	-0.05081	BMP4
3-hydroxy-DL-kynurenine-1300	1/100	0.561955	0.561955	0.724338	0.724338	0.088464	-0.05098	IFITM3
5152487-896	1/100	0.561955	0.561955	0.724338	0.724338	0.08891	-0.05124	TSPAN1
2-aminobenzenesulfonamide-5422	1/100	0.561955	0.561955	0.724338	0.724338	0.08907	-0.05133	QPCT
5155877-6544	1/100	0.561955	0.561955	0.724338	0.724338	0.089914	-0.05182	TSPAN1
0198306-0000-7102	1/100	0.561955	0.561955	0.724338	0.724338	0.092539	-0.05333	MMP1
zalcitabine-7352	2/100	0.197972	0.467314	0.369318	0.724338	0.033155	-0.0537	IFITM1;IFI6
3-hydroxy-DL-kynurenine-5641	1/100	0.561955	0.561955	0.724338	0.724338	0.094504	-0.05447	TFF2

15(S)-15-methylprostaglandin E2-7521	1/100	0.561955	0.561955	0.724338	0.724338	0.094596	-0.05452	ITM2C
15-delta prostaglandin J2-564	1/100	0.561955	0.561955	0.724338	0.724338	0.094631	-0.05454	DYNLL1
6-benzylaminopurine-4748	1/100	0.561955	0.561955	0.724338	0.724338	0.094893	-0.05469	IFI6
trimetazidine-5479	2/100	0.197972	0.467314	0.369318	0.724338	0.034447	-0.05579	RNF43;IFNGR2
10-methoxyharmalan-6355	1/100	0.561955	0.561955	0.724338	0.724338	0.09778	-0.05635	CDH3
11-deoxy-16,16-dimethylprostaglandin E2-7533	1/100	0.561955	0.561955	0.724338	0.724338	0.098355	-0.05669	PDZK1IP1
0297417-0002B-6895	1/100	0.561955	0.561955	0.724338	0.724338	0.099355	-0.05726	BMP4
2-deoxy-D-glucose-344	1/100	0.561955	0.561955	0.724338	0.724338	0.100132	-0.05771	PIGR
15-delta prostaglandin J2-1172	1/100	0.561955	0.561955	0.724338	0.724338	0.101897	-0.05873	IFI6
xylometazoline-2270	2/100	0.197972	0.467314	0.369318	0.724338	0.036783	-0.05958	RNF43;S100A4
alpha-ergocryptine-4374	1/100	0.561955	0.561955	0.724338	0.724338	0.103395	-0.05959	IFI6
zalcitabine-2932	2/100	0.197972	0.467314	0.369318	0.724338	0.037579	-0.06086	ANXA3;IFI6
0297417-0002B-6902	1/100	0.561955	0.561955	0.724338	0.724338	0.106699	-0.06149	ID1
16-phenyltetranorprostaglandin E2-7541	1/100	0.561955	0.561955	0.724338	0.724338	0.108469	-0.06251	IFI6
5109870-904	1/100	0.561955	0.561955	0.724338	0.724338	0.108586	-0.06258	BMP4
0317956-0000-3858	1/100	0.561955	0.561955	0.724338	0.724338	0.110576	-0.06373	TGFBI
7-aminocephalosporanic acid-3258	1/100	0.561955	0.561955	0.724338	0.724338	0.110609	-0.06375	BMP4
vinburnine-7154	2/100	0.197972	0.467314	0.369318	0.724338	0.039853	-0.06455	SECTM1;SOX9
acebutolol-1911	1/100	0.561955	0.561955	0.724338	0.724338	0.112592	-0.06489	PRSS23
7-aminocephalosporanic acid-1322	1/100	0.561955	0.561955	0.724338	0.724338	0.11272	-0.06496	SLC12A2
valproic acid-4433	2/100	0.197972	0.467314	0.369318	0.724338	0.040186	-0.06509	IFI6;TGFBI
5707885-6390	1/100	0.561955	0.561955	0.724338	0.724338	0.113512	-0.06542	TXNIP
6-azathymine-3987	1/100	0.561955	0.561955	0.724338	0.724338	0.11399	-0.0657	MMP3
3-nitropropionic acid-6407	1/100	0.561955	0.561955	0.724338	0.724338	0.114132	-0.06578	MMP3
5149715-890	1/100	0.561955	0.561955	0.724338	0.724338	0.115523	-0.06658	BMP4
5253409-961	1/100	0.561955	0.561955	0.724338	0.724338	0.11636	-0.06706	TMPRSS3
2-aminobenzenesulfonamide-3400	1/100	0.561955	0.561955	0.724338	0.724338	0.116531	-0.06716	AP1S1
acemetacin-7442	1/100	0.561955	0.561955	0.724338	0.724338	0.118269	-0.06816	LGALS3BP
5194442-6599	1/100	0.561955	0.561955	0.724338	0.724338	0.119892	-0.0691	IFI6
acemetacin-6361	1/100	0.561955	0.561955	0.724338	0.724338	0.120027	-0.06918	CDH3
vorinostat-1161	2/100	0.197972	0.467314	0.369318	0.724338	0.043514	-0.07048	IFNGR2;CD14
acetylsalicylsalicylic acid-6778	1/100	0.561955	0.561955	0.724338	0.724338	0.12425	-0.07161	GPX2
acetylsalicylsalicylic acid-2223	1/100	0.561955	0.561955	0.724338	0.724338	0.124641	-0.07184	AP1S1
troleandomycin-1885	2/100	0.197972	0.467314	0.369318	0.724338	0.044637	-0.07229	BACE2;IFI6
acenocoumarol-2240	1/100	0.561955	0.561955	0.724338	0.724338	0.125466	-0.07231	AP1S1
6-bromoindirubin-3'-oxime-6585	1/100	0.561955	0.561955	0.724338	0.724338	0.125572	-0.07237	CTGF
adrenosterone-6486	1/100	0.561955	0.561955	0.724338	0.724338	0.12589	-0.07255	PDIA3

troglitazone-462	2/100	0.197972	0.467314	0.369318	0.724338	0.045052	-0.07297	PRSS23;CTGF
5707885-6433	1/100	0.561955	0.561955	0.724338	0.724338	0.130193	-0.07503	SPINK1
valproic acid-1155	2/100	0.197972	0.467314	0.369318	0.724338	0.047557	-0.07703	IFITM3;TCN1
(-)-isoprenaline-6149	1/100	0.561955	0.561955	0.724338	0.724338	0.13385	-0.07714	SPP1
15-delta prostaglandin J2-6190	1/100	0.561955	0.561955	0.724338	0.724338	0.134227	-0.07736	ANXA3
troglitazone-1232	2/100	0.197972	0.467314	0.369318	0.724338	0.047876	-0.07754	BMP4;CTGF
5194442-6553	1/100	0.561955	0.561955	0.724338	0.724338	0.134971	-0.07779	AP1S1
zomepirac-3454	2/100	0.197972	0.467314	0.369318	0.724338	0.048226	-0.07811	LGALS3BP;S100A4
3-acetamidocoumarin-2941	1/100	0.561955	0.561955	0.724338	0.724338	0.136425	-0.07863	IFI6
0225151-0000-6384	1/100	0.561955	0.561955	0.724338	0.724338	0.136944	-0.07893	SLPI
5224221-956	1/100	0.561955	0.561955	0.724338	0.724338	0.140175	-0.08079	SOX9
5286656-889	1/100	0.561955	0.561955	0.724338	0.724338	0.140583	-0.08102	TMPRSS3
adenosine phosphate-3237	1/100	0.561955	0.561955	0.724338	0.724338	0.140639	-0.08106	RCN1
3-nitropropionic acid-6372	1/100	0.561955	0.561955	0.724338	0.724338	0.141245	-0.0814	AP1S1
acetohexamide-7482	1/100	0.561955	0.561955	0.724338	0.724338	0.144416	-0.08323	AP1S1
15-delta prostaglandin J2-1069	1/100	0.561955	0.561955	0.724338	0.724338	0.145812	-0.08404	IFNGR2
5279552-960	1/100	0.561955	0.561955	0.724338	0.724338	0.145956	-0.08412	RNF43
7-aminocephalosporanic acid-4826	1/100	0.561955	0.561955	0.724338	0.724338	0.147795	-0.08518	S100A4
5182598-976	1/100	0.561955	0.561955	0.724338	0.724338	0.148797	-0.08576	BMP4
6-bromoindirubin-3'-oxime-7048	1/100	0.561955	0.561955	0.724338	0.724338	0.149604	-0.08622	CTGF
albendazole-7164	1/100	0.561955	0.561955	0.724338	0.724338	0.15078	-0.0869	TUBB
5252917-828	1/100	0.561955	0.561955	0.724338	0.724338	0.151159	-0.08712	S100A4
acetylsalicylic acid-984	1/100	0.561955	0.561955	0.724338	0.724338	0.152122	-0.08767	TUBA1A
15-delta prostaglandin J2-446	1/100	0.561955	0.561955	0.724338	0.724338	0.152574	-0.08793	CTGF
xylazine-2788	2/100	0.197972	0.467314	0.369318	0.724338	0.054415	-0.08813	ID3;TSPAN1
tubocurarine chloride-6351	2/100	0.197972	0.467314	0.369318	0.724338	0.05443	-0.08816	CDH3;TSPAN1
acetazolamide-1686	1/100	0.561955	0.561955	0.724338	0.724338	0.153624	-0.08854	PDIA3
amantadine-4806	1/100	0.561955	0.561955	0.724338	0.724338	0.15517	-0.08943	IFI6
acacetin-6044	1/100	0.561955	0.561955	0.724338	0.724338	0.157421	-0.09073	ID1
acetylsalicylic acid-6964	1/100	0.561955	0.561955	0.724338	0.724338	0.158911	-0.09159	TSPAN1
acetylsalicylic acid-4428	1/100	0.561955	0.561955	0.724338	0.724338	0.162054	-0.0934	BMP4
AH-6809-7075	1/100	0.561955	0.561955	0.724338	0.724338	0.162402	-0.0936	IFI6
alverine-2273	1/100	0.561955	0.561955	0.724338	0.724338	0.162427	-0.09361	SECTM1
alpha-estradiol-6930	1/100	0.561955	0.561955	0.724338	0.724338	0.163039	-0.09397	LGALS3BP
aciclovir-5643	1/100	0.561955	0.561955	0.724338	0.724338	0.163064	-0.09398	RNF43
6-benzylaminopurine-3726	1/100	0.561955	0.561955	0.724338	0.724338	0.163453	-0.0942	PSMB9
valproic acid-6168	2/100	0.197972	0.467314	0.369318	0.724338	0.058515	-0.09477	TSPAN13;ID3

AG-013608-5909	1/100	0.561955	0.561955	0.724338	0.724338	0.164499	-0.09481	CTGF
AG-028671-6582	1/100	0.561955	0.561955	0.724338	0.724338	0.164939	-0.09506	CTGF
aconitine-7149	1/100	0.561955	0.561955	0.724338	0.724338	0.165338	-0.09529	IFI6
aconitine-1784	1/100	0.561955	0.561955	0.724338	0.724338	0.167976	-0.09681	RPS14P3
8-azaguanine-1791	1/100	0.561955	0.561955	0.724338	0.724338	0.168261	-0.09697	ID1
alpha-estradiol-2670	1/100	0.561955	0.561955	0.724338	0.724338	0.168697	-0.09723	CTSD
adrenosterone-3107	1/100	0.561955	0.561955	0.724338	0.724338	0.169498	-0.09769	ISG15
zuclopenthixol-4843	2/100	0.197972	0.467314	0.369318	0.724338	0.060483	-0.09796	RNF43;SECTM1
8-azaguanine-7444	1/100	0.561955	0.561955	0.724338	0.724338	0.17017	-0.09807	LGALS3BP
5707885-6385	1/100	0.561955	0.561955	0.724338	0.724338	0.172508	-0.09942	ITM2C
alclometasone-6229	1/100	0.561955	0.561955	0.724338	0.724338	0.172703	-0.09953	LGALS3BP
alverine-6345	1/100	0.561955	0.561955	0.724338	0.724338	0.172981	-0.09969	CDH3
allantoin-1800	1/100	0.561955	0.561955	0.724338	0.724338	0.173344	-0.0999	IFI6
amikacin-3233	1/100	0.561955	0.561955	0.724338	0.724338	0.173401	-0.09994	LYZ
acepromazine-1777	1/100	0.561955	0.561955	0.724338	0.724338	0.174174	-0.10038	TUBA1A
alprostadil-7358	1/100	0.561955	0.561955	0.724338	0.724338	0.177444	-0.10227	IFI6
amiloride-1970	1/100	0.561955	0.561955	0.724338	0.724338	0.179225	-0.10329	ITGAV
8-azaguanine-1670	1/100	0.561955	0.561955	0.724338	0.724338	0.180639	-0.10411	TMPRSS3
alprenolol-3188	1/100	0.561955	0.561955	0.724338	0.724338	0.181304	-0.10449	LYZ
acetylsalicylic acid-1042	1/100	0.561955	0.561955	0.724338	0.724338	0.181713	-0.10473	IFNGR2
alpha-estradiol-1210	1/100	0.561955	0.561955	0.724338	0.724338	0.181969	-0.10487	CCL20
altizide-4491	1/100	0.561955	0.561955	0.724338	0.724338	0.182505	-0.10518	RPS14P3
acepromazine-2769	1/100	0.561955	0.561955	0.724338	0.724338	0.18403	-0.10606	GPX2
acenocoumarol-1394	1/100	0.561955	0.561955	0.724338	0.724338	0.184918	-0.10657	PIGR
zoxazolamine-2625	2/100	0.197972	0.467314	0.369318	0.724338	0.065822	-0.10661	PDIA3;S100A4
acetohexamide-1829	1/100	0.561955	0.561955	0.724338	0.724338	0.185244	-0.10676	S100A4
adrenosterone-5464	1/100	0.561955	0.561955	0.724338	0.724338	0.185305	-0.1068	GPX2
withaferin A-3819	2/100	0.197972	0.467314	0.369318	0.724338	0.066818	-0.10822	TXNIP;CXCL1
AH-23848-6890	1/100	0.561955	0.561955	0.724338	0.724338	0.189136	-0.10901	SECTM1
acetylsalicylic acid-1204	1/100	0.561955	0.561955	0.724338	0.724338	0.189924	-0.10946	PGM1
alfaxalone-6514	1/100	0.561955	0.561955	0.724338	0.724338	0.190331	-0.10969	SECTM1
alfuzosin-1586	1/100	0.561955	0.561955	0.724338	0.724338	0.192552	-0.11097	IFITM3
alimemazine-3478	1/100	0.561955	0.561955	0.724338	0.724338	0.192837	-0.11114	LGALS3BP
AG-012559-6884	1/100	0.561955	0.561955	0.724338	0.724338	0.193842	-0.11172	IFI6
amprolium-1479	1/100	0.561955	0.561955	0.724338	0.724338	0.193912	-0.11176	ID3
zaprinast-5349	2/100	0.197972	0.467314	0.369318	0.724338	0.070052	-0.11346	IFNGR2;IFI6
amikacin-5314	1/100	0.561955	0.561955	0.724338	0.724338	0.197821	-0.11401	IFI6

alfuzosin-5242	1/100	0.561955	0.561955	0.724338	0.724338	0.198583	-0.11445	IFI6
verapamil-1927	2/100	0.197972	0.467314	0.369318	0.724338	0.070796	-0.11466	MMP1;TSPAN1
alcuronium chloride-4409	1/100	0.561955	0.561955	0.724338	0.724338	0.200584	-0.1156	AP1S1
amodiaquine-3186	1/100	0.561955	0.561955	0.724338	0.724338	0.201307	-0.11602	TMPRSS3
adenosine phosphate-6760	1/100	0.561955	0.561955	0.724338	0.724338	0.20151	-0.11614	PDIA3
amiodarone-4657	1/100	0.561955	0.561955	0.724338	0.724338	0.201544	-0.11616	TGFBI
troglitazone-431	2/100	0.197972	0.467314	0.369318	0.724338	0.072721	-0.11778	ID3;CTGF
alsterpaullone-7056	1/100	0.561955	0.561955	0.724338	0.724338	0.204606	-0.11792	CTGF
apomorphine-6683	1/100	0.561955	0.561955	0.724338	0.724338	0.205081	-0.11819	IFI6
AG-028671-6557	1/100	0.561955	0.561955	0.724338	0.724338	0.206002	-0.11873	CTGF
amoxicillin-5385	1/100	0.561955	0.561955	0.724338	0.724338	0.206451	-0.11898	IFI27
amoxicillin-1265	1/100	0.561955	0.561955	0.724338	0.724338	0.206607	-0.11907	CTSD
ampicillin-2030	1/100	0.561955	0.561955	0.724338	0.724338	0.208027	-0.11989	GGH
aminoglutethimide-2390	1/100	0.561955	0.561955	0.724338	0.724338	0.208809	-0.12034	CKB
ambroxol-3238	1/100	0.561955	0.561955	0.724338	0.724338	0.209884	-0.12096	RCN1
anabasine-6774	1/100	0.561955	0.561955	0.724338	0.724338	0.211477	-0.12188	S100A4
amiodarone-2434	1/100	0.561955	0.561955	0.724338	0.724338	0.212428	-0.12243	IGFBP2
amoxapine-4996	1/100	0.561955	0.561955	0.724338	0.724338	0.212733	-0.12261	IFI27
amiloride-1470	1/100	0.561955	0.561955	0.724338	0.724338	0.214219	-0.12346	GPX2
alexidine-2576	1/100	0.561955	0.561955	0.724338	0.724338	0.214902	-0.12386	C3
vorinostat-6179	2/100	0.197972	0.467314	0.369318	0.724338	0.076527	-0.12395	IFNGR2;CD14
AG-013608-5944	1/100	0.561955	0.561955	0.724338	0.724338	0.216381	-0.12471	QPCT
alpha-estradiol-6970	1/100	0.561955	0.561955	0.724338	0.724338	0.219293	-0.12639	S100A4
amylocaine-1491	1/100	0.561955	0.561955	0.724338	0.724338	0.220141	-0.12687	ID3
wortmannin-6202	2/100	0.197972	0.467314	0.369318	0.724338	0.079064	-0.12806	CCL20;TCN1
withaferin A-3902	2/100	0.197972	0.467314	0.369318	0.724338	0.0791	-0.12811	TXNIP;ITM2C
apomorphine-1505	1/100	0.561955	0.561955	0.724338	0.724338	0.222327	-0.12813	TFF2
amiloride-4109	1/100	0.561955	0.561955	0.724338	0.724338	0.222484	-0.12822	PLP2
amiloride-1890	1/100	0.561955	0.561955	0.724338	0.724338	0.222514	-0.12824	IGFBP2
alfuzosin-5605	1/100	0.561955	0.561955	0.724338	0.724338	0.225217	-0.1298	S100A6
alprostadil-6555	1/100	0.561955	0.561955	0.724338	0.724338	0.225272	-0.12983	CTGF
atracurium besilate-1702	1/100	0.561955	0.561955	0.724338	0.724338	0.225405	-0.12991	LGALS3BP
aciclovir-1960	1/100	0.561955	0.561955	0.724338	0.724338	0.226037	-0.13027	GPX2
W-13-440	2/100	0.197972	0.467314	0.369318	0.724338	0.080551	-0.13046	RNF43;GPX2
ajmaline-1749	1/100	0.561955	0.561955	0.724338	0.724338	0.227378	-0.13105	CCL20
ambroxol-5319	1/100	0.561955	0.561955	0.724338	0.724338	0.228018	-0.13141	IFI6
ampyrone-4507	1/100	0.561955	0.561955	0.724338	0.724338	0.229907	-0.1325	IFI6

ascorbic acid-5407	1/100	0.561955	0.561955	0.724338	0.724338	0.230756	-0.13299	IFI27
vidarabine-2706	2/100	0.197972	0.467314	0.369318	0.724338	0.082242	-0.1332	TSPAN13;IGFBP2
AR-A014418-7070	1/100	0.561955	0.561955	0.724338	0.724338	0.232003	-0.13371	GPX2
AR-A014418-7092	1/100	0.561955	0.561955	0.724338	0.724338	0.234101	-0.13492	BMP4
aminocaproic acid-6501	1/100	0.561955	0.561955	0.724338	0.724338	0.234625	-0.13522	GPX2
alpha-estradiol-990	1/100	0.561955	0.561955	0.724338	0.724338	0.235041	-0.13546	PGM1
aminoglutethimide-7421	1/100	0.561955	0.561955	0.724338	0.724338	0.236493	-0.1363	CKB
amrinone-2724	1/100	0.561955	0.561955	0.724338	0.724338	0.236822	-0.13649	CKB
apramycin-7334	1/100	0.561955	0.561955	0.724338	0.724338	0.238084	-0.13722	IFI6
articaine-7272	1/100	0.561955	0.561955	0.724338	0.724338	0.23812	-0.13724	SECTM1
aminohippuric acid-6453	1/100	0.561955	0.561955	0.724338	0.724338	0.240423	-0.13856	TSPAN1
amoxicillin-2620	1/100	0.561955	0.561955	0.724338	0.724338	0.240662	-0.1387	PDIA3
alprostadil-4179	1/100	0.561955	0.561955	0.724338	0.724338	0.245171	-0.1413	C3
vinburnine-1788	2/100	0.197972	0.467314	0.369318	0.724338	0.087367	-0.1415	RPS14P3;CKB
AR-A014418-7097	1/100	0.561955	0.561955	0.724338	0.724338	0.246586	-0.14212	C3
benperidol-2836	1/100	0.561955	0.561955	0.724338	0.724338	0.247877	-0.14286	LGALS3BP
alverine-2110	1/100	0.561955	0.561955	0.724338	0.724338	0.248536	-0.14324	CCL20
antazoline-3173	1/100	0.561955	0.561955	0.724338	0.724338	0.248803	-0.14339	ETS2
arecoline-5423	1/100	0.561955	0.561955	0.724338	0.724338	0.249321	-0.14369	IFI6
amylocaine-4089	1/100	0.561955	0.561955	0.724338	0.724338	0.24957	-0.14384	TSPAN1
articaine-3138	1/100	0.561955	0.561955	0.724338	0.724338	0.2496	-0.14385	TSPAN13
antazoline-7128	1/100	0.561955	0.561955	0.724338	0.724338	0.249831	-0.14399	IGFBP2
arcaine-3010	1/100	0.561955	0.561955	0.724338	0.724338	0.250806	-0.14455	CTSD
arcaine-3349	1/100	0.561955	0.561955	0.724338	0.724338	0.250886	-0.14459	RNF43
zaprinast-3226	2/100	0.197972	0.467314	0.369318	0.724338	0.089858	-0.14554	TFF2;LYZ
amodiaquine-1570	1/100	0.561955	0.561955	0.724338	0.724338	0.252988	-0.14581	ITM2C
androsterone-2650	1/100	0.561955	0.561955	0.724338	0.724338	0.254999	-0.14696	TMPRSS3
benfluorex-2621	1/100	0.561955	0.561955	0.724338	0.724338	0.255702	-0.14737	GPX2
atracurium besilate-1824	1/100	0.561955	0.561955	0.724338	0.724338	0.257649	-0.14849	CTGF
amphotericin B-3303	1/100	0.561955	0.561955	0.724338	0.724338	0.258234	-0.14883	RPS14P3
artemisinin-7007	1/100	0.561955	0.561955	0.724338	0.724338	0.258379	-0.14891	S100A4
apigenin-1321	1/100	0.561955	0.561955	0.724338	0.724338	0.259055	-0.1493	RCN1
azacitidine-4128	1/100	0.561955	0.561955	0.724338	0.724338	0.25973	-0.14969	ID1
bacitracin-7448	1/100	0.561955	0.561955	0.724338	0.724338	0.260046	-0.14987	GPX2
aztreonam-2118	1/100	0.561955	0.561955	0.724338	0.724338	0.26083	-0.15033	CCL20
atropine oxide-2054	1/100	0.561955	0.561955	0.724338	0.724338	0.264979	-0.15272	IL32
atropine-2761	1/100	0.561955	0.561955	0.724338	0.724338	0.265847	-0.15322	RNF43

asiaticoside-2943	1/100	0.561955	0.561955	0.724338	0.724338	0.266513	-0.1536	IFI6
BAS-012416453-6908	1/100	0.561955	0.561955	0.724338	0.724338	0.266871	-0.15381	CTSD
bacitracin-6488	1/100	0.561955	0.561955	0.724338	0.724338	0.268315	-0.15464	TFF2
anisomycin-5364	1/100	0.561955	0.561955	0.724338	0.724338	0.269444	-0.15529	IFI6
wortmannin-389	2/100	0.197972	0.467314	0.369318	0.724338	0.095905	-0.15533	PIGR;DSG2
astemizole-1365	1/100	0.561955	0.561955	0.724338	0.724338	0.26956	-0.15536	PIGR
ampicillin-1530	1/100	0.561955	0.561955	0.724338	0.724338	0.270245	-0.15575	TFF2
apigenin-4578	1/100	0.561955	0.561955	0.724338	0.724338	0.270414	-0.15585	CXCL1
baclofen-1536	1/100	0.561955	0.561955	0.724338	0.724338	0.271061	-0.15622	C3
astemizole-2211	1/100	0.561955	0.561955	0.724338	0.724338	0.273356	-0.15754	SECTM1
androsterone-5696	1/100	0.561955	0.561955	0.724338	0.724338	0.273973	-0.1579	S100A4
arachidonyltrifluoromethane-594	1/100	0.561955	0.561955	0.724338	0.724338	0.273994	-0.15791	IFITM3
bambuterol-3199	1/100	0.561955	0.561955	0.724338	0.724338	0.275816	-0.15896	LYZ
atropine-5865	1/100	0.561955	0.561955	0.724338	0.724338	0.276063	-0.1591	CDH3
bendroflumethiazide-3934	1/100	0.561955	0.561955	0.724338	0.724338	0.276858	-0.15956	BMP4
atropine-7219	1/100	0.561955	0.561955	0.724338	0.724338	0.277976	-0.16021	BMP4
arachidonic acid-443	1/100	0.561955	0.561955	0.724338	0.724338	0.280745	-0.1618	SLC12A2
anisomycin-2658	1/100	0.561955	0.561955	0.724338	0.724338	0.28111	-0.16201	BMP4
BCB000040-7493	1/100	0.561955	0.561955	0.724338	0.724338	0.282302	-0.1627	BMP4
arcaine-4974	1/100	0.561955	0.561955	0.724338	0.724338	0.28344	-0.16336	IFI27
azapropazone-6522	1/100	0.561955	0.561955	0.724338	0.724338	0.286773	-0.16528	SECTM1
benfotiamine-2177	1/100	0.561955	0.561955	0.724338	0.724338	0.288146	-0.16607	TSPAN13
azacyclonol-1520	1/100	0.561955	0.561955	0.724338	0.724338	0.292635	-0.16866	BMP4
azathioprine-4667	1/100	0.561955	0.561955	0.724338	0.724338	0.293364	-0.16908	OLFM4
azacitidine-4010	1/100	0.561955	0.561955	0.724338	0.724338	0.294006	-0.16945	ID1
atropine-1768	1/100	0.561955	0.561955	0.724338	0.724338	0.294853	-0.16993	CTSH
azacitidine-3348	1/100	0.561955	0.561955	0.724338	0.724338	0.296637	-0.17096	ID1
azaperone-3573	1/100	0.561955	0.561955	0.724338	0.724338	0.296761	-0.17103	S100A4
apomorphine-1923	1/100	0.561955	0.561955	0.724338	0.724338	0.298012	-0.17175	SECTM1
azaperone-7231	1/100	0.561955	0.561955	0.724338	0.724338	0.298131	-0.17182	BMP4
beta-escin-4364	1/100	0.561955	0.561955	0.724338	0.724338	0.303453	-0.17489	QPCT
azathioprine-1945	1/100	0.561955	0.561955	0.724338	0.724338	0.303482	-0.17491	GPX2
azlocillin-6262	1/100	0.561955	0.561955	0.724338	0.724338	0.303839	-0.17511	TGFBI
atropine methonitrate-3116	1/100	0.561955	0.561955	0.724338	0.724338	0.303964	-0.17518	ISG15
benzbromarone-5015	1/100	0.561955	0.561955	0.724338	0.724338	0.304045	-0.17523	S100A4
benzathine benzylpenicillin-4022	1/100	0.561955	0.561955	0.724338	0.724338	0.304494	-0.17549	MMP3
benzylpenicillin-6155	1/100	0.561955	0.561955	0.724338	0.724338	0.304749	-0.17564	HIF1A

azacyclonol-5398	1/100	0.561955	0.561955	0.724338	0.724338	0.305271	-0.17594	IFI27
aztreonam-2282	1/100	0.561955	0.561955	0.724338	0.724338	0.307325	-0.17712	TFF2
benzthiazide-3329	1/100	0.561955	0.561955	0.724338	0.724338	0.308612	-0.17786	TSPAN1
atropine oxide-4476	1/100	0.561955	0.561955	0.724338	0.724338	0.309484	-0.17837	IFI6
BCB000040-7488	1/100	0.561955	0.561955	0.724338	0.724338	0.30972	-0.1785	RCN1
benzocaine-2167	1/100	0.561955	0.561955	0.724338	0.724338	0.310115	-0.17873	TCN1
benserazide-5322	1/100	0.561955	0.561955	0.724338	0.724338	0.311246	-0.17938	IFI6
bepridil-5674	1/100	0.561955	0.561955	0.724338	0.724338	0.31165	-0.17961	AP1S1
benzamil-4294	1/100	0.561955	0.561955	0.724338	0.724338	0.313847	-0.18088	MMP3
bemegride-3389	1/100	0.561955	0.561955	0.724338	0.724338	0.31457	-0.1813	C3
benzonatate-1801	1/100	0.561955	0.561955	0.724338	0.724338	0.314868	-0.18147	TCN1
benfotiamine-6032	1/100	0.561955	0.561955	0.724338	0.724338	0.315958	-0.1821	IFITM1
bethanechol-5114	1/100	0.561955	0.561955	0.724338	0.724338	0.317643	-0.18307	TSPAN1
bacampicillin-4592	1/100	0.561955	0.561955	0.724338	0.724338	0.31875	-0.18371	GPX2
benzocaine-4808	1/100	0.561955	0.561955	0.724338	0.724338	0.321253	-0.18515	IFI6
bephenium hydroxynaphthoate-3089	1/100	0.561955	0.561955	0.724338	0.724338	0.321355	-0.18521	LY6E
beta-escin-2194	1/100	0.561955	0.561955	0.724338	0.724338	0.321578	-0.18534	IFI6
benzamil-2200	1/100	0.561955	0.561955	0.724338	0.724338	0.32278	-0.18603	IFI6
BCB000038-7520	1/100	0.561955	0.561955	0.724338	0.724338	0.323436	-0.18641	QPCT
benzathine benzylpenicillin-7359	1/100	0.561955	0.561955	0.724338	0.724338	0.324951	-0.18728	IFI6
benfotiamine-3837	1/100	0.561955	0.561955	0.724338	0.724338	0.326259	-0.18803	TFF2
bephenium hydroxynaphthoate-5628	1/100	0.561955	0.561955	0.724338	0.724338	0.329095	-0.18967	TMPRSS3
azathioprine-338	1/100	0.561955	0.561955	0.724338	0.724338	0.330878	-0.1907	RCN1
betazole-5445	1/100	0.561955	0.561955	0.724338	0.724338	0.331054	-0.1908	IFI6
bemegride-6668	1/100	0.561955	0.561955	0.724338	0.724338	0.331325	-0.19095	PSMB9
benzethonium chloride-6070	1/100	0.561955	0.561955	0.724338	0.724338	0.332155	-0.19143	BMP4
beclometasone-3001	1/100	0.561955	0.561955	0.724338	0.724338	0.333525	-0.19222	SLC12A2
benzamil-6056	1/100	0.561955	0.561955	0.724338	0.724338	0.338155	-0.19489	MMP3
betonicine-4767	1/100	0.561955	0.561955	0.724338	0.724338	0.342416	-0.19735	TFF2
bergenin-5870	1/100	0.561955	0.561955	0.724338	0.724338	0.343655	-0.19806	CTSD
bretylium tosilate-5020	1/100	0.561955	0.561955	0.724338	0.724338	0.344775	-0.19871	SLPI
verteporfin-6817	2/100	0.197972	0.467314	0.369318	0.724338	0.122727	-0.19877	ITGAV;DSG2
bucladesine-3483	1/100	0.561955	0.561955	0.724338	0.724338	0.345024	-0.19885	TMPRSS3
BCB000039-7531	1/100	0.561955	0.561955	0.724338	0.724338	0.345377	-0.19905	PDZK1IP1
benzamil-4760	1/100	0.561955	0.561955	0.724338	0.724338	0.346286	-0.19958	SPINK1
berberine-1778	1/100	0.561955	0.561955	0.724338	0.724338	0.346399	-0.19964	C3
benzathine benzylpenicillin-4140	1/100	0.561955	0.561955	0.724338	0.724338	0.347965	-0.20054	IFI6

bezafibrate-1275	1/100	0.561955	0.561955	0.724338	0.724338	0.34962	-0.2015	IFITM3
brinzolamide-6670	1/100	0.561955	0.561955	0.724338	0.724338	0.349808	-0.20161	C3
bicuculline-2139	1/100	0.561955	0.561955	0.724338	0.724338	0.351903	-0.20281	CTSD
betamethasone-1590	1/100	0.561955	0.561955	0.724338	0.724338	0.352833	-0.20335	IFITM3
bethanechol-3537	1/100	0.561955	0.561955	0.724338	0.724338	0.352913	-0.2034	S100A4
butyl hydroxybenzoate-5608	1/100	0.561955	0.561955	0.724338	0.724338	0.354877	-0.20453	RNF43
benzbromarone-6669	1/100	0.561955	0.561955	0.724338	0.724338	0.35645	-0.20543	TGFBI
brompheniramine-3271	1/100	0.561955	0.561955	0.724338	0.724338	0.356851	-0.20567	TSPAN1
benzonatate-1679	1/100	0.561955	0.561955	0.724338	0.724338	0.358152	-0.20641	LGALS3BP
benzamil-3738	1/100	0.561955	0.561955	0.724338	0.724338	0.359012	-0.20691	CXCL1
capsaicin-4612	1/100	0.561955	0.561955	0.724338	0.724338	0.359328	-0.20709	TGFBI
betazole-6344	1/100	0.561955	0.561955	0.724338	0.724338	0.359484	-0.20718	CTSD
bethanechol-5539	1/100	0.561955	0.561955	0.724338	0.724338	0.361255	-0.2082	LGALS3BP
bezafibrate-6653	1/100	0.561955	0.561955	0.724338	0.724338	0.362448	-0.20889	PSMB9
buflomedil-4840	1/100	0.561955	0.561955	0.724338	0.724338	0.362563	-0.20896	LGALS3BP
biperiden-3321	1/100	0.561955	0.561955	0.724338	0.724338	0.363887	-0.20972	SLPI
berberine-6791	1/100	0.561955	0.561955	0.724338	0.724338	0.366069	-0.21098	TXNIP
bupropion-6256	1/100	0.561955	0.561955	0.724338	0.724338	0.366344	-0.21114	SECTM1
bendroflumethiazide-3415	1/100	0.561955	0.561955	0.724338	0.724338	0.368111	-0.21215	ENC1
beta-escin-4544	1/100	0.561955	0.561955	0.724338	0.724338	0.369715	-0.21308	GPX2
budesonide-5431	1/100	0.561955	0.561955	0.724338	0.724338	0.370601	-0.21359	IFI6
blebbistatin-837	1/100	0.561955	0.561955	0.724338	0.724338	0.371409	-0.21406	PGM1
bromperidol-7457	1/100	0.561955	0.561955	0.724338	0.724338	0.372953	-0.21495	TSPAN1
bezafibrate-4999	1/100	0.561955	0.561955	0.724338	0.724338	0.375163	-0.21622	IFI27
betulinic acid-4181	1/100	0.561955	0.561955	0.724338	0.724338	0.375764	-0.21657	AP1S1
betulinic acid-1345	1/100	0.561955	0.561955	0.724338	0.724338	0.377033	-0.2173	TXNIP
beta-escin-6050	1/100	0.561955	0.561955	0.724338	0.724338	0.377809	-0.21774	TFF2
butirosin-6080	1/100	0.561955	0.561955	0.724338	0.724338	0.379155	-0.21852	MMP3
biotin-6689	1/100	0.561955	0.561955	0.724338	0.724338	0.379183	-0.21854	TFF2
bromopride-4741	1/100	0.561955	0.561955	0.724338	0.724338	0.379898	-0.21895	IFI6
benzydamine-1552	1/100	0.561955	0.561955	0.724338	0.724338	0.380183	-0.21911	IFITM3
calycanthine-5744	1/100	0.561955	0.561955	0.724338	0.724338	0.380459	-0.21927	GPX2
buflomedil-3274	1/100	0.561955	0.561955	0.724338	0.724338	0.38383	-0.22121	SECTM1
bezafibrate-2630	1/100	0.561955	0.561955	0.724338	0.724338	0.385182	-0.22199	BMP4
bisoprolol-5348	1/100	0.561955	0.561955	0.724338	0.724338	0.385331	-0.22208	RPS14P3
blebbistatin-954	1/100	0.561955	0.561955	0.724338	0.724338	0.387678	-0.22343	TMPRSS3
bromopride-2182	1/100	0.561955	0.561955	0.724338	0.724338	0.388807	-0.22408	IFI6

calcium folinate-4725	1/100	0.561955	0.561955	0.724338	0.724338	0.389094	-0.22425	SPINK1
bromocriptine-5665	1/100	0.561955	0.561955	0.724338	0.724338	0.390298	-0.22494	IFI27
caffeic acid-6753	1/100	0.561955	0.561955	0.724338	0.724338	0.390388	-0.22499	IFI6
biperiden-4684	1/100	0.561955	0.561955	0.724338	0.724338	0.391395	-0.22557	DUOX2
buspirone-2637	1/100	0.561955	0.561955	0.724338	0.724338	0.391464	-0.22561	LGALS3BP
calycanthine-2764	1/100	0.561955	0.561955	0.724338	0.724338	0.39212	-0.22599	GPX2
cantharidin-3075	1/100	0.561955	0.561955	0.724338	0.724338	0.392303	-0.2261	CD14
brinzolamide-3230	1/100	0.561955	0.561955	0.724338	0.724338	0.392378	-0.22614	LYZ
butamben-5792	1/100	0.561955	0.561955	0.724338	0.724338	0.394641	-0.22744	CTGF
bisacodyl-2435	1/100	0.561955	0.561955	0.724338	0.724338	0.394822	-0.22755	IFI6
bromocriptine-1507	1/100	0.561955	0.561955	0.724338	0.724338	0.39526	-0.2278	TFF2
bumetanide-7440	1/100	0.561955	0.561955	0.724338	0.724338	0.39599	-0.22822	BMP4
betulin-2952	1/100	0.561955	0.561955	0.724338	0.724338	0.396008	-0.22823	ID3
bupivacaine-5537	1/100	0.561955	0.561955	0.724338	0.724338	0.398987	-0.22995	RNF43
calcium pantothenate-4189	1/100	0.561955	0.561955	0.724338	0.724338	0.399217	-0.23008	IFI6
bumetanide-2409	1/100	0.561955	0.561955	0.724338	0.724338	0.399382	-0.23018	CKB
C-75-6399	1/100	0.561955	0.561955	0.724338	0.724338	0.399415	-0.2302	TFF2
bupropion-1564	1/100	0.561955	0.561955	0.724338	0.724338	0.400658	-0.23091	MEST
canavanine-4782	1/100	0.561955	0.561955	0.724338	0.724338	0.401889	-0.23162	DSG2
bromperidol-1723	1/100	0.561955	0.561955	0.724338	0.724338	0.402877	-0.23219	ISG15
butirosin-6779	1/100	0.561955	0.561955	0.724338	0.724338	0.404091	-0.23289	IFI6
carcinine-3242	1/100	0.561955	0.561955	0.724338	0.724338	0.405083	-0.23346	SECTM1
carbinoxamine-7138	1/100	0.561955	0.561955	0.724338	0.724338	0.405492	-0.2337	MMP1
butacaine-5748	1/100	0.561955	0.561955	0.724338	0.724338	0.406889	-0.2345	IFI6
calcium folinate-3703	1/100	0.561955	0.561955	0.724338	0.724338	0.408386	-0.23537	MMP3
cefadroxil-4080	1/100	0.561955	0.561955	0.724338	0.724338	0.409994	-0.23629	IFI6
cefoperazone-6323	1/100	0.561955	0.561955	0.724338	0.724338	0.41008	-0.23634	SECTM1
carbimazole-2437	1/100	0.561955	0.561955	0.724338	0.724338	0.410834	-0.23678	IFI6
carcinine-4225	1/100	0.561955	0.561955	0.724338	0.724338	0.411029	-0.23689	LY6E
buflomedil-4258	1/100	0.561955	0.561955	0.724338	0.724338	0.41231	-0.23763	IFI6
butacaine-2728	1/100	0.561955	0.561955	0.724338	0.724338	0.412534	-0.23776	IGFBP2
carmustine-6883	1/100	0.561955	0.561955	0.724338	0.724338	0.414245	-0.23874	RNF43
canrenoic acid-2228	1/100	0.561955	0.561955	0.724338	0.724338	0.414763	-0.23904	TFF2
cefixime-4567	1/100	0.561955	0.561955	0.724338	0.724338	0.416967	-0.24031	LGALS3BP
cefepime-5761	1/100	0.561955	0.561955	0.724338	0.724338	0.417671	-0.24072	IFI6
calycanthine-6221	1/100	0.561955	0.561955	0.724338	0.724338	0.419374	-0.2417	IGFBP2
bisoprolol-2642	1/100	0.561955	0.561955	0.724338	0.724338	0.420434	-0.24231	BMP4

C-75-6423	1/100	0.561955	0.561955	0.724338	0.724338	0.421606	-0.24299	S100A4
carmustine-6888	1/100	0.561955	0.561955	0.724338	0.724338	0.422593	-0.24355	SECTM1
bucladesine-591	1/100	0.561955	0.561955	0.724338	0.724338	0.423874	-0.24429	GPX2
captopril-1907	1/100	0.561955	0.561955	0.724338	0.724338	0.42521	-0.24506	IFI6
bufexamac-7413	1/100	0.561955	0.561955	0.724338	0.724338	0.4277	-0.2465	TGFBI
cefapirin-2730	1/100	0.561955	0.561955	0.724338	0.724338	0.428504	-0.24696	SLC12A2
CAY-10397-7071	1/100	0.561955	0.561955	0.724338	0.724338	0.42874	-0.2471	GPX2
captopril-1988	1/100	0.561955	0.561955	0.724338	0.724338	0.429348	-0.24745	PLP2
cefamandole-4718	1/100	0.561955	0.561955	0.724338	0.724338	0.430009	-0.24783	SPINK1
cefmetazole-7222	1/100	0.561955	0.561955	0.724338	0.724338	0.431251	-0.24854	S100A4
captopril-1488	1/100	0.561955	0.561955	0.724338	0.724338	0.431446	-0.24866	LGALS3BP
cefixime-1310	1/100	0.561955	0.561955	0.724338	0.724338	0.432079	-0.24902	ANXA3
carbenoxolone-3353	1/100	0.561955	0.561955	0.724338	0.724338	0.433183	-0.24966	BMP4
chenodeoxycholic acid-2402	1/100	0.561955	0.561955	0.724338	0.724338	0.434302	-0.2503	CKB
ceftazidime-5473	1/100	0.561955	0.561955	0.724338	0.724338	0.434346	-0.25033	IFI6
cefalonium-2921	1/100	0.561955	0.561955	0.724338	0.724338	0.435309	-0.25088	IFI6
cefamandole-3436	1/100	0.561955	0.561955	0.724338	0.724338	0.438627	-0.2528	GPX2
cefalexin-5250	1/100	0.561955	0.561955	0.724338	0.724338	0.439582	-0.25335	GPX2
cefazolin-3426	1/100	0.561955	0.561955	0.724338	0.724338	0.441479	-0.25444	LGALS3BP
cefotetan-1319	1/100	0.561955	0.561955	0.724338	0.724338	0.442837	-0.25522	IFITM3
chenodeoxycholic acid-7310	1/100	0.561955	0.561955	0.724338	0.724338	0.447507	-0.25791	IFI6
cephaeline-2429	1/100	0.561955	0.561955	0.724338	0.724338	0.45099	-0.25992	SPP1
cefoxitin-7148	1/100	0.561955	0.561955	0.724338	0.724338	0.451073	-0.25997	LY6E
chlorhexidine-6302	1/100	0.561955	0.561955	0.724338	0.724338	0.451608	-0.26028	CDH3
cefotaxime-1389	1/100	0.561955	0.561955	0.724338	0.724338	0.45198	-0.26049	IFITM3
cefalotin-4482	1/100	0.561955	0.561955	0.724338	0.724338	0.453884	-0.26159	RPS14P3
cefuroxime-6088	1/100	0.561955	0.561955	0.724338	0.724338	0.455747	-0.26266	BMP4
cefalonium-4245	1/100	0.561955	0.561955	0.724338	0.724338	0.455788	-0.26269	LY6E
cefoxitin-6796	1/100	0.561955	0.561955	0.724338	0.724338	0.455937	-0.26277	TFF2
chlorpromazine-1822	1/100	0.561955	0.561955	0.724338	0.724338	0.456714	-0.26322	IFI6
cefamandole-3696	1/100	0.561955	0.561955	0.724338	0.724338	0.457285	-0.26355	TSPAN8
carcinine-1305	1/100	0.561955	0.561955	0.724338	0.724338	0.459055	-0.26457	QPCT
cefazolin-3686	1/100	0.561955	0.561955	0.724338	0.724338	0.46044	-0.26537	IFI6
chlorogenic acid-3282	1/100	0.561955	0.561955	0.724338	0.724338	0.461781	-0.26614	TMPRSS3
butirosin-666	1/100	0.561955	0.561955	0.724338	0.724338	0.463088	-0.26689	CTSE
cefapirin-3471	1/100	0.561955	0.561955	0.724338	0.724338	0.466222	-0.2687	LGALS3BP
ciclosporin-4586	1/100	0.561955	0.561955	0.724338	0.724338	0.466486	-0.26885	GPX2

chloroquine-2869	1/100	0.561955	0.561955	0.724338	0.724338	0.466654	-0.26895	TFF2
chenodeoxycholic acid-7433	1/100	0.561955	0.561955	0.724338	0.724338	0.469466	-0.27057	TSPAN1
cefamandole-7394	1/100	0.561955	0.561955	0.724338	0.724338	0.469562	-0.27062	IGFBP2
carbarsone-3250	1/100	0.561955	0.561955	0.724338	0.724338	0.470725	-0.27129	SECTM1
ceftazidime-1721	1/100	0.561955	0.561955	0.724338	0.724338	0.471726	-0.27187	NOP10
chloramphenicol-1795	1/100	0.561955	0.561955	0.724338	0.724338	0.474363	-0.27339	IFI6
Chicago Sky Blue 6B-3266	1/100	0.561955	0.561955	0.724338	0.724338	0.479197	-0.27618	SECTM1
carbinoxamine-6786	1/100	0.561955	0.561955	0.724338	0.724338	0.481303	-0.27739	BMP4
carbimazole-5399	1/100	0.561955	0.561955	0.724338	0.724338	0.483127	-0.27844	QPCT
cefsulodin-4067	1/100	0.561955	0.561955	0.724338	0.724338	0.483575	-0.2787	AP1S1
cefuroxime-6261	1/100	0.561955	0.561955	0.724338	0.724338	0.486429	-0.28035	SECTM1
celecoxib-377	1/100	0.561955	0.561955	0.724338	0.724338	0.48798	-0.28124	PIGR
clorsulon-2884	1/100	0.561955	0.561955	0.724338	0.724338	0.49192	-0.28351	BMP4
chenodeoxycholic acid-6012	1/100	0.561955	0.561955	0.724338	0.724338	0.492395	-0.28378	IFI6
chlorcyclizine-4367	1/100	0.561955	0.561955	0.724338	0.724338	0.493576	-0.28446	RNF43
chlorpromazine-5493	1/100	0.561955	0.561955	0.724338	0.724338	0.495519	-0.28558	IFI6
chlorpromazine-1217	1/100	0.561955	0.561955	0.724338	0.724338	0.496464	-0.28613	CCL20
cinchonidine-2772	1/100	0.561955	0.561955	0.724338	0.724338	0.496794	-0.28632	RNF43
chlormezanone-1620	1/100	0.561955	0.561955	0.724338	0.724338	0.49934	-0.28779	IFITM3
cisapride-3305	1/100	0.561955	0.561955	0.724338	0.724338	0.500481	-0.28844	C3
chlorzoxazone-2100	1/100	0.561955	0.561955	0.724338	0.724338	0.500798	-0.28863	CCL20
cimetidine-4144	1/100	0.561955	0.561955	0.724338	0.724338	0.502976	-0.28988	AP1S1
chlortalidone-1581	1/100	0.561955	0.561955	0.724338	0.724338	0.503945	-0.29044	SLPI
chlorpropamide-144	1/100	0.561955	0.561955	0.724338	0.724338	0.50402	-0.29048	PIGR
chlorzoxazone-1416	1/100	0.561955	0.561955	0.724338	0.724338	0.504485	-0.29075	IFITM3
chlorpromazine-1864	1/100	0.561955	0.561955	0.724338	0.724338	0.504879	-0.29098	AP1S1
chrysin-3106	1/100	0.561955	0.561955	0.724338	0.724338	0.505262	-0.2912	IFI6
chlorprothixene-5291	1/100	0.561955	0.561955	0.724338	0.724338	0.506091	-0.29168	AP1S1
chlorphenamine-2055	1/100	0.561955	0.561955	0.724338	0.724338	0.506844	-0.29211	LGALS3BP
cinchocaine-1969	1/100	0.561955	0.561955	0.724338	0.724338	0.508076	-0.29282	CTSH
chlortalidone-3198	1/100	0.561955	0.561955	0.724338	0.724338	0.508253	-0.29292	LYZ
clebopride-5412	1/100	0.561955	0.561955	0.724338	0.724338	0.508396	-0.29301	IFI27
chlorpromazine-1642	1/100	0.561955	0.561955	0.724338	0.724338	0.508811	-0.29325	TMPRSS3
chlortalidone-7152	1/100	0.561955	0.561955	0.724338	0.724338	0.509634	-0.29372	LY6E
chlorpromazine-4441	1/100	0.561955	0.561955	0.724338	0.724338	0.510141	-0.29401	PSMB9
ciclopirox-2456	1/100	0.561955	0.561955	0.724338	0.724338	0.511097	-0.29456	IFI6
cephaeline-3290	1/100	0.561955	0.561955	0.724338	0.724338	0.512204	-0.2952	PRSS23

chrysin-5505	1/100	0.561955	0.561955	0.724338	0.724338	0.512691	-0.29548	ID1
clomifene-2624	1/100	0.561955	0.561955	0.724338	0.724338	0.513432	-0.29591	TMPRSS3
chlorpropamide-5391	1/100	0.561955	0.561955	0.724338	0.724338	0.513737	-0.29608	QPCT
chlorphenamine-2217	1/100	0.561955	0.561955	0.724338	0.724338	0.514958	-0.29679	SECTM1
citiolone-6031	1/100	0.561955	0.561955	0.724338	0.724338	0.515615	-0.29717	MMP3
ciclacillin-4536	1/100	0.561955	0.561955	0.724338	0.724338	0.518876	-0.29905	PSMB9
clofilium tosylate-3187	1/100	0.561955	0.561955	0.724338	0.724338	0.520302	-0.29987	LYZ
clobetasol-6095	1/100	0.561955	0.561955	0.724338	0.724338	0.521281	-0.30043	SLPI
chlorphenamine-1371	1/100	0.561955	0.561955	0.724338	0.724338	0.523123	-0.30149	PIGR
clemizole-4695	1/100	0.561955	0.561955	0.724338	0.724338	0.524488	-0.30228	S100A4
chlortetracycline-2042	1/100	0.561955	0.561955	0.724338	0.724338	0.525	-0.30258	IFITM3
chlorpromazine-1055	1/100	0.561955	0.561955	0.724338	0.724338	0.527672	-0.30412	S100A6
clomifene-6648	1/100	0.561955	0.561955	0.724338	0.724338	0.527941	-0.30427	PSMB9
clomifene-4994	1/100	0.561955	0.561955	0.724338	0.724338	0.528174	-0.3044	IFI27
chlorprothixene-1272	1/100	0.561955	0.561955	0.724338	0.724338	0.529972	-0.30544	IFITM3
cinoxacin-3463	1/100	0.561955	0.561955	0.724338	0.724338	0.530368	-0.30567	PSME2
cloperastine-4732	1/100	0.561955	0.561955	0.724338	0.724338	0.531276	-0.30619	PDIA3
chlortetracycline-1541	1/100	0.561955	0.561955	0.724338	0.724338	0.531814	-0.3065	TFF2
clioquinol-6461	1/100	0.561955	0.561955	0.724338	0.724338	0.532518	-0.30691	SECTM1
cinchonidine-1780	1/100	0.561955	0.561955	0.724338	0.724338	0.533643	-0.30756	HSPB1
ciprofloxacin-1522	1/100	0.561955	0.561955	0.724338	0.724338	0.534341	-0.30796	TFF2
clebopride-6311	1/100	0.561955	0.561955	0.724338	0.724338	0.541643	-0.31217	LY6E
cicloheximide-3464	1/100	0.561955	0.561955	0.724338	0.724338	0.54232	-0.31256	BMP4
cloperastine-3408	1/100	0.561955	0.561955	0.724338	0.724338	0.543288	-0.31311	LGALS3BP
ciprofibrate-6138	1/100	0.561955	0.561955	0.724338	0.724338	0.546033	-0.3147	TSPAN13
citalopram-3820	1/100	0.561955	0.561955	0.724338	0.724338	0.546644	-0.31505	BMP4
ciprofloxacin-6700	1/100	0.561955	0.561955	0.724338	0.724338	0.548086	-0.31588	IFI6
cisapride-6706	1/100	0.561955	0.561955	0.724338	0.724338	0.54816	-0.31592	CDH3
clozapine-2644	1/100	0.561955	0.561955	0.724338	0.724338	0.548421	-0.31607	LGALS3BP
clemizole-3672	1/100	0.561955	0.561955	0.724338	0.724338	0.550954	-0.31753	MMP3
colforsin-7059	1/100	0.561955	0.561955	0.724338	0.724338	0.551155	-0.31765	CTGF
clofilium tosylate-4492	1/100	0.561955	0.561955	0.724338	0.724338	0.551816	-0.31803	PSMB9
conessine-2135	1/100	0.561955	0.561955	0.724338	0.724338	0.552984	-0.3187	SLPI
cimetidine-1964	1/100	0.561955	0.561955	0.724338	0.724338	0.55369	-0.31911	SLPI
cinchocaine-1889	1/100	0.561955	0.561955	0.724338	0.724338	0.554618	-0.31964	CKB
clofazimine-5642	1/100	0.561955	0.561955	0.724338	0.724338	0.554676	-0.31968	TXNIP
clopamide-1605	1/100	0.561955	0.561955	0.724338	0.724338	0.555086	-0.31991	IFITM3

clonidine-6814	1/100	0.561955	0.561955	0.724338	0.724338	0.555446	-0.32012	GPX2
cinchonine-4107	1/100	0.561955	0.561955	0.724338	0.724338	0.556359	-0.32065	IFI6
clioquinol-5623	1/100	0.561955	0.561955	0.724338	0.724338	0.556773	-0.32089	BMP4
cinchocaine-1469	1/100	0.561955	0.561955	0.724338	0.724338	0.557832	-0.3215	LGALS3BP
corynanthine-4227	1/100	0.561955	0.561955	0.724338	0.724338	0.558609	-0.32194	TGFBI
ciclacillin-3800	1/100	0.561955	0.561955	0.724338	0.724338	0.559244	-0.32231	C3
cloperastine-2549	1/100	0.561955	0.561955	0.724338	0.724338	0.559458	-0.32243	ANXA3
clindamycin-1373	1/100	0.561955	0.561955	0.724338	0.724338	0.560524	-0.32305	IFITM3
cytisine-5739	1/100	0.561955	0.561955	0.724338	0.724338	0.563408	-0.32471	IFI6
cobalt chloride-454	1/100	0.561955	0.561955	0.724338	0.724338	0.565427	-0.32587	IFITM3
co-dergocrine mesilate-2793	1/100	0.561955	0.561955	0.724338	0.724338	0.566181	-0.32631	BMP4
convolamine-1779	1/100	0.561955	0.561955	0.724338	0.724338	0.5663	-0.32638	ITGAV
clozapine-6188	1/100	0.561955	0.561955	0.724338	0.724338	0.567304	-0.32696	ANXA3
clozapine-4670	1/100	0.561955	0.561955	0.724338	0.724338	0.571384	-0.32931	OLFM4
cyproterone-6124	1/100	0.561955	0.561955	0.724338	0.724338	0.571556	-0.32941	CCL20
cimetidine-1884	1/100	0.561955	0.561955	0.724338	0.724338	0.571925	-0.32962	LY6E
clomipramine-3182	1/100	0.561955	0.561955	0.724338	0.724338	0.571971	-0.32965	LYZ
ciprofibrate-3561	1/100	0.561955	0.561955	0.724338	0.724338	0.573987	-0.33081	CDH3
clonidine-4478	1/100	0.561955	0.561955	0.724338	0.724338	0.574469	-0.33109	C3
corticosterone-4064	1/100	0.561955	0.561955	0.724338	0.724338	0.574765	-0.33126	PSMB9
cyclopentolate-6132	1/100	0.561955	0.561955	0.724338	0.724338	0.578216	-0.33325	TSPAN13
corticosterone-1307	1/100	0.561955	0.561955	0.724338	0.724338	0.579397	-0.33393	SLPI
chlortetracycline-1958	1/100	0.561955	0.561955	0.724338	0.724338	0.57957	-0.33403	DEK
convolamine-2771	1/100	0.561955	0.561955	0.724338	0.724338	0.58038	-0.33449	SECTM1
cloxacillin-1443	1/100	0.561955	0.561955	0.724338	0.724338	0.580566	-0.3346	FOS
ciprofloxacin-1939	1/100	0.561955	0.561955	0.724338	0.724338	0.581544	-0.33516	GPX2
clobetasol-4497	1/100	0.561955	0.561955	0.724338	0.724338	0.583152	-0.33609	CXCL1
convolamine-7230	1/100	0.561955	0.561955	0.724338	0.724338	0.584077	-0.33662	GPX2
cyclobenzaprine-1332	1/100	0.561955	0.561955	0.724338	0.724338	0.584429	-0.33683	LY6E
CP-319743-7486	1/100	0.561955	0.561955	0.724338	0.724338	0.584739	-0.337	TMPRSS3
cobalt chloride-379	1/100	0.561955	0.561955	0.724338	0.724338	0.585997	-0.33773	BACE2
cyanocobalamin-4572	1/100	0.561955	0.561955	0.724338	0.724338	0.587697	-0.33871	C3
CP-863187-7553	1/100	0.561955	0.561955	0.724338	0.724338	0.590099	-0.34009	AP1S1
clozapine-5630	1/100	0.561955	0.561955	0.724338	0.724338	0.590727	-0.34046	S100A6
cycloserine-6139	1/100	0.561955	0.561955	0.724338	0.724338	0.591277	-0.34077	GPX2
clozapine-2689	1/100	0.561955	0.561955	0.724338	0.724338	0.594938	-0.34288	LY6E
crotamiton-3050	1/100	0.561955	0.561955	0.724338	0.724338	0.59564	-0.34329	IGFBP2

co-dergocrine mesilate-2136	1/100	0.561955	0.561955	0.724338	0.724338	0.59743	-0.34432	PLCB4
cortisone-2385	1/100	0.561955	0.561955	0.724338	0.724338	0.597683	-0.34446	CKB
clozapine-4453	1/100	0.561955	0.561955	0.724338	0.724338	0.598427	-0.34489	BMP4
cyproheptadine-1521	1/100	0.561955	0.561955	0.724338	0.724338	0.598662	-0.34503	TFF2
corticosterone-3244	1/100	0.561955	0.561955	0.724338	0.724338	0.599031	-0.34524	TMPRSS3
colecalciferol-6656	1/100	0.561955	0.561955	0.724338	0.724338	0.599334	-0.34542	PSMB9
clotrimazole-6207	1/100	0.561955	0.561955	0.724338	0.724338	0.599709	-0.34563	TSPAN1
cycloserine-7134	1/100	0.561955	0.561955	0.724338	0.724338	0.60029	-0.34597	TCN1
colchicine-1598	1/100	0.561955	0.561955	0.724338	0.724338	0.601526	-0.34668	SPP1
cromoglicic acid-5754	1/100	0.561955	0.561955	0.724338	0.724338	0.602134	-0.34703	IFI6
CP-863187-7508	1/100	0.561955	0.561955	0.724338	0.724338	0.602707	-0.34736	RNF43
clotrimazole-1549	1/100	0.561955	0.561955	0.724338	0.724338	0.60519	-0.34879	TUBA1A
clomipramine-1566	1/100	0.561955	0.561955	0.724338	0.724338	0.606431	-0.34951	ITGAV
cyclizine-2880	1/100	0.561955	0.561955	0.724338	0.724338	0.608679	-0.3508	C3
corbadrine-3450	1/100	0.561955	0.561955	0.724338	0.724338	0.609538	-0.3513	LGALS3BP
cotinine-5246	1/100	0.561955	0.561955	0.724338	0.724338	0.610219	-0.35169	IFI27
CP-319743-7532	1/100	0.561955	0.561955	0.724338	0.724338	0.612277	-0.35288	GPX2
desipramine-6693	1/100	0.561955	0.561955	0.724338	0.724338	0.6127	-0.35312	ID3
dantrolene-3867	1/100	0.561955	0.561955	0.724338	0.724338	0.614058	-0.3539	RPS14P3
CP-645525-01-7515	1/100	0.561955	0.561955	0.724338	0.724338	0.617767	-0.35604	RCN1
colchicine-644	1/100	0.561955	0.561955	0.724338	0.724338	0.618947	-0.35672	GPX2
clotrimazole-905	1/100	0.561955	0.561955	0.724338	0.724338	0.619037	-0.35677	BMP4
clozapine-5589	1/100	0.561955	0.561955	0.724338	0.724338	0.619246	-0.35689	QPCT
CP-690334-01-4380	1/100	0.561955	0.561955	0.724338	0.724338	0.619281	-0.35691	IFI27
CP-690334-01-4561	1/100	0.561955	0.561955	0.724338	0.724338	0.619615	-0.35711	C3
copper sulfate-459	1/100	0.561955	0.561955	0.724338	0.724338	0.620259	-0.35748	CTSE
dapsone-1705	1/100	0.561955	0.561955	0.724338	0.724338	0.620532	-0.35763	TMPRSS3
corynanthine-4811	1/100	0.561955	0.561955	0.724338	0.724338	0.624344	-0.35983	S100A4
CP-320650-01-4557	1/100	0.561955	0.561955	0.724338	0.724338	0.626379	-0.361	CTGF
dehydrocholic acid-5681	1/100	0.561955	0.561955	0.724338	0.724338	0.627018	-0.36137	GPX2
dexibuprofen-6471	1/100	0.561955	0.561955	0.724338	0.724338	0.628272	-0.36209	PDIA3
dacarbazine-1762	1/100	0.561955	0.561955	0.724338	0.724338	0.62851	-0.36223	ITGAV
diclofenac-5861	1/100	0.561955	0.561955	0.724338	0.724338	0.628622	-0.3623	S100A4
cotinine-2011	1/100	0.561955	0.561955	0.724338	0.724338	0.633571	-0.36515	GGH
CP-690334-01-4558	1/100	0.561955	0.561955	0.724338	0.724338	0.633845	-0.36531	CXCL1
desoxycortone-3099	1/100	0.561955	0.561955	0.724338	0.724338	0.633924	-0.36535	LY6E
cyclobenzaprine-4834	1/100	0.561955	0.561955	0.724338	0.724338	0.635849	-0.36646	TFF2

danazol-2038	1/100	0.561955	0.561955	0.724338	0.724338	0.636599	-0.36689	IFITM3
dapsone-1827	1/100	0.561955	0.561955	0.724338	0.724338	0.637028	-0.36714	GPX2
depudecin-874	1/100	0.561955	0.561955	0.724338	0.724338	0.637061	-0.36716	TMPRSS3
difenidol-7406	1/100	0.561955	0.561955	0.724338	0.724338	0.637922	-0.36766	AP1S1
denatonium benzoate-5480	1/100	0.561955	0.561955	0.724338	0.724338	0.638736	-0.36812	GPX2
CP-944629-7544	1/100	0.561955	0.561955	0.724338	0.724338	0.639315	-0.36846	IFI6
CP-320650-01-4379	1/100	0.561955	0.561955	0.724338	0.724338	0.640013	-0.36886	SECTM1
copper sulfate-438	1/100	0.561955	0.561955	0.724338	0.724338	0.640434	-0.3691	RNF43
CP-320650-01-3822	1/100	0.561955	0.561955	0.724338	0.724338	0.640719	-0.36927	BMP4
diclofenamide-3366	1/100	0.561955	0.561955	0.724338	0.724338	0.641025	-0.36944	SECTM1
cyproheptadine-1938	1/100	0.561955	0.561955	0.724338	0.724338	0.642486	-0.37029	S100A4
dantrolene-2369	1/100	0.561955	0.561955	0.724338	0.724338	0.643788	-0.37104	C3
dilazep-2294	1/100	0.561955	0.561955	0.724338	0.724338	0.644265	-0.37131	GPX2
deptropine-6523	1/100	0.561955	0.561955	0.724338	0.724338	0.646546	-0.37263	SECTM1
diclofenamide-3027	1/100	0.561955	0.561955	0.724338	0.724338	0.646795	-0.37277	IGFBP2
diflunisal-4210	1/100	0.561955	0.561955	0.724338	0.724338	0.649515	-0.37434	IFI6
dihydroergocristine-7034	1/100	0.561955	0.561955	0.724338	0.724338	0.650376	-0.37483	TMPRSS3
cytochalasin B-1122	1/100	0.561955	0.561955	0.724338	0.724338	0.651245	-0.37533	TXNIP
danazol-1954	1/100	0.561955	0.561955	0.724338	0.724338	0.653457	-0.37661	CTGF
cyproterone-4470	1/100	0.561955	0.561955	0.724338	0.724338	0.655272	-0.37765	IFI6
demecarium bromide-1781	1/100	0.561955	0.561955	0.724338	0.724338	0.656154	-0.37816	C3
denatonium benzoate-6502	1/100	0.561955	0.561955	0.724338	0.724338	0.656457	-0.37834	SECTM1
dextromethorphan-1281	1/100	0.561955	0.561955	0.724338	0.724338	0.657121	-0.37872	FOS
dimethadione-3029	1/100	0.561955	0.561955	0.724338	0.724338	0.659527	-0.38011	IGFBP2
diloxanide-5025	1/100	0.561955	0.561955	0.724338	0.724338	0.661309	-0.38113	SLPI
dienestrol-6208	1/100	0.561955	0.561955	0.724338	0.724338	0.661699	-0.38136	ID3
dexamethasone-6271	1/100	0.561955	0.561955	0.724338	0.724338	0.663795	-0.38257	SECTM1
dextromethorphan-2636	1/100	0.561955	0.561955	0.724338	0.724338	0.665173	-0.38336	BMP4
decamethonium bromide-4174	1/100	0.561955	0.561955	0.724338	0.724338	0.665206	-0.38338	AP1S1
dacarbazine-4480	1/100	0.561955	0.561955	0.724338	0.724338	0.665915	-0.38379	IFI6
dicoumarol-4323	1/100	0.561955	0.561955	0.724338	0.724338	0.667534	-0.38472	IFI6
DL-thiorphan-2752	1/100	0.561955	0.561955	0.724338	0.724338	0.670764	-0.38658	CEACAM6
dicoumarol-3848	1/100	0.561955	0.561955	0.724338	0.724338	0.671851	-0.38721	MMP3
dexibuprofen-5311	1/100	0.561955	0.561955	0.724338	0.724338	0.672154	-0.38739	IFI6
diclofenac-333	1/100	0.561955	0.561955	0.724338	0.724338	0.672451	-0.38756	CTSD
decamethonium bromide-4094	1/100	0.561955	0.561955	0.724338	0.724338	0.672794	-0.38775	IFI6
danazol-1538	1/100	0.561955	0.561955	0.724338	0.724338	0.673393	-0.3881	C3

delsoline-5858	1/100	0.561955	0.561955	0.724338	0.724338	0.674086	-0.3885	CTSD
diloxanide-3062	1/100	0.561955	0.561955	0.724338	0.724338	0.674698	-0.38885	IGFBP2
dinoprost-6308	1/100	0.561955	0.561955	0.724338	0.724338	0.676884	-0.39011	MMP1
cyclic adenosine monophosphate-5108	1/100	0.561955	0.561955	0.724338	0.724338	0.678398	-0.39098	IFI6
dexverapamil-164	1/100	0.561955	0.561955	0.724338	0.724338	0.679316	-0.39151	DEK
diethylstilbestrol-3429	1/100	0.561955	0.561955	0.724338	0.724338	0.679582	-0.39167	ID3
desoxycortone-6476	1/100	0.561955	0.561955	0.724338	0.724338	0.683458	-0.3939	TMPRSS3
diprophylline-1689	1/100	0.561955	0.561955	0.724338	0.724338	0.686987	-0.39593	TSPAN1
dehydrocholic acid-2023	1/100	0.561955	0.561955	0.724338	0.724338	0.687663	-0.39632	ETS2
dinoprost-5409	1/100	0.561955	0.561955	0.724338	0.724338	0.688748	-0.39695	IFI27
digoxigenin-5640	1/100	0.561955	0.561955	0.724338	0.724338	0.688854	-0.39701	BMP4
dimenhydrinate-6352	1/100	0.561955	0.561955	0.724338	0.724338	0.689295	-0.39726	CDH3
diphenhydramine-1830	1/100	0.561955	0.561955	0.724338	0.724338	0.691441	-0.3985	GPX2
dicoumarol-3423	1/100	0.561955	0.561955	0.724338	0.724338	0.695294	-0.40072	AP1S1
dorzolamide-6142	1/100	0.561955	0.561955	0.724338	0.724338	0.696057	-0.40116	SLC12A2
dirithromycin-1712	1/100	0.561955	0.561955	0.724338	0.724338	0.696568	-0.40146	CCL20
dexamethasone-2079	1/100	0.561955	0.561955	0.724338	0.724338	0.698627	-0.40264	PHLDA1
diprophylline-1853	1/100	0.561955	0.561955	0.724338	0.724338	0.698974	-0.40284	LY6E
dicycloverine-1983	1/100	0.561955	0.561955	0.724338	0.724338	0.699572	-0.40319	LYZ
diethylcarbamazine-5066	1/100	0.561955	0.561955	0.724338	0.724338	0.700344	-0.40363	IFI6
diphenylpyraline-2205	1/100	0.561955	0.561955	0.724338	0.724338	0.700508	-0.40373	IFI6
dipyridamole-1934	1/100	0.561955	0.561955	0.724338	0.724338	0.702297	-0.40476	ITGAV
dihydroergocristine-1745	1/100	0.561955	0.561955	0.724338	0.724338	0.702815	-0.40506	HSPB1
diflorasone-4158	1/100	0.561955	0.561955	0.724338	0.724338	0.703332	-0.40535	TCN1
disulfiram-6210	1/100	0.561955	0.561955	0.724338	0.724338	0.703679	-0.40555	LGALS3BP
dl-alpha tocopherol-6616	1/100	0.561955	0.561955	0.724338	0.724338	0.703977	-0.40573	PSMB9
diphenylpyraline-4765	1/100	0.561955	0.561955	0.724338	0.724338	0.704509	-0.40603	IFI27
difenidol-7447	1/100	0.561955	0.561955	0.724338	0.724338	0.7054	-0.40655	GPX2
dehydrocholic acid-1940	1/100	0.561955	0.561955	0.724338	0.724338	0.705808	-0.40678	CKB
diphenylpyraline-3640	1/100	0.561955	0.561955	0.724338	0.724338	0.706274	-0.40705	SLPI
deferoxamine-573	1/100	0.561955	0.561955	0.724338	0.724338	0.706956	-0.40744	PDZK1IP1
diltiazem-1532	1/100	0.561955	0.561955	0.724338	0.724338	0.70736	-0.40768	TFF2
diphemanil metilsulfate-1494	1/100	0.561955	0.561955	0.724338	0.724338	0.708447	-0.4083	LGALS3BP
digoxin-3283	1/100	0.561955	0.561955	0.724338	0.724338	0.709918	-0.40915	BMP4
dipyridamole-1517	1/100	0.561955	0.561955	0.724338	0.724338	0.710303	-0.40937	S100A4
dydrogesterone-4836	1/100	0.561955	0.561955	0.724338	0.724338	0.710371	-0.40941	TFF2
diphemanil metilsulfate-1912	1/100	0.561955	0.561955	0.724338	0.724338	0.710652	-0.40957	CXCL1

dexpanthenol-7455	1/100	0.561955	0.561955	0.724338	0.724338	0.712545	-0.41066	AP1S1
dihydrostreptomycin-6228	1/100	0.561955	0.561955	0.724338	0.724338	0.714266	-0.41166	IGFBP2
dropropizine-5106	1/100	0.561955	0.561955	0.724338	0.724338	0.714336	-0.4117	GPX2
diperodon-4498	1/100	0.561955	0.561955	0.724338	0.724338	0.716664	-0.41304	IFI6
dilazep-4688	1/100	0.561955	0.561955	0.724338	0.724338	0.716905	-0.41318	TMPRSS3
dioxybenzone-4638	1/100	0.561955	0.561955	0.724338	0.724338	0.717156	-0.41332	IFI6
dosulepin-2864	1/100	0.561955	0.561955	0.724338	0.724338	0.717982	-0.4138	TFF2
disulfiram-1369	1/100	0.561955	0.561955	0.724338	0.724338	0.71996	-0.41494	IFITM3
dirithromycin-2863	1/100	0.561955	0.561955	0.724338	0.724338	0.72243	-0.41636	SECTM1
drofenine-6776	1/100	0.561955	0.561955	0.724338	0.724338	0.724976	-0.41783	BMP4
dorzolamide-6259	1/100	0.561955	0.561955	0.724338	0.724338	0.725053	-0.41787	SECTM1
doxylamine-4235	1/100	0.561955	0.561955	0.724338	0.724338	0.726733	-0.41884	LY6E
dicycloverine-1902	1/100	0.561955	0.561955	0.724338	0.724338	0.727052	-0.41902	S100A6
digoxin-5324	1/100	0.561955	0.561955	0.724338	0.724338	0.727146	-0.41908	IFI6
dizocilpine-6223	1/100	0.561955	0.561955	0.724338	0.724338	0.727966	-0.41955	TSPAN1
dihydroergocristine-2895	1/100	0.561955	0.561955	0.724338	0.724338	0.729478	-0.42042	SECTM1
dihydroergotamine-1398	1/100	0.561955	0.561955	0.724338	0.724338	0.733032	-0.42247	HSPB1
erythromycin-6729	1/100	0.561955	0.561955	0.724338	0.724338	0.734675	-0.42342	CEACAM6
dipyridamole-2017	1/100	0.561955	0.561955	0.724338	0.724338	0.735986	-0.42417	SPP1
ellipticine-5779	1/100	0.561955	0.561955	0.724338	0.724338	0.738191	-0.42544	IER2
dosulepin-5986	1/100	0.561955	0.561955	0.724338	0.724338	0.738555	-0.42565	IFI6
doxazosin-6642	1/100	0.561955	0.561955	0.724338	0.724338	0.741528	-0.42737	ID1
domperidone-2655	1/100	0.561955	0.561955	0.724338	0.724338	0.741716	-0.42748	TSPAN1
dizocilpine-1386	1/100	0.561955	0.561955	0.724338	0.724338	0.744783	-0.42924	ITGAV
dropropizine-5531	1/100	0.561955	0.561955	0.724338	0.724338	0.746829	-0.43042	TSPAN1
eldeline-4306	1/100	0.561955	0.561955	0.724338	0.724338	0.747757	-0.43096	IFI6
dydrogesterone-4254	1/100	0.561955	0.561955	0.724338	0.724338	0.747924	-0.43105	PSMB9
dobutamine-6286	1/100	0.561955	0.561955	0.724338	0.724338	0.748973	-0.43166	CTGF
ergocalciferol-6746	1/100	0.561955	0.561955	0.724338	0.724338	0.749608	-0.43202	GPX2
dipyridamole-4656	1/100	0.561955	0.561955	0.724338	0.724338	0.749744	-0.4321	IL32
dimethyloxalylglycine-584	1/100	0.561955	0.561955	0.724338	0.724338	0.751765	-0.43327	CTSE
epiandrosterone-4626	1/100	0.561955	0.561955	0.724338	0.724338	0.752271	-0.43356	TGFBI
estriol-7220	1/100	0.561955	0.561955	0.724338	0.724338	0.753696	-0.43438	LGALS3BP
docosahexaenoic acid ethyl ester-664	1/100	0.561955	0.561955	0.724338	0.724338	0.754052	-0.43459	GPX2
emetine-2145	1/100	0.561955	0.561955	0.724338	0.724338	0.754066	-0.43459	SPP1
drofenine-7129	1/100	0.561955	0.561955	0.724338	0.724338	0.754665	-0.43494	PDIA3
doxepin-6337	1/100	0.561955	0.561955	0.724338	0.724338	0.755197	-0.43525	LY6E

domperidone-1301	1/100	0.561955	0.561955	0.724338	0.724338	0.755452	-0.43539	SLPI
enalapril-7026	1/100	0.561955	0.561955	0.724338	0.724338	0.75811	-0.43692	BMP4
droperidol-4629	1/100	0.561955	0.561955	0.724338	0.724338	0.758378	-0.43708	SPINK1
disopyramide-2408	1/100	0.561955	0.561955	0.724338	0.724338	0.758531	-0.43717	FOS
enoxacin-1597	1/100	0.561955	0.561955	0.724338	0.724338	0.759432	-0.43769	FOS
epivincamine-2775	1/100	0.561955	0.561955	0.724338	0.724338	0.759834	-0.43792	RNF43
erythromycin-1510	1/100	0.561955	0.561955	0.724338	0.724338	0.759994	-0.43801	GPX2
edrophonium chloride-6655	1/100	0.561955	0.561955	0.724338	0.724338	0.760129	-0.43809	TGFBI
enilconazole-3139	1/100	0.561955	0.561955	0.724338	0.724338	0.763597	-0.44009	TSPAN13
doxylamine-1973	1/100	0.561955	0.561955	0.724338	0.724338	0.763853	-0.44023	IFITM3
dydrogesterone-2156	1/100	0.561955	0.561955	0.724338	0.724338	0.764904	-0.44084	FOS
enalapril-2397	1/100	0.561955	0.561955	0.724338	0.724338	0.76515	-0.44098	SLC12A2
dyclonine-7423	1/100	0.561955	0.561955	0.724338	0.724338	0.765474	-0.44117	BMP4
dropropizine-2398	1/100	0.561955	0.561955	0.724338	0.724338	0.766307	-0.44165	SLPI
dopamine-491	1/100	0.561955	0.561955	0.724338	0.724338	0.767516	-0.44235	TSPAN1
doxepin-7415	1/100	0.561955	0.561955	0.724338	0.724338	0.767939	-0.44259	TSPAN1
estradiol-5601	1/100	0.561955	0.561955	0.724338	0.724338	0.769908	-0.44372	SLPI
droperidol-1290	1/100	0.561955	0.561955	0.724338	0.724338	0.770452	-0.44404	LY6E
enoxacin-4655	1/100	0.561955	0.561955	0.724338	0.724338	0.772147	-0.44501	SPINK1
dizocilpine-5746	1/100	0.561955	0.561955	0.724338	0.724338	0.772568	-0.44526	IFI6
estradiol-121	1/100	0.561955	0.561955	0.724338	0.724338	0.772757	-0.44537	ID3
equilin-5620	1/100	0.561955	0.561955	0.724338	0.724338	0.774118	-0.44615	BMP4
etamsylate-7335	1/100	0.561955	0.561955	0.724338	0.724338	0.775255	-0.44681	S100A4
esculin-3390	1/100	0.561955	0.561955	0.724338	0.724338	0.775604	-0.44701	SECTM1
estropipate-6808	1/100	0.561955	0.561955	0.724338	0.724338	0.775919	-0.44719	BMP4
econazole-7427	1/100	0.561955	0.561955	0.724338	0.724338	0.776563	-0.44756	AP1S1
dosulepin-7284	1/100	0.561955	0.561955	0.724338	0.724338	0.777756	-0.44825	TCN1
estradiol-1182	1/100	0.561955	0.561955	0.724338	0.724338	0.778139	-0.44847	TCN1
dyclonine-7022	1/100	0.561955	0.561955	0.724338	0.724338	0.781017	-0.45013	TGFBI
estradiol-1633	1/100	0.561955	0.561955	0.724338	0.724338	0.782076	-0.45074	ID3
erythromycin-1928	1/100	0.561955	0.561955	0.724338	0.724338	0.782263	-0.45084	GPX2
estrone-6448	1/100	0.561955	0.561955	0.724338	0.724338	0.784106	-0.45191	SECTM1
epirizole-1845	1/100	0.561955	0.561955	0.724338	0.724338	0.78421	-0.45197	CTSD
etamsylate-4576	1/100	0.561955	0.561955	0.724338	0.724338	0.784337	-0.45204	TGFBI
estradiol-5205	1/100	0.561955	0.561955	0.724338	0.724338	0.786341	-0.45319	RNF43
esculin-6310	1/100	0.561955	0.561955	0.724338	0.724338	0.78643	-0.45325	MMP1
estradiol-5905	1/100	0.561955	0.561955	0.724338	0.724338	0.787363	-0.45378	DUOX2

etamivan-7260	1/100	0.561955	0.561955	0.724338	0.724338	0.789671	-0.45511	CDH3
estradiol-2668	1/100	0.561955	0.561955	0.724338	0.724338	0.792592	-0.4568	ISG15
etofylline-2256	1/100	0.561955	0.561955	0.724338	0.724338	0.794009	-0.45761	SECTM1
etacrynic acid-3181	1/100	0.561955	0.561955	0.724338	0.724338	0.794143	-0.45769	LYZ
esculetin-7459	1/100	0.561955	0.561955	0.724338	0.724338	0.795082	-0.45823	GPX2
esculetin-3120	1/100	0.561955	0.561955	0.724338	0.724338	0.795435	-0.45844	PLCB4
estriol-5866	1/100	0.561955	0.561955	0.724338	0.724338	0.798353	-0.46012	ITM2C
estradiol-4432	1/100	0.561955	0.561955	0.724338	0.724338	0.799586	-0.46083	IFI6
estrone-4993	1/100	0.561955	0.561955	0.724338	0.724338	0.801572	-0.46197	ID1
ethisterone-2366	1/100	0.561955	0.561955	0.724338	0.724338	0.801919	-0.46217	TSPAN13
ethambutol-1981	1/100	0.561955	0.561955	0.724338	0.724338	0.802057	-0.46225	LYZ
eldeline-2171	1/100	0.561955	0.561955	0.724338	0.724338	0.802749	-0.46265	IFI6
equilin-4659	1/100	0.561955	0.561955	0.724338	0.724338	0.802994	-0.46279	IL32
eucatropine-2556	1/100	0.561955	0.561955	0.724338	0.724338	0.804205	-0.46349	LY6E
estradiol-387	1/100	0.561955	0.561955	0.724338	0.724338	0.805039	-0.46397	CSTB
etacrynic acid-5742	1/100	0.561955	0.561955	0.724338	0.724338	0.806217	-0.46465	IFI6
ellipticine-1765	1/100	0.561955	0.561955	0.724338	0.724338	0.806361	-0.46473	CD14
edrophonium chloride-5001	1/100	0.561955	0.561955	0.724338	0.724338	0.807632	-0.46547	TMPRSS3
ethoxyquin-3764	1/100	0.561955	0.561955	0.724338	0.724338	0.808264	-0.46583	RNF43
ethaverine-6737	1/100	0.561955	0.561955	0.724338	0.724338	0.812379	-0.4682	CTGF
fenbufen-2346	1/100	0.561955	0.561955	0.724338	0.724338	0.814402	-0.46937	C3
estradiol-782	1/100	0.561955	0.561955	0.724338	0.724338	0.814454	-0.4694	GPX2
etynodiol-6678	1/100	0.561955	0.561955	0.724338	0.724338	0.81811	-0.4715	TGFBI
etidronic acid-4564	1/100	0.561955	0.561955	0.724338	0.724338	0.81877	-0.47188	C3
etofenamate-4108	1/100	0.561955	0.561955	0.724338	0.724338	0.818966	-0.472	IFI6
etamivan-2879	1/100	0.561955	0.561955	0.724338	0.724338	0.820859	-0.47309	TFF2
ethisterone-2326	1/100	0.561955	0.561955	0.724338	0.724338	0.820912	-0.47312	SECTM1
fenbufen-4279	1/100	0.561955	0.561955	0.724338	0.724338	0.824772	-0.47534	MMP3
estradiol-6718	1/100	0.561955	0.561955	0.724338	0.724338	0.82546	-0.47574	GPX2
ethisterone-4340	1/100	0.561955	0.561955	0.724338	0.724338	0.825579	-0.47581	TGFBI
fenbufen-3721	1/100	0.561955	0.561955	0.724338	0.724338	0.826786	-0.4765	BMP4
estropipate-6068	1/100	0.561955	0.561955	0.724338	0.724338	0.827962	-0.47718	MMP3
ethambutol-1481	1/100	0.561955	0.561955	0.724338	0.724338	0.829115	-0.47785	BMP4
ethambutol-1900	1/100	0.561955	0.561955	0.724338	0.724338	0.829382	-0.478	ITGAV
etofenamate-2907	1/100	0.561955	0.561955	0.724338	0.724338	0.830129	-0.47843	IFI6
etidronic acid-3325	1/100	0.561955	0.561955	0.724338	0.724338	0.831306	-0.47911	TMPRSS3
etomidate-3519	1/100	0.561955	0.561955	0.724338	0.724338	0.831634	-0.4793	LGALS3BP

famprofazone-3753	1/100	0.561955	0.561955	0.724338	0.724338	0.833553	-0.4804	IFI6
etamivan-7021	1/100	0.561955	0.561955	0.724338	0.724338	0.834214	-0.48079	PGM1
estropipate-4472	1/100	0.561955	0.561955	0.724338	0.724338	0.837015	-0.4824	IFI6
exisulind-314	1/100	0.561955	0.561955	0.724338	0.724338	0.842821	-0.48575	SPINK1
etilefrine-4590	1/100	0.561955	0.561955	0.724338	0.724338	0.84341	-0.48609	CTGF
fenbendazole-3805	1/100	0.561955	0.561955	0.724338	0.724338	0.844382	-0.48665	BMP4
fenoprofen-3714	1/100	0.561955	0.561955	0.724338	0.724338	0.845007	-0.48701	TSPAN1
felbinac-3061	1/100	0.561955	0.561955	0.724338	0.724338	0.845258	-0.48715	GPX2
fludrocortisone-2328	1/100	0.561955	0.561955	0.724338	0.724338	0.846033	-0.4876	C3
ethoxyquin-2559	1/100	0.561955	0.561955	0.724338	0.724338	0.846783	-0.48803	TSPAN13
etofylline-5467	1/100	0.561955	0.561955	0.724338	0.724338	0.847943	-0.4887	LGALS3BP
eucatropine-3416	1/100	0.561955	0.561955	0.724338	0.724338	0.848227	-0.48886	GPX2
eticlopride-4634	1/100	0.561955	0.561955	0.724338	0.724338	0.848676	-0.48912	IL32
etomidate-7460	1/100	0.561955	0.561955	0.724338	0.724338	0.84943	-0.48955	LGALS3BP
flecainide-2557	1/100	0.561955	0.561955	0.724338	0.724338	0.850112	-0.48995	SLC12A2
etodolac-7006	1/100	0.561955	0.561955	0.724338	0.724338	0.852053	-0.49107	S100A4
florfenicol-5300	1/100	0.561955	0.561955	0.724338	0.724338	0.853125	-0.49168	ISG15
etiocholanolone-4298	1/100	0.561955	0.561955	0.724338	0.724338	0.854019	-0.4922	MMP3
flavoxate-2373	1/100	0.561955	0.561955	0.724338	0.724338	0.854454	-0.49245	CKB
etifenin-2477	1/100	0.561955	0.561955	0.724338	0.724338	0.857785	-0.49437	TSPAN13
etynodiol-5024	1/100	0.561955	0.561955	0.724338	0.724338	0.857948	-0.49446	S100A4
famprofazone-3928	1/100	0.561955	0.561955	0.724338	0.724338	0.858132	-0.49457	IFI6
etifenin-4117	1/100	0.561955	0.561955	0.724338	0.724338	0.858202	-0.49461	IFI6
fenofibrate-7474	1/100	0.561955	0.561955	0.724338	0.724338	0.861333	-0.49641	GPX2
fendiline-3190	1/100	0.561955	0.561955	0.724338	0.724338	0.86257	-0.49713	LYZ
fenspiride-2106	1/100	0.561955	0.561955	0.724338	0.724338	0.862572	-0.49713	CCL20
ethotoin-2196	1/100	0.561955	0.561955	0.724338	0.724338	0.862973	-0.49736	FOS
fluvoxamine-3995	1/100	0.561955	0.561955	0.724338	0.724338	0.863914	-0.4979	GPX2
fipexide-5737	1/100	0.561955	0.561955	0.724338	0.724338	0.863978	-0.49794	IFI6
F0447-0125-6401	1/100	0.561955	0.561955	0.724338	0.724338	0.864106	-0.49801	PROM1
flufenamic acid-5059	1/100	0.561955	0.561955	0.724338	0.724338	0.865092	-0.49858	IFI6
fipexide-1560	1/100	0.561955	0.561955	0.724338	0.724338	0.868008	-0.50026	TUBA1A
fenoprofen-3612	1/100	0.561955	0.561955	0.724338	0.724338	0.868372	-0.50047	CTSD
flunisolide-6023	1/100	0.561955	0.561955	0.724338	0.724338	0.86929	-0.501	MMP3
finasteride-3641	1/100	0.561955	0.561955	0.724338	0.724338	0.869849	-0.50132	SLPI
etoposide-1626	1/100	0.561955	0.561955	0.724338	0.724338	0.870575	-0.50174	CKS2
F0447-0125-6396	1/100	0.561955	0.561955	0.724338	0.724338	0.871125	-0.50206	IFI6

flucytosine-3073	1/100	0.561955	0.561955	0.724338	0.724338	0.871255	-0.50213	LY6E
etilefrine-2930	1/100	0.561955	0.561955	0.724338	0.724338	0.871826	-0.50246	MEST
flunisolide-4303	1/100	0.561955	0.561955	0.724338	0.724338	0.872714	-0.50297	IFI6
flunixin-3713	1/100	0.561955	0.561955	0.724338	0.724338	0.875139	-0.50437	C3
fluocinonide-3757	1/100	0.561955	0.561955	0.724338	0.724338	0.876528	-0.50517	MMP3
flumetasone-2551	1/100	0.561955	0.561955	0.724338	0.724338	0.878031	-0.50604	TSPAN13
fenspiride-1422	1/100	0.561955	0.561955	0.724338	0.724338	0.88042	-0.50742	SPP1
fludroxycortide-3679	1/100	0.561955	0.561955	0.724338	0.724338	0.881123	-0.50782	IFI6
fludroxycortide-6039	1/100	0.561955	0.561955	0.724338	0.724338	0.882396	-0.50855	MMP3
ethaverine-3037	1/100	0.561955	0.561955	0.724338	0.724338	0.882522	-0.50863	SPP1
etofylline-5048	1/100	0.561955	0.561955	0.724338	0.724338	0.883333	-0.50909	IFI6
fosfosal-4823	1/100	0.561955	0.561955	0.724338	0.724338	0.885703	-0.51046	RCN1
fasudil-343	1/100	0.561955	0.561955	0.724338	0.724338	0.886773	-0.51108	TGFBI
fipexide-3176	1/100	0.561955	0.561955	0.724338	0.724338	0.888627	-0.51215	LYZ
flumequine-5104	1/100	0.561955	0.561955	0.724338	0.724338	0.889344	-0.51256	IFI6
famotidine-5011	1/100	0.561955	0.561955	0.724338	0.724338	0.889945	-0.51291	IFI27
fenoprofen-4274	1/100	0.561955	0.561955	0.724338	0.724338	0.890064	-0.51297	IFI6
fenspiride-6001	1/100	0.561955	0.561955	0.724338	0.724338	0.892331	-0.51428	IFI6
famotidine-1946	1/100	0.561955	0.561955	0.724338	0.724338	0.893031	-0.51468	GPX2
fulvestrant-5565	1/100	0.561955	0.561955	0.724338	0.724338	0.894897	-0.51576	TMPRSS3
fluoxetine-2453	1/100	0.561955	0.561955	0.724338	0.724338	0.897902	-0.51749	IFI6
fluorocurarine-6083	1/100	0.561955	0.561955	0.724338	0.724338	0.900501	-0.51899	TFF2
fenoterol-5432	1/100	0.561955	0.561955	0.724338	0.724338	0.901261	-0.51943	IFI6
etiocholanolone-6060	1/100	0.561955	0.561955	0.724338	0.724338	0.902145	-0.51994	SPINK1
flucloxacillin-3128	1/100	0.561955	0.561955	0.724338	0.724338	0.903607	-0.52078	IFI6
fluphenazine-5597	1/100	0.561955	0.561955	0.724338	0.724338	0.903874	-0.52093	TMPRSS3
fasudil-436	1/100	0.561955	0.561955	0.724338	0.724338	0.904139	-0.52109	DPEP1
florfenicol-3083	1/100	0.561955	0.561955	0.724338	0.724338	0.904262	-0.52116	CTSD
fludrocortisone-2368	1/100	0.561955	0.561955	0.724338	0.724338	0.904571	-0.52133	C3
fludrocortisone-3785	1/100	0.561955	0.561955	0.724338	0.724338	0.904723	-0.52142	BMP4
fluvastatin-5290	1/100	0.561955	0.561955	0.724338	0.724338	0.906425	-0.5224	AP1S1
fursultiamine-7349	1/100	0.561955	0.561955	0.724338	0.724338	0.908545	-0.52362	IFI6
flumetasone-4734	1/100	0.561955	0.561955	0.724338	0.724338	0.908966	-0.52387	BMP4
flumequine-2276	1/100	0.561955	0.561955	0.724338	0.724338	0.911545	-0.52535	C3
fluticasone-4011	1/100	0.561955	0.561955	0.724338	0.724338	0.91293	-0.52615	MMP3
fulvestrant-6197	1/100	0.561955	0.561955	0.724338	0.724338	0.913037	-0.52621	ID1
fludrocortisone-282	1/100	0.561955	0.561955	0.724338	0.724338	0.916276	-0.52808	S100A4

fulvestrant-1076	1/100	0.561955	0.561955	0.724338	0.724338	0.916493	-0.52821	PRSS23
flecainide-3843	1/100	0.561955	0.561955	0.724338	0.724338	0.918462	-0.52934	TFF2
fluoxetine-6757	1/100	0.561955	0.561955	0.724338	0.724338	0.919074	-0.52969	IFI6
fluticasone-4129	1/100	0.561955	0.561955	0.724338	0.724338	0.91926	-0.5298	BMP4
flurbiprofen-4674	1/100	0.561955	0.561955	0.724338	0.724338	0.91997	-0.53021	SPINK1
flufenamic acid-2104	1/100	0.561955	0.561955	0.724338	0.724338	0.920719	-0.53064	TSPAN8
fludrocortisone-281	1/100	0.561955	0.561955	0.724338	0.724338	0.922028	-0.5314	CTSE
flutamide-3885	1/100	0.561955	0.561955	0.724338	0.724338	0.922299	-0.53155	QPCT
flumequine-5529	1/100	0.561955	0.561955	0.724338	0.724338	0.922731	-0.5318	LGALS3BP
flutamide-3803	1/100	0.561955	0.561955	0.724338	0.724338	0.923314	-0.53214	C3
genistein-1015	1/100	0.561955	0.561955	0.724338	0.724338	0.923841	-0.53244	BMP4
fluphenazine-7234	1/100	0.561955	0.561955	0.724338	0.724338	0.926202	-0.5338	GPX2
furosemide-3197	1/100	0.561955	0.561955	0.724338	0.724338	0.9272	-0.53438	LYZ
fulvestrant-4429	1/100	0.561955	0.561955	0.724338	0.724338	0.928671	-0.53522	PSMB9
gentamicin-2245	1/100	0.561955	0.561955	0.724338	0.724338	0.928773	-0.53528	AP1S1
flucloxacillin-6507	1/100	0.561955	0.561955	0.724338	0.724338	0.929383	-0.53563	LGALS3BP
folic acid-5844	1/100	0.561955	0.561955	0.724338	0.724338	0.929947	-0.53596	LGALS3BP
fulvestrant-367	1/100	0.561955	0.561955	0.724338	0.724338	0.931221	-0.53669	RCN1
fusaric acid-3986	1/100	0.561955	0.561955	0.724338	0.724338	0.93132	-0.53675	GPX2
fludroxycortide-7378	1/100	0.561955	0.561955	0.724338	0.724338	0.93305	-0.53775	IGFBP2
fulvestrant-6965	1/100	0.561955	0.561955	0.724338	0.724338	0.934871	-0.5388	PRSS23
guanethidine-5731	1/100	0.561955	0.561955	0.724338	0.724338	0.935213	-0.53899	IFI6
fulvestrant-1179	1/100	0.561955	0.561955	0.724338	0.724338	0.935556	-0.53919	TSPAN13
geldanamycin-1169	1/100	0.561955	0.561955	0.724338	0.724338	0.936613	-0.5398	IFI6
flunarizine-7252	1/100	0.561955	0.561955	0.724338	0.724338	0.938907	-0.54112	ID3
fulvestrant-1205	1/100	0.561955	0.561955	0.724338	0.724338	0.941259	-0.54248	IL32
furazolidone-4098	1/100	0.561955	0.561955	0.724338	0.724338	0.941711	-0.54274	IFI6
fluorometholone-2509	1/100	0.561955	0.561955	0.724338	0.724338	0.942145	-0.54299	TSPAN13
fluphenazine-6196	1/100	0.561955	0.561955	0.724338	0.724338	0.945495	-0.54492	TSPAN13
fulvestrant-1630	1/100	0.561955	0.561955	0.724338	0.724338	0.945729	-0.54506	PRSS23
fluphenazine-2697	1/100	0.561955	0.561955	0.724338	0.724338	0.945768	-0.54508	IGFBP2
glibenclamide-7202	1/100	0.561955	0.561955	0.724338	0.724338	0.947119	-0.54586	LGALS3BP
genistein-5595	1/100	0.561955	0.561955	0.724338	0.724338	0.947249	-0.54593	SECTM1
flurbiprofen-3095	1/100	0.561955	0.561955	0.724338	0.724338	0.949184	-0.54705	SLPI
fulvestrant-5235	1/100	0.561955	0.561955	0.724338	0.724338	0.95028	-0.54768	TMPRSS3
fulvestrant-5202	1/100	0.561955	0.561955	0.724338	0.724338	0.950389	-0.54774	TMPRSS3
glycopyrronium bromide-3687	1/100	0.561955	0.561955	0.724338	0.724338	0.951492	-0.54838	IFI6

furazolidone-3358	1/100	0.561955	0.561955	0.724338	0.724338	0.951586	-0.54843	C3
fulvestrant-7096	1/100	0.561955	0.561955	0.724338	0.724338	0.951711	-0.5485	LY6E
fusidic acid-2647	1/100	0.561955	0.561955	0.724338	0.724338	0.95524	-0.55054	BMP4
fulvestrant-310	1/100	0.561955	0.561955	0.724338	0.724338	0.955391	-0.55062	LCN2
guanethidine-1554	1/100	0.561955	0.561955	0.724338	0.724338	0.957519	-0.55185	ANXA3
folic acid-1790	1/100	0.561955	0.561955	0.724338	0.724338	0.959333	-0.5529	ZWINT
gentamicin-7237	1/100	0.561955	0.561955	0.724338	0.724338	0.962838	-0.55492	S100A4
gabexate-7357	1/100	0.561955	0.561955	0.724338	0.724338	0.964128	-0.55566	IFI6
glimepiride-4973	1/100	0.561955	0.561955	0.724338	0.724338	0.964855	-0.55608	IFI27
gossypol-2202	1/100	0.561955	0.561955	0.724338	0.724338	0.965705	-0.55657	CTSD
guanadrel-3698	1/100	0.561955	0.561955	0.724338	0.724338	0.966384	-0.55696	MMP3
homochlorcyclizine-5998	1/100	0.561955	0.561955	0.724338	0.724338	0.966819	-0.55721	IFI6
genistein-4459	1/100	0.561955	0.561955	0.724338	0.724338	0.967752	-0.55775	PSMB9
fluphenazine-1237	1/100	0.561955	0.561955	0.724338	0.724338	0.968899	-0.55841	BMP4
geldanamycin-2688	1/100	0.561955	0.561955	0.724338	0.724338	0.970264	-0.5592	IER2
guanfacine-5621	1/100	0.561955	0.561955	0.724338	0.724338	0.97116	-0.55971	BMP4
gabexate-4220	1/100	0.561955	0.561955	0.724338	0.724338	0.974651	-0.56172	TGFBI
guanadrel-3438	1/100	0.561955	0.561955	0.724338	0.724338	0.976694	-0.5629	LGALS3BP
gibberellic acid-4234	1/100	0.561955	0.561955	0.724338	0.724338	0.977351	-0.56328	PSMB9
griseofulvin-4687	1/100	0.561955	0.561955	0.724338	0.724338	0.977837	-0.56356	GPX2
glimepiride-2154	1/100	0.561955	0.561955	0.724338	0.724338	0.977844	-0.56356	TFF2
griseofulvin-3664	1/100	0.561955	0.561955	0.724338	0.724338	0.981212	-0.56551	BMP4
gemfibrozil-1430	1/100	0.561955	0.561955	0.724338	0.724338	0.98162	-0.56574	LYZ
fulvestrant-7490	1/100	0.561955	0.561955	0.724338	0.724338	0.98183	-0.56586	TMPRSS3
genistein-6994	1/100	0.561955	0.561955	0.724338	0.724338	0.98376	-0.56697	GPX2
gliclazide-2870	1/100	0.561955	0.561955	0.724338	0.724338	0.983915	-0.56706	TFF2
genistein-382	1/100	0.561955	0.561955	0.724338	0.724338	0.985862	-0.56819	TCN1
guanfacine-4660	1/100	0.561955	0.561955	0.724338	0.724338	0.986387	-0.56849	IL32
glycopyrronium bromide-7386	1/100	0.561955	0.561955	0.724338	0.724338	0.986579	-0.5686	CDH3
fusaric acid-1308	1/100	0.561955	0.561955	0.724338	0.724338	0.987404	-0.56907	QPCT
ganciclovir-6289	1/100	0.561955	0.561955	0.724338	0.724338	0.988825	-0.56989	PDIA3
griseofulvin-2332	1/100	0.561955	0.561955	0.724338	0.724338	0.991063	-0.57118	TSPAN13
guanfacine-1279	1/100	0.561955	0.561955	0.724338	0.724338	0.992232	-0.57186	SLPI
glipizide-4991	1/100	0.561955	0.561955	0.724338	0.724338	0.992629	-0.57209	TSPAN1
ginkgolide A-4121	1/100	0.561955	0.561955	0.724338	0.724338	0.992775	-0.57217	IFI6
harman-2150	1/100	0.561955	0.561955	0.724338	0.724338	0.994203	-0.57299	RCN1
glipizide-6645	1/100	0.561955	0.561955	0.724338	0.724338	0.996022	-0.57404	C3

halcinonide-7379	1/100	0.561955	0.561955	0.724338	0.724338	0.997599	-0.57495	CXCL1
geldanamycin-972	1/100	0.561955	0.561955	0.724338	0.724338	0.997959	-0.57516	ID1
halcinonide-4703	1/100	0.561955	0.561955	0.724338	0.724338	0.998097	-0.57524	RCN1
hesperetin-6750	1/100	0.561955	0.561955	0.724338	0.724338	0.999552	-0.57608	IFI6
haloperidol-7003	1/100	0.561955	0.561955	0.724338	0.724338	1.000952	-0.57688	GPX2
hecogenin-3457	1/100	0.561955	0.561955	0.724338	0.724338	1.001465	-0.57718	IGFBP2
guanabenz-5703	1/100	0.561955	0.561955	0.724338	0.724338	1.001541	-0.57722	BMP4
glimepiride-6628	1/100	0.561955	0.561955	0.724338	0.724338	1.002296	-0.57766	PSMB9
gemfibrozil-5488	1/100	0.561955	0.561955	0.724338	0.724338	1.003525	-0.57836	RNF43
GW-8510-7067	1/100	0.561955	0.561955	0.724338	0.724338	1.004457	-0.5789	SOX9
halcinonide-3680	1/100	0.561955	0.561955	0.724338	0.724338	1.005673	-0.5796	MMP3
gramine-3999	1/100	0.561955	0.561955	0.724338	0.724338	1.005931	-0.57975	IFI6
heptaminol-6015	1/100	0.561955	0.561955	0.724338	0.724338	1.007857	-0.58086	IFITM1
helveticoside-6047	1/100	0.561955	0.561955	0.724338	0.724338	1.009417	-0.58176	BMP4
haloperidol-1041	1/100	0.561955	0.561955	0.724338	0.724338	1.012274	-0.58341	ANXA3
H-89-6873	1/100	0.561955	0.561955	0.724338	0.724338	1.013187	-0.58393	IFI6
gliclazide-1720	1/100	0.561955	0.561955	0.724338	0.724338	1.013332	-0.58402	SLC12A2
guaifenesin-2569	1/100	0.561955	0.561955	0.724338	0.724338	1.014209	-0.58452	CKB
heptaminol-1866	1/100	0.561955	0.561955	0.724338	0.724338	1.015231	-0.58511	LY6E
gallamine triethiodide-2059	1/100	0.561955	0.561955	0.724338	0.724338	1.017075	-0.58617	TFF2
glafenine-2387	1/100	0.561955	0.561955	0.724338	0.724338	1.017953	-0.58668	SPP1
harmaline-2805	1/100	0.561955	0.561955	0.724338	0.724338	1.021545	-0.58875	TSPAN1
fulvestrant-7495	1/100	0.561955	0.561955	0.724338	0.724338	1.021646	-0.58881	BMP4
heptaminol-1703	1/100	0.561955	0.561955	0.724338	0.724338	1.025018	-0.59075	TMPRSS3
homosalate-3879	1/100	0.561955	0.561955	0.724338	0.724338	1.025794	-0.5912	TFF2
heliotrine-3717	1/100	0.561955	0.561955	0.724338	0.724338	1.025811	-0.59121	RPS14P3
hesperidin-5313	1/100	0.561955	0.561955	0.724338	0.724338	1.027428	-0.59214	AP1S1
genistein-6952	1/100	0.561955	0.561955	0.724338	0.724338	1.027862	-0.59239	IFNGR2
hexylcaine-5768	1/100	0.561955	0.561955	0.724338	0.724338	1.028162	-0.59256	GPX2
hexestrol-2515	1/100	0.561955	0.561955	0.724338	0.724338	1.028664	-0.59285	CCL20
haloperidol-1539	1/100	0.561955	0.561955	0.724338	0.724338	1.029148	-0.59313	TFF2
gossypol-3740	1/100	0.561955	0.561955	0.724338	0.724338	1.030564	-0.59395	BMP4
IC-86621-7518	1/100	0.561955	0.561955	0.724338	0.724338	1.030652	-0.594	BMP4
halcinonide-2185	1/100	0.561955	0.561955	0.724338	0.724338	1.032121	-0.59485	QPCT
harmol-6022	1/100	0.561955	0.561955	0.724338	0.724338	1.032334	-0.59497	TMPRSS3
heliotrine-4739	1/100	0.561955	0.561955	0.724338	0.724338	1.032667	-0.59516	TFF2
harman-4408	1/100	0.561955	0.561955	0.724338	0.724338	1.033466	-0.59562	IFI27

hydroxyzine-1524	1/100	0.561955	0.561955	0.724338	0.724338	1.033703	-0.59576	C3
homosalate-3797	1/100	0.561955	0.561955	0.724338	0.724338	1.034791	-0.59638	RNF43
harpagoside-7355	1/100	0.561955	0.561955	0.724338	0.724338	1.034952	-0.59648	IFI6
gabexate-4804	1/100	0.561955	0.561955	0.724338	0.724338	1.036016	-0.59709	SECTM1
hexestrol-6077	1/100	0.561955	0.561955	0.724338	0.724338	1.03695	-0.59763	BMP4
haloperidol-983	1/100	0.561955	0.561955	0.724338	0.724338	1.03757	-0.59799	IGFBP2
hydrocortisone-5284	1/100	0.561955	0.561955	0.724338	0.724338	1.03794	-0.5982	TSPAN1
hemicholinium-3216	1/100	0.561955	0.561955	0.724338	0.724338	1.040861	-0.59988	BMP4
harmalol-2892	1/100	0.561955	0.561955	0.724338	0.724338	1.041772	-0.60041	C3
halofantrine-3130	1/100	0.561955	0.561955	0.724338	0.724338	1.042349	-0.60074	LY6E
homosalate-4533	1/100	0.561955	0.561955	0.724338	0.724338	1.042581	-0.60087	TGFBI
hydrastinine-2283	1/100	0.561955	0.561955	0.724338	0.724338	1.042801	-0.601	SLPI
imidurea-5481	1/100	0.561955	0.561955	0.724338	0.724338	1.043644	-0.60149	IFNGR2
harmalol-5495	1/100	0.561955	0.561955	0.724338	0.724338	1.043667	-0.6015	IFI6
heptaminol-7313	1/100	0.561955	0.561955	0.724338	0.724338	1.047068	-0.60346	TCN1
homochlorcyclizine-2386	1/100	0.561955	0.561955	0.724338	0.724338	1.047101	-0.60348	ID3
genistein-703	1/100	0.561955	0.561955	0.724338	0.724338	1.04764	-0.60379	PIGR
heptaminol-1825	1/100	0.561955	0.561955	0.724338	0.724338	1.049274	-0.60473	IL32
harpagoside-6636	1/100	0.561955	0.561955	0.724338	0.724338	1.051354	-0.60593	C3
hydrastine hydrochloride-6011	1/100	0.561955	0.561955	0.724338	0.724338	1.052894	-0.60682	IFI6
imidurea-5062	1/100	0.561955	0.561955	0.724338	0.724338	1.056169	-0.60871	LGALS3BP
hymecromone-4623	1/100	0.561955	0.561955	0.724338	0.724338	1.056229	-0.60874	OLFM4
haloperidol-1628	1/100	0.561955	0.561955	0.724338	0.724338	1.057593	-0.60953	TSPAN1
harmine-2750	1/100	0.561955	0.561955	0.724338	0.724338	1.058101	-0.60982	ATP1B1
imipramine-1807	1/100	0.561955	0.561955	0.724338	0.724338	1.059343	-0.61053	IFI6
helveticoside-4327	1/100	0.561955	0.561955	0.724338	0.724338	1.059551	-0.61065	LY6E
hesperetin-1947	1/100	0.561955	0.561955	0.724338	0.724338	1.059875	-0.61084	GPX2
haloperidol-6923	1/100	0.561955	0.561955	0.724338	0.724338	1.060366	-0.61112	LY6E
halofantrine-6509	1/100	0.561955	0.561955	0.724338	0.724338	1.061971	-0.61205	SECTM1
hydrastine hydrochloride-2889	1/100	0.561955	0.561955	0.724338	0.724338	1.062858	-0.61256	BMP4
hydrochlorothiazide-4970	1/100	0.561955	0.561955	0.724338	0.724338	1.068954	-0.61607	IFI27
hexylcaine-2708	1/100	0.561955	0.561955	0.724338	0.724338	1.068973	-0.61608	ISG15
hexamethonium bromide-1482	1/100	0.561955	0.561955	0.724338	0.724338	1.069344	-0.6163	BMP4
hydralazine-2349	1/100	0.561955	0.561955	0.724338	0.724338	1.06983	-0.61658	SLC12A2
hydroxyachillin-4797	1/100	0.561955	0.561955	0.724338	0.724338	1.071601	-0.6176	IFI6
hesperetin-2031	1/100	0.561955	0.561955	0.724338	0.724338	1.07336	-0.61861	MEST
isoniazid-2246	1/100	0.561955	0.561955	0.724338	0.724338	1.073943	-0.61895	GPX2

hymecromone-3383	1/100	0.561955	0.561955	0.724338	0.724338	1.074872	-0.61948	AP1S1
hydroquinine-2767	1/100	0.561955	0.561955	0.724338	0.724338	1.076957	-0.62069	LGALS3BP
hexetidine-5420	1/100	0.561955	0.561955	0.724338	0.724338	1.077417	-0.62095	IFI27
hymecromone-5684	1/100	0.561955	0.561955	0.724338	0.724338	1.07793	-0.62125	IFITM1
imipenem-7294	1/100	0.561955	0.561955	0.724338	0.724338	1.078512	-0.62158	TCN1
hexetidine-6319	1/100	0.561955	0.561955	0.724338	0.724338	1.079829	-0.62234	GPX2
iodixanol-4848	1/100	0.561955	0.561955	0.724338	0.724338	1.080662	-0.62282	TFF2
ketanserin-3209	1/100	0.561955	0.561955	0.724338	0.724338	1.083141	-0.62425	LYZ
isocorydine-4505	1/100	0.561955	0.561955	0.724338	0.724338	1.083188	-0.62428	RNF43
H-89-6878	1/100	0.561955	0.561955	0.724338	0.724338	1.083884	-0.62468	RNF43
ketotifen-7199	1/100	0.561955	0.561955	0.724338	0.724338	1.085304	-0.6255	GPX2
hesperetin-1531	1/100	0.561955	0.561955	0.724338	0.724338	1.085541	-0.62563	TFF2
hycanthone-1614	1/100	0.561955	0.561955	0.724338	0.724338	1.085776	-0.62577	SLC12A2
hydroflumethiazide-1851	1/100	0.561955	0.561955	0.724338	0.724338	1.086544	-0.62621	IFITM3
indoprofen-4832	1/100	0.561955	0.561955	0.724338	0.724338	1.087362	-0.62668	SECTM1
iobenguane-7299	1/100	0.561955	0.561955	0.724338	0.724338	1.087914	-0.627	LGALS3BP
ikarugamycin-974	1/100	0.561955	0.561955	0.724338	0.724338	1.090829	-0.62868	RNF43
hydrastinine-1436	1/100	0.561955	0.561955	0.724338	0.724338	1.090965	-0.62876	LY6E
imipenem-5997	1/100	0.561955	0.561955	0.724338	0.724338	1.092247	-0.6295	BMP4
hexamethonium bromide-1901	1/100	0.561955	0.561955	0.724338	0.724338	1.092898	-0.62987	IGFBP2
indometacin-5049	1/100	0.561955	0.561955	0.724338	0.724338	1.093394	-0.63016	TSPAN1
isoetarine-3451	1/100	0.561955	0.561955	0.724338	0.724338	1.095175	-0.63119	PGM1
hydroxyachillin-2157	1/100	0.561955	0.561955	0.724338	0.724338	1.098665	-0.6332	TFF2
isometheptene-5502	1/100	0.561955	0.561955	0.724338	0.724338	1.099835	-0.63387	RNF43
hydroxyzine-2024	1/100	0.561955	0.561955	0.724338	0.724338	1.099841	-0.63388	RPS14P3
ioxaglic acid-3528	1/100	0.561955	0.561955	0.724338	0.724338	1.100161	-0.63406	SECTM1
idoxuridine-4200	1/100	0.561955	0.561955	0.724338	0.724338	1.101836	-0.63503	LY6E
IC-86621-7548	1/100	0.561955	0.561955	0.724338	0.724338	1.102067	-0.63516	BMP4
indometacin-503	1/100	0.561955	0.561955	0.724338	0.724338	1.103208	-0.63582	SLPI
isoconazole-5857	1/100	0.561955	0.561955	0.724338	0.724338	1.103567	-0.63602	ITM2C
indapamide-4335	1/100	0.561955	0.561955	0.724338	0.724338	1.104474	-0.63655	TGFBI
iloprost-488	1/100	0.561955	0.561955	0.724338	0.724338	1.105398	-0.63708	LGALS3BP
ioversol-3026	1/100	0.561955	0.561955	0.724338	0.724338	1.105593	-0.63719	IGFBP2
indoprofen-3345	1/100	0.561955	0.561955	0.724338	0.724338	1.107562	-0.63833	BMP4
isoconazole-1372	1/100	0.561955	0.561955	0.724338	0.724338	1.108034	-0.6386	ZWINT
iobenguane-2878	1/100	0.561955	0.561955	0.724338	0.724338	1.108361	-0.63879	C3
isopropamide iodide-3461	1/100	0.561955	0.561955	0.724338	0.724338	1.11144	-0.64056	S100A4

isoetarine-2711	1/100	0.561955	0.561955	0.724338	0.724338	1.113498	-0.64175	LY6E
kawain-2299	1/100	0.561955	0.561955	0.724338	0.724338	1.114957	-0.64259	TGFBI
iopromide-3481	1/100	0.561955	0.561955	0.724338	0.724338	1.11594	-0.64315	TMPRSS3
kanamycin-4625	1/100	0.561955	0.561955	0.724338	0.724338	1.116471	-0.64346	IFI6
isoxsuprine-1904	1/100	0.561955	0.561955	0.724338	0.724338	1.117305	-0.64394	SLC12A2
ketanserin-6649	1/100	0.561955	0.561955	0.724338	0.724338	1.117572	-0.64409	PDIA3
kawain-3670	1/100	0.561955	0.561955	0.724338	0.724338	1.117621	-0.64412	IFI6
isosorbide-6038	1/100	0.561955	0.561955	0.724338	0.724338	1.121363	-0.64628	MMP3
iproniazid-2288	1/100	0.561955	0.561955	0.724338	0.724338	1.122572	-0.64698	MMP3
ifosfamide-6279	1/100	0.561955	0.561955	0.724338	0.724338	1.122966	-0.6472	SECTM1
iopamidol-3473	1/100	0.561955	0.561955	0.724338	0.724338	1.123671	-0.64761	LGALS3BP
isopropamide iodide-6781	1/100	0.561955	0.561955	0.724338	0.724338	1.124406	-0.64803	GPX2
hydrocortisone-3284	1/100	0.561955	0.561955	0.724338	0.724338	1.124415	-0.64804	SECTM1
khellin-4987	1/100	0.561955	0.561955	0.724338	0.724338	1.12464	-0.64817	IFI27
isocarboxazid-3684	1/100	0.561955	0.561955	0.724338	0.724338	1.125559	-0.6487	IFI6
ketoprofen-3626	1/100	0.561955	0.561955	0.724338	0.724338	1.126054	-0.64898	RNF43
hyoscyamine-5524	1/100	0.561955	0.561955	0.724338	0.724338	1.126888	-0.64946	TSPAN1
iocetamic acid-3361	1/100	0.561955	0.561955	0.724338	0.724338	1.126962	-0.64951	BMP4
isocorydine-2780	1/100	0.561955	0.561955	0.724338	0.724338	1.130268	-0.65141	GPX2
isoniazid-2083	1/100	0.561955	0.561955	0.724338	0.724338	1.130765	-0.6517	LGALS3BP
ipratropium bromide-2762	1/100	0.561955	0.561955	0.724338	0.724338	1.136338	-0.65491	GPX2
hydrochlorothiazide-1987	1/100	0.561955	0.561955	0.724338	0.724338	1.137816	-0.65576	SLC12A2
karakoline-2203	1/100	0.561955	0.561955	0.724338	0.724338	1.140624	-0.65738	IFI6
khellin-1922	1/100	0.561955	0.561955	0.724338	0.724338	1.141319	-0.65778	PRSS23
iopanoic acid-3527	1/100	0.561955	0.561955	0.724338	0.724338	1.143168	-0.65885	BMP4
kawain-2337	1/100	0.561955	0.561955	0.724338	0.724338	1.143316	-0.65893	FOS
ketoconazole-2640	1/100	0.561955	0.561955	0.724338	0.724338	1.143664	-0.65913	ID1
kaempferol-6157	1/100	0.561955	0.561955	0.724338	0.724338	1.143756	-0.65918	GPX2
khellin-1504	1/100	0.561955	0.561955	0.724338	0.724338	1.145523	-0.6602	BMP4
idoxuridine-1980	1/100	0.561955	0.561955	0.724338	0.724338	1.145845	-0.66039	ITGAV
lincomycin-5992	1/100	0.561955	0.561955	0.724338	0.724338	1.147318	-0.66124	RNF43
ketoprofen-4286	1/100	0.561955	0.561955	0.724338	0.724338	1.149163	-0.6623	MMP3
ioxaglic acid-2966	1/100	0.561955	0.561955	0.724338	0.724338	1.149539	-0.66252	IFI6
isocorydine-6843	1/100	0.561955	0.561955	0.724338	0.724338	1.150774	-0.66323	GPX2
lactobionic acid-3246	1/100	0.561955	0.561955	0.724338	0.724338	1.154448	-0.66535	SECTM1
letrozole-4824	1/100	0.561955	0.561955	0.724338	0.724338	1.154506	-0.66538	RNF43
kawain-7369	1/100	0.561955	0.561955	0.724338	0.724338	1.154948	-0.66564	SECTM1

kinetin-6073	1/100	0.561955	0.561955	0.724338	0.724338	1.155721	-0.66608	TFF2
IC-86621-7513	1/100	0.561955	0.561955	0.724338	0.724338	1.156359	-0.66645	TFF2
isotretinoin-7438	1/100	0.561955	0.561955	0.724338	0.724338	1.157055	-0.66685	CKB
isoxicam-7028	1/100	0.561955	0.561955	0.724338	0.724338	1.158562	-0.66772	PGM1
ipratropium bromide-5823	1/100	0.561955	0.561955	0.724338	0.724338	1.160698	-0.66895	LGALS3BP
isoniazid-1399	1/100	0.561955	0.561955	0.724338	0.724338	1.161123	-0.66919	PIGR
levonorgestrel-3708	1/100	0.561955	0.561955	0.724338	0.724338	1.161377	-0.66934	IFI6
isradipine-6508	1/100	0.561955	0.561955	0.724338	0.724338	1.162561	-0.67002	GPX2
lansoprazole-3529	1/100	0.561955	0.561955	0.724338	0.724338	1.162813	-0.67017	RNF43
lasalocid-6639	1/100	0.561955	0.561955	0.724338	0.724338	1.165733	-0.67185	ID3
L-methionine sulfoximine-2831	1/100	0.561955	0.561955	0.724338	0.724338	1.168712	-0.67357	TFF2
lidoflazine-6278	1/100	0.561955	0.561955	0.724338	0.724338	1.168822	-0.67363	SECTM1
lasalocid-4985	1/100	0.561955	0.561955	0.724338	0.724338	1.173291	-0.67621	ID3
josamycin-2034	1/100	0.561955	0.561955	0.724338	0.724338	1.173952	-0.67659	CTSD
labetalol-3167	1/100	0.561955	0.561955	0.724338	0.724338	1.174086	-0.67667	LYZ
levopropoxyphene-5083	1/100	0.561955	0.561955	0.724338	0.724338	1.174961	-0.67717	GPX2
lymecycline-5994	1/100	0.561955	0.561955	0.724338	0.724338	1.17512	-0.67726	RNF43
iproniazid-5458	1/100	0.561955	0.561955	0.724338	0.724338	1.175455	-0.67745	LGALS3BP
letrozole-2916	1/100	0.561955	0.561955	0.724338	0.724338	1.178054	-0.67895	IFI6
ketotifen-5842	1/100	0.561955	0.561955	0.724338	0.724338	1.179703	-0.6799	TSPAN1
levodopa-1472	1/100	0.561955	0.561955	0.724338	0.724338	1.180786	-0.68053	RNF43
indometacin-452	1/100	0.561955	0.561955	0.724338	0.724338	1.18099	-0.68064	CTGF
lovastatin-6633	1/100	0.561955	0.561955	0.724338	0.724338	1.185053	-0.68299	CTGF
laudanosine-7270	1/100	0.561955	0.561955	0.724338	0.724338	1.185719	-0.68337	SECTM1
latamoxef-5609	1/100	0.561955	0.561955	0.724338	0.724338	1.185852	-0.68345	IFNGR2
lisuride-6682	1/100	0.561955	0.561955	0.724338	0.724338	1.186669	-0.68392	PSMB9
lorglumide-5619	1/100	0.561955	0.561955	0.724338	0.724338	1.189229	-0.68539	BMP4
levodopa-1972	1/100	0.561955	0.561955	0.724338	0.724338	1.190605	-0.68619	ITGAV
levothyroxine sodium-4069	1/100	0.561955	0.561955	0.724338	0.724338	1.190938	-0.68638	IFI6
ketoprofen-2316	1/100	0.561955	0.561955	0.724338	0.724338	1.192677	-0.68738	TFF2
levamisole-2257	1/100	0.561955	0.561955	0.724338	0.724338	1.193642	-0.68794	C3
mafenide-2124	1/100	0.561955	0.561955	0.724338	0.724338	1.194624	-0.6885	TSPAN8
lorglumide-4658	1/100	0.561955	0.561955	0.724338	0.724338	1.195023	-0.68873	IL32
kinetin-6813	1/100	0.561955	0.561955	0.724338	0.724338	1.195117	-0.68879	GPX2
maprotiline-6676	1/100	0.561955	0.561955	0.724338	0.724338	1.197877	-0.69038	PSMB9
lobelanidine-2897	1/100	0.561955	0.561955	0.724338	0.724338	1.197918	-0.6904	TFF2
LM-1685-612	1/100	0.561955	0.561955	0.724338	0.724338	1.199362	-0.69123	TCN1

lidocaine-1917	1/100	0.561955	0.561955	0.724338	0.724338	1.199441	-0.69128	CTSD
khellin-2004	1/100	0.561955	0.561955	0.724338	0.724338	1.202971	-0.69331	ISG15
LY-294002-1641	1/100	0.561955	0.561955	0.724338	0.724338	1.203306	-0.69351	S100A4
lincomycin-7411	1/100	0.561955	0.561955	0.724338	0.724338	1.203622	-0.69369	TFF3
lasalocid-3360	1/100	0.561955	0.561955	0.724338	0.724338	1.206828	-0.69554	QPCT
lanatoside C-3963	1/100	0.561955	0.561955	0.724338	0.724338	1.206901	-0.69558	BMP4
laudanosine-1741	1/100	0.561955	0.561955	0.724338	0.724338	1.20725	-0.69578	IER2
LM-1685-253	1/100	0.561955	0.561955	0.724338	0.724338	1.209138	-0.69687	DPEP1
levonorgestrel-4730	1/100	0.561955	0.561955	0.724338	0.724338	1.209528	-0.69709	S100A4
mebeverine-7147	1/100	0.561955	0.561955	0.724338	0.724338	1.209641	-0.69716	IFI6
lincomycin-2380	1/100	0.561955	0.561955	0.724338	0.724338	1.21024	-0.6975	C3
lobeline-6258	1/100	0.561955	0.561955	0.724338	0.724338	1.210651	-0.69774	S100A4
liothyronine-2984	1/100	0.561955	0.561955	0.724338	0.724338	1.210808	-0.69783	CTSD
lomustine-7089	1/100	0.561955	0.561955	0.724338	0.724338	1.211282	-0.6981	TXNIP
levodopa-4571	1/100	0.561955	0.561955	0.724338	0.724338	1.211471	-0.69821	TSPAN1
levocabastine-7009	1/100	0.561955	0.561955	0.724338	0.724338	1.212901	-0.69904	BMP4
lobelanidine-1747	1/100	0.561955	0.561955	0.724338	0.724338	1.213059	-0.69913	ANXA3
LY-294002-1216	1/100	0.561955	0.561955	0.724338	0.724338	1.213548	-0.69941	PSMB9
lomustine-7045	1/100	0.561955	0.561955	0.724338	0.724338	1.213967	-0.69965	TXNIP
levcycloserine-4524	1/100	0.561955	0.561955	0.724338	0.724338	1.214246	-0.69981	GPX2
lidoflazine-5804	1/100	0.561955	0.561955	0.724338	0.724338	1.214277	-0.69983	GPX2
meclofenamic acid-7038	1/100	0.561955	0.561955	0.724338	0.724338	1.222029	-0.7043	LGALS3BP
mecamylamine-3525	1/100	0.561955	0.561955	0.724338	0.724338	1.222201	-0.7044	BMP4
lisuride-5028	1/100	0.561955	0.561955	0.724338	0.724338	1.223912	-0.70538	IFI27
loracarbef-2970	1/100	0.561955	0.561955	0.724338	0.724338	1.224409	-0.70567	QPCT
LY-294002-5236	1/100	0.561955	0.561955	0.724338	0.724338	1.224859	-0.70593	QPCT
meclocycline-6637	1/100	0.561955	0.561955	0.724338	0.724338	1.225471	-0.70628	PSMB9
lomefloxacin-3723	1/100	0.561955	0.561955	0.724338	0.724338	1.228916	-0.70827	RPS14P3
levamisole-2094	1/100	0.561955	0.561955	0.724338	0.724338	1.23018	-0.70899	TCN1
levcycloserine-3789	1/100	0.561955	0.561955	0.724338	0.724338	1.231237	-0.7096	MMP3
LY-294002-5970	1/100	0.561955	0.561955	0.724338	0.724338	1.231239	-0.7096	CXCL1
lysergol-3261	1/100	0.561955	0.561955	0.724338	0.724338	1.232212	-0.71017	SECTM1
lobelanidine-5500	1/100	0.561955	0.561955	0.724338	0.724338	1.233268	-0.71077	RNF43
LY-294002-6945	1/100	0.561955	0.561955	0.724338	0.724338	1.233631	-0.71098	IFNGR2
lomustine-7094	1/100	0.561955	0.561955	0.724338	0.724338	1.23505	-0.7118	TXNIP
megestrol-6468	1/100	0.561955	0.561955	0.724338	0.724338	1.235832	-0.71225	ID1
loperamide-1533	1/100	0.561955	0.561955	0.724338	0.724338	1.236074	-0.71239	RNF43

mefenamic acid-1821	1/100	0.561955	0.561955	0.724338	0.724338	1.236479	-0.71262	IFI6
loracarbef-5492	1/100	0.561955	0.561955	0.724338	0.724338	1.237444	-0.71318	QPCT
memantine-4017	1/100	0.561955	0.561955	0.724338	0.724338	1.242602	-0.71615	MMP3
mefloquine-5724	1/100	0.561955	0.561955	0.724338	0.724338	1.244071	-0.717	IFI6
mepenzolate bromide-3829	1/100	0.561955	0.561955	0.724338	0.724338	1.244163	-0.71705	S100A4
memantine-2934	1/100	0.561955	0.561955	0.724338	0.724338	1.244371	-0.71717	ISG15
LY-294002-258	1/100	0.561955	0.561955	0.724338	0.724338	1.244667	-0.71734	ID3
LY-294002-6987	1/100	0.561955	0.561955	0.724338	0.724338	1.24468	-0.71735	S100A4
LY-294002-1168	1/100	0.561955	0.561955	0.724338	0.724338	1.244986	-0.71753	IFI6
levothyroxine sodium-3249	1/100	0.561955	0.561955	0.724338	0.724338	1.245578	-0.71787	SECTM1
mafenide-1441	1/100	0.561955	0.561955	0.724338	0.724338	1.246122	-0.71818	LY6E
loracarbef-3532	1/100	0.561955	0.561955	0.724338	0.724338	1.248082	-0.71931	ETS2
lumicolchicine-1317	1/100	0.561955	0.561955	0.724338	0.724338	1.250079	-0.72046	QPCT
LY-294002-1054	1/100	0.561955	0.561955	0.724338	0.724338	1.251164	-0.72109	TSPAN1
luteolin-3379	1/100	0.561955	0.561955	0.724338	0.724338	1.252391	-0.7218	ID1
lycorine-3891	1/100	0.561955	0.561955	0.724338	0.724338	1.252868	-0.72207	RCN1
levopropoxyphene-5503	1/100	0.561955	0.561955	0.724338	0.724338	1.253046	-0.72217	QPCT
meclocycline-1341	1/100	0.561955	0.561955	0.724338	0.724338	1.25469	-0.72312	CTSH
meclozine-5607	1/100	0.561955	0.561955	0.724338	0.724338	1.254818	-0.72319	TMPRSS3
merbromin-7398	1/100	0.561955	0.561955	0.724338	0.724338	1.255972	-0.72386	IFI6
LY-294002-1664	1/100	0.561955	0.561955	0.724338	0.724338	1.257866	-0.72495	BMP4
LY-294002-1652	1/100	0.561955	0.561955	0.724338	0.724338	1.258876	-0.72553	S100A4
meclofenamic acid-2291	1/100	0.561955	0.561955	0.724338	0.724338	1.259924	-0.72614	C3
LY-294002-1177	1/100	0.561955	0.561955	0.724338	0.724338	1.260744	-0.72661	ANXA3
meclofenoxate-2546	1/100	0.561955	0.561955	0.724338	0.724338	1.261542	-0.72707	LY6E
mephenesin-2304	1/100	0.561955	0.561955	0.724338	0.724338	1.262838	-0.72782	TFF2
LY-294002-328	1/100	0.561955	0.561955	0.724338	0.724338	1.263585	-0.72825	DPEP1
mebeverine-3193	1/100	0.561955	0.561955	0.724338	0.724338	1.264411	-0.72872	LYZ
mepyramine-5869	1/100	0.561955	0.561955	0.724338	0.724338	1.265449	-0.72932	CTSD
lynestrenol-2037	1/100	0.561955	0.561955	0.724338	0.724338	1.267799	-0.73068	PIGR
lymecycline-7291	1/100	0.561955	0.561955	0.724338	0.724338	1.267814	-0.73068	PDIA3
methanthelinium bromide-6254	1/100	0.561955	0.561955	0.724338	0.724338	1.273419	-0.73391	S100A6
meprylcaine-3544	1/100	0.561955	0.561955	0.724338	0.724338	1.273433	-0.73392	S100A4
metanephrine-6734	1/100	0.561955	0.561955	0.724338	0.724338	1.274722	-0.73467	IFI6
methapyrilene-6644	1/100	0.561955	0.561955	0.724338	0.724338	1.275192	-0.73494	C3
meglumine-3068	1/100	0.561955	0.561955	0.724338	0.724338	1.2767	-0.73581	CTSD
merbromin-3700	1/100	0.561955	0.561955	0.724338	0.724338	1.276856	-0.7359	IFI6

mefenamic acid-1863	1/100	0.561955	0.561955	0.724338	0.724338	1.278877	-0.73706	LY6E
lynestrenol-1953	1/100	0.561955	0.561955	0.724338	0.724338	1.279071	-0.73717	IFI6
mepacrine-3179	1/100	0.561955	0.561955	0.724338	0.724338	1.279841	-0.73762	LYZ
LY-294002-501	1/100	0.561955	0.561955	0.724338	0.724338	1.27986	-0.73763	MMP12
mefexamide-7478	1/100	0.561955	0.561955	0.724338	0.724338	1.281479	-0.73856	GPX2
lobeline-2763	1/100	0.561955	0.561955	0.724338	0.724338	1.281927	-0.73882	IGFBP2
medrysone-3705	1/100	0.561955	0.561955	0.724338	0.724338	1.28223	-0.73899	IFI6
mebhydrolin-3269	1/100	0.561955	0.561955	0.724338	0.724338	1.282333	-0.73905	PDIA3
meglumine-6685	1/100	0.561955	0.561955	0.724338	0.724338	1.28244	-0.73911	GPX2
lynestrenol-5355	1/100	0.561955	0.561955	0.724338	0.724338	1.282469	-0.73913	ID1
mephenytoin-3580	1/100	0.561955	0.561955	0.724338	0.724338	1.282817	-0.73933	S100A4
medrysone-4727	1/100	0.561955	0.561955	0.724338	0.724338	1.284713	-0.74042	IFI6
methacholine chloride-5773	1/100	0.561955	0.561955	0.724338	0.724338	1.285662	-0.74097	IFI6
metampicillin-5540	1/100	0.561955	0.561955	0.724338	0.724338	1.286534	-0.74147	ETS2
mefenamic acid-5109	1/100	0.561955	0.561955	0.724338	0.724338	1.286776	-0.74161	PDIA3
meclofenamic acid-1445	1/100	0.561955	0.561955	0.724338	0.724338	1.291948	-0.74459	IFITM3
mesalazine-3584	1/100	0.561955	0.561955	0.724338	0.724338	1.293319	-0.74538	TFF3
LY-294002-5942	1/100	0.561955	0.561955	0.724338	0.724338	1.294884	-0.74628	CXCL1
memantine-4135	1/100	0.561955	0.561955	0.724338	0.724338	1.295049	-0.74638	TCN1
mestranol-4792	1/100	0.561955	0.561955	0.724338	0.724338	1.296288	-0.74709	IFI6
meglumine-6445	1/100	0.561955	0.561955	0.724338	0.724338	1.297925	-0.74804	CTSE
meteneprost-7552	1/100	0.561955	0.561955	0.724338	0.724338	1.300676	-0.74962	IFI6
mephenesin-4698	1/100	0.561955	0.561955	0.724338	0.724338	1.300838	-0.74972	RCN1
metanephrine-5334	1/100	0.561955	0.561955	0.724338	0.724338	1.301759	-0.75025	TMPRSS3
mepenzolate bromide-4304	1/100	0.561955	0.561955	0.724338	0.724338	1.305178	-0.75222	IFI6
mefenamic acid-1699	1/100	0.561955	0.561955	0.724338	0.724338	1.308178	-0.75395	PDIA3
metamizole sodium-3835	1/100	0.561955	0.561955	0.724338	0.724338	1.30997	-0.75498	SLPI
mephentermine-4707	1/100	0.561955	0.561955	0.724338	0.724338	1.31075	-0.75543	TMPRSS3
methylbenzethonium chloride-3943	1/100	0.561955	0.561955	0.724338	0.724338	1.311733	-0.756	AP1S1
metaraminol-4692	1/100	0.561955	0.561955	0.724338	0.724338	1.312179	-0.75625	SPINK1
mesoridazine-1725	1/100	0.561955	0.561955	0.724338	0.724338	1.314439	-0.75756	RPS14P3
metacycline-2901	1/100	0.561955	0.561955	0.724338	0.724338	1.314784	-0.75775	ISG15
mephenesin-2342	1/100	0.561955	0.561955	0.724338	0.724338	1.315101	-0.75794	TSPAN13
mestranol-3346	1/100	0.561955	0.561955	0.724338	0.724338	1.315286	-0.75804	C3
mepyramine-1568	1/100	0.561955	0.561955	0.724338	0.724338	1.316026	-0.75847	IFITM3
methotrexate-1957	1/100	0.561955	0.561955	0.724338	0.724338	1.316633	-0.75882	ITGAV
merbromin-4722	1/100	0.561955	0.561955	0.724338	0.724338	1.317461	-0.7593	RCN1

mercaptopurine-334	1/100	0.561955	0.561955	0.724338	0.724338	1.319914	-0.76071	PSMB9
methylbenzethonium chloride-6045	1/100	0.561955	0.561955	0.724338	0.724338	1.320947	-0.76131	GPX2
methapyrilene-3205	1/100	0.561955	0.561955	0.724338	0.724338	1.321243	-0.76148	LYZ
methocarbamol-2274	1/100	0.561955	0.561955	0.724338	0.724338	1.322349	-0.76211	TFF2
mephenytoin-6158	1/100	0.561955	0.561955	0.724338	0.724338	1.326963	-0.76477	GPX2
meprylcaine-6123	1/100	0.561955	0.561955	0.724338	0.724338	1.328896	-0.76589	GPX2
metamizole sodium-4310	1/100	0.561955	0.561955	0.724338	0.724338	1.330123	-0.76659	IFI6
metformin-61	1/100	0.561955	0.561955	0.724338	0.724338	1.336349	-0.77018	GPX2
meropenem-7180	1/100	0.561955	0.561955	0.724338	0.724338	1.336582	-0.77032	CKB
meropenem-6141	1/100	0.561955	0.561955	0.724338	0.724338	1.33683	-0.77046	CKB
methacholine chloride-3452	1/100	0.561955	0.561955	0.724338	0.724338	1.336996	-0.77056	LGALS3BP
mexiletine-3781	1/100	0.561955	0.561955	0.724338	0.724338	1.337769	-0.771	TCN1
mebendazole-4694	1/100	0.561955	0.561955	0.724338	0.724338	1.338708	-0.77154	BMP4
mesalazine-5888	1/100	0.561955	0.561955	0.724338	0.724338	1.339106	-0.77177	LY6E
metoclopramide-2315	1/100	0.561955	0.561955	0.724338	0.724338	1.339111	-0.77177	C3
methoxamine-2488	1/100	0.561955	0.561955	0.724338	0.724338	1.339867	-0.77221	IFI6
mefexamide-1438	1/100	0.561955	0.561955	0.724338	0.724338	1.340753	-0.77272	RPS14P3
metrifonate-5989	1/100	0.561955	0.561955	0.724338	0.724338	1.341603	-0.77321	S100A4
metaraminol-3669	1/100	0.561955	0.561955	0.724338	0.724338	1.342043	-0.77346	CTGF
minoxidil-4216	1/100	0.561955	0.561955	0.724338	0.724338	1.342108	-0.7735	IFI6
metformin-1816	1/100	0.561955	0.561955	0.724338	0.724338	1.343702	-0.77442	TCN1
methylbenzethonium chloride-3768	1/100	0.561955	0.561955	0.724338	0.724338	1.345803	-0.77563	PSMB9
metformin-1858	1/100	0.561955	0.561955	0.724338	0.724338	1.34694	-0.77629	C3
methyldopate-7360	1/100	0.561955	0.561955	0.724338	0.724338	1.347385	-0.77654	IFI6
metergoline-3221	1/100	0.561955	0.561955	0.724338	0.724338	1.348108	-0.77696	RCN1
mebendazole-2338	1/100	0.561955	0.561955	0.724338	0.724338	1.348175	-0.777	PLCB4
methotrexate-6654	1/100	0.561955	0.561955	0.724338	0.724338	1.348256	-0.77705	TGFBI
methylbenzethonium chloride-3850	1/100	0.561955	0.561955	0.724338	0.724338	1.349483	-0.77775	IFI6
methotrexate-1599	1/100	0.561955	0.561955	0.724338	0.724338	1.349929	-0.77801	CKS2
methoxamine-6627	1/100	0.561955	0.561955	0.724338	0.724338	1.3532	-0.77989	PSMB9
meticrane-1792	1/100	0.561955	0.561955	0.724338	0.724338	1.353371	-0.77999	S100A4
methoxsalen-5007	1/100	0.561955	0.561955	0.724338	0.724338	1.355067	-0.78097	GPX2
metformin-1	1/100	0.561955	0.561955	0.724338	0.724338	1.356549	-0.78182	GPX2
methanthelinium bromide-3560	1/100	0.561955	0.561955	0.724338	0.724338	1.358513	-0.78296	SECTM1
metyrapone-4606	1/100	0.561955	0.561955	0.724338	0.724338	1.358989	-0.78323	OLFM4
mimosine-5302	1/100	0.561955	0.561955	0.724338	0.724338	1.359054	-0.78327	IFI6
metoprolol-4508	1/100	0.561955	0.561955	0.724338	0.724338	1.360009	-0.78382	PSMB9

metanephrine-2015	1/100	0.561955	0.561955	0.724338	0.724338	1.360495	-0.7841	SLPI
meteneprost-7504	1/100	0.561955	0.561955	0.724338	0.724338	1.360513	-0.78411	IFI6
mepyramine-3184	1/100	0.561955	0.561955	0.724338	0.724338	1.361104	-0.78445	LYZ
metanephrine-1933	1/100	0.561955	0.561955	0.724338	0.724338	1.367843	-0.78833	CTGF
methotrexate-6318	1/100	0.561955	0.561955	0.724338	0.724338	1.367933	-0.78839	TXNIP
mevalolactone-2718	1/100	0.561955	0.561955	0.724338	0.724338	1.371841	-0.79064	SPP1
metrifonate-1675	1/100	0.561955	0.561955	0.724338	0.724338	1.372365	-0.79094	LGALS3BP
metolazone-1514	1/100	0.561955	0.561955	0.724338	0.724338	1.372486	-0.79101	S100A4
metoclopramide-2353	1/100	0.561955	0.561955	0.724338	0.724338	1.373966	-0.79186	SPP1
methyldopate-6640	1/100	0.561955	0.561955	0.724338	0.724338	1.375272	-0.79262	PDIA3
metoclopramide-4750	1/100	0.561955	0.561955	0.724338	0.724338	1.375719	-0.79287	RCN1
mevalolactone-3459	1/100	0.561955	0.561955	0.724338	0.724338	1.376945	-0.79358	TMPRSS3
methylergometrine-3222	1/100	0.561955	0.561955	0.724338	0.724338	1.377501	-0.7939	LYZ
methylprednisolone-6785	1/100	0.561955	0.561955	0.724338	0.724338	1.381788	-0.79637	ISG15
minaprine-4230	1/100	0.561955	0.561955	0.724338	0.724338	1.382262	-0.79664	IFI6
minocycline-1135	1/100	0.561955	0.561955	0.724338	0.724338	1.387257	-0.79952	IGFBP2
methotrexate-5000	1/100	0.561955	0.561955	0.724338	0.724338	1.387478	-0.79965	TXNIP
methyldopa-1619	1/100	0.561955	0.561955	0.724338	0.724338	1.38767	-0.79976	SLC12A2
metrizamide-4156	1/100	0.561955	0.561955	0.724338	0.724338	1.388325	-0.80014	AP1S1
metoclopramide-3625	1/100	0.561955	0.561955	0.724338	0.724338	1.390005	-0.80111	SLPI
molindone-4199	1/100	0.561955	0.561955	0.724338	0.724338	1.393186	-0.80294	LY6E
midodrine-2087	1/100	0.561955	0.561955	0.724338	0.724338	1.393453	-0.80309	AP1S1
midodrine-7156	1/100	0.561955	0.561955	0.724338	0.724338	1.394344	-0.80361	MMP1
midodrine-2250	1/100	0.561955	0.561955	0.724338	0.724338	1.394647	-0.80378	AP1S1
monorden-544	1/100	0.561955	0.561955	0.724338	0.724338	1.394649	-0.80378	CTSE
methylergometrine-1607	1/100	0.561955	0.561955	0.724338	0.724338	1.394997	-0.80398	HSPB1
minoxidil-1996	1/100	0.561955	0.561955	0.724338	0.724338	1.395191	-0.8041	C3
methocarbamol-2111	1/100	0.561955	0.561955	0.724338	0.724338	1.39589	-0.8045	TCN1
molsidomine-5426	1/100	0.561955	0.561955	0.724338	0.724338	1.395962	-0.80454	IFI6
metixene-2451	1/100	0.561955	0.561955	0.724338	0.724338	1.396477	-0.80484	GPX2
meticrane-7282	1/100	0.561955	0.561955	0.724338	0.724338	1.396575	-0.80489	IFI6
molindone-2917	1/100	0.561955	0.561955	0.724338	0.724338	1.39695	-0.80511	C3
molindone-7337	1/100	0.561955	0.561955	0.724338	0.724338	1.398603	-0.80606	TSPAN1
minaprine-1968	1/100	0.561955	0.561955	0.724338	0.724338	1.39917	-0.80639	CTSD
mitoxantrone-3232	1/100	0.561955	0.561955	0.724338	0.724338	1.400283	-0.80703	IER2
metrifonate-1839	1/100	0.561955	0.561955	0.724338	0.724338	1.403231	-0.80873	C3
metrifonate-1797	1/100	0.561955	0.561955	0.724338	0.724338	1.40407	-0.80921	IL32

miconazole-1896	1/100	0.561955	0.561955	0.724338	0.724338	1.40618	-0.81043	IFI6
methyldopa-4677	1/100	0.561955	0.561955	0.724338	0.724338	1.407505	-0.81119	OLFM4
midecamycin-1526	1/100	0.561955	0.561955	0.724338	0.724338	1.40805	-0.81151	TFF2
metrizamide-3255	1/100	0.561955	0.561955	0.724338	0.724338	1.409093	-0.81211	PGM1
monensin-3443	1/100	0.561955	0.561955	0.724338	0.724338	1.409227	-0.81218	S100A4
N-acetyl-L-aspartic acid-4007	1/100	0.561955	0.561955	0.724338	0.724338	1.410237	-0.81277	MMP3
monocrotaline-2749	1/100	0.561955	0.561955	0.724338	0.724338	1.410873	-0.81313	TSPAN1
milrinone-7210	1/100	0.561955	0.561955	0.724338	0.724338	1.413114	-0.81443	LGALS3BP
molindone-4784	1/100	0.561955	0.561955	0.724338	0.724338	1.413134	-0.81444	S100A6
monorden-1219	1/100	0.561955	0.561955	0.724338	0.724338	1.414992	-0.81551	CCL20
moroxydine-1944	1/100	0.561955	0.561955	0.724338	0.724338	1.417353	-0.81687	ID1
monorden-1160	1/100	0.561955	0.561955	0.724338	0.724338	1.41758	-0.817	IFI6
metitepine-6312	1/100	0.561955	0.561955	0.724338	0.724338	1.418672	-0.81763	CDH3
metixene-5018	1/100	0.561955	0.561955	0.724338	0.724338	1.41896	-0.81779	IFI27
monastrol-311	1/100	0.561955	0.561955	0.724338	0.724338	1.419638	-0.81818	SPP1
nadide-6091	1/100	0.561955	0.561955	0.724338	0.724338	1.419782	-0.81827	TFF2
metoprolol-2543	1/100	0.561955	0.561955	0.724338	0.724338	1.421036	-0.81899	MEST
MS-275-7074	1/100	0.561955	0.561955	0.724338	0.724338	1.422085	-0.8196	AP1S1
N6-methyladenosine-2626	1/100	0.561955	0.561955	0.724338	0.724338	1.424653	-0.82108	S100A4
N-acetylmuramic acid-4406	1/100	0.561955	0.561955	0.724338	0.724338	1.424957	-0.82125	AP1S1
monorden-1644	1/100	0.561955	0.561955	0.724338	0.724338	1.425117	-0.82134	IER2
metyrapone-3070	1/100	0.561955	0.561955	0.724338	0.724338	1.426792	-0.82231	IFI6
metyrapone-5667	1/100	0.561955	0.561955	0.724338	0.724338	1.426884	-0.82236	BMP4
myosmine-4293	1/100	0.561955	0.561955	0.724338	0.724338	1.427355	-0.82263	MMP3
nadolol-3359	1/100	0.561955	0.561955	0.724338	0.724338	1.427975	-0.82299	QPCT
monorden-484	1/100	0.561955	0.561955	0.724338	0.724338	1.430879	-0.82466	ID3
naftifine-7273	1/100	0.561955	0.561955	0.724338	0.724338	1.432762	-0.82575	MMP1
miconazole-1977	1/100	0.561955	0.561955	0.724338	0.724338	1.433362	-0.82609	C3
morantel-7010	1/100	0.561955	0.561955	0.724338	0.724338	1.433532	-0.82619	LGALS3BP
metronidazole-2003	1/100	0.561955	0.561955	0.724338	0.724338	1.433738	-0.82631	IFITM3
minoxidil-4800	1/100	0.561955	0.561955	0.724338	0.724338	1.435206	-0.82716	IFI6
moxisylyte-7015	1/100	0.561955	0.561955	0.724338	0.724338	1.435751	-0.82747	LGALS3BP
monorden-6938	1/100	0.561955	0.561955	0.724338	0.724338	1.436991	-0.82819	ID1
monorden-489	1/100	0.561955	0.561955	0.724338	0.724338	1.439587	-0.82968	CTSE
niclosamide-4136	1/100	0.561955	0.561955	0.724338	0.724338	1.439592	-0.82969	BMP4
monensin-1105	1/100	0.561955	0.561955	0.724338	0.724338	1.443285	-0.83181	TXNIP
myricetin-1334	1/100	0.561955	0.561955	0.724338	0.724338	1.444371	-0.83244	SLPI

moxisylyte-1682	1/100	0.561955	0.561955	0.724338	0.724338	1.444422	-0.83247	S100A4
monorden-836	1/100	0.561955	0.561955	0.724338	0.724338	1.444697	-0.83263	ID1
moxonidine-4084	1/100	0.561955	0.561955	0.724338	0.724338	1.446994	-0.83395	LUM
nifenazone-2285	1/100	0.561955	0.561955	0.724338	0.724338	1.450037	-0.8357	C3
nadide-7227	1/100	0.561955	0.561955	0.724338	0.724338	1.451266	-0.83641	TSPAN1
monorden-449	1/100	0.561955	0.561955	0.724338	0.724338	1.452236	-0.83697	MMP3
monorden-6979	1/100	0.561955	0.561955	0.724338	0.724338	1.452651	-0.83721	ID1
myosmine-3737	1/100	0.561955	0.561955	0.724338	0.724338	1.453546	-0.83773	C3
nicardipine-6297	1/100	0.561955	0.561955	0.724338	0.724338	1.453819	-0.83788	TSPAN8
napelline-4486	1/100	0.561955	0.561955	0.724338	0.724338	1.454764	-0.83843	IFI6
mianserin-1385	1/100	0.561955	0.561955	0.724338	0.724338	1.455937	-0.83911	SLC12A2
monensin-3704	1/100	0.561955	0.561955	0.724338	0.724338	1.456859	-0.83964	BMP4
naproxen-6096	1/100	0.561955	0.561955	0.724338	0.724338	1.457262	-0.83987	SLPI
moxonidine-7343	1/100	0.561955	0.561955	0.724338	0.724338	1.457877	-0.84022	ISG15
nafcillin-2983	1/100	0.561955	0.561955	0.724338	0.724338	1.459216	-0.84099	ANXA3
nalbuphine-7177	1/100	0.561955	0.561955	0.724338	0.724338	1.460313	-0.84163	IGFBP2
mimosine-2638	1/100	0.561955	0.561955	0.724338	0.724338	1.461417	-0.84226	BMP4
monobenzone-5312	1/100	0.561955	0.561955	0.724338	0.724338	1.461825	-0.8425	CKS2
nalidixic acid-2336	1/100	0.561955	0.561955	0.724338	0.724338	1.461995	-0.8426	TSPAN13
naringenin-3278	1/100	0.561955	0.561955	0.724338	0.724338	1.462992	-0.84317	ID3
naphazoline-1466	1/100	0.561955	0.561955	0.724338	0.724338	1.467589	-0.84582	TSPAN1
netilmicin-2963	1/100	0.561955	0.561955	0.724338	0.724338	1.467967	-0.84604	C3
naftifine-3536	1/100	0.561955	0.561955	0.724338	0.724338	1.468771	-0.8465	SECTM1
nifurtimox-2908	1/100	0.561955	0.561955	0.724338	0.724338	1.468966	-0.84661	IFI6
naringenin-1342	1/100	0.561955	0.561955	0.724338	0.724338	1.470364	-0.84742	SLPI
monastrol-614	1/100	0.561955	0.561955	0.724338	0.724338	1.471045	-0.84781	NOP10
nabumetone-5428	1/100	0.561955	0.561955	0.724338	0.724338	1.471968	-0.84834	IFI6
naftopidil-4193	1/100	0.561955	0.561955	0.724338	0.724338	1.472414	-0.8486	IGFBP2
N-acetylmuramic acid-1326	1/100	0.561955	0.561955	0.724338	0.724338	1.472609	-0.84871	CTSD
N-acetyl-L-aspartic acid-1329	1/100	0.561955	0.561955	0.724338	0.724338	1.472857	-0.84886	CTSD
moxisylyte-1804	1/100	0.561955	0.561955	0.724338	0.724338	1.473601	-0.84929	IFI6
neomycin-1383	1/100	0.561955	0.561955	0.724338	0.724338	1.473982	-0.8495	PIGR
neomycin-2229	1/100	0.561955	0.561955	0.724338	0.724338	1.474819	-0.84999	S100A4
mycophenolic acid-2857	1/100	0.561955	0.561955	0.724338	0.724338	1.476134	-0.85075	BMP4
moroxydine-6705	1/100	0.561955	0.561955	0.724338	0.724338	1.477225	-0.85137	IFI6
monastrol-627	1/100	0.561955	0.561955	0.724338	0.724338	1.484182	-0.85538	TSPAN1
nifuroxazide-4253	1/100	0.561955	0.561955	0.724338	0.724338	1.485446	-0.85611	TSPAN8

niflumic acid-5071	1/100	0.561955	0.561955	0.724338	0.724338	1.486086	-0.85648	GPX2
nortriptyline-6003	1/100	0.561955	0.561955	0.724338	0.724338	1.486749	-0.85686	IFI6
nicotinic acid-5301	1/100	0.561955	0.561955	0.724338	0.724338	1.48687	-0.85693	ETS2
myricetin-4090	1/100	0.561955	0.561955	0.724338	0.724338	1.486924	-0.85696	S100A4
natamycin-3548	1/100	0.561955	0.561955	0.724338	0.724338	1.486968	-0.85699	TFF3
naproxen-6358	1/100	0.561955	0.561955	0.724338	0.724338	1.487713	-0.85742	IGFBP2
morantel-1676	1/100	0.561955	0.561955	0.724338	0.724338	1.487902	-0.85753	TMPRSS3
nabumetone-6487	1/100	0.561955	0.561955	0.724338	0.724338	1.488963	-0.85814	GPX2
moxisylyte-1846	1/100	0.561955	0.561955	0.724338	0.724338	1.491492	-0.8596	C3
nialamide-4347	1/100	0.561955	0.561955	0.724338	0.724338	1.492394	-0.86012	ITM2C
naproxen-1869	1/100	0.561955	0.561955	0.724338	0.724338	1.492491	-0.86017	ANXA3
natamycin-5809	1/100	0.561955	0.561955	0.724338	0.724338	1.492661	-0.86027	CTSD
nifuroxazide-2850	1/100	0.561955	0.561955	0.724338	0.724338	1.492931	-0.86043	BMP4
nifuroxazide-4835	1/100	0.561955	0.561955	0.724338	0.724338	1.494668	-0.86143	RNF43
nefopam-2317	1/100	0.561955	0.561955	0.724338	0.724338	1.494964	-0.8616	C3
naloxone-1506	1/100	0.561955	0.561955	0.724338	0.724338	1.496876	-0.8627	TMPRSS3
ondansetron-5796	1/100	0.561955	0.561955	0.724338	0.724338	1.497619	-0.86313	MMP3
mometasone-5116	1/100	0.561955	0.561955	0.724338	0.724338	1.498095	-0.8634	IFI6
neomycin-2066	1/100	0.561955	0.561955	0.724338	0.724338	1.498595	-0.86369	S100A4
naproxen-2533	1/100	0.561955	0.561955	0.724338	0.724338	1.499342	-0.86412	ANXA3
naproxen-1828	1/100	0.561955	0.561955	0.724338	0.724338	1.500319	-0.86468	S100A4
nifenazone-1439	1/100	0.561955	0.561955	0.724338	0.724338	1.50052	-0.8648	LY6E
ofloxacin-2302	1/100	0.561955	0.561955	0.724338	0.724338	1.50072	-0.86492	BMP4
nordihydroguaiaretic acid-1003	1/100	0.561955	0.561955	0.724338	0.724338	1.502241	-0.86579	IFNGR2
nadide-2529	1/100	0.561955	0.561955	0.724338	0.724338	1.502285	-0.86582	GPX2
niclosamide-1498	1/100	0.561955	0.561955	0.724338	0.724338	1.502534	-0.86596	BMP4
nitrendipine-6304	1/100	0.561955	0.561955	0.724338	0.724338	1.503416	-0.86647	CDH3
natamycin-6126	1/100	0.561955	0.561955	0.724338	0.724338	1.504579	-0.86714	ANXA3
nifedipine-1814	1/100	0.561955	0.561955	0.724338	0.724338	1.504848	-0.86729	IFITM1
nocodazole-2239	1/100	0.561955	0.561955	0.724338	0.724338	1.505406	-0.86762	BMP4
nalbuphine-2225	1/100	0.561955	0.561955	0.724338	0.724338	1.506565	-0.86828	AP1S1
neostigmine bromide-3294	1/100	0.561955	0.561955	0.724338	0.724338	1.50709	-0.86859	S100A6
nefopam-3730	1/100	0.561955	0.561955	0.724338	0.724338	1.508355	-0.86932	C3
nordihydroguaiaretic acid-6983	1/100	0.561955	0.561955	0.724338	0.724338	1.5094	-0.86992	PGM1
nimodipine-3103	1/100	0.561955	0.561955	0.724338	0.724338	1.509833	-0.87017	IFITM3
netilmicin-3524	1/100	0.561955	0.561955	0.724338	0.724338	1.510712	-0.87067	S100A4
nystatin-4223	1/100	0.561955	0.561955	0.724338	0.724338	1.51353	-0.8723	GPX2

nitrofurantoin-4697	1/100	0.561955	0.561955	0.724338	0.724338	1.513839	-0.87248	QPCT
nifuroxazide-2490	1/100	0.561955	0.561955	0.724338	0.724338	1.515381	-0.87336	FOS
nordihydroguaiaretic acid-5220	1/100	0.561955	0.561955	0.724338	0.724338	1.517471	-0.87457	QPCT
norfloxacin-2090	1/100	0.561955	0.561955	0.724338	0.724338	1.517869	-0.8748	CCL20
orphenadrine-4359	1/100	0.561955	0.561955	0.724338	0.724338	1.520058	-0.87606	ITM2C
nalbuphine-5820	1/100	0.561955	0.561955	0.724338	0.724338	1.520528	-0.87633	ITM2C
oxolinic acid-5094	1/100	0.561955	0.561955	0.724338	0.724338	1.523054	-0.87779	IFI6
noscapine-1753	1/100	0.561955	0.561955	0.724338	0.724338	1.523679	-0.87815	CKB
naphazoline-1966	1/100	0.561955	0.561955	0.724338	0.724338	1.526259	-0.87963	SLPI
oxantel-5338	1/100	0.561955	0.561955	0.724338	0.724338	1.526372	-0.8797	RPS14P3
orciprenaline-4831	1/100	0.561955	0.561955	0.724338	0.724338	1.526714	-0.8799	TSPAN1
nordihydroguaiaretic acid-2683	1/100	0.561955	0.561955	0.724338	0.724338	1.527288	-0.88023	SLPI
oleandomycin-1518	1/100	0.561955	0.561955	0.724338	0.724338	1.527308	-0.88024	TFF2
orphenadrine-2356	1/100	0.561955	0.561955	0.724338	0.724338	1.529695	-0.88161	ITGAV
natamycin-7167	1/100	0.561955	0.561955	0.724338	0.724338	1.530548	-0.88211	ID3
nomifensine-2224	1/100	0.561955	0.561955	0.724338	0.724338	1.532005	-0.88295	PGM1
nisoxetine-6496	1/100	0.561955	0.561955	0.724338	0.724338	1.53428	-0.88426	SECTM1
nitrofurantoin-2341	1/100	0.561955	0.561955	0.724338	0.724338	1.535317	-0.88485	C3
noretynodrel-1818	1/100	0.561955	0.561955	0.724338	0.724338	1.535567	-0.885	IL32
niclosamide-4018	1/100	0.561955	0.561955	0.724338	0.724338	1.536761	-0.88569	SOX9
octopamine-6491	1/100	0.561955	0.561955	0.724338	0.724338	1.539421	-0.88722	QPCT
nilutamide-3104	1/100	0.561955	0.561955	0.724338	0.724338	1.542013	-0.88871	IGFBP2
norethisterone-7414	1/100	0.561955	0.561955	0.724338	0.724338	1.542478	-0.88898	TSPAN1
noscapine-2745	1/100	0.561955	0.561955	0.724338	0.724338	1.543061	-0.88932	TMPRSS3
orciprenaline-2845	1/100	0.561955	0.561955	0.724338	0.724338	1.544104	-0.88992	BMP4
nilutamide-5362	1/100	0.561955	0.561955	0.724338	0.724338	1.544461	-0.89012	DEK
nicergoline-2058	1/100	0.561955	0.561955	0.724338	0.724338	1.544975	-0.89042	TCN1
nitrendipine-336	1/100	0.561955	0.561955	0.724338	0.724338	1.54527	-0.89059	TGFBI
NU-1025-608	1/100	0.561955	0.561955	0.724338	0.724338	1.54612	-0.89108	DPEP1
nisoxetine-5516	1/100	0.561955	0.561955	0.724338	0.724338	1.546463	-0.89128	TSPAN1
nitrendipine-3087	1/100	0.561955	0.561955	0.724338	0.724338	1.548684	-0.89256	LY6E
norcyclobenzaprine-4190	1/100	0.561955	0.561955	0.724338	0.724338	1.54888	-0.89267	ID1
orciprenaline-4248	1/100	0.561955	0.561955	0.724338	0.724338	1.551714	-0.89431	CTGF
nipecotic acid-5999	1/100	0.561955	0.561955	0.724338	0.724338	1.552127	-0.89454	ISG15
ornidazole-5064	1/100	0.561955	0.561955	0.724338	0.724338	1.553042	-0.89507	GPX2
nitrofurantoin-3674	1/100	0.561955	0.561955	0.724338	0.724338	1.553427	-0.89529	MMP3
nifedipine-1856	1/100	0.561955	0.561955	0.724338	0.724338	1.554658	-0.896	C3

orphenadrine-2318	1/100	0.561955	0.561955	0.724338	0.724338	1.556629	-0.89714	RCN1
oxaprozin-3876	1/100	0.561955	0.561955	0.724338	0.724338	1.556748	-0.89721	C3
novobiocin-632	1/100	0.561955	0.561955	0.724338	0.724338	1.55695	-0.89732	IGFBP2
norfloxacin-1406	1/100	0.561955	0.561955	0.724338	0.724338	1.558393	-0.89815	SLC12A2
nortriptyline-2391	1/100	0.561955	0.561955	0.724338	0.724338	1.559545	-0.89882	CKB
nifurtimox-7328	1/100	0.561955	0.561955	0.724338	0.724338	1.560041	-0.8991	IFI6
oxamic acid-439	1/100	0.561955	0.561955	0.724338	0.724338	1.561329	-0.89985	TSPAN1
noscapine-7204	1/100	0.561955	0.561955	0.724338	0.724338	1.563052	-0.90084	S100A4
novobiocin-2990	1/100	0.561955	0.561955	0.724338	0.724338	1.563511	-0.9011	CTSD
noretynodrel-1696	1/100	0.561955	0.561955	0.724338	0.724338	1.564	-0.90139	S100A4
oxedrine-6798	1/100	0.561955	0.561955	0.724338	0.724338	1.564621	-0.90174	IFI6
nordihydroguaiaretic acid-415	1/100	0.561955	0.561955	0.724338	0.724338	1.564786	-0.90184	RNF43
nimesulide-1428	1/100	0.561955	0.561955	0.724338	0.724338	1.565733	-0.90238	RPS14P3
oxamniquine-4124	1/100	0.561955	0.561955	0.724338	0.724338	1.572556	-0.90632	TMPRSS3
oxybenzone-3092	1/100	0.561955	0.561955	0.724338	0.724338	1.574099	-0.90721	IFI6
parbendazole-3799	1/100	0.561955	0.561955	0.724338	0.724338	1.575972	-0.90829	IFI6
NS-398-6911	1/100	0.561955	0.561955	0.724338	0.724338	1.576087	-0.90835	ID1
oxaprozin-4530	1/100	0.561955	0.561955	0.724338	0.724338	1.577023	-0.90889	C3
nomifensine-7217	1/100	0.561955	0.561955	0.724338	0.724338	1.580319	-0.91079	TSPAN1
niridazole-4621	1/100	0.561955	0.561955	0.724338	0.724338	1.581025	-0.9112	OLFM4
oxantel-2632	1/100	0.561955	0.561955	0.724338	0.724338	1.584677	-0.9133	TGFBI
oxprenolol-3568	1/100	0.561955	0.561955	0.724338	0.724338	1.586264	-0.91422	GPX2
oxybuprocaine-4115	1/100	0.561955	0.561955	0.724338	0.724338	1.586395	-0.91429	IFI6
pempidine-4307	1/100	0.561955	0.561955	0.724338	0.724338	1.586612	-0.91442	IFI6
oleandomycin-2018	1/100	0.561955	0.561955	0.724338	0.724338	1.587775	-0.91509	MEST
oleandomycin-5676	1/100	0.561955	0.561955	0.724338	0.724338	1.589297	-0.91596	IFNGR2
oxymetazoline-2114	1/100	0.561955	0.561955	0.724338	0.724338	1.591266	-0.9171	CCL20
paclitaxel-5320	1/100	0.561955	0.561955	0.724338	0.724338	1.591278	-0.91711	TGFBI
oxymetazoline-6350	1/100	0.561955	0.561955	0.724338	0.724338	1.594035	-0.9187	LCN2
orphenadrine-4537	1/100	0.561955	0.561955	0.724338	0.724338	1.594926	-0.91921	C3
ozagrel-7281	1/100	0.561955	0.561955	0.724338	0.724338	1.599915	-0.92208	IFI6
orlistat-6415	1/100	0.561955	0.561955	0.724338	0.724338	1.600647	-0.92251	QPCT
oxybenzone-6309	1/100	0.561955	0.561955	0.724338	0.724338	1.601374	-0.92293	CTSD
papaverine-5769	1/100	0.561955	0.561955	0.724338	0.724338	1.602883	-0.9238	MMP3
pentetic acid-5264	1/100	0.561955	0.561955	0.724338	0.724338	1.602901	-0.92381	SECTM1
oxaprozin-3794	1/100	0.561955	0.561955	0.724338	0.724338	1.603868	-0.92436	RNF43
oxetacaine-1484	1/100	0.561955	0.561955	0.724338	0.724338	1.604195	-0.92455	BMP4

oxaprozin-863	1/100	0.561955	0.561955	0.724338	0.724338	1.604782	-0.92489	SOX9
oxolinic acid-1419	1/100	0.561955	0.561955	0.724338	0.724338	1.607203	-0.92629	IFITM3
oxytetracycline-1553	1/100	0.561955	0.561955	0.724338	0.724338	1.608105	-0.9268	IFITM3
oxamniquine-2924	1/100	0.561955	0.561955	0.724338	0.724338	1.608113	-0.92681	C3
paracetamol-3364	1/100	0.561955	0.561955	0.724338	0.724338	1.611736	-0.9289	C3
pancuronium bromide-4570	1/100	0.561955	0.561955	0.724338	0.724338	1.611937	-0.92901	IFI6
oxytetracycline-3170	1/100	0.561955	0.561955	0.724338	0.724338	1.613566	-0.92995	LYZ
pentetrazol-5508	1/100	0.561955	0.561955	0.724338	0.724338	1.613829	-0.9301	GPX2
pentetrazol-2255	1/100	0.561955	0.561955	0.724338	0.724338	1.616383	-0.93158	SLPI
orlistat-6420	1/100	0.561955	0.561955	0.724338	0.724338	1.616896	-0.93187	C3
oxolinic acid-5519	1/100	0.561955	0.561955	0.724338	0.724338	1.617687	-0.93233	TSPAN1
oxybenzone-5410	1/100	0.561955	0.561955	0.724338	0.724338	1.618112	-0.93257	QPCT
oxaprozin-971	1/100	0.561955	0.561955	0.724338	0.724338	1.618875	-0.93301	SOX9
pargyline-2265	1/100	0.561955	0.561955	0.724338	0.724338	1.621296	-0.93441	AP1S1
pergolide-7434	1/100	0.561955	0.561955	0.724338	0.724338	1.623909	-0.93591	TSPAN1
palmatine-2138	1/100	0.561955	0.561955	0.724338	0.724338	1.624723	-0.93638	RPS14P3
pargyline-2102	1/100	0.561955	0.561955	0.724338	0.724338	1.625663	-0.93692	TCN1
ozagrel-5983	1/100	0.561955	0.561955	0.724338	0.724338	1.626665	-0.9375	IFI6
pempidine-3832	1/100	0.561955	0.561955	0.724338	0.724338	1.627102	-0.93775	TFF2
phenoxybenzamine-6451	1/100	0.561955	0.561955	0.724338	0.724338	1.628139	-0.93835	TXNIP
pentamidine-2473	1/100	0.561955	0.561955	0.724338	0.724338	1.628791	-0.93873	IGFBP2
pentetic acid-3049	1/100	0.561955	0.561955	0.724338	0.724338	1.629555	-0.93917	GPX2
phentolamine-2362	1/100	0.561955	0.561955	0.724338	0.724338	1.629926	-0.93938	CTSD
pheniramine-4012	1/100	0.561955	0.561955	0.724338	0.724338	1.630055	-0.93946	MMP3
oxybuprocaine-3996	1/100	0.561955	0.561955	0.724338	0.724338	1.630085	-0.93947	GPX2
oxetacaine-1984	1/100	0.561955	0.561955	0.724338	0.724338	1.630431	-0.93967	MEST
parbendazole-4357	1/100	0.561955	0.561955	0.724338	0.724338	1.630793	-0.93988	QPCT
penbutolol-3534	1/100	0.561955	0.561955	0.724338	0.724338	1.63093	-0.93996	PDIA3
PF-00562151-00-6863	1/100	0.561955	0.561955	0.724338	0.724338	1.633595	-0.9415	SPINK1
oxyphenbutazone-4506	1/100	0.561955	0.561955	0.724338	0.724338	1.635824	-0.94278	C3
pentoxifylline-6021	1/100	0.561955	0.561955	0.724338	0.724338	1.638176	-0.94414	IFI6
PF-00562151-00-5922	1/100	0.561955	0.561955	0.724338	0.724338	1.638615	-0.94439	IL32
parthenolide-2885	1/100	0.561955	0.561955	0.724338	0.724338	1.639344	-0.94481	TXNIP
oxybutynin-7126	1/100	0.561955	0.561955	0.724338	0.724338	1.639367	-0.94482	IGFBP2
penbutolol-7476	1/100	0.561955	0.561955	0.724338	0.724338	1.641193	-0.94587	GPX2
pinacidil-5456	1/100	0.561955	0.561955	0.724338	0.724338	1.642249	-0.94648	IFI6
PF-00562151-00-5917	1/100	0.561955	0.561955	0.724338	0.724338	1.648642	-0.95017	TFF2

PHA-00846566E-7046	1/100	0.561955	0.561955	0.724338	0.724338	1.649718	-0.95079	RNF43
oxedrine-6156	1/100	0.561955	0.561955	0.724338	0.724338	1.650706	-0.95136	TSPAN13
paroxetine-4556	1/100	0.561955	0.561955	0.724338	0.724338	1.650852	-0.95144	TGFBI
paromomycin-4595	1/100	0.561955	0.561955	0.724338	0.724338	1.651117	-0.95159	C3
perhexiline-2410	1/100	0.561955	0.561955	0.724338	0.724338	1.651473	-0.9518	C3
PHA-00767505E-6550	1/100	0.561955	0.561955	0.724338	0.724338	1.653726	-0.9531	PGM1
paclitaxel-2043	1/100	0.561955	0.561955	0.724338	0.724338	1.654824	-0.95373	NOP10
phenelzine-2357	1/100	0.561955	0.561955	0.724338	0.724338	1.65552	-0.95413	C3
phenindione-5991	1/100	0.561955	0.561955	0.724338	0.724338	1.658165	-0.95566	IFITM1
palmatine-2795	1/100	0.561955	0.561955	0.724338	0.724338	1.659732	-0.95656	SLPI
pheniramine-4130	1/100	0.561955	0.561955	0.724338	0.724338	1.663915	-0.95897	TCN1
pergolide-7031	1/100	0.561955	0.561955	0.724338	0.724338	1.664386	-0.95924	BMP4
pentoxifylline-7319	1/100	0.561955	0.561955	0.724338	0.724338	1.665075	-0.95964	IGFBP2
phenformin-3622	1/100	0.561955	0.561955	0.724338	0.724338	1.666381	-0.96039	SLPI
oxyphenbutazone-3582	1/100	0.561955	0.561955	0.724338	0.724338	1.668282	-0.96149	BMP4
pirenzepine-5872	1/100	0.561955	0.561955	0.724338	0.724338	1.669143	-0.96198	TFF2
PF-00875133-00-5928	1/100	0.561955	0.561955	0.724338	0.724338	1.66943	-0.96215	GPX2
pepstatin-1328	1/100	0.561955	0.561955	0.724338	0.724338	1.669645	-0.96227	QPCT
phentolamine-3779	1/100	0.561955	0.561955	0.724338	0.724338	1.672176	-0.96373	IFI6
pentoxifylline-2127	1/100	0.561955	0.561955	0.724338	0.724338	1.674531	-0.96509	TCN1
piperacillin-3939	1/100	0.561955	0.561955	0.724338	0.724338	1.675324	-0.96555	RCN1
pipemidic acid-6752	1/100	0.561955	0.561955	0.724338	0.724338	1.675503	-0.96565	GPX2
PHA-00851261E-4333	1/100	0.561955	0.561955	0.724338	0.724338	1.67612	-0.966	IGFBP2
PHA-00767505E-6596	1/100	0.561955	0.561955	0.724338	0.724338	1.676298	-0.96611	PLP2
piperacillin-4320	1/100	0.561955	0.561955	0.724338	0.724338	1.678158	-0.96718	IFI6
PF-01378883-00-6405	1/100	0.561955	0.561955	0.724338	0.724338	1.6786	-0.96743	QPCT
pimozide-6780	1/100	0.561955	0.561955	0.724338	0.724338	1.678981	-0.96765	SECTM1
phenanthridinone-1115	1/100	0.561955	0.561955	0.724338	0.724338	1.679305	-0.96784	ID1
phenelzine-4360	1/100	0.561955	0.561955	0.724338	0.724338	1.679598	-0.96801	CTSD
phenazone-1489	1/100	0.561955	0.561955	0.724338	0.724338	1.681725	-0.96923	BMP4
phentolamine-3860	1/100	0.561955	0.561955	0.724338	0.724338	1.682864	-0.96989	SECTM1
phenindione-1718	1/100	0.561955	0.561955	0.724338	0.724338	1.683713	-0.97038	MEST
pepstatin-3264	1/100	0.561955	0.561955	0.724338	0.724338	1.684068	-0.97058	PDIA3
PF-00562151-00-5954	1/100	0.561955	0.561955	0.724338	0.724338	1.685332	-0.97131	IFI6
pipenzolate bromide-2719	1/100	0.561955	0.561955	0.724338	0.724338	1.68598	-0.97169	PGM1
pindolol-1392	1/100	0.561955	0.561955	0.724338	0.724338	1.686099	-0.97176	S100A10
PHA-00745360-4384	1/100	0.561955	0.561955	0.724338	0.724338	1.686915	-0.97223	C3

phensuximide-2960	1/100	0.561955	0.561955	0.724338	0.724338	1.68752	-0.97257	C3
pinacidil-6356	1/100	0.561955	0.561955	0.724338	0.724338	1.687735	-0.9727	CDH3
pirenperone-5639	1/100	0.561955	0.561955	0.724338	0.724338	1.690349	-0.9742	TFF2
phenylpropanolamine-1602	1/100	0.561955	0.561955	0.724338	0.724338	1.69127	-0.97474	IFI6
pirinixic acid-481	1/100	0.561955	0.561955	0.724338	0.724338	1.692562	-0.97548	CTGF
phenazopyridine-6100	1/100	0.561955	0.561955	0.724338	0.724338	1.693076	-0.97578	MMP3
PHA-00767505E-6591	1/100	0.561955	0.561955	0.724338	0.724338	1.694329	-0.9765	BMP4
perhexiline-7441	1/100	0.561955	0.561955	0.724338	0.724338	1.694379	-0.97653	LGALS3BP
phenoxybenzamine-5613	1/100	0.561955	0.561955	0.724338	0.724338	1.695405	-0.97712	TMPRSS3
piperacillin-3763	1/100	0.561955	0.561955	0.724338	0.724338	1.695416	-0.97713	BMP4
pivmecillinam-6014	1/100	0.561955	0.561955	0.724338	0.724338	1.696987	-0.97803	RNF43
pheniramine-1992	1/100	0.561955	0.561955	0.724338	0.724338	1.69788	-0.97855	C3
pizotifen-6513	1/100	0.561955	0.561955	0.724338	0.724338	1.698586	-0.97895	TFF2
PHA-00745360-4381	1/100	0.561955	0.561955	0.724338	0.724338	1.698858	-0.97911	TFF2
PHA-00816795-7072	1/100	0.561955	0.561955	0.724338	0.724338	1.698922	-0.97915	TSPAN1
phensuximide-3521	1/100	0.561955	0.561955	0.724338	0.724338	1.699402	-0.97942	PDIA3
picotamide-1387	1/100	0.561955	0.561955	0.724338	0.724338	1.699754	-0.97963	CTSH
phensuximide-5522	1/100	0.561955	0.561955	0.724338	0.724338	1.699866	-0.97969	ISG15
pirinixic acid-495	1/100	0.561955	0.561955	0.724338	0.724338	1.70006	-0.9798	SLPI
pirenzepine-1388	1/100	0.561955	0.561955	0.724338	0.724338	1.702529	-0.98122	IFITM3
physostigmine-2768	1/100	0.561955	0.561955	0.724338	0.724338	1.702556	-0.98124	GPX2
phthalylsulfathiazole-4653	1/100	0.561955	0.561955	0.724338	0.724338	1.702588	-0.98126	IGFBP2
phenacetin-2471	1/100	0.561955	0.561955	0.724338	0.724338	1.705933	-0.98319	TSPAN13
piroxicam-2089	1/100	0.561955	0.561955	0.724338	0.724338	1.707663	-0.98418	TCN1
picrotoxinin-4260	1/100	0.561955	0.561955	0.724338	0.724338	1.708038	-0.9844	TSPAN8
pimethixene-7468	1/100	0.561955	0.561955	0.724338	0.724338	1.708393	-0.9846	PGM1
pindolol-4496	1/100	0.561955	0.561955	0.724338	0.724338	1.711019	-0.98612	CTSD
phentolamine-2323	1/100	0.561955	0.561955	0.724338	0.724338	1.711394	-0.98633	C3
pheneticillin-2542	1/100	0.561955	0.561955	0.724338	0.724338	1.71334	-0.98746	ANXA3
PF-00562151-00-6907	1/100	0.561955	0.561955	0.724338	0.724338	1.714808	-0.9883	GPX2
pregnenolone-2856	1/100	0.561955	0.561955	0.724338	0.724338	1.719092	-0.99077	LGALS3BP
picotamide-2070	1/100	0.561955	0.561955	0.724338	0.724338	1.719399	-0.99095	CTSD
picotamide-6787	1/100	0.561955	0.561955	0.724338	0.724338	1.721019	-0.99188	IFI6
pirenperone-2455	1/100	0.561955	0.561955	0.724338	0.724338	1.721581	-0.9922	TCN1
pirlindole-3140	1/100	0.561955	0.561955	0.724338	0.724338	1.72486	-0.99409	ISG15
prenylamine-5489	1/100	0.561955	0.561955	0.724338	0.724338	1.727728	-0.99575	RNF43
piromidic acid-2996	1/100	0.561955	0.561955	0.724338	0.724338	1.729869	-0.99698	CTSD

piroxicam-7445	1/100	0.561955	0.561955	0.724338	0.724338	1.730156	-0.99715	TMPRSS3
phenylpropanolamine-3217	1/100	0.561955	0.561955	0.724338	0.724338	1.730667	-0.99744	BMP4
pregnenolone-4802	1/100	0.561955	0.561955	0.724338	0.724338	1.730897	-0.99757	SECTM1
piracetam-1710	1/100	0.561955	0.561955	0.724338	0.724338	1.731175	-0.99773	CKB
piribedil-5434	1/100	0.561955	0.561955	0.724338	0.724338	1.733269	-0.99894	IFI6
PNU-0230031-3632	1/100	0.561955	0.561955	0.724338	0.724338	1.733362	-0.99899	CTSD
prednisolone-5101	1/100	0.561955	0.561955	0.724338	0.724338	1.734898	-0.99988	MMP1
phenyl biguanide-22	1/100	0.561955	0.561955	0.724338	0.724338	1.737804	-1.00155	TCN1
PNU-0251126-3692	1/100	0.561955	0.561955	0.724338	0.724338	1.739864	-1.00274	IFI6
pilocarpine-6741	1/100	0.561955	0.561955	0.724338	0.724338	1.741788	-1.00385	IFI6
pimethixene-2395	1/100	0.561955	0.561955	0.724338	0.724338	1.743895	-1.00507	ISG15
piperine-4830	1/100	0.561955	0.561955	0.724338	0.724338	1.746745	-1.00671	ID1
Prestwick-682-2164	1/100	0.561955	0.561955	0.724338	0.724338	1.748298	-1.0076	TFF2
piracetam-5043	1/100	0.561955	0.561955	0.724338	0.724338	1.748462	-1.0077	IFI6
piretanide-6144	1/100	0.561955	0.561955	0.724338	0.724338	1.750655	-1.00896	GPX2
PNU-0251126-7390	1/100	0.561955	0.561955	0.724338	0.724338	1.750758	-1.00902	CDH3
pipenzolate bromide-3460	1/100	0.561955	0.561955	0.724338	0.724338	1.751304	-1.00934	BMP4
piperidolate-6129	1/100	0.561955	0.561955	0.724338	0.724338	1.753481	-1.01059	TSPAN13
piribedil-3512	1/100	0.561955	0.561955	0.724338	0.724338	1.753512	-1.01061	GPX2
Prestwick-860-3378	1/100	0.561955	0.561955	0.724338	0.724338	1.754503	-1.01118	AP1S1
podophyllotoxin-7198	1/100	0.561955	0.561955	0.724338	0.724338	1.756543	-1.01235	TUBB
pivampicillin-5046	1/100	0.561955	0.561955	0.724338	0.724338	1.756661	-1.01242	AP1S1
phenformin-21	1/100	0.561955	0.561955	0.724338	0.724338	1.756749	-1.01247	TCN1
pioglitazone-7523	1/100	0.561955	0.561955	0.724338	0.724338	1.757685	-1.01301	ITM2C
Prestwick-692-4424	1/100	0.561955	0.561955	0.724338	0.724338	1.759747	-1.0142	AP1S1
Prestwick-1085-6131	1/100	0.561955	0.561955	0.724338	0.724338	1.760913	-1.01487	TCN1
pralidoxime-3066	1/100	0.561955	0.561955	0.724338	0.724338	1.762704	-1.01591	CTSD
Prestwick-685-2188	1/100	0.561955	0.561955	0.724338	0.724338	1.763556	-1.0164	CTSD
pirenperone-4679	1/100	0.561955	0.561955	0.724338	0.724338	1.76416	-1.01674	OLFM4
prednisolone-2393	1/100	0.561955	0.561955	0.724338	0.724338	1.765478	-1.0175	C3
prednisone-1478	1/100	0.561955	0.561955	0.724338	0.724338	1.765882	-1.01774	SOX9
PNU-0230031-4288	1/100	0.561955	0.561955	0.724338	0.724338	1.766784	-1.01826	CTGF
Prestwick-691-4092	1/100	0.561955	0.561955	0.724338	0.724338	1.766844	-1.01829	TGFBI
practolol-5664	1/100	0.561955	0.561955	0.724338	0.724338	1.769763	-1.01997	BMP4
pioglitazone-5930	1/100	0.561955	0.561955	0.724338	0.724338	1.772409	-1.0215	CTGF
pirinixic acid-464	1/100	0.561955	0.561955	0.724338	0.724338	1.773907	-1.02236	CTGF
PNU-0251126-7388	1/100	0.561955	0.561955	0.724338	0.724338	1.774423	-1.02266	CKS2

piribedil-2951	1/100	0.561955	0.561955	0.724338	0.724338	1.774606	-1.02276	RCN1
pivampicillin-3506	1/100	0.561955	0.561955	0.724338	0.724338	1.775561	-1.02332	S100A6
Prestwick-1100-3880	1/100	0.561955	0.561955	0.724338	0.724338	1.775634	-1.02336	S100A4
Prestwick-664-4275	1/100	0.561955	0.561955	0.724338	0.724338	1.776009	-1.02357	MMP3
Prestwick-674-3716	1/100	0.561955	0.561955	0.724338	0.724338	1.776548	-1.02388	PSMB9
Prestwick-642-2815	1/100	0.561955	0.561955	0.724338	0.724338	1.776705	-1.02397	BMP4
Prestwick-1100-4356	1/100	0.561955	0.561955	0.724338	0.724338	1.777919	-1.02467	IFI27
pralidoxime-6283	1/100	0.561955	0.561955	0.724338	0.724338	1.77959	-1.02564	CDH3
practolol-4603	1/100	0.561955	0.561955	0.724338	0.724338	1.780497	-1.02616	TSPAN1
prazosin-6315	1/100	0.561955	0.561955	0.724338	0.724338	1.780715	-1.02629	CDH3
Prestwick-665-3681	1/100	0.561955	0.561955	0.724338	0.724338	1.782583	-1.02736	MMP3
piracetam-5462	1/100	0.561955	0.561955	0.724338	0.724338	1.783273	-1.02776	GPX2
Prestwick-685-3683	1/100	0.561955	0.561955	0.724338	0.724338	1.783837	-1.02809	IFI6
procarbazine-2971	1/100	0.561955	0.561955	0.724338	0.724338	1.784282	-1.02834	ID3
PNU-0251126-3689	1/100	0.561955	0.561955	0.724338	0.724338	1.785163	-1.02885	TSPAN8
pramocaine-3894	1/100	0.561955	0.561955	0.724338	0.724338	1.7864	-1.02956	TFF2
prednisone-4577	1/100	0.561955	0.561955	0.724338	0.724338	1.789402	-1.03129	GPX2
Prestwick-972-7266	1/100	0.561955	0.561955	0.724338	0.724338	1.789405	-1.03129	CDH3
Prestwick-967-2925	1/100	0.561955	0.561955	0.724338	0.724338	1.789893	-1.03158	ID3
practolol-1587	1/100	0.561955	0.561955	0.724338	0.724338	1.792178	-1.03289	IFITM3
Prestwick-857-6635	1/100	0.561955	0.561955	0.724338	0.724338	1.793269	-1.03352	PSMB9
pronetalol-3984	1/100	0.561955	0.561955	0.724338	0.724338	1.794826	-1.03442	MMP3
Prestwick-559-2877	1/100	0.561955	0.561955	0.724338	0.724338	1.795065	-1.03456	TFF2
Prestwick-689-5816	1/100	0.561955	0.561955	0.724338	0.724338	1.795286	-1.03468	PDIA3
Prestwick-1103-3540	1/100	0.561955	0.561955	0.724338	0.724338	1.795296	-1.03469	RNF43
Prestwick-689-2514	1/100	0.561955	0.561955	0.724338	0.724338	1.796632	-1.03546	ANXA3
pirinixic acid-368	1/100	0.561955	0.561955	0.724338	0.724338	1.796983	-1.03566	MMP7
Prestwick-981-3125	1/100	0.561955	0.561955	0.724338	0.724338	1.797154	-1.03576	IFITM3
Prestwick-984-7323	1/100	0.561955	0.561955	0.724338	0.724338	1.800471	-1.03767	GPX2
probucol-5626	1/100	0.561955	0.561955	0.724338	0.724338	1.801898	-1.03849	TFF2
prenylamine-2886	1/100	0.561955	0.561955	0.724338	0.724338	1.802016	-1.03856	BMP4
Prestwick-1082-7027	1/100	0.561955	0.561955	0.724338	0.724338	1.802298	-1.03872	AP1S1
Prestwick-860-4618	1/100	0.561955	0.561955	0.724338	0.724338	1.802704	-1.03896	IGFBP2
propranolol-6759	1/100	0.561955	0.561955	0.724338	0.724338	1.803591	-1.03947	PDIA3
proglumide-3861	1/100	0.561955	0.561955	0.724338	0.724338	1.804159	-1.0398	TFF2
proglumide-4337	1/100	0.561955	0.561955	0.724338	0.724338	1.807317	-1.04162	CDH3
Prestwick-1100-4534	1/100	0.561955	0.561955	0.724338	0.724338	1.809022	-1.0426	TGFBI

Prestwick-664-2178	1/100	0.561955	0.561955	0.724338	0.724338	1.811431	-1.04399	ATP1B1
procainamide-1263	1/100	0.561955	0.561955	0.724338	0.724338	1.81284	-1.0448	SLPI
Prestwick-984-2903	1/100	0.561955	0.561955	0.724338	0.724338	1.813817	-1.04536	GPX2
Prestwick-972-6511	1/100	0.561955	0.561955	0.724338	0.724338	1.814213	-1.04559	TFF2
prilocaine-3624	1/100	0.561955	0.561955	0.724338	0.724338	1.814928	-1.046	TSPAN1
primaquine-3279	1/100	0.561955	0.561955	0.724338	0.724338	1.815047	-1.04607	BMP4
propafenone-1722	1/100	0.561955	0.561955	0.724338	0.724338	1.816448	-1.04688	DYNLL1
prochlorperazine-2675	1/100	0.561955	0.561955	0.724338	0.724338	1.81665	-1.047	ANXA3
Prestwick-674-6034	1/100	0.561955	0.561955	0.724338	0.724338	1.817394	-1.04742	BMP4
Prestwick-664-3715	1/100	0.561955	0.561955	0.724338	0.724338	1.818276	-1.04793	PSMB9
Prestwick-1100-3798	1/100	0.561955	0.561955	0.724338	0.724338	1.819255	-1.0485	C3
prochlorperazine-1156	1/100	0.561955	0.561955	0.724338	0.724338	1.820107	-1.04899	IFI6
Prestwick-692-2820	1/100	0.561955	0.561955	0.724338	0.724338	1.82229	-1.05025	SLPI
propafenone-2871	1/100	0.561955	0.561955	0.724338	0.724338	1.823888	-1.05117	LGALS3BP
prochlorperazine-6664	1/100	0.561955	0.561955	0.724338	0.724338	1.825125	-1.05188	TGFBI
pseudopelletierine-7184	1/100	0.561955	0.561955	0.724338	0.724338	1.825322	-1.05199	LGALS3BP
proglumide-2363	1/100	0.561955	0.561955	0.724338	0.724338	1.826085	-1.05243	ISG15
propranolol-5358	1/100	0.561955	0.561955	0.724338	0.724338	1.826308	-1.05256	ID1
Prestwick-691-4172	1/100	0.561955	0.561955	0.724338	0.724338	1.82849	-1.05382	AP1S1
pridinol-3456	1/100	0.561955	0.561955	0.724338	0.724338	1.828971	-1.0541	ID1
pridinol-7214	1/100	0.561955	0.561955	0.724338	0.724338	1.829347	-1.05431	ID3
Prestwick-920-5056	1/100	0.561955	0.561955	0.724338	0.724338	1.831072	-1.05531	IFI6
ramifenazone-6097	1/100	0.561955	0.561955	0.724338	0.724338	1.831778	-1.05572	SLPI
proadifen-7165	1/100	0.561955	0.561955	0.724338	0.724338	1.83302	-1.05643	PGM1
protoveratrine A-2800	1/100	0.561955	0.561955	0.724338	0.724338	1.833922	-1.05695	LY6E
procarbazine-3533	1/100	0.561955	0.561955	0.724338	0.724338	1.834129	-1.05707	LGALS3BP
pronetalol-4104	1/100	0.561955	0.561955	0.724338	0.724338	1.83474	-1.05742	IFI6
Prestwick-860-3040	1/100	0.561955	0.561955	0.724338	0.724338	1.834753	-1.05743	CCL20
pyrantel-2260	1/100	0.561955	0.561955	0.724338	0.724338	1.834851	-1.05749	MMP3
Prestwick-675-6042	1/100	0.561955	0.561955	0.724338	0.724338	1.835142	-1.05765	MMP3
Prestwick-984-6603	1/100	0.561955	0.561955	0.724338	0.724338	1.841336	-1.06122	PSMB9
Prestwick-692-2165	1/100	0.561955	0.561955	0.724338	0.724338	1.842447	-1.06186	TFF2
propoxycaine-6803	1/100	0.561955	0.561955	0.724338	0.724338	1.843087	-1.06223	IFI6
ribavirin-6018	1/100	0.561955	0.561955	0.724338	0.724338	1.844304	-1.06293	IFI6
proxyphylline-5993	1/100	0.561955	0.561955	0.724338	0.724338	1.846802	-1.06437	RNF43
propantheline bromide-4214	1/100	0.561955	0.561955	0.724338	0.724338	1.847419	-1.06473	PSMB9
prilocaine-4284	1/100	0.561955	0.561955	0.724338	0.724338	1.848014	-1.06507	LY6E

progesterone-2426	1/100	0.561955	0.561955	0.724338	0.724338	1.850845	-1.0667	SPP1
quercetin-4846	1/100	0.561955	0.561955	0.724338	0.724338	1.851454	-1.06706	ID1
profenamine-3038	1/100	0.561955	0.561955	0.724338	0.724338	1.85202	-1.06738	IGFBP2
propoxycaine-3583	1/100	0.561955	0.561955	0.724338	0.724338	1.852315	-1.06755	IFITM1
propoxycaine-7155	1/100	0.561955	0.561955	0.724338	0.724338	1.853726	-1.06836	IFI6
quercetin-4264	1/100	0.561955	0.561955	0.724338	0.724338	1.853992	-1.06852	TSPAN8
riboflavin-6822	1/100	0.561955	0.561955	0.724338	0.724338	1.85444	-1.06878	IFI27
procainamide-5663	1/100	0.561955	0.561955	0.724338	0.724338	1.854819	-1.06899	BMP4
promazine-3752	1/100	0.561955	0.561955	0.724338	0.724338	1.855378	-1.06932	BMP4
pyrazinamide-2478	1/100	0.561955	0.561955	0.724338	0.724338	1.855469	-1.06937	IGFBP2
promazine-3927	1/100	0.561955	0.561955	0.724338	0.724338	1.856117	-1.06974	BMP4
propranolol-3396	1/100	0.561955	0.561955	0.724338	0.724338	1.856616	-1.07003	QPCT
quinethazone-4529	1/100	0.561955	0.561955	0.724338	0.724338	1.857079	-1.0703	AP1S1
propofol-6707	1/100	0.561955	0.561955	0.724338	0.724338	1.858481	-1.0711	IFI6
primidone-3402	1/100	0.561955	0.561955	0.724338	0.724338	1.858507	-1.07112	SLPI
ricinine-6067	1/100	0.561955	0.561955	0.724338	0.724338	1.862674	-1.07352	SLPI
proscillaridin-4404	1/100	0.561955	0.561955	0.724338	0.724338	1.863147	-1.07379	BMP4
R-atenolol-4259	1/100	0.561955	0.561955	0.724338	0.724338	1.864466	-1.07455	TSPAN8
pyrantel-2097	1/100	0.561955	0.561955	0.724338	0.724338	1.865436	-1.07511	TCN1
quercetin-2859	1/100	0.561955	0.561955	0.724338	0.724338	1.868099	-1.07665	ID1
pyrazinamide-2839	1/100	0.561955	0.561955	0.724338	0.724338	1.868966	-1.07715	LGALS3BP
ribavirin-3142	1/100	0.561955	0.561955	0.724338	0.724338	1.869079	-1.07721	IFI6
quinisocaine-4207	1/100	0.561955	0.561955	0.724338	0.724338	1.869231	-1.0773	CTSD
pyrithyldione-2740	1/100	0.561955	0.561955	0.724338	0.724338	1.870984	-1.07831	IGFBP2
pseudopelletierine-2766	1/100	0.561955	0.561955	0.724338	0.724338	1.872569	-1.07922	TSPAN1
quipazine-2782	1/100	0.561955	0.561955	0.724338	0.724338	1.874627	-1.08041	C3
raloxifene-388	1/100	0.561955	0.561955	0.724338	0.724338	1.877348	-1.08198	DSG2
proxyphylline-3115	1/100	0.561955	0.561955	0.724338	0.724338	1.878341	-1.08255	IFI6
proadifen-3446	1/100	0.561955	0.561955	0.724338	0.724338	1.880425	-1.08375	S100A4
pyrvinium-5439	1/100	0.561955	0.561955	0.724338	0.724338	1.880935	-1.08405	BMP4
riluzole-2334	1/100	0.561955	0.561955	0.724338	0.724338	1.881812	-1.08455	C3
rescinnamine-2130	1/100	0.561955	0.561955	0.724338	0.724338	1.883152	-1.08532	C3
quinpirole-3539	1/100	0.561955	0.561955	0.724338	0.724338	1.883738	-1.08566	PDIA3
repaglinide-3558	1/100	0.561955	0.561955	0.724338	0.724338	1.883853	-1.08573	LGALS3BP
procaine-1838	1/100	0.561955	0.561955	0.724338	0.724338	1.884633	-1.08618	C3
rilmenidine-3133	1/100	0.561955	0.561955	0.724338	0.724338	1.884905	-1.08633	GPX2
pyrithyldione-7153	1/100	0.561955	0.561955	0.724338	0.724338	1.888013	-1.08813	CDH3

propidium iodide-6104	1/100	0.561955	0.561955	0.724338	0.724338	1.888821	-1.08859	SLPI
rotenone-5948	1/100	0.561955	0.561955	0.724338	0.724338	1.88905	-1.08872	PROM1
rilmenidine-6512	1/100	0.561955	0.561955	0.724338	0.724338	1.890184	-1.08938	SECTM1
quinidine-6267	1/100	0.561955	0.561955	0.724338	0.724338	1.892346	-1.09062	LGALS3BP
probucol-1608	1/100	0.561955	0.561955	0.724338	0.724338	1.897138	-1.09338	RPS14P3
rolipram-3072	1/100	0.561955	0.561955	0.724338	0.724338	1.897289	-1.09347	SLPI
pyrimethamine-1894	1/100	0.561955	0.561955	0.724338	0.724338	1.898936	-1.09442	SECTM1
pyrvinium-2957	1/100	0.561955	0.561955	0.724338	0.724338	1.899795	-1.09492	SPP1
R-atenolol-2855	1/100	0.561955	0.561955	0.724338	0.724338	1.901318	-1.09579	TFF2
rifampicin-4008	1/100	0.561955	0.561955	0.724338	0.724338	1.901598	-1.09595	MMP3
resveratrol-958	1/100	0.561955	0.561955	0.724338	0.724338	1.902252	-1.09633	AP1S1
ribostamycin-3444	1/100	0.561955	0.561955	0.724338	0.724338	1.902462	-1.09645	LGALS3BP
raubasine-2898	1/100	0.561955	0.561955	0.724338	0.724338	1.905117	-1.09798	PGM1
rosiglitazone-1174	1/100	0.561955	0.561955	0.724338	0.724338	1.906354	-1.0987	IFI6
ramifenazone-2534	1/100	0.561955	0.561955	0.724338	0.724338	1.907968	-1.09963	GPX2
rosiglitazone-1658	1/100	0.561955	0.561955	0.724338	0.724338	1.912131	-1.10203	S100A4
pyrantel-5088	1/100	0.561955	0.561955	0.724338	0.724338	1.912686	-1.10234	IFI6
rosiglitazone-1013	1/100	0.561955	0.561955	0.724338	0.724338	1.913277	-1.10269	TSPAN1
ramifenazone-7233	1/100	0.561955	0.561955	0.724338	0.724338	1.913952	-1.10307	GPX2
scopolamine N-oxide-2099	1/100	0.561955	0.561955	0.724338	0.724338	1.914796	-1.10356	CTSD
remoxipride-3124	1/100	0.561955	0.561955	0.724338	0.724338	1.91499	-1.10367	GPX2
pyridoxine-5813	1/100	0.561955	0.561955	0.724338	0.724338	1.915441	-1.10393	CTSD
puromycin-2448	1/100	0.561955	0.561955	0.724338	0.724338	1.916152	-1.10434	CD14
R-atenolol-2496	1/100	0.561955	0.561955	0.724338	0.724338	1.916229	-1.10439	IFI6
resveratrol-662	1/100	0.561955	0.561955	0.724338	0.724338	1.916232	-1.10439	GPX2
rescinnamine-2785	1/100	0.561955	0.561955	0.724338	0.724338	1.916593	-1.1046	TMPRSS3
riluzole-7365	1/100	0.561955	0.561955	0.724338	0.724338	1.917551	-1.10515	IGFBP2
quipazine-7240	1/100	0.561955	0.561955	0.724338	0.724338	1.917797	-1.10529	TGFBI
ritodrine-4619	1/100	0.561955	0.561955	0.724338	0.724338	1.918385	-1.10563	CTGF
quinpirole-2977	1/100	0.561955	0.561955	0.724338	0.724338	1.921289	-1.1073	RCN1
rosiglitazone-369	1/100	0.561955	0.561955	0.724338	0.724338	1.922764	-1.10815	DSG2
riluzole-4689	1/100	0.561955	0.561955	0.724338	0.724338	1.922798	-1.10817	RCN1
raloxifene-2738	1/100	0.561955	0.561955	0.724338	0.724338	1.924352	-1.10907	SLPI
roxithromycin-3331	1/100	0.561955	0.561955	0.724338	0.724338	1.924397	-1.10909	AP1S1
saquinavir-3549	1/100	0.561955	0.561955	0.724338	0.724338	1.926954	-1.11057	S100A4
S-propranolol-3523	1/100	0.561955	0.561955	0.724338	0.724338	1.927113	-1.11066	LGALS3BP
retrorsine-2129	1/100	0.561955	0.561955	0.724338	0.724338	1.928784	-1.11162	CTSD

rimexolone-5092	1/100	0.561955	0.561955	0.724338	0.724338	1.93085	-1.11281	IFI6
protriptyline-3119	1/100	0.561955	0.561955	0.724338	0.724338	1.933054	-1.11408	LY6E
sanguinarine-4168	1/100	0.561955	0.561955	0.724338	0.724338	1.933373	-1.11427	AP1S1
ronidazole-6134	1/100	0.561955	0.561955	0.724338	0.724338	1.935419	-1.11545	ANXA3
quipazine-1789	1/100	0.561955	0.561955	0.724338	0.724338	1.935769	-1.11565	C3
saquinavir-6127	1/100	0.561955	0.561955	0.724338	0.724338	1.935877	-1.11571	IFI6
rosiglitazone-430	1/100	0.561955	0.561955	0.724338	0.724338	1.937134	-1.11644	CTGF
scopoletin-6510	1/100	0.561955	0.561955	0.724338	0.724338	1.938021	-1.11695	TGFBI
raloxifene-376	1/100	0.561955	0.561955	0.724338	0.724338	1.93847	-1.11721	CTSE
SB-202190-6882	1/100	0.561955	0.561955	0.724338	0.724338	1.938644	-1.11731	RNF43
remoxipride-5443	1/100	0.561955	0.561955	0.724338	0.724338	1.939971	-1.11807	S100A4
reserpine-3003	1/100	0.561955	0.561955	0.724338	0.724338	1.940226	-1.11822	LY6E
salsolinol-4232	1/100	0.561955	0.561955	0.724338	0.724338	1.941317	-1.11885	TSPAN8
scoulerine-5536	1/100	0.561955	0.561955	0.724338	0.724338	1.943564	-1.12014	RNF43
rifabutin-4349	1/100	0.561955	0.561955	0.724338	0.724338	1.944199	-1.12051	CTSD
roxithromycin-4778	1/100	0.561955	0.561955	0.724338	0.724338	1.944932	-1.12093	IFI6
sirolimus-402	1/100	0.561955	0.561955	0.724338	0.724338	1.945484	-1.12125	RCN1
SB-203580-7066	1/100	0.561955	0.561955	0.724338	0.724338	1.947261	-1.12227	IFI6
repaglinide-7216	1/100	0.561955	0.561955	0.724338	0.724338	1.947435	-1.12237	S100A4
quercetin-2499	1/100	0.561955	0.561955	0.724338	0.724338	1.948252	-1.12284	IFI6
SC-560-6870	1/100	0.561955	0.561955	0.724338	0.724338	1.949314	-1.12346	TMPRSS3
sparteine-4568	1/100	0.561955	0.561955	0.724338	0.724338	1.949697	-1.12368	RPS14P3
rimexolone-2955	1/100	0.561955	0.561955	0.724338	0.724338	1.95394	-1.12612	SPP1
rosiglitazone-6950	1/100	0.561955	0.561955	0.724338	0.724338	1.954784	-1.12661	IFNGR2
SC-19220-7060	1/100	0.561955	0.561955	0.724338	0.724338	1.954879	-1.12666	IFI6
securinine-6831	1/100	0.561955	0.561955	0.724338	0.724338	1.95538	-1.12695	TXNIP
saquinavir-6246	1/100	0.561955	0.561955	0.724338	0.724338	1.956564	-1.12763	LGALS3BP
salsolinol-4816	1/100	0.561955	0.561955	0.724338	0.724338	1.958292	-1.12863	TSPAN1
SB-203580-6894	1/100	0.561955	0.561955	0.724338	0.724338	1.95914	-1.12912	C3
selegiline-4065	1/100	0.561955	0.561955	0.724338	0.724338	1.960029	-1.12963	LUM
sitosterol-4154	1/100	0.561955	0.561955	0.724338	0.724338	1.962776	-1.13121	AP1S1
pyridoxine-2751	1/100	0.561955	0.561955	0.724338	0.724338	1.963198	-1.13146	RNF43
STOCK1N-35215-6427	1/100	0.561955	0.561955	0.724338	0.724338	1.96368	-1.13173	TGFBI
SC-58125-507	1/100	0.561955	0.561955	0.724338	0.724338	1.964822	-1.13239	ID3
resveratrol-841	1/100	0.561955	0.561955	0.724338	0.724338	1.964965	-1.13248	ID1
serotonin-2449	1/100	0.561955	0.561955	0.724338	0.724338	1.965459	-1.13276	IGFBP2
scopoletin-3131	1/100	0.561955	0.561955	0.724338	0.724338	1.966719	-1.13349	GPX2

rotenone-5915	1/100	0.561955	0.561955	0.724338	0.724338	1.970593	-1.13572	ITM2C
scopolamine N-oxide-5436	1/100	0.561955	0.561955	0.724338	0.724338	1.971759	-1.13639	GPX2
roxithromycin-2992	1/100	0.561955	0.561955	0.724338	0.724338	1.97216	-1.13662	ISG15
salbutamol-7376	1/100	0.561955	0.561955	0.724338	0.724338	1.975076	-1.1383	CTGF
roxithromycin-4192	1/100	0.561955	0.561955	0.724338	0.724338	1.977379	-1.13963	GPX2
rottlerin-941	1/100	0.561955	0.561955	0.724338	0.724338	1.977587	-1.13975	BMP4
SC-19220-7095	1/100	0.561955	0.561955	0.724338	0.724338	1.979804	-1.14103	LY6E
spectinomycin-3327	1/100	0.561955	0.561955	0.724338	0.724338	1.980174	-1.14124	RNF43
sirolimus-1022	1/100	0.561955	0.561955	0.724338	0.724338	1.981784	-1.14217	BMP4
sirolimus-326	1/100	0.561955	0.561955	0.724338	0.724338	1.983858	-1.14336	MMP3
rimexolone-5517	1/100	0.561955	0.561955	0.724338	0.724338	1.9849	-1.14396	LGALS3BP
sirolimus-921	1/100	0.561955	0.561955	0.724338	0.724338	1.985191	-1.14413	TMPRSS3
ronidazole-7131	1/100	0.561955	0.561955	0.724338	0.724338	1.985578	-1.14436	S100P
sirolimus-5980	1/100	0.561955	0.561955	0.724338	0.724338	1.985644	-1.14439	BMP4
SR-95639A-3272	1/100	0.561955	0.561955	0.724338	0.724338	1.986118	-1.14467	RPS14P3
salbutamol-4700	1/100	0.561955	0.561955	0.724338	0.724338	1.989313	-1.14651	RCN1
ritodrine-2635	1/100	0.561955	0.561955	0.724338	0.724338	1.990622	-1.14726	TSPAN1
SB-202190-6887	1/100	0.561955	0.561955	0.724338	0.724338	1.991963	-1.14804	RNF43
sotalol-2918	1/100	0.561955	0.561955	0.724338	0.724338	1.992451	-1.14832	IFI6
sulindac-5103	1/100	0.561955	0.561955	0.724338	0.724338	1.993102	-1.14869	TSPAN1
roxarsone-5051	1/100	0.561955	0.561955	0.724338	0.724338	1.995074	-1.14983	LGALS3BP
sirolimus-4445	1/100	0.561955	0.561955	0.724338	0.724338	1.996108	-1.15042	C3
sirolimus-5932	1/100	0.561955	0.561955	0.724338	0.724338	1.996406	-1.1506	PSMB9
SB-202190-7058	1/100	0.561955	0.561955	0.724338	0.724338	1.997138	-1.15102	TSPAN1
spiradoline-3818	1/100	0.561955	0.561955	0.724338	0.724338	1.997666	-1.15132	IL32
selegiline-4146	1/100	0.561955	0.561955	0.724338	0.724338	1.997757	-1.15137	AP1S1
sirolimus-5975	1/100	0.561955	0.561955	0.724338	0.724338	1.997967	-1.1515	ITM2C
rotenone-5920	1/100	0.561955	0.561955	0.724338	0.724338	1.99848	-1.15179	IL32
semustine-7540	1/100	0.561955	0.561955	0.724338	0.724338	1.999411	-1.15233	IFI6
spironolactone-5781	1/100	0.561955	0.561955	0.724338	0.724338	2.002249	-1.15396	CXCL1
sirolimus-5218	1/100	0.561955	0.561955	0.724338	0.724338	2.002607	-1.15417	IFI6
sirolimus-1221	1/100	0.561955	0.561955	0.724338	0.724338	2.004963	-1.15553	BMP4
spironolactone-1380	1/100	0.561955	0.561955	0.724338	0.724338	2.006202	-1.15624	PIGR
STOCK1N-28457-6864	1/100	0.561955	0.561955	0.724338	0.724338	2.006759	-1.15656	IFI6
salsolidin-2824	1/100	0.561955	0.561955	0.724338	0.724338	2.006997	-1.1567	BMP4
sulfamethizole-6099	1/100	0.561955	0.561955	0.724338	0.724338	2.007291	-1.15687	TFF2
sotalol-4160	1/100	0.561955	0.561955	0.724338	0.724338	2.008987	-1.15785	AP1S1

succinylsulfathiazole-4265	1/100	0.561955	0.561955	0.724338	0.724338	2.00905	-1.15788	TSPAN8
sirolimus-6180	1/100	0.561955	0.561955	0.724338	0.724338	2.009152	-1.15794	ID3
spiperone-1559	1/100	0.561955	0.561955	0.724338	0.724338	2.012161	-1.15968	LYZ
sirolimus-2681	1/100	0.561955	0.561955	0.724338	0.724338	2.01292	-1.16011	C3
scoulerine-2891	1/100	0.561955	0.561955	0.724338	0.724338	2.014168	-1.16083	BMP4
sertaconazole-4475	1/100	0.561955	0.561955	0.724338	0.724338	2.017023	-1.16248	IFI6
spiradoline-4375	1/100	0.561955	0.561955	0.724338	0.724338	2.017029	-1.16248	MMP3
sulfametoxydiazine-3453	1/100	0.561955	0.561955	0.724338	0.724338	2.017983	-1.16303	ETS2
scopolamine-4803	1/100	0.561955	0.561955	0.724338	0.724338	2.022075	-1.16539	IFI6
spaglumic acid-2962	1/100	0.561955	0.561955	0.724338	0.724338	2.022911	-1.16587	CKB
sulfapyridine-6101	1/100	0.561955	0.561955	0.724338	0.724338	2.024438	-1.16675	QPCT
staurosporine-425	1/100	0.561955	0.561955	0.724338	0.724338	2.024887	-1.16701	CDH3
sulfadimidine-3940	1/100	0.561955	0.561955	0.724338	0.724338	2.025194	-1.16719	IFI6
sirolimus-5927	1/100	0.561955	0.561955	0.724338	0.724338	2.027741	-1.16865	TFF2
sirolimus-1148	1/100	0.561955	0.561955	0.724338	0.724338	2.027872	-1.16873	ANXA3
sulfachlorpyridazine-3944	1/100	0.561955	0.561955	0.724338	0.724338	2.028422	-1.16905	MMP3
selegiline-2465	1/100	0.561955	0.561955	0.724338	0.724338	2.028885	-1.16931	CCL20
sisomicin-2853	1/100	0.561955	0.561955	0.724338	0.724338	2.029112	-1.16945	TFF2
staurosporine-423	1/100	0.561955	0.561955	0.724338	0.724338	2.029868	-1.16988	ID1
sisomicin-2493	1/100	0.561955	0.561955	0.724338	0.724338	2.030752	-1.17039	IFI6
skimmianine-5766	1/100	0.561955	0.561955	0.724338	0.724338	2.031309	-1.17071	GPX2
terguride-5400	1/100	0.561955	0.561955	0.724338	0.724338	2.032609	-1.17146	TGFBI
sulfapyridine-6799	1/100	0.561955	0.561955	0.724338	0.724338	2.033939	-1.17223	IFI6
spironolactone-6255	1/100	0.561955	0.561955	0.724338	0.724338	2.035174	-1.17294	TSPAN1
sirolimus-987	1/100	0.561955	0.561955	0.724338	0.724338	2.038835	-1.17505	IFNGR2
sulfamethoxypyridazine-3409	1/100	0.561955	0.561955	0.724338	0.724338	2.039397	-1.17537	AP1S1
sulfadimidine-4322	1/100	0.561955	0.561955	0.724338	0.724338	2.040195	-1.17583	IFI6
sodium phenylbutyrate-408	1/100	0.561955	0.561955	0.724338	0.724338	2.040618	-1.17608	SLPI
sparteine-2134	1/100	0.561955	0.561955	0.724338	0.724338	2.042463	-1.17714	LY6E
sulfamethoxypyridazine-4733	1/100	0.561955	0.561955	0.724338	0.724338	2.045076	-1.17865	IFI6
sirolimus-6940	1/100	0.561955	0.561955	0.724338	0.724338	2.047282	-1.17992	LGALS3BP
spiramycin-3419	1/100	0.561955	0.561955	0.724338	0.724338	2.047394	-1.17998	TSPAN1
succinylsulfathiazole-2821	1/100	0.561955	0.561955	0.724338	0.724338	2.048132	-1.18041	RNF43
sulfafurazole-5622	1/100	0.561955	0.561955	0.724338	0.724338	2.048204	-1.18045	GPX2
solasodine-3924	1/100	0.561955	0.561955	0.724338	0.724338	2.05062	-1.18184	IFI6
sirolimus-1242	1/100	0.561955	0.561955	0.724338	0.724338	2.051351	-1.18226	SECTM1
sodium phenylbutyrate-341	1/100	0.561955	0.561955	0.724338	0.724338	2.053927	-1.18375	TMPRSS3

sulfacetamide-1695	1/100	0.561955	0.561955	0.724338	0.724338	2.054421	-1.18403	S100A4
solasodine-2170	1/100	0.561955	0.561955	0.724338	0.724338	2.055897	-1.18488	IFI6
sulfaphenazole-1794	1/100	0.561955	0.561955	0.724338	0.724338	2.056808	-1.18541	LY6E
solanine-2808	1/100	0.561955	0.561955	0.724338	0.724338	2.056893	-1.18546	BMP4
sulfanilamide-2709	1/100	0.561955	0.561955	0.724338	0.724338	2.057019	-1.18553	TSPAN13
spiramycin-4319	1/100	0.561955	0.561955	0.724338	0.724338	2.057224	-1.18565	IFI6
splitomicin-661	1/100	0.561955	0.561955	0.724338	0.724338	2.057312	-1.1857	GPX2
suramin sodium-7496	1/100	0.561955	0.561955	0.724338	0.724338	2.057512	-1.18581	SPINK1
sodium phenylbutyrate-363	1/100	0.561955	0.561955	0.724338	0.724338	2.058266	-1.18625	IFITM3
sulfadiazine-1810	1/100	0.561955	0.561955	0.724338	0.724338	2.059668	-1.18706	IFI6
simvastatin-3002	1/100	0.561955	0.561955	0.724338	0.724338	2.060801	-1.18771	C3
sulfadiazine-5098	1/100	0.561955	0.561955	0.724338	0.724338	2.062138	-1.18848	IFI6
sulfaguanidine-1995	1/100	0.561955	0.561955	0.724338	0.724338	2.062465	-1.18867	ITM2C
sulfacetamide-1859	1/100	0.561955	0.561955	0.724338	0.724338	2.062514	-1.1887	C3
STOCK1N-35874-6561	1/100	0.561955	0.561955	0.724338	0.724338	2.063343	-1.18917	TSPAN1
sulfamethoxazole-7366	1/100	0.561955	0.561955	0.724338	0.724338	2.067617	-1.19164	MMP1
sulfadoxine-7205	1/100	0.561955	0.561955	0.724338	0.724338	2.069329	-1.19262	S100A4
STOCK1N-28457-6869	1/100	0.561955	0.561955	0.724338	0.724338	2.071554	-1.19391	MMP3
sulfamonomethoxine-7200	1/100	0.561955	0.561955	0.724338	0.724338	2.072376	-1.19438	BMP4
tetrandrine-6082	1/100	0.561955	0.561955	0.724338	0.724338	2.072736	-1.19459	AP1S1
sirolimus-1059	1/100	0.561955	0.561955	0.724338	0.724338	2.073359	-1.19495	S100A6
sulfathiazole-1463	1/100	0.561955	0.561955	0.724338	0.724338	2.076792	-1.19692	TSPAN1
sulfathiazole-1883	1/100	0.561955	0.561955	0.724338	0.724338	2.077025	-1.19706	CTGF
sulfachlorpyridazine-3769	1/100	0.561955	0.561955	0.724338	0.724338	2.079881	-1.19871	TSPAN8
spaglumic acid-7465	1/100	0.561955	0.561955	0.724338	0.724338	2.081799	-1.19981	AP1S1
sulfinpyrazone-6230	1/100	0.561955	0.561955	0.724338	0.724338	2.081891	-1.19986	PGM1
sulfapyridine-2538	1/100	0.561955	0.561955	0.724338	0.724338	2.08336	-1.20071	TSPAN13
sulfamonomethoxine-3484	1/100	0.561955	0.561955	0.724338	0.724338	2.083827	-1.20098	S100A4
spiradoline-3901	1/100	0.561955	0.561955	0.724338	0.724338	2.084283	-1.20124	S100A4
STOCK1N-35215-6380	1/100	0.561955	0.561955	0.724338	0.724338	2.087816	-1.20328	MMP3
tanespimycin-6184	1/100	0.561955	0.561955	0.724338	0.724338	2.087818	-1.20328	ID3
suloctidil-6675	1/100	0.561955	0.561955	0.724338	0.724338	2.089057	-1.20399	TGFBI
tamoxifen-375	1/100	0.561955	0.561955	0.724338	0.724338	2.089776	-1.20441	CTSE
sulfadiazine-1852	1/100	0.561955	0.561955	0.724338	0.724338	2.093062	-1.2063	CTSD
stachydrine-1751	1/100	0.561955	0.561955	0.724338	0.724338	2.093184	-1.20637	ITGAV
succinylsulfathiazole-4847	1/100	0.561955	0.561955	0.724338	0.724338	2.093375	-1.20648	PSME2
syrosingopine-6213	1/100	0.561955	0.561955	0.724338	0.724338	2.09412	-1.20691	ENC1

tamoxifen-6768	1/100	0.561955	0.561955	0.724338	0.724338	2.095566	-1.20774	IFI6
tacrine-1278	1/100	0.561955	0.561955	0.724338	0.724338	2.098615	-1.2095	C3
sulfinpyrazone-5753	1/100	0.561955	0.561955	0.724338	0.724338	2.099116	-1.20979	IFI6
tacrolimus-378	1/100	0.561955	0.561955	0.724338	0.724338	2.100526	-1.2106	DPEP1
sulfacetamide-1817	1/100	0.561955	0.561955	0.724338	0.724338	2.100888	-1.21081	IL32
tenoxicam-2860	1/100	0.561955	0.561955	0.724338	0.724338	2.100889	-1.21081	TFF2
sulfathiazole-1963	1/100	0.561955	0.561955	0.724338	0.724338	2.101227	-1.21101	MEST
sulfinpyrazone-3192	1/100	0.561955	0.561955	0.724338	0.724338	2.101368	-1.21109	LYZ
tacrolimus-284	1/100	0.561955	0.561955	0.724338	0.724338	2.102128	-1.21153	IFI6
sulpiride-4566	1/100	0.561955	0.561955	0.724338	0.724338	2.103438	-1.21228	IFI6
terazosin-5831	1/100	0.561955	0.561955	0.724338	0.724338	2.105101	-1.21324	ITM2C
thioproperazine-2073	1/100	0.561955	0.561955	0.724338	0.724338	2.105205	-1.2133	TFF2
syrosingopine-2753	1/100	0.561955	0.561955	0.724338	0.724338	2.106217	-1.21388	TMPRSS3
tanespimycin-2686	1/100	0.561955	0.561955	0.724338	0.724338	2.106877	-1.21426	IER2
sulfanilamide-4474	1/100	0.561955	0.561955	0.724338	0.724338	2.107342	-1.21453	IFI6
sulpiride-1887	1/100	0.561955	0.561955	0.724338	0.724338	2.107602	-1.21468	IFI6
tetrandrine-2520	1/100	0.561955	0.561955	0.724338	0.724338	2.107795	-1.21479	ANXA3
suramin sodium-7501	1/100	0.561955	0.561955	0.724338	0.724338	2.110021	-1.21608	IFI6
sulmazole-4127	1/100	0.561955	0.561955	0.724338	0.724338	2.113305	-1.21797	MMP3
tanespimycin-1159	1/100	0.561955	0.561955	0.724338	0.724338	2.115542	-1.21926	PGM1
terazosin-2530	1/100	0.561955	0.561955	0.724338	0.724338	2.11639	-1.21975	GPX2
tanespimycin-1650	1/100	0.561955	0.561955	0.724338	0.724338	2.118387	-1.2209	BMP4
sulfamethoxazole-3667	1/100	0.561955	0.561955	0.724338	0.724338	2.118858	-1.22117	IFI6
thalidomide-1411	1/100	0.561955	0.561955	0.724338	0.724338	2.119597	-1.22159	SLPI
tetracycline-1397	1/100	0.561955	0.561955	0.724338	0.724338	2.126651	-1.22566	LYZ
sulindac-307	1/100	0.561955	0.561955	0.724338	0.724338	2.12941	-1.22725	LCN2
terguride-3096	1/100	0.561955	0.561955	0.724338	0.724338	2.13129	-1.22833	LY6E
theobromine-6613	1/100	0.561955	0.561955	0.724338	0.724338	2.131963	-1.22872	CTSD
sulfasalazine-2882	1/100	0.561955	0.561955	0.724338	0.724338	2.133405	-1.22955	SECTM1
tolazoline-1918	1/100	0.561955	0.561955	0.724338	0.724338	2.134224	-1.23002	IGFBP2
tetryzoline-6769	1/100	0.561955	0.561955	0.724338	0.724338	2.13441	-1.23013	TFF2
timolol-5645	1/100	0.561955	0.561955	0.724338	0.724338	2.13506	-1.23051	RNF43
sulfamerazine-2181	1/100	0.561955	0.561955	0.724338	0.724338	2.136313	-1.23123	LY6E
theophylline-4812	1/100	0.561955	0.561955	0.724338	0.724338	2.138378	-1.23242	GPX2
syrosingopine-1761	1/100	0.561955	0.561955	0.724338	0.724338	2.139303	-1.23295	MEST
tanespimycin-6166	1/100	0.561955	0.561955	0.724338	0.724338	2.144926	-1.23619	ID3
thioridazine-4164	1/100	0.561955	0.561955	0.724338	0.724338	2.145463	-1.2365	AP1S1

sulindac sulfide-308	1/100	0.561955	0.561955	0.724338	0.724338	2.146565	-1.23714	LCN2
thalidomide-5990	1/100	0.561955	0.561955	0.724338	0.724338	2.146981	-1.23738	IFI6
tinidazole-3430	1/100	0.561955	0.561955	0.724338	0.724338	2.148043	-1.23799	TMPRSS3
terazosin-6092	1/100	0.561955	0.561955	0.724338	0.724338	2.148136	-1.23804	SLPI
thiocolchicoside-5095	1/100	0.561955	0.561955	0.724338	0.724338	2.148452	-1.23823	IFI6
theobromine-3334	1/100	0.561955	0.561955	0.724338	0.724338	2.150198	-1.23923	ID1
tanespimycin-1006	1/100	0.561955	0.561955	0.724338	0.724338	2.150234	-1.23925	ID1
thiocolchicoside-5520	1/100	0.561955	0.561955	0.724338	0.724338	2.150316	-1.2393	GPX2
thiethylperazine-6154	1/100	0.561955	0.561955	0.724338	0.724338	2.150575	-1.23945	GPX2
tetramisole-2849	1/100	0.561955	0.561955	0.724338	0.724338	2.152109	-1.24033	TFF2
thioridazine-422	1/100	0.561955	0.561955	0.724338	0.724338	2.15383	-1.24132	ITGAV
tamoxifen-1366	1/100	0.561955	0.561955	0.724338	0.724338	2.153877	-1.24135	PIGR
ticlopidine-1975	1/100	0.561955	0.561955	0.724338	0.724338	2.154563	-1.24175	SLPI
thiethylperazine-6232	1/100	0.561955	0.561955	0.724338	0.724338	2.155615	-1.24235	LGALS3BP
tetrandrine-7178	1/100	0.561955	0.561955	0.724338	0.724338	2.155623	-1.24236	LGALS3BP
tanespimycin-1147	1/100	0.561955	0.561955	0.724338	0.724338	2.156284	-1.24274	IFITM3
tinidazole-4548	1/100	0.561955	0.561955	0.724338	0.724338	2.156954	-1.24312	CDH3
tolbutamide-3886	1/100	0.561955	0.561955	0.724338	0.724338	2.159642	-1.24467	C3
suxibuzone-6065	1/100	0.561955	0.561955	0.724338	0.724338	2.159797	-1.24476	AP1S1
thioridazine-1655	1/100	0.561955	0.561955	0.724338	0.724338	2.161163	-1.24555	LGALS3BP
tolazoline-4262	1/100	0.561955	0.561955	0.724338	0.724338	2.161253	-1.2456	TSPAN8
thioproperazine-5791	1/100	0.561955	0.561955	0.724338	0.724338	2.161417	-1.2457	MMP3
tiaprofenic acid-4091	1/100	0.561955	0.561955	0.724338	0.724338	2.161557	-1.24578	IFI6
tanespimycin-6185	1/100	0.561955	0.561955	0.724338	0.724338	2.162193	-1.24614	ID3
tretinoin-6243	1/100	0.561955	0.561955	0.724338	0.724338	2.164471	-1.24746	ISG15
suprofen-3343	1/100	0.561955	0.561955	0.724338	0.724338	2.166392	-1.24856	BMP4
tiapride-2292	1/100	0.561955	0.561955	0.724338	0.724338	2.166782	-1.24879	BMP4
tetrahydroalstonine-5728	1/100	0.561955	0.561955	0.724338	0.724338	2.167162	-1.24901	IFI6
syrosingopine-5733	1/100	0.561955	0.561955	0.724338	0.724338	2.167217	-1.24904	CTGF
testosterone-4676	1/100	0.561955	0.561955	0.724338	0.724338	2.16727	-1.24907	IL32
tomatidine-5808	1/100	0.561955	0.561955	0.724338	0.724338	2.168257	-1.24964	S100P
thioridazine-1010	1/100	0.561955	0.561955	0.724338	0.724338	2.171273	-1.25138	BMP4
tolfenamic acid-5454	1/100	0.561955	0.561955	0.724338	0.724338	2.171502	-1.25151	IFI6
thiamphenicol-1704	1/100	0.561955	0.561955	0.724338	0.724338	2.172037	-1.25182	RPS14P3
thiostrepton-4563	1/100	0.561955	0.561955	0.724338	0.724338	2.174875	-1.25345	CXCL1
tiaprofenic acid-4171	1/100	0.561955	0.561955	0.724338	0.724338	2.176483	-1.25438	AP1S1
thapsigargin-7053	1/100	0.561955	0.561955	0.724338	0.724338	2.177931	-1.25521	BMP4

tetracycline-2243	1/100	0.561955	0.561955	0.724338	0.724338	2.178308	-1.25543	TFF2
tretinoin-849	1/100	0.561955	0.561955	0.724338	0.724338	2.179926	-1.25636	BMP4
tolazamide-4003	1/100	0.561955	0.561955	0.724338	0.724338	2.180544	-1.25672	TGFBI
thioproperazine-1390	1/100	0.561955	0.561955	0.724338	0.724338	2.181366	-1.25719	IFITM3
tranexamic acid-1401	1/100	0.561955	0.561955	0.724338	0.724338	2.184026	-1.25873	SLPI
thioguanosine-6643	1/100	0.561955	0.561955	0.724338	0.724338	2.185887	-1.2598	C3
tiaprofenic acid-2492	1/100	0.561955	0.561955	0.724338	0.724338	2.186526	-1.26017	IFI6
triamcinolone-5835	1/100	0.561955	0.561955	0.724338	0.724338	2.18693	-1.2604	TSPAN8
thiamine-2894	1/100	0.561955	0.561955	0.724338	0.724338	2.189633	-1.26196	TGFBI
tiapride-4686	1/100	0.561955	0.561955	0.724338	0.724338	2.18968	-1.26199	BMP4
thiamine-7479	1/100	0.561955	0.561955	0.724338	0.724338	2.189739	-1.26202	GPX2
tomatidine-1754	1/100	0.561955	0.561955	0.724338	0.724338	2.18989	-1.26211	RPS14P3
thalidomide-606	1/100	0.561955	0.561955	0.724338	0.724338	2.191706	-1.26315	MMP3
tolazoline-1500	1/100	0.561955	0.561955	0.724338	0.724338	2.192211	-1.26344	GPX2
thiocolchicoside-2875	1/100	0.561955	0.561955	0.724338	0.724338	2.193536	-1.26421	C3
thioperamide-5635	1/100	0.561955	0.561955	0.724338	0.724338	2.193774	-1.26435	RNF43
tiapride-7362	1/100	0.561955	0.561955	0.724338	0.724338	2.195245	-1.26519	IGFBP2
thalidomide-2258	1/100	0.561955	0.561955	0.724338	0.724338	2.195487	-1.26533	BMP4
tetryzoline-6069	1/100	0.561955	0.561955	0.724338	0.724338	2.195743	-1.26548	RPS14P3
tioguanine-642	1/100	0.561955	0.561955	0.724338	0.724338	2.196581	-1.26596	GPX2
thioridazine-1171	1/100	0.561955	0.561955	0.724338	0.724338	2.197686	-1.2666	MEST
thioperamide-3392	1/100	0.561955	0.561955	0.724338	0.724338	2.197886	-1.26672	PDIA3
todralazine-1841	1/100	0.561955	0.561955	0.724338	0.724338	2.1982	-1.2669	SPP1
trichostatin A-7105	1/100	0.561955	0.561955	0.724338	0.724338	2.19845	-1.26704	AP1S1
trapidil-7475	1/100	0.561955	0.561955	0.724338	0.724338	2.201535	-1.26882	LGALS3BP
tacrine-2633	1/100	0.561955	0.561955	0.724338	0.724338	2.20261	-1.26944	TSPAN1
tolfenamic acid-6354	1/100	0.561955	0.561955	0.724338	0.724338	2.204282	-1.2704	TSPAN1
thioridazine-1905	1/100	0.561955	0.561955	0.724338	0.724338	2.204731	-1.27066	ID3
tremorine-3196	1/100	0.561955	0.561955	0.724338	0.724338	2.205176	-1.27092	LYZ
tremorine-6273	1/100	0.561955	0.561955	0.724338	0.724338	2.206242	-1.27153	SECTM1
trichostatin A-2721	1/100	0.561955	0.561955	0.724338	0.724338	2.206671	-1.27178	PLCB4
tiapride-2331	1/100	0.561955	0.561955	0.724338	0.724338	2.206825	-1.27187	C3
tolbutamide-4540	1/100	0.561955	0.561955	0.724338	0.724338	2.207151	-1.27206	C3
tranylcypromine-2264	1/100	0.561955	0.561955	0.724338	0.724338	2.207869	-1.27247	BMP4
trichostatin A-2474	1/100	0.561955	0.561955	0.724338	0.724338	2.208876	-1.27305	RCN1
tetramisole-4587	1/100	0.561955	0.561955	0.724338	0.724338	2.20916	-1.27321	GPX2
thapsigargin-7103	1/100	0.561955	0.561955	0.724338	0.724338	2.209701	-1.27352	SOX9

tolazoline-4844	1/100	0.561955	0.561955	0.724338	0.724338	2.210322	-1.27388	GPX2
tretinoin-390	1/100	0.561955	0.561955	0.724338	0.724338	2.211196	-1.27439	PIGR
tinidazole-3813	1/100	0.561955	0.561955	0.724338	0.724338	2.211827	-1.27475	C3
tetraethylenepentamine-405	1/100	0.561955	0.561955	0.724338	0.724338	2.212764	-1.27529	SLPI
thalidomide-7288	1/100	0.561955	0.561955	0.724338	0.724338	2.21465	-1.27638	PDIA3
trichostatin A-3114	1/100	0.561955	0.561955	0.724338	0.724338	2.215439	-1.27683	GPX2
trichostatin A-3462	1/100	0.561955	0.561955	0.724338	0.724338	2.216873	-1.27766	IFNGR2
torasemide-2956	1/100	0.561955	0.561955	0.724338	0.724338	2.217596	-1.27807	IFI6
tretinoin-966	1/100	0.561955	0.561955	0.724338	0.724338	2.221525	-1.28034	ID3
tolbutamide-3804	1/100	0.561955	0.561955	0.724338	0.724338	2.22213	-1.28069	RNF43
tridihexethyl-5067	1/100	0.561955	0.561955	0.724338	0.724338	2.22494	-1.28231	TSPAN8
tolbutamide-2320	1/100	0.561955	0.561955	0.724338	0.724338	2.225566	-1.28267	BMP4
ticlopidine-4155	1/100	0.561955	0.561955	0.724338	0.724338	2.226434	-1.28317	AP1S1
trichostatin A-992	1/100	0.561955	0.561955	0.724338	0.724338	2.22709	-1.28355	IFNGR2
trichostatin A-2566	1/100	0.561955	0.561955	0.724338	0.724338	2.227249	-1.28364	OLFM4
tolbutamide-142	1/100	0.561955	0.561955	0.724338	0.724338	2.229229	-1.28478	PIGR
tomatidine-2746	1/100	0.561955	0.561955	0.724338	0.724338	2.232189	-1.28649	BMP4
trifluoperazine-910	1/100	0.561955	0.561955	0.724338	0.724338	2.232207	-1.2865	LGALS3BP
triprolidine-7248	1/100	0.561955	0.561955	0.724338	0.724338	2.234323	-1.28772	TUBA1A
trichostatin A-2137	1/100	0.561955	0.561955	0.724338	0.724338	2.234474	-1.2878	SLC12A2
tretinoin-6931	1/100	0.561955	0.561955	0.724338	0.724338	2.235327	-1.28829	IFNGR2
tubocurarine chloride-5449	1/100	0.561955	0.561955	0.724338	0.724338	2.238834	-1.29031	GPX2
tremorine-1579	1/100	0.561955	0.561955	0.724338	0.724338	2.239897	-1.29093	TUBA1A
trichostatin A-1050	1/100	0.561955	0.561955	0.724338	0.724338	2.240741	-1.29141	IFNGR2
tomatidine-7166	1/100	0.561955	0.561955	0.724338	0.724338	2.240771	-1.29143	IFNGR2
tretinoin-5767	1/100	0.561955	0.561955	0.724338	0.724338	2.240825	-1.29146	CXCL1
tretinoin-384	1/100	0.561955	0.561955	0.724338	0.724338	2.240965	-1.29154	ID3
trichostatin A-2370	1/100	0.561955	0.561955	0.724338	0.724338	2.243304	-1.29289	CD14
triamterene-1819	1/100	0.561955	0.561955	0.724338	0.724338	2.24386	-1.29321	IFI6
trichostatin A-331	1/100	0.561955	0.561955	0.724338	0.724338	2.247259	-1.29517	LCN2
trichostatin A-1672	1/100	0.561955	0.561955	0.724338	0.724338	2.247284	-1.29519	IFNGR2
trichostatin A-1306	1/100	0.561955	0.561955	0.724338	0.724338	2.247782	-1.29547	ISG15
todralazine-5512	1/100	0.561955	0.561955	0.724338	0.724338	2.248162	-1.29569	RNF43
trifluoperazine-5442	1/100	0.561955	0.561955	0.724338	0.724338	2.250125	-1.29682	GPX2
tonzonium bromide-3080	1/100	0.561955	0.561955	0.724338	0.724338	2.250443	-1.29701	IFI6
trimethobenzamide-4180	1/100	0.561955	0.561955	0.724338	0.724338	2.251667	-1.29771	AP1S1
tropine-3569	1/100	0.561955	0.561955	0.724338	0.724338	2.252304	-1.29808	BMP4

trichostatin A-1284	1/100	0.561955	0.561955	0.724338	0.724338	2.253808	-1.29894	LY6E
tretinoin-4435	1/100	0.561955	0.561955	0.724338	0.724338	2.254761	-1.29949	BMP4
tolnaftate-1501	1/100	0.561955	0.561955	0.724338	0.724338	2.255696	-1.30003	SLPI
trichostatin A-2523	1/100	0.561955	0.561955	0.724338	0.724338	2.255759	-1.30007	RCN1
trifluoperazine-5584	1/100	0.561955	0.561955	0.724338	0.724338	2.256123	-1.30028	SLPI
trichostatin A-1971	1/100	0.561955	0.561955	0.724338	0.724338	2.256185	-1.30032	CD14
trichostatin A-7005	1/100	0.561955	0.561955	0.724338	0.724338	2.256481	-1.30049	IFNGR2
trichostatin A-1785	1/100	0.561955	0.561955	0.724338	0.724338	2.259789	-1.30239	CD14
tretinoin-5571	1/100	0.561955	0.561955	0.724338	0.724338	2.260005	-1.30252	TMPRSS3
trichostatin A-3177	1/100	0.561955	0.561955	0.724338	0.724338	2.26235	-1.30387	LYZ
trimethadione-2846	1/100	0.561955	0.561955	0.724338	0.724338	2.264175	-1.30492	TFF2
tranylcypromine-2101	1/100	0.561955	0.561955	0.724338	0.724338	2.26541	-1.30563	TSPAN8
trichostatin A-4153	1/100	0.561955	0.561955	0.724338	0.724338	2.266661	-1.30635	AP1S1
trichostatin A-3332	1/100	0.561955	0.561955	0.724338	0.724338	2.267115	-1.30661	TSPAN1
trichostatin A-2881	1/100	0.561955	0.561955	0.724338	0.724338	2.267459	-1.30681	TMPRSS3
trichostatin A-873	1/100	0.561955	0.561955	0.724338	0.724338	2.269477	-1.30798	IFNGR2
trichostatin A-364	1/100	0.561955	0.561955	0.724338	0.724338	2.269481	-1.30798	IFITM3
trimethobenzamide-2002	1/100	0.561955	0.561955	0.724338	0.724338	2.269531	-1.30801	PGAM1
tridihexethyl-5486	1/100	0.561955	0.561955	0.724338	0.724338	2.269759	-1.30814	QPCT
troglitazone-1012	1/100	0.561955	0.561955	0.724338	0.724338	2.270079	-1.30832	BMP4
valproic acid-1002	1/100	0.561955	0.561955	0.724338	0.724338	2.272111	-1.30949	S100A4
trichostatin A-2904	1/100	0.561955	0.561955	0.724338	0.724338	2.27287	-1.30993	IFI6
trichostatin A-3227	1/100	0.561955	0.561955	0.724338	0.724338	2.273955	-1.31056	IFNGR2
trichostatin A-6910	1/100	0.561955	0.561955	0.724338	0.724338	2.275	-1.31116	ID1
tretinoin-6170	1/100	0.561955	0.561955	0.724338	0.724338	2.27574	-1.31159	ISG15
triamterene-1861	1/100	0.561955	0.561955	0.724338	0.724338	2.275748	-1.31159	C3
trimetazidine-1727	1/100	0.561955	0.561955	0.724338	0.724338	2.276849	-1.31222	LYZ
trihexyphenidyl-4015	1/100	0.561955	0.561955	0.724338	0.724338	2.278411	-1.31312	TGFBI
trifluoperazine-4448	1/100	0.561955	0.561955	0.724338	0.724338	2.27933	-1.31365	PSMB9
trifluoperazine-6341	1/100	0.561955	0.561955	0.724338	0.724338	2.279581	-1.3138	CDH3
troglitazone-2692	1/100	0.561955	0.561955	0.724338	0.724338	2.279707	-1.31387	TXNIP
triflupromazine-7466	1/100	0.561955	0.561955	0.724338	0.724338	2.279832	-1.31394	AP1S1
trimipramine-3342	1/100	0.561955	0.561955	0.724338	0.724338	2.283493	-1.31605	S100A4
troglitazone-6991	1/100	0.561955	0.561955	0.724338	0.724338	2.284659	-1.31673	GPX2
triamcinolone-7192	1/100	0.561955	0.561955	0.724338	0.724338	2.284661	-1.31673	LGALS3BP
troglitazone-1657	1/100	0.561955	0.561955	0.724338	0.724338	2.287053	-1.31811	S100A4
trichostatin A-5935	1/100	0.561955	0.561955	0.724338	0.724338	2.287402	-1.31831	IFNGR2

trimipramine-4083	1/100	0.561955	0.561955	0.724338	0.724338	2.287814	-1.31854	ID1
valinomycin-5911	1/100	0.561955	0.561955	0.724338	0.724338	2.288172	-1.31875	SOX9
valproic acid-5211	1/100	0.561955	0.561955	0.724338	0.724338	2.288776	-1.3191	TMPRSS3
valproic acid-1634	1/100	0.561955	0.561955	0.724338	0.724338	2.289259	-1.31938	TGFBI
troglitazone-5592	1/100	0.561955	0.561955	0.724338	0.724338	2.293094	-1.32159	BMP4
tridihexethyl-2964	1/100	0.561955	0.561955	0.724338	0.724338	2.293284	-1.3217	C3
triflusal-1717	1/100	0.561955	0.561955	0.724338	0.724338	2.293389	-1.32176	ENC1
trichostatin A-6546	1/100	0.561955	0.561955	0.724338	0.724338	2.293621	-1.32189	S100A4
trichostatin A-3077	1/100	0.561955	0.561955	0.724338	0.724338	2.299642	-1.32536	CD14
tropine-5790	1/100	0.561955	0.561955	0.724338	0.724338	2.300006	-1.32557	MMP3
TTNPB-451	1/100	0.561955	0.561955	0.724338	0.724338	2.305245	-1.32859	MMP3
valproic acid-6173	1/100	0.561955	0.561955	0.724338	0.724338	2.306537	-1.32933	CD14
valproic acid-1163	1/100	0.561955	0.561955	0.724338	0.724338	2.308046	-1.3302	IFI6
trimethobenzamide-1502	1/100	0.561955	0.561955	0.724338	0.724338	2.308115	-1.33024	RNF43
triflusal-7451	1/100	0.561955	0.561955	0.724338	0.724338	2.30852	-1.33048	AP1S1
trimethobenzamide-1920	1/100	0.561955	0.561955	0.724338	0.724338	2.309098	-1.33081	CTSD
troglitazone-1070	1/100	0.561955	0.561955	0.724338	0.724338	2.309812	-1.33122	S100A6
trichostatin A-5976	1/100	0.561955	0.561955	0.724338	0.724338	2.310057	-1.33136	IFNGR2
trifluoperazine-5221	1/100	0.561955	0.561955	0.724338	0.724338	2.310395	-1.33156	BMP4
trimethoprim-2345	1/100	0.561955	0.561955	0.724338	0.724338	2.310844	-1.33182	CTSD
tropine-6264	1/100	0.561955	0.561955	0.724338	0.724338	2.31222	-1.33261	TSPAN1
troglitazone-6949	1/100	0.561955	0.561955	0.724338	0.724338	2.313179	-1.33316	BMP4
trapidil-6515	1/100	0.561955	0.561955	0.724338	0.724338	2.314752	-1.33407	SECTM1
valproic acid-348	1/100	0.561955	0.561955	0.724338	0.724338	2.319385	-1.33674	TCN1
valinomycin-5957	1/100	0.561955	0.561955	0.724338	0.724338	2.321382	-1.33789	BMP4
valproic acid-1240	1/100	0.561955	0.561955	0.724338	0.724338	2.32142	-1.33791	CCL20
tridihexethyl-3526	1/100	0.561955	0.561955	0.724338	0.724338	2.322883	-1.33876	PDIA3
verapamil-6287	1/100	0.561955	0.561955	0.724338	0.724338	2.322926	-1.33878	CDH3
valproic acid-6929	1/100	0.561955	0.561955	0.724338	0.724338	2.326019	-1.34056	BMP4
troglitazone-370	1/100	0.561955	0.561955	0.724338	0.724338	2.327513	-1.34142	DSG2
valproic acid-1222	1/100	0.561955	0.561955	0.724338	0.724338	2.327769	-1.34157	CCL20
valdecoxib-6408	1/100	0.561955	0.561955	0.724338	0.724338	2.327827	-1.3416	MMP3
trichostatin A-7073	1/100	0.561955	0.561955	0.724338	0.724338	2.32879	-1.34216	AP1S1
velnacrine-3292	1/100	0.561955	0.561955	0.724338	0.724338	2.329649	-1.34266	TFF2
velnacrine-4997	1/100	0.561955	0.561955	0.724338	0.724338	2.329674	-1.34267	GPX2
trimetazidine-5060	1/100	0.561955	0.561955	0.724338	0.724338	2.330907	-1.34338	GPX2
xylometazoline-7020	1/100	0.561955	0.561955	0.724338	0.724338	2.331478	-1.34371	IFITM1

vigabatrin-5415	1/100	0.561955	0.561955	0.724338	0.724338	2.332132	-1.34409	IFI27
trioxysalen-6216	1/100	0.561955	0.561955	0.724338	0.724338	2.333274	-1.34474	TSPAN1
valproic acid-458	1/100	0.561955	0.561955	0.724338	0.724338	2.337116	-1.34696	SLCO1B3
valproic acid-994	1/100	0.561955	0.561955	0.724338	0.724338	2.339609	-1.3484	TSPAN1
tretinoin-1049	1/100	0.561955	0.561955	0.724338	0.724338	2.34355	-1.35067	IFNGR2
vanoxerine-4641	1/100	0.561955	0.561955	0.724338	0.724338	2.345206	-1.35162	TGFBI
valproic acid-5582	1/100	0.561955	0.561955	0.724338	0.724338	2.3455	-1.35179	SLPI
trichostatin A-6143	1/100	0.561955	0.561955	0.724338	0.724338	2.34592	-1.35203	ISG15
vincamine-2367	1/100	0.561955	0.561955	0.724338	0.724338	2.348084	-1.35328	TSPAN13
tyloxapol-3074	1/100	0.561955	0.561955	0.724338	0.724338	2.348993	-1.3538	LY6E
valproic acid-1150	1/100	0.561955	0.561955	0.724338	0.724338	2.351886	-1.35547	CD14
zomepirac-6815	1/100	0.561955	0.561955	0.724338	0.724338	2.352758	-1.35597	BMP4
valinomycin-5906	1/100	0.561955	0.561955	0.724338	0.724338	2.355146	-1.35735	DUOX2
zimeldine-2012	1/100	0.561955	0.561955	0.724338	0.724338	2.356949	-1.35839	IFITM3
velnacrine-2430	1/100	0.561955	0.561955	0.724338	0.724338	2.357502	-1.35871	IFI6
tropine-6147	1/100	0.561955	0.561955	0.724338	0.724338	2.359167	-1.35967	TSPAN13
vinpocetine-3174	1/100	0.561955	0.561955	0.724338	0.724338	2.360575	-1.36048	LYZ
tropicamide-3722	1/100	0.561955	0.561955	0.724338	0.724338	2.361081	-1.36077	C3
vincamine-3865	1/100	0.561955	0.561955	0.724338	0.724338	2.362716	-1.36171	RCN1
vancomycin-4423	1/100	0.561955	0.561955	0.724338	0.724338	2.362729	-1.36172	IFI6
valproic acid-6999	1/100	0.561955	0.561955	0.724338	0.724338	2.363023	-1.36189	TMPRSS3
vancomycin-2498	1/100	0.561955	0.561955	0.724338	0.724338	2.363633	-1.36224	CTSD
tyloxapol-5672	1/100	0.561955	0.561955	0.724338	0.724338	2.366142	-1.36369	BMP4
valproic acid-347	1/100	0.561955	0.561955	0.724338	0.724338	2.366327	-1.36379	TCN1
valproic acid-1647	1/100	0.561955	0.561955	0.724338	0.724338	2.366961	-1.36416	PLP2
W-13-643	1/100	0.561955	0.561955	0.724338	0.724338	2.367035	-1.3642	S100P
valproic acid-1209	1/100	0.561955	0.561955	0.724338	0.724338	2.36711	-1.36424	CTSD
zuclopenthixol-4261	1/100	0.561955	0.561955	0.724338	0.724338	2.368162	-1.36485	TSPAN8
tyloxapol-4611	1/100	0.561955	0.561955	0.724338	0.724338	2.37049	-1.36619	OLFM4
zoxazolamine-1270	1/100	0.561955	0.561955	0.724338	0.724338	2.371401	-1.36672	C3
valproic acid-989	1/100	0.561955	0.561955	0.724338	0.724338	2.381705	-1.37266	TSPAN1
zuclopenthixol-2936	1/100	0.561955	0.561955	0.724338	0.724338	2.384089	-1.37403	TCN1
valproic acid-1020	1/100	0.561955	0.561955	0.724338	0.724338	2.38699	-1.3757	TFF3
wortmannin-4467	1/100	0.561955	0.561955	0.724338	0.724338	2.38919	-1.37697	BMP4
valproic acid-6974	1/100	0.561955	0.561955	0.724338	0.724338	2.389969	-1.37742	LGALS3BP
valproic acid-497	1/100	0.561955	0.561955	0.724338	0.724338	2.390236	-1.37757	PRSS23
valproic acid-345	1/100	0.561955	0.561955	0.724338	0.724338	2.3905	-1.37773	CDH3

wortmannin-1081	1/100	0.561955	0.561955	0.724338	0.724338	2.391981	-1.37858	ID1
vidarabine-5850	1/100	0.561955	0.561955	0.724338	0.724338	2.392516	-1.37889	TSPAN1
zaprinast-6749	1/100	0.561955	0.561955	0.724338	0.724338	2.394446	-1.38	DUOX2
troleandomycin-1965	1/100	0.561955	0.561955	0.724338	0.724338	2.394624	-1.3801	DYNLL1
tropicamide-4280	1/100	0.561955	0.561955	0.724338	0.724338	2.395147	-1.3804	IFI6
xylometazoline-1423	1/100	0.561955	0.561955	0.724338	0.724338	2.396533	-1.3812	SLPI
tyrphostin AG-1478-1141	1/100	0.561955	0.561955	0.724338	0.724338	2.396538	-1.38121	RAN
vinpocetine-7213	1/100	0.561955	0.561955	0.724338	0.724338	2.396838	-1.38138	ENC1
wortmannin-1668	1/100	0.561955	0.561955	0.724338	0.724338	2.39873	-1.38247	BMP4
verapamil-161	1/100	0.561955	0.561955	0.724338	0.724338	2.402345	-1.38455	DEK
zidovudine-3211	1/100	0.561955	0.561955	0.724338	0.724338	2.403942	-1.38547	LYZ
zalcitabine-4215	1/100	0.561955	0.561955	0.724338	0.724338	2.406873	-1.38716	IFI6
vorinostat-6980	1/100	0.561955	0.561955	0.724338	0.724338	2.412116	-1.39018	IFNGR2
xylazine-2132	1/100	0.561955	0.561955	0.724338	0.724338	2.414677	-1.39166	C3
yohimbine-1119	1/100	0.561955	0.561955	0.724338	0.724338	2.417501	-1.39329	TSPAN13
wortmannin-7002	1/100	0.561955	0.561955	0.724338	0.724338	2.421285	-1.39547	BMP4
vinpocetine-1557	1/100	0.561955	0.561955	0.724338	0.724338	2.421953	-1.39585	IFITM3
yohimbine-1763	1/100	0.561955	0.561955	0.724338	0.724338	2.42213	-1.39595	DYNLL1
wortmannin-506	1/100	0.561955	0.561955	0.724338	0.724338	2.429636	-1.40028	ID3
zuclopenthixol-7356	1/100	0.561955	0.561955	0.724338	0.724338	2.438071	-1.40514	BMP4
Y-27632-832	1/100	0.561955	0.561955	0.724338	0.724338	2.44147	-1.4071	AP1S1
zardaverine-4793	1/100	0.561955	0.561955	0.724338	0.724338	2.476341	-1.4272	IFI6
wortmannin-1243	1/100	0.561955	0.561955	0.724338	0.724338	2.48105	-1.42991	CCL20
zardaverine-2926	1/100	0.561955	0.561955	0.724338	0.724338	2.49035	-1.43527	PLCB4

Supplementary Table 7

Co-upregulated genes (Biological Process)

Term	Overlap	P-value	Adjusted P-value	Old P-value	Old Adjusted P-value	Odds Ratio	Combined Score	Genes
cotranslational protein targeting to membrane (GO:0006613)	18/93	4.792364864807434E-16	2.4455437905112338E-12	0	0	14.127619496114903	498.34241803043886	RPL4;RPL3;RPS7;RPL34;SSR2;RPL36A;RPL6;RPS15;RPL7A;RPS17;RPL27A;RPL36;RPS3;RPL38;RPL26;RPL29;UBA52;RPL19
SRP-dependent cotranslational protein targeting to membrane (GO:0006614)	17/89	4.02098222985794E-15	1.0259536159482534E-11	0	0	13.942425982120888	462.1530822338789	RPL4;RPL3;RPS7;RPL34;RPL36A;RPL6;RPS15;RPL7A;RPS17;RPL27A;RPL36;RPS3;

								RPL38;RPL26;RPL29;UBA52; RPL19
regulation of cellular amine metabolic process (GO:0033238)	12/51	3.392150906210871E-12	8.242926702092416E-10	0	0	17.174753112924	453.57761975318425	PSMD8;PSMB6;PSMB7; PSMB4;PSMC5;PSMB5; PSMB3;PSMC1;ODC1; PSME1;PSME2;OAZ1
regulation of cellular amino acid metabolic process (GO:0006521)	12/51	3.392150906210871E-12	7.868248215633671E-10	0	0	17.174753112924	453.57761975318425	PSMD8;PSMB6;PSMB7; PSMB4;PSMC5;PSMB5; PSMB3;PSMC1;ODC1; PSME1;PSME2;OAZ1
mitochondrial electron transport, cytochrome c to oxygen (GO:0006123)	7/21	8.279073842396288E-9	7.411949792587415E-7	0	0	24.330900243309	452.7867330800124	COX8A;COX7B;COX7A2L; NDUFA4;COX4I1;COX5B; COX6A1
mitochondrial ATP synthesis coupled electron transport (GO:0042775)	16/85	3.353734190328852E-14	2.1392631966560165E-11	0	0	13.739802490339201	426.29271815471674	COX8A;COX7B;NDUFA13; NDUFB11;NDUFA4;COX4I1; NDUFA1;UQCR11;COX6A1; COX5B;COX7A2L;NDUFS6; UQCRQ;NDUFS3;UQCRC1; CYC1
respiratory electron transport chain (GO:0022904)	17/94	1.0508861667327692E-14	1.7875573696124405E-11	0	0	13.200807578816587	424.8885525169374	COX8A;COX7B;NDUFA13; NDUFB11;NDUFA4;COX4I1; NDUFA1;UQCR11;ETFB; COX6A1;COX5B;COX7A2L; NDUFS6;UQCRQ;NDUFS3; UQCRC1;CYC1
protein targeting to ER (GO:0045047)	17/97	1.8179685571837578E-14	1.8554187094617432E-11	0	0	12.792535179471741	404.7362609880498	RPL4;RPL3;RPS7;RPL34; RPL36A;RPL6;RPS15;RPL7A; RPS17;RPL27A;RPL36;RPS3; RPL38;RPL26;RPL29;UBA52; RPL19
negative regulation of ubiquitin protein ligase activity (GO:1904667)	14/77	2.2875975288399996E-12	6.144005362984483E-10	0	0	13.271400132714001	355.720225124239	RPS7;UBE2C;PSMD8;PSMB6; PSMB7;PSMB4;PSMC5; PSMB5;UBB;PSMB3;PSMC1; PSME1;PSME2;UBA52
regulation of cellular ketone metabolic process (GO:0010565)	12/61	3.296385306069602E-11	5.256704442772869E-9	0	0	14.359219815723344	346.56852243541454	PSMD8;PSMB6;PSMB7; PSMB4;PSMC5;PSMB5; PSMB3;PSMC1;ODC1; PSME1;PSME2;OAZ1

Co-upregulated genes (Cellular Component)

Term	Overlap	P-value	Adjusted P-value	Old P-value	Old Adjusted P-value	Odds Ratio	Combined Score	Genes
methylosome (GO:0034709)	6/12	5.397756519747526E-9	2.0061661731728305E-7	0	0	36.496350364963504	694.7913295258877	SNRPD2;PRMT1;SNRPG;ERH; SNRPF;SNRPB
cytosolic part (GO:0044445)	22/159	4.281221960525418E-16	9.547124971971682E-14	0	0	10.099618968920717	357.3964588503926	RPL4;CCT3;RPL3;RPS7;RPL34; RPL36A;RPL6;RPS15;RPL7A;

								RPS17;PSMC5;RPL27A;PSMC1;RPL36;NHP2;RPS3;RPL38;RPL26;RPL29;CCT7;CCT5;RPL19
cytosolic large ribosomal subunit (GO:0022625)	13/69	8.789620552539858E-12	7.840341532865554E-10	0	0	13.752247963609436	350.0971590523819	RPL4;RPL3;RPL34;RPL36A;RPL6;RPL7A;RPL27A;RPL36;NHP2;RPL38;RPL26;RPL29;RPL19
large ribosomal subunit (GO:0015934)	13/72	1.554649886195744E-11	9.905340703475739E-10	0	0	13.179237631792375	327.9941337499377	RPL4;RPL3;RPL34;RPL36A;RPL6;RPL7A;RPL27A;RPL36;NHP2;RPL38;RPL26;RPL29;RPL19
DNA-directed RNA polymerase complex (GO:0000428)	5/16	1.7961559736478113E-6	5.340570428312825E-5	0	0	22.81021897810219	301.7760434954286	POLR1D;POLR2F;POLR2G;POLR2J;POLR2L
U7 snRNP (GO:0005683)	3/7	8.54627820496677E-5	0.0015246560317660719	0	0	31.282586027111574	293.0374215158226	SNRPG;SNRPF;SNRPB
mitochondrial respiratory chain complex IV (GO:0005751)	5/17	2.516126824602767E-6	6.2344031320713E-5	0	0	21.468441391155004	276.78810247234173	COX8A;NDUFA4;COX4I1;COX5B;COX6A1
cytosolic ribosome (GO:0022626)	17/124	1.1940463740291881E-12	1.775148942723393E-10	0	0	10.007063809748056	274.7306601497309	RPL4;RPL3;RPS7;RPL34;RPL36A;RPL6;RPS15;RPL7A;RPS17;RPL27A;RPL36;NHP2;RPS3;RPL38;RPL26;RPL29;RPL19
focal adhesion (GO:0005925)	33/356	3.820886748176853E-18	1.7041154896868764E-15	0	0	6.766177314852785	271.36463991450444	RPL4;CYFIP1;RPL3;HSPB1;RPL6;ACTG1;RPS15;PDLIM1;RPL7A;RPS17;PLAU;CFL1;RPS3;CAPN2;FLNA;RPL38;MISP;RPL19;CAP1;RPS7;YWHAZ;RHOB;GNB2;FAT1;LAP3;P4HB;PFN1;EZR;ATP6V0C;PPIA;EPHA2;HSPA1A;PLEC
U4 snRNP (GO:0005687)	4/12	1.564449263581597E-5	3.3225922455113914E-4	0	0	24.330900243309	269.23093944445066	SNRPD2;SNRPG;SNRPF;SNRPB

Co-upregulated genes (Molecular Function)

Term	Overlap	P-value	Adjusted P-value	Old P-value	Old Adjusted P-value	Odds Ratio	Combined Score	Genes
rRNA methyltransferase activity (GO:0008649)	3/8	1.3535474375265513E-4	0.01731036778436734	0	0	27.37226277372263	243.82148251299836	TRMT112;FBL;EMG1
oxidoreductase activity, acting on diphenols and related substances as donors, cytochrome as acceptor (GO:0016681)	3/8	1.3535474375265513E-4	0.015579331005930605	0	0	27.37226277372263	243.82148251299836	UQCRQ;UQCRC1;UQCR11
ubiquinol-cytochrome-c reductase activity (GO:0008121)	3/8	1.3535474375265513E-4	0.014163028187209641	0	0	27.37226277372263	243.82148251299836	UQCRQ;UQCRC1;UQCR11

RNA binding (GO:0003723)	75/1387	9.994990493203466E-26	1.150423405767719E-22	0	0	3.946973723680263	227.2080491992189	RPL4;RPL3;RPL34;HSPB1;YBX1;RPL6;RPS15;FBL;RPL7A;MRPL3;NGRN;RPS17;ZFP36;LGALS1;SNRPD2;C1QBP;RPL36;RPL38;HMGN2;LRRC47;CCT3;HSP90AA1;RPS7;PRMT1;PABPC4;MRPS21;HLA-A;ILF2;YWHAZ;NME1;PKM;KARS;OAS1;NHP2;SNRPG;S100A4;ERH;RPL26;SNRPF;PFN1;EZR;RPL29;ALDOA;SLC25A5;PPIA;SRSF9;SNRPB;PLEC;RPL36A;U2AF1;HSD17B10;EMG1;PUF60;SERPINH1;RPS3;FLNA;POLR2G;RPL19;JUN;XRCC5;TUBB4B;HSPE1;EIF1;LSM7;KRT18;RPL27A;PSMC1;UBE2N;FAU;P4HB;SLIRP;EIF3D;NOP10;APOBEC3B;HSPA1A
cadherin binding (GO:0045296)	26/313	2.181615942542665E-13	1.2555199749333037E-10	0	0	6.063291434434831	176.76641147954444	RPL34;NDRG1;RPL6;PSMB6;PDLIM1;RPL7A;DNAJB1;LDHA;PUF60;FLNA;SFN;S100A11;YWHAZ;GPRC5A;KRT18;PKM;PLIN3;TAGLN2;S100P;PFN1;EZR;RPL29;ALDOA;EPHA2;HSPA1A;PLEC
RNA polymerase II activity (GO:0001055)	3/11	3.868251748101178E-4	0.031802555443317546	0	0	19.907100199071	156.42079054436408	POLR2F;POLR2J;POLR2L
proton-transporting ATP synthase activity, rotational mechanism (GO:0046933)	3/11	3.868251748101178E-4	0.029682385080429707	0	0	19.907100199071	156.42079054436408	ATP5H;ATP6V0C;ATP5L
proteasome-activating ATPase activity (GO:0036402)	2/6	0.0027049624712037644	0.11119327872698333	0	0	24.330900243309	143.86051680393575	PSMC5;PSMC1
RNA polymerase I activity (GO:0001054)	3/12	5.105529251083533E-4	0.034567436282336154	0	0	18.248175182481752	138.3214644644389	POLR1D;POLR2F;POLR2L
hydrogen ion transmembrane transporter activity (GO:0015078)	7/51	5.8029341219940986E-6	0.0013358354348830415	0	0	10.018605982539002	120.79580391666858	UQCRQ;UQCRC1;UQCR11;ATP5H;ATP6V0C;ATP5L;ATP6V1F

Co-downregulated genes (Biological Process)

Term	Overlap	P-value	Adjusted P-value	Old P-value	Old Adjusted P-value	Odds Ratio	Combined Score	Genes
SRP-dependent cotranslational protein targeting to membrane (GO:0006614)	47/89	4.030077843800058E-75	2.0565487236911697E-71	0	0	61.0508540624797	10458.017179043958	RPL4;RPL5;RPL30;RPL3;RPL31;RPLP1;RPLP0;RPL11;RPL36A;SRP14;RPL10A;RPL8;RPL9;RPL6;RPL7;RPL7A;RPS16;RPS15A;RPS19;RPS18;RPL14;RPS3;RPL13;RPS2;RPS11;RPL39;RPS10;RPL17;RPS13;RPL19;RPL41;RPL21;

								RPS7;RPS8;RPL23;RPS5;RPS6;RPL13A;RPS3A;RPL23A;RPS25;RPS27;RPS29;RPL24;RPS20;RPS24;RPS23
cotranslational protein targeting to membrane (GO:0006613)	47/93	7.036894363056097E-74	1.795463596733763E-70	0	0	58.42501087699671	9841.116424470907	RPL4;RPL5;RPL30;RPL3;RPL31;RPLP1;RPLP0;RPL11;RPL36A;SRP14;RPL10A;RPL8;RPL9;RPL6;RPL7;RPL7A;RPS16;RPS15A;RPS19;RPS18;RPL14;RPS3;RPL13;RPS2;RPS11;RPL39;RPS10;RPL17;RPS13;RPL19;RPL41;RPL21;RPS7;RPS8;RPL23;RPS5;RPS6;RPL13A;RPS3A;RPL23A;RPS25;RPS27;RPS29;RPL24;RPS20;RPS24;RPS23
protein targeting to ER (GO:0045047)	47/97	1.0331357440862874E-72	1.757363900690775E-69	0	0	56.015732077945295	9284.805221384107	RPL4;RPL5;RPL30;RPL3;RPL31;RPLP1;RPLP0;RPL11;RPL36A;SRP14;RPL8;RPL10A;RPL9;RPL6;RPL7;RPL7A;RPS16;RPS15A;RPS19;RPS18;RPL14;RPS3;RPL13;RPS2;RPS11;RPL39;RPS10;RPL17;RPS13;RPL19;RPL41;RPL21;RPS7;RPS8;RPL23;RPS5;RPS6;RPL13A;RPS3A;RPL23A;RPS25;RPS27;RPS29;RPL24;RPS20;RPS24;RPS23
nuclear-transcribed mRNA catabolic process, nonsense-mediated decay (GO:0000184)	48/112	6.151890324232557E-71	7.848274081139685E-68	0	0	49.5458298926507	8009.914890083896	RPL4;RPL5;RPL30;RPL3;RPL31;RPLP1;RPLP0;RPL11;RPL36A;RPL8;RPL10A;RPL9;RPL6;RPL7;RPL7A;RPS16;RPS15A;RPS19;RPS18;RPL14;RPS3;RPL13;RPS2;RPS11;RPL39;RPS10;RPL17;RPS13;RPL19;RPL41;RPL21;RPS7;RPS8;RPL23;RPS5;RPS6;RPL13A;RPS3A;RPL23A;RPS25;RPS27;RPS29;RPL24;RPS20;EIF3E;PABPC1;RPS24;RPS23
viral gene expression (GO:0019080)	46/110	2.7646666317041517E-67	2.821618764317257E-64	0	0	48.344718864950075	7409.1318274558	RPL4;RPL5;RPL30;RPL3;RPL31;RPLP1;RPLP0;RPL11;RPL36A;RPL8;RPL10A;RPL9;RPL6;RPL7;RPL7A;RPS16;RPS15A;RPS19;RPS18;RPL14;RPS3;RPL13;RPS2;RPS11;RPL39;RPS10;RPL17;RPS13;RPL19;RPL41;RPL21;RPS7;RPS8;RPL23;RPS5;RPS6;RPL13A;RPS3A;RPL23A;RPS25;RPS27;RPS29;RPL24;RPS20;RPS24;RPS23
viral transcription (GO:0019083)	46/113	1.3263652031639266E-66	1.1280736052909196E-63	0	0	47.06123075349123	7138.632152911588	RPL4;RPL5;RPL30;RPL3;RPL31;RPLP1;RPLP0;RPL11;RPL36A;RPL8;RPL10A;RPL9;RPL6;RPL7;RPL7A;RPS16;RPS15A;RPS19;RPS18;RPL14;RPS3;RPL13;RPS2;RPS11;RPL39;RPS10;RPL17;RPS13;RPL19;RPL41;RPL21;RPS7;RPS8;RPL23;RPS5;RPS6;RPL13A;RPS3A;RPL23A;RPS25;RPS27;RPS29;RPL24;RPS20;RPS24;RPS23
nuclear-transcribed mRNA catabolic process (GO:0000956)	50/174	1.875949765246553E-63	1.367567378864737E-60	0	0	33.22038402763936	4798.14450156091	RPL4;EIF4A2;RPL5;RPL30;RPL3;RPL31;RPLP1;RPLP0;RPL8;RPL10A;RPL9;RPL6;RPL7;RPL7A;RPS16;RPS19;RPS18;RPS11;RPL39;RPS10;RPS13;RPS7;RPL21;RPS8;RPS5;RPL23;RPS6;RPL13A;RPS3A;RPL24;PABPC1;RPL11;RPL36A;RPS15A;RPL14;RPS3;RPL13;RPS2;RPL17;EIF4B;RPL19;RPL41;RPL23A;RPS25;RPS27;RPS29;RPS20;EIF3E;RPS24;RPS23
cytoplasmic translation (GO:0002181)	24/54	6.602954365395516E-36	1.8719375625896288E-33	0	0	51.380860629415544	4162.134677856887	RPL4;RPL30;RPL41;RPS7;RPL31;RPLP1;RPLP0;RPL11;RPL36A;RPS3A;RPL10A;RPL8;RPL9;RPL6;RPL7;RPS29;RPS3;RPL24;RPS20;RPL39;RPL17;EIF4B;RPL19;RPS23
peptide biosynthetic process (GO:0043043)	47/174	4.33360130601095E-58	2.7642959330717347E-55	0	0	31.227160985980998	4124.593857474888	RPL4;RPL5;RPL30;RPL3;RPL31;RPLP1;RPLP0;RPL11;RPL36A;RPL8;RPL10A;RPL9;RPL6;RPL7;RPL7A;RPS16;RPS15A;RPS19;RPS18;RPL14;RPS3;RPL13;RPS2;RPS11;RPL39;RPS10;RPL17;RPS13;RPL19;RPL41;RPS7;RPL21;RPS8;RPS5;RPL23;RPS6;RPL13A;RPS3A;RPL23A;EEF1A1;RPS25;RPS27;RPS29;RPL24;RPS20;RPS24;RPS23

translation (GO:0006412)	51/232	4.335543823841741E-58	2.458253348118267E-55	0	0	25.41359378114411	3356.70595247571	RPL4;RPL5;RPL30;RPL3;RPL31;RPLP1;RPLP0;RPL8;RPL10A RPS25;RPS27;QARS;RPS29;RPS20;RPS24;RPS23

Co-downregulated genes (Cellular Component)

Term	Overlap	P-value	Adjusted P-value	Old P-value	Old Adjusted P-value	Odds Ratio	Combined Score	Genes
cytosolic ribosome (GO:0022626)	47/124	2.8712310173601983E-66	1.2805690337426485E-63	0	0	43.81875815774753	6612.946240791307	RPL4;RPL5;RPL30;RPL3;RPL31;RPLP1;RPLP0;RPL11; RPL36A;RPS27L;RPL8;RPL10A;RPL9;RPL6;RPL7;RPL7A; RPS16;RPS15A;RPS19;RPS18;RPL14;RPS3;RPL13;RPS2; RPS11;RPL39;RPS10;RPL17;RPS13;RPL19;RPL41; RPL21;RPS7;RPS8;RPL23;RPS5; RPS6;RPL13A;RPS3A;RPL23A;RPS25;RPS27;RPS29; RPL24;RPS20;RPS24;RPS23
cytosolic part (GO:0044445)	47/159	3.327622545209981E-60	7.420598275818259E-58	0	0	34.17311956956411	4680.106032361233	RPL4;RPL5;RPL30;RPL3;RPL31;RPLP1;RPLP0;RPL11; RPL36A;RPS27L;RPL8;RPL10A;RPL9;RPL6;RPL7;RPL7A; RPS16;RPS15A;RPS19;RPS18;RPL14;RPS3;RPL13;RPS2;
ribosome (GO:0005840)	28/76	6.166385724791565E-39	9.167360110856794E-37	0	0	42.59202920596288	3747.319380576326	RPL30;RPL31;RPL11;RPL36A;RPL10A;RPL8;RPL9;RPL6; RPL7A;RPS19;RPS18;RPS3;RPL13;RPS11;RPL39;RPS10; RPS13;RPL19;RPL41;RPS7;RPL23;RPS5;RPL13A;RPS25; RPS27;RPS29;RPL24;RPS23
cytosolic large ribosomal subunit (GO:0022625)	26/69	1.669116379559417E-36	1.8610647632087502E-34	0	0	43.56203401189579	3588.6738600335275	RPL4;RPL5;RPL30;RPL3;RPL31;RPLP1;RPLP0;RPL11; RPL36A;RPL10A;RPL8;RPL9;RPL6;RPL7;RPL7A;RPL14; RPL13;RPL39;RPL17;RPL19;RPL41;RPL21;RPL23; RPL13A;RPL23A;RPL24
cytosolic small ribosomal subunit (GO:0022627)	21/49	4.310278878129821E-31	2.7462633994941426E-29	0	0	49.5458298926507	3464.2015826205047	RPS7;RPS8;RPS5;RPS6;RPS27L;RPS3A;RPS25;RPS16; RPS15A;RPS27;RPS19;RPS18;RPS29;RPS3;RPS20;RPS2 RPS11;RPS10;RPS13;RPS24;RPS23
large ribosomal subunit (GO:0015934)	26/72	6.419716739579755E-36	5.726387331705142E-34	0	0	41.74694926140013	3382.9093178678013	RPL4;RPL5;RPL30;RPL3;RPL31;RPLP1;RPLP0;RPL11; RPL36A;RPL10A;RPL8;RPL9;RPL6;RPL7;RPL7A;RPL14; RPL13;RPL39;RPL17;RPL19;RPL41;RPL21;RPL23; RPL13A;RPL23A;RPL24
small ribosomal subunit (GO:0015935)	21/53	3.409406377026717E-30	1.9007440551923948E-28	0	0	45.80652197622424	3108.018991231179	RPS7;RPS8;RPS5;RPS6;RPS27L;RPS3A;RPS25;RPS16; RPS15A;RPS27;RPS19;RPS18;RPS29;RPS3;RPS20; RPS2;RPS11;RPS10;RPS13;RPS24;RPS23
polysomal ribosome (GO:0042788)	14/28	2.7737504710980747E-22	1.3745474556774904E-20	0	0	57.80346820809249	2869.171773454496	RPL30;RPL41;RPL31;RPL11;RPL36A;RPL10A;RPL8; RPL6;RPL7A;RPS29;RPL24;RPL39;RPL19;RPS23
polysome (GO:0005844)	16/63	1.2479291866109934E-19	5.565764172285031E-18	0	0	29.360491788237454	1277.9926595848308	RPL30;RPL41;RPL31;RPL11;RPL36A;RPL10A;RPL8; RPL6;RPL7A;RPS29;RPS3;RPL24;RPL39;EIF4B;RPL19; RPS23
focal adhesion (GO:0005925)	42/356	1.120950787677404E-35	8.332400855068703E-34	0	0	13.639020588426316	1097.6179263836373	RPL4;RPL5;RPL30;RPL3;RPL31;RPLP1;RPLP0;GDI2; RPL8;RPL10A;RPL9;ACTB;RPL6;RPL7;HSP90B1; PPP1CC;RPL7A;LASP1;RPS16;RPS19;YWHAQ;RPS18; RPS3;RPS2;RPS11;RPS10;RPS13;RPL19;HSPA8; ANXA1;RPS7;RPS8;RPS5;RPL23;RPL13A;RPS3A; YWHAZ;RPS29;FAT1;PABPC1;PPIB;CD46

Co-downregulated genes (Cellular Component)

Term	Overlap	P-value	Adjusted P-value	Old P-value	Old Adjusted P-value	Odds Ratio	Combined Score	Genes
ubiquitin-protein transferase inhibitor activity (GO:0055105)	4/6	8.001540621063451E-8	1.8419546509688064E-5	0	0	77.07129094412332	1259.425560168038	RPL5;RPS7;RPL23;RPL11
RNA binding (GO:0003723)	82/1387	1.2147200175015294E-48	1.3981427401442603E-45	0	0	6.834728757121246	754.0726906612813	RPL4;EIF4A2;TOP2A;RPL5;RPL30;RPL3;HSP90AB1;RPL31;RPLP0;GDI2;ATP5C1;HMGB1;RPL8;RPL10A;SRP14;RPL9;RPL6;RPL7;LGALS3;PPP1CC;RPL7A;RPS16;LGALS1;RPS19;RPS18;RPS11;RPL39;RPS10;LBR;RPS13;HSP90AA1;RPS7;RPL21;RPS8;RPS5;RPL23;RPS6;RPL13A;RPS3A;YWHAZ;EEF1A1;HADHB;NCL;RPL24;PABPC1;PPIB;KPNB1;ATP5A1;RPL11;RPL36A;RPS27L;RTN4;HSP90B1;HSPD1;RPS15A;UBC;RPL14;RPS3;RPL13;LTA4H;RPS2;HNRNPA1;RPL17;EIF4B;RPL19;HSPA8;RPL41;RPL23A;NAP1L1;EEF2;ASS1;RPS25;H1F0;RPS27;KRT18;EIF3L;EIF3H;RPS20;EIF3E;RPS24;TPT1;RPS23
DNA polymerase binding (GO:0070182)	5/16	1.847846715323945E-7	3.0383879561969438E-5	0	0	36.127167630057805	560.118303014174	HSP90AA1;PCNA;HSP90AB1;PTGES3;HMGB1
mRNA 5'-UTR binding (GO:0048027)	6/22	2.5540267450414112E-8	9.798949278475547E-6	0	0	31.529164477141354	551.2246825403585	RPL5;RPL41;RPS7;NCL;RPS3A;RPS13
small ribosomal subunit rRNA binding (GO:0070181)	2/7	0.0015182716250434199	0.09197529686447244	0	0	33.03055326176713	214.37432468661018	RPS3;RPS13
rRNA binding (GO:0019843)	6/43	1.7954505276210418E-6	2.2961817303242434E-4	0	0	16.131200430165347	213.41988832690478	RPL5;RPL23;RPS5;RPL11;RPS3;RPS13
disordered domain specific binding (GO:0097718)	4/23	4.210552069859518E-5	0.004405768574916641	0	0	20.105554159336517	202.5701270246767	HSP90AA1;HSP90AB1;CALM2;GAPDH
phospholipase inhibitor activity (GO:0004859)	2/8	0.0020128647561129787	0.11032415877552565	0	0	28.901734104046245	179.427639301184	ANXA1;ANXA4
protein phosphatase activator activity (GO:0072542)	2/8	0.0020128647561129787	0.10530942428572904	0	0	28.901734104046245	179.427639301184	IGFBP3;CALM2
MHC class II protein complex binding (GO:0023026)	3/16	3.2786274064216056E-4	0.02515800096527512	0	0	21.676300578034684	173.90712812463758	HSPA8;HSP90AA1;HSP90AB1

Supplementary Table 8

Term	Overlap	P-value	Adjusted P-value	Old P-value	Old Adjusted P-value	Odds Ratio	Combined Score	Genes

Parkinson disease	23/142	2.41E-18	3.71E-16	0	0	11.82276139	479.6093921	COX8A;NDUFA13;COX7B;NDUFB11;NDUFA4;COX4I1;NDUFA1;UQCR11;COX6A1;COX5B;COX7A2L;UBB;UQCRQ;NDUFS6;UQCRC1;PPIF;NDUFS3;VDAC2;VDAC1;COX1;CYC1;SLC25A5;ND2
Proteasome	16742	1.75E-11	7.72E-10	0	0	17.84266018	441.8971384	PSMD8;PSMB6;PSMB7;PSMB4;PSMC5;PSMB5;PSMB3;PSMC1;ADRM1;PSME1;PSME2
Huntington disease	26/193	1.61E-18	4.97E-16	0	0	9.83321357	402.8444259	COX7B;NDUFA13;NDUFB11;COX4I1;UQCR11;COX5B;COX6A1;POLR2F;COX1;POLR2G;CYC1;POLR2J;POLR2L;COX8A;NDUFA4;NDUFA1;SOD1;COX7A2L;UQCRQ;NDUFS6;UQCRC1;PPIF;NDUFS3;VDAC2;VDAC1;SLC25A5
Oxidative phosphorylation	20/133	1.86E-15	1.91E-13	0	0	10.97634597	372.3099687	COX8A;NDUFA13;COX7B;NDUFB11;NDUFA4;COX4I1;NDUFA1;UQCR11;COX6A1;COX5B;COX7A2L;UQCRQ;NDUFS6;UQCRC1;NDUFS3;COX1;CYC1;ND2;ATP6V0C;ATP6V1F
Non-alcoholic fatty liver disease (NAFLD)	20/149	1.76E-14	1.35E-12	0	0	9.79767795	310.32531	COX8A;NDUFA13;JUN;COX7B;CXCL8;NDUFB11;NDUFA4;COX4I1;NDUFA1;UQCR11;COX6A1;COX5B;COX7A2L;CASP7;UQCRQ;NDUFS6;UQCRC1;NDUFS3;COX1;CYC1
Ribosome	20/153	2.95E-14	1.81E-12	0	0	9.541529507	297.2742216	RPL4;RPL3;RPS7;RPL34;RPL36A;MRPS21;RPL6;RPS15;MRPL3;RPL7A;RPS17;RPL27A;RPL36;RPS3;RPL38;RPL26;FAU;RPL29;UBA52;RPL19
Alzheimer disease	20/171	2.52E-13	1.29E-11	0	0	8.53715798	247.6523864	COX8A;NDUFA13;COX7B;NDUFB11;NDUFA4;COX4I1;NDUFA1;UQCR11;COX6A1;COX5B;HSD17B10;COX7A2L;CASP7;UQCRQ;NDUFS6;CAPN2;UQCRC1;NDUFS3;COX1;CYC1
Cardiac muscle contraction	28825	6.68E-10	2.29E-08	0	0	11.22964627	237.2378415	COX8A;COX7B;COX7A2L;UQCRQ;COX4I1;UQCRC1;UQCR11;COX1;CYC1;COX6A1;ATP1B1;COX5B
Glutathione metabolism	20668	9.01E-07	2.77E-05	0	0	10.42752868	145.1515481	RRM2;GPX4;GSTO1;GSTP1;ODC1;SMS;LAP3;GCLM
Thermogenesis	19/231	4.92E-10	1.90E-08	0	0	6.003728631	128.6706831	COX8A;COX7B;NDUFA13;NDUFB11;NDUFA4;COX4I1;NDUFA1;UQCR11;COX5B;COX6A1;ACTG1;COX7A2L;UQCRQ;NDUFS6;UQCRC1;NDUFS3;COX1;CYC1;ND2
Mineral absorption	18810	5.80E-06	1.49E-04	0	0	10.01860598	120.7958039	MT2A;FTH1;MT1X;MT1H;ATP1B1;MT1HL1;MT1E
RNA polymerase	11444	5.91E-05	0.001212539	0	0	11.77301625	114.6348998	POLR1D;POLR2F;POLR2G;POLR2J;POLR2L
Glycolysis / Gluconeogenesis	25020	3.99E-05	8.77E-04	0	0	7.513954487	76.11830424	ALDH1A3;LDHA;TPI1;PKM;PGAM1;PGK1;ALDOA
Pathogenic Escherichia coli infection	20241	1.03E-04	0.001872244	0	0	7.96284008	73.07899501	KRT18;EZR;TUBB4B;YWHAZ;TUBA4A;ACTG1
Epstein-Barr virus infection	13/201	4.31E-06	1.21E-04	0	0	4.720920943	58.32263235	JUN;ADRM1;HLA-B;ISG15;HLA-A;PSMD8;CCNA2;PSMC5;IRAK1;OAS1;CDK4;PSMC1;IRF7
Kaposi sarcoma-associated herpesvirus infection	12/186	1.04E-05	2.45E-04	0	0	4.709206499	54.051738	ZFP36;JUN;CXCL8;UBB;GNG5;CDK4;GNB2;HLA-B;IRF7;HLA-A;FOS;GABARAP
Salmonella infection	31594	1.79E-04	0.003066279	0	0	5.941266338	51.2554139	JUN;CXCL8;FLNA;FOS;PFN1;ACTG1;PFN2
Spliceosome	9/134	9.78E-05	0.0018835	0	0	4.902494825	45.26049153	LSM7;SNRPD2;PUF60;SNRPG;U2AF1;SNRPF;SNRPB;SRSF9;HSPA1A
Ferroptosis	14702	0.002137077	0.021232891	0	0	7.299270073	44.87822216	GPX4;FTH1;VDAC2;GCLM
Pyrimidine metabolism	20941	0.001089171	0.01242462	0	0	6.402868485	43.68253428	PNP;RRM2;UPP1;TYMS;NME1
Pertussis	27912	6.13E-04	0.007555842	0	0	5.762581637	42.62384655	CASP7;JUN;CXCL8;IRAK1;CFL1;FOS
Antigen processing and presentation	28277	6.58E-04	0.007790064	0	0	5.687742914	41.67357724	HSP90AA1;HLA-B;PSME1;PSME2;HLA-A;HSPA1A

Retrograde endocannabinoid signaling	9/148	2.08E-04	0.003374309	0	0	4.438745315	37.62824473	NDUFA13;GNG5;NDUFB11;NDUFS6;NDUFA4;GNB2;NDUFS3;NDUFA1;ND2
Pentose phosphate pathway	11018	0.007856264	0.060493234	0	0	7.299270073	35.37550429	TALDO1;ALDOA;TKT
Shigellosis	23863	0.001966305	0.020187394	0	0	5.614823133	34.98932803	CXCL8;U2AF1;PFN1;ACTG1;PFN2
Phenylalanine metabolism	42767	0.022209392	0.134127306	0	0	8.587376556	32.6942038	ALDH1A3;MIF
Cellular senescence	9/160	3.71E-04	0.005445001	0	0	4.105839416	32.43052551	CCNA2;CXCL8;CDK4;CAPN2;HLA-B;VDAC2;VDAC1;HLA-A;SLC25A5
Arginine and proline metabolism	17989	0.004500892	0.03648091	0	0	5.958587815	32.19710879	AOC1;ODC1;SMS;LAP3
Necroptosis	9/162	4.07E-04	0.005692857	0	0	4.055150041	31.6609907	HSP90AA1;PARP4;FTH1;CAPN2;CHMP2A;VDAC2;VDAC1;SLC25A5;PPIA
Human T-cell leukemia virus 1 infection	11/219	2.29E-04	0.003522875	0	0	3.666300037	30.73402658	FOSL1;CCNA2;JUN;ZFP36;CDK4;HLA-B;VDAC2;VDAC1;HLA-A;FOS;SLC25A5
Protein processing in endoplasmic reticulum	9/165	4.65E-04	0.006225339	0	0	3.98142004	30.55235385	BCAP31;DNAJB1;HSP90AA1;HSPH1;SSR4;SSR2;CAPN2;P4HB;HSPA1A
Influenza A	9/171	6.02E-04	0.007725858	0	0	3.841721091	28.487239	DNAJB1;JUN;CXCL8;OAS1;IRF7;VDAC1;PRSS3;ACTG1;HSPA1A
Phagosome	8/152	0.001201916	0.013221075	0	0	3.841721091	25.83111191	LAMP1;HLA-B;HLA-A;TUBB4B;ATP6V0C;TUBA4A;ACTG1;ATP6V1F
Drug metabolism	6/108	0.0037354	0.031094679	0	0	4.055150041	22.66788501	ALDH1A3;RRM2;GSTO1;GSTP1;UPP1;NME1
Measles	7/138	0.002964186	0.027665738	0	0	3.702528298	21.55298274	JUN;IRAK1;OAS1;CDK4;IRF7;FOS;HSPA1A
Fluid shear stress and atherosclerosis	7/139	0.003085733	0.027953115	0	0	3.675891404	21.25020281	JUN;HSP90AA1;SDC4;GSTO1;GSTP1;FOS;ACTG1
Cytosolic DNA-sensing pathway	23102	0.0109069	0.076348301	0	0	4.634457189	20.94014438	IRF7;POLR1D;POLR2F;POLR2L
Histidine metabolism	44958	0.039160905	0.204433197	0	0	6.347191368	20.5653847	ALDH1A3;AOC1
Viral carcinogenesis	9/201	0.001870364	0.019864551	0	0	3.268329883	20.53041439	CCNA2;JUN;PKM;CDK4;PSMC1;HLA-B;IRF7;HLA-A;YWHAZ
Apoptosis	7/143	0.003610437	0.030889291	0	0	3.573069266	20.0946792	JUN;CASP7;PARP4;CAPN2;FOS;TUBA4A;ACTG1
Mitophagy	23833	0.012137605	0.081269184	0	0	4.491858506	19.81559464	FIS1;JUN;UBB;GABARAP
Rheumatoid arthritis	33359	0.008313027	0.062449083	0	0	4.010587952	19.21044134	JUN;CXCL8;FOS;ATP6V0C;ATP6V1F
NOD-like receptor signaling pathway	8/178	0.003228528	0.028411049	0	0	3.280570819	18.81646481	JUN;HSP90AA1;CXCL8;OAS1;IRF7;VDAC2;VDAC1;GABARAP
IL-17 signaling pathway	34090	0.009088784	0.06665108	0	0	3.924338749	18.44719484	FOSL1;HSP90AA1;JUN;CXCL8;FOS
Human immunodeficiency virus 1 infection	9/212	0.002680312	0.025798006	0	0	3.098746729	18.35022644	JUN;IRAK1;GNG5;GNB2;CFL1;HLA-B;HLA-A;FOS;APOBEC3B
Epithelial cell signaling in Helicobacter pylori infection	24929	0.014144737	0.090762062	0	0	4.293688278	18.28429656	JUN;CXCL8;ATP6V0C;ATP6V1F
Sulfur metabolism	44075	0.116776526	0.404125506	0	0	8.110300081	17.41681431	PAPSS1
Cysteine and methionine metabolism	17227	0.026483314	0.153903035	0	0	4.659108557	16.91834317	LDHA;SMS;GCLM
Collecting duct acid secretion	46419	0.052434857	0.256348188	0	0	5.406866721	15.94043637	ATP6V0C;ATP6V1F

Ribosome biogenesis in eukaryotes	5/101	0.012691032	0.08316676	0	0	3.613500036	15.77964773	POP5;FBL;EMG1;NHP2;NOP10
Hepatitis B	7/163	0.007313618	0.057758826	0	0	3.13465586	15.41629159	CCNA2;JUN;CXCL8;IRAK1;IRF7;FOS;YWHAZ
Vibrio cholerae infection	18323	0.031075054	0.167914325	0	0	4.379562044	15.20299234	ATP6V0C;ACTG1;ATP6V1F
Toll-like receptor signaling pathway	5/104	0.014259594	0.089631736	0	0	3.509264458	14.91551556	JUN;CXCL8;IRAK1;IRF7;FOS
Estrogen signaling pathway	6/137	0.011580667	0.07926323	0	0	3.196760616	14.25249582	JUN;HSP90AA1;KRT18;FOS;KRT20;HSPA1A
Tight junction	7/170	0.009112295	0.065269463	0	0	3.005581795	14.12061596	JUN;CDK4;CLDN7;EZR;MYL9;TUBA4A;ACTG1
Legionellosis	20149	0.039591511	0.203236425	0	0	3.98142004	12.85656488	CASP7;CXCL8;HSPA1A
beta-Alanine metabolism	11355	0.06705332	0.299310472	0	0	4.709206499	12.72553405	ALDH1A3;SMS
Fructose and mannose metabolism	12086	0.074809406	0.320018014	0	0	4.423800044	11.47008031	TPI1;ALDOA
Viral myocarditis	21610	0.047166685	0.2381531	0	0	3.711493257	11.33515075	HLA-B;HLA-A;ACTG1
Lysosome	5/123	0.027250177	0.152600992	0	0	2.967182957	10.68985597	LAPTM4B;CD63;LAMP1;HEXB;ATP6V0C
Prion diseases	12816	0.082834795	0.335698905	0	0	4.17101147	10.389602	SOD1;HSPA1A
Non-homologous end-joining	41275	0.164215916	0.491053419	0	0	5.614823133	10.14358875	XRCC5
Tyrosine metabolism	13181	0.086942106	0.347768424	0	0	4.055150041	9.904756005	ALDH1A3;MIF
Purine metabolism	5/129	0.032529819	0.172744556	0	0	2.829174447	9.69161462	PNP;PKM;RRM2;NME1;PAPSS1
RNA transport	6/165	0.026366736	0.156172206	0	0	2.654280027	9.65003865	CYFIP1;POP5;PABPC4;EIF3I;EIF3D;EIF1
cGMP-PKG signaling pathway	6/166	0.027053656	0.154306038	0	0	2.638290388	9.524051866	PPIF;VDAC2;VDAC1;ATP1B1;SLC25A5;MYL9
Central carbon metabolism in cancer	23802	0.059755688	0.274697788	0	0	3.36889388	9.491827851	LDHA;PKM;PGAM1
Aldosterone-regulated sodium reabsorption	13547	0.09110921	0.350770459	0	0	3.945551391	9.452343186	SFN;ATP1B1
Human papillomavirus infection	10/330	0.01601388	0.098645501	0	0	2.211900022	9.144656999	BCAP31;CCNA2;PKM;CDK4;PSMC1;HLA-B;ISG15;HLA-A;ATP6V0C;ATP6V1F
Allograft rejection	13912	0.095333713	0.3625035	0	0	3.841721091	9.02947283	HLA-B;HLA-A
HIF-1 signaling pathway	4/100	0.048731876	0.242087386	0	0	2.919708029	8.821669832	LDHA;PGK1;TIMP1;ALDOA
Pyruvate metabolism	14277	0.099613273	0.374157171	0	0	3.743215422	8.633576125	LDHA;PKM
Regulation of actin cytoskeleton	7/214	0.028438104	0.156409572	0	0	2.387611706	8.499958189	CYFIP1;CFL1;PFN1;EZR;MYL9;PFN2;ACTG1
Chagas disease (American trypanosomiasis)	4/103	0.053264632	0.256336042	0	0	2.83466799	8.312614929	JUN;CXCL8;IRAK1;FOS
Glucagon signaling pathway	4/103	0.053264632	0.25239241	0	0	2.83466799	8.312614929	LDHA;PKM;PRMT1;PGAM1
RIG-I-like receptor signaling pathway	25628	0.07132039	0.30938986	0	0	3.128258603	8.260395267	CXCL8;IRF7;ISG15
Bladder cancer	15008	0.108328467	0.392531386	0	0	3.560619548	7.913787811	CXCL8;CDK4
Graft-versus-host disease	15008	0.108328467	0.387967068	0	0	3.560619548	7.913787811	HLA-B;HLA-A

p53 signaling pathway	26359	0.076207024	0.321531006	0	0	3.04136253	7.829384548	RRM2;CDK4;SFN
Leishmaniasis	27089	0.081237245	0.338122586	0	0	2.959163543	7.428629288	JUN;IRAK1;FOS
Metabolism of xenobiotics by cytochrome P450	27089	0.081237245	0.333614285	0	0	2.959163543	7.428629288	ALDH1A3;GSTO1;GSTP1
Type I diabetes mellitus	15738	0.117237108	0.401211435	0	0	3.395009336	7.27739546	HLA-B;HLA-A
Leukocyte transendothelial migration	4/112	0.06818326	0.300006344	0	0	2.606882169	7.000928572	CLDN7;EZR;MYL9;ACTG1
Vasopressin-regulated water reabsorption	16103	0.121758664	0.412106246	0	0	3.317850033	6.986444458	ARHGDIA;DYNLL1
Selenocompound metabolism	42736	0.20911606	0.590896758	0	0	4.293688278	6.719046237	PAPSS1
Hepatitis C	5/155	0.062432043	0.282780432	0	0	2.354603249	6.530907984	OAS1;CDK4;CLDN7;IRF7;YWHAZ
Proteoglycans in cancer	6/201	0.058895521	0.274845766	0	0	2.178886589	6.170585533	CD63;SDC4;PLAU;FLNA;EZR;ACTG1
Other glycan degradation	43101	0.219960372	0.615889043	0	0	4.055150041	6.140745637	HEXB
Chemical carcinogenesis	30011	0.102716803	0.381165966	0	0	2.670464661	6.077388909	ALDH1A3;GSTO1;GSTP1
Cell cycle	4/124	0.09105407	0.354995615	0	0	2.354603249	5.642339939	CCNA2;CDK4;SFN;YWHAZ
Glycosaminoglycan degradation	43466	0.230656527	0.628692125	0	0	3.841721091	5.635134741	HEXB
Cholesterol metabolism	18295	0.149703834	0.470497766	0	0	2.919708029	5.544806929	VDAC2;VDAC1
One carbon pool by folate	43831	0.241206539	0.646014036	0	0	3.649635036	5.1901522	TYMS
GABAergic synapse	32568	0.123143769	0.412263922	0	0	2.460428114	5.153127415	GNG5;GNB2;GABARAP
Relaxin signaling pathway	4/130	0.103698373	0.380227368	0	0	2.245929253	5.089879514	JUN;GNG5;GNB2;FOS
Autoimmune thyroid disease	19391	0.164117376	0.495570116	0	0	2.754441537	4.977753477	HLA-B;HLA-A
Human cytomegalovirus infection	6/225	0.089897086	0.354978237	0	0	1.946472019	4.689225794	CXCL8;GNG5;CDK4;GNB2;HLA-B;HLA-A
NF-kappa B signaling pathway	34759	0.141721442	0.459475834	0	0	2.305032655	4.503784455	CXCL8;IRAK1;PLAU
Circadian entrainment	35490	0.148111785	0.475191977	0	0	2.257506208	4.311358234	GNG5;GNB2;FOS
Prostate cancer	35490	0.148111785	0.470293091	0	0	2.257506208	4.311358234	HSP90AA1;PLAU;GSTP1
Proximal tubule bicarbonate reclamation	44927	0.271999491	0.686687239	0	0	3.173595684	4.131879037	ATP1B1
AGE-RAGE signaling pathway in diabetic complications	3/100	0.157867928	0.491144666	0	0	2.189781022	4.042328083	JUN;CXCL8;CDK4
Cell adhesion molecules (CAMs)	4/145	0.138516685	0.458743429	0	0	2.013591744	3.980396666	SDC4;CLDN7;HLA-B;HLA-A
T cell receptor signaling pathway	3/101	0.161163333	0.49146838	0	0	2.168100022	3.95751306	JUN;CDK4;FOS
MAPK signaling pathway	7/295	0.112044721	0.396664071	0	0	1.732030187	3.791166729	JUN;IRAK1;FLNA;HSPB1;FOS;EPHA2;HSPA1A

Focal adhesion	5/199	0.138727611	0.454554301	0	0	1.833987456	3.622570699	JUN;CAPN2;FLNA;MYL9;ACTG1
Oxytocin signaling pathway	4/153	0.158768364	0.48900656	0	0	1.908305901	3.511872472	JUN;FOS;MYL9;ACTG1
Th17 cell differentiation	3/107	0.181353746	0.537086094	0	0	2.04652432	3.494042753	JUN;HSP90AA1;FOS
Pathways in cancer	11/530	0.113671648	0.397850767	0	0	1.514942845	3.294154244	JUN;CASP7;HSP90AA1;CXCL8;GNG5;GSTO1;CDK4;GSTP1;GNB2;CKS2;FOS
TNF signaling pathway	3/110	0.191693985	0.562302356	0	0	1.99071002	3.288364313	JUN;CASP7;FOS
Aminoacyl-tRNA biosynthesis	24139	0.22873797	0.629029418	0	0	2.211900022	3.262946617	KARS;IARS
Cholinergic synapse	3/112	0.198668714	0.577263812	0	0	1.955161627	3.159769147	GNG5;GNB2;FOS
Toxoplasmosis	3/113	0.202178918	0.57658432	0	0	1.937859311	3.097866242	IRAK1;PPIF;HSPA1A
Amphetamine addiction	24869	0.238865797	0.645356715	0	0	2.146844139	3.073966087	JUN;FOS
B cell receptor signaling pathway	25965	0.254095737	0.66323294	0	0	2.056132415	2.816992221	JUN;FOS
Hepatocellular carcinoma	4/168	0.199374917	0.573901629	0	0	1.737921446	2.802516898	CDK4;GSTO1;GSTP1;ACTG1
Neurotrophin signaling pathway	3/119	0.223528289	0.620240658	0	0	1.840152119	2.756947741	JUN;IRAK1;ARHGDIA
Nicotinate and nicotinamide metabolism	10959	0.339093515	0.791218202	0	0	2.433090024	2.631336628	PNP
Gastric acid secretion	27426	0.274429381	0.68718902	0	0	1.946472019	2.516907667	ATP1B1;EZR
Osteoclast differentiation	3/127	0.252626419	0.665033651	0	0	1.724237025	2.372280277	FOSL1;JUN;FOS
Propanoate metabolism	11689	0.357105556	0.808739054	0	0	2.281021898	2.348822686	LDHA
Autophagy	3/128	0.25630391	0.663374826	0	0	1.710766423	2.329022679	VAMP8;LAMP1;GABARAP
Synaptic vesicle cycle	28522	0.289665906	0.719492733	0	0	1.871607711	2.318972618	ATP6V0C;ATP6V1F
RNA degradation	28887	0.294737334	0.714796055	0	0	1.847916474	2.257545434	LSM7;PABPC4
Base excision repair	12055	0.36592731	0.805040081	0	0	2.211900022	2.223668597	PARP4
Dopaminergic synapse	3/131	0.267379174	0.680601535	0	0	1.671588566	2.204971582	GNG5;GNB2;FOS
Endocytosis	5/244	0.243638763	0.646902923	0	0	1.495752064	2.112104567	UBB;HLA-B;CHMP2A;HLA-A;HSPA1A
Pentose and glucuronate interconversions	12420	0.374628447	0.818337317	0	0	2.146844139	2.107815697	DCXR
SNARE interactions in vesicular transport	12420	0.374628447	0.812574379	0	0	2.146844139	2.107815697	VAMP8
Calcium signaling pathway	4/188	0.257604739	0.661185497	0	0	1.553036186	2.106427844	PPIF;VDAC2;VDAC1;SLC25A5
Apelin signaling pathway	3/137	0.289683421	0.71377995	0	0	1.598380308	1.98033982	GNG5;GNB2;GABARAP
Ubiquitin mediated proteolysis	3/137	0.289683421	0.70811503	0	0	1.598380308	1.98033982	UBE2C;UBE2S;UBE2N
Colorectal cancer	31444	0.330050393	0.781965546	0	0	1.697504668	1.881700782	JUN;FOS
Gap junction	32174	0.340057634	0.78750189	0	0	1.658925017	1.789383151	TUBB4B;TUBA4A

Protein digestion and absorption	32905	0.350018455	0.804520031	0	0	1.622060016	1.702788965	PRSS3;ATP1B1
Breast cancer	3/147	0.327105749	0.780996672	0	0	1.489646954	1.664638416	JUN;CDK4;FOS
Morphine addiction	33270	0.354979922	0.809880119	0	0	1.604235181	1.661496828	GNG5;GNB2
Rap1 signaling pathway	4/206	0.312501605	0.751956987	0	0	1.417333995	1.648565905	PFN1;EPHA2;PFN2;ACTG1
Th1 and Th2 cell differentiation	33635	0.35992798	0.80331752	0	0	1.586797842	1.621471476	JUN;FOS
Small cell lung cancer	34001	0.364862063	0.808471335	0	0	1.5697355	1.582663692	CDK4;CKS2
Glycine, serine and threonine metabolism	14611	0.42439281	0.883195847	0	0	1.824817518	1.564043457	PGAM1
cAMP signaling pathway	4/212	0.33107446	0.778404073	0	0	1.377220768	1.52239633	JUN;FOS;ATP1B1;MYL9
Amoebiasis	35096	0.37957511	0.817546391	0	0	1.520681265	1.473088174	CXCL8;HSPB1
Tryptophan metabolism	15342	0.440087944	0.903647244	0	0	1.737921446	1.426452381	AOC1
Pancreatic secretion	35827	0.389304403	0.832678862	0	0	1.489646954	1.405323572	PRSS3;ATP1B1
Choline metabolism in cancer	36192	0.394143795	0.837215785	0	0	1.474600015	1.372910823	JUN;FOS
Progesterone-mediated oocyte maturation	36192	0.394143795	0.83148143	0	0	1.474600015	1.372910823	CCNA2;HSP90AA1
Carbohydrate digestion and absorption	16072	0.455356635	0.916665645	0	0	1.658925017	1.305033765	ATP1B1
Longevity regulating pathway	2/102	0.408555643	0.856021347	0	0	1.431229426	1.281132333	SOD1;HSPA1A
PI3K-Akt signaling pathway	6/354	0.357254436	0.803170558	0	0	1.237164419	1.273422052	HSP90AA1;GNG5;CDK4;GNB2;YWHAZ;EPHA2
Glycosphingolipid biosynthesis	16438	0.462834685	0.91969731	0	0	1.622060016	1.249611257	HEXB
Type II diabetes mellitus	16803	0.47021043	0.922451034	0	0	1.586797842	1.197357921	PKM
Parathyroid hormone synthesis, secretion and action	2/106	0.427508223	0.883708274	0	0	1.377220768	1.170337081	NACA;FOS
Amino sugar and nucleotide sugar metabolism	17533	0.484660561	0.93297158	0	0	1.520681265	1.101439336	HEXB
Endocrine and other factor-regulated calcium reabsorption	17533	0.484660561	0.927176725	0	0	1.520681265	1.101439336	ATP1B1
Valine, leucine and isoleucine degradation	17533	0.484660561	0.921453412	0	0	1.520681265	1.101439336	HSD17B10
Cocaine addiction	17899	0.49173768	0.92917304	0	0	1.489646954	1.057366118	JUN
Malaria	17899	0.49173768	0.923507351	0	0	1.489646954	1.057366118	CXCL8
Ovarian steroidogenesis	17899	0.49173768	0.917910336	0	0	1.489646954	1.057366118	HSD17B2
Serotonergic synapse	2/113	0.459887952	0.919775905	0	0	1.291906208	1.003517086	GNG5;GNB2
Tuberculosis	3/179	0.445213538	0.90811768	0	0	1.223341353	0.989929354	LAMP1;IRAK1;ATP6V0C
Glutamatergic synapse	2/114	0.464426956	0.916945528	0	0	1.280573697	0.98213726	GNG5;GNB2

Amyotrophic lateral sclerosis (ALS)	18629	0.505602719	0.93810625	0	0	1.431229426	0.976104276	SOD1
Axon guidance	3/181	0.45236874	0.91664192	0	0	1.209823769	0.959701942	CFL1;MYL9;EPHA2
Thyroid hormone signaling pathway	2/116	0.473437328	0.922903145	0	0	1.25849484	0.941021564	ATP1B1;ACTG1
Chemokine signaling pathway	3/190	0.484076169	0.937707296	0	0	1.152516327	0.83616559	CXCL8;GNG5;GNB2
Platelet activation	2/124	0.50854556	0.937916362	0	0	1.177301625	0.796091913	VAMP8;ACTG1
VEGF signaling pathway	21551	0.557394719	1	0	0	1.237164419	0.723099887	HSPB1
Steroid hormone biosynthesis	21916	0.563476335	1	0	0	1.216545012	0.697846646	HSD17B2
Natural killer cell mediated cytotoxicity	2/131	0.537989392	0.986313885	0	0	1.114392378	0.690830152	HLA-B;HLA-A
FoxO signaling pathway	2/132	0.54209563	0.987961267	0	0	1.105950011	0.677187407	PRMT1;GABARAP
Inflammatory bowel disease (IBD)	23743	0.592657944	1	0	0	1.122964627	0.587465322	JUN
Non-small cell lung cancer	24108	0.598256709	1	0	0	1.105950011	0.568165603	CDK4
Renal cell carcinoma	25204	0.614597117	1	0	0	1.057865228	0.514956438	JUN
Prolactin signaling pathway	25569	0.619895398	1	0	0	1.042752868	0.498649144	FOS
Adherens junction	26299	0.630275223	1	0	0	1.01378751	0.46796299	ACTG1
Arrhythmogenic right ventricular cardiomyopathy (ARVC)	26299	0.630275223	1	0	0	1.01378751	0.46796299	ACTG1
Bile secretion	26299	0.630275223	1	0	0	1.01378751	0.46796299	ATP1B1
Melanoma	26299	0.630275223	1	0	0	1.01378751	0.46796299	CDK4
Ras signaling pathway	3/232	0.618718379	1	0	0	0.94387113	0.453157315	GNG5;GNB2;EPHA2
Bacterial invasion of epithelial cells	27030	0.640372603	1	0	0	0.986387848	0.439638074	ACTG1
Inositol phosphate metabolism	27030	0.640372603	1	0	0	0.986387848	0.439638074	TPI1
PPAR signaling pathway	27030	0.640372603	1	0	0	0.986387848	0.439638074	FABP5
Thyroid hormone synthesis	27030	0.640372603	1	0	0	0.986387848	0.439638074	ATP1B1
Glioma	27395	0.645317777	1	0	0	0.97323601	0.426289446	CDK4
Pancreatic cancer	27395	0.645317777	1	0	0	0.97323601	0.426289446	CDK4
Chronic myeloid leukemia	27760	0.650195196	1	0	0	0.960430273	0.413448578	CDK4
Wnt signaling pathway	2/158	0.639852548	1	0	0	0.923958237	0.412563544	FOSL1;JUN
Hippo signaling pathway	2/160	0.646652211	1	0	0	0.912408759	0.39776156	YWHAZ;ACTG1
Complement and coagulation cascades	28856	0.664430104	1	0	0	0.923958237	0.377737773	PLAU

Herpes simplex virus 1 infection	6/492	0.669528681	1	0	0	0.890154887	0.357113472	IRAK1;OAS1;HLA-B;IRF7;HLA-A;SRSF9
ECM-receptor interaction	29952	0.678087764	1	0	0	0.890154887	0.345806083	SDC4
Peroxisome	30317	0.682516099	1	0	0	0.879430129	0.335915191	SOD1
ErbB signaling pathway	31048	0.691191502	1	0	0	0.858737656	0.317164754	JUN
Hypertrophic cardiomyopathy (HCM)	31048	0.691191502	1	0	0	0.858737656	0.317164754	ACTG1
Insulin secretion	31413	0.695440216	1	0	0	0.848752334	0.30827553	ATP1B1
Alcoholism	2/180	0.709149181	1	0	0	0.811030008	0.278742388	GNG5;GNB2
Salivary secretion	32874	0.711860521	1	0	0	0.811030008	0.275647433	ATP1B1
Dilated cardiomyopathy (DCM)	33239	0.715825856	1	0	0	0.80211759	0.268162637	ACTG1
Fc gamma R-mediated phagocytosis	33239	0.715825856	1	0	0	0.80211759	0.268162637	CFL1
mRNA surveillance pathway	33239	0.715825856	1	0	0	0.80211759	0.268162637	PABPC4
GnRH signaling pathway	33970	0.723594147	1	0	0	0.78486775	0.253924036	JUN
Transcriptional misregulation in cancer	2/186	0.726010828	1	0	0	0.78486775	0.25130708	CXCL8;PLAU
Glycerophospholipid metabolism	35431	0.738501658	1	0	0	0.752502069	0.228107406	PTDSS1
Aldosterone synthesis and secretion	35796	0.742101625	1	0	0	0.744823477	0.222157816	ATP1B1
C-type lectin receptor signaling pathway	1/104	0.762687717	1	0	0	0.701852892	0.190136591	JUN
AMPK signaling pathway	1/120	0.809925298	1	0	0	0.608272506	0.12823191	CCNA2
Oocyte meiosis	1/125	0.822669016	1	0	0	0.583941606	0.113986176	YWHAZ
Vascular smooth muscle contraction	1/132	0.839092509	1	0	0	0.552975006	0.097010793	MYL9
Systemic lupus erythematosus	1/133	0.841311564	1	0	0	0.548817299	0.094831908	SNRPB
Adrenergic signaling in cardiomyocytes	1/145	0.865677206	1	0	0	0.503397936	0.07261172	ATP1B1
Phospholipase D signaling pathway	1/148	0.871162001	1	0	0	0.493193924	0.068024919	CXCL8
mTOR signaling pathway	1/152	0.878129467	1	0	0	0.480215136	0.062409354	ATP6V1F
Cushing syndrome	1/155	0.883107522	1	0	0	0.47092065	0.058539353	CDK4
MicroRNAs in cancer	2/299	0.918238254	1	0	0	0.48824549	0.041646553	PLAU;EZR
Cytokine-cytokine receptor interaction	1/294	0.983189119	1	0	0	0.248274492	0.004209193	CXCL8
Neuroactive ligand-receptor interaction	1/338	0.990926197	1	0	0	0.215954736	0.001968475	PRSS3

Supplementary Table 9

Co-upregulated genes_single gene perturbation

Term	Overlap	P-value	Adjusted P-value	Old P-value	Old Adjusted P-value	Odds Ratio	Combined Score	Genes
laudanosine-1741	12/100	1.24E-08	7.55E-05	0	0	8.759124	159.4806	CCT3;VAMP8;POP5;UBL5;IRAK1;GNG5;RPL34;CFL1;PSME1;CHMP2A;ERH;SOD1
monastrol-614	11/100	1.26E-07	3.84E-04	0	0	8.029197	127.5701	CHCHD2;SDC4;NDUFB11;UBE2S;RPL34;ADRM1;CIB1;PHLDA2;CCT7;NOP10;RHOB
baclofen-2036	10/100	1.16E-06	0.002353	0	0	7.29927	99.77644	VAMP8;PSMB6;POP5;MTCH1;PTDSS1;FABP5;RPL34;CFL1;PSME1;SOD1
niclosamide-1998	9/100	9.56E-06	0.014574	0	0	6.569343	75.93012	IFITM3;BCAP31;TM9SF2;PRDX4;RRM2;CDK4;ODC1;GCLM;DYNLL1
harmine-1758	9/100	9.56E-06	0.011659	0	0	6.569343	75.93012	MT2A;DCXR;IRF7;MT1X;MT1H;S100P;PHLDA2;CRIP1;MT1E
midecamycin-2026	8/100	7.02E-05	0.071382	0	0	5.839416	55.84814	XRCC5;PRMT1;RPL34;RPL36;VDAC2;POLR2G;RPL26;GABARAP
harmol-1750	8/100	7.02E-05	0.061185	0	0	5.839416	55.84814	MT2A;IRF7;PLIN3;MT1X;MT1H;ISG15;S100P;MT1E
nocodazole-1393	8/100	7.02E-05	0.053537	0	0	5.839416	55.84814	MRPL3;EMG1;ODC1;HSPB1;POLR2F;STRA13;HSPE1;NME1
ambroxol-6719	8/100	7.02E-05	0.047588	0	0	5.839416	55.84814	MT2A;LSM7;POLR2F;S100A4;IGFBP6;PFN1;MYL9;MLF2
AG-012559-6920	8/100	7.02E-05	0.042829	0	0	5.839416	55.84814	GPX4;UBE2S;PRMT1;SH3BGRL3;CYC1;POLR2J;ATP6V1F;MLF2

Co-downregulated genes_single gene perturbation

Term	Overlap	P-value	Adjusted P-value	Old P-value	Old Adjusted P-value	Odds Ratio	Combined Score	Genes
disulfiram-6210	13/100	1.11E-09	6.76E-06	0	0	9.489051	195.6664	JUN;MT1X;NDRG1;FOSL1;DNAJB1;MT2A;HSPH1;MAFF;SERPINH1;MT1H;EPHA2;MT1E;HSPA1A
ciclopirox-2456	13/100	1.11E-09	3.38E-06	0	0	9.489051	195.6664	JUN;MT1X;FOS;EIF1;RHOB;FOSL1;DNAJB1;ZFP36;MT2A;MAFF;MT1H;PHLDA2;MT1E

15-delta prostaglandin J2-1011	12/100	1.24E-08	2.52E-05	0	0	8.759124	159.4806	FOSL1;DNAJB1;JUN;HSPH1;UBB;MAFF;SERPINH1;UPP1;GCLM;NDRG1;TUBA4A;HSPA1A
disulfiram-1369	12/100	1.24E-08	1.89E-05	0	0	8.759124	159.4806	DNAJB1;MT2A;HSP90AA1;HSPH1;FTH1;HSPB1;MT1X;MT1H;GCLM;MT1E;S100A10;HSPA1A
1,4-chrysenequinone-7139	12/100	1.24E-08	1.51E-05	0	0	8.759124	159.4806	DNAJB1;ZFP36;JUN;HSPH1;TUFT1;MAFF;SERPINH1;HSPB1;FOS;KRT6A;RHOB;HSPA1A
phenoxybenzamine-5248	11/100	1.26E-07	0.000128	0	0	8.029197	127.5701	FOSL1;DNAJB1;MT2A;JUN;MAFF;SERPINH1;MT1X;MT1H;FOS;EPHA2;MT1E
15-delta prostaglandin J2-1656	11/100	1.26E-07	0.00011	0	0	8.029197	127.5701	FOSL1;DNAJB1;JUN;HSPH1;UBB;MAFF;SERPINH1;GCLM;NDRG1;TUBA4A;HSPA1A
MG-262-7068	11/100	1.26E-07	9.6E-05	0	0	8.029197	127.5701	DNAJB1;JUN;CASP7;HSPH1;UBB;SERPINH1;HSPB1;TMEM97;FOS;KRT6A;HSPA1A
tanespimycin-521	11/100	1.26E-07	8.53E-05	0	0	8.029197	127.5701	IFITM3;DNAJB1;HSP90AA1;LGALS1;HSPH1;SERPINH1;CKS2;MT1X;S100A4;HSPE1;HSPA1A
15-delta prostaglandin J2-2691	11/100	1.26E-07	7.68E-05	0	0	8.029197	127.5701	DNAJB1;HSPH1;TUFT1;FTH1;MAFF;SERPINH1;HSPB1;ATP1B1;GCLM;RHOB;HSPA1A

Supplementary Table 10

Co-upregulated genes (Biological Process)

Term	Overlap	P-value	Adjusted P-value	Old P-value	Old Adjusted P-value	Odds Ratio	Combined Score	Genes
SRP-dependent cotranslational protein targeting to membrane (GO:0006614)	18/89	5.868564312959966E-17	2.9947283689034704E-13	0	0	15.862524785194978	592.8513399718037	RPL4;RPL3;RPS7;RPL23;RPL12;RPL36A;RPL6;RPS4X;RPL7A;RPS16;RPL24;RPL13;SEC61B;RPS2;RPL29;RPS27A;RPS21;RPS23
cotranslational protein targeting to membrane (GO:0006613)	18/93	1.347410121984525E-16	3.437916926243516E-13	0	0	15.180265654648958	554.7351372471227	RPL4;RPL3;RPS7;RPL23;SSR2;RPL12;RPL36A;RPL6;RPS4X;RPL7A;RPS16;RPL24;RPL13;RPS2;RPL29;RPS27A;RPS21;RPS23
protein targeting to ER (GO:0045047)	18/97	2.9698047665210095E-16	5.051637907852237E-13	0	0	14.554275318374774	520.3570445956904	RPL4;RPL3;RPS7;RPL23;RPL12;RPL36A;RPL6;RPS4X;RPL7A;RPS16;SEC61G;RPL24;RPL13;RPS2;RPL29;RPS27A;RPS21;RPS23

aminoglycoside antibiotic metabolic process (GO:0030647)	4/8	1.7355761511813577E-6	3.4064019613378724E-4	0	0	39.21568627450981	520.1635734933923	AKR1B10;AKR1C1;AKR1C3;AKR1C2
daunorubicin metabolic process (GO:0044597)	4/8	1.7355761511813577E-6	3.280238925732766E-4	0	0	39.21568627450981	520.1635734933923	AKR1B10;AKR1C1;AKR1C3;AKR1C2
doxorubicin metabolic process (GO:0044598)	4/8	1.7355761511813577E-6	3.1630875355280247E-4	0	0	39.21568627450981	520.1635734933923	AKR1B10;AKR1C1;AKR1C3;AKR1C2
L-ascorbic acid metabolic process (GO:0019852)	4/9	3.092735206187276E-6	5.442147502473679E-4	0	0	34.85838779956427	442.2293569882183	CYB5R3;GSTO1;SLC2A1;SLC2A3
quinone metabolic process (GO:1901661)	4/10	5.102925455874272E-6	8.400073742363359E-4	0	0	31.372549019607845	382.2963627887528	AKR1B10;AKR1C1;AKR1C3;AKR1C2
viral gene expression (GO:0019080)	17/110	4.8878966631987034E-14	3.5632766674718544E-11	0	0	12.121212121212121	371.5082329840923	RPL4;RPL3;RPS7;RPL23;RPL12;RPL36A;RPL6;RPS4X;RP
nuclear-transcribed mRNA catabolic process, nonsense-mediated decay (GO:0000184)	17/112	6.652432551022984E-14	4.2434204134837864E-11	0	0	11.904761904761905	361.20486567845273	RPL4;RPL3;RPS7;RPL23;RPL12;RPL36A;RPL6;RPS4X;RP

Co-upregulated genes (Cellular Component)

Term	Overlap	P-value	Adjusted P-value	Old P-value	Old Adjusted P-value	Odds Ratio	Combined Score	Genes
cytosolic ribosome (GO:0022626)	19/124	1.79208128143945E-15	7.992682515219947E-13	0	0	12.017710309930425	408.0661453164889	RPL4;RPL3;RPS7;RPL23;RPL12;RPL36A;RPL6;RPS4X;RPL7A;RPS16;RPL36AL;NHP2;RPL24;RPL13;RPS2;RPL29;RPS27A;RPS21;RPS23
cytosolic large ribosomal subunit (GO:0022625)	12/69	6.596933338018798E-11	7.3555806718909595E-9	0	0	13.640238704177323	319.7521722439985	RPL4;RPL7A;RPL3;RPL36AL;RPL23;RPL12;NHP2;RPL36A;RPL24;RPL13;RPL29;RPL6
large ribosomal subunit (GO:0015934)	12/72	1.1115314852688792E-10	9.914860848598403E-9	0	0	13.071895424836601	299.6093091384567	RPL4;RPL7A;RPL3;RPL36AL;RPL23;RPL12;NHP2;RPL36A;RPL24;RPL13;RPL29;RPL6
cytosolic part (GO:0044445)	19/159	1.8961866097311505E-13	4.228496139700466E-11	0	0	9.372302380071526	274.5499895693678	RPL4;RPL3;RPS7;RPL23;RPL12;RPL36A;RPL6;RPS4X;RPL7A;RPS16;RPL36AL;NHP2;RPL24;RPL13;RPS2;RPL29;RPS27A;RPS21;RPS23
polysomal ribosome (GO:0042788)	6/28	1.2059173127580105E-6	4.481992679083939E-5	0	0	16.80672268907563	229.04655504586123	RPL7A;RPL36A;RPL24;RPS21;RPL6;RPS23
pseudopodium (GO:0031143)	3/9	1.6256406745380933E-4	0.0029001429633759585	0	0	26.143790849673202	228.08989209518475	RAB25;MSN;F2RL1
ribosome (GO:0005840)	11/76	3.216598664744497E-9	2.0494328635372082E-7	0	0	11.351909184726523	221.98591832899	RPS4X;RPL7A;RPS7;RPL23;RPL12;RPL36A;RPL24;RPL13;RPS21;RPL6;RPS23
mitochondrial respiratory chain complex IV (GO:0005751)	4/17	5.390284754173382E-5	0.0012652984212428045	0	0	18.45444059976932	181.37628145526102	NDUFA4;UQCRFS1;COX5B;COX7C

ficolin-1-rich granule (GO:0101002)	17/184	2.282537138763403E-10	1.6966859398141296E-8	0	0	7.246376811594203	160.87364728620025	GPI;CSTB;PSMD11;JUP;DYNLT1;COPB1;SLC2A3;EEF2;DYNLL1;RHOA;LGALS3;FTH1;QPCT;LTA4H;ALDOA;PGM1;CD55
phagocytic vesicle membrane (GO:0030670)	6/38	7.9419326793496E-6	2.0835893970528953E-4	0	0	12.38390092879257	145.4285312919793	VAMP8;ATP6V0B;ATP6V0E1;HLA-B;HLA-C;ATP6V0D1

Co-upregulated genes (Molecular Function)

Term	Overlap	P-value	Adjusted P-value	Old P-value	Old Adjusted P-value	Odds Ratio	Combined Score	Genes
ketosteroid monooxygenase activity (GO:0047086)	3/6	3.982105923372529E-5	0.0045834039178017805	0	0	39.21568627450981	397.29861408378144	AKR1C1;AKR1C3;AKR1C2
alditol:NADP+ 1-oxidoreductase activity (GO:0004032)	4/12	1.178866548435601E-5	0.001696094246561721	0	0	26.143790849673202	296.6894651056666	AKR1B10;AKR1C1;AKR1C3;AKR1C2
bile acid binding (GO:0032052)	3/8	1.0940663990166563E-4	0.011447912956983376	0	0	29.411764705882355	268.24820517702017	AKR1C1;AKR1C3;AKR1C2
cadherin binding (GO:0045296)	28/313	5.916104816098611E-16	6.809436643329501E-13	0	0	7.016225020359582	246.01469155495982	RAB1A;BZW2;NDRG1;RPL6;RTN4;SDCBP;RPL7A;LASP1;PCBP1;RARS;SFN;RPS2;CLIC1;S100A11;JUP;ANXA2;HSPA5;EEF2;YWHAZ;EEF1G;EIF5;GPRC5A;KRT18;RPL24;S100P;FXYD5;RPL29;ALDOA
aldo-keto reductase (NADP) activity (GO:0004033)	5/20	4.294780432138016E-6	8.238820462318094E-4	0	0	19.607843137254903	242.31588481514083	ALDH3A1;AKR1B10;AKR1C1;AKR1C3;AKR1C2
alcohol dehydrogenase (NADP+) activity (GO:0008106)	5/20	4.294780432138016E-6	7.061846110558366E-4	0	0	19.607843137254903	242.31588481514083	ALDH3A1;AKR1B10;AKR1C1;AKR1C3;AKR1C2
oxidoreductase activity, acting on NAD(P)H, quinone or similar compound as acceptor (GO:0016655)	4/15	3.1542240148594455E-5	0.004033902045670247	0	0	20.915032679738562	216.76722516736152	NQO1;AKR1C1;AKR1C3;AKR1C2
cytochrome-b5 reductase activity, acting on NAD(P)H (GO:0004128)	2/6	0.0023481615688298284	0.10395130637396663	0	0	26.143790849673202	158.27771419940805	NQO1;CYB5R3
aldehyde dehydrogenase [NAD(P)+] activity (GO:0004030)	2/6	0.0023481615688298284	0.10010125798974565	0	0	26.143790849673202	158.27771419940805	ALDH3A1;ALDH2
ubiquitin-protein transferase inhibitor activity (GO:0055105)	2/6	0.0023481615688298284	0.09652621306154044	0	0	26.143790849673202	158.27771419940805	RPS7;RPL23

Co-downregulated genes (Biological Process)

Term	Overlap	P-value	Adjusted P-value	Old P-value	Old Adjusted P-value	Odds Ratio	Combined Score	Genes
antigen processing and presentation of exogenous peptide antigen via MHC class I, TAP-independent (GO:0002480)	6/9	3.453835059133739E-10	8.392819193694986E-8	0	0	51.88067444876783	1130.2913465596098	HLA-B;HLA-C;HLA-A;HLA-F;B2M;HLA-G
cotranslational protein targeting to membrane (GO:0006613)	21/93	1.378540936194063E-20	3.517347198699152E-17	0	0	17.57248650684072	803.6016906955451	RPL4;RPL30;RPS9;ARL6IP1;RPL21;RPS8;RPLP1;RPS5;RPS6;RPS3A;RPL8;RPL9;SRP9;RPS26;RPS25;RPL27A;RPL36;RPL24;RPL27;RPS20;RPL19
nuclear-transcribed mRNA catabolic process, nonsense-mediated decay (GO:0000184)	23/112	1.984264889214852E-21	1.012570372966339E-17	0	0	15.981100611450804	761.8036993641952	RPL4;RPL30;RPS9;RPL21;RPS8;RPS5;RPLP1;EIF4A3;RPS6;RPS3A;RPL8;RPL9;PNRC2;RPS26;RPS25;RPL27A;RPL36;RPL24;RPL27;RPS20;EIF3E;PABPC1;RPL19
SRP-dependent cotranslational protein targeting to membrane (GO:0006614)	20/89	1.287764851367707E-19	1.642866009132352E-16	0	0	17.487867791719495	760.6559476183503	RPL4;RPL30;RPS9;RPL21;RPS8;RPLP1;RPS5;RPS6;RPS3A;RPL8;RPL9;SRP9;RPS26;RPS25;RPL27A;RPL36;RPL24;RPL27;RPS20;RPL19
protein targeting to ER (GO:0045047)	20/97	8.075619425808144E-19	8.241977185979792E-16	0	0	16.04556941714469	668.4627091602829	RPL4;RPL30;RPS9;RPL21;RPS8;RPS5;RPLP1;RPS6;RPS3A;RPL8;RPL9;SRP9;RPS26;RPS25;RPL27A;RPL36;RPL24;RPL27;RPS20;RPL19
cytoplasmic translation (GO:0002181)	13/54	1.3332537925642784E-13	3.779774501919729E-11	0	0	18.734687995388384	555.4082566936905	RPL4;RPL30;RPLP1;RPS3A;RPL8;RPL9;RPS26;RPL27A;RPL36;RPL24;RPL22L1;RPS20;RPL19
cellular response to type I interferon (GO:0071357)	14/65	7.987598444010929E-14	2.5475446787367356E-11	0	0	16.761448668063455	505.49681676794	IFITM3;IFITM1;IFITM2;HLA-B;IFI6;HLA-C;ISG15;HLA-A;HLA-F;HLA-G;PSMB8;BST2;IFI27;IRAK1
type I interferon signaling pathway (GO:0060337)	14/65	7.987598444010929E-14	2.3976891093992807E-11	0	0	16.761448668063455	505.49681676794	IFITM3;IFITM1;IFITM2;HLA-B;IFI6;HLA-C;ISG15;HLA-A;HLA-F;HLA-G;PSMB8;BST2;IFI27;IRAK1
positive regulation of establishment of protein localization (GO:1904951)	5/12	2.4793581614598014E-7	3.012420166173659E-5	0	0	32.425421530479895	493.1937720538401	CCT6A;CCT2;DKC1;CHORDC1;CCT7
viral gene expression (GO:0019080)	19/110	2.0472092549869403E-16	1.0446908828198356E-13	0	0	13.441811107180756	485.58386647593244	RPL4;RPL30;RPS9;RPL21;RPS8;RPS5;RPLP1;RPS6;RPS3A;RPL8;RPL9;RPS26;RPS25;RPL27A;RPL36;RPL24;RPL27;RPS20;RPL19

Co-downregulated genes (Cellular Component)

Term	Overlap	P-value	Adjusted P-value	Old P-value	Old Adjusted P-value	Odds Ratio	Combined Score	Genes
cytosolic ribosome (GO:0022626)	20/124	1.3110893136931503E-16	2.923729169535725E-14	0	0	12.551776076314798	459.02476665064415	RPL4;RPL30;RPS9;RPL21;RPS8;RPS5;RPLP1;RPS6;RPS3A;RPL8;RPL9;RPS26;RPS25;RPL27A;RPL36;RPL24;RPL27;RPS20;RPL22L1;RPL19
cytosolic part (GO:0044445)	23/159	7.849058169327927E-18	3.5006799435202554E-15	0	0	11.257127474732643	443.3747776412399	RPL4;CCT2;RPL30;RPS9;RPL21;RPS8;RPS5;RPLP1;RPS6;RPS3A;RPL8;RPL9;CCT6A;RPS26;RPS25;RPL27A;RPL36;RPL24;RPL27;RPS20;RPL22L1;CCT7;RPL19
cytosolic large ribosomal subunit (GO:0022625)	12/69	7.221940347510629E-11	8.052463487474352E-9	0	0	13.534088986635087	316.03873943417966	RPL4;RPL30;RPL21;RPLP1;RPL27A;RPL36;RPL24;RPL27;RPL22L1;RPL8;RPL9;RPL19
large ribosomal subunit (GO:0015934)	12/72	1.2165012041087613E-10	1.085119074065015E-8	0	0	12.970168612191957	296.1072899725743	RPL4;RPL30;RPL21;RPLP1;RPL27A;RPL36;RPL24;RPL27;RPL22L1;RPL8;RPL9;RPL19
polysomal ribosome (GO:0042788)	6/28	1.261980765938927E-6	4.690361846739679E-5	0	0	16.67593107281823	226.50630408388997	RPS26;RPL30;RPL36;RPL24;RPL8;RPL19
ribosome (GO:0005840)	11/76	3.4902874556531516E-9	2.594447008702176E-7	0	0	11.263567479008806	219.33862290055288	RPS26;RPS25;RPL30;RPS9;RPS5;RPL36;RPL24;RPL27;RPL8;RPL9;RPL19
integral component of lumenal side of endoplasmic reticulum membrane (GO:0071556)	6/29	1.5741466368914669E-6	5.400533846566109E-5	0	0	16.10089896685898	215.13694754385662	BCAP31;HLA-B;HLA-C;HLA-A;HLA-F;HLA-G
phagocytic vesicle membrane (GO:0030670)	7/38	4.78845881748512E-7	1.9415023932712392E-5	0	0	14.335449518738479	208.6078420831019	ATP6V0B;HLA-B;HLA-C;HLA-A;HLA-F;B2M;HLA-G
cytosolic small ribosomal subunit (GO:0022627)	8/49	1.9068454589935397E-7	1.0630663433888983E-5	0	0	12.705471293575794	196.58725153887588	RPS26;RPS25;RPS9;RPS8;RPS5;RPS6;RPS3A;RPS20
small ribosomal subunit (GO:0015935)	8/53	3.5850861000232726E-7	1.5989484006103797E-5	0	0	11.746567799721019	174.33449127614406	RPS26;RPS25;RPS9;RPS8;RPS5;RPS6;RPS3A;RPS20

Co-downregulated genes (Molecular Function)

Term	Overlap	P-value	Adjusted P-value	Old P-value	Old Adjusted P-value	Odds Ratio	Combined Score	Genes
MHC class II protein complex binding (GO:0023026)	6/16	3.0530181606323314E-8	1.1713413009626044E-5	0	0	29.182879377431906	504.99659771680666	YWHAE;HSPA8;HSP90AA1;HSP90AB1;PKM;CD81
MHC protein complex binding (GO:0023023)	6/19	1.001448627937522E-7	2.8816684268902194E-5	0	0	24.575056317837394	396.06753400622614	YWHAE;HSPA8;HSP90AA1;HSP90AB1;PKM;CD81
RNA binding (GO:0003723)	77/1387	3.1910004789570174E-29	3.672841551279527E-26	0	0	4.320272457702007	283.4730926337388	RPL4;TOP2A;YWHAE;EIF4A1;RPL30;HSP90AB1;EIF4A3;GDI2;HMGB2;MRPL37;YBX1;RPL8;RPL9;PPP1CC;FBL;PTBP1;MRPL3;TUBA1B;C1QBP;SUMO2;RPL36;NUSAP1;KPNA2;HMGN2;LBR;RPS9;HSP90AA1;RPL21;RPS8;RPS5;RPS6;RPS3A;

								HLA-A;SRP9;NME1;NSA2;DDX39A;PKM;KARS;HNRNPUL1;DKC1;EEF1D;SRSF2;RPL24;RPL27;ERH;PABPC1;MRPL16;DDX21;SLC3A2;STIP1;PUF60;TRA2B;RPL19;HSPA8;NPM1;DUT;NOP58;ALYREF;TBCA;DEK;EIF2S2;HSPE1;RPS26;CCT6A;BST2;RPS25;KRT18;RPL27A;EIF3H;RPS20;RPL22L1;EIF3E;SLIRP;RBMX;RAN;AARS
DNA polymerase binding (GO:0070182)	4/16	4.292405139860489E-5	0.008234263859965705	0	0	19.455252918287936	195.6435457289362 6	HSP90AA1;PCNA;HSP90AB1;PTGES3
phosphoserine residue binding (GO:0050815)	2/6	0.0023845737769834317	0.1372322208653965	0	0	25.940337224383914	156.64681920810733	YWHAE;YWHAB
poly(A) binding (GO:0008143)	3/14	6.8736790149438E-4	0.06085849650923319	0	0	16.67593107281823	121.44481751250139	EIF4A3;PABPC3;PABPC1
cadherin binding (GO:0045296)	21/313	7.660991999297058E-10	4.408900895595457E-7	0	0	5.221218035578871	109.59184954580664	YWHAE;HSPA8;HSP90AB1;YWHAB;AHSA1;SLC3A2;PAICS;RPS26;LDHA;ATIC;KRT18;PKM;PUF60;CDH1;EEF1D;KIF5B;RPL24;EIF3E;PROM1;S100A11;RAN
telomerase activity (GO:0003720)	2/8	0.004376212314776258	0.2289554715594306	0	0	19.455252918287936	105.67260110967956	DKC1;PTGES3
RNA-directed DNA polymerase activity (GO:0003964)	2/8	0.004376212314776258	0.21900088583945534	0	0	19.455252918287936	105.67260110967956	DKC1;PTGES3
peptidase activator activity (GO:0016504)	3/16	0.0010375794448162583	0.07464087131146958	0	0	14.591439688715953	100.25580839699609	ADRM1;PSME1;PSME2
